# Unveiling Repulsion in Intramolecular H-Bonded Systems

**DOI:** 10.1021/jacs.5c00385

**Published:** 2025-04-07

**Authors:** Ivan V. Smolyar, Scott L. Cockroft

**Affiliations:** EaStCHEM School of Chemistry, University of Edinburgh, Joseph Black Building, David Brewster Rd, Edinburgh, EH9 3FJ, United Kingdom

## Abstract

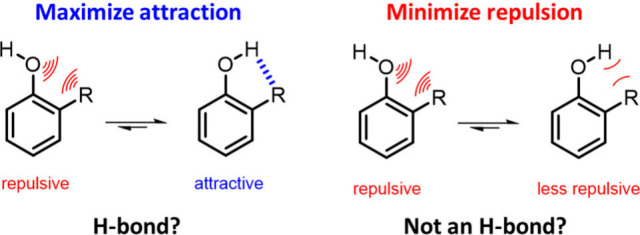

Intramolecular H-bonds govern molecular conformation and play critical roles in pharmaceutical design, catalysis, and supramolecular chemistry. Despite this, the experimental influence of *ortho*-substituents on the energetics of an adjacent H-bond defies classical Hammett analysis. By using synthetic molecular balances, we show that substituents positioned *ortho* to an OH H-bond donor can compete strongly with H-bonding to an external acceptor. Computational dissection of the experimental trends reveals that this competition is rarely dominated by stabilizing OH···R H-bonds, but rather by the avoidance of repulsive HO···R interactions. We provide a framework for rationalizing the influence of *ortho*-substituents on molecular conformation and the energetics of intramolecular H-bonds. Our work challenges the intuitive bias of attributing close contacts to attractive interactions and highlights the critical role of repulsion in molecular design.

Intramolecular H-bonds determine the structure and properties of many compounds. The manipulation of a molecule’s capacity to form intramolecular H-bonds has garnered considerable attention since it can determine bioactivity and physicochemical properties.^1−4^ Conformational freedom plays a major role in molecular design. Solvent-resistant intramolecular H-bonds have been identified that are so strong that they mimic rigid heteroaryl fragments.^5,6^ Such intramolecular H-bonds mask polar sites, which increases lipophilicity^7^ and membrane permeability.^8^ Conversely, solvent disruption of weak intramolecular H-bonds exposes polar groups, which enhances solubility and affects bioavailability.^1−4^ Similarly, the control of H-bond directionality is essential in foldamers,^9,10^ supramolecular materials,^11^ reagent and catalyst design,^12−14^ excited state stabilization,^15^ and studies of molecular interactions.^5,16−18^

While intramolecular H-bonding is known to compete with intermolecular interactions, the influence of adjacent substituents appears surprisingly perplexing. For example, only a weak correlation was found between the strength of intramolecular and intermolecular H-bonds in *ortho*-substituted phenols vs several measures of their H-bond donor ability.^19^ Indeed, parametrizing the effects of *ortho*-substituents is a long-standing challenge.^20^

Here we deconvolute the effects of *ortho* substituents on an H-bond donor in an experimental system. Competition between two intramolecular H-bonds was assessed using a series of synthetic molecular balances (Figure 1). The key energetic contributions determining the conformational preferences were dissected (Figures 2 and 3), and the sum of these components shown to account for the observed energetic trends (Figures 3 and 4). We reveal that minimization of repulsion often dominates over attraction in determining preferred intramolecular contacts, which ultimately governs the conformations of molecules and the interactions that they form.

**Figure 1:**
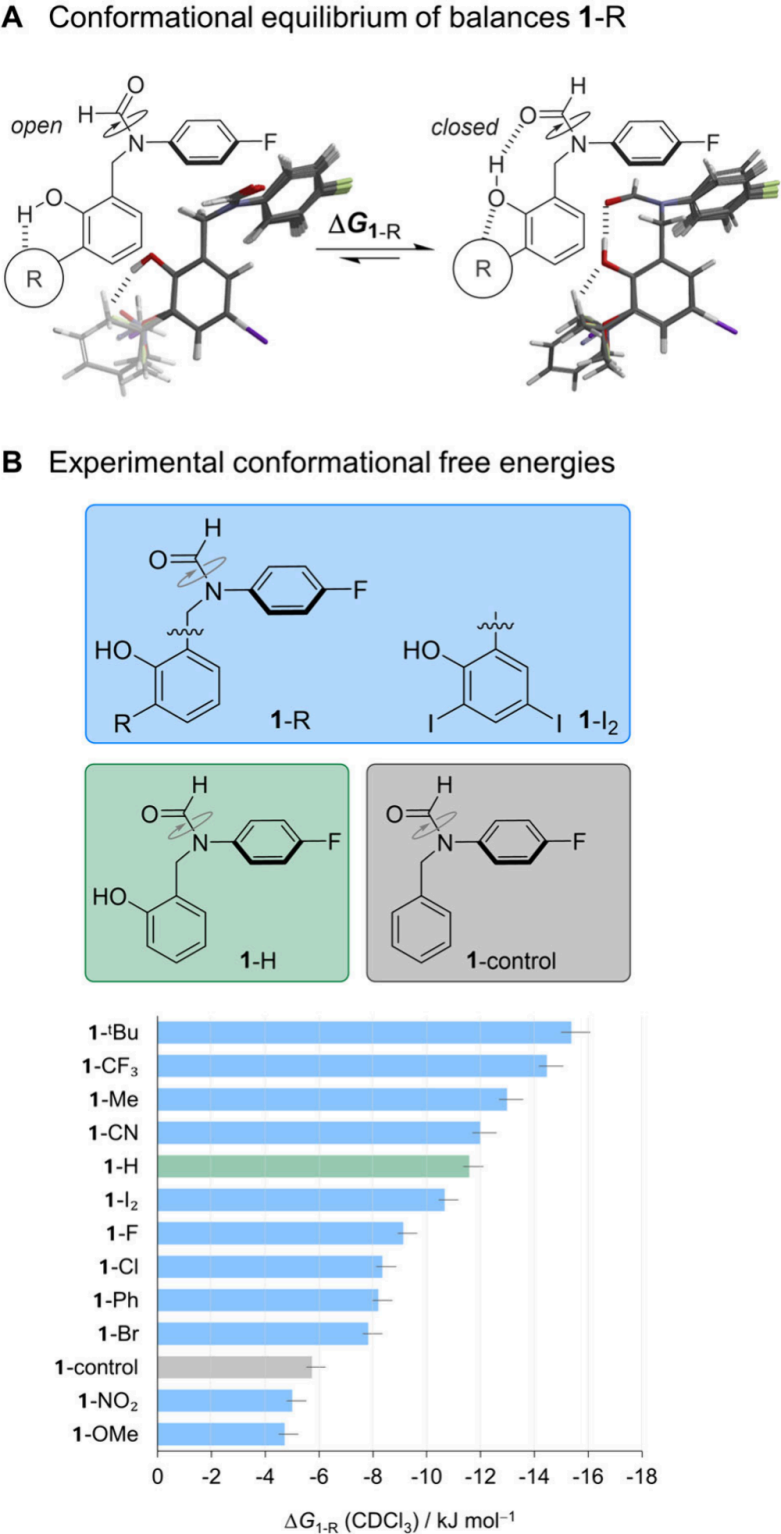
A) Molecular balances used to measure the competition between OH···R and OH···O=C interactions. Superimposed geometries calculated using M06-2X/aug-cc-pVDZ (aug-cc-pVDZ-PP for **1**-I_2_). Coordinates are provided in the Supporting Information. B) Experimentally determined conformational free energy differences between the *open* and *closed* conformers determined at 300 K.

**Figure 2:**
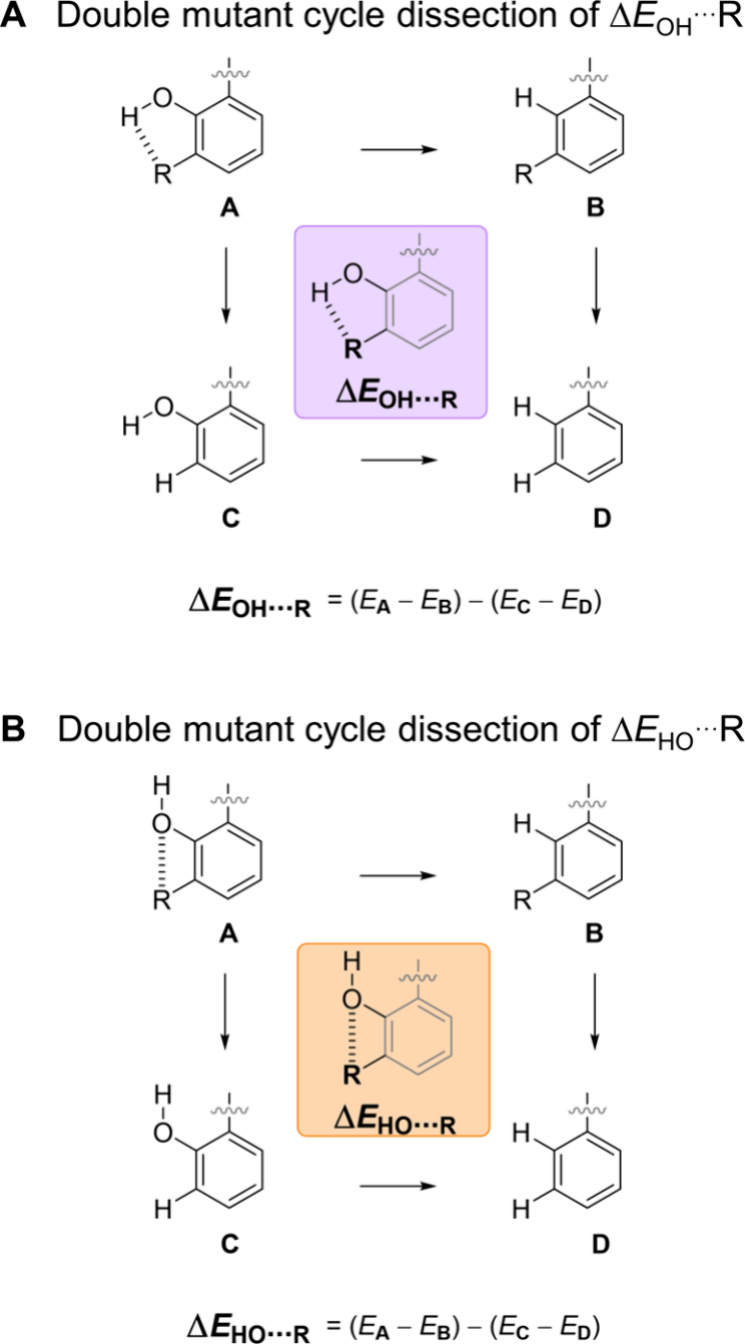
A) Competing OH···R and B) HO···R interactions determine the orientation of the OH group. Interaction energies were dissected using the thermodynamic double mutant cycles shown using the full balance structures and M06-2X calculations (Section S4.2, Supporting Information).

**Figure 3:**
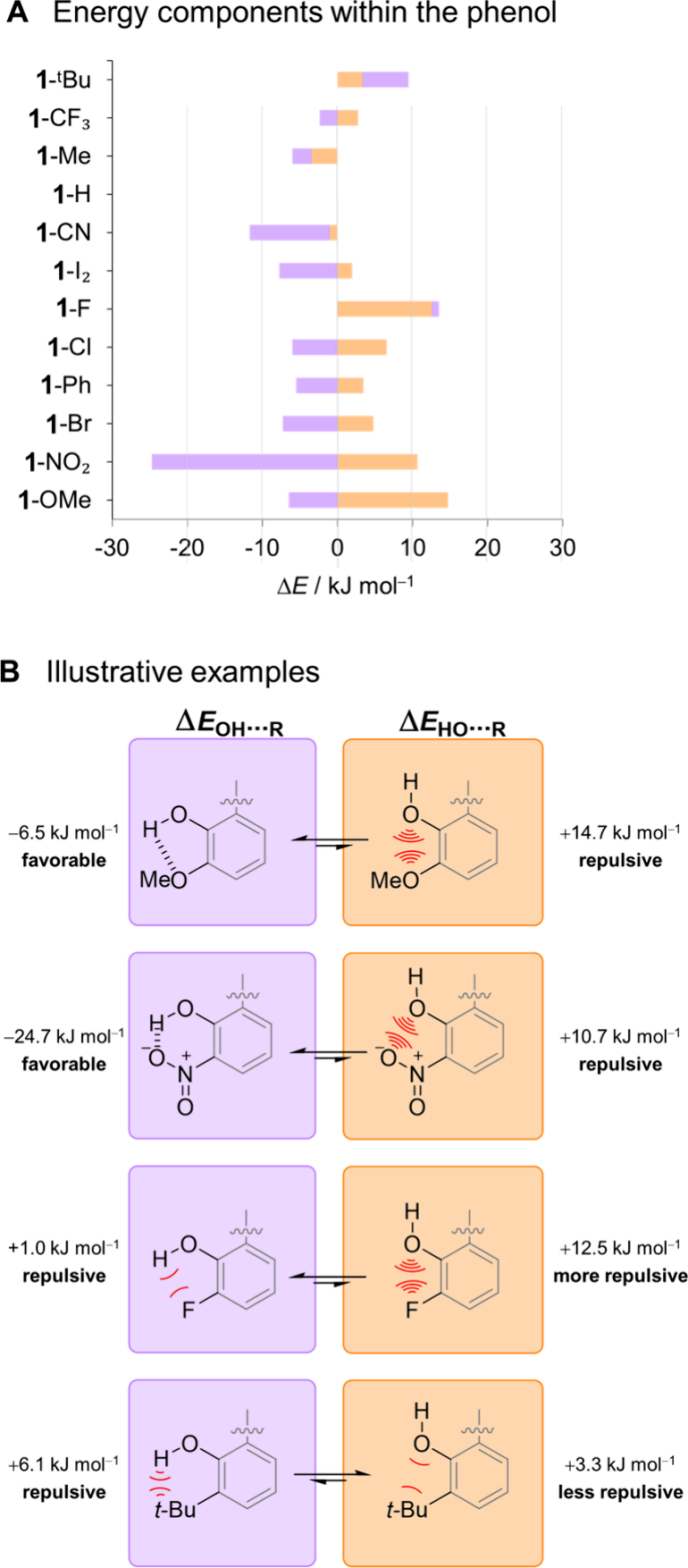
A) Competing OH···R (purple) and HO···R interactions (orange) dissected using the thermodynamic double mutant cycles shown in Figure 2. The minimization of repulsion often dominates over attractive components. B) Illustrative examples showing how these competitive interactions determine the conformation, and hence the ability of the OH group to engage in external interactions.

**Figure 4:**
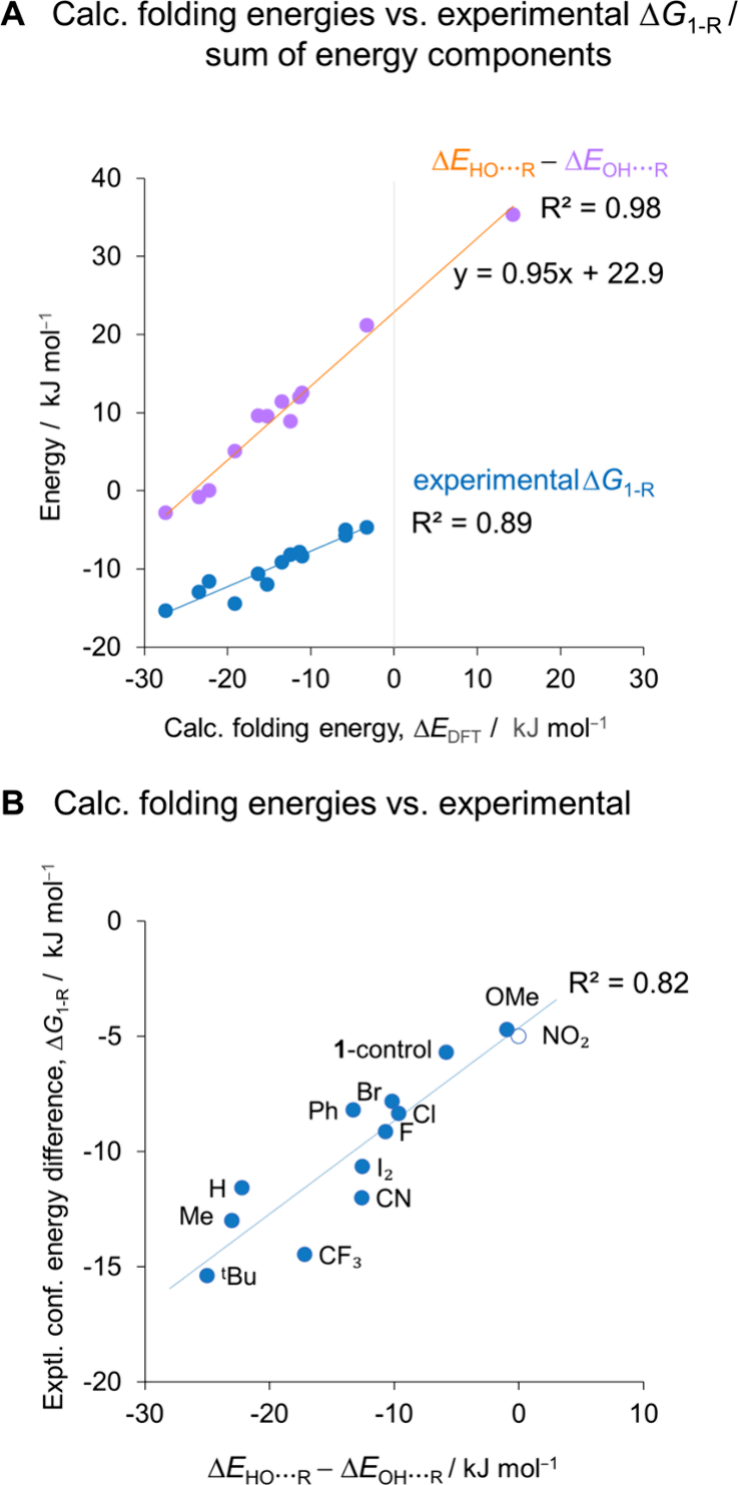
A) Experimental folding free energies (blue, Δ*G*_1-R_ from Figure 1B) and dissected intramolecular phenol interactions (purple/orange, Δ*E*_OH_···_R_ – Δ*E*_HO_···_R_) both correlate with the *open*/*closed* folding energies calculated for the full balance structures (Δ*E*_DFT_, Section S4.1, Supporting Information). All calculated energies determined in the gas phase using M06-2X. Δ*E*_OH_···_R_ – Δ*E*_HO_···_R_ = 0 for **1**-NO_2_ (hollow circle) since the primary OH···O=C H-bond cannot compete with the 35 kJ mol^–1^ preference for the OH···O_2_N conformer, and cannot manifest in the experimental Δ*G*_1-R_.

We approached the challenge of deconvoluting substituent effects on intramolecular H-bonding by synthesizing the **1**-R series of molecular balances (Figure 1A).^16,21−25^ The **1**-R series exchange between *open* and *closed* conformations via slow rotation of the formyl group, which either establishes or disrupts an H-bond with a neighboring phenol OH. This design enables the effect of an *ortho* R-substituent on the H-bond donor ability of the OH group to be measured. DFT calculations confirmed that the balances can host intramolecular OH···O=C H-bonds (Figure 1A, right), except for **1**-control, in which the R and OH groups were replaced by protons (Figure 1B, Section S4.1, Supporting Information). The minimized geometries of each conformer were remarkably consistent as the R group was varied (Figure 1A and Table S5, Supporting Information). Slow rotation of the formyl group about the C–N bond gives rise to well-separated peaks in ^19^F NMR spectra that correspond to the *open* and *closed* conformers. Integration of these peaks provides the *open*/*closed* ratio, *K*, from which the free energy difference between the two conformers was determined using Δ*G*_1-R_ = −*RT* ln *K* (Figure 1B).^23,24^

The rotation of the OH group was rapid on the NMR time scale and could not be resolved. However, comparing the energies of **1**-H and **1**-control revealed that introducing the OH group increases the *closed*:*open* ratio from 10:1 → 107:1, corresponding to an OH···O=C H-bond energy of 5.9 kJ mol^–1^ in CDCl_3_. The occurrence of this primary OH···O=C H-bond in each of the *closed* conformers was confirmed by the large additional downfield chemical shifts (Δδ = +3.2 to +4.3 ppm) of the phenolic OH protons compared to the literature values of the corresponding phenols (Table S1, Supporting Information).^26^ Intramolecular symmetry adapted perturbation theory (I-SAPT) calculations^27^ indicated that the gas-phase energies of the primary OH···O=C H-bond varied little as the R substituent was changed (−20 ± 3 kJ mol^–1^, Section S4.4, Supporting Information). Meanwhile, varying the R substituent shifted the experimental *open*/*closed* equilibrium by ∼10 kJ mol^–1^ despite the typical energetically damping influence of the solvent (Figure 1B).^23^

The energetic ordering in Figure 1B and Table 1 defies expectations based on conventional understanding of electronic substituent effects. The lack of Hammett correlations (Figure S3, Supporting Information) can be ascribed to the R substituents engaging in competitive interactions with the *ortho* phenolic OH (Figures 2 and 3).

**Table 1:** Experimental Δ*G*_1-R_ Values and Errors (in kJ mol^–**1**^) for the **1**-R Series in CDCl_3_ at 300 K

Balance	*K*	Δ*G*_1-R_	Error +/–
**1**-OMe	6.6 ± 0.05	–4.7	0.22/0.52
**1**-NO_2_	7.5 ± 0.07	–5.0	0.23/0.53
**1**-control	10.0 ± 0.15	–5.7	0.24/0.54
**1**-Br	23.1 ± 0.55	–7.8	0.26/0.56
**1**-Ph	26.8 ± 0.66	–8.2	0.59/0.84
**1**-Cl	28.5 ± 0.71	–8.4	0.61/0.85
**1**-F	39.0 ± 1.55	–9.1	0.71/0.92
**1**-I_2_	72.0 ± 16	–10.7	0.83/1.00
**1**-H	104.1 ± 25	–11.5	0.87/1.03
**1**-Me	123.2 ± 30	–12.0	0.89/1.04
**1**-CN	183.0 ± 45	–13.0	0.92/1.06
**1**-CF_3_	331.7 ± 85	–14.5	0.94/1.07
**1**-*^t^*Bu	477.8 ± 125	–15.4	0.95/1.08

The OH group may point toward or away from the R substituent, which influences the ability of the OH···O=C interaction to form, and hence the global energy difference between the NMR-resolved *open*/*closed* conformers. Since rotation of the OH group is too rapid to be resolved by NMR spectroscopy, we turned to computational chemistry to dissect the energetic contributions to the conformational preferences. First, we confirmed that the calculated gas-phase conformational energy differences Δ*E*_DFT_ correlated with the experimental Δ*G*_1-R_ values (R^2^ = 0.89, Figure 4A, blue, and Section S4.1, Supporting Information). Next, we used thermodynamic double mutant cycles (DMCs) to isolate the HO···R and OH···R interactions within the phenol fragment (Figure 2, Section S4.2, Supporting Information).^28,29^ In brief, the gas phase energy of each molecular balance was minimized in conformations in which the OH proton pointed either toward or away from the R group. The geometries of these balances were then frozen, and the energies of three “mutants” calculated in which the R group, OH group, or both were replaced with a proton. The energy differences between the two parallel mutations were then subtracted from each other to provide the dissected HO···R and OH···R interaction energies (*E*_HO···R_, pink and *E*_HO···R_, orange in Figures 2 and 3).

Figure 1B indicates that R = NO_2_ and OMe both compete strongly with the primary OH···O=C interaction. Figure 3A and 3B shows this is partially attributable to the most intuitive explanation; favorable OH···R H-bonds (purple). However, HO···R repulsion (orange) makes a dominant contribution when R = OMe. Flipping the phenolic OH proton to point toward the formyl O=C incurs a large repulsive O···O penalty, which cannot be compensated by forming the OH···O=C H-bond. The lack of strong H-bonding to either the OMe or formyl O=C also accounts for the small chemical shift of the OH proton in **1**-OMe (5.0 ppm vs 8.5–10.9 ppm for other balances, Table S1, Supporting Information). Indeed, H-bonds in 5-membered rings are remarkably weak compared to 6-membered rings (as in **1**-NO_2_).^3^

Figure 3A shows that a similar balance of attractive and repulsive interactions occurs with other R groups that “compete” with the primary OH···O=C H-bond despite not being good H-bond acceptors (i.e., R = F, Cl, Br, I, Ph).^30−33^ The key realization is that the R substituent may “compete” with the primary OH···O=C H-bond without necessarily forming a strong, or even a favorable(!) OH···R interaction. For example, the OH···F interaction is weakly repulsive, but the HO···F interaction (when the OH proton points away from the F) is *even more repulsive* due to O···F repulsion. Hence, the purple phenol conformation in Figure 3B is preferred as the *least destabilized* conformer, and not because of a stabilizing OH···F H-bond. This is consistent with the view that “organic fluorine hardly ever accepts H-bonds”.^34,35^Figure 3A similarly confirms that the CF_3_ group is a very weak H-bond acceptor.^35^

Like the F substituent, Figure 3 shows that the interactions between the bulky ^t^Bu group and the OH are destabilizing irrespective of the orientation of the OH group. However, the OH proton is driven to point away from the ^t^Bu because the purple conformer is more destabilized than the orange conformer in Figure 3B (bottom). Therefore, the ^t^Bu substituent promotes H-bonding to the formyl C=O and drives the equilibrium toward the *closed* conformer. We note that *ortho*^t^Bu groups have been used in prior studies to direct the orientation of H-bonds.^14,17,18,36^

The difference between the two double mutant cycle dissected energies (Δ*E*_OH_···_R_ – Δ*E*_HO_···_R_) governs the orientation of the phenol OH group. This term also correlates with the calculated and experimental *open*/*closed* conformational energy differences of the full balances (R^2^ = 0.98 and 0.82, respectively, Figure 4A-4B). The gradient of the like-for-like comparison of the calculated energies (purple/orange in Figure 4A) is close to unity (0.95), which supports the hypothesis that conformational preference of the phenol OH governs the energetics of the overall *open*/*closed* conformational equilibrium. Similarly, the y-intercept of 22.9 kJ mol^–1^ approximates the gas-phase energies not accounted for in Δ*E*_OH_···_R_ – Δ*E*_HO_···_R_ (i.e., the primary OH···O=C H-bond and the intrinsic steric bias toward the *closed* conformer). Notably, the Δ*E*_OH_···_R_ – Δ*E*_HO_···_R_ energies do not consider solvent effects or the OH···O=C interaction energy. Hence, the correlations in Figure 4 indicate that the propensity for the “external” OH···O=C H-bond to form was dominated by the intramolecular interactions within the phenol fragment. This explains the inability of Hammett analysis to account for the conformational trends. Nonetheless, substituent effects on the H-bond donor ability of the OH group are evident in the similarly distributed scatter of the blue points in Figure 4A and 4B. Electron-withdrawing substituents fall below the best fit lines, since the strengthening of the OH···O=C H-bond is not encoded in Δ*E*_OH_···_R_ – Δ*E*_HO_···_R_. This makes the R = CF_3_ and CN balances the largest outliers in both graphs. The R = NO_2_ substituent effect is not manifested in Δ*G*_1-R_ since the OH···O=C H-bond cannot compete with the 35 kJ mol^–1^ preference for the OH···NO_2_ conformer.

As we have seen, the ability of a phenol OH to form H-bonds with an external acceptor is dominated by conformational preferences imposed by interactions occurring within the phenol fragment itself (i.e., Δ*E*_OH_···_R_ – Δ*E*_HO_···_R_). Moreover, major aspects of Δ*E*_OH_···_R_ – Δ*E*_HO_···_R_ arise from repulsion between the OH and R groups.

In conclusion, we synthesized a series of molecular balances to explore the competition of two intramolecular interactions (R···HO vs C=O···HO). NMR spectroscopy determined the conformational preferences of series **1**-R and revealed initially puzzling substituent effects as the R-group was varied that could not be described using classic Hammett analysis. The intrinsic strength of the C=O···H H-bond was found to have a minor influence on the *open*/*closed* conformer ratio. The *open*/*closed* conformational equilibrium was instead dominated by the relative energies of the OH···R interactions in which the OH group either pointed toward or away from the *ortho*-R group. Computational dissection of the energetic contributions revealed that both HO···R repulsion and OH···R attraction determined the apparent strength of the adjacent competing OH···O=C H-bond (i.e., the position of the open/closed conformer ratio). In some cases, the avoidance of otherwise repulsive secondary interactions dominated the observed behavior (e.g., R = OMe, F, *^t^*Bu). The results underscore the necessity of considering repulsion alongside attraction in considering overall behavior. Indeed, chemists have been trained with an intrinsic bias toward recognizing favorable interactions such as H-bonds, which results in repulsive contributions that can clearly dominate the experimental behavior being overlooked. Our data show that even the behavior of simple *ortho*-substituted phenols can strongly diverge from the intuitive (but often erroneous) assumption that the spatial proximity of two fragments implies a favorable or attractive interaction. Accordingly, we caution that analyses of structural data based solely on the consideration of attractive interactions may miss crucial repulsive factors, potentially skewing interpretations of molecular conformation and crystal structure. This insight is especially relevant for compound design, including pharmaceuticals, that may be required to bind to targets or crystallize with a particular conformation. Fortunately, we show that the easily performed double mutant cycle analysis can reveal the often-repulsive origins of the conformational preferences of molecules.

## Supporting Information Available

Synthesis and characterization details, experimental methods, computational methods, tables of data (PDF)
