# Supplementary material for: Unveiling Repulsion in Intramolecular H-Bonded Systems
Source: J Am Chem Soc. 2025 Apr 7;147(15):12381–5. doi: 10.1021/jacs.5c00385 (PMC12057996; doi:10.1021/jacs.5c00385)
Supplement: Supplementary file 1 — ja5c00385_si_001.pdf [file ja5c00385_si_001.pdf]

Supplementary Information

Unveiling Repulsion in Intramolecular H-bonded Systems

Ivan V. Smolyar, Scott L. Cockroft*

EaStCHEM School of Chemistry, University of Edinburgh, Joseph Black Building, David Brewster Rd, Edinburgh, EH9 3FJ, United Kingdom

Table of Contents

S1: General Information 2

S2: Experimental Conformational Free Energy Measurements 3

S3: Methanol dilution experiments 9

S4: Computational Details 11

S4.1: Calculated conformer energies and geometries of compound series **1**-R 11

S4.2: DMC analysis of OH···R and HO···R interactions in compounds **1**-R. 35

S4.2.1: DMC analysis of HO···R interactions in closed conformers of compounds **1**-R. 37

S4.2.2: DMC analysis of OH···R interactions in closed conformers of compounds **1**-R. 56

S4.3: Summary of Computational Results and Supplementary Values for main text Figure 3 & 4 . 75

S4.4: SAPT calculations of the OH···O=CH hydrogen bond energy in balances **1**-R 75

S5: Compound synthesis and characterization 78

General procedure A: Synthesis of imines **2**-R 79

General procedure B: Synthesis of amines **3**-R 108

General procedure C: Synthesis of formamide molecular balances **1**-R. 134

References 212

S1: General Information

Unless stated otherwise, all chemicals were purchased from commercial sources (Sigma Aldrich UK, Acros UK, VWR UK or Fluorochem UK) and used without further purification. Dry solvents were obtained by means of a "Glass Contour" brand solvent purification system, where solvents were passed through filter columns and dispensed under an argon atmosphere. Flash column chromatography was performed using Geduran® Si60 (40-63 mm, Merck, Germany) as the stationary phase, and thin-layer chromatography (TLC) was performed on pre-coated silica gel plates (0.25 mm thick, 60F254, Merck, Germany) and observed under UV light (lmax 254 nm). Mass spectrometry was performed by the University of Edinburgh technician-supported mass spectrometry service, using a Bruker micrOTOF II or ThermoElectron MAT XP spectrometer for EI or ESI-HRMS. Melting points were measured in a Gallenkamp melting point apparatus. ^{1}H and ^{13}C NMR spectra were recorded on Bruker Ultrashield 400 MHz, Bruker Ascend 500 MHz equipped with a DCH cryoprobe and Bruker Ascend 500 MHz with prodigy cryoprobe and Bruker Ultrashield 600 MHz with TCI cryoprobe, at a constant temperature of 25 °C, unless otherwise stated. ^{1}H, ^{13}C and ^{19}F chemical shifts are reported in parts per million (ppm) from low to high field. ^{1}H and ^{13}C values are referenced to the literature values for chemical shifts of residual non-deuterated solvent, with respect to tetramethylsilane. ^{19}F is referenced externally to $CFCl_3$ at 0 ppm. Standard abbreviations indicating multiplicity are used as follows: bs (broad singlet), d (doublet), dd (doublet of doublets), m (multiplet), q (quartet), s (singlet), t (triplet), tt (triplet of triplets), *J* (coupling constant). All spectra were analysed using MestReNova (Version 14.2.3). NMR tubes, precision glassware and glass syringes were dried under vacuum before use. Deuterated solvents were stood over oven-dried activated 4 Å molecular sieves for a minimum of 24 h prior to use. Non-deuterated anhydrous solvents were used directly as commercially obtained anhydrous solvents or redistilled under reduced pressure from analytical-grade solvents.

S2: Experimental Conformational Free Energy Measurements

Since rotation around the formamide bond is slow on the NMR timescale, discrete peaks corresponding to the open (unfolded) and closed (folded) conformers are observed. Thus, integration of the conformer peaks provides direct access to the conformational equilibrium constant, *K*, which can be used to determine the conformational free energy difference. All molecular torsion balances were fully characterized in DMSO-d_6 by ^{1}H and ^{13}C-NMR, prior to the determination of experimental conformational free energies in $CDCl_3$. The NMR resonances corresponding to the folded and unfolded conformers were assigned using 2D NMR methods. The assignment of conformer peaks was greatly simplified due to the steric differences on each side of the balance; all balances were found to prefer the closed conformation. The major and minor conformers were unambiguously distinguished by analysing the HMBC spectra of the molecular balances H-C three bond correlations. An example of such spectrum is shown in Figure S1. The formyl proton has a cross peak with the *trans*-CH_2 aliphatic carbon in the closed conformer (cross-peak at {8.57, 42.49}), whereas in the unbound conformer the formyl proton has a *trans*-C quaternary aromatic carbon (cross-peak at {8.51, 135.17}). The conformation (open or closed) of major and minor species were assigned by the relative integrals of the formamide ^{1}H signals.

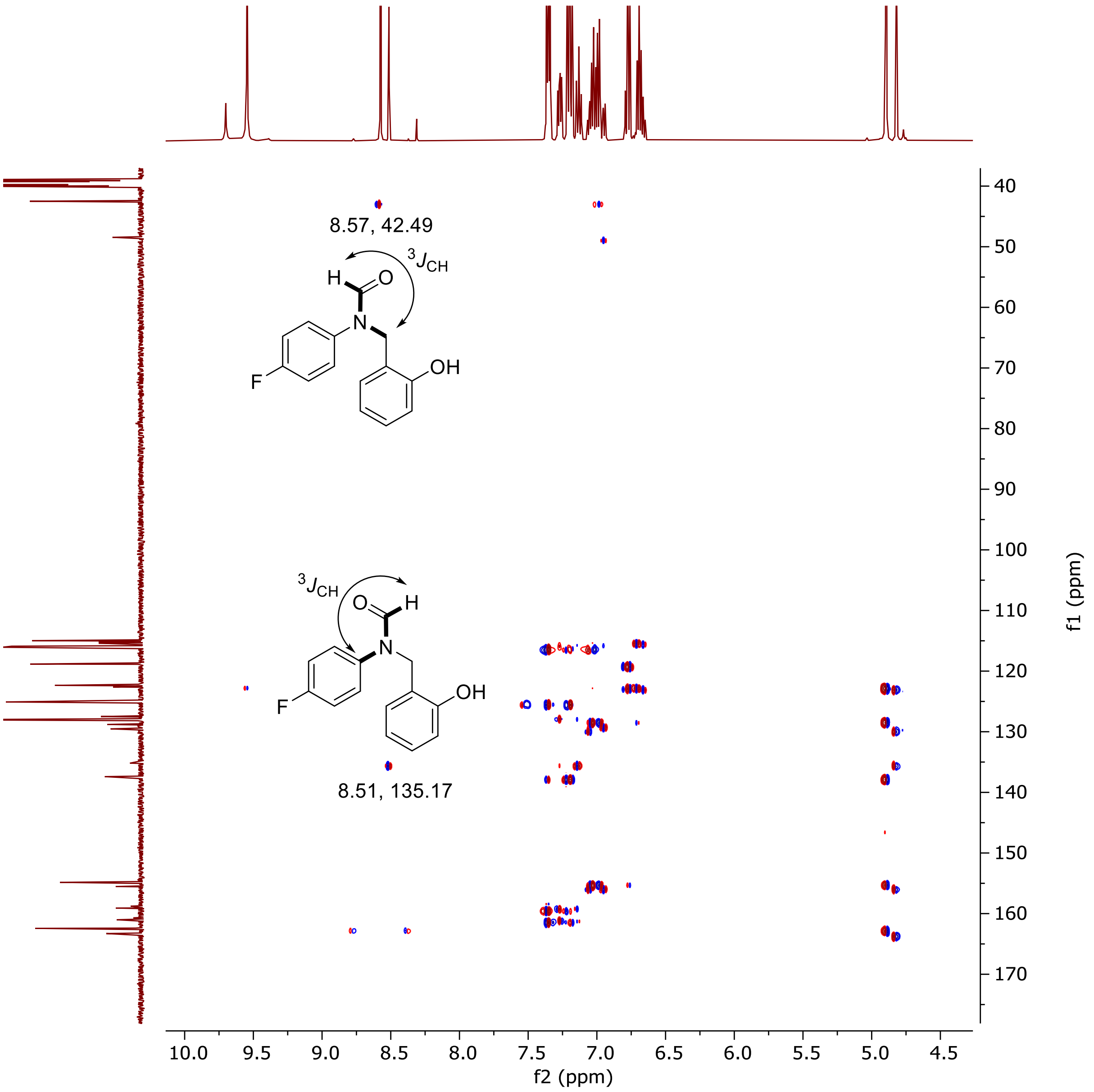

Figure S1: Example HMBC spectrum of compound **1**-H in DMSO-d_6; Key cross-peaks corresponding to two distinct conformations of the formamide fragments are annotated.

To confirm that the primary OH···O=C H-bond forms in the closed conformer, we compared the chemical shifts of the phenolic OH proton in the closed conformer to the literature values of the corresponding *ortho*-substituted phenols. The chemical shift observed in the NMR spectra provided an indication of the phenolic proton environment, with noticeable downfield shifts corresponding to the presence of hydrogen bonding.[1] Compared to the reported values, a large downfield shift (3.2 - 4.2 ppm) was observed for the phenolic OH ^{1}H NMR signal in the closed conformers of balances **1**-R (Table S1). This observation is consistent with hydrogen bonds forming in the closed conformer of these compounds. Two exceptions are compounds

1-OMe and **1**-NO_2, which display no evidence of H-bond formation in the closed conformer despite it being more favoured than the open conformer.

Table S1: Comparison of the observed chemical shifts ($CDCl_3$) of OH hydrogen in closed conformers of balances **1**-R and reported literature values of same chemical shifts in ortho-substituted phenols phenol-R

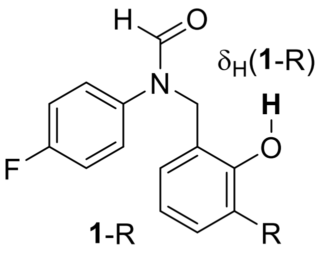

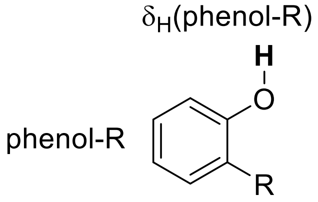

R	δ_H(**1**-R)	δ_H (phenol-R)
^{t}Bu	8.89	4.82[2]
CF_3	9.75	5.47[3]
Me	8.88	4.80[4]
H	8.96	5.35[4]
I_2	9.55	5.32[5]
F	8.68	5.88[4]; 5.09[6]
Ph	8.51	5.23
Br	8.75	5.48[6]
Cl	8.75	5.51[6]
NO_2	10.87	10.57[4]
OMe	4.97	5.60[6]; 5.83[7]

NMR spectra used for conformational free energy determination were determined by ^{19}F NMR spectroscopy using a Bruker Ultrashield 500 MHz, heteronuclear (512 scans). Conformer ratios were determined at balance concentrations of 5 mM and were found to be independent of concentration in a range of 10–100 mM up to the detection limit. In the worst-case scenario, the value of ΔG would change by 0.5 kJ/mol, which was used as a conservative error to account for intermolecular self-aggregation of the molecular balances in the $CDCl_3$ solution. Errors in estimation of equilibrium constants $\Delta K_{1\text{-R}}$ from the integrals of the minor and major peaks were then determined as follows:

$$\Delta K = \frac{1}{SNR_{minor}} + \frac{K}{SNR_{minor}}$$

Where SNR is Signal-to-Noise Ratio parameter of the minor conformers ^{19}F signal evaluated as implemented in MestReNova (Version 14.2.3). A representative example of compounds which have of ^{19}F NMR spectra for compounds with different

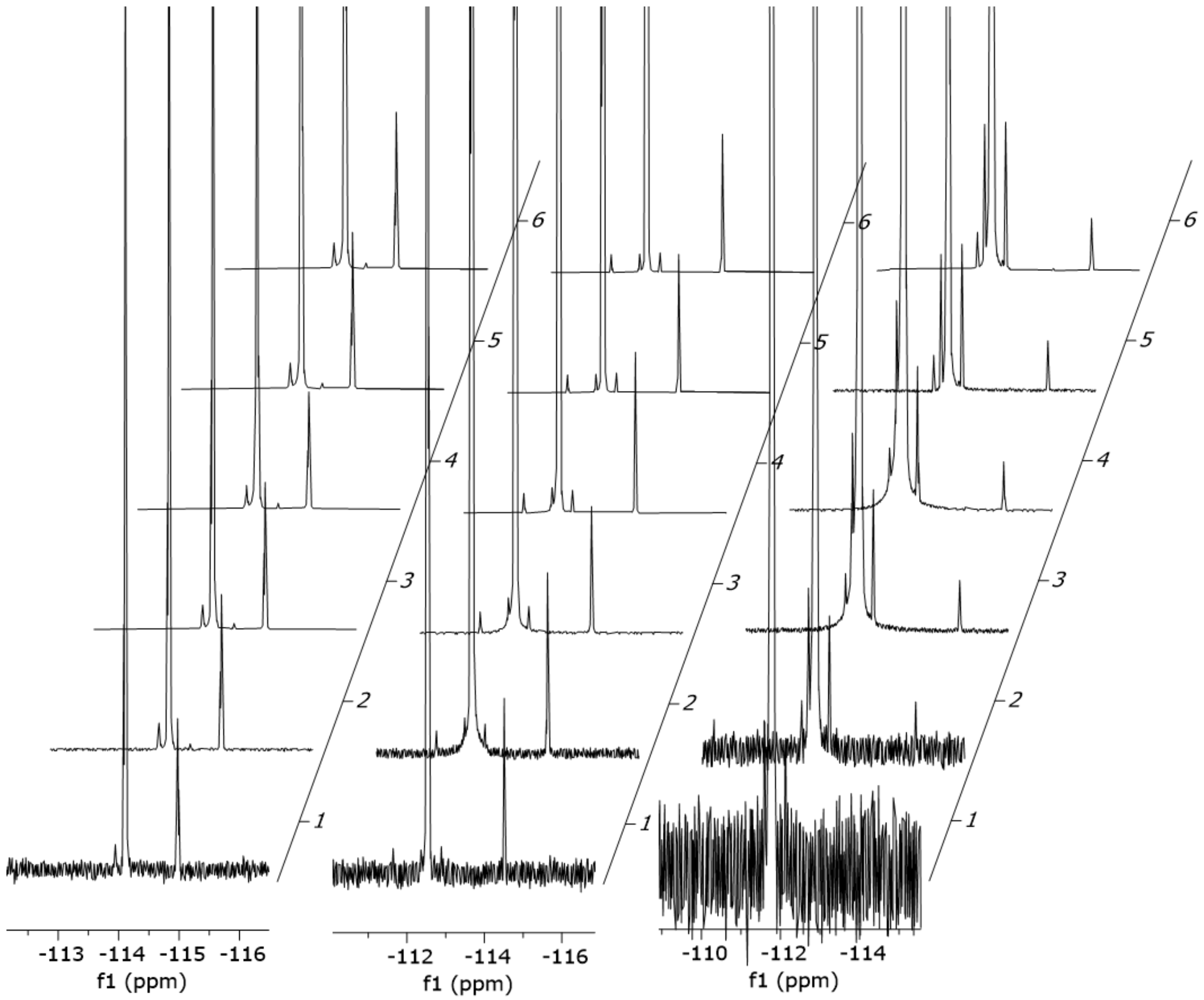

Figure S2: Selected examples of dilution experiments. Compounds **1**-OMe, **1**-Br and **1**-CF_3 belong to different extrema of the experimental dataset.

Table S2: Experimental determination of dependence of K on the concentration of the molecular balances **1**-OMe, **1**-Br and **1**-CF_3

Run	C, mM	*K*	SNR* of minor conformer	Δ*G*, kJ/mol	Positive error, kJ/mol	Negative error, kJ/mol
**1-OMe**						
1	1.0	7.12 ± 0.41	20	-4.89	0.14	0.15
2	5.0	6.73 ± 0.05	141	-4.75	0.02	0.02
3	10.0	6.62 ± 0.01	359	-4.71	0.01	0.01
4	25.0	6.56 ± 0.01	629	-4.68	0.01	0.01
5	50.0	6.48 ± 0.01	666	-4.66	0.01	0.01
6	100.0	6.47 ± 0.01	977	-4.65	0.01	0.01
**1-Br**						
1	1.0	23.62 ± 1.42	17	-7.88	0.16	0.15
2	5.0	23.17 ± 0.55	44	-7.83	0.06	0.06
3	10.0	23.10 ± 0.20	123	-7.83	0.02	0.02
4	25.0	22.78 ± 0.06	424	-7.79	0.01	0.01
5	50.0	22.51 ± 0.05	1000	-7.76	0.01	0.01
6	100.0	22.47 ± 0.02	1037	-7.76	0.01	0.01
**1-CF_3**						
1	1.0	***n/d***	***≈ 1***	***n/d****	***n/d***	***n/d***
2	5.0	340.66 ± 85.20	4	-14.54	0.72	0.55
3	10.0	335.48 ± 13.25	25	-14.50	0.10	0.10
4	25.0	325.07 ± 6.65	49	-14.41	0.05	0.05
5	50.0	321.21 ± 2.85	113	-14.39	0.02	0.02
6	100.0	318.38 ± 1.86	175	-14.36	0.01	0.01

*signal-to-noise ratio; **not determined

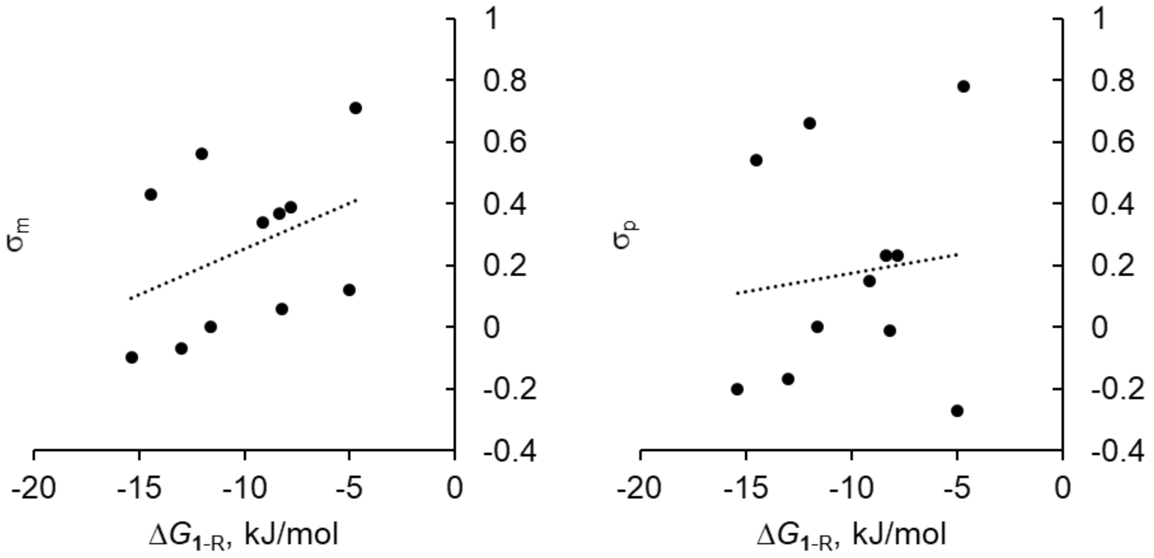

Figure S3: Absence of Hammett correlation between the observed conformational energies and R-group substituent constants σ_m (left, $R^2 = 0.16$) and σ_p (right, $R^2 = 0.01$).

S3: Methanol dilution experiments

To confirm that we were not assigning a random impurity as the minor conformer in NMR spectra, especially in the case of compounds with a large value of *K*, dilutions with CD_3OD were conducted. An illustrative example is shown in Figure S4, where the signal of the minor conformer is gradually increasing as the intramolecular H-bond becomes disrupted by the addition of CD_3OD. Hence, to confirm the identity of the peak corresponding to the minor conformer, 10 mM solutions of the molecular balances in were prepared in mixtures of $CDCl_3$ and CD_3OD (up to 25% v/v).

Table S3: Δ*G* (kJ/mol) of molecular balances **1-R** in $CDCl_3$ upon dilution with CD_3OD.

**1-R**	**Run**					
	**1**	**2**	**3**	**4**	**5**	**6**
	**C(CD_3OD), % (v/v)**					
	**0**	**1**	**5**	**10**	**15**	**25**
**1**-tBu	-15.4	-15.0	-13.6	-12.5	-11.6	-11.1
**1**-CF3	-14.5	-13.8	-11.5	-10.1	-9.4	-8.4
**1**-CN	-12.0	-11.4	-9.4	-7.9	-7.3	-6.5
**1**-NO2	-5.0	-4.9	-4.4	-4.3	-4.2	-4.2
**1**-Br	-7.8	-7.5	-6.2	-5.6	-5.2	-4.6
**1**-H	-11.6	-9.6	-5.9	-4.5	-4.0	-3.2
**1**-Me	-13.0	-12.1	-8.5	-7.2	-6.5	-6.0
**1**-Ph	-8.2	-7.9	-7.3	-6.6	-6.2	-6.0
**1**-OMe	-4.7	-4.5	-4.0	-3.5	-3.4	n/d*
**1**-control	-5.7	-5.7	-5.6	-5.4	-5.3	-5.1
**1**-I2	-10.7	-10.2	-8.8	-7.8	-7.3	-6.6
**1**-Cl	-8.3	-7.9	-6.3	-5.7	-5.2	-4.6
**1**-F	-9.1	-7.6	-5.2	-4.4	-4.0	-3.4

*not determined due to the signal overlap of minor and major conformers

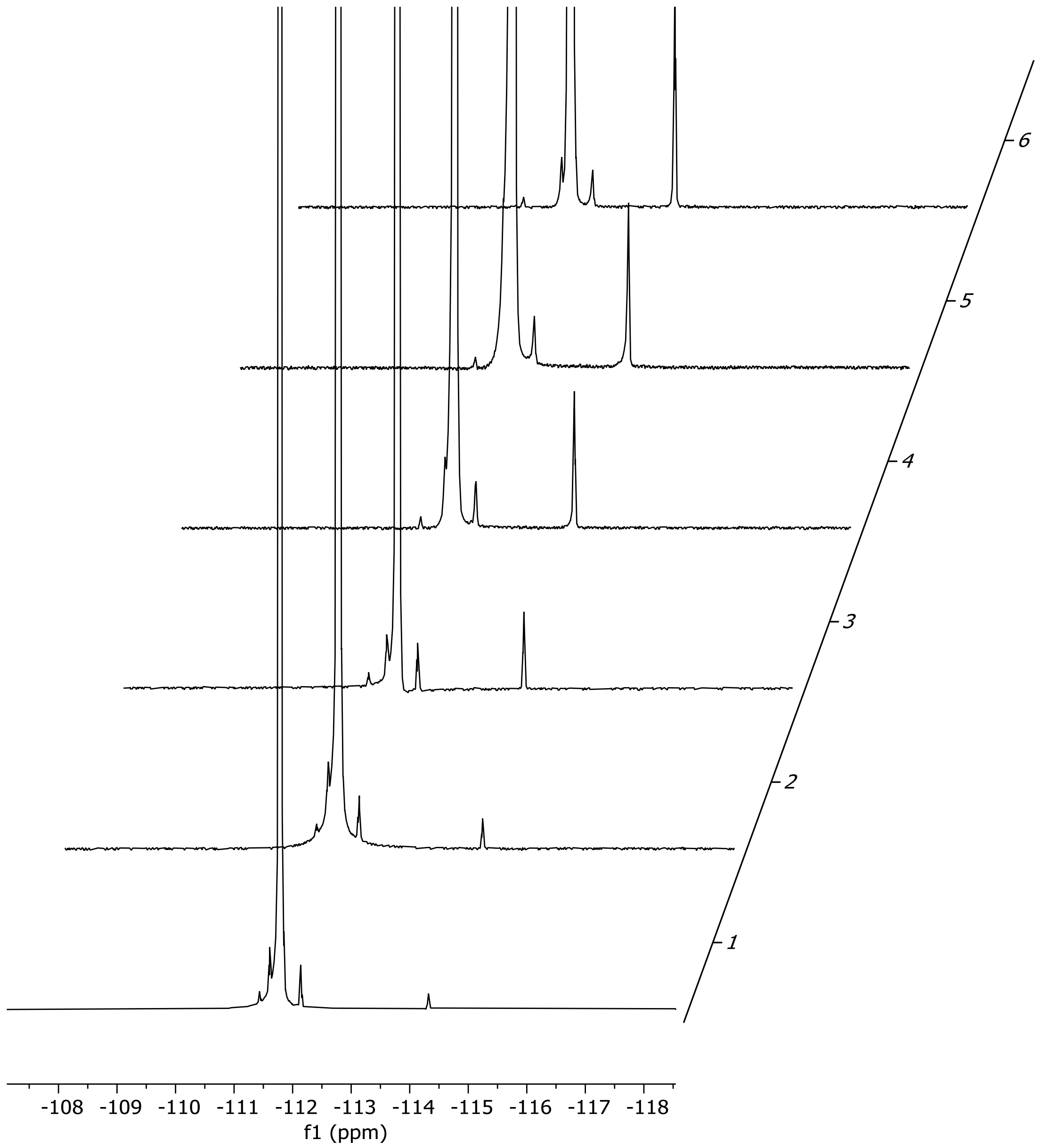

Figure S4: Example of dilution of compound **1**-CF_3 with CD_3OD.

S4: Computational Details

S4.1: Calculated conformer energies and geometries of compound series **1**-R

Geometry optimisations were run using M06-2X/aug-cc-pVDZ level of theory (aug-cc-pVDZ-PP for iodine atoms) using Psi4[8] Python API. Total (SCF) energies of all species in the table are given in kJ/mol. ΔE_{DFT} corresponds to the energy of the lowest energy closed conformer minus the energy of the open conformer (Table S4). In all cases the closed-bonded conformation was found to be the lowest energy closed conformer, with the exception of **1-**NO_2, which strongly preferred the closed-nonbonded conformer due to the strong $OH{\cdots}O_2N$ hydrogen bond. ΔE_{DFT} was found to correlate with the experimental $\Delta G_{\mathrm{1\text{-}R}}$ values (blue line, main text Figure 4A).

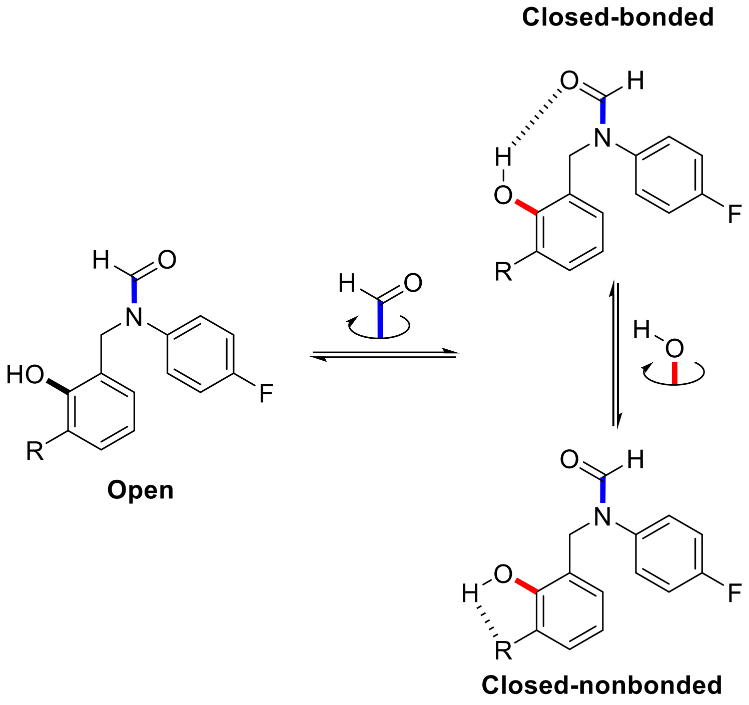

Table S4: Total SCF energies (kJ/mol) of individual conformers of compounds **1**-R calculated using M06-2X/aug-cc-pVDZ (aug-cc-pVDZ-PP for iodine atoms).

Balance	Open	Closed-nonbonded	Closed-bonded	ΔE_{DFT}
**1**-H	-2220023.54	-2220027.34	**-2220045.74**	-22.2
**1**-CF_3	-3104811.22	-3104816.60	**-3104830.32**	-19.1
**1**-Br	-8976973.22	-8976977.86	**-8976984.55**	-11.33
**1**-Cl	-3426693.76	-3426698.07	**-3426704.77**	-11.01
**1**-F	-2480535.99	-2480540.39	**-2480549.46**	-13.47
**1**-Me	-2323210.16	-2323212.87	**-2323233.59**	-23.43
**1**-NO_2	-2756878.55	-2756884.35	-2756864.22	-5.80<br>+14.33[a]
**1**-OMe	-2520640.23	-2520643.14	**-2520643.45**	-3.22
**1**-Ph	-2826508.49	-2826513.75	**-2826520.91**	-12.42
**1**-^{t}Bu	-2632730.38	-2632735.85	**-2632757.82**	-27.44
**1**-CN	-2462182.45	-2462188.05	**-2462197.64**	-15.19
**1**-control	-2022538.79	**-2022544.59**	**-2022544.59**	-5.80
**1**-I_2	-3769174.94	-3769182.10	**-3769191.80**	-16.86

[a]Value used for comparison of double-mutant cycle energy comparison in main-text Figure 4 that uses the equivalent geometry change.

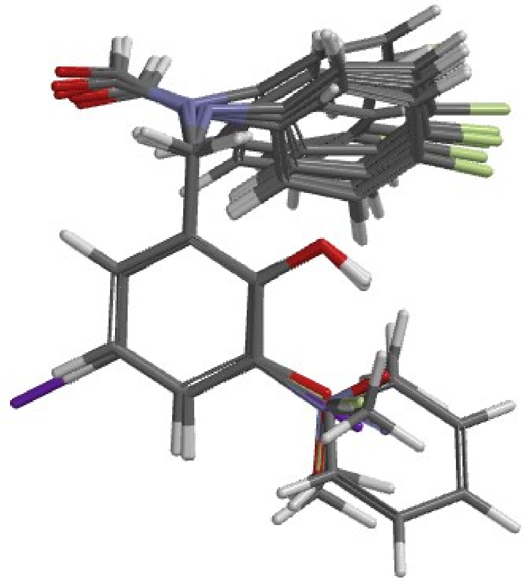

Figure S5: Superimposed ground-state geometries of the **Closed-Nonbonded** conformers of molecular balance series **1**-R. Images of the **Closed-Bonded**, and **Open** conformers are provided in main text Figure 1. Coordinates for all calculated geometries are provided on the pages that follow.

Open Conformer Geometries

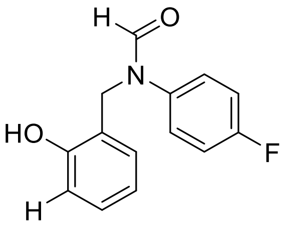

1-H (**Open**)

C	-3.010710	-0.052392	1.012179
C	-2.619785	-0.131565	-1.755716
C	-4.093005	0.102293	0.151548
C	-1.720293	-0.243307	0.496666
C	-1.537127	-0.279618	-0.891570
C	-3.880849	0.060179	-1.217581
N	-0.607535	-0.442395	1.360664
F	-4.932845	0.206035	-2.050937
C	-0.523466	0.114774	2.614002
O	-1.355257	0.813391	3.152110
C	0.576806	-1.122315	0.853964
C	1.570306	-0.212797	0.160702
C	3.496922	1.363546	-1.130057
C	2.709842	-0.795214	-0.408848
C	1.416343	1.168700	0.071777
C	2.370989	1.960142	-0.569570
C	3.670061	-0.017910	-1.051785
O	2.816696	-2.154535	-0.290394
H	-3.167412	-0.021803	2.084651
H	-2.487808	-0.157941	-2.835191
H	-5.099602	0.248448	0.537514
H	-0.545046	-0.410922	-1.316472
H	0.430728	-0.148553	3.110191
H	1.073186	-1.621606	1.695654
H	0.258177	-1.922582	0.176173
H	4.248209	1.970502	-1.633030
H	0.530334	1.634369	0.503483
H	2.231651	3.037416	-0.630458
H	3.630027	-2.452200	-0.711711
H	4.550460	-0.492140	-1.487960

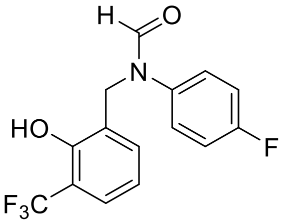

1-CF_3 (**Open**)

C	-3.646714	-0.276184	0.558508
C	-2.828004	-0.075641	-2.108612
C	-4.591219	-0.106319	-0.448494
C	-2.281837	-0.340678	0.242320
C	-1.883998	-0.239240	-1.096771
C	-4.167213	-0.007661	-1.764875
N	-1.305820	-0.546090	1.257311
C	-0.013775	-1.115030	0.905064
C	1.018687	-0.093649	0.471763
C	3.019941	1.708034	-0.312888
C	2.290602	-0.562682	0.115779
C	0.771073	1.274940	0.430079
C	1.761503	2.178016	0.039376
C	3.286951	0.338988	-0.275019
C	4.638095	-0.188266	-0.647545
F	4.570737	-1.046022	-1.694587
F	5.191810	-0.904039	0.363091
F	5.505303	0.772643	-0.974030
O	2.465405	-1.908864	0.183525
C	-1.458238	-0.092512	2.546874
O	-2.408476	0.513180	2.991607
F	-5.086381	0.156486	-2.739216
H	-3.969773	-0.353772	1.590722
H	-2.529520	0.004315	-3.151652
H	-5.653409	-0.055929	-0.218854
H	-0.831402	-0.275628	-1.367548
H	0.372345	-1.663547	1.773146
H	-0.159307	-1.864510	0.118509
H	3.806302	2.394111	-0.619851
H	-0.218511	1.642315	0.701816
H	1.548596	3.244231	0.011603
H	3.365022	-2.165103	-0.052150
H	-0.578775	-0.345891	3.171000

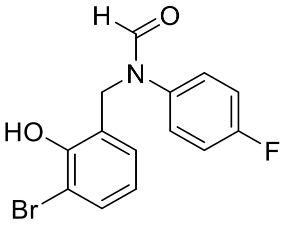

1-Br (**Open**)

C	3.153894	-0.386396	0.357263
C	2.226256	2.235265	0.050992
C	4.057162	0.653642	0.165829
C	1.776093	-0.125936	0.392259
C	1.323658	1.190753	0.237111
C	3.579771	1.946739	0.015202
F	4.460652	2.953163	-0.167381
N	0.838855	-1.171529	0.626631
C	1.054437	-2.473174	0.240111
O	2.032599	-2.904772	-0.330333
C	-0.472341	-0.845550	1.166527
C	-1.502050	-0.466978	0.120142
C	-3.499291	0.248563	-1.715830
C	-2.754740	-0.033083	0.580176
C	-1.265923	-0.536894	-1.249253
C	-2.256516	-0.183666	-2.166432
C	-3.736599	0.321346	-0.346200
O	-2.930958	0.013181	1.924187
H	3.517546	-1.400737	0.475716
H	1.885248	3.261029	-0.070025
H	5.128723	0.468229	0.138980
H	0.260348	1.416399	0.247738
H	0.198862	-3.121848	0.512221
H	-0.838202	-1.711928	1.731604
H	-0.363424	-0.034788	1.895505
H	-4.284210	0.532430	-2.413327
H	-0.289307	-0.862291	-1.605789
H	-2.059143	-0.241111	-3.234593
H	-3.820456	0.337659	2.123288
Br	-5.420944	0.922284	0.297681

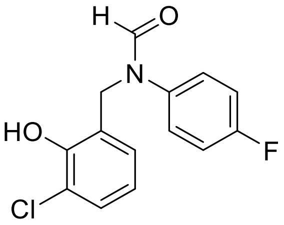

1-Cl (**Open**)

C	3.135504	-0.575505	0.255266
C	2.369856	2.107632	0.054481
C	4.097742	0.410982	0.064771
C	1.778943	-0.229998	0.342006
C	1.407900	1.117086	0.239327
C	3.700211	1.735607	-0.033320
F	4.637225	2.690306	-0.215306
N	0.783417	-1.220199	0.575118
C	0.902667	-2.521559	0.147510
O	1.836338	-3.001801	-0.457266
C	-0.490514	-0.828651	1.159622
C	-1.525658	-0.368067	0.152341
C	-3.539277	0.487849	-1.607760
C	-2.755370	0.079493	0.655173
C	-1.319295	-0.379770	-1.224131
C	-2.317048	0.044677	-2.102812
C	-3.746161	0.501535	-0.232549
O	-2.915296	0.071741	2.002628
H	3.437509	-1.613718	0.332575
H	2.092049	3.156093	-0.026632
H	5.153747	0.158803	-0.001797
H	0.362361	1.410504	0.290439
H	0.011217	-3.119068	0.421899
H	-0.892102	-1.683868	1.717094
H	-0.312851	-0.042784	1.902264
H	-4.332993	0.823075	-2.271770
H	-0.360836	-0.717425	-1.616307
H	-2.141335	0.031544	-3.176139
H	-3.787960	0.424842	2.223883
Cl	-5.269991	1.050647	0.429394

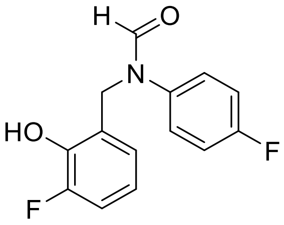

1-F (**Open**)

C	3.093214	-0.737281	0.168282
C	2.439087	1.980213	0.037762
C	4.091054	0.210944	-0.034117
C	1.756253	-0.335462	0.302576
C	1.441533	1.028454	0.234607
C	3.748616	1.553290	-0.096706
F	4.720390	2.470338	-0.289690
N	0.723821	-1.284167	0.548780
C	0.773597	-2.583354	0.101877
O	1.671234	-3.097309	-0.529220
C	-0.516562	-0.841937	1.168068
C	-1.543657	-0.299071	0.194148
C	-3.539948	0.726443	-1.510197
C	-2.714192	0.245174	0.727380
C	-1.385204	-0.324249	-1.192261
C	-2.370647	0.181966	-2.039612
C	-3.684102	0.746135	-0.135353
O	-2.874278	0.270570	2.078561
H	3.351944	-1.788643	0.218475
H	2.204817	3.040930	-0.016669
H	5.132678	-0.085477	-0.137207
H	0.412087	1.366559	0.322131
H	-0.138229	-3.143449	0.387488
H	-0.951746	-1.690580	1.710420
H	-0.283375	-0.086685	1.927052
H	-4.329638	1.132321	-2.138804
H	-0.470238	-0.736279	-1.615616
H	-2.226974	0.155734	-3.117352
H	-3.734527	0.658150	2.284805
F	-4.797009	1.266721	0.440394

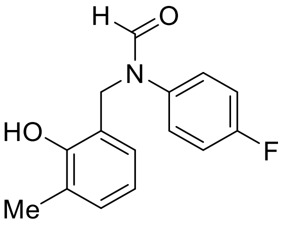

1-Me (**Open**)

C	3.576794	-0.902633	0.117972
C	2.953831	1.824271	0.090941
C	4.588335	0.041530	-0.029164
C	2.241808	-0.492216	0.246001
C	1.942731	0.876507	0.231458
C	4.260819	1.388890	-0.041636
N	1.196019	-1.439156	0.429248
F	5.244931	2.302598	-0.185589
C	1.235168	-2.709195	-0.094012
O	2.135873	-3.198777	-0.741051
C	-0.050293	-1.015323	1.053931
C	-1.061497	-0.419596	0.095150
C	-3.018502	0.696873	-1.555074
C	-2.256992	0.074483	0.628322
C	-0.863559	-0.347622	-1.282022
C	-1.837815	0.209388	-2.109178
C	-3.250977	0.639206	-0.178967
O	-2.396792	-0.029400	1.987224
C	-4.522364	1.158425	0.439561
H	3.823579	-1.958146	0.127956
H	2.732002	2.889041	0.076516
H	5.628290	-0.262218	-0.128431
H	0.914492	1.219655	0.314479
H	0.311847	-3.272079	0.144512
H	-0.497101	-1.884026	1.553464
H	0.182696	-0.299688	1.850582
H	-3.781379	1.133760	-2.199692
H	0.066613	-0.719874	-1.710371
H	-1.673881	0.264568	-3.183422
H	-3.244025	0.340005	2.257631
H	-5.185058	1.565401	-0.330300
H	-4.3226	1.9637	1.162309
H	-5.073	0.36165	0.961651

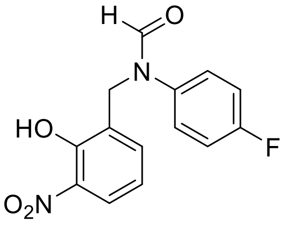

1-NO_2 (Open)

C	-3.482661	-0.226486	0.616948
C	-2.704986	-0.143906	-2.069401
C	-4.441209	-0.088045	-0.381062
C	-2.123924	-0.317313	0.281649
C	-1.746795	-0.276259	-1.066732
C	-4.037491	-0.047108	-1.707290
N	-1.133206	-0.492226	1.289486
C	0.143748	-1.094650	0.941786
C	1.182315	-0.108116	0.443706
C	3.195815	1.630609	-0.461423
C	2.431576	-0.637479	0.062266
C	0.966410	1.258806	0.364460
C	1.965731	2.133823	-0.086508
C	3.424482	0.251291	-0.387399
O	2.577456	-1.964429	0.163089
N	4.731126	-0.244433	-0.792197
O	4.942351	-1.458055	-0.713447
O	5.554925	0.543032	-1.194649
F	-4.970271	0.088582	-2.672482
C	-1.261021	0.017190	2.561187
O	-2.193887	0.659795	2.989741
H	-3.789299	-0.258226	1.656185
H	-2.423075	-0.110780	-3.119233
H	-5.498981	-0.017768	-0.137435
H	-0.699679	-0.338184	-1.352927
H	0.540035	-1.607707	1.826786
H	-0.021902	-1.876800	0.192649
H	3.998437	2.271710	-0.815174
H	-0.005939	1.658227	0.652995
H	1.772491	3.202221	-0.140357
H	3.486332	-2.179909	-0.130377
H	-0.378900	-0.227410	3.185161

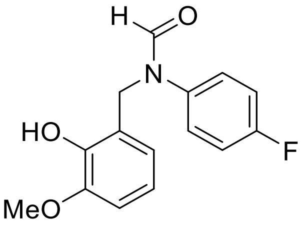

1-OMe (**Open**)

C	3.886247	-0.486793	0.349957
C	2.904235	2.127912	0.201336
C	4.766931	0.579999	0.203113
C	2.504516	-0.256733	0.417945
C	2.024405	1.056885	0.342125
C	4.262164	1.869240	0.130377
F	5.120768	2.902143	-0.009728
N	1.591744	-1.331995	0.605266
C	1.817573	-2.597547	0.120201
O	2.790505	-2.971368	-0.498897
C	0.285441	-1.064163	1.191128
C	-0.770731	-0.632151	0.194672
C	-2.831573	0.173801	-1.540904
C	-2.008319	-0.244177	0.696821
C	-0.567903	-0.615719	-1.189143
C	-1.587370	-0.217431	-2.045739
C	-3.039119	0.160256	-0.165889
O	-2.205732	-0.265659	2.045066
H	4.271148	-1.498686	0.409080
H	2.542089	3.151868	0.140352
H	5.841407	0.417631	0.150982
H	0.956661	1.258497	0.378997
H	0.977660	-3.277560	0.361732
H	-0.053586	-1.973692	1.702906
H	0.399375	-0.304007	1.972436
H	-3.621523	0.485061	-2.219341
H	0.400951	-0.905594	-1.593590
H	-1.419622	-0.205257	-3.120725
H	-3.107142	0.037091	2.219197
O	-4.194008	0.513717	0.475220
C	-5.271763	0.944832	-0.336839
H	-4.991580	1.838844	-0.91044
H	-5.583740	0.145519	-1.02289
H	-6.090120	1.185236	0.345214

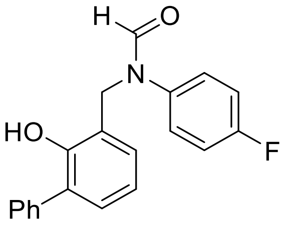

1-Ph (**Open**)

C	-4.859191	0.170839	0.334339
C	-3.651952	-2.290591	-0.218561
C	-5.644464	-0.940452	0.042462
C	-3.460637	0.063627	0.346149
C	-2.867920	-1.174967	0.067078
C	-5.029011	-2.152408	-0.229704
F	-5.793461	-3.229164	-0.512728
N	-2.641631	1.178970	0.676446
C	-3.004703	2.479007	0.419648
O	-4.041660	2.856079	-0.082365
C	-1.283133	0.950156	1.149916
C	-0.255361	0.796187	0.046463
C	1.730849	0.475345	-1.890985
C	1.051932	0.452100	0.416093
C	-0.549062	0.980636	-1.301374
C	0.439183	0.817868	-2.273399
C	2.063660	0.294944	-0.542901
H	-5.332915	1.122311	0.547344
H	-3.200786	-3.256213	-0.436159
H	-6.730193	-0.872112	0.031458
H	-1.786237	-1.283203	0.055621
H	-2.213040	3.187530	0.731490
H	-1.278509	0.065284	1.795814
H	-0.997576	1.791249	1.793897
H	2.513734	0.361060	-2.640242
H	-1.564891	1.239922	-1.598414
H	0.199380	0.962058	-3.324856
O	1.259330	0.261548	1.753030
H	2.159815	-0.055785	1.899813
C	3.448667	-0.072883	-0.146428
C	6.073043	-0.774404	0.571135
C	4.145992	0.661920	0.824461
C	4.090166	-1.159296	-0.756240
C	5.392057	-1.505603	-0.402575
C	5.447328	0.310917	1.182622
H	3.674767	1.534607	1.279123
H	3.553400	-1.740161	-1.505980
H	5.875589	-2.354235	-0.884589
H	5.978163	0.895485	1.932909
H	7.089277	-1.048173	0.850187

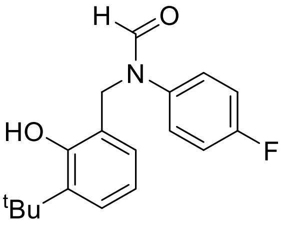

1-^{t}Bu (**Open**)

C	3.023084	-0.913282	-0.161107
C	5.742595	-0.303294	-0.090467
C	3.957490	-1.940379	-0.044180
C	3.437199	0.421836	-0.245807
C	4.807269	0.718942	-0.213772
C	5.302679	-1.615354	-0.005882
N	2.471644	1.454523	-0.402508
C	1.169372	1.138891	-0.974614
C	0.135507	0.682472	0.035882
C	-1.866291	-0.148816	1.772959
C	-1.132196	0.329661	-0.447196
C	0.387961	0.614690	1.401731
C	-0.614252	0.196950	2.272279
C	-2.169377	-0.094233	0.405444
C	-3.557180	-0.476851	-0.128287
O	-1.256251	0.442929	-1.808076
C	2.648198	2.720656	0.100260
O	3.614548	3.128660	0.708449
F	6.213233	-2.605388	0.116544
H	1.965126	-1.164459	-0.171292
H	6.809038	-0.089691	-0.065013
H	3.647673	-2.980876	0.024218
H	5.140495	1.748722	-0.278767
H	0.796017	2.031076	-1.492522
H	1.303689	0.378359	-1.751948
H	-2.628132	-0.471794	2.476799
H	1.373481	0.879833	1.783482
H	-0.421512	0.139391	3.341604
H	-2.139275	0.183824	-2.081045
H	1.773702	3.365552	-0.113814
C	-3.453873	-1.684607	-1.081732
H	-2.810913	-1.523551	-1.955758
H	-4.451732	-1.951302	-1.452762
H	-3.044162	-2.547353	-0.542833
C	-4.221066	0.729822	-0.822554
H	-3.654227	1.138177	-1.667948
H	-4.348748	1.546865	-0.102242
H	-5.211438	0.441925	-1.197788
C	-4.498111	-0.898614	1.007120
H	-4.117895	-1.779083	1.538957
H	-5.476338	-1.156984	0.583743
H	-4.647028	-0.087844	1.730446

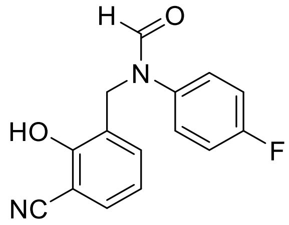

1-CN (**Open**)

C	-3.315655	-0.228763	0.619921
C	-2.558806	-0.029440	-2.065870
C	-4.280361	-0.031646	-0.362946
C	-1.960883	-0.322609	0.269553
C	-1.594394	-0.220696	-1.078617
C	-3.887191	0.065600	-1.688959
F	-4.826084	0.253466	-2.639512
N	-0.963091	-0.561204	1.256663
C	-1.075740	-0.133944	2.559652
O	-2.003924	0.476412	3.042063
C	0.308172	-1.145795	0.859866
C	1.340861	-0.135966	0.400030
C	3.343080	1.651756	-0.451446
C	2.577674	-0.622812	-0.043745
C	1.129940	1.238693	0.408555
C	2.119979	2.133585	-0.012914
C	3.574045	0.269034	-0.468222
O	2.748986	-1.965626	-0.035936
H	-3.615360	-0.306146	1.658992
H	-2.284390	0.049997	-3.115470
H	-5.335153	0.040349	-0.106683
H	-0.550713	-0.279801	-1.377796
H	-0.184589	-0.414357	3.155020
H	0.714049	-1.705941	1.711407
H	0.128803	-1.885929	0.071753
H	4.129334	2.325315	-0.785096
H	0.166485	1.622426	0.743785
H	1.928261	3.203928	0.002518
H	3.630937	-2.198805	-0.360652
C	4.817584	-0.294289	-0.913007
N	5.778143	-0.846793	-1.252908

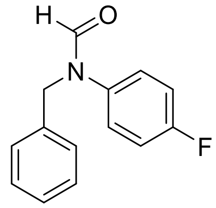

1-control (**Open**)

C	2.874607	-1.011707	-0.120031
C	2.486218	1.743148	0.163418
C	3.942820	-0.137154	-0.290409
C	1.599082	-0.516660	0.188422
C	1.416903	0.865810	0.327167
C	3.731882	1.225674	-0.146371
F	4.770027	2.072628	-0.309995
N	0.503388	-1.398395	0.407854
C	0.387789	-2.619734	-0.212658
O	1.171923	-3.109436	-0.995847
C	-0.632079	-0.938489	1.194129
C	-1.671747	-0.145205	0.420573
C	-3.663250	1.289191	-0.931533
C	-2.675629	0.518845	1.132864
C	-1.669319	-0.079891	-0.972966
C	-2.661693	0.635365	-1.645467
C	-3.667584	1.230047	0.463090
H	3.031271	-2.078801	-0.230321
H	2.354900	2.817858	0.267883
H	4.937757	-0.507532	-0.528105
H	0.435900	1.277999	0.550699
H	-0.543568	-3.139283	0.085980
H	-1.112503	-1.823298	1.630704
H	-0.252048	-0.341644	2.031843
H	-4.436493	1.846778	-1.457830
H	-0.884652	-0.577834	-1.541803
H	-2.647641	0.681592	-2.733506
H	-4.444671	1.742356	1.028795
H	-2.681589	0.477773	2.223421

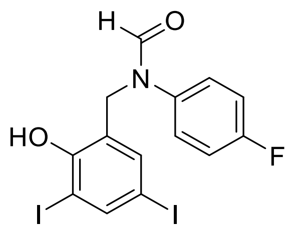

1-I_2 **(Open)**

C	-4.982914	-0.993780	0.667063
C	-4.078707	-1.145712	-1.976688
C	-5.892574	-0.939164	-0.383871
C	-3.610624	-1.117352	0.405255
C	-3.169611	-1.192724	-0.921746
C	-5.426321	-1.014061	-1.687766
F	-6.312329	-0.957681	-2.703882
N	-2.667721	-1.207866	1.467929
C	-2.857024	-0.610444	2.692818
O	-3.809559	0.056477	3.030640
C	-1.390424	-1.867378	1.244881
C	-0.283270	-0.956233	0.746151
C	1.861552	0.631330	-0.123574
C	0.986073	-1.530681	0.563452
C	-0.470770	0.398818	0.492136
C	0.598336	1.184708	0.057997
C	2.045154	-0.728749	0.131169
O	1.086222	-2.855022	0.835502
H	-5.338234	-0.933249	1.689270
H	-3.748122	-1.204560	-3.011064
H	-6.960176	-0.844927	-0.197386
H	-2.109599	-1.276902	-1.149524
H	-2.006193	-0.808560	3.373696
H	-1.066891	-2.325199	2.187795
H	-1.537263	-2.698622	0.546014
H	2.696760	1.241433	-0.457008
H	-1.456574	0.839725	0.630132
H	1.994559	-3.156095	0.692188
I	0.299783	3.237946	-0.326711
I	3.959941	-1.585505	-0.132294

Closed-Nonbonded Conformer Geometries

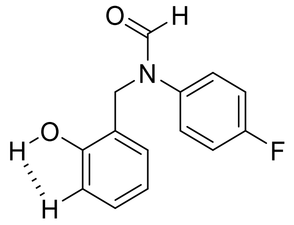

1-H (**Closed-Nonbonded**)

C	-1.586024	-0.812874	0.139028
C	-3.165943	1.371301	-0.608389
C	-2.583656	-0.984059	-0.819464
C	-1.386885	0.434969	0.733342
C	-2.176222	1.523214	0.357190
C	-3.348407	0.116123	-1.175764
F	-4.307439	-0.037158	-2.112428
N	-0.337951	0.616102	1.682146
C	-0.310859	-0.132539	2.824887
O	0.649253	-0.233051	3.564252
C	0.912316	1.227338	1.205424
C	1.641329	0.290887	0.270567
C	2.869011	-1.555887	-1.445275
C	1.430350	0.352345	-1.113172
C	2.475708	-0.709487	0.774457
C	3.090769	-1.631772	-0.070957
C	2.041411	-0.565838	-1.969060
O	0.606083	1.338731	-1.581172
H	-0.941491	-1.646011	0.418296
H	-3.796732	2.200982	-0.920100
H	-2.763790	-1.945676	-1.294926
H	-2.008016	2.491683	0.825458
H	-1.279261	-0.627503	3.028599
H	0.663269	2.165860	0.702180
H	1.518174	1.431755	2.091863
H	3.342222	-2.269318	-2.118546
H	2.624437	-0.762259	1.853196
H	3.740155	-2.402638	0.339563
H	1.866859	-0.503234	-3.044316
H	0.521330	1.258016	-2.536878

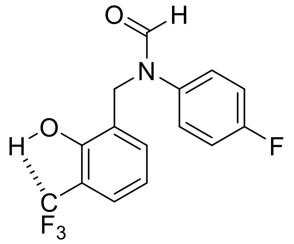

1-CF_3 (**Closed-Nonbonded**)

C	-2.469146	0.685224	1.364507
C	-2.100123	1.626078	-1.246744
C	-2.843676	1.985699	1.044108
C	-1.907620	-0.143661	0.391679
C	-1.718299	0.328979	-0.907952
C	-2.650840	2.427333	-0.258650
N	-1.465078	-1.452826	0.747206
C	-0.190253	-1.568614	1.469329
C	0.967795	-1.236740	0.556767
C	3.004564	-0.610747	-1.265787
C	1.431222	0.082783	0.448213
C	1.538433	-2.222734	-0.248591
C	2.553652	-1.921350	-1.155530
C	2.447402	0.388712	-0.469539
C	2.916327	1.805440	-0.590877
F	3.370627	2.290704	0.591810
F	1.907653	2.638495	-0.951078
F	3.896902	1.962717	-1.485563
O	0.851020	1.008809	1.256098
C	-1.936336	-2.546283	0.075379
O	-1.438589	-3.654684	0.126353
F	-3.016932	3.685620	-0.574113
H	-2.608211	0.304524	2.375102
H	-1.965738	2.021969	-2.250830
H	-3.285584	2.654585	1.779179
H	-1.251910	-0.318725	-1.649669
H	-0.220943	-0.893022	2.327971
H	-0.114274	-2.602695	1.814377
H	3.793012	-0.350055	-1.968435
H	1.156860	-3.240045	-0.164051
H	2.988987	-2.702880	-1.774239
H	1.207954	1.890933	1.102143
H	-2.848859	-2.323544	-0.508572

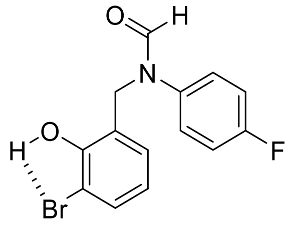

1-Br (**Closed-Nonbonded**)

C	-2.184425	0.898370	1.250202
C	-1.714609	1.842026	-1.344223
C	-2.527881	2.205995	0.922386
C	-1.604768	0.063711	0.293007
C	-1.363385	0.538203	-0.997172
C	-2.285872	2.648165	-0.371657
F	-2.622087	3.913356	-0.694649
N	-1.197432	-1.255338	0.654496
C	-1.674030	-2.333321	-0.038212
O	-1.199022	-3.451680	0.010272
C	0.064378	-1.397244	1.394524
C	1.238869	-1.036888	0.513465
C	3.302213	-0.336081	-1.255282
C	1.722143	0.280925	0.493179
C	1.794128	-1.983155	-0.348248
C	2.820655	-1.641950	-1.227817
C	2.750692	0.609069	-0.397516
H	-2.360884	0.517189	2.254901
H	-1.542165	2.239043	-2.342233
H	-2.980935	2.880259	1.645702
H	-0.881001	-0.115136	-1.723701
H	-2.569474	-2.086401	-0.639305
H	0.130351	-2.442845	1.705198
H	0.020875	-0.749471	2.273957
H	4.101069	-0.043991	-1.933525
H	1.394243	-2.996732	-0.329181
H	3.247319	-2.388809	-1.894007
O	1.159982	1.177079	1.340032
H	1.577004	2.041213	1.220488
Br	3.384049	2.404440	-0.435081

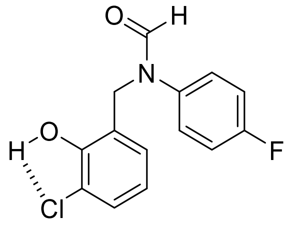

1-Cl (**Closed-Nonbonded**)

C	-1.537040	-0.212746	0.974190
C	-3.008322	-1.531628	-1.005171
C	-2.229591	-1.378808	1.296835
C	-1.589219	0.301200	-0.322684
C	-2.324332	-0.359720	-1.308461
C	-2.946068	-2.015778	0.295511
F	-3.609324	-3.151180	0.593252
N	-0.841661	1.467572	-0.661570
C	-1.047137	2.638181	0.012743
O	-0.305556	3.600695	-0.031458
C	0.423422	1.283474	-1.385370
C	1.477967	0.691257	-0.478395
C	3.342196	-0.406558	1.310883
C	1.635268	-0.700813	-0.400714
C	2.255574	1.513322	0.338481
C	3.183807	0.974643	1.228297
C	2.568679	-1.227209	0.499100
O	0.871395	-1.481332	-1.204552
H	-0.935680	0.295876	1.727296
H	-3.588686	-2.067975	-1.752823
H	-2.208321	-1.800094	2.299577
H	-2.355143	0.049752	-2.317086
H	-1.988150	2.632151	0.594594
H	0.235134	0.630586	-2.242009
H	0.736384	2.271721	-1.732382
H	4.058971	-0.855138	1.995522
H	2.109315	2.591275	0.276124
H	3.785869	1.626242	1.857904
H	1.081147	-2.410848	-1.042681
Cl	2.749104	-2.968118	0.585043

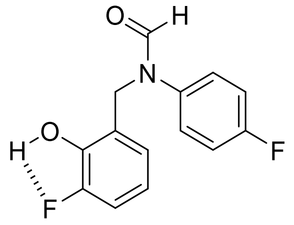

1-F (**Closed-Nonbonded**)

C	1.672594	0.180202	0.770488
C	2.865162	1.346011	-1.474341
C	2.441072	1.333328	0.923099
C	1.512720	-0.398401	-0.490239
C	2.108595	0.187217	-1.608873
C	3.016206	1.893876	-0.206670
F	3.749157	3.017324	-0.072521
N	0.692511	-1.553851	-0.649466
C	0.948055	-2.686759	0.071458
O	0.177640	-3.620115	0.188565
C	-0.649001	-1.365438	-1.217574
C	-1.544949	-0.641404	-0.240051
C	-3.107078	0.715631	1.672012
C	-1.652775	0.750986	-0.283770
C	-2.220877	-1.340711	0.766231
C	-2.995537	-0.675756	1.713620
C	-2.433629	1.394513	0.675773
O	-1.003898	1.456969	-1.248712
H	1.176570	-0.268358	1.630668
H	3.341383	1.823977	-2.327488
H	2.585666	1.802404	1.893656
H	1.973447	-0.270333	-2.587442
H	1.956939	-2.680276	0.524420
H	-0.550049	-0.799397	-2.148173
H	-1.045922	-2.361176	-1.430370
H	-3.701835	1.274026	2.391887
H	-2.115568	-2.424613	0.799926
H	-3.516649	-1.235758	2.486982
H	-1.174406	2.397568	-1.113157
F	-2.505544	2.748312	0.590063

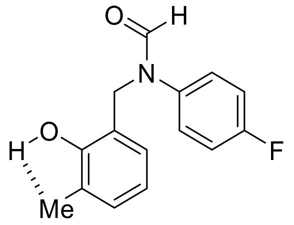

1-Me (**Closed-Nonbonded**)

C	-2.587344	-0.391228	1.240615
C	-2.391890	0.859544	-1.256508
C	-3.380393	0.718575	0.964796
C	-1.697915	-0.876150	0.280777
C	-1.597169	-0.247237	-0.961573
C	-3.263179	1.320417	-0.281234
N	-0.840391	-1.972369	0.595033
C	0.386730	-1.670915	1.349964
C	1.331071	-0.842128	0.510711
C	2.932744	0.695094	-1.183964
C	1.297989	0.555876	0.567226
C	2.184901	-1.457721	-0.407210
C	2.987548	-0.696628	-1.253223
C	2.092805	1.344143	-0.279201
C	2.015772	2.846414	-0.199838
O	0.447478	1.124480	1.476642
C	-0.893903	-3.117410	-0.149369
O	-0.053452	-3.995977	-0.133057
F	-4.029167	2.397295	-0.553584
H	-2.651931	-0.889014	2.206851
H	-2.337480	1.367050	-2.217154
H	-4.084768	1.117014	1.691420
H	-0.875115	-0.618407	-1.688266
H	0.102396	-1.141471	2.263346
H	0.842030	-2.631659	1.603198
H	3.555726	1.297147	-1.845790
H	2.196230	-2.546573	-0.458057
H	3.652340	-1.182693	-1.964717
H	0.476981	2.083136	1.395667
H	-1.812783	-3.178013	-0.763009
H	0.997480	3.209587	-0.407261
H	2.313290	3.215752	0.792907
H	2.683373	3.304069	-0.936140

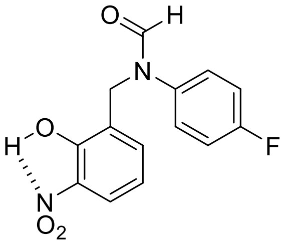

1-NO$_2$ (**Closed-Nonbonded**)

C	-2.555784	0.383283	1.219745
C	-2.429005	1.299162	-1.423052
C	-3.191663	1.568919	0.873480
C	-1.848731	-0.341924	0.258319
C	-1.780047	0.121034	-1.057213
C	-3.116582	2.000939	-0.445602
N	-1.157237	-1.529499	0.637352
C	0.056623	-1.404123	1.452963
C	1.229075	-0.940784	0.617780
C	3.350170	-0.095380	-1.018787
C	1.476999	0.440372	0.474763
C	2.033875	-1.856223	-0.048620
C	3.093650	-1.444271	-0.867986
C	2.548320	0.837800	-0.351578
O	0.681456	1.288229	1.136673
N	2.858428	2.246758	-0.536939
O	2.160409	3.080313	0.048464
O	3.782759	2.552842	-1.254623
F	-3.738355	3.147041	-0.783877
C	-1.367488	-2.698098	-0.038013
O	-0.657876	-3.681893	0.052987
H	-2.603087	0.013378	2.242742
H	-2.394295	1.681305	-2.440832
H	-3.747681	2.156070	1.601074
H	-1.202181	-0.438242	-1.792396
H	-0.143698	-0.695278	2.260686
H	0.263900	-2.392833	1.870293
H	4.161600	0.270050	-1.642140
H	1.811420	-2.916629	0.069000
H	3.710016	-2.179171	-1.380099
H	0.990355	2.195139	0.934447
H	-2.275343	-2.668288	-0.669013

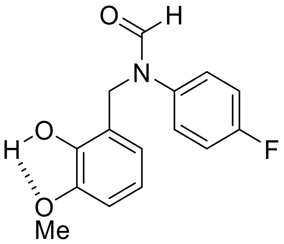

1-OMe (**Closed-Nonbonded**)

C	2.367242	-1.099474	0.279364
C	2.367305	0.822408	2.304760
C	2.617065	-1.487441	1.594577
C	2.150551	0.244902	-0.035086
C	2.142393	1.200342	0.985312
C	2.609805	-0.515812	2.582047
F	2.832463	-0.884298	3.860931
N	1.907160	0.637824	-1.379500
C	2.663910	0.134663	-2.400729
O	2.415997	0.268985	-3.583461
C	0.681214	1.382491	-1.685905
C	-0.539088	0.559925	-1.348713
C	-2.741878	-1.047582	-0.667127
C	-1.309727	0.862251	-0.232615
C	-0.876555	-0.557257	-2.126686
C	-1.963217	-1.351142	-1.792207
C	-2.413197	0.056327	0.107989
O	-1.001744	1.940772	0.541702
H	2.316357	-1.849768	-0.507904
H	2.366552	1.548719	3.114548
H	2.791169	-2.528015	1.859385
H	1.954983	2.245389	0.748214
H	3.559472	-0.401410	-2.036321
H	0.677008	2.323223	-1.127805
H	0.717627	1.601094	-2.756528
H	-3.592625	-1.673697	-0.411646
H	-0.265726	-0.789410	-2.999564
H	-2.222186	-2.212853	-2.403824
H	-1.642937	1.974316	1.264400
O	-3.075352	0.471050	1.231343
C	-4.163899	-0.324681	1.663043
H	-4.955972	-0.346413	0.902103
H	-3.832193	-1.348959	1.881607
H	-4.541976	0.143530	2.574295

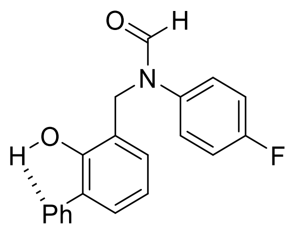

1-Ph (**Closed-Nonbonded**)

C	-2.875073	1.331288	-1.140218
C	-1.881783	-1.173228	-1.902654
C	-2.587030	1.085442	-2.478890
C	-2.663156	0.339626	-0.181300
C	-2.161846	-0.906567	-0.562983
C	-2.094485	-0.164780	-2.830162
F	-1.809575	-0.405934	-4.127170
N	-2.884513	0.627074	1.198763
C	-3.758462	-0.134771	1.923731
O	-3.824246	-0.150796	3.137331
C	-1.821922	1.357009	1.911113
C	-0.568955	0.516147	1.976096
C	1.651749	-1.177822	1.981177
C	0.388368	0.599435	0.955736
C	-0.395534	-0.422763	2.993658
C	0.715940	-1.264227	3.007723
C	1.500726	-0.260201	0.936682
H	-3.258100	2.301606	-0.827893
H	-1.495852	-2.136904	-2.228334
H	-2.739744	1.839311	-3.247691
H	-1.971344	-1.660609	0.200140
H	-4.434060	-0.728865	1.279311
H	-2.203143	1.561028	2.914615
H	-1.637883	2.296954	1.383484
H	2.511493	-1.847638	1.964669
H	-1.155959	-0.493309	3.771764
H	0.844811	-1.991473	3.806867
O	0.189132	1.550345	-0.002455
H	0.936459	1.554193	-0.613678
C	2.492458	-0.209331	-0.169584
C	4.377359	-0.161300	-2.251350
C	2.082584	-0.305824	-1.508633
C	3.860184	-0.092554	0.109038
C	4.795277	-0.071113	-0.923094
C	3.018916	-0.279256	-2.541589
H	1.021921	-0.429272	-1.736737
H	4.185765	-0.008963	1.145665
H	5.855430	0.022208	-0.690676
H	2.684955	-0.362626	-3.574919
H	5.109137	-0.141538	-3.057553

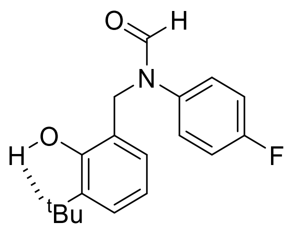

1-^{t}Bu (**Closed-Nonbonded**)

C	3.212050	-1.195785	-0.326710
C	2.320606	1.238843	-1.381511
C	3.359927	-0.942617	-1.686813
C	2.616054	-0.246853	0.505373
C	2.167476	0.965338	-0.023561
C	2.907482	0.272573	-2.184591
N	2.396061	-0.548444	1.882694
C	1.202630	-1.345701	2.216689
C	-0.050837	-0.564125	1.897106
C	-2.208510	1.046951	1.216301
C	-0.637356	-0.656680	0.626455
C	-0.567717	0.337502	2.826777
C	-1.652496	1.139154	2.491007
C	-1.723639	0.160466	0.247132
C	-2.323878	0.105938	-1.165120
O	-0.064298	-1.579492	-0.207367
C	2.955200	0.238204	2.850853
O	2.636734	0.229219	4.024025
F	3.042909	0.522177	-3.503733
H	3.551178	-2.138893	0.098678
H	1.982392	2.174759	-1.820927
H	3.818682	-1.663241	-2.360039
H	1.675720	1.682910	0.632699
H	1.245537	-2.280222	1.651750
H	1.260277	-1.555484	3.287562
H	-3.048911	1.692442	0.976208
H	-0.095022	0.411615	3.806052
H	-2.064398	1.840792	3.213427
H	-0.480219	-1.545671	-1.071364
H	3.764379	0.881142	2.455643
C	-2.906900	-1.290598	-1.462770
H	-2.185282	-2.113879	-1.403752
H	-3.338027	-1.303058	-2.471932
H	-3.703258	-1.517820	-0.744014
C	-1.260763	0.511864	-2.207072
H	-0.341700	-0.086390	-2.186163
H	-0.956789	1.551383	-2.033030
H	-1.683640	0.441339	-3.217254
C	-3.484030	1.097645	-1.321767
H	-4.304506	0.870750	-0.630170
H	-3.875516	1.031189	-2.344189
H	-3.157601	2.130757	-1.152582

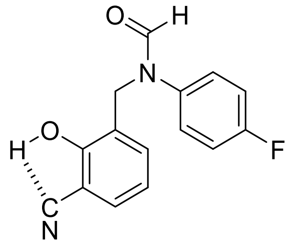

1-CN (**Closed-Nonbonded**)

C	-2.285552	1.249712	-0.837566
C	-1.861553	-1.348886	-1.790522
C	-2.628135	0.934273	-2.148422
C	-1.729508	0.277972	-0.003400
C	-1.509900	-1.014291	-0.483498
C	-2.410205	-0.362534	-2.595454
F	-2.747524	-0.673679	-3.861919
N	-1.328251	0.623861	1.321857
C	-1.806845	-0.081438	2.391223
O	-1.333327	-0.043447	3.510712
C	-0.079592	1.379231	1.483253
C	1.112944	0.517590	1.136070
C	3.239971	-1.203840	0.474002
C	1.633457	0.516713	-0.165565
C	1.667143	-0.345974	2.080772
C	2.723464	-1.203933	1.761104
C	2.696772	-0.344468	-0.489834
H	-2.447589	2.255650	-0.453292
H	-1.706473	-2.348452	-2.190654
H	-3.064277	1.669141	-2.821363
H	-1.047294	-1.755276	0.167901
H	-2.700813	-0.681090	2.136883
H	-0.030341	1.689463	2.529917
H	-0.123126	2.260359	0.837441
H	4.063502	-1.858342	0.195577
H	1.243319	-0.346718	3.085226
H	3.139406	-1.866868	2.516407
O	1.084207	1.355170	-1.074258
H	1.522697	1.260933	-1.931702
C	3.193884	-0.304785	-1.836296
N	3.519538	-0.206047	-2.944599

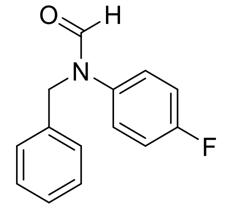

1-control (**Closed-Nonbonded**)

C	-2.327605	-0.644774	1.106806
C	-2.447064	0.839283	-1.263180
C	-3.366005	0.262039	0.916229
C	-1.347698	-0.810540	0.125234
C	-1.404356	-0.060121	-1.051248
C	-3.403782	0.983841	-0.269747
F	-4.408426	1.862049	-0.458973
N	-0.261670	-1.706439	0.351616
C	-0.034039	-2.745359	-0.507063
O	0.978874	-3.416816	-0.528754
C	0.823133	-1.251350	1.231247
C	1.585882	-0.111600	0.595653
C	2.892306	1.995331	-0.704739
C	2.514459	-0.376138	-0.417890
C	1.317619	1.212073	0.949961
C	1.968366	2.263817	0.303652
C	3.164576	0.673419	-1.063892
H	-2.275807	-1.236653	2.019698
H	-2.515490	1.433909	-2.171484
H	-4.144412	0.408236	1.661869
H	-0.612950	-0.170839	-1.791835
H	-0.892425	-2.933866	-1.178996
H	1.471328	-2.115518	1.402117
H	0.379965	-0.934540	2.182048
H	3.402185	2.814367	-1.210204
H	2.714533	-1.410631	-0.698766
H	0.586956	1.422523	1.732388
H	1.752138	3.293044	0.587252
H	3.889410	0.461253	-1.848998

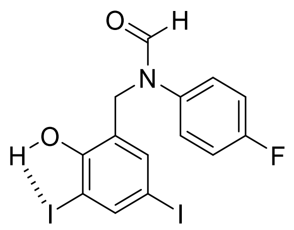

1-I_2 (**Closed-Nonbonded**)

C	3.060336	-1.507215	1.177129
C	5.152746	-0.208943	-0.132429
C	4.080768	-0.920616	1.922179
C	3.087123	-1.468401	-0.219413
C	4.140863	-0.815057	-0.866549
C	5.108850	-0.276835	1.253536
F	6.096083	0.305297	1.966846
N	2.058079	-2.107299	-0.975528
C	1.769491	-3.434274	-0.778219
O	0.841125	-4.029284	-1.286841
C	1.208711	-1.332210	-1.897891
C	0.082208	-0.596123	-1.196233
C	-2.012366	0.729003	0.128062
C	0.169380	0.787741	-0.963569
C	-1.046549	-1.296570	-0.771639
C	-2.081535	-0.638552	-0.114728
C	-0.886585	1.426449	-0.299895
O	1.279332	1.429788	-1.403498
H	2.229106	-1.984705	1.683651
H	5.977668	0.299268	-0.618114
H	4.075005	-0.944793	3.005642
H	4.174824	-0.791047	-1.949869
H	2.494831	-3.923883	-0.106498
H	1.834107	-0.629869	-2.444771
H	0.792278	-2.048872	-2.604962
H	-2.815000	1.244995	0.637885
H	-1.091817	-2.362809	-0.957277
H	1.223855	2.371992	-1.183553
I	-0.764858	3.533424	0.083284
I	-3.796414	-1.736069	0.541159

Closed-Bonded Conformer Geometries

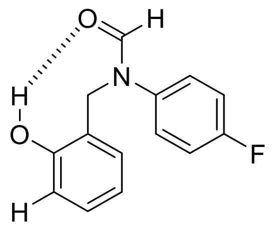

1-H (**Closed-Bonded**)

C	-0.151597	-1.757079	1.163826
C	0.061722	-3.586471	-0.941570
C	0.568531	-2.936550	1.348429
C	-0.783042	-1.502588	-0.054310
C	-0.670825	-2.415666	-1.106079
C	0.661411	-3.824925	0.288855
F	1.360950	-4.963228	0.455297
N	-1.523731	-0.298042	-0.238304
C	-2.526211	0.028469	0.609679
O	-3.069626	1.125787	0.649062
C	-1.000093	0.721350	-1.164439
C	0.115912	1.542823	-0.565984
C	2.233392	3.058643	0.462202
C	-0.142785	2.746880	0.114070
C	1.440469	1.110375	-0.696522
C	2.501343	1.850038	-0.184755
C	0.926269	3.503936	0.610391
O	-1.386925	3.251959	0.285607
H	-0.212830	-1.018819	1.961892
H	0.163371	-4.317629	-1.740385
H	1.066519	-3.162747	2.288577
H	-1.170144	-2.210403	-2.051852
H	-2.836231	-0.803617	1.264374
H	-0.632122	0.191912	-2.048589
H	-1.844307	1.350490	-1.463545
H	3.052311	3.658926	0.856785
H	1.636271	0.174841	-1.223513
H	3.524397	1.497554	-0.299204
H	0.699212	4.440661	1.115976
H	-2.061608	2.543124	0.304027

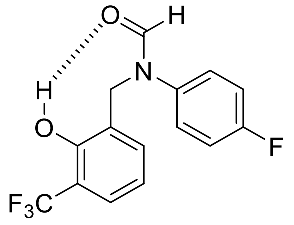

1-CF_3 (**Closed-Bonded**)

C	-0.594805	3.131046	0.853577
C	0.868862	3.121511	-1.532631
C	0.232889	4.207866	0.551959
C	-0.690582	2.051316	-0.027181
C	0.047768	2.041783	-1.211086
C	0.945364	4.180051	-0.640311
N	-1.516046	0.935787	0.309385
C	-1.041525	0.000124	1.342589
C	0.005621	-0.959003	0.830356
C	1.992224	-2.731805	-0.037233
C	-0.359812	-2.205687	0.288096
C	1.358334	-0.618388	0.907583
C	2.354507	-1.486996	0.473719
C	0.651424	-3.088749	-0.128126
C	0.250490	-4.427927	-0.683435
F	-0.484692	-4.315251	-1.803381
F	-0.469124	-5.150226	0.192348
F	1.330056	-5.172982	-1.004681
O	-1.632764	-2.624047	0.180968
C	-2.559871	0.584927	-0.475334
O	-3.176998	-0.468958	-0.370420
F	1.742356	5.222165	-0.939479
H	-1.179494	3.127691	1.772393
H	1.453463	3.144072	-2.449496
H	0.323985	5.065230	1.215018
H	-0.011813	1.177916	-1.871417
H	-0.619277	0.603386	2.152130
H	-1.916253	-0.533629	1.727379
H	2.751582	-3.435263	-0.370877
H	1.630256	0.350538	1.329603
H	3.402752	-1.205713	0.544851
H	-2.259454	-1.871538	0.094346
H	-2.829420	1.350752	-1.221211

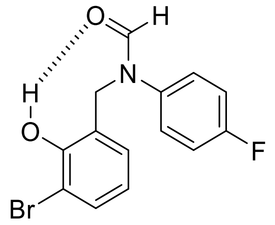

1-Br (**Closed-Bonded**)

C	-2.648083	-0.635708	0.783606
C	-2.636625	0.883344	-1.567909
C	-3.733311	0.184764	0.490956
C	-1.560307	-0.698383	-0.089843
C	-1.550051	0.067536	-1.256186
C	-3.704031	0.925359	-0.683898
F	-4.754516	1.715082	-0.974091
N	-0.436718	-1.518465	0.234955
C	-0.080576	-2.547783	-0.566213
O	0.981241	-3.153960	-0.480388
C	0.508102	-1.035296	1.257048
C	1.441394	0.032279	0.736457
C	3.156014	2.056319	-0.154205
C	2.686214	-0.315372	0.174902
C	1.068970	1.376256	0.823150
C	1.911200	2.389287	0.376854
C	3.532793	0.722441	-0.250017
H	-2.644629	-1.240716	1.689051
H	-2.658167	1.489051	-2.470863
H	-4.597880	0.249245	1.147637
H	-0.679479	0.033593	-1.909474
H	-0.851226	-2.817102	-1.307413
H	1.061203	-1.903973	1.627409
H	-0.090517	-0.628193	2.077757
H	3.841537	2.827915	-0.497953
H	0.101119	1.625748	1.260220
H	1.612291	3.432626	0.451693
O	3.121469	-1.582953	0.066663
H	2.375324	-2.215976	-0.019708
Br	5.227246	0.283033	-0.970195

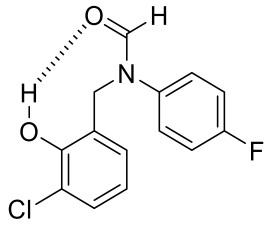

1-Cl (**Closed-Bonded**)

C	-1.551841	-0.044132	1.217134
C	-3.681265	-0.166984	-0.593374
C	-2.638079	-0.872801	1.490721
C	-1.535155	0.731679	0.057050
C	-2.596826	0.666837	-0.847618
C	-3.678538	-0.918549	0.575217
F	-4.726795	-1.724114	0.825888
N	-0.405436	1.557250	-0.230261
C	-0.067407	2.574025	0.595950
O	1.000657	3.172891	0.552507
C	0.567370	1.078226	-1.227309
C	1.453554	-0.025851	-0.700909
C	3.075937	-2.117264	0.206389
C	2.668661	0.274340	-0.055299
C	1.062313	-1.358037	-0.861087
C	1.858239	-2.404439	-0.407810
C	3.471315	-0.796826	0.374531
O	3.125495	1.524160	0.137656
H	-0.700172	-0.009271	1.895165
H	-4.525177	-0.234643	-1.276261
H	-2.678536	-1.486665	2.387543
H	-2.574076	1.278842	-1.748158
H	-0.860594	2.840016	1.314718
H	-0.006860	0.707458	-2.082060
H	1.153068	1.944203	-1.551620
H	3.730293	-2.912007	0.558895
H	0.116748	-1.570637	-1.361725
H	1.544189	-3.437614	-0.540559
H	2.397476	2.181467	0.163003
Cl	5.001443	-0.451556	1.131681

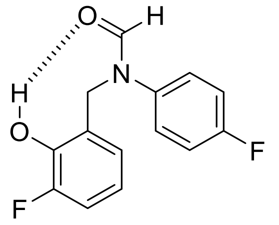

1-F (**Closed-Bonded**)

C	-1.654530	0.088777	1.216397
C	-3.596013	-0.209190	-0.774267
C	-2.774477	-0.703006	1.463193
C	-1.514112	0.747404	-0.005815
C	-2.483588	0.594601	-1.000260
C	-3.720381	-0.837928	0.458614
F	-4.799979	-1.609170	0.683269
N	-0.360778	1.549996	-0.253245
C	-0.045460	2.571999	0.576676
O	1.032195	3.154609	0.577614
C	0.644449	1.057459	-1.209336
C	1.496716	-0.054010	-0.645047
C	3.063178	-2.159739	0.340275
C	2.682290	0.236899	0.050140
C	1.102934	-1.386646	-0.818995
C	1.870842	-2.437165	-0.331706
C	3.445155	-0.843661	0.516463
O	3.165883	1.475319	0.267494
H	-0.872792	0.184565	1.968460
H	-4.368211	-0.343496	-1.528205
H	-2.910722	-1.223026	2.408845
H	-2.369212	1.115980	-1.949710
H	-0.867553	2.858432	1.254176
H	0.103224	0.690835	-2.086826
H	1.252462	1.916766	-1.509824
H	3.703240	-2.948159	0.731902
H	0.178157	-1.594395	-1.358467
H	1.554387	-3.467493	-0.479100
H	2.450204	2.145114	0.275017
F	4.592494	-0.571671	1.162270

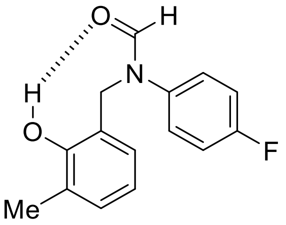

1-Me (**Closed-Bonded**)

C	-1.099243	-0.619454	-3.001656
C	1.361008	0.705038	-3.174619
C	-0.830238	0.188778	-4.101710
C	-0.148752	-0.765635	-1.988622
C	1.071723	-0.093823	-2.069963
C	0.399901	0.831135	-4.166113
N	-0.444999	-1.569858	-0.847453
C	-1.387565	-1.038499	0.152919
C	-0.781623	0.042206	1.015397
C	0.276323	2.077974	2.602282
C	-0.116338	-0.266032	2.212947
C	-0.886449	1.381229	0.620245
C	-0.358010	2.401097	1.400467
C	0.405069	0.758290	3.025883
C	1.082185	0.389635	4.316164
O	0.028116	-1.530991	2.680155
C	0.335509	-2.629219	-0.532641
O	0.293190	-3.226936	0.536041
F	0.667480	1.607164	-5.233298
H	-2.048080	-1.149252	-2.929530
H	2.305602	1.236695	-3.265386
H	-1.548934	0.317550	-4.907964
H	1.786643	-0.183769	-1.253339
H	-2.241600	-0.630376	-0.396851
H	-1.734503	-1.886466	0.751913
H	0.683922	2.871301	3.229701
H	-1.402371	1.617040	-0.312022
H	-0.447238	3.439094	1.085781
H	0.001177	-2.193433	1.960422
H	1.015129	-2.932169	-1.347260
H	0.393606	-0.156244	4.973233
H	1.932538	-0.278182	4.129601
H	1.436823	1.286114	4.835276

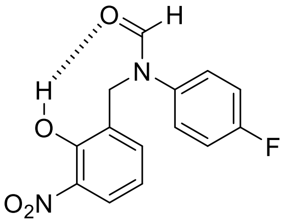

1-NO_2 (**Closed-Bonded**)

C	-2.948274	-0.647043	0.799808
C	-2.886656	0.961583	-1.491202
C	-4.003731	0.221800	0.542631
C	-1.864722	-0.713040	-0.078445
C	-1.829290	0.097195	-1.214552
C	-3.950556	1.006655	-0.603002
F	-4.971716	1.843868	-0.857940
N	-0.767551	-1.580891	0.211986
C	-0.430768	-2.575686	-0.641036
O	0.621147	-3.202335	-0.585945
C	0.177042	-1.179358	1.266511
C	1.110042	-0.076763	0.825145
C	2.783249	2.023383	0.032824
C	2.343805	-0.384343	0.209572
C	0.744766	1.253813	1.026460
C	1.563774	2.310327	0.630850
C	3.165658	0.699813	-0.149114
H	-2.966024	-1.287505	1.680413
H	-2.888270	1.601099	-2.370865
H	-4.864994	0.289806	1.203372
H	-0.962620	0.059821	-1.873206
H	-1.206061	-2.793890	-1.393945
H	0.727163	-2.075216	1.571159
H	-0.417496	-0.831742	2.116929
H	3.460180	2.807496	-0.298141
H	-0.211342	1.462695	1.508965
H	1.259491	3.341115	0.795287
O	2.746779	-1.632471	-0.047202
H	1.990967	-2.259189	-0.122239
N	4.486771	0.467239	-0.749230
O	5.152080	-0.449407	-0.321016
O	4.837155	1.241171	-1.624831

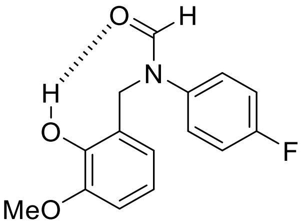

1-OMe (**Closed-Bonded**)

C	-2.251967	-0.016405	1.238126
C	-4.369253	-0.221038	-0.578043
C	-3.297499	-0.899994	1.499175
C	-2.270860	0.775781	0.088967
C	-3.328746	0.670271	-0.817317
C	-4.332360	-0.986731	0.580908
F	-5.339569	-1.848703	0.816987
N	-1.191041	1.666776	-0.184298
C	-0.850954	2.626953	0.706740
O	0.181741	3.283068	0.656768
C	-0.255462	1.323610	-1.268988
C	0.735430	0.255576	-0.872645
C	2.560095	-1.759438	-0.210799
C	1.983606	0.600953	-0.345741
C	0.396948	-1.096874	-1.043378
C	1.295902	-2.095421	-0.710990
C	2.909400	-0.424260	-0.031482
O	2.396799	1.872123	-0.148222
H	-1.402947	0.046787	1.917283
H	-5.206748	-0.321737	-1.264911
H	-3.307997	-1.526968	2.387729
H	-3.337863	1.295686	-1.708712
H	-1.611857	2.790316	1.489457
H	-0.858049	0.968003	-2.110505
H	0.248580	2.247944	-1.568135
H	3.263595	-2.549945	0.036068
H	-0.582003	-1.351615	-1.451418
H	1.032608	-3.142661	-0.846846
H	1.638689	2.462332	0.036453
O	4.105907	0.005510	0.442110
C	5.066151	-0.982000	0.752525
H	4.708685	-1.644560	1.554440
H	5.318880	-1.581888	-0.133990
H	5.952157	-0.441450	1.092684

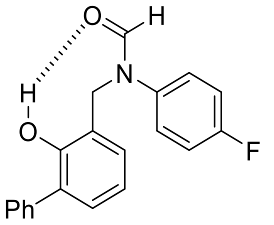

1-Ph (**Closed-Bonded**)

C	-4.297464	-0.671284	0.704276
C	-4.144290	1.037358	-1.507063
C	-5.334448	0.220593	0.452831
C	-3.183475	-0.709663	-0.137973
C	-3.103069	0.152375	-1.232620
C	-5.236984	1.054656	-0.654244
F	-6.242334	1.914284	-0.904213
N	-2.111131	-1.606228	0.146263
C	-1.696874	-2.498153	-0.782884
O	-0.656922	-3.141681	-0.707613
C	-1.265333	-1.338977	1.323094
C	-0.285982	-0.211003	1.101902
C	1.490737	1.904574	0.761051
C	1.007602	-0.457236	0.604616
C	-0.673081	1.097620	1.403512
C	0.205571	2.160872	1.232005
C	1.915586	0.611593	0.440496
H	-4.353462	-1.350460	1.553786
H	-4.108676	1.716864	-2.355656
H	-6.215604	0.269168	1.088559
H	-2.211839	0.141169	-1.858284
H	-2.401136	-2.617992	-1.623362
H	-0.754562	-2.274184	1.573215
H	-1.936758	-1.079264	2.147571
H	2.200448	2.724471	0.652696
H	-1.677696	1.274677	1.791484
H	-0.097874	3.175764	1.481095
O	1.436013	-1.703144	0.293151
H	0.696826	-2.290146	0.029856
C	3.292721	0.401123	-0.078545
C	5.920790	0.106875	-1.033019
C	3.812981	1.283791	-1.034814
C	4.109260	-0.637710	0.387654
C	5.412411	-0.779144	-0.082554
C	5.114627	1.138774	-1.509910
H	3.181592	2.081954	-1.425266
H	3.719356	-1.335119	1.125363
H	6.036153	-1.588574	0.295328
H	5.497578	1.830867	-2.258951
H	6.938741	-0.009461	-1.402834

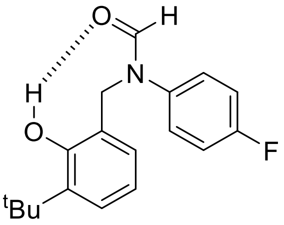

1-^{t}Bu (**Closed-Bonded**)

C	-4.239126	-0.610686	1.035486
C	-4.239551	0.917320	-1.309799
C	-5.304156	0.245074	0.774042
C	-3.174949	-0.702741	0.135634
C	-3.171238	0.068107	-1.027888
C	-5.281943	0.990203	-0.398474
N	-2.068194	-1.554054	0.431968
C	-1.116111	-1.126197	1.474192
C	-0.154413	-0.065112	0.994074
C	1.606398	1.924464	0.194084
C	1.108160	-0.406419	0.475919
C	-0.527367	1.278889	1.083711
C	0.344835	2.276622	0.676666
C	2.026812	0.596861	0.082351
C	3.422751	0.234932	-0.440625
O	1.494439	-1.706654	0.386583
C	-1.724599	-2.550159	-0.414801
O	-0.672921	-3.176868	-0.355232
F	-6.312325	1.817164	-0.656851
H	-4.232865	-1.218816	1.938793
H	-4.263441	1.527928	-2.209428
H	-6.148309	0.334264	1.454216
H	-2.316981	0.014835	-1.701240
H	-1.712500	-0.729048	2.301467
H	-0.591889	-2.020790	1.823838
H	2.280106	2.723658	-0.102695
H	-1.508349	1.533442	1.488512
H	0.059501	3.324508	0.746264
H	0.727777	-2.295499	0.225329
H	-2.497051	-2.773310	-1.169908
C	3.306643	-0.604074	-1.725376
H	2.780391	-1.544466	-1.544941
H	4.311952	-0.833137	-2.103739
H	2.771186	-0.040036	-2.500219
C	4.201165	-0.543987	0.634650
H	3.709966	-1.488207	0.881971
H	4.291430	0.056995	1.548804
H	5.212908	-0.761873	0.267230
C	4.235839	1.489975	-0.780509
H	3.757178	2.084523	-1.569218
H	5.224150	1.184054	-1.144733
H	4.384693	2.128316	0.099890

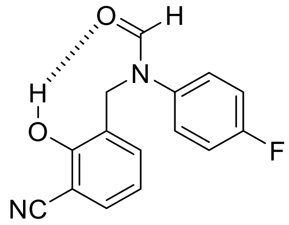

1-CN (**Closed-Bonded**)

C	-1.871846	0.114080	1.182110
C	-3.788098	-0.226465	-0.826654
C	-2.995264	-0.671995	1.431532
C	-1.715603	0.745460	-0.052642
C	-2.672747	0.571710	-1.056026
C	-3.928524	-0.828312	0.418210
F	-5.011117	-1.593237	0.645638
N	-0.561108	1.546462	-0.301915
C	-0.244952	2.575210	0.519063
O	0.834475	3.155271	0.511978
C	0.440990	1.056645	-1.261122
C	1.311796	-0.041051	-0.697933
C	2.921708	-2.134194	0.249347
C	2.504078	0.259072	-0.011504
C	0.943022	-1.375943	-0.872966
C	1.729966	-2.425537	-0.401575
C	3.308132	-0.804585	0.444448
O	2.951215	1.503458	0.206440
H	-1.101843	0.228287	1.943566
H	-4.551517	-0.375743	-1.586649
H	-3.144428	-1.170650	2.386616
H	-2.547959	1.073978	-2.014370
H	-1.064912	2.869607	1.194982
H	-0.102540	0.676408	-2.131212
H	1.038040	1.918648	-1.575465
H	3.569357	-2.928037	0.616252
H	0.016343	-1.594509	-1.406441
H	1.423130	-3.457805	-0.553986
H	2.227889	2.169253	0.195799
C	4.543212	-0.514175	1.123600
N	5.539102	-0.321310	1.680881

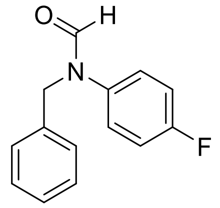

1-control (**Closed-Bonded**)

C	-2.327605	-0.644775	1.106806
C	-2.447062	0.839285	-1.263179
C	-3.366003	0.262039	0.916231
C	-1.347698	-0.810541	0.125233
C	-1.404355	-0.060121	-1.051248
C	-3.403780	0.983843	-0.269745
F	-4.408423	1.862053	-0.458970
N	-0.261670	-1.706440	0.351616
C	-0.034039	-2.745359	-0.507064
O	0.978875	-3.416815	-0.528756
C	0.823132	-1.251350	1.231247
C	1.585881	-0.111600	0.595653
C	2.892304	1.995330	-0.704741
C	2.514461	-0.376139	-0.417887
C	1.317615	1.212073	0.949957
C	1.968361	2.263816	0.303648
C	3.164579	0.673418	-1.063890
H	-2.275807	-1.236655	2.019698
H	-2.515487	1.433913	-2.171482
H	-4.144410	0.408236	1.661870
H	-0.612949	-0.170838	-1.791835
H	-0.892424	-2.933867	-1.178997
H	1.471328	-2.115518	1.402118
H	0.379963	-0.934540	2.182048
H	3.402183	2.814367	-1.210205
H	2.714538	-1.410632	-0.698760
H	0.586949	1.422522	1.732382
H	1.752130	3.293044	0.587244
H	3.889415	0.461252	-1.848993

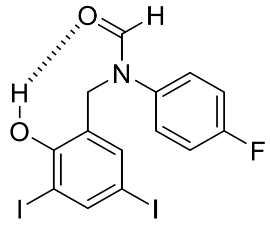

1-I$_2$ (**Closed-Bonded**)

C	-3.271160	0.989756	1.022921
C	-5.575664	0.736682	-0.547635
C	-4.340932	0.228131	1.490236
C	-3.353792	1.625895	-0.216631
C	-4.503257	1.499337	-0.999252
C	-5.468951	0.115606	0.690563
F	-6.502691	-0.625180	1.128498
N	-2.242535	2.377991	-0.711317
C	-1.895562	3.557585	-0.151514
O	-0.860274	4.162846	-0.406202
C	-1.336940	1.740583	-1.680417
C	-0.289182	0.860185	-1.035081
C	1.643886	-0.841963	0.069014
C	0.977422	1.378697	-0.695814
C	-0.582507	-0.485878	-0.798890
C	0.373412	-1.326349	-0.239965
C	1.936077	0.499461	-0.162996
O	1.321992	2.661222	-0.900182
H	-2.360099	1.082254	1.611978
H	-6.486220	0.622830	-1.131395
H	-4.305471	-0.282175	2.449925
H	-4.555633	2.008412	-1.960307
H	-2.644769	3.952361	0.555014
H	-1.961785	1.136552	-2.345613
H	-0.870697	2.537603	-2.268849
H	2.402342	-1.497295	0.489439
H	-1.564889	-0.872159	-1.068265
H	0.542018	3.258071	-0.837772
I	3.858109	1.229890	0.294117
I	-0.093233	-3.353887	0.123702

Table S5: Geometric parameters of closed-bonded conformations of series 1^{-}R calculated using M06-2X/aug-cc-pVDZ (aug-cc-pVDZ-PP for iodine atoms).

R	OH···O=CH distance, Å	O-H bond length, Å	O-H-O angle, °	C-C-O-H dihedral, °
Br	1.742	0.982	167.95	28.80
CF_3	1.739	0.983	167.71	27.86
CH_3	1.784	0.979	167.15	25.78
Cl	1.757	0.981	166.86	25.68
CN	1.736	0.983	166.70	24.42
F	1.767	0.980	167.88	26.62
H	1.773	0.979	167.02	28.24
NO_2	1.727	0.985	167.71	23.98
OMe	1.784	0.978	167.48	29.59
Ph	1.761	0.980	169.10	27.94
^{t}Bu	1.754	0.980	168.87	31.68
I_2	1.724	0.984	168.23	30.41
Average	1.754	0.981	167.72	27.58
St. Dev.	0.019	0.002	0.72	2.27

S4.2: DMC analysis of OH···R and HO···R interactions in compounds **1**-R.

The DMC approach involves creating two single mutations (species **B** and **C**) to a given molecule **A** and one double mutation **D**, where both mutations are present simultaneously (Figure S6). It posits that if the free energy change from a single **X**-mutation differs from that of the same mutation applied to a **Y**-mutant, it implies an interaction between residues **X** and **Y**. Hence, the difference in free energy between parallel mutations quantifies the direct or indirect **X**···**Y** interaction of interest. The DMC methodology isolates individual functional

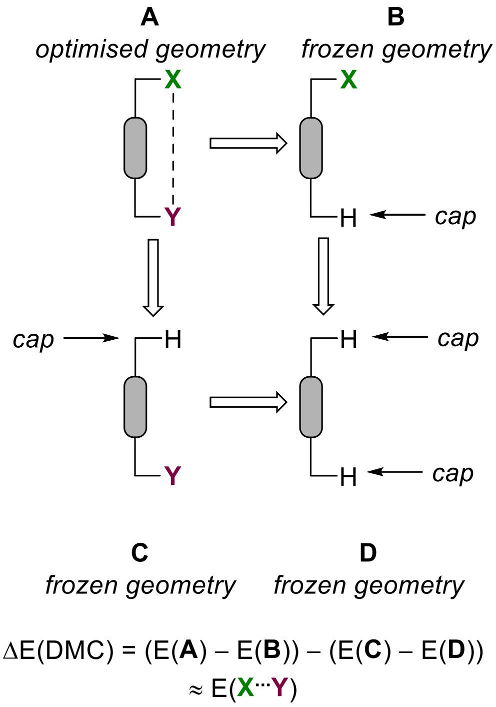

Figure S6: Double mutant cycle.

group interactions within a complex array, a process often simplified by the additive nature of secondary effects in synthetic systems. In accord with Figure S6, species **A** for each molecular balance **1**-R corresponded to its energy minimum from section S4.1. Then, the OH fragment in species **A** was removed and the dissected linker bond was capped with a hydrogen atom, which was placed at a bond distance 1.096 Å (which is the average length of a C−H bond) to give the species **B**. Next, a similar proton capping procedure was performed with the R-group to give the species **C**; and finally, both OH fragment and the R-group were replaced with the capping hydrogens to give the molecule **D**. Without re-optimisation of geometries of **B-D**, their single-point energies were computed and used to evaluate the quantities of $E_{OH\text{-}R}$ or $E_{HO\text{-}R}$, depending on the selection of the conformer species **A**. Calculations were run in Psi4 software using M06-2X/aug-cc-PVDZ (aug-cc-PVDZ-PP for iodine atoms) closed-bonded (CB) geometries of molecular balances **1**-R.

S4.2.1: DMC analysis of HO···R interactions in closed conformers of compounds **1**-R.

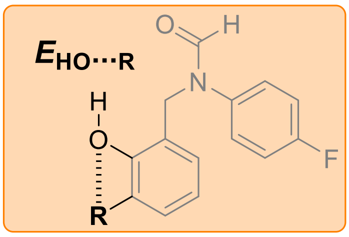

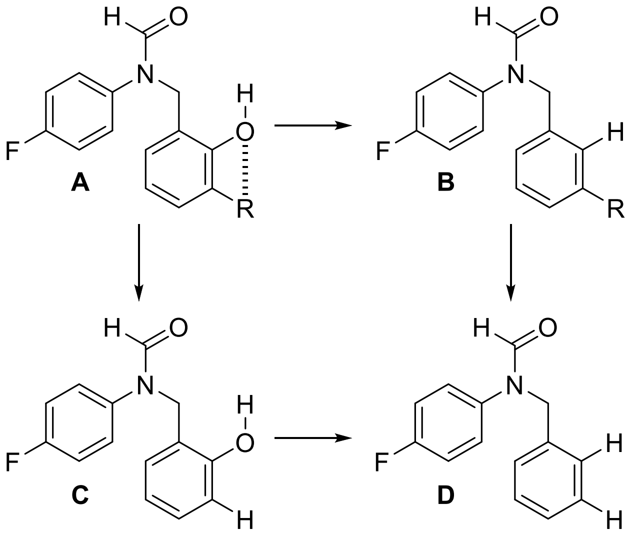

Table S6: Energies of species (kJ/mol) involved in the DMC cycle calculation of $E_{HO\ldots R}$

**1-R**	***E*(A)**	***E*(B)**	***E*(C)**	***E*(D)**	$E_{HO\ldots R}$
**1**-F	-2480549.46	-2283055.54	-2220044.08	-2022537.64	12.52
**1**-Br	-8976984.55	-8779483.5	-2220045.02	-2022539.17	4.80
**1**-CF_3	-3104830.32	-2907326.52	-2220045.58	-2022539.07	2.71
**1**-Me	-2323233.59	-2125723.27	-2220045.22	-2022538.32	-3.42
**1**-Cl	-3426704.77	-3229205.32	-2220045.14	-2022539.13	6.56
**1**-CN	-2462197.64	-2264690.55	-2220044.60	-2022538.56	-1.05
**1**-NO_2	-2756864.22	-2559370.72	-2220041.61	-2022537.46	10.65
**1**-OMe	-2520643.45	-2323151.05	-2220044.14	-2022537.00	14.74
**1**-Ph	-2826520.91	-2629019.23	-2220043.80	-2022538.67	3.45
**1**-^{t}Bu	-2632757.82	-2435256.34	-2220041.99	-2022537.18	3.33
**1**-I_2	-3769174.943	-3571682.054	-2994593.99	-2797108.837	-7.7
**1**-H			-2220045.74		
**1**-control				-2022544.59	

Geometries of Species B

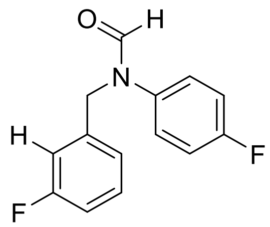

1-F (**B**)

C	-1.566932	0.170708	1.227278
C	-3.508415	-0.127260	-0.763387
C	-2.686879	-0.621076	1.474073
C	-1.426513	0.829335	0.005066
C	-2.395989	0.676532	-0.989380
C	-3.632783	-0.755997	0.469495
F	-4.712381	-1.527240	0.694149
N	-0.273179	1.631927	-0.242365
C	0.042138	2.653930	0.587556
O	1.119794	3.236540	0.588495
C	0.732048	1.139390	-1.198455
C	1.584314	0.027920	-0.634166
C	3.150777	-2.077809	0.351156
C	2.769889	0.318830	0.061021
C	1.190532	-1.304715	-0.808114
C	1.958441	-2.355234	-0.320825
C	3.532754	-0.761730	0.527343
H	-0.785194	0.266496	1.979341
H	-4.280613	-0.261566	-1.517324
H	-2.823124	-1.141095	2.419726
H	-2.281614	1.197910	-1.938829
H	-0.779955	2.940363	1.265056
H	0.190823	0.772765	-2.075946
H	1.340060	1.998696	-1.498944
H	3.790839	-2.866229	0.742783
H	0.265756	-1.512465	-1.347587
H	1.641986	-3.385563	-0.468220
F	4.680093	-0.489740	1.173151
H	3.163328	1.326377	0.237854

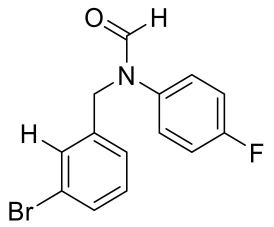

1-Br (**B**)

C	-2.563401	-0.720202	0.782236
C	-2.551943	0.798850	-1.569279
C	-3.648629	0.100270	0.489586
C	-1.475625	-0.782877	-0.091212
C	-1.465368	-0.016958	-1.257556
C	-3.619349	0.840865	-0.685268
F	-4.669834	1.630588	-0.975461
N	-0.352035	-1.602960	0.233586
C	0.004106	-2.632277	-0.567583
O	1.065924	-3.238455	-0.481758
C	0.592784	-1.119790	1.255679
C	1.526077	-0.052215	0.735087
C	3.240697	1.971825	-0.155575
C	2.770897	-0.399866	0.173532
C	1.153653	1.291762	0.821781
C	1.995883	2.304793	0.375484
C	3.617476	0.637947	-0.251386
H	-2.559947	-1.325210	1.687682
H	-2.573485	1.404557	-2.472233
H	-4.513198	0.164751	1.146267
H	-0.594796	-0.050901	-1.910844
H	-0.766543	-2.901596	-1.308783
H	1.145886	-1.988468	1.626040
H	-0.005835	-0.712687	2.076387
H	3.926220	2.743421	-0.499323
H	0.185801	1.541254	1.258851
H	1.696974	3.348133	0.450324
Br	5.311929	0.198539	-0.971565
H	3.125681	-1.433094	0.085305

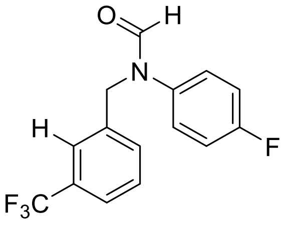

1-CF_3 (**B**)

C	-0.672759	3.070146	0.855907
C	0.790908	3.060612	-1.530301
C	0.154936	4.146966	0.554290
C	-0.768535	1.990417	-0.024851
C	-0.030185	1.980883	-1.208756
C	0.867410	4.119152	-0.637980
N	-1.594000	0.874887	0.311715
C	-1.119479	-0.060775	1.344920
C	-0.072333	-1.019902	0.832686
C	1.914270	-2.792705	-0.034903
C	-0.437765	-2.266587	0.290426
C	1.280381	-0.679287	0.909913
C	2.276553	-1.547895	0.476049
C	0.573470	-3.149649	-0.125796
C	0.172536	-4.488827	-0.681105
F	-0.562645	-4.376151	-1.801051
F	-0.547077	-5.211126	0.194678
F	1.252103	-5.233882	-1.002351
C	-2.637824	0.524027	-0.473004
O	-3.254952	-0.529858	-0.368090
F	1.664402	5.161266	-0.937149
H	-1.257447	3.066792	1.774724
H	1.375510	3.083172	-2.447166
H	0.246032	5.004331	1.217348
H	-0.089767	1.117016	-1.869087
H	-0.697231	0.542486	2.154461
H	-1.994207	-0.594529	1.729709
H	2.673629	-3.496163	-0.368547
H	1.552303	0.289639	1.331933
H	3.324798	-1.266612	0.547181
H	-2.907374	1.289853	-1.218881
H	-1.475663	-2.607695	0.203080

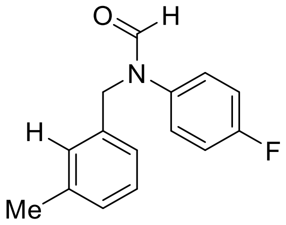

1-Me (**B**)

C	-1.098340	-0.695584	-2.937592
C	1.361911	0.628910	-3.110555
C	-0.829336	0.112649	-4.037646
C	-0.147849	-0.841764	-1.924558
C	1.072626	-0.169952	-2.005899
C	0.400804	0.755006	-4.102049
N	-0.444096	-1.645987	-0.783389
C	-1.386662	-1.114628	0.216983
C	-0.780721	-0.033923	1.079462
C	0.277226	2.001845	2.666347
C	-0.115435	-0.342161	2.277012
C	-0.885546	1.305100	0.684310
C	-0.357107	2.324968	1.464532
C	0.405972	0.682161	3.089947
C	1.083088	0.313506	4.380229
C	0.336412	-2.705348	-0.468577
O	0.294093	-3.303066	0.600105
F	0.668383	1.531035	-5.169234
H	-2.047177	-1.225381	-2.865466
H	2.306505	1.160566	-3.201322
H	-1.548032	0.241421	-4.843900
H	1.787546	-0.259898	-1.189275
H	-2.240698	-0.706505	-0.332787
H	-1.733600	-1.962595	0.815977
H	0.684825	2.795173	3.293766
H	-1.401468	1.540911	-0.247958
H	-0.446335	3.362965	1.149846
H	1.016032	-3.008298	-1.283196
H	0.394509	-0.232373	5.037298
H	1.933442	-0.354311	4.193666
H	1.437726	1.209985	4.899341
H	0.001304	-1.364428	2.654582

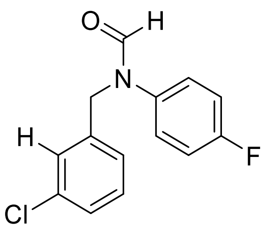

1-Cl (**B**)

C	-1.466257	0.039059	1.223985
C	-3.595681	-0.083793	-0.586523
C	-2.552495	-0.789610	1.497573
C	-1.449571	0.814870	0.063901
C	-2.511242	0.750029	-0.840767
C	-3.592955	-0.835358	0.582068
F	-4.641211	-1.640923	0.832739
N	-0.319852	1.640442	-0.223410
C	0.018177	2.657217	0.602801
O	1.086241	3.256083	0.559358
C	0.652954	1.161418	-1.220458
C	1.539139	0.057341	-0.694058
C	3.161521	-2.034073	0.213240
C	2.754246	0.357532	-0.048448
C	1.147897	-1.274846	-0.854236
C	1.943823	-2.321248	-0.400959
C	3.556900	-0.713635	0.381382
H	-0.614588	0.073920	1.902016
H	-4.439593	-0.151451	-1.269410
H	-2.592952	-1.403474	2.394394
H	-2.488492	1.362034	-1.741307
H	-0.775009	2.923208	1.321570
H	0.078725	0.790649	-2.075209
H	1.238652	2.027394	-1.544769
H	3.815877	-2.828816	0.565746
H	0.202332	-1.487446	-1.354874
H	1.629773	-3.354423	-0.533708
Cl	5.087028	-0.368365	1.138532
H	3.126614	1.376267	0.108831

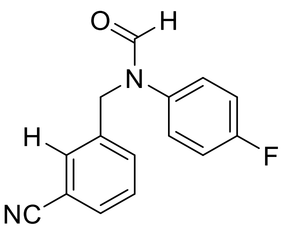

1-CN (**B**)

C	-1.794868	0.193944	1.189960
C	-3.711120	-0.146600	-0.818804
C	-2.918286	-0.592130	1.439383
C	-1.638625	0.825324	-0.044792
C	-2.595769	0.651575	-1.048176
C	-3.851546	-0.748448	0.426060
F	-4.934140	-1.513373	0.653488
N	-0.484130	1.626326	-0.294065
C	-0.167974	2.655074	0.526913
O	0.911453	3.235136	0.519828
C	0.517968	1.136509	-1.253273
C	1.388774	0.038813	-0.690083
C	2.998686	-2.054330	0.257197
C	2.581057	0.338937	-0.003654
C	1.020000	-1.296079	-0.865117
C	1.806944	-2.345673	-0.393725
C	3.385111	-0.724720	0.452298
H	-1.024865	0.308151	1.951416
H	-4.474539	-0.295878	-1.578799
H	-3.067450	-1.090786	2.394466
H	-2.470982	1.153843	-2.006521
H	-0.987934	2.949472	1.202832
H	-0.025562	0.756273	-2.123362
H	1.115018	1.998513	-1.567615
H	3.646335	-2.848173	0.624102
H	0.093322	-1.514644	-1.398591
H	1.500108	-3.377941	-0.546136
C	4.620191	-0.434310	1.131450
N	5.616081	-0.241445	1.688731
H	2.946742	1.356640	0.174589

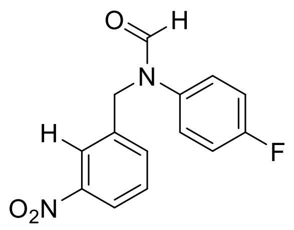

1-NO_2 (**B**)

C	-2.881711	-0.727164	0.794374
C	-2.820092	0.881462	-1.496636
C	-3.937168	0.141679	0.537197
C	-1.798159	-0.793161	-0.083879
C	-1.762727	0.017074	-1.219986
C	-3.883993	0.926534	-0.608435
F	-4.905153	1.763747	-0.863374
N	-0.700987	-1.661013	0.206553
C	-0.364204	-2.655808	-0.646470
O	0.687711	-3.282457	-0.591379
C	0.243605	-1.259479	1.261078
C	1.176605	-0.156884	0.819711
C	2.849813	1.943262	0.027391
C	2.410369	-0.464465	0.204138
C	0.811330	1.173692	1.021026
C	1.630338	2.230206	0.625417
C	3.232223	0.619692	-0.154547
H	-2.899460	-1.367627	1.674980
H	-2.821706	1.520978	-2.376299
H	-4.798431	0.209684	1.197938
H	-0.896056	-0.020300	-1.878640
H	-1.139497	-2.874011	-1.399379
H	0.793727	-2.155338	1.565726
H	-0.350932	-0.911864	2.111495
H	3.526744	2.727375	-0.303575
H	-0.144779	1.382574	1.503531
H	1.326055	3.260994	0.789854
N	4.553335	0.387118	-0.754663
O	5.218645	-0.529529	-0.326450
O	4.903719	1.161050	-1.630265
H	2.740837	-1.488020	-0.006435

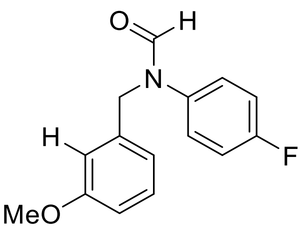

1-OMe (**B**)

C	-2.199946	0.065485	1.240361
C	-4.317232	-0.139148	-0.575808
C	-3.245477	-0.818104	1.501410
C	-2.218839	0.857671	0.091202
C	-3.276725	0.752161	-0.815082
C	-4.280338	-0.904840	0.583143
F	-5.287548	-1.766813	0.819222
N	-1.139020	1.748666	-0.182063
C	-0.798932	2.708843	0.708975
O	0.233762	3.364959	0.659003
C	-0.203440	1.405501	-1.266753
C	0.787452	0.337466	-0.870411
C	2.612117	-1.677548	-0.208564
C	2.035628	0.682844	-0.343506
C	0.448970	-1.014984	-1.041144
C	1.347924	-2.013531	-0.708756
C	2.961423	-0.342370	-0.029247
H	-1.350926	0.128677	1.919519
H	-5.154727	-0.239846	-1.262676
H	-3.255976	-1.445078	2.389964
H	-3.285842	1.377577	-1.706477
H	-1.559835	2.872207	1.491692
H	-0.806028	1.049893	-2.108270
H	0.300602	2.329834	-1.565900
H	3.315617	-2.468055	0.038303
H	-0.529981	-1.269725	-1.449183
H	1.084630	-3.060771	-0.844612
O	4.157930	0.087400	0.444345
C	5.118173	-0.900110	0.754760
H	4.760708	-1.562669	1.556675
H	5.370902	-1.499998	-0.131755
H	6.004180	-0.359560	1.094919
H	2.370793	1.713965	-0.183286

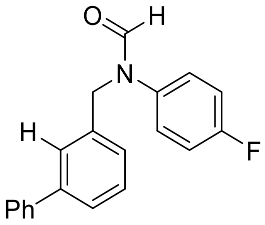

1-Ph (**B**)

C	-4.277506	-0.736090	0.703521
C	-4.124331	0.972553	-1.507818
C	-5.314489	0.155788	0.452075
C	-3.163516	-0.774469	-0.138728
C	-3.083110	0.087569	-1.233375
C	-5.217026	0.989851	-0.654999
F	-6.222375	1.849479	-0.904968
N	-2.091172	-1.671034	0.145507
C	-1.676915	-2.562959	-0.783640
O	-0.636963	-3.206487	-0.708369
C	-1.245374	-1.403783	1.322339
C	-0.266023	-0.275809	1.101147
C	1.510696	1.839769	0.760296
C	1.027561	-0.522042	0.603860
C	-0.653122	1.032815	1.402757
C	0.225530	2.096066	1.231250
C	1.935546	0.546788	0.439740
H	-4.333504	-1.415266	1.553031
H	-4.088717	1.652059	-2.356412
H	-6.195646	0.204363	1.087804
H	-2.191880	0.076363	-1.859040
H	-2.381177	-2.682798	-1.624117
H	-0.734603	-2.338990	1.572460
H	-1.916799	-1.144069	2.146816
H	2.220408	2.659665	0.651940
H	-1.657737	1.209872	1.790729
H	-0.077914	3.110959	1.480340
C	3.312681	0.336317	-0.079301
C	5.940751	0.042070	-1.033774
C	3.832940	1.218985	-1.035569
C	4.129220	-0.702516	0.386899
C	5.432371	-0.843950	-0.083310
C	5.134587	1.073969	-1.510665
H	3.201552	2.017149	-1.426022
H	3.739316	-1.399925	1.124608
H	6.056113	-1.653380	0.294573
H	5.517539	1.766062	-2.259707
H	6.958701	-0.074267	-1.403589
H	1.374386	-1.530679	0.351711

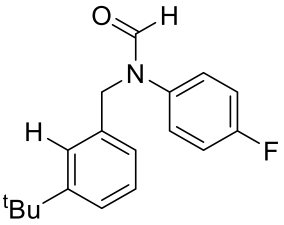

1-^{t}Bu (**B**)

C	-4.219551	-0.672817	1.040560
C	-4.219976	0.855188	-1.304725
C	-5.284581	0.182943	0.779115
C	-3.155374	-0.764872	0.140708
C	-3.151663	0.005975	-1.022814
C	-5.262368	0.928071	-0.393400
N	-2.048619	-1.616185	0.437042
C	-1.096535	-1.188328	1.479266
C	-0.134837	-0.127244	0.999148
C	1.625974	1.862333	0.199158
C	1.127736	-0.468550	0.480993
C	-0.507791	1.216757	1.088785
C	0.364411	2.214491	0.681740
C	2.046388	0.534730	0.087424
C	3.442327	0.172801	-0.435552
C	-1.705023	-2.612291	-0.409727
O	-0.653345	-3.239000	-0.350158
F	-6.292750	1.755033	-0.651777
H	-4.213289	-1.280948	1.943867
H	-4.243865	1.465797	-2.204355
H	-6.128734	0.272132	1.459290
H	-2.297405	-0.047297	-1.696166
H	-1.692924	-0.791179	2.306542
H	-0.572313	-2.082922	1.828912
H	2.299682	2.661527	-0.097622
H	-1.488773	1.471311	1.493585
H	0.079077	3.262377	0.751338
H	-2.477475	-2.835442	-1.164835
C	3.326219	-0.666205	-1.720303
H	2.799967	-1.606597	-1.539867
H	4.331528	-0.895269	-2.098666
H	2.790762	-0.102168	-2.495145
C	4.220742	-0.606119	0.639724
H	3.729542	-1.550339	0.887045
H	4.311007	-0.005137	1.553878
H	5.232484	-0.824004	0.272304
C	4.255415	1.427844	-0.775436
H	3.776754	2.022392	-1.564144
H	5.243727	1.121923	-1.139659
H	4.404269	2.066185	0.104964
H	1.439182	-1.516896	0.408963

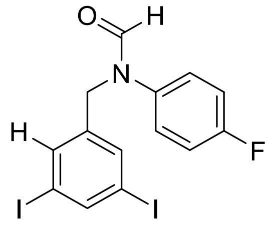

1-I_2 (**B**)

C	-3.271160	0.989756	1.022921
C	-5.575664	0.736682	-0.547635
C	-4.340932	0.228131	1.490236
C	-3.353792	1.625895	-0.216631
C	-4.503257	1.499337	-0.999252
C	-5.468951	0.115606	0.690563
F	-6.502691	-0.625180	1.128498
N	-2.242535	2.377991	-0.711317
C	-1.895562	3.557585	-0.151514
O	-0.860274	4.162846	-0.406202
C	-1.336940	1.740583	-1.680417
C	-0.289182	0.860185	-1.035081
C	1.643886	-0.841963	0.069014
C	0.977422	1.378697	-0.695814
C	-0.582507	-0.485878	-0.798890
C	0.373412	-1.326349	-0.239965
C	1.936077	0.499461	-0.162996
H	-2.360099	1.082254	1.611978
H	-6.486220	0.622830	-1.131395
H	-4.305471	-0.282175	2.449925
H	-4.555633	2.008412	-1.960307
H	-2.644769	3.952361	0.555014
H	-1.961785	1.136552	-2.345613
H	-0.870697	2.537603	-2.268849
H	2.402342	-1.497295	0.489439
H	-1.564889	-0.872159	-1.068265
I	3.858109	1.229890	0.294117
I	-0.093233	-3.353887	0.123702
H	1.258486	2.424847	-0.862516

Geometries of Species C

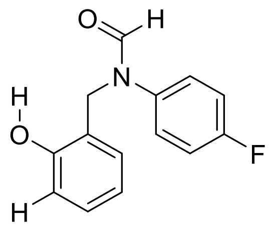

1-F (**C**)

C	-1.647464	0.090452	1.220375
C	-3.588947	-0.207515	-0.770290
C	-2.767411	-0.701331	1.467170
C	-1.507045	0.749079	-0.001837
C	-2.476521	0.596277	-0.996283
C	-3.713315	-0.836253	0.462592
F	-4.792913	-1.607495	0.687246
N	-0.353711	1.551671	-0.249268
C	-0.038394	2.573674	0.580653
O	1.039262	3.156285	0.581592
C	0.651516	1.059134	-1.205359
C	1.503782	-0.052335	-0.641069
C	3.070245	-2.158064	0.344253
C	2.689357	0.238574	0.054118
C	1.110000	-1.384971	-0.815017
C	1.877909	-2.435490	-0.327728
C	3.452222	-0.841986	0.520440
O	3.172950	1.476995	0.271471
H	-0.865726	0.186240	1.972438
H	-4.361145	-0.341821	-1.524227
H	-2.903656	-1.221351	2.412823
H	-2.362146	1.117655	-1.945732
H	-0.860487	2.860108	1.258153
H	0.110291	0.692510	-2.082849
H	1.259528	1.918441	-1.505847
H	3.710307	-2.946484	0.735880
H	0.185224	-1.592720	-1.354490
H	1.561454	-3.465818	-0.475123
H	2.457271	2.146789	0.278995
H	4.387566	-0.620252	1.046921

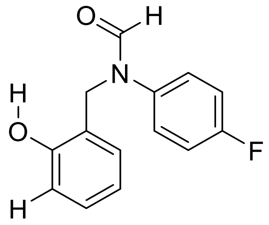

1-Br (**C**)

C	-2.624306	-0.641874	0.773500
C	-2.612848	0.877178	-1.578016
C	-3.709534	0.178598	0.480850
C	-1.536530	-0.704549	-0.099949
C	-1.526273	0.061370	-1.266292
C	-3.680254	0.919193	-0.694005
F	-4.730739	1.708916	-0.984197
N	-0.412940	-1.524632	0.224849
C	-0.056798	-2.553949	-0.576319
O	1.005019	-3.160126	-0.490494
C	0.531880	-1.041462	1.246942
C	1.465172	0.026113	0.726351
C	3.179792	2.050153	-0.164311
C	2.709992	-0.321538	0.164796
C	1.092748	1.370091	0.813044
C	1.934978	2.383121	0.366748
C	3.556571	0.716275	-0.260123
H	-2.620852	-1.246882	1.678945
H	-2.634390	1.482885	-2.480969
H	-4.574103	0.243079	1.137531
H	-0.655701	0.027427	-1.919580
H	-0.827448	-2.823268	-1.317520
H	1.084981	-1.910140	1.617303
H	-0.066740	-0.634359	2.067651
H	3.865315	2.821749	-0.508059
H	0.124897	1.619583	1.250114
H	1.636069	3.426461	0.441587
O	3.145247	-1.589120	0.056557
H	2.399102	-2.222143	-0.029814
H	4.537692	0.461849	-0.677120

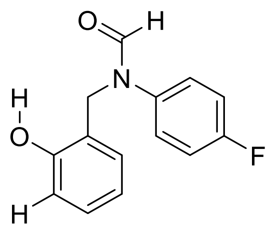

1-CF_3 (**C**)

C	-0.585890	2.630985	0.761364
C	0.877777	2.621450	-1.624845
C	0.241804	3.707805	0.459746
C	-0.681667	1.551255	-0.119395
C	0.056683	1.541721	-1.303300
C	0.954279	3.679990	-0.732524
N	-1.507132	0.435725	0.217171
C	-1.032611	-0.499937	1.250376
C	0.014535	-1.459065	0.738143
C	2.001139	-3.231867	-0.129446
C	-0.350897	-2.705749	0.195883
C	1.367249	-1.118450	0.815370
C	2.363422	-1.987058	0.381506
C	0.660338	-3.588812	-0.220340
O	-1.623849	-3.124109	0.088755
C	-2.550956	0.084865	-0.567548
O	-3.168084	-0.969020	-0.462633
F	1.751271	4.722104	-1.031693
H	-1.170579	2.627630	1.680180
H	1.462378	2.644011	-2.541710
H	0.332900	4.565169	1.122804
H	-0.002899	0.677854	-1.963630
H	-0.610363	0.103324	2.059917
H	-1.907339	-1.033691	1.635166
H	2.760497	-3.935326	-0.463091
H	1.639171	-0.149523	1.237390
H	3.411667	-1.705775	0.452637
H	-2.250539	-2.371600	0.002133
H	-2.820506	0.850691	-1.313425
H	0.368201	-4.564595	-0.624962

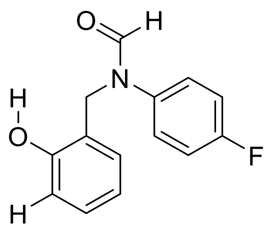

1-Me (**C**)

C	-0.967698	-0.594393	-2.525404
C	1.492553	0.730100	-2.698368
C	-0.698694	0.213840	-3.625459
C	-0.017207	-0.740574	-1.512370
C	1.203268	-0.068761	-1.593712
C	0.531446	0.856197	-3.689862
N	-0.313454	-1.544796	-0.371202
C	-1.256020	-1.013437	0.629170
C	-0.650079	0.067268	1.491649
C	0.407868	2.103036	3.078534
C	0.015207	-0.240971	2.689199
C	-0.754904	1.406291	1.096497
C	-0.226465	2.426159	1.876719
C	0.536614	0.783352	3.502135
O	0.159661	-1.505930	3.156407
C	0.467054	-2.604158	-0.056390
O	0.424735	-3.201875	1.012293
F	0.799025	1.632225	-4.757047
H	-1.916535	-1.124191	-2.453278
H	2.437147	1.261756	-2.789135
H	-1.417390	0.342611	-4.431713
H	1.918188	-0.158707	-0.777088
H	-2.110056	-0.605315	0.079400
H	-1.602958	-1.861405	1.228165
H	0.815467	2.896363	3.705953
H	-1.270826	1.642102	0.164230
H	-0.315693	3.464156	1.562033
H	0.132722	-2.168371	2.436674
H	1.146674	-2.907108	-0.871008
H	1.030350	0.514538	4.442975

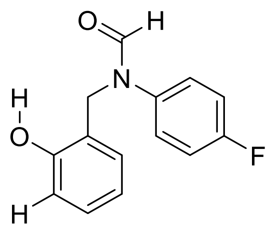

1-Cl (**C**)

C	-1.532931	-0.039865	1.226492
C	-3.662356	-0.162717	-0.584016
C	-2.619169	-0.868535	1.500079
C	-1.516246	0.735946	0.066407
C	-2.577916	0.671104	-0.838261
C	-3.659629	-0.914282	0.584574
F	-4.707885	-1.719847	0.835246
N	-0.386526	1.561517	-0.220904
C	-0.048497	2.578292	0.605307
O	1.019567	3.177158	0.561865
C	0.586280	1.082493	-1.217952
C	1.472465	-0.021584	-0.691552
C	3.094847	-2.112998	0.215746
C	2.687572	0.278607	-0.045941
C	1.081223	-1.353771	-0.851730
C	1.877149	-2.400173	-0.398453
C	3.490226	-0.792559	0.383888
O	3.144406	1.528427	0.147013
H	-0.681262	-0.005004	1.904523
H	-4.506268	-0.230376	-1.266904
H	-2.659626	-1.482398	2.396900
H	-2.555166	1.283109	-1.738801
H	-0.841684	2.844284	1.324076
H	0.012051	0.711725	-2.072703
H	1.171978	1.948470	-1.542263
H	3.749203	-2.907741	0.568252
H	0.135658	-1.566371	-1.352368
H	1.563099	-3.433348	-0.531202
H	2.416387	2.185734	0.172360
H	4.453048	-0.575301	0.860320

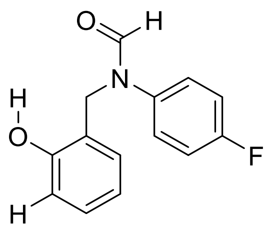

1-CN (**C**)

C	-1.677394	0.105677	1.243537
C	-3.593646	-0.234867	-0.765227
C	-2.800812	-0.680397	1.492959
C	-1.521151	0.737057	0.008784
C	-2.478295	0.563308	-0.994599
C	-3.734072	-0.836714	0.479637
F	-4.816666	-1.601640	0.707065
N	-0.366656	1.538059	-0.240489
C	-0.050500	2.566807	0.580489
O	1.028927	3.146869	0.573404
C	0.635442	1.048243	-1.199696
C	1.506248	-0.049454	-0.636506
C	3.116160	-2.142597	0.310773
C	2.698531	0.250670	0.049923
C	1.137474	-1.384346	-0.811540
C	1.924418	-2.433940	-0.340148
C	3.502585	-0.812987	0.505874
O	3.145668	1.495056	0.267867
H	-0.907391	0.219884	2.004993
H	-4.357065	-0.384145	-1.525223
H	-2.949976	-1.179053	2.448043
H	-2.353508	1.065576	-1.952944
H	-0.870460	2.861205	1.256409
H	0.091912	0.668006	-2.069785
H	1.232492	1.910246	-1.514039
H	3.763809	-2.936440	0.677679
H	0.210796	-1.602911	-1.345015
H	1.617583	-3.466208	-0.492559
H	2.422341	2.160851	0.257225
H	4.443206	-0.591815	1.023108

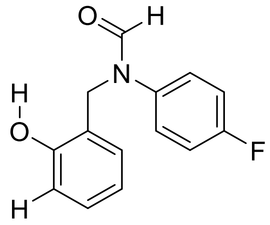

1-NO_2 (**C**)

C	-2.604106	-0.622621	0.729862
C	-2.542488	0.986004	-1.561149
C	-3.659563	0.246222	0.472684
C	-1.520554	-0.688619	-0.148392
C	-1.485122	0.121617	-1.284498
C	-3.606388	1.031077	-0.672948
F	-4.627549	1.868290	-0.927887
N	-0.423383	-1.556470	0.142040
C	-0.086600	-2.551265	-0.710983
O	0.965316	-3.177914	-0.655892
C	0.521210	-1.154937	1.196565
C	1.454210	-0.052342	0.755199
C	3.127418	2.047805	-0.037122
C	2.687974	-0.359922	0.139625
C	1.088935	1.278235	0.956513
C	1.907942	2.334749	0.560904
C	3.509827	0.724235	-0.219060
H	-2.621856	-1.263084	1.610467
H	-2.544102	1.625520	-2.440812
H	-4.520826	0.314227	1.133425
H	-0.618451	0.084243	-1.943153
H	-0.861893	-2.769468	-1.463891
H	1.071332	-2.050795	1.501213
H	-0.073327	-0.807321	2.046982
H	3.804349	2.831918	-0.368088
H	0.132826	1.487117	1.439019
H	1.603660	3.365537	0.725341
O	3.090948	-1.608050	-0.117148
H	2.335136	-2.234767	-0.192186
H	4.495124	0.550779	-0.666632

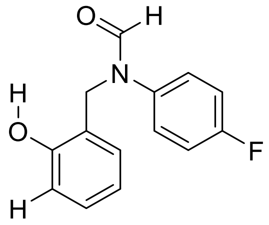

1-OMe (**C**)

C	-1.542775	-0.168649	1.350015
C	-3.660060	-0.373282	-0.466154
C	-2.588306	-1.052238	1.611064
C	-1.561667	0.623537	0.200856
C	-2.619554	0.518027	-0.705428
C	-3.623167	-1.138975	0.692797
F	-4.630377	-2.000948	0.928876
N	-0.481849	1.514532	-0.072409
C	-0.141761	2.474709	0.818629
O	0.890934	3.130825	0.768657
C	0.453731	1.171366	-1.157099
C	1.444623	0.103332	-0.760757
C	3.269289	-1.911682	-0.098911
C	2.692799	0.448710	-0.233852
C	1.106141	-1.249118	-0.931490
C	2.005095	-2.247665	-0.599102
C	3.618594	-0.576504	0.080407
O	3.105992	1.719879	-0.036333
H	-0.693754	-0.105457	2.029172
H	-4.497556	-0.473981	-1.153022
H	-2.598805	-1.679212	2.499618
H	-2.628671	1.143442	-1.596824
H	-0.902664	2.638072	1.601346
H	-0.148857	0.815759	-1.998616
H	0.957773	2.095700	-1.456246
H	3.972789	-2.702189	0.147957
H	0.127190	-1.503859	-1.339529
H	1.741802	-3.294905	-0.734958
H	2.347882	2.310088	0.148342
H	4.585187	-0.229316	0.462996

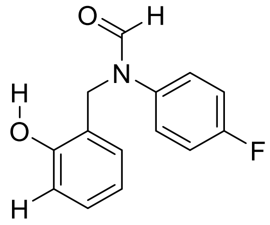

1-Ph (**C**)

C	-2.627286	-0.603386	0.468430
C	-2.474111	1.105257	-1.742910
C	-3.664269	0.288492	0.216984
C	-1.513296	-0.641765	-0.373820
C	-1.432890	0.220273	-1.468467
C	-3.566806	1.122554	-0.890090
F	-4.572156	1.982183	-1.140059
N	-0.440952	-1.538330	-0.089584
C	-0.026695	-2.430255	-1.018731
O	1.013257	-3.073784	-0.943460
C	0.404847	-1.271079	1.087247
C	1.384197	-0.143105	0.866056
C	3.160917	1.972473	0.525205
C	2.677782	-0.389338	0.368769
C	0.997099	1.165519	1.167665
C	1.875751	2.228770	0.996159
C	3.585766	0.679492	0.204649
H	-2.683284	-1.282562	1.317940
H	-2.438497	1.784763	-2.591503
H	-4.545426	0.337067	0.852712
H	-0.541660	0.209067	-2.094131
H	-0.730956	-2.550095	-1.859209
H	0.915618	-2.206286	1.337368
H	-0.266579	-1.011366	1.911725
H	3.870628	2.792369	0.416849
H	-0.007517	1.342575	1.555638
H	1.572306	3.243663	1.245249
O	3.106193	-1.635246	0.057304
H	2.367006	-2.222249	-0.205990
H	4.601012	0.524330	-0.177996

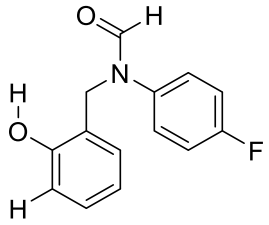

1-^{t}Bu (**C**)

C	-2.619590	-0.576528	0.765969
C	-2.620016	0.951478	-1.579316
C	-3.684621	0.279232	0.504525
C	-1.555414	-0.668583	-0.133883
C	-1.551703	0.102265	-1.297405
C	-3.662408	1.024361	-0.667991
N	-0.448658	-1.519896	0.162451
C	0.503425	-1.092039	1.204676
C	1.465123	-0.030954	0.724557
C	3.225934	1.958623	-0.075433
C	2.727696	-0.372261	0.206402
C	1.092169	1.313047	0.814194
C	1.964371	2.310780	0.407149
C	3.646349	0.631020	-0.187167
O	3.113975	-1.672496	0.117066
C	-0.105063	-2.516002	-0.684318
O	0.946615	-3.142711	-0.624749
F	-4.692790	1.851323	-0.926368
H	-2.613329	-1.184658	1.669277
H	-2.643905	1.562086	-2.478946
H	-4.528774	0.368422	1.184699
H	-0.697445	0.048993	-1.970757
H	-0.092964	-0.694890	2.031951
H	1.027647	-1.986633	1.554321
H	3.899642	2.757817	-0.372213
H	0.111187	1.567601	1.218995
H	1.679037	3.358667	0.476748
H	2.347314	-2.261342	-0.044188
H	-0.877515	-2.739152	-1.439426
H	4.643711	0.372431	-0.560819

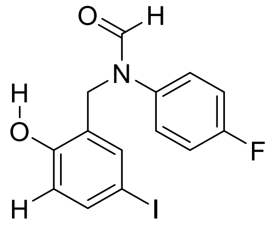

1-I_2 (**C**)

C	-3.271160	0.989756	1.022921
C	-5.575664	0.736682	-0.547635
C	-4.340932	0.228131	1.490236
C	-3.353792	1.625895	-0.216631
C	-4.503257	1.499337	-0.999252
C	-5.468951	0.115606	0.690563
F	-6.502691	-0.625180	1.128498
N	-2.242535	2.377991	-0.711317
C	-1.895562	3.557585	-0.151514
O	-0.860274	4.162846	-0.406202
C	-1.336940	1.740583	-1.680417
C	-0.289182	0.860185	-1.035081
C	1.643886	-0.841963	0.069014
C	0.977422	1.378697	-0.695814
C	-0.582507	-0.485878	-0.798890
C	0.373412	-1.326349	-0.239965
C	1.936077	0.499461	-0.162996
O	1.321992	2.661222	-0.900182
H	-2.360099	1.082254	1.611978
H	-6.486220	0.622830	-1.131395
H	-4.305471	-0.282175	2.449925
H	-4.555633	2.008412	-1.960307
H	-2.644769	3.952361	0.555014
H	-1.961785	1.136552	-2.345613
H	-0.870697	2.537603	-2.268849
H	2.402342	-1.497295	0.489439
H	-1.564889	-0.872159	-1.068265
H	0.542018	3.258071	-0.837772
I	-0.093233	-3.353887	0.123702
H	2.936173	0.879527	0.074855

Geometries of Species D

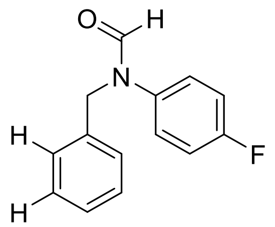

1-F (**D**)

C	-1.559622	0.172441	1.231392
C	-3.501105	-0.125527	-0.759272
C	-2.679569	-0.619343	1.478188
C	-1.419203	0.831068	0.009181
C	-2.388680	0.678265	-0.985265
C	-3.625473	-0.754265	0.473610
F	-4.705071	-1.525507	0.698264
N	-0.265869	1.633660	-0.238250
C	0.049449	2.655663	0.591671
O	1.127104	3.238274	0.592609
C	0.739358	1.141123	-1.194341
C	1.591625	0.029653	-0.630052
C	3.158087	-2.076076	0.355271
C	2.777200	0.320563	0.065135
C	1.197843	-1.302982	-0.804000
C	1.965751	-2.353502	-0.316711
C	3.540064	-0.759997	0.531458
H	-0.777884	0.268229	1.983456
H	-4.273304	-0.259833	-1.513210
H	-2.815815	-1.139362	2.423841
H	-2.274304	1.199643	-1.934715
H	-0.772645	2.942097	1.269171
H	0.198133	0.774498	-2.071832
H	1.347371	2.000430	-1.494830
H	3.798150	-2.864496	0.746898
H	0.273066	-1.510732	-1.343472
H	1.649296	-3.383831	-0.464105
H	3.170639	1.328111	0.241969
H	4.475408	-0.538263	1.057939

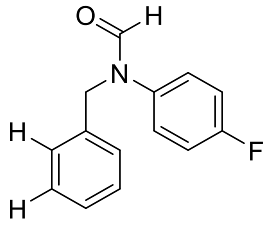

1-Br (**D**)

C	-2.538803	-0.726581	0.771782
C	-2.527345	0.792471	-1.579734
C	-3.624031	0.093891	0.479132
C	-1.451027	-0.789256	-0.101667
C	-1.440771	-0.023337	-1.268010
C	-3.594751	0.834487	-0.695723
F	-4.645236	1.624209	-0.985915
N	-0.327438	-1.609338	0.223131
C	0.028704	-2.638656	-0.578037
O	1.090522	-3.244833	-0.492212
C	0.617382	-1.126169	1.245224
C	1.550675	-0.058594	0.724633
C	3.265295	1.965446	-0.166029
C	2.795495	-0.406245	0.163077
C	1.178250	1.285384	0.811326
C	2.020480	2.298414	0.365030
C	3.642074	0.631568	-0.261841
H	-2.535350	-1.331589	1.677227
H	-2.548887	1.398178	-2.482687
H	-4.488601	0.158372	1.135813
H	-0.570199	-0.057280	-1.921299
H	-0.741946	-2.907975	-1.319238
H	1.170483	-1.994847	1.615585
H	0.018763	-0.719066	2.065933
H	3.950818	2.737042	-0.509777
H	0.210399	1.534876	1.248396
H	1.721571	3.341754	0.439869
H	3.150279	-1.439473	0.074850
H	4.623194	0.377143	-0.678838

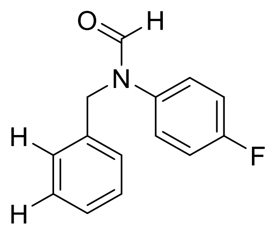

1-CF_3 (**D**)

C	-0.671601	2.546541	0.760755
C	0.792067	2.537006	-1.625454
C	0.156094	3.623361	0.459138
C	-0.767377	1.466812	-0.120003
C	-0.029027	1.457278	-1.303908
C	0.868568	3.595547	-0.733133
N	-1.592842	0.351282	0.216563
C	-1.118321	-0.584381	1.249768
C	-0.071175	-1.543508	0.737534
C	1.915429	-3.316310	-0.130055
C	-0.436607	-2.790192	0.195274
C	1.281539	-1.202893	0.814761
C	2.277712	-2.071501	0.380897
C	0.574628	-3.673255	-0.220948
C	-2.636666	0.000422	-0.568156
O	-3.253794	-1.053463	-0.463242
F	1.665560	4.637661	-1.032302
H	-1.256289	2.543186	1.679572
H	1.376668	2.559567	-2.542319
H	0.247190	4.480726	1.122196
H	-0.088609	0.593411	-1.964239
H	-0.696073	0.018881	2.059309
H	-1.993049	-1.118134	1.634557
H	2.674787	-4.019769	-0.463699
H	1.553461	-0.233967	1.236781
H	3.325957	-1.790218	0.452029
H	-2.906216	0.766248	-1.314033
H	0.282490	-4.649038	-0.625571
H	-1.474505	-3.131301	0.107928

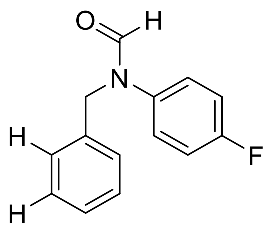

1-Me (**D**)

C	-0.962166	-0.677533	-2.438290
C	1.498085	0.646960	-2.611253
C	-0.693161	0.130699	-3.538345
C	-0.011675	-0.823714	-1.425256
C	1.208800	-0.151902	-1.506598
C	0.536978	0.773057	-3.602747
N	-0.307922	-1.627937	-0.284088
C	-1.250488	-1.096578	0.716285
C	-0.644546	-0.015873	1.578763
C	0.413400	2.019896	3.165649
C	0.020739	-0.324111	2.776314
C	-0.749372	1.323150	1.183612
C	-0.220933	2.343019	1.963833
C	0.542146	0.700211	3.589249
C	0.472587	-2.687298	0.030725
O	0.430267	-3.285015	1.099407
F	0.804557	1.549085	-4.669932
H	-1.911003	-1.207331	-2.366164
H	2.442679	1.178616	-2.702020
H	-1.411857	0.259471	-4.344599
H	1.923720	-0.241847	-0.689973
H	-2.104524	-0.688455	0.166514
H	-1.597426	-1.944545	1.315279
H	0.820999	2.813223	3.793067
H	-1.265294	1.558962	0.251344
H	-0.310161	3.381015	1.649147
H	1.152207	-2.990248	-0.783894
H	1.035882	0.431398	4.530089
H	0.137478	-1.346377	3.153884

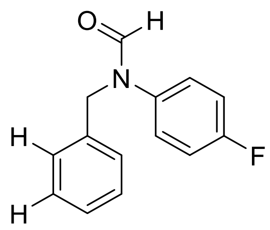

1-Cl (**D**)

C	-1.446695	0.043474	1.233665
C	-3.576119	-0.079379	-0.576843
C	-2.532933	-0.785196	1.507253
C	-1.430009	0.819285	0.073581
C	-2.491680	0.754443	-0.831087
C	-3.573393	-0.830944	0.591748
F	-4.621649	-1.636509	0.842419
N	-0.300289	1.644856	-0.213730
C	0.037740	2.661631	0.612481
O	1.105803	3.260497	0.569038
C	0.672516	1.165832	-1.210778
C	1.558701	0.061755	-0.684378
C	3.181084	-2.029659	0.222920
C	2.773808	0.361946	-0.038768
C	1.167460	-1.270432	-0.844556
C	1.963386	-2.316834	-0.391279
C	3.576462	-0.709221	0.391062
H	-0.595026	0.078334	1.911696
H	-4.420031	-0.147037	-1.259730
H	-2.573390	-1.399060	2.404074
H	-2.468930	1.366448	-1.731627
H	-0.755447	2.927622	1.331249
H	0.098287	0.795063	-2.065529
H	1.258214	2.031809	-1.535089
H	3.835440	-2.824402	0.575426
H	0.221894	-1.483032	-1.345194
H	1.649335	-3.350009	-0.524028
H	3.146176	1.380681	0.118511
H	4.539285	-0.491962	0.867493

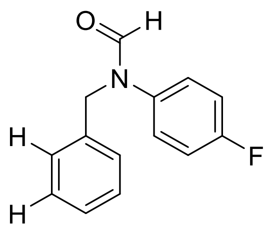

1-CN (**D**)

C	-1.591057	0.188006	1.253776
C	-3.507308	-0.152538	-0.754989
C	-2.714475	-0.598068	1.503198
C	-1.434813	0.819386	0.019023
C	-2.391958	0.645636	-0.984360
C	-3.647734	-0.754386	0.489875
F	-4.730328	-1.519311	0.717304
N	-0.280318	1.620388	-0.230250
C	0.035838	2.649136	0.590728
O	1.115265	3.229198	0.583643
C	0.721780	1.130571	-1.189457
C	1.592586	0.032875	-0.626267
C	3.202498	-2.060268	0.321012
C	2.784869	0.332998	0.060162
C	1.223812	-1.302017	-0.801301
C	2.010756	-2.351611	-0.329909
C	3.588923	-0.730659	0.516113
H	-0.821053	0.302213	2.015231
H	-4.270728	-0.301817	-1.514984
H	-2.863639	-1.096724	2.458282
H	-2.267170	1.147905	-1.942705
H	-0.784122	2.943534	1.266648
H	0.178250	0.750334	-2.059546
H	1.318830	1.992574	-1.503800
H	3.850147	-2.854111	0.687918
H	0.297133	-1.520583	-1.334776
H	1.703920	-3.383879	-0.482321
H	3.150553	1.350702	0.238404
H	4.529544	-0.509486	1.033347

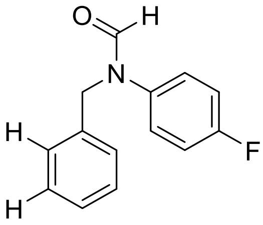

1-NO_2 (**D**)

C	-2.521084	-0.707426	0.721641
C	-2.459466	0.901200	-1.569369
C	-3.576541	0.161417	0.464464
C	-1.437532	-0.773423	-0.156612
C	-1.402100	0.036812	-1.292719
C	-3.523366	0.946272	-0.681168
F	-4.544527	1.783485	-0.936107
N	-0.340360	-1.641275	0.133820
C	-0.003577	-2.636070	-0.719203
O	1.048338	-3.262719	-0.664112
C	0.604232	-1.239741	1.188345
C	1.537232	-0.137146	0.746978
C	3.210440	1.963000	-0.045343
C	2.770996	-0.444727	0.131405
C	1.171957	1.193430	0.948293
C	1.990965	2.249944	0.552684
C	3.592849	0.639430	-0.227281
H	-2.538833	-1.347889	1.602246
H	-2.461079	1.540716	-2.449032
H	-4.437804	0.229422	1.125205
H	-0.535429	-0.000562	-1.951373
H	-0.778870	-2.854273	-1.472112
H	1.154354	-2.135600	1.492993
H	0.009695	-0.892126	2.038762
H	3.887371	2.747113	-0.376308
H	0.215848	1.402312	1.430798
H	1.686682	3.280732	0.717121
H	4.578146	0.465974	-0.674852
H	3.101464	-1.468282	-0.079168

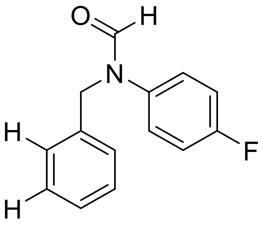

1-OMe (**D**)

C	-1.459122	-0.080713	1.356417
C	-3.576408	-0.285347	-0.459753
C	-2.504654	-0.964302	1.617465
C	-1.478015	0.711473	0.207257
C	-2.535902	0.605963	-0.699027
C	-3.539515	-1.051039	0.699198
F	-4.546724	-1.913012	0.935278
N	-0.398196	1.602468	-0.066008
C	-0.058109	2.562645	0.825030
O	0.974586	3.218761	0.775058
C	0.537383	1.259302	-1.150698
C	1.528275	0.191268	-0.754355
C	3.352941	-1.823746	-0.092509
C	2.776451	0.536645	-0.227451
C	1.189793	-1.161182	-0.925088
C	2.088747	-2.159729	-0.592700
C	3.702246	-0.488568	0.086808
H	-0.610102	-0.017521	2.035574
H	-4.413904	-0.386045	-1.146621
H	-2.515152	-1.591277	2.506019
H	-2.545019	1.231378	-1.590422
H	-0.819012	2.726008	1.607747
H	-0.065205	0.903695	-1.992215
H	1.041425	2.183636	-1.449845
H	4.056441	-2.614253	0.154358
H	0.210842	-1.415923	-1.333128
H	1.825454	-3.206969	-0.728556
H	4.668839	-0.141380	0.469398
H	3.111616	1.567766	-0.067231

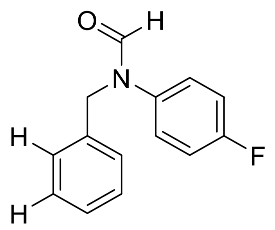

1-Ph (**D**)

C	-2.542851	-0.688197	0.459281
C	-2.389677	1.020446	-1.752058
C	-3.579835	0.203681	0.207836
C	-1.428862	-0.726576	-0.382968
C	-1.348455	0.135462	-1.477615
C	-3.482372	1.037743	-0.899239
F	-4.487721	1.897372	-1.149208
N	-0.356518	-1.623141	-0.098732
C	0.057739	-2.515066	-1.027880
O	1.097691	-3.158595	-0.952609
C	0.489281	-1.355890	1.078099
C	1.468631	-0.227916	0.856907
C	3.245351	1.887662	0.516057
C	2.762216	-0.474149	0.359621
C	1.081533	1.080708	1.158517
C	1.960185	2.143959	0.987010
C	3.670200	0.594681	0.195501
H	-2.598849	-1.367373	1.308792
H	-2.354063	1.699952	-2.600652
H	-4.460991	0.252256	0.843564
H	-0.457225	0.124256	-2.103280
H	-0.646522	-2.634906	-1.868357
H	1.000052	-2.291097	1.328220
H	-0.182145	-1.096177	1.902577
H	3.955062	2.707558	0.407701
H	0.076917	1.257764	1.546489
H	1.656740	3.158852	1.236101
H	3.109041	-1.482787	0.107471
H	4.685446	0.439519	-0.187145

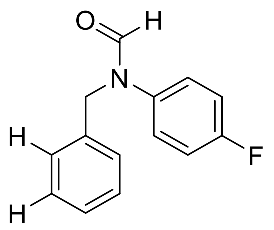

1-^{t}Bu (**D**)

C	-2.536068	-0.663191	0.763849
C	-2.536493	0.864815	-1.581437
C	-3.601099	0.192569	0.502404
C	-1.471891	-0.755246	-0.136003
C	-1.468181	0.015602	-1.299526
C	-3.578886	0.937698	-0.670111
N	-0.365136	-1.606559	0.160331
C	0.586947	-1.178702	1.202555
C	1.548645	-0.117617	0.722436
C	3.309456	1.871959	-0.077553
C	2.811218	-0.458924	0.204282
C	1.175691	1.226384	0.812073
C	2.047893	2.224117	0.405028
C	3.729871	0.544357	-0.189287
C	-0.021541	-2.602665	-0.686438
O	1.030137	-3.229374	-0.626869
F	-4.609268	1.764660	-0.928489
H	-2.529807	-1.271321	1.667156
H	-2.560383	1.475423	-2.481066
H	-4.445252	0.281759	1.182579
H	-0.613923	-0.037670	-1.972878
H	-0.009442	-0.781553	2.029830
H	1.111169	-2.073296	1.552201
H	3.983165	2.671153	-0.374333
H	0.194709	1.480937	1.216874
H	1.762559	3.272003	0.474627
H	-0.793993	-2.825816	-1.441546
H	3.122665	-1.507270	0.132252
H	4.727233	0.285767	-0.562940

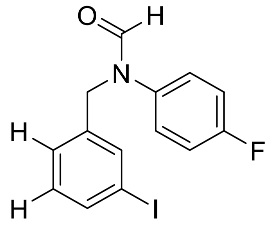

1-I_2 (**D**)

C	-3.271160	0.989756	1.022921
C	-5.575664	0.736682	-0.547635
C	-4.340932	0.228131	1.490236
C	-3.353792	1.625895	-0.216631
C	-4.503257	1.499337	-0.999252
C	-5.468951	0.115606	0.690563
F	-6.502691	-0.625180	1.128498
N	-2.242535	2.377991	-0.711317
C	-1.895562	3.557585	-0.151514
O	-0.860274	4.162846	-0.406202
C	-1.336940	1.740583	-1.680417
C	-0.289182	0.860185	-1.035081
C	1.643886	-0.841963	0.069014
C	0.977422	1.378697	-0.695814
C	-0.582507	-0.485878	-0.798890
C	0.373412	-1.326349	-0.239965
C	1.936077	0.499461	-0.162996
H	-2.360099	1.082254	1.611978
H	-6.486220	0.622830	-1.131395
H	-4.305471	-0.282175	2.449925
H	-4.555633	2.008412	-1.960307
H	-2.644769	3.952361	0.555014
H	-1.961785	1.136552	-2.345613
H	-0.870697	2.537603	-2.268849
H	2.402342	-1.497295	0.489439
H	-1.564889	-0.872159	-1.068265
I	-0.093233	-3.353887	0.123702
H	2.936173	0.879527	0.074855
H	1.258486	2.424847	-0.862516

S4.2.2: DMC analysis of OH···R interactions in closed conformers of compounds **1**-R.

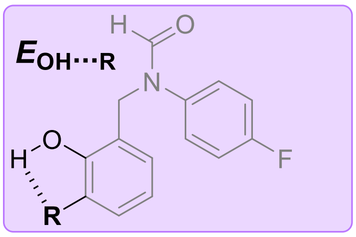

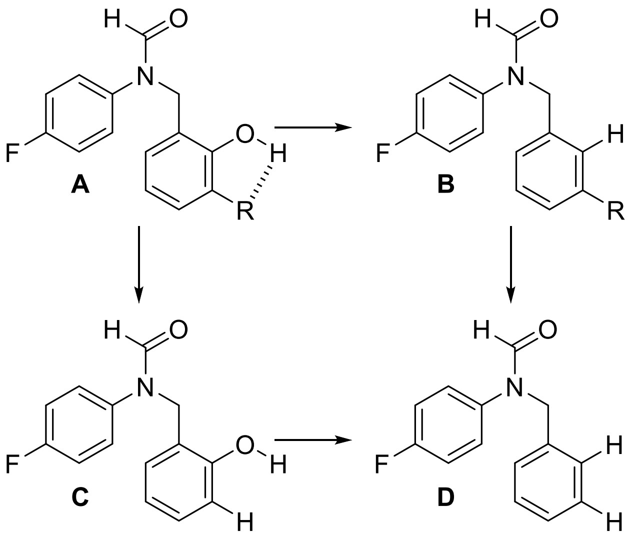

Table S7: Energies (kJ/mol) of species involved in the DMC cycle calculation of $E_{OH\cdots R}$

**1-R**	**E(A)**	**E(B)**	**E(C)**	**E(D)**	$E_{OH\cdots R}$
**1**-F	-2480535.99	-2283052.3	-2220020.49	-2022535.78	1.02
**1**-Br	-8976973.22	-8779479.92	-2220022.05	-2022535.99	-7.24
**1**-CF_3	-3104811.22	-2907321.78	-2220022.64	-2022535.79	-2.59
**1**-CH_3	-2323210.16	-2125721.06	-2220022.98	-2022536.21	-2.33
**1**-Cl	-3426693.76	-3229201.74	-2220022.53	-2022536.52	-6.01
**1**-CN	-2462182.45	-2264685.56	-2220022.16	-2022535.92	-10.65
**1**-NO_2	-2756878.55	-2559369.33	-2220018.06	-2022533.56	-24.72
**1**-OMe	-2520640.23	-2323149.48	-2220018.54	-2022534.29	-6.5
**1**-Ph	-2826508.49	-2629016.43	-2220022.48	-2022535.90	-5.48
**1**-^{t}Bu	-2632730.38	-2435248.84	-2220020.00	-2022532.32	6.14
**1**-I_2	-3769191.80	-3571686.98	-2994620.10	-2797113.34	1.90
**1**-H			-2220023.54		
**1**-control				-2022538.79	

Geometries of Species B

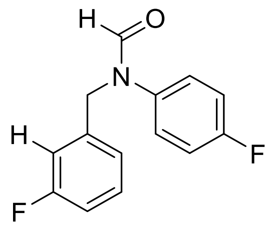

1-F (**B**)

C	2.963363	-0.714416	0.256137
C	2.309237	2.003079	0.125616
C	3.961204	0.233809	0.053738
C	1.626402	-0.312596	0.390431
C	1.311682	1.051320	0.322462
C	3.618765	1.576156	-0.008851
F	4.590539	2.493204	-0.201835
N	0.593970	-1.261302	0.636635
C	0.643746	-2.560489	0.189731
O	1.541383	-3.074444	-0.441366
C	-0.646413	-0.819072	1.255923
C	-1.673509	-0.276205	0.282003
C	-3.669799	0.749308	-1.422343
C	-2.844044	0.268040	0.815234
C	-1.515055	-0.301384	-1.104406
C	-2.500498	0.204832	-1.951757
C	-3.813954	0.769001	-0.047499
H	3.222093	-1.765778	0.306330
H	2.074966	3.063795	0.071186
H	5.002828	-0.062612	-0.049352
H	0.282236	1.389424	0.409986
H	-0.268080	-3.120584	0.475342
H	-1.081598	-1.667715	1.798275
H	-0.413226	-0.063820	2.014907
H	-4.459490	1.155186	-2.050950
H	-0.600089	-0.713414	-1.527762
H	-2.356825	0.178599	-3.029498
F	-4.926861	1.289587	0.528249
H	-2.972972	0.288492	1.903433

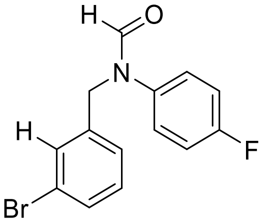

1-Br (**B**)

C	3.020989	-0.374447	0.439375
C	2.093350	2.247215	0.133103
C	3.924256	0.665592	0.247940
C	1.643187	-0.113986	0.474370
C	1.190752	1.202703	0.319223
C	3.446865	1.958689	0.097313
F	4.327747	2.965113	-0.085270
N	0.705949	-1.159579	0.708743
C	0.921531	-2.461225	0.322223
O	1.899693	-2.892823	-0.248222
C	-0.605248	-0.833601	1.248639
C	-1.634956	-0.455028	0.202254
C	-3.632198	0.260513	-1.633718
C	-2.887646	-0.021134	0.662288
C	-1.398830	-0.524944	-1.167142
C	-2.389422	-0.171717	-2.084321
C	-3.869505	0.333296	-0.264089
H	3.384641	-1.388788	0.557828
H	1.752342	3.272979	0.012086
H	4.995818	0.480178	0.221092
H	0.127441	1.428349	0.329850
H	0.065956	-3.109899	0.594333
H	-0.971108	-1.699978	1.813716
H	-0.496330	-0.022839	1.977616
H	-4.417116	0.544379	-2.331216
H	-0.422213	-0.850341	-1.523678
H	-2.192049	-0.229162	-3.152482
Br	-5.553851	0.934233	0.379793
H	-3.030045	0.016251	1.748355

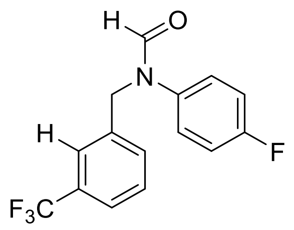

1-CF3 (**B**)

C	-3.540500	-0.351989	0.557288
C	-2.721790	-0.151446	-2.109832
C	-4.485005	-0.182124	-0.449714
C	-2.175622	-0.416483	0.241100
C	-1.777784	-0.315045	-1.097990
C	-4.060998	-0.083466	-1.766095
N	-1.199606	-0.621895	1.256091
C	0.092439	-1.190835	0.903845
C	1.124902	-0.169454	0.470543
C	3.126156	1.632230	-0.314108
C	2.396817	-0.638487	0.114559
C	0.877288	1.199135	0.428859
C	1.867718	2.102211	0.038156
C	3.393166	0.263183	-0.276238
C	4.744310	-0.264071	-0.648765
F	4.676952	-1.121827	-1.695807
F	5.298025	-0.979844	0.361871
F	5.611518	0.696838	-0.975250
C	-1.352024	-0.168317	2.545654
O	-2.302262	0.437375	2.990388
F	-4.980167	0.080681	-2.740436
H	-3.863559	-0.429577	1.589502
H	-2.423306	-0.071490	-3.152872
H	-5.547195	-0.131734	-0.220074
H	-0.725188	-0.351433	-1.368768
H	0.478560	-1.739353	1.771926
H	-0.053092	-1.940316	0.117289
H	3.912517	2.318307	-0.621071
H	-0.112297	1.566510	0.700597
H	1.654811	3.168426	0.010383
H	-0.472560	-0.421696	3.169781
H	2.537774	-1.724012	0.169188

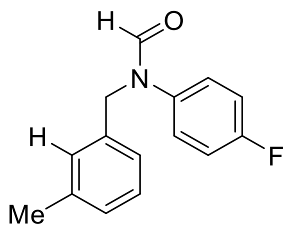

1-Me (**B**)

C	3.474545	-0.892657	0.197016
C	2.851582	1.834247	0.169986
C	4.486086	0.051505	0.049881
C	2.139559	-0.482240	0.325046
C	1.840482	0.886483	0.310503
C	4.158570	1.398866	0.037409
N	1.093770	-1.429180	0.508292
F	5.142682	2.312575	-0.106544
C	1.132919	-2.699219	-0.014968
O	2.033624	-3.188802	-0.662006
C	-0.152543	-1.005347	1.132976
C	-1.163747	-0.409620	0.174194
C	-3.120752	0.706849	-1.476030
C	-2.359241	0.084459	0.707367
C	-0.965809	-0.337646	-1.202978
C	-1.940065	0.219364	-2.030133
C	-3.353227	0.649182	-0.099922
C	-4.624614	1.168401	0.518605
H	3.721330	-1.948170	0.207000
H	2.629753	2.899017	0.155560
H	5.526041	-0.252242	-0.049387
H	0.812243	1.229631	0.393524
H	0.209598	-3.262104	0.223556
H	-0.599351	-1.874050	1.632509
H	0.080446	-0.289713	1.929627
H	-3.883629	1.143736	-2.120647
H	-0.035636	-0.709898	-1.631327
H	-1.776131	0.274543	-3.104377
H	-5.287309	1.575377	-0.251256
H	-4.424850	1.973676	1.241353
H	-5.175245	0.371626	1.040696
H	-2.471080	0.001354	1.794474

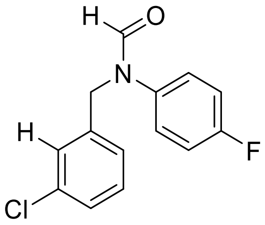

1-Cl (**B**)

C	3.003825	-0.560907	0.340887
C	2.238176	2.122231	0.140101
C	3.966062	0.425580	0.150391
C	1.647264	-0.215400	0.427627
C	1.276220	1.131684	0.324947
C	3.568531	1.750205	0.052300
F	4.505546	2.704905	-0.129686
N	0.651738	-1.205601	0.660739
C	0.770988	-2.506961	0.233130
O	1.704658	-2.987204	-0.371645
C	-0.622193	-0.814053	1.245243
C	-1.657338	-0.353469	0.237961
C	-3.670957	0.502447	-1.522139
C	-2.887050	0.094092	0.740794
C	-1.450975	-0.365172	-1.138510
C	-2.448728	0.059275	-2.017192
C	-3.877841	0.516133	-0.146929
H	3.305830	-1.599120	0.418195
H	1.960369	3.170691	0.058988
H	5.022068	0.173402	0.083823
H	0.230682	1.425102	0.376059
H	-0.120463	-3.104470	0.507520
H	-1.023781	-1.669270	1.802715
H	-0.444531	-0.028186	1.987885
H	-4.464673	0.837674	-2.186149
H	-0.492516	-0.702827	-1.530687
H	-2.273015	0.046142	-3.090519
Cl	-5.401671	1.065246	0.515014
H	-3.016223	0.087830	1.829137

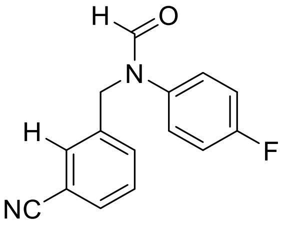

1-CN (**B**)

C	-2.315655	0.771237	1.619921
C	-1.558806	0.970560	-1.065870
C	-3.280361	0.968354	0.637054
C	-0.960883	0.677391	1.269553
C	-0.594394	0.779304	-0.078617
C	-2.887191	1.065600	-0.688959
F	-3.826084	1.253466	-1.639512
N	0.036909	0.438796	2.256663
C	-0.075740	0.866056	3.559652
O	-1.003924	1.476412	4.042063
C	1.308172	-0.145795	1.859866
C	2.340861	0.864034	1.400030
C	4.343080	2.651756	0.548554
C	3.577674	0.377188	0.956255
C	2.129940	2.238693	1.408555
C	3.119979	3.133585	0.987086
C	4.574045	1.269034	0.531778
H	-2.615360	0.693854	2.658992
H	-1.284390	1.049997	-2.115470
H	-4.335153	1.040349	0.893317
H	0.449287	0.720199	-0.377796
H	0.815411	0.585643	4.155020
H	1.714049	-0.705941	2.711407
H	1.128803	-0.885929	1.071753
H	5.129334	3.325315	0.214904
H	1.166485	2.622426	1.743785
H	2.928261	4.203928	1.002518
C	5.817584	0.705711	0.086993
N	6.778143	0.153207	-0.252908
H	3.716372	-0.709982	0.962577

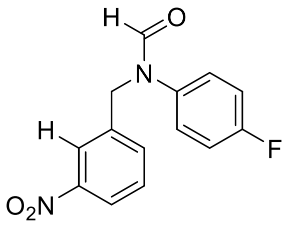

1-NO_2 (**B**)

C	-2.482661	0.773514	1.616948
C	-1.704986	0.856094	-1.069401
C	-3.441209	0.911955	0.618938
C	-1.123924	0.682687	1.281649
C	-0.746795	0.723741	-0.066732
C	-3.037491	0.952892	-0.707290
N	-0.133206	0.507774	2.289486
C	1.143748	-0.094650	1.941786
C	2.182315	0.891884	1.443706
C	4.195815	2.630609	0.538577
C	3.431576	0.362521	1.062266
C	1.966410	2.258806	1.364460
C	2.965731	3.133823	0.913492
C	4.424482	1.251291	0.612601
N	5.731126	0.755567	0.207803
O	5.942351	-0.458055	0.286553
O	6.554925	1.543032	-0.194649
F	-3.970271	1.088582	-1.672482
C	-0.261021	1.017190	3.561187
O	-1.193887	1.659795	3.989741
H	-2.789299	0.741774	2.656185
H	-1.423075	0.889220	-2.119233
H	-4.498981	0.982232	0.862565
H	0.300321	0.661816	-0.352927
H	1.540035	-0.607707	2.826786
H	0.978098	-0.876800	1.192649
H	4.998437	3.271710	0.184826
H	0.994061	2.658227	1.652995
H	2.772491	4.202221	0.859643
H	0.621098	0.772593	4.185161
H	3.551004	-0.723821	1.144808

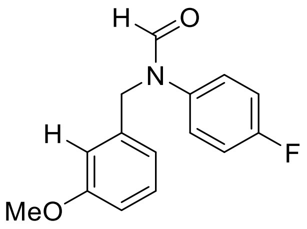

1-OMe (**B**)

C	3.790921	-0.485797	0.425204
C	2.808908	2.128909	0.276583
C	4.671605	0.580995	0.278360
C	2.409189	-0.255736	0.493191
C	1.929078	1.057882	0.417372
C	4.166837	1.870237	0.205623
F	5.025441	2.903140	0.065519
N	1.496418	-1.330998	0.680513
C	1.722247	-2.596551	0.195448
O	2.695179	-2.970372	-0.423651
C	0.190114	-1.063167	1.266374
C	-0.866058	-0.631155	0.269919
C	-2.926901	0.174798	-1.465657
C	-2.103646	-0.243181	0.772068
C	-0.663230	-0.614723	-1.113896
C	-1.682697	-0.216435	-1.970492
C	-3.134446	0.161253	-0.090643
H	4.175821	-1.497690	0.484327
H	2.446763	3.152865	0.215599
H	5.746080	0.418627	0.226228
H	0.861334	1.259494	0.454244
H	0.882334	-3.276564	0.436979
H	-0.148913	-1.972696	1.778153
H	0.304048	-0.303011	2.047683
H	-3.716851	0.486057	-2.144094
H	0.305624	-0.904597	-1.518344
H	-1.514949	-0.204260	-3.045479
O	-4.289336	0.514714	0.550467
C	-5.367090	0.945829	-0.261592
H	-5.086903	1.839841	-0.835192
H	-5.679065	0.146516	-0.947642
H	-6.185442	1.186233	0.420460
H	-2.262412	-0.260457	1.856370

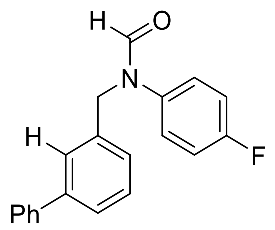

1-Ph (**B**)

C	-4.802760	0.168442	0.389833
C	-3.595521	-2.292988	-0.163066
C	-5.588033	-0.942849	0.097956
C	-3.404206	0.061230	0.401644
C	-2.811488	-1.177364	0.122573
C	-4.972580	-2.154805	-0.174209
F	-5.737030	-3.231561	-0.457233
N	-2.585200	1.176573	0.731940
C	-2.948272	2.476610	0.475143
O	-3.985229	2.853682	-0.026871
C	-1.226701	0.947759	1.205411
C	-0.198929	0.793790	0.101958
C	1.787282	0.472948	-1.835490
C	1.108364	0.449703	0.471588
C	-0.492630	0.978239	-1.245879
C	0.495615	0.815471	-2.217905
C	2.120093	0.292547	-0.487407
H	-5.276484	1.119915	0.602839
H	-3.144355	-3.258611	-0.380665
H	-6.673762	-0.874509	0.086953
H	-1.729806	-1.285600	0.111115
H	-2.156609	3.185133	0.786985
H	-1.222078	0.062887	1.851309
H	-0.941144	1.788852	1.849392
H	2.570167	0.358663	-2.584748
H	-1.508460	1.237526	-1.542919
H	0.255812	0.959661	-3.269362
C	3.505099	-0.075280	-0.090934
C	6.129476	-0.776801	0.626630
C	4.202424	0.659523	0.879956
C	4.146599	-1.161693	-0.700745
C	5.448490	-1.508000	-0.347081
C	5.503761	0.308520	1.238117
H	3.731199	1.532210	1.334618
H	3.609832	-1.742558	-1.450486
H	5.932022	-2.356632	-0.829094
H	6.034596	0.893089	1.988404
H	7.145710	-1.050570	0.905682
H	1.274734	0.296847	1.544049

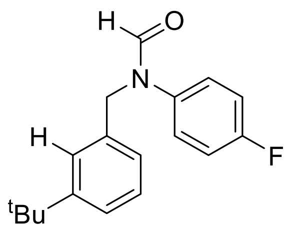

1-^{t}Bu (**B**)

C	4.023084	0.086718	0.838893
C	6.742595	0.696706	0.909533
C	4.957490	-0.940379	0.955820
C	4.437199	1.421836	0.754193
C	5.807269	1.718942	0.786228
C	6.302679	-0.615354	0.994118
N	3.471644	2.454523	0.597492
C	2.169372	2.138891	0.025386
C	1.135507	1.682472	1.035882
C	-0.866291	0.851184	2.772959
C	-0.132196	1.329661	0.552804
C	1.387961	1.614690	2.401731
C	0.385748	1.196950	3.272279
C	-1.169377	0.905767	1.405444
C	-2.557180	0.523149	0.871713
C	3.648198	3.720656	1.100260
O	4.614548	4.128660	1.708449
F	7.213233	-1.605388	1.116544
H	2.965126	-0.164459	0.828708
H	7.809038	0.910309	0.934987
H	4.647673	-1.980876	1.024218
H	6.140495	2.748722	0.721233
H	1.796017	3.031076	-0.492522
H	2.303689	1.378359	-0.751948
H	-1.628132	0.528206	3.476799
H	2.373481	1.879833	2.783482
H	0.578488	1.139391	4.341604
H	2.773702	4.365552	0.886186
C	-2.453873	-0.684607	-0.081732
H	-1.810913	-0.523551	-0.955758
H	-3.451732	-0.951302	-0.452762
H	-2.044162	-1.547353	0.457167
C	-3.221066	1.729822	0.177446
H	-2.654227	2.138177	-0.667948
H	-3.348748	2.546865	0.897758
H	-4.211438	1.441925	-0.197788
C	-3.498111	0.101386	2.007120
H	-3.117895	-0.779083	2.538957
H	-4.476338	-0.156984	1.583743
H	-3.647028	0.912156	2.730446
H	-0.231352	1.420196	-0.534940

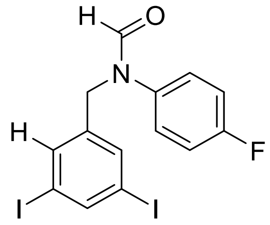

1-I_2 (**B**)

C	-2.194697	0.818221	1.387644
C	-1.290490	0.666289	-1.256107
C	-3.104357	0.872837	0.336710
C	-0.822407	0.694649	1.125836
C	-0.381393	0.619277	-0.201165
C	-2.638104	0.797940	-0.967185
F	-3.524112	0.854320	-1.983301
N	0.120497	0.604135	2.188510
C	-0.068807	1.201557	3.413399
O	-1.021342	1.868478	3.751221
C	1.397793	-0.055377	1.965462
C	2.504947	0.855768	1.466732
C	4.649769	2.443331	0.597007
C	3.774290	0.281320	1.284033
C	2.317447	2.210819	1.212717
C	3.386553	2.996709	0.778578
C	4.833371	1.083252	0.851750
H	-2.550016	0.878752	2.409851
H	-0.959905	0.607441	-2.290483
H	-4.171959	0.967074	0.523195
H	0.678618	0.535099	-0.428943
H	0.782025	1.003441	4.094277
H	1.721327	-0.513198	2.908376
H	1.250954	-0.886621	1.266595
H	5.484977	3.053434	0.263574
H	1.331644	2.651726	1.350713
I	3.088000	5.049947	0.393870
I	6.748159	0.226496	0.588287
H	3.855255	-0.789329	1.503969

Geometries of Species C

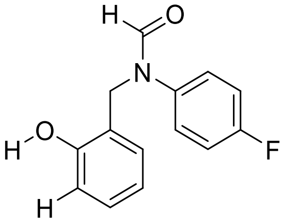

1-F (**C**)

C	3.086083	-0.733945	0.171971
C	2.431956	1.983549	0.041451
C	4.083923	0.214280	-0.030427
C	1.749122	-0.332126	0.306266
C	1.434401	1.031791	0.238297
C	3.741484	1.556627	-0.093016
F	4.713259	2.473675	-0.286000
N	0.716689	-1.280831	0.552469
C	0.766465	-2.580019	0.105566
O	1.664102	-3.093973	-0.525531
C	-0.523694	-0.838601	1.171758
C	-1.550789	-0.295734	0.197838
C	-3.547080	0.729779	-1.506508
C	-2.721325	0.248510	0.731069
C	-1.392336	-0.320913	-1.188571
C	-2.377779	0.185303	-2.035922
C	-3.691235	0.749471	-0.131664
O	-2.881411	0.273906	2.082251
H	3.344813	-1.785307	0.222165
H	2.197685	3.044266	-0.012980
H	5.125547	-0.082141	-0.133517
H	0.404956	1.369895	0.325821
H	-0.145361	-3.140113	0.391177
H	-0.958878	-1.687244	1.714109
H	-0.290507	-0.083349	1.930741
H	-4.336770	1.135657	-2.135115
H	-0.477370	-0.732944	-1.611927
H	-2.234106	0.159070	-3.113663
H	-3.741660	0.661486	2.288495
H	-4.590186	1.169975	0.333396

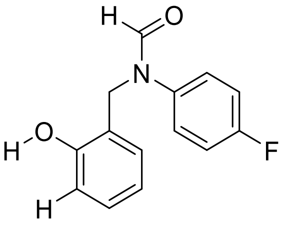

1-Br (**C**)

C	3.130124	-0.377916	0.366350
C	2.202486	2.243746	0.060078
C	4.033392	0.662123	0.174916
C	1.752322	-0.117455	0.401346
C	1.299888	1.199234	0.246198
C	3.556001	1.955220	0.024288
F	4.436882	2.961644	-0.158295
N	0.815084	-1.163048	0.635718
C	1.030667	-2.464694	0.249198
O	2.008829	-2.896291	-0.321247
C	-0.496112	-0.837070	1.175614
C	-1.525821	-0.458497	0.129229
C	-3.523062	0.257044	-1.706743
C	-2.778511	-0.024602	0.589263
C	-1.289694	-0.528413	-1.240166
C	-2.280287	-0.175185	-2.157346
C	-3.760370	0.329827	-0.337113
O	-2.954729	0.021662	1.933274
H	3.493777	-1.392257	0.484803
H	1.861477	3.269510	-0.060938
H	5.104954	0.476710	0.148067
H	0.236577	1.424880	0.256825
H	0.175092	-3.113368	0.521308
H	-0.861972	-1.703447	1.740691
H	-0.387194	-0.026308	1.904592
H	-4.307981	0.540910	-2.404241
H	-0.313077	-0.853810	-1.596703
H	-2.082913	-0.232631	-3.225506
H	-3.844227	0.346140	2.132375
H	-4.731604	0.676342	0.034164

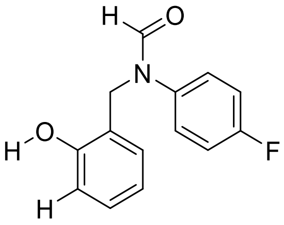

1-CF_3 (**C**)

C	-3.125712	-0.320143	0.478328
C	-2.307002	-0.119600	-2.188792
C	-4.070217	-0.150278	-0.528674
C	-1.760834	-0.384637	0.162140
C	-1.362996	-0.283199	-1.176951
C	-3.646210	-0.051620	-1.845056
N	-0.784818	-0.590049	1.177131
C	0.507227	-1.158989	0.824884
C	1.539689	-0.137608	0.391583
C	3.540944	1.664076	-0.393068
C	2.811605	-0.606641	0.035599
C	1.292076	1.230981	0.349899
C	2.282506	2.134057	-0.040804
C	3.807954	0.295029	-0.355199
O	2.986408	-1.952823	0.103346
C	-0.937236	-0.136471	2.466694
O	-1.887474	0.469221	2.911428
F	-4.565379	0.112527	-2.819396
H	-3.448771	-0.397731	1.510542
H	-2.008518	-0.039644	-3.231832
H	-5.132407	-0.099888	-0.299034
H	-0.310400	-0.319587	-1.447729
H	0.893348	-1.707507	1.692966
H	0.361696	-1.908470	0.038329
H	4.327305	2.350153	-0.700031
H	0.302491	1.598356	0.621636
H	2.069599	3.200272	-0.068577
H	3.886025	-2.209062	-0.132330
H	-0.057772	-0.389850	3.090821
H	4.796870	-0.090874	-0.627855

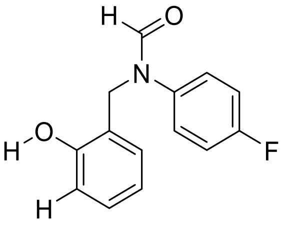

1-Me (**C**)

C	3.079231	-0.768228	0.183375
C	2.456268	1.958676	0.156345
C	4.090773	0.175935	0.036240
C	1.744245	-0.357811	0.311405
C	1.445168	1.010912	0.296862
C	3.763256	1.523295	0.023768
N	0.698456	-1.304751	0.494651
F	4.747369	2.437004	-0.120185
C	0.737605	-2.574790	-0.028609
O	1.638310	-3.064373	-0.675647
C	-0.547856	-0.880918	1.119335
C	-1.559061	-0.285191	0.160553
C	-3.516066	0.831278	-1.489671
C	-2.754555	0.208888	0.693726
C	-1.361122	-0.213217	-1.216619
C	-2.335378	0.343793	-2.043774
C	-3.748541	0.773611	-0.113563
O	-2.894356	0.105005	2.052628
H	3.326016	-1.823741	0.193359
H	2.234440	3.023447	0.141919
H	5.130728	-0.127813	-0.063028
H	0.416929	1.354060	0.379882
H	-0.185716	-3.137675	0.209915
H	-0.994664	-1.749621	1.618868
H	-0.314867	-0.165283	1.915986
H	-4.278942	1.268165	-2.134288
H	-0.430950	-0.585469	-1.644968
H	-2.171444	0.398973	-3.118018
H	-3.741588	0.474410	2.323035
H	-4.673687	1.151430	0.336519

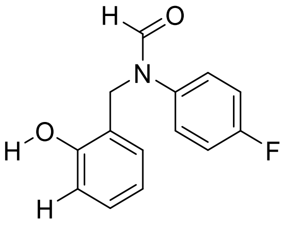

1-Cl (**C**)

C	3.116526	-0.568666	0.263511
C	2.350877	2.114471	0.062725
C	4.078764	0.417821	0.073015
C	1.759965	-0.223159	0.350250
C	1.388922	1.123925	0.247571
C	3.681232	1.742446	-0.025076
F	4.618247	2.697146	-0.207062
N	0.764439	-1.213360	0.583362
C	0.883689	-2.514721	0.155754
O	1.817360	-2.994963	-0.449022
C	-0.509492	-0.821812	1.167867
C	-1.544637	-0.361228	0.160585
C	-3.558256	0.494688	-1.599516
C	-2.774349	0.086332	0.663417
C	-1.338274	-0.372931	-1.215887
C	-2.336027	0.051516	-2.094569
C	-3.765140	0.508374	-0.224305
O	-2.934275	0.078580	2.010873
H	3.418531	-1.606879	0.340819
H	2.073070	3.162932	-0.018388
H	5.134769	0.165642	0.006447
H	0.343383	1.417343	0.298683
H	-0.007762	-3.112230	0.430143
H	-0.911080	-1.677030	1.725338
H	-0.331830	-0.035945	1.910508
H	-4.351972	0.829914	-2.263526
H	-0.379815	-0.710586	-1.608063
H	-2.160314	0.038383	-3.167895
H	-3.806939	0.431681	2.232128
H	-4.719610	0.852317	0.190311

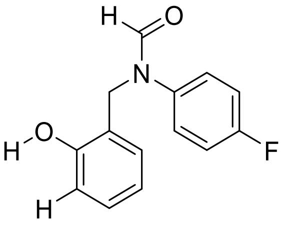

1-CN (**C**)

C	-2.315655	0.771237	1.619921
C	-1.558806	0.970560	-1.065870
C	-3.280361	0.968354	0.637054
C	-0.960883	0.677391	1.269553
C	-0.594394	0.779304	-0.078617
C	-2.887191	1.065600	-0.688959
F	-3.826084	1.253466	-1.639512
N	0.036909	0.438796	2.256663
C	-0.075740	0.866056	3.559652
O	-1.003924	1.476412	4.042063
C	1.308172	-0.145795	1.859866
C	2.340861	0.864034	1.400030
C	4.343080	2.651756	0.548554
C	3.577674	0.377188	0.956255
C	2.129940	2.238693	1.408555
C	3.119979	3.133585	0.987086
C	4.574045	1.269034	0.531778
O	3.748986	-0.965626	0.964064
H	-2.615360	0.693854	2.658992
H	-1.284390	1.049997	-2.115470
H	-4.335153	1.040349	0.893317
H	0.449287	0.720199	-0.377796
H	0.815411	0.585643	4.155020
H	1.714049	-0.705941	2.711407
H	1.128803	-0.885929	1.071753
H	5.129334	3.325315	0.214904
H	1.166485	2.622426	1.743785
H	2.928261	4.203928	1.002518
H	4.630937	-1.198805	0.639348
H	5.523277	0.839032	0.192260

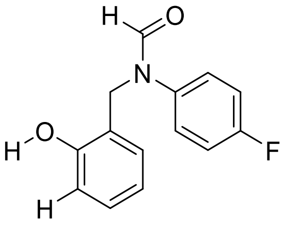

1-NO_2 (**C**)

C	-2.482661	0.773514	1.616948
C	-1.704986	0.856094	-1.069401
C	-3.441209	0.911955	0.618938
C	-1.123924	0.682687	1.281649
C	-0.746795	0.723741	-0.066732
C	-3.037491	0.952892	-0.707290
N	-0.133206	0.507774	2.289486
C	1.143748	-0.094650	1.941786
C	2.182315	0.891884	1.443706
C	4.195815	2.630609	0.538577
C	3.431576	0.362521	1.062266
C	1.966410	2.258806	1.364460
C	2.965731	3.133823	0.913492
C	4.424482	1.251291	0.612601
O	3.577456	-0.964429	1.163089
F	-3.970271	1.088582	-1.672482
C	-0.261021	1.017190	3.561187
O	-1.193887	1.659795	3.989741
H	-2.789299	0.741774	2.656185
H	-1.423075	0.889220	-2.119233
H	-4.498981	0.982232	0.862565
H	0.300321	0.661816	-0.352927
H	1.540035	-0.607707	2.826786
H	0.978098	-0.876800	1.192649
H	4.998437	3.271710	0.184826
H	0.994061	2.658227	1.652995
H	2.772491	4.202221	0.859643
H	4.486332	-1.179909	0.869623
H	0.621098	0.772593	4.185161
H	5.408755	0.877871	0.307673

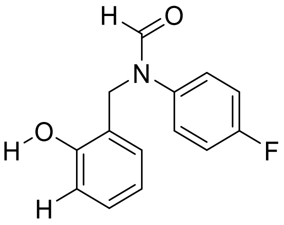

1-OMe (**C**)

C	3.147367	-0.347307	0.290033
C	2.165355	2.267399	0.141412
C	4.028052	0.719485	0.143189
C	1.765636	-0.117247	0.358021
C	1.285525	1.196371	0.282201
C	3.523284	2.008726	0.070453
F	4.381888	3.041630	-0.069652
N	0.852865	-1.192509	0.545342
C	1.078693	-2.458061	0.060278
O	2.051626	-2.831883	-0.558821
C	-0.453439	-0.924677	1.131204
C	-1.509611	-0.492665	0.134748
C	-3.570454	0.313287	-1.600828
C	-2.747199	-0.104691	0.636897
C	-1.306783	-0.476233	-1.249067
C	-2.326250	-0.077945	-2.105663
C	-3.777999	0.299743	-0.225813
O	-2.944613	-0.126173	1.985142
H	3.532268	-1.359200	0.349156
H	1.803210	3.291354	0.080429
H	5.102527	0.557117	0.091058
H	0.217781	1.397983	0.319074
H	0.238781	-3.138074	0.301809
H	-0.792466	-1.834207	1.642983
H	-0.339505	-0.164521	1.912512
H	-4.360404	0.624547	-2.279265
H	-0.337929	-0.766108	-1.653514
H	-2.158502	-0.065771	-3.180649
H	-3.846023	0.176577	2.159273
H	-4.703682	0.583053	0.288058

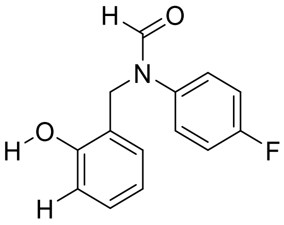

1-Ph (**C**)

C	-3.136396	-0.005016	0.440848
C	-1.929157	-2.466445	-0.112052
C	-3.921669	-1.116307	0.148971
C	-1.737842	-0.112227	0.452659
C	-1.145124	-1.350821	0.173588
C	-3.306216	-2.328263	-0.123194
F	-4.070666	-3.405019	-0.406218
N	-0.918836	1.003116	0.782955
C	-1.281908	2.303153	0.526158
O	-2.318865	2.680225	0.024144
C	0.439663	0.774302	1.256426
C	1.467435	0.620333	0.152973
C	3.453645	0.299491	-1.784476
C	2.774728	0.276245	0.522603
C	1.173733	0.804782	-1.194865
C	2.161979	0.642013	-2.166890
C	3.786456	0.119090	-0.436392
H	-3.610120	0.946457	0.653854
H	-1.477991	-3.432068	-0.329650
H	-5.007399	-1.047967	0.137967
H	-0.063442	-1.459058	0.162130
H	-0.490245	3.011675	0.837999
H	0.444286	-0.110570	1.902323
H	0.725220	1.615395	1.900407
H	4.236530	0.185206	-2.533734
H	0.157904	1.064068	-1.491905
H	1.922176	0.786203	-3.218347
O	2.982126	0.085694	1.859539
H	3.882611	-0.231639	2.006322
H	4.807383	-0.152046	-0.144141

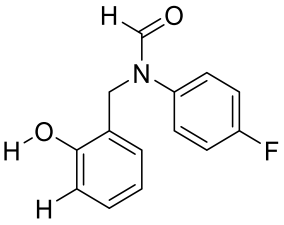

1-^{t}Bu (**C**)

C	4.023084	0.086718	0.838893
C	6.742595	0.696706	0.909533
C	4.957490	-0.940379	0.955820
C	4.437199	1.421836	0.754193
C	5.807269	1.718942	0.786228
C	6.302679	-0.615354	0.994118
N	3.471644	2.454523	0.597492
C	2.169372	2.138891	0.025386
C	1.135507	1.682472	1.035882
C	-0.866291	0.851184	2.772959
C	-0.132196	1.329661	0.552804
C	1.387961	1.614690	2.401731
C	0.385748	1.196950	3.272279
C	-1.169377	0.905767	1.405444
O	-0.256251	1.442929	-0.808076
C	3.648198	3.720656	1.100260
O	4.614548	4.128660	1.708449
F	7.213233	-1.605388	1.116544
H	2.965126	-0.164459	0.828708
H	7.809038	0.910309	0.934987
H	4.647673	-1.980876	1.024218
H	6.140495	2.748722	0.721233
H	1.796017	3.031076	-0.492522
H	2.303689	1.378359	-0.751948
H	-1.628132	0.528206	3.476799
H	2.373481	1.879833	2.783482
H	0.578488	1.139391	4.341604
H	-1.139275	1.183824	-1.081045
H	2.773702	4.365552	0.886186
H	-2.160059	0.632636	1.024440

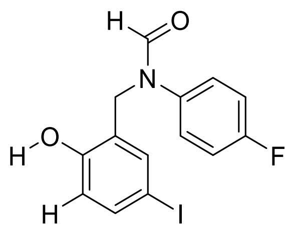

1-I_2 (**C**)

C	-2.194697	0.818221	1.387644
C	-1.290490	0.666289	-1.256107
C	-3.104357	0.872837	0.336710
C	-0.822407	0.694649	1.125836
C	-0.381393	0.619277	-0.201165
C	-2.638104	0.797940	-0.967185
F	-3.524112	0.854320	-1.983301
N	0.120497	0.604135	2.188510
C	-0.068807	1.201557	3.413399
O	-1.021342	1.868478	3.751221
C	1.397793	-0.055377	1.965462
C	2.504947	0.855768	1.466732
C	4.649769	2.443331	0.597007
C	3.774290	0.281320	1.284033
C	2.317447	2.210819	1.212717
C	3.386553	2.996709	0.778578
C	4.833371	1.083252	0.851750
O	3.874440	-1.043021	1.556083
H	-2.550016	0.878752	2.409851
H	-0.959905	0.607441	-2.290483
H	-4.171959	0.967074	0.523195
H	0.678618	0.535099	-0.428943
H	0.782025	1.003441	4.094277
H	1.721327	-0.513198	2.908376
H	1.250954	-0.886621	1.266595
H	5.484977	3.053434	0.263574
H	1.331644	2.651726	1.350713
H	4.782777	-1.344094	1.412769
I	3.088000	5.049947	0.393870
H	5.825995	0.639111	0.715171

Geometries of Species D

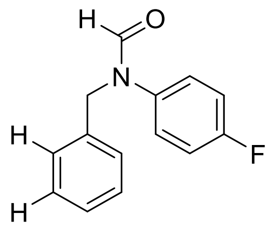

1-F (**D**)

C	2.955985	-0.710964	0.259953
C	2.301859	2.006530	0.129433
C	3.953826	0.237260	0.057555
C	1.619025	-0.309145	0.394248
C	1.304304	1.054771	0.326279
C	3.611387	1.579607	-0.005034
F	4.583161	2.496655	-0.198018
N	0.586592	-1.257850	0.640451
C	0.636368	-2.557038	0.193548
O	1.534005	-3.070993	-0.437549
C	-0.653791	-0.815621	1.259740
C	-1.680886	-0.272754	0.285820
C	-3.677177	0.752759	-1.418526
C	-2.851422	0.271491	0.819051
C	-1.522433	-0.297933	-1.100589
C	-2.507876	0.208283	-1.947940
C	-3.821332	0.772452	-0.043682
H	3.214715	-1.762327	0.310147
H	2.067588	3.067247	0.075002
H	4.995450	-0.059161	-0.045535
H	0.274858	1.392875	0.413803
H	-0.275458	-3.117133	0.479159
H	-1.088976	-1.664264	1.802091
H	-0.420604	-0.060369	2.018723
H	-4.466868	1.158637	-2.047133
H	-0.607467	-0.709963	-1.523945
H	-2.364203	0.182051	-3.025681
H	-4.720283	1.192955	0.421379
H	-2.980350	0.291943	1.907250

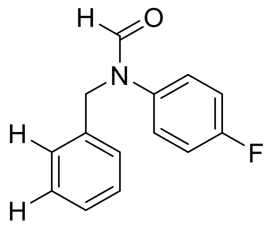

1-Br (**D**)

C	2.996399	-0.365674	0.448775
C	2.068760	2.255988	0.142503
C	3.899666	0.674365	0.257341
C	1.618597	-0.105213	0.483771
C	1.166162	1.211476	0.328623
C	3.422275	1.967462	0.106713
F	4.303157	2.973886	-0.075870
N	0.681359	-1.150806	0.718143
C	0.896941	-2.452452	0.331623
O	1.875103	-2.884049	-0.238822
C	-0.629838	-0.824828	1.258039
C	-1.659546	-0.446255	0.211654
C	-3.656788	0.269286	-1.624318
C	-2.912236	-0.012360	0.671688
C	-1.423420	-0.516171	-1.157742
C	-2.414012	-0.162943	-2.074921
C	-3.894095	0.342069	-0.254689
H	3.360051	-1.380015	0.567228
H	1.727752	3.281752	0.021486
H	4.971228	0.488952	0.230492
H	0.102851	1.437122	0.339250
H	0.041366	-3.101126	0.603733
H	-0.995698	-1.691205	1.823116
H	-0.520920	-0.014066	1.987016
H	-4.441707	0.553152	-2.321816
H	-0.446803	-0.841568	-1.514278
H	-2.216639	-0.220389	-3.143081
H	-3.054635	0.025025	1.757755
H	-4.865330	0.688584	0.116589

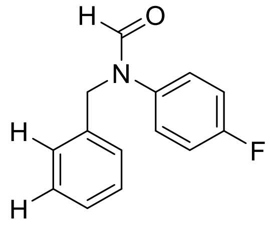

1-CF_3 (**D**)

C	-2.990544	-0.405306	0.474217
C	-2.171834	-0.204763	-2.192903
C	-3.935049	-0.235441	-0.532785
C	-1.625666	-0.469799	0.158029
C	-1.227828	-0.368362	-1.181061
C	-3.511042	-0.136783	-1.849166
N	-0.649650	-0.675212	1.173020
C	0.642395	-1.244152	0.820774
C	1.674857	-0.222771	0.387473
C	3.676112	1.578913	-0.397179
C	2.946773	-0.691804	0.031488
C	1.427244	1.145819	0.345788
C	2.417674	2.048895	-0.044914
C	3.943122	0.209866	-0.359309
C	-0.802068	-0.221634	2.462583
O	-1.752306	0.384058	2.907317
F	-4.430211	0.027365	-2.823507
H	-3.313603	-0.482893	1.506431
H	-1.873350	-0.124807	-3.235943
H	-4.997239	-0.185051	-0.303145
H	-0.175232	-0.404750	-1.451839
H	1.028516	-1.792669	1.688855
H	0.496864	-1.993632	0.034218
H	4.462473	2.264990	-0.704142
H	0.437659	1.513193	0.617526
H	2.204767	3.115110	-0.072688
H	0.077396	-0.475012	3.086710
H	3.087730	-1.777329	0.086117
H	4.932038	-0.176037	-0.631966

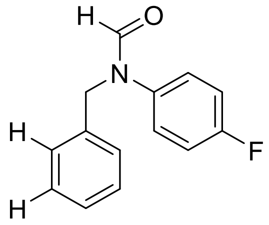

1-Me (**D**)

C	2.949246	-0.752586	0.272852
C	2.326284	1.974318	0.245822
C	3.960788	0.191577	0.125717
C	1.614261	-0.342169	0.400882
C	1.315184	1.026554	0.386339
C	3.633272	1.538937	0.113245
N	0.568471	-1.289108	0.584128
F	4.617384	2.452646	-0.030708
C	0.607621	-2.559147	0.060868
O	1.508326	-3.048730	-0.586170
C	-0.677841	-0.865275	1.208812
C	-1.689045	-0.269548	0.250030
C	-3.646050	0.846921	-1.400194
C	-2.884540	0.224530	0.783202
C	-1.491107	-0.197575	-1.127142
C	-2.465363	0.359436	-1.954297
C	-3.878525	0.789253	-0.024086
H	3.196032	-1.808099	0.282836
H	2.104455	3.039089	0.231396
H	5.000743	-0.112171	0.026449
H	0.286945	1.369703	0.469359
H	-0.315700	-3.122032	0.299392
H	-1.124649	-1.733978	1.708345
H	-0.444852	-0.149641	2.005463
H	-4.408927	1.283807	-2.044811
H	-0.560935	-0.569827	-1.555491
H	-2.301429	0.414615	-3.028542
H	-2.996378	0.141425	1.870310
H	-4.803671	1.167072	0.425995

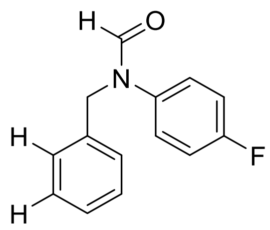

1-Cl (**D**)

C	2.984192	-0.553832	0.349415
C	2.218543	2.129305	0.148630
C	3.946429	0.432655	0.158920
C	1.627630	-0.208325	0.436155
C	1.256587	1.138759	0.333476
C	3.548898	1.757280	0.060829
F	4.485913	2.711980	-0.121157
N	0.632105	-1.198526	0.669267
C	0.751355	-2.499886	0.241659
O	1.685025	-2.980129	-0.363117
C	-0.641827	-0.806978	1.253771
C	-1.676971	-0.346394	0.246490
C	-3.690590	0.509522	-1.513611
C	-2.906684	0.101166	0.749322
C	-1.470608	-0.358097	-1.129982
C	-2.468361	0.066350	-2.008664
C	-3.897474	0.523208	-0.138400
H	3.286197	-1.592045	0.426724
H	1.940736	3.177766	0.067517
H	5.002435	0.180477	0.092352
H	0.211048	1.432177	0.384588
H	-0.140096	-3.097396	0.516048
H	-1.043415	-1.662196	1.811243
H	-0.464164	-0.021111	1.996413
H	-4.484306	0.844748	-2.177621
H	-0.512149	-0.695752	-1.522159
H	-2.292648	0.053217	-3.081990
H	-3.035856	0.094905	1.837666
H	-4.851944	0.867151	0.276216

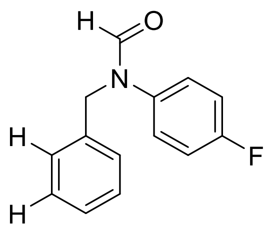

1-CN (**D**)

C	-2.315655	0.771237	1.619921
C	-1.558806	0.970560	-1.065870
C	-3.280361	0.968354	0.637054
C	-0.960883	0.677391	1.269553
C	-0.594394	0.779304	-0.078617
C	-2.887191	1.065600	-0.688959
F	-3.826084	1.253466	-1.639512
N	0.036909	0.438796	2.256663
C	-0.075740	0.866056	3.559652
O	-1.003924	1.476412	4.042063
C	1.308172	-0.145795	1.859866
C	2.340861	0.864034	1.400030
C	4.343080	2.651756	0.548554
C	3.577674	0.377188	0.956255
C	2.129940	2.238693	1.408555
C	3.119979	3.133585	0.987086
C	4.574045	1.269034	0.531778
H	-2.615360	0.693854	2.658992
H	-1.284390	1.049997	-2.115470
H	-4.335153	1.040349	0.893317
H	0.449287	0.720199	-0.377796
H	0.815411	0.585643	4.155020
H	1.714049	-0.705941	2.711407
H	1.128803	-0.885929	1.071753
H	5.129334	3.325315	0.214904
H	1.166485	2.622426	1.743785
H	2.928261	4.203928	1.002518
H	3.716372	-0.709982	0.962577
H	5.523277	0.839032	0.192260

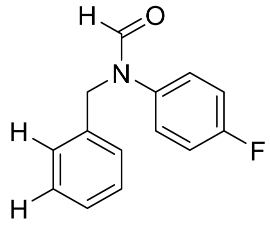

1-NO_2 (**D**)

C	-2.482661	0.773514	1.616948
C	-1.704986	0.856094	-1.069401
C	-3.441209	0.911955	0.618938
C	-1.123924	0.682687	1.281649
C	-0.746795	0.723741	-0.066732
C	-3.037491	0.952892	-0.707290
N	-0.133206	0.507774	2.289486
C	1.143748	-0.094650	1.941786
C	2.182315	0.891884	1.443706
C	4.195815	2.630609	0.538577
C	3.431576	0.362521	1.062266
C	1.966410	2.258806	1.364460
C	2.965731	3.133823	0.913492
C	4.424482	1.251291	0.612601
F	-3.970271	1.088582	-1.672482
C	-0.261021	1.017190	3.561187
O	-1.193887	1.659795	3.989741
H	-2.789299	0.741774	2.656185
H	-1.423075	0.889220	-2.119233
H	-4.498981	0.982232	0.862565
H	0.300321	0.661816	-0.352927
H	1.540035	-0.607707	2.826786
H	0.978098	-0.876800	1.192649
H	4.998437	3.271710	0.184826
H	0.994061	2.658227	1.652995
H	2.772491	4.202221	0.859643
H	0.621098	0.772593	4.185161
H	3.551004	-0.723821	1.144808
H	5.408755	0.877871	0.307673

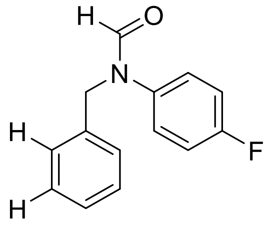

1-OMe (**D**)

C	3.013413	-0.341363	0.373592
C	2.031401	2.273342	0.224972
C	3.894098	0.725429	0.226748
C	1.631682	-0.111303	0.441580
C	1.151571	1.202315	0.365760
C	3.389330	2.014670	0.154012
F	4.247934	3.047574	0.013907
N	0.718910	-1.186565	0.628901
C	0.944739	-2.452117	0.143837
O	1.917671	-2.825939	-0.475262
C	-0.587393	-0.918733	1.214763
C	-1.643565	-0.486722	0.218307
C	-3.704408	0.319231	-1.517269
C	-2.881153	-0.098747	0.720456
C	-1.440737	-0.470289	-1.165508
C	-2.460204	-0.072002	-2.022104
C	-3.911954	0.305686	-0.142254
H	3.398314	-1.353256	0.432715
H	1.669256	3.297298	0.163988
H	4.968573	0.563061	0.174617
H	0.083827	1.403927	0.402633
H	0.104826	-3.132130	0.385368
H	-0.926420	-1.828263	1.726542
H	-0.473460	-0.158577	1.996071
H	-4.494358	0.630491	-2.195706
H	-0.471883	-0.760164	-1.569955
H	-2.292456	-0.059827	-3.097090
H	-3.039920	-0.116024	1.804758
H	-4.837636	0.588997	0.371617

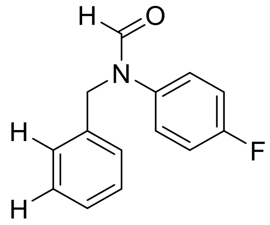

1-Ph (**D**)

C	-3.001098	-0.014303	0.519151
C	-1.793859	-2.475733	-0.033748
C	-3.786372	-1.125594	0.227274
C	-1.602544	-0.121515	0.530962
C	-1.009826	-1.360109	0.251891
C	-3.170918	-2.337550	-0.044891
F	-3.935368	-3.414306	-0.327915
N	-0.783538	0.993828	0.861258
C	-1.146610	2.293865	0.604461
O	-2.183567	2.670938	0.102448
C	0.574961	0.765014	1.334729
C	1.602733	0.611045	0.231276
C	3.588943	0.290203	-1.706172
C	2.910026	0.266958	0.600906
C	1.309031	0.795494	-1.116561
C	2.297277	0.632726	-2.088587
C	3.921754	0.109802	-0.358089
H	-3.474822	0.937170	0.732157
H	-1.342693	-3.441356	-0.251347
H	-4.872101	-1.057254	0.216271
H	0.071856	-1.468345	0.240433
H	-0.354947	3.002388	0.916303
H	0.579584	-0.119858	1.980627
H	0.860518	1.606107	1.978710
H	4.371828	0.175918	-2.455430
H	0.293202	1.054781	-1.413601
H	2.057474	0.776916	-3.140044
H	3.076396	0.114102	1.673367
H	4.942681	-0.161333	-0.065837

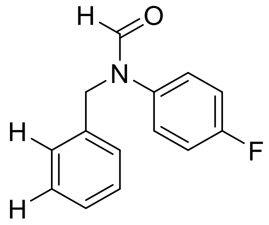

1-^{t}Bu (**D**)

C	4.023084	0.086718	0.838893
C	6.742595	0.696706	0.909533
C	4.957490	-0.940379	0.955820
C	4.437199	1.421836	0.754193
C	5.807269	1.718942	0.786228
C	6.302679	-0.615354	0.994118
N	3.471644	2.454523	0.597492
C	2.169372	2.138891	0.025386
C	1.135507	1.682472	1.035882
C	-0.866291	0.851184	2.772959
C	-0.132196	1.329661	0.552804
C	1.387961	1.614690	2.401731
C	0.385748	1.196950	3.272279
C	-1.169377	0.905767	1.405444
C	3.648198	3.720656	1.100260
O	4.614548	4.128660	1.708449
F	7.213233	-1.605388	1.116544
H	2.965126	-0.164459	0.828708
H	7.809038	0.910309	0.934987
H	4.647673	-1.980876	1.024218
H	6.140495	2.748722	0.721233
H	1.796017	3.031076	-0.492522
H	2.303689	1.378359	-0.751948
H	-1.628132	0.528206	3.476799
H	2.373481	1.879833	2.783482
H	0.578488	1.139391	4.341604
H	2.773702	4.365552	0.886186
H	-2.160059	0.632636	1.024440
H	-0.231352	1.420196	-0.534940

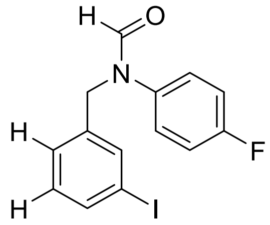

1-I$_2$ (**D**)

C	-2.194697	0.818221	1.387644
C	-1.290490	0.666289	-1.256107
C	-3.104357	0.872837	0.336710
C	-0.822407	0.694649	1.125836
C	-0.381393	0.619277	-0.201165
C	-2.638104	0.797940	-0.967185
F	-3.524112	0.854320	-1.983301
N	0.120497	0.604135	2.188510
C	-0.068807	1.201557	3.413399
O	-1.021342	1.868478	3.751221
C	1.397793	-0.055377	1.965462
C	2.504947	0.855768	1.466732
C	4.649769	2.443331	0.597007
C	3.774290	0.281320	1.284033
C	2.317447	2.210819	1.212717
C	3.386553	2.996709	0.778578
C	4.833371	1.083252	0.851750
H	-2.550016	0.878752	2.409851
H	-0.959905	0.607441	-2.290483
H	-4.171959	0.967074	0.523195
H	0.678618	0.535099	-0.428943
H	0.782025	1.003441	4.094277
H	1.721327	-0.513198	2.908376
H	1.250954	-0.886621	1.266595
H	5.484977	3.053434	0.263574
H	1.331644	2.651726	1.350713
I	3.088000	5.049947	0.393870
H	3.855255	-0.789329	1.503969
H	5.825995	0.639111	0.715171

S4.3: Summary of Computational Results and Supplementary Values for main text Figures 3 and 4.

Table S8: Numerical values for Figures 3 and 4

**1-R**	ΔE_{DFT}	$E_{HO\cdots R}$ - $E_{OH\cdots R}$	$E_{HO\cdots R}$	$E_{OH\cdots R}$	ΔG_{exp}
**1**-OMe	-3.22	-1.0	14.7	-6.5	-4.7
**1**-NO_2	-5.80	13.2	10.6	-24.7	-5.0
**1**-Br	-11.33	-10.2	4.8	-7.2	-7.8
**1**-Ph	-12.42	-13.3	3.4	-5.5	-8.2
**1**-Cl	-11.01	-9.6	6.6	-6.0	-8.3
**1**-F	-13.47	-10.7	12.5	1.0	-9.1
**1**-I_2	-16.30	-12.5	1.9	-7.7	-10.7
**1**-CN	-15.19	-12.6	-1.1	-10.6	-12.0
**1**-Me	-23.43	-23.0	-3.4	-2.6	-13.0
**1**-CF_3	-19.10	-17.2	2.7	-2.3	-14.5
**1**-^{t}Bu	-27.44	-25.0	3.3	6.1	-15.4
**1**-H	-22.2	-22.2	0	0	-11.6
**1**-control	-5.80	-5.8	0	0	-5.7

S4.4: SAPT calculations of the OH···O=CH hydrogen bond energy in balances **1**-R

I-SAPT(0)/jun-cc-PVDZ calculations were run using Psi4[8] Python API. I-SAPT allows intramolecular systems to be dissected into chemically meaningful fragments, and to evaluate the energy of their interaction.[9] In the case of the **1**-R series, the interacting fragments were defined to be the phenolic OH and formamide CHO (Figure S7). When used alone, the I-SAPT values of the CHO···HO H-bond strength cannot describe the experimental behaviour of these systems and is remarkably poorly correlated (R^2 = 0.17, Figure S8) this variation of 6 kJ/mol is small compared to the variance of the relative energies of the *closed* formyl conformer in which the CHO···HO hydrogen bond was also formed determined in the earlier DFT analysis (−27 to +14 kJ/mol). This means that while the substituent effect on the CHO···HO H-bond strength exists, the direct interactions between the OH and the *ortho*-substituent R make a larger contribution. For example, I-SAPT indicates that the *ortho*-nitrophenol in the closed-bonded conformation of compound **1**-NO_2 hosts the strongest CHO···HO H-bond, but in the NMR experiment this conformation is not observed. On the other side of the spectrum, *ortho*-cresol (compound **1**-CH_3) forms the weakest H-bond in the closed conformer, but this conformer is exceedingly dominant in the NMR experiment.

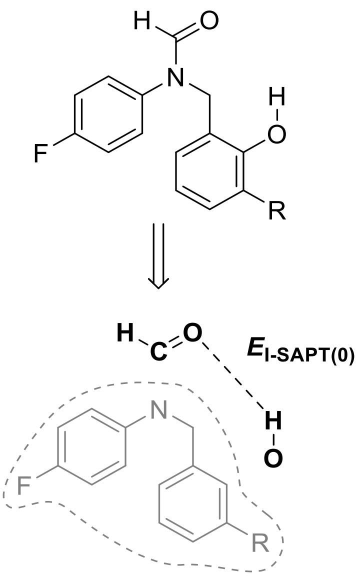

Figure S7: Fragments defined in the I-SAPT partitioning algorithm

Table S9: I-SAPT(0) decomposition of OH···OCH interaction energy (kJ/mol)

**1**-R	ELST	EXCH	IND	DISP	**TOT**
NO_2	-55.69	62.43	-23.33	-9.51	**-26.11**
CF_3	-52.77	60.10	-22.22	-9.29	**-24.18**
Br	-51.93	58.91	-21.65	-9.15	**-23.82**
Cl	-51.19	59.04	-21.52	-9.15	**-22.83**
F	-50.18	57.03	-20.79	-8.99	**-22.93**
OMe	-47.67	53.73	-19.52	-8.71	**-22.16**
Ph	-50.79	57.49	-20.99	-9.03	**-23.32**
H	-47.39	54.83	-19.57	-8.69	**-20.82**
Me	-47.52	55.84	-19.92	-8.80	**-20.40**
^{t}Bu	-50.78	59.24	-21.33	-9.19	**-22.06**

ELST: Electrostatic energy, EXCH: Exchange energy;
IND: induction energy; DISP: dispersion energy;
TOT: Total I-SAPT(0) interaction energy (sum of above contributions)

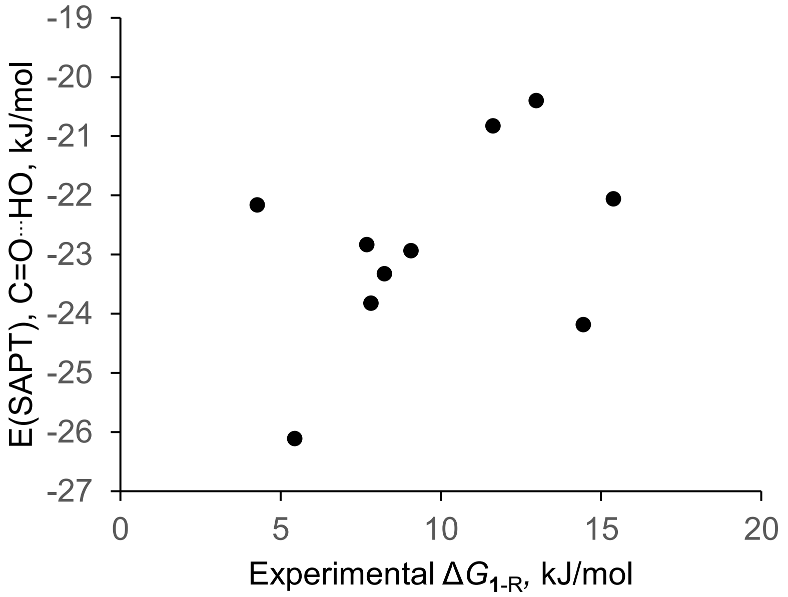

Figure S8: Absence of correlation between the observed conformer ratio in balances **1-R** and the calculated energies of CHO···HO hydrogen bonds.

S5: Compound synthesis and characterization

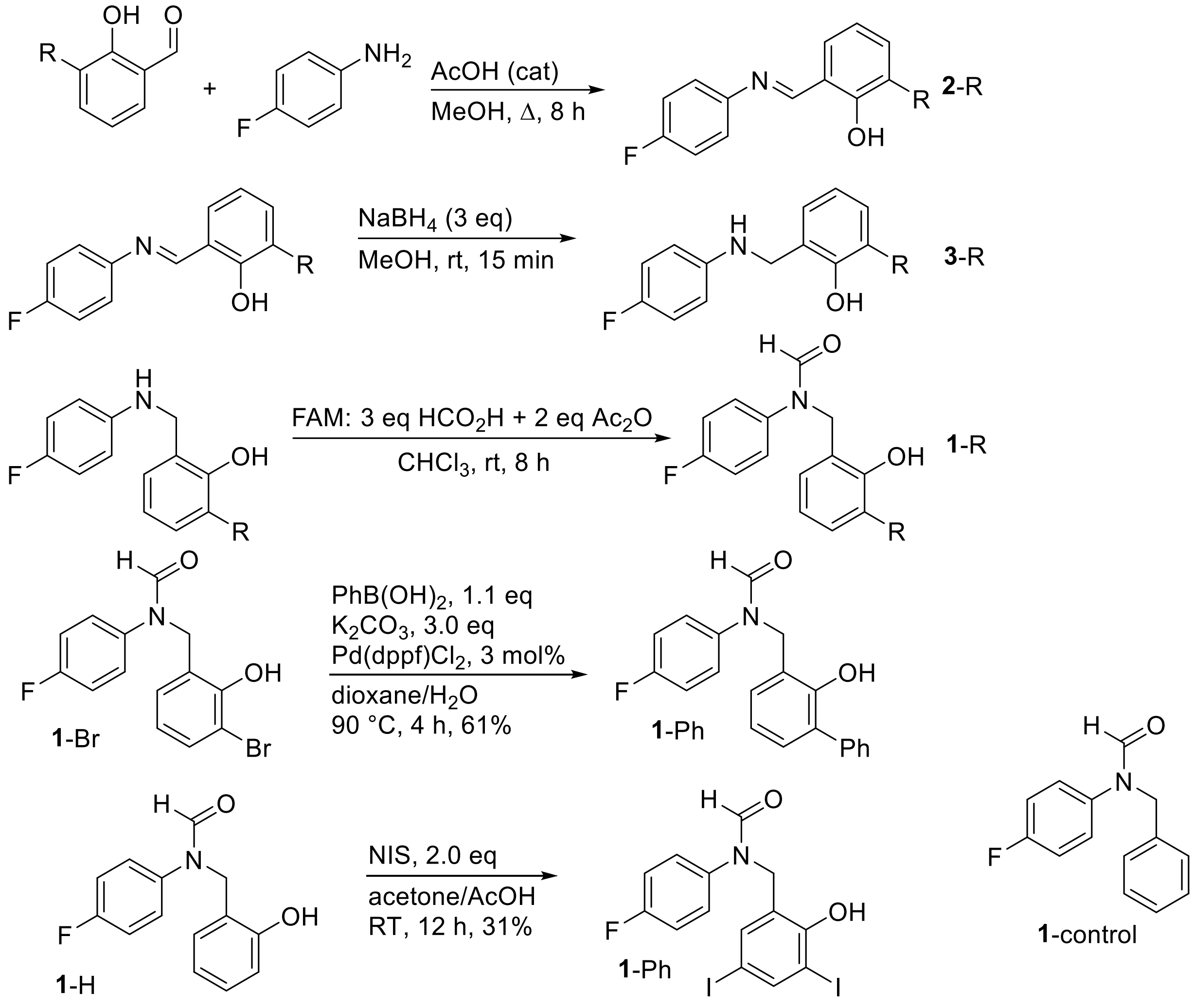

Synthesis of **1**-R commenced with preparation of imines **2-**R using 3-substituted salicylaldehydes and 4-fluoroaniline as the starting materials. The latter were isolated by recrystallisation from methanol. The subsequent reduction of isolated **2-**R with $NaBH_4$ allowed for a clean formation of amines **3**-R without concomitant over-amination which otherwise was a noticeable side-reaction if the reductive amination of 3-R-salicylaldehydes was carried out in a single one-pot step. The final formylation of **3**-R with a mixture of formic acid and acetic anhydride (FAM) produced the target balances **1**-R in 72-91% yield. The control molecular balance **1**-control, which lacks an OH group was synthesized identically using benzaldehyde as the starting material. Additionally, the synthesis of the remaining compound **1**-Ph was accomplished in a single step through a Suzuki cross-coupling reaction, utilizing balance **1**-Br and phenylboronic acid. A di-iodinated compound **1**-I_2 was isolated after iodination of balance **1**-H with NIS.

General procedure A: Synthesis of imines **2**-R

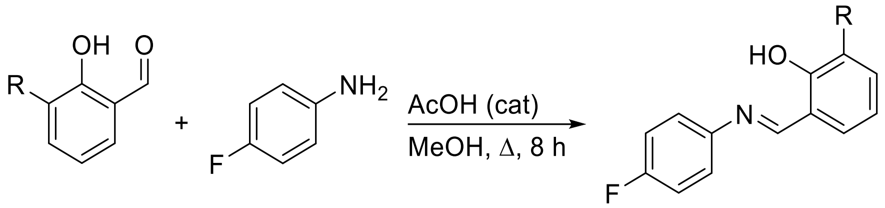

In a 25 mL flask, the corresponding aldehyde (1.0 eq, 5 mmol) and 4-fluoroaniline (1.2 eq, 6 mmol) were dissolved in 10 mL methanol. Two droplets of acetic acid were added and the reaction mixture was refluxed overnight. TLC (100% DCM) analysis was used to confirm the full consumption of the aldehyde. The reaction was allowed to cool down to room temperature. For all compounds except **2-**Me and **2-**^{t}Bu (see below), the flask was immersed in an ice bath for 3 h to ensure complete precipitation of the desired product. The precipitate was collected by filtration and washed with cold methanol (2 x 5 mL) to provide the corresponding imines **2-**R. In case of **2-**Me and **2-**^{t}Bu, the rection mixture was concentrated under reduced pressure, and to the residual crude product was added 100 μL acetic acid. The resulting mixture was passed through a 10 cm silica plug (eluting with 100% DCM).

2-{(1*E*)-[(4-fluorophenyl)imino]methyl}-6-methoxyphenol (2-OMe)

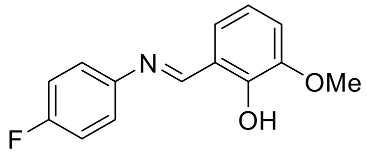

Following general procedure A, 3-methoxy-2-hydroxybenzaldehyde (5 mmol, 760 mg, 1.0 eq), 4-fluoroaniline (6 mmol, 667 mg, 1.2 eq) were dissolved in 10 mL MeOH, two droplets of acetic acid were added and the reaction mixture was refluxed for 8 h. Upon cooling down, the red precipitate was formed, which was collected by filtration and washed with cold methanol to provide the title compound as a red solid (932 mg, 76%). Mp 92 – 95 °C. Spectroscopic data matches literature values.[10] ^{1}H NMR (400 MHz, $CDCl_3$) δ 13.48 (s, 1H), 8.59 (s, 1H), 7.31 – 7.23 (m, 2H), 7.16 – 7.06 (m, 2H), 7.02 (dd, J = 7.6, 1.5 Hz, 1H), 7.00 (dd, J = 7.6, 1.5 Hz, 1H), 6.89 (t, J = 7.9 Hz, 1H), 3.94 (s, 3H). ^{13}C NMR (151 MHz, $CDCl_3$) δ 162.56, 161.84 (d, J = 246.5 Hz), 151.40, 148.62, 144.52 (d, J = 3.3 Hz), 123.91, 122.75 (d, J = 8.4 Hz), 119.17, 118.78, 116.36 (d, J = 22.9 Hz), 115.00, 56.35. ^{19}F NMR (471 MHz, $CDCl_3$) δ -115.39 (ddd, J = 13.1, 8.3, 4.9 Hz). HRMS (ESI+): m/z calcd. for $C_{14}H_{12}FNO_2Na$ $[M+Na]^+$: 268.0744, found 268.0715.

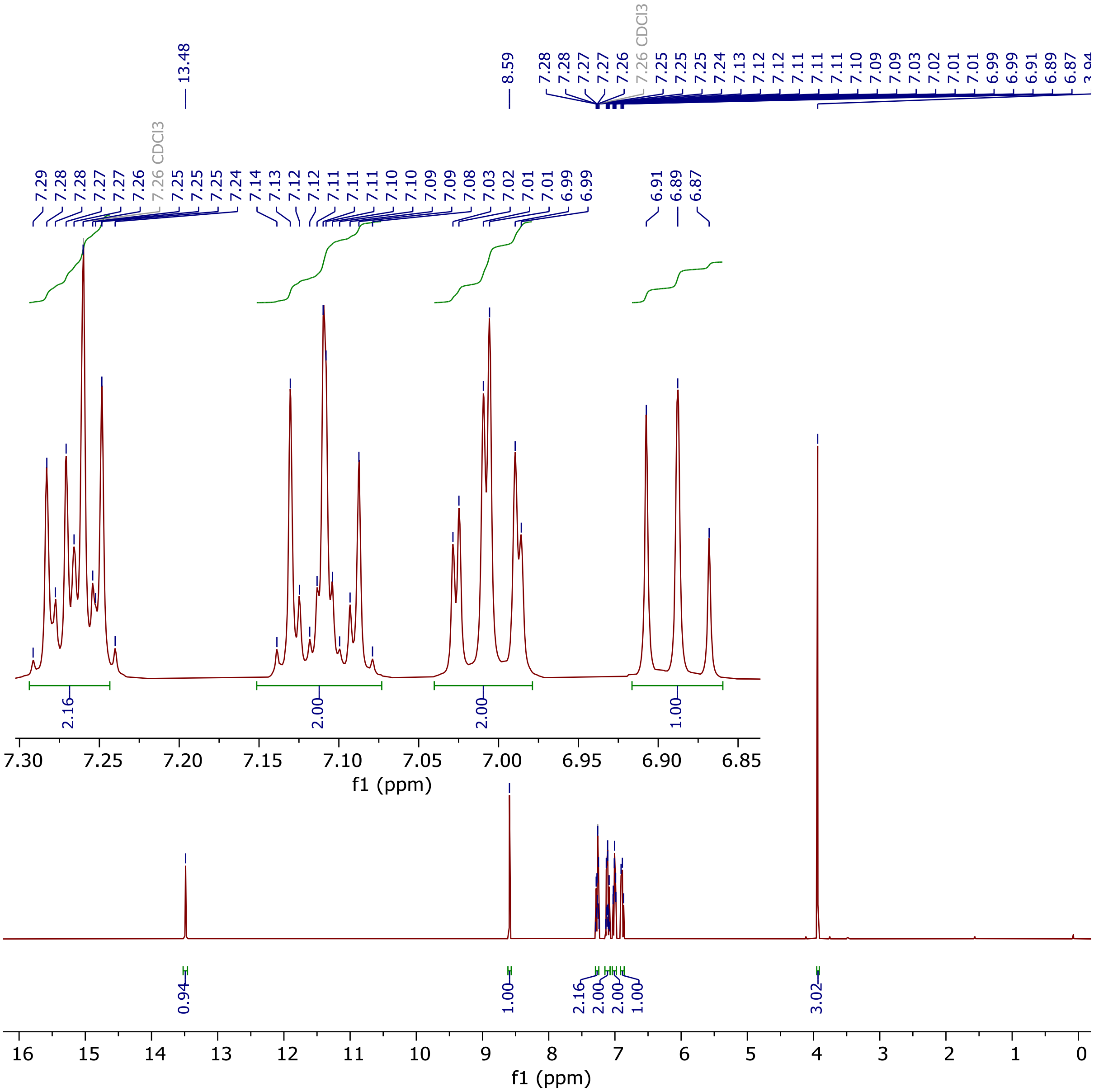

Figure S9: ^{1}H NMR spectrum of compound **2**-OMe in $CDCl_3$.

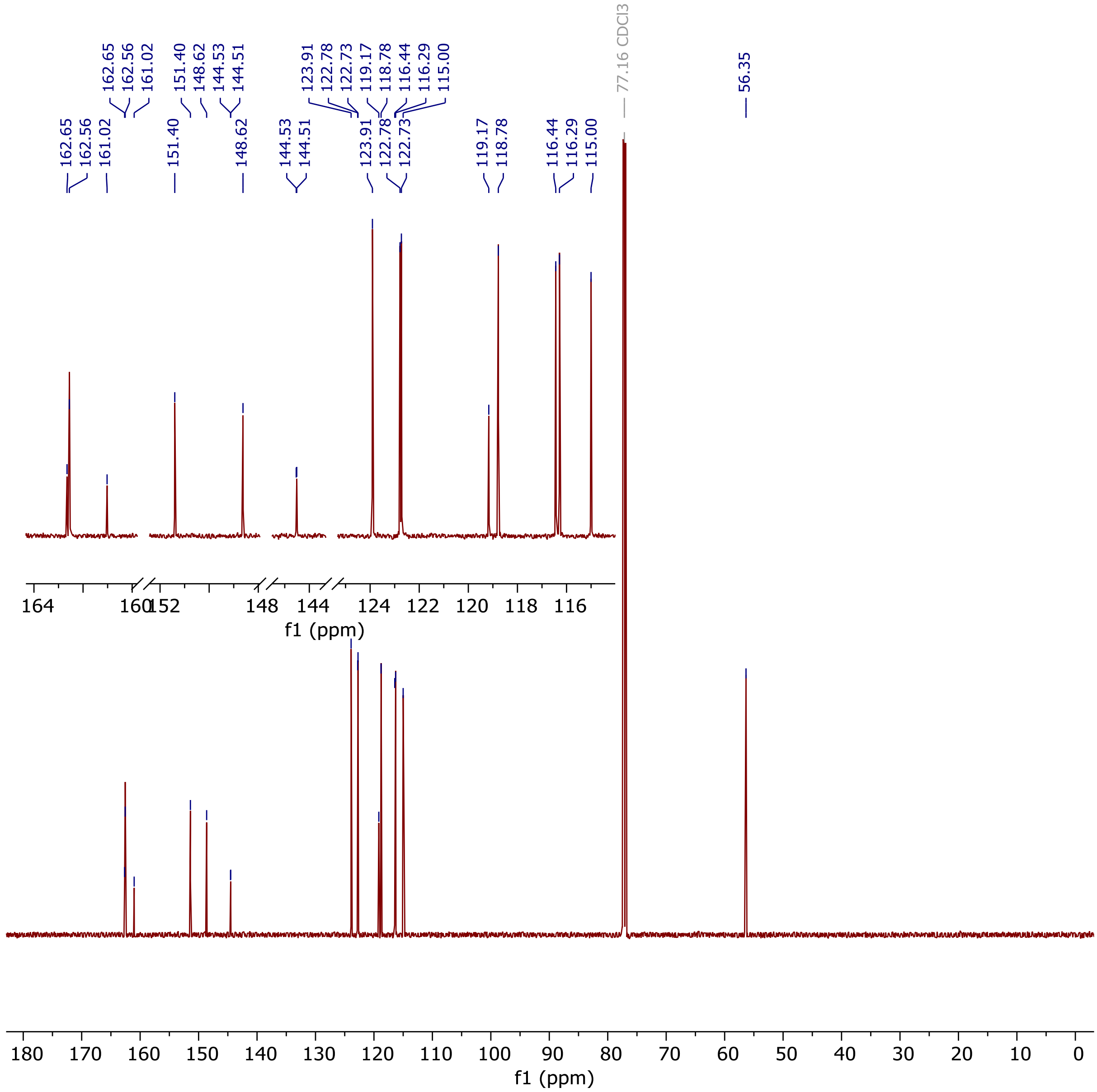

Figure S10: ^{13}C NMR spectrum of compound **2**-OMe in $CDCl_3$.

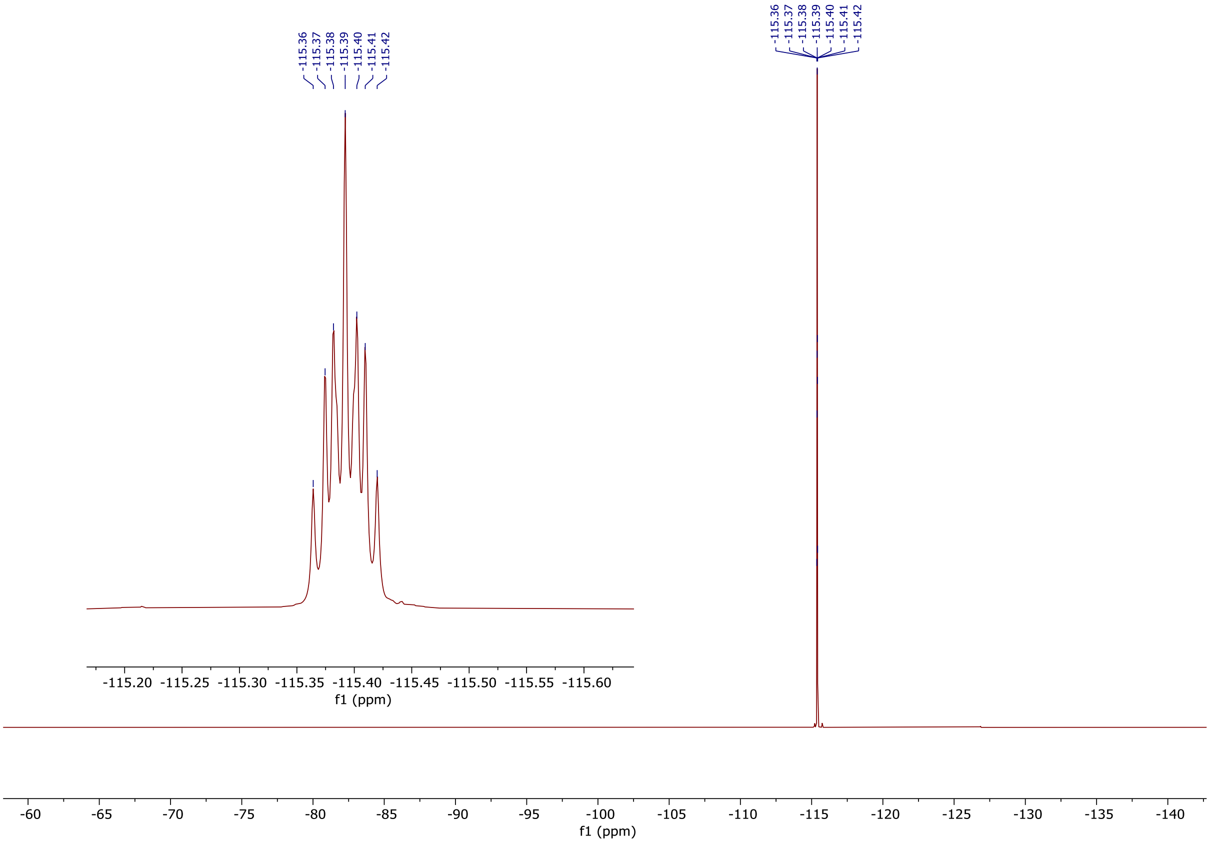

Figure S11: ^{19}F NMR spectrum of compound **2**-OMe in $CDCl_3$.

3-{(1*E*)-[(4-fluorophenyl)imino]methyl}-2-hydroxybenzonitrile (2-CN)

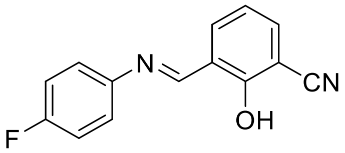

Following general procedure A, 3-formyl-2-hydroxybenzonitrile (5 mmol, 735 mg, 1.0 eq), 4-fluoroaniline (6 mmol, 667 mg, 1.2 eq) were dissolved in 10 mL MeOH. Two droplets of acetic acid were added and the reaction mixture was refluxed for 8 h. Upon cooling down, the orange precipitate was formed, which was collected by filtration and washed with cold methanol to provide the title compound as an orange solid (960 mg, 80%). Mp 144 – 146 °C. ^{1}H NMR (400 MHz, $CDCl_3$) δ 14.45 (s, 1H), 8.63 (s, 1H), 7.65 (dd, J = 7.8, 1.6 Hz, 1H), 7.61 (dd, J = 7.7, 1.7 Hz, 1H), 7.34 – 7.28 (m, 2H), 7.20 – 7.10 (m, 2H), 7.02 (t, J = 7.7 Hz, 1H). ^{13}C NMR (151 MHz, $CDCl_3$) δ 163.67, 162.38 (d, J = 248.5 Hz), 160.69, 143.16 (d, J = 3.2 Hz), 136.59, 136.46, 122.95 (d, J = 8.4 Hz), 119.72, 119.25, 116.72 (d, J = 22.8 Hz), 115.88, 101.98. ^{19}F NMR (471 MHz, $CDCl_3$) δ -113.58 (ddd, J = 12.9, 8.2, 4.8 Hz). HRMS (ESI+): m/z calcd. for $C_{14}H_9FN_2ONa$ [M+Na]$^+$: 263.0591, found 263.0623.

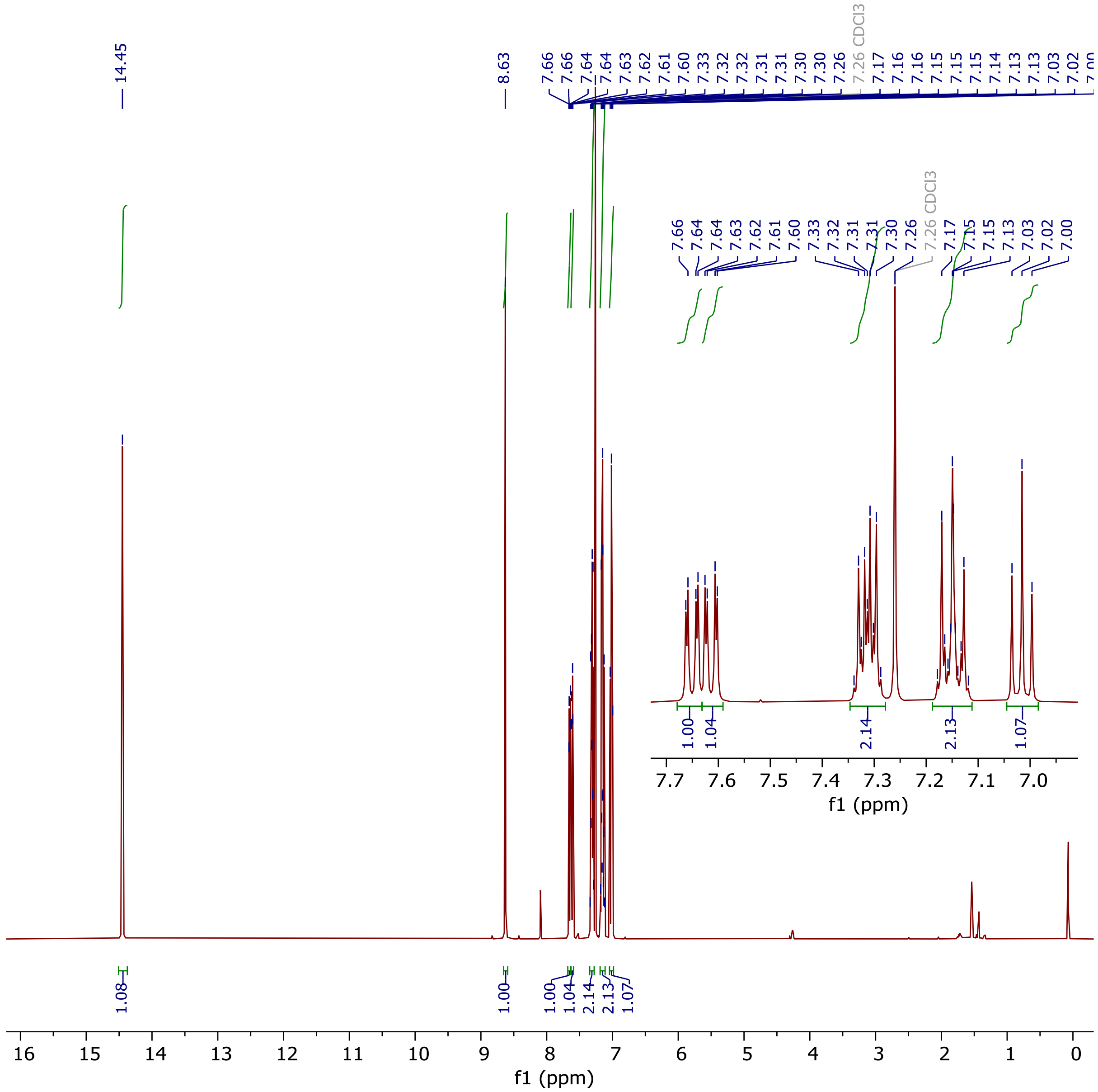

Figure S12: ^{1}H NMR spectrum of compound **2**-CN in $CDCl_3$.

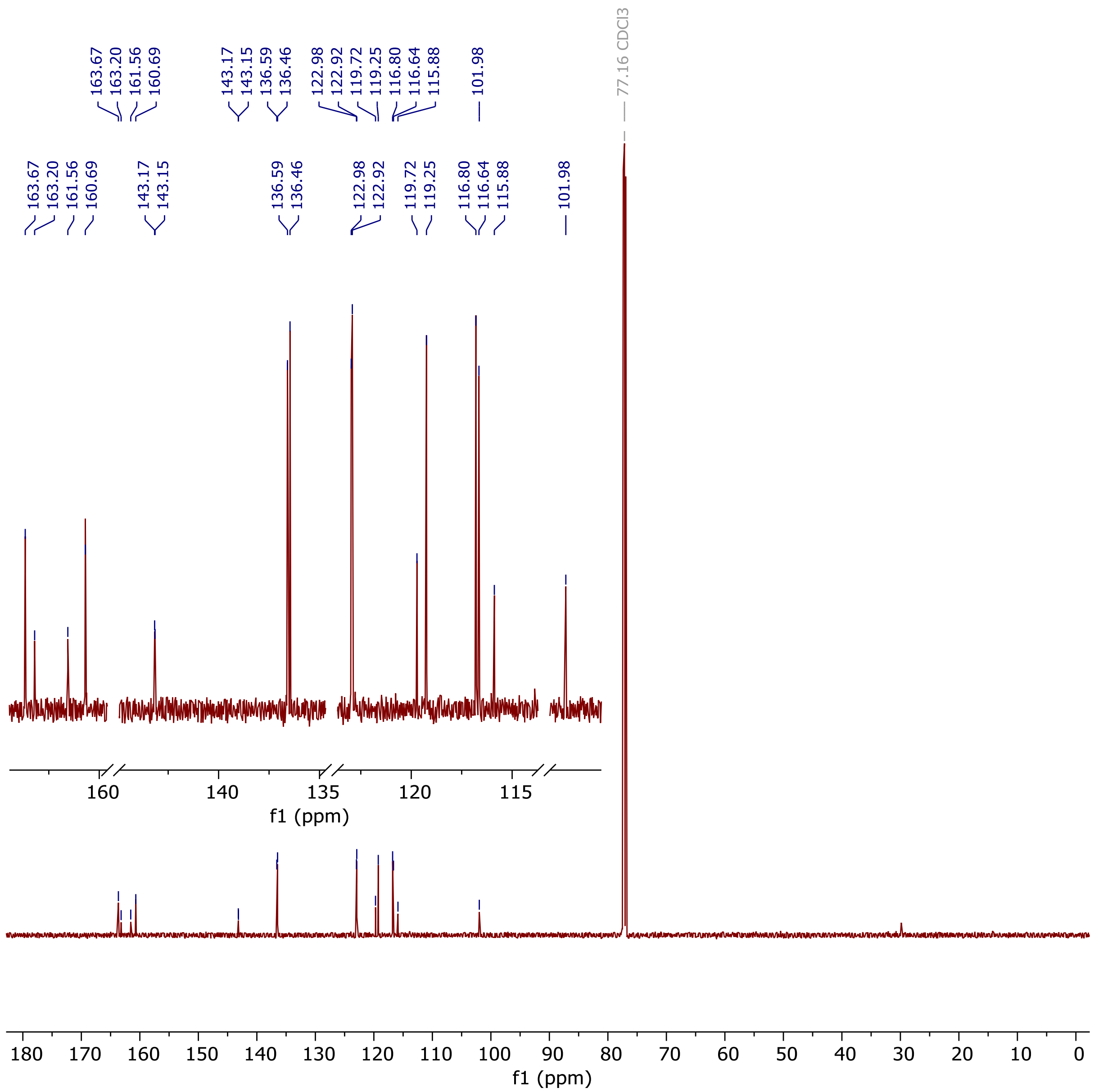

Figure S13: ^{13}C NMR spectrum of compound **2**-CN in $CDCl_3$.

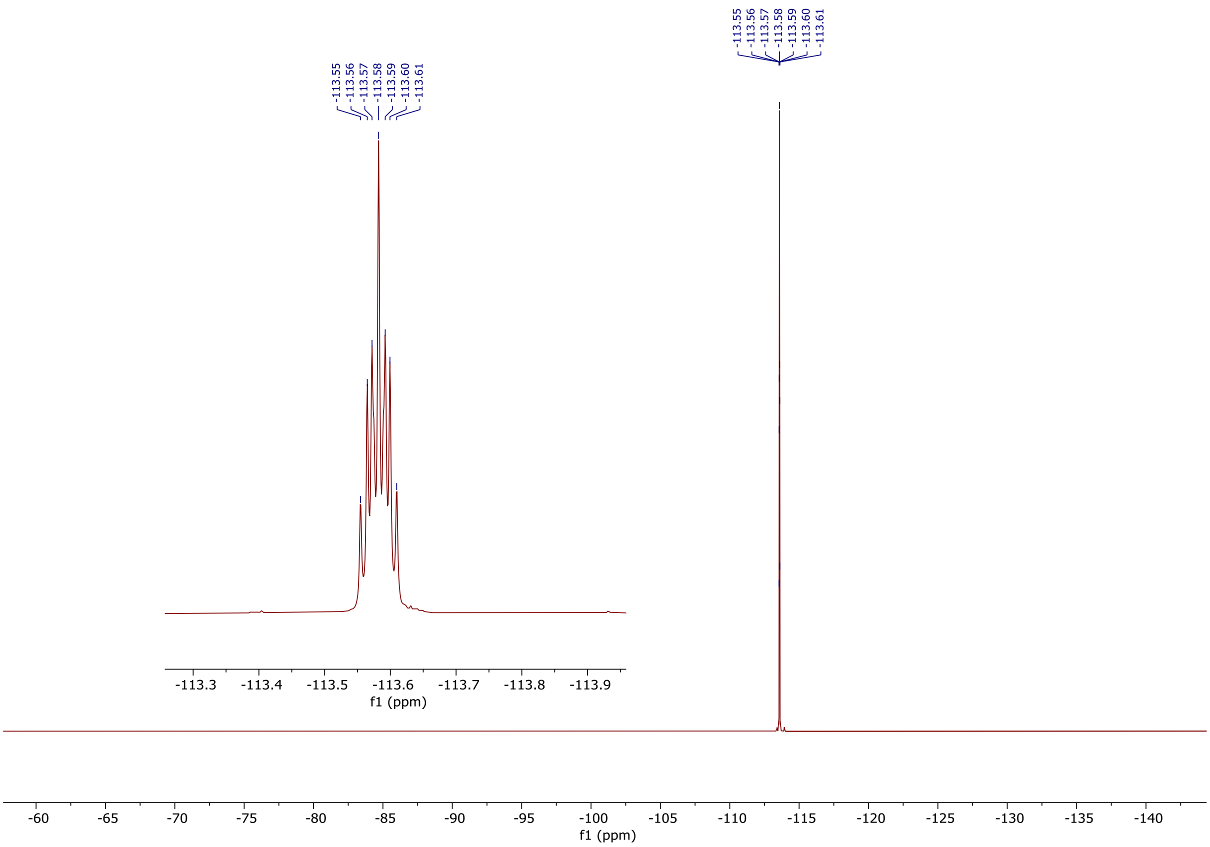

Figure S14: ^{19}F NMR spectrum of compound **2**-CN in $CDCl_3$.

2-{(1*E*)-[(4-fluorophenyl)imino]methyl}-6-(trifluoromethyl)phenol (2-CF$_3$)

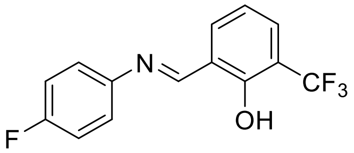

Following general procedure A, 2-hydroxy-3-(trifluoromethyl)benzaldehyde (5 mmol, 950 mg, 1.0 eq), 4-fluoroaniline (6 mmol, 667 mg, 1.2 eq) were dissolved in 10 mL MeOH. Two droplets of acetic acid were added and the reaction mixture was refluxed for 8 h. Upon cooling down, the yellow precipitate was formed, which was collected by filtration and washed with cold methanol to provide the title compound as a yellow solid (1.06 g, 75%). Mp 99 – 101 °C. ^{1}H NMR (400 MHz, $CDCl_3$) δ 14.34 (s, 1H), 8.65 (s, 1H), 7.68 (d, *J* = 7.9 Hz, 1H), 7.57 (dd, *J* = 7.7, 1.7 Hz, 1H), 7.35 – 7.25 (m, 2H), 7.19 – 7.08 (m, 2H), 7.01 (td, *J* = 7.7, 0.9 Hz, 1H). ^{13}C NMR (151 MHz, $CDCl_3$) δ 161.39 (d, *J* = 492.3 Hz), 161.39, 161.37, 143.56 (d, *J* = 3.0 Hz), 135.87, 130.48 (q, *J* = 4.8 Hz), 123.69 (q, *J* = 272.5 Hz), 122.84 (d, *J* = 8.5 Hz), 119.96, 118.43 (q, *J* = 31.2 Hz), 118.35, 116.62 (d, *J* = 22.7 Hz). ^{19}F NMR (471 MHz, $CDCl_3$) δ -62.79, -114.22 (ddd, *J* = 13.0, 8.2, 4.8 Hz). HRMS (ESI+): m/z calcd. for $C_{14}H_9F_4NONa$ [M+Na]$^+$: 306.0512, found 306.0490.

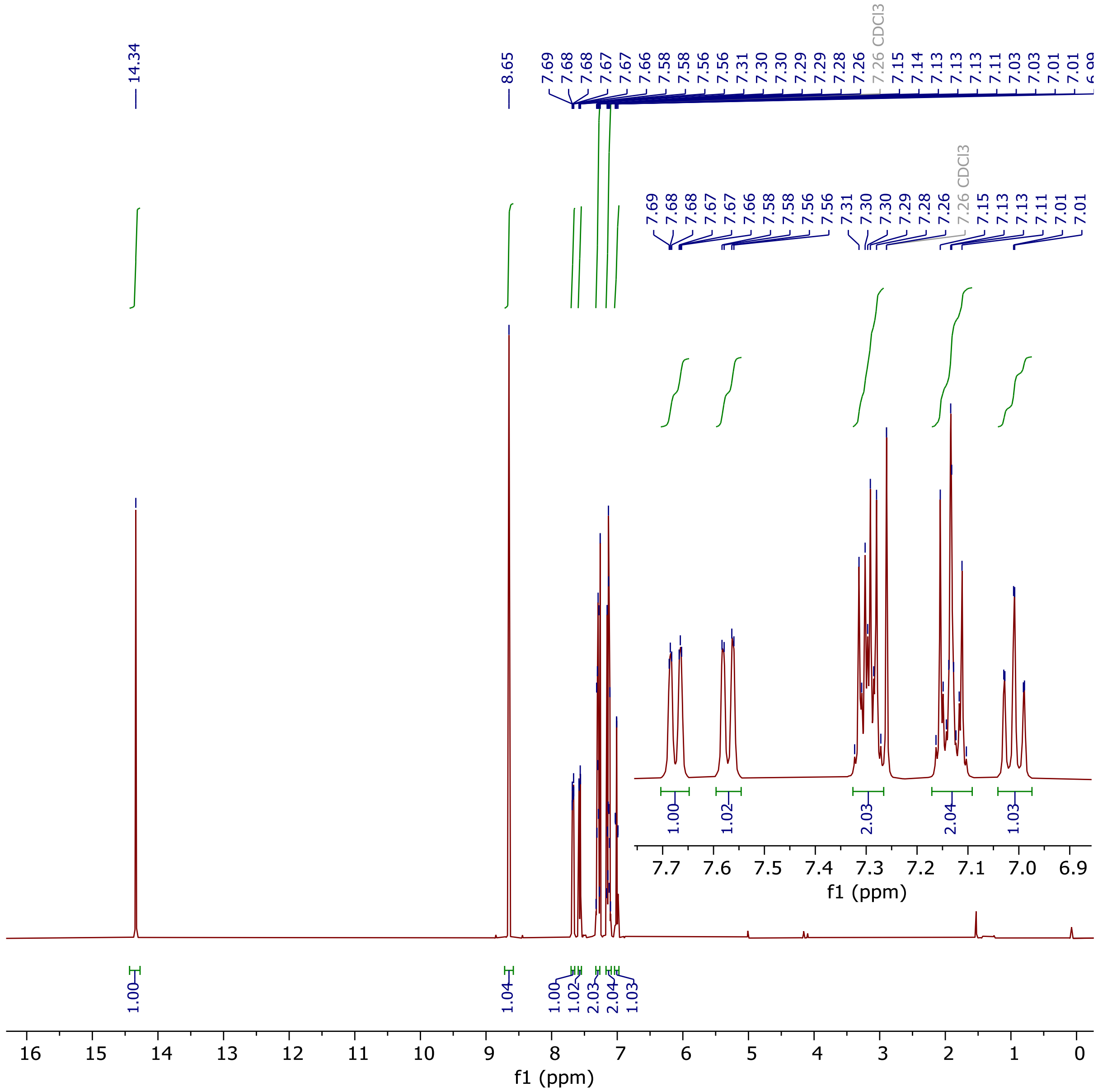

Figure S15: ^{1}H NMR spectrum of compound **2**-CF_3 in $CDCl_3$.

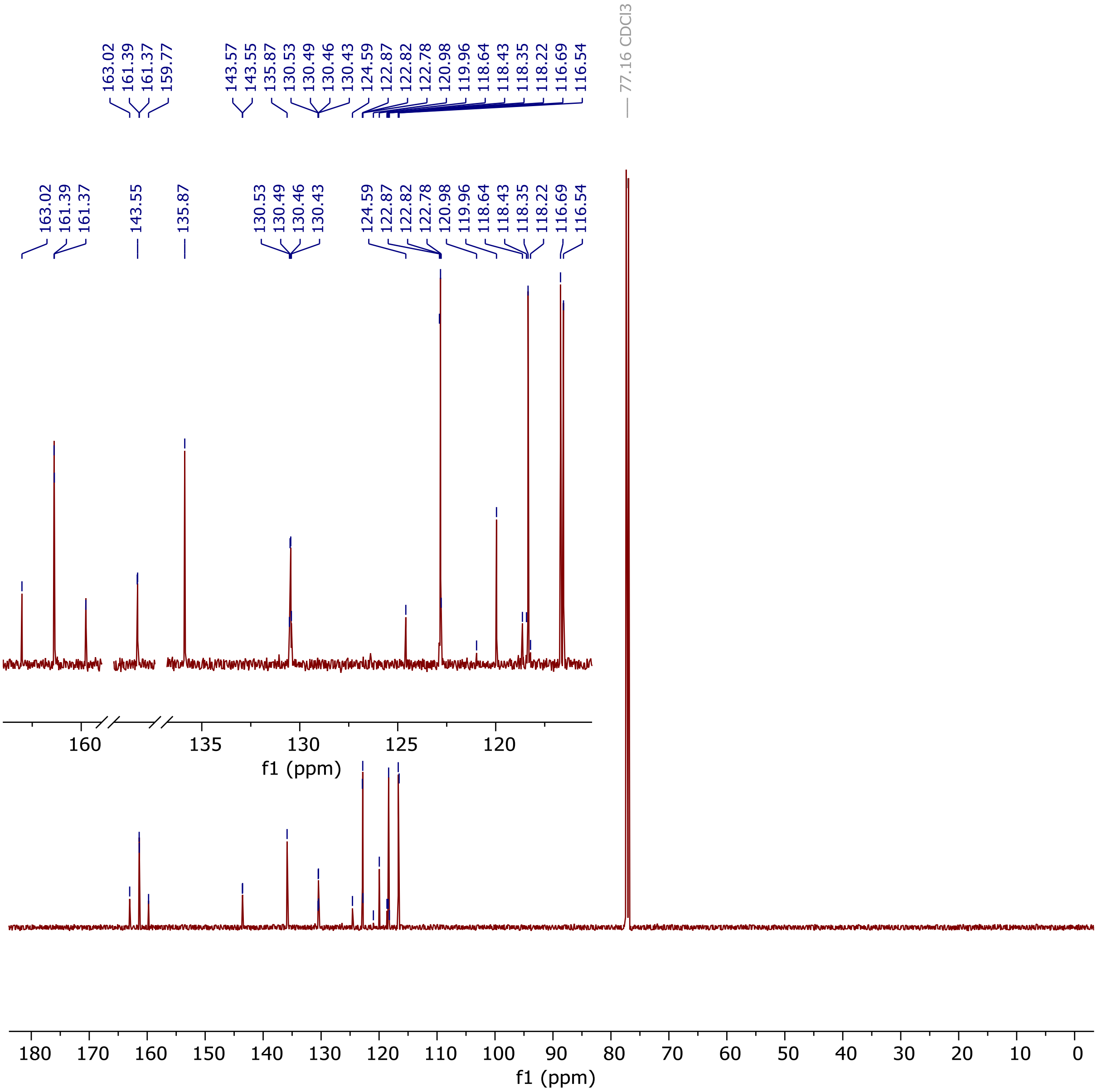

Figure S16: ^{13}C NMR spectrum of compound **2**-CF_3 in $CDCl_3$.

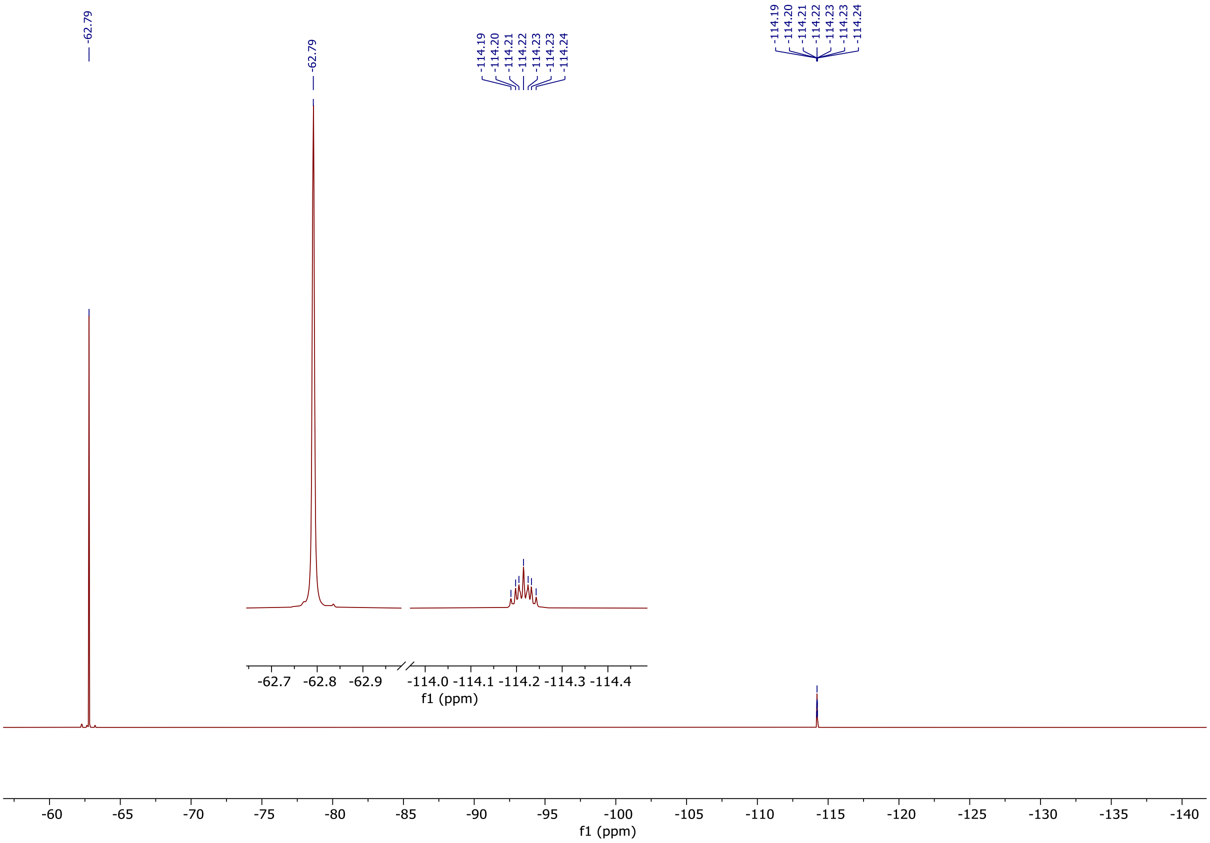

Figure S17: ^{19}F NMR spectrum of compound **2**-CF_3 in $CDCl_3$.

(1*E*)-*N*-(4-fluorophenyl)-1-phenylmethanimine (2-control)

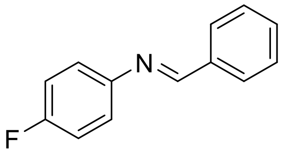

Following general procedure A, benzaldehyde (5 mmol, 530 mg, 1.0 eq), 4-fluoroaniline (6 mmol, 667 mg, 1.2 eq) were dissolved in 10 mL MeOH. Two droplets of acetic acid were added and the reaction mixture was refluxed for 8 h. Upon cooling down, the colourless precipitate was formed which was collected by filtration and washed with cold methanol to provide the title compound as a colourless solid (698 mg, 70% yield). Mp 50 – 52 °C. Spectroscopic data matches literature values.[11] ^{1}H NMR (400 MHz, $CDCl_3$) δ 8.45 (s, 1H), 7.95 – 7.85 (m, 2H), 7.54 – 7.43 (m, 3H), 7.26 – 7.16 (m, 2H), 7.14 – 7.03 (m, 2H). ^{13}C NMR (151 MHz, $CDCl_3$) δ 161.39 (d, J = 244.5 Hz), 160.28, 148.21 (d, J = 3.0 Hz), 136.24, 131.58, 128.94, 128.91, 122.44 (d, J = 8.3 Hz), 116.00 (d, J = 22.6 Hz). ^{19}F NMR (471 MHz, $CDCl_3$) δ -117.31 (ddd, J = 13.3, 8.4, 4.9 Hz). HRMS (ESI+): m/z calcd. for $C_{13}H_{11}FN$ $[M+H]^+$: 200.0870, found 200.0869.

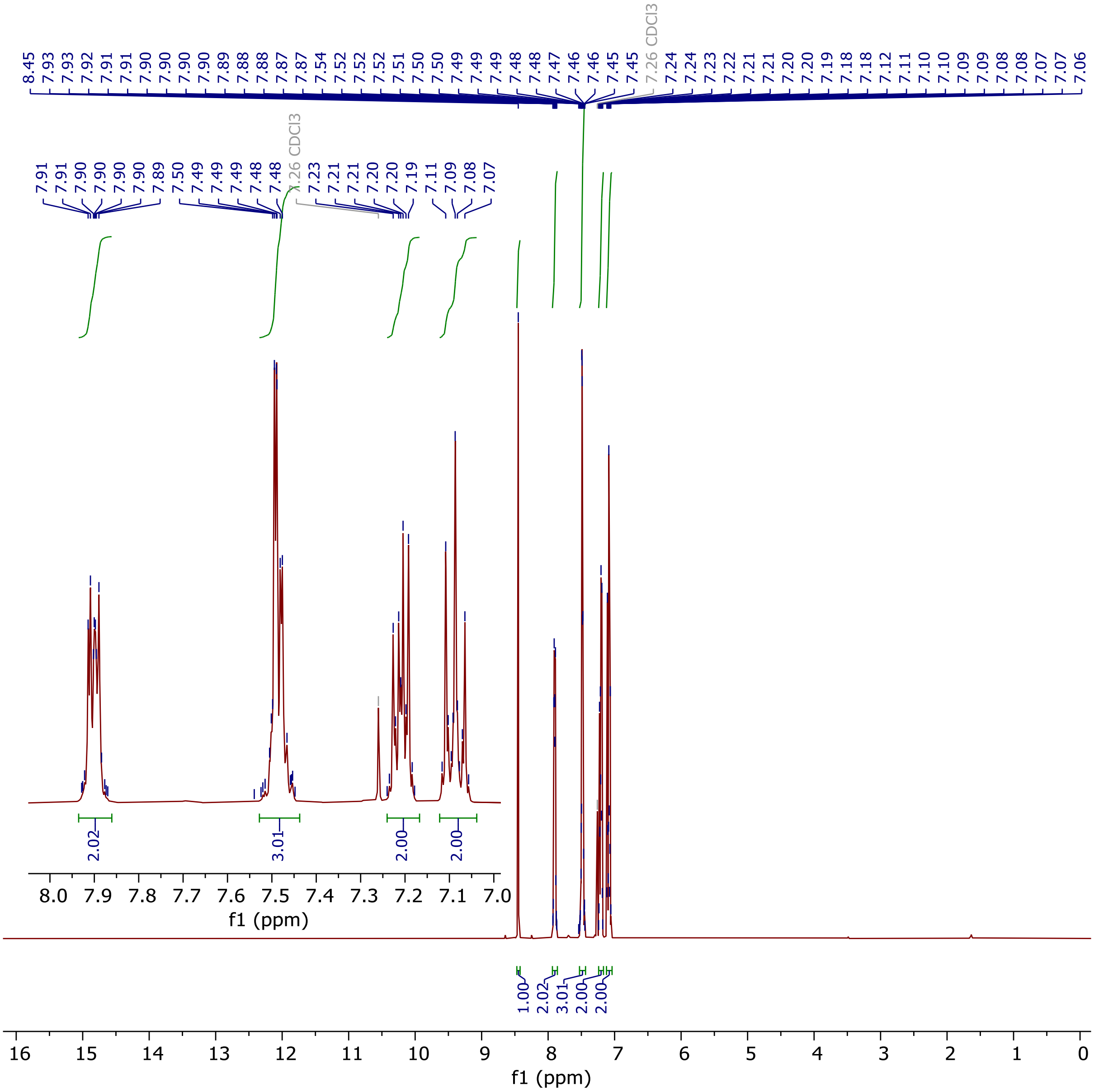

Figure S18: ^{1}H NMR spectrum of compound **2**-control in $CDCl_3$.

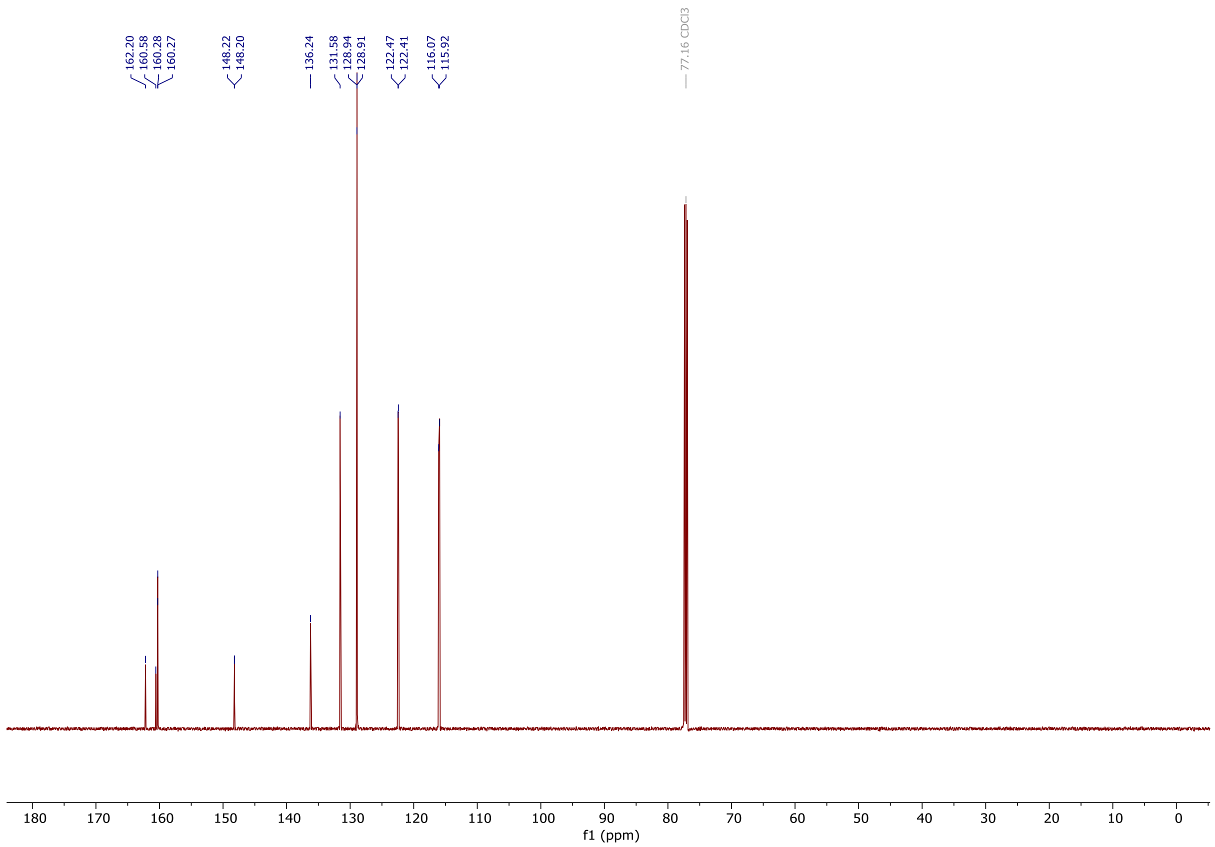

Figure S19: ^{13}C NMR spectrum of compound **2**-control in $CDCl_3$.

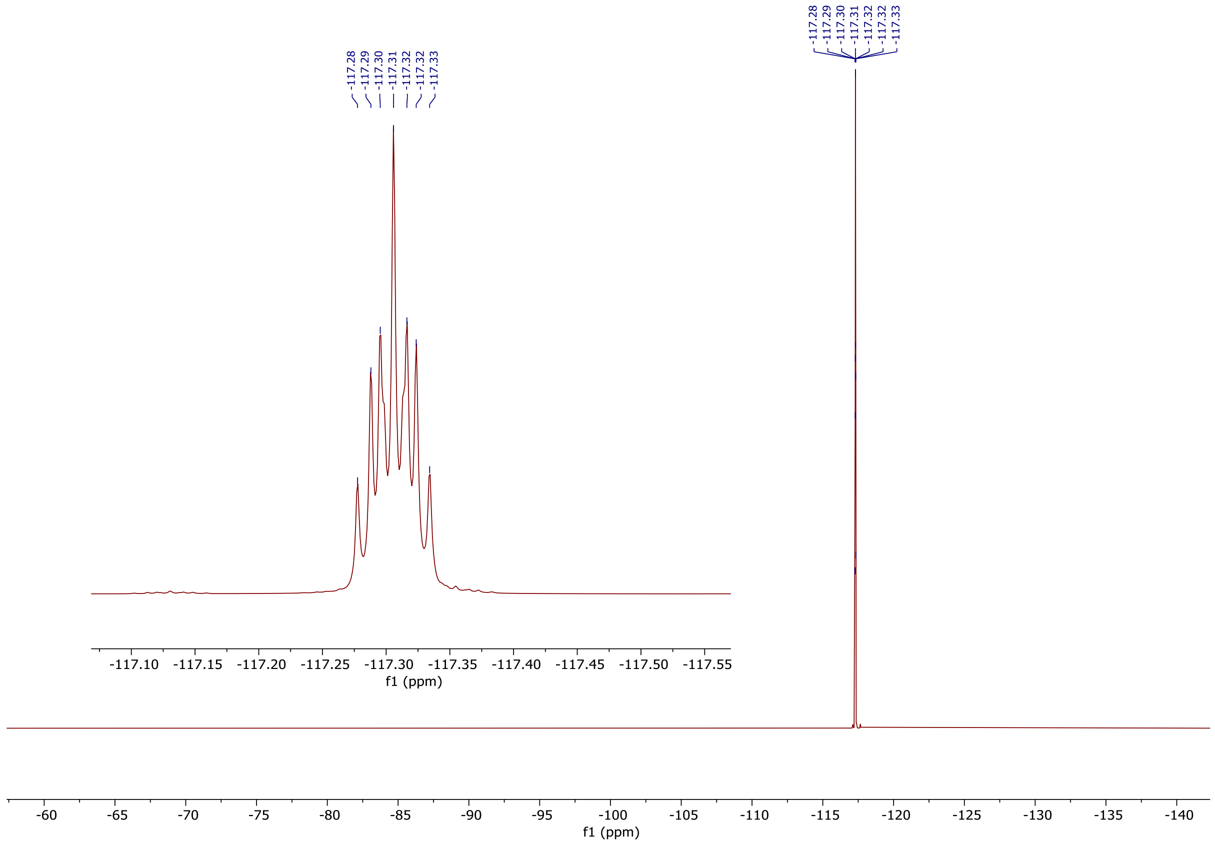

Figure S20: ^{19}F NMR spectrum of compound **2**-control in $CDCl_3$.

2-bromo-6-[(1*E*)-[(4-fluorophenyl)imino]methyl]phenol (2-Br)

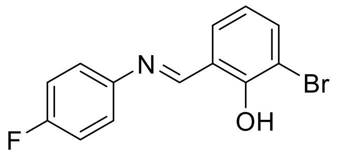

Following general procedure A, 2-hydroxy-3-bromobenzaldehyde (5 mmol, 1.00 g, 1.0 eq), 4-fluoroaniline (6 mmol, 667 mg, 1.2 eq) were dissolved in 10 mL MeOH. Two droplets of acetic acid were added and the reaction mixture was refluxed for 8 h. Upon cooling down, the yellow precipitate was formed which was collected by filtration and washed with cold methanol to provide the title compound as a yellow solid (1.10 g, 75%). Mp 80 – 82 °C. ^{1}H NMR (400 MHz, $CDCl_3$) δ 14.21 (s, 1H), 8.56 (s, 1H), 7.63 (dd, *J* = 7.9, 1.6 Hz, 1H), 7.35 (dd, *J* = 7.7, 1.6 Hz, 1H), 7.33 – 7.23 (m, 2H), 7.17 – 7.07 (m, 2H), 6.84 (t, *J* = 7.8 Hz, 1H). ^{13}C NMR (151 MHz, $CDCl_3$) δ 162.09 (d, *J* = 247.3 Hz), 161.32, 143.62 (d, *J* = 3.0 Hz), 136.44, 131.52, 122.83 (d, *J* = 8.7 Hz), 119.99 (d, *J* = 2.9 Hz), 116.54 (d, *J* = 22.7 Hz), 111.27. ^{19}F NMR (471 MHz, $CDCl_3$) δ -114.43 (ddd, *J* = 13.0, 8.2, 4.8 Hz). HRMS (ESI+): m/z calcd. for $C_{13}H_{10}BrFNO$ [M+H]$^+$: 293.9924, found 293.9901.

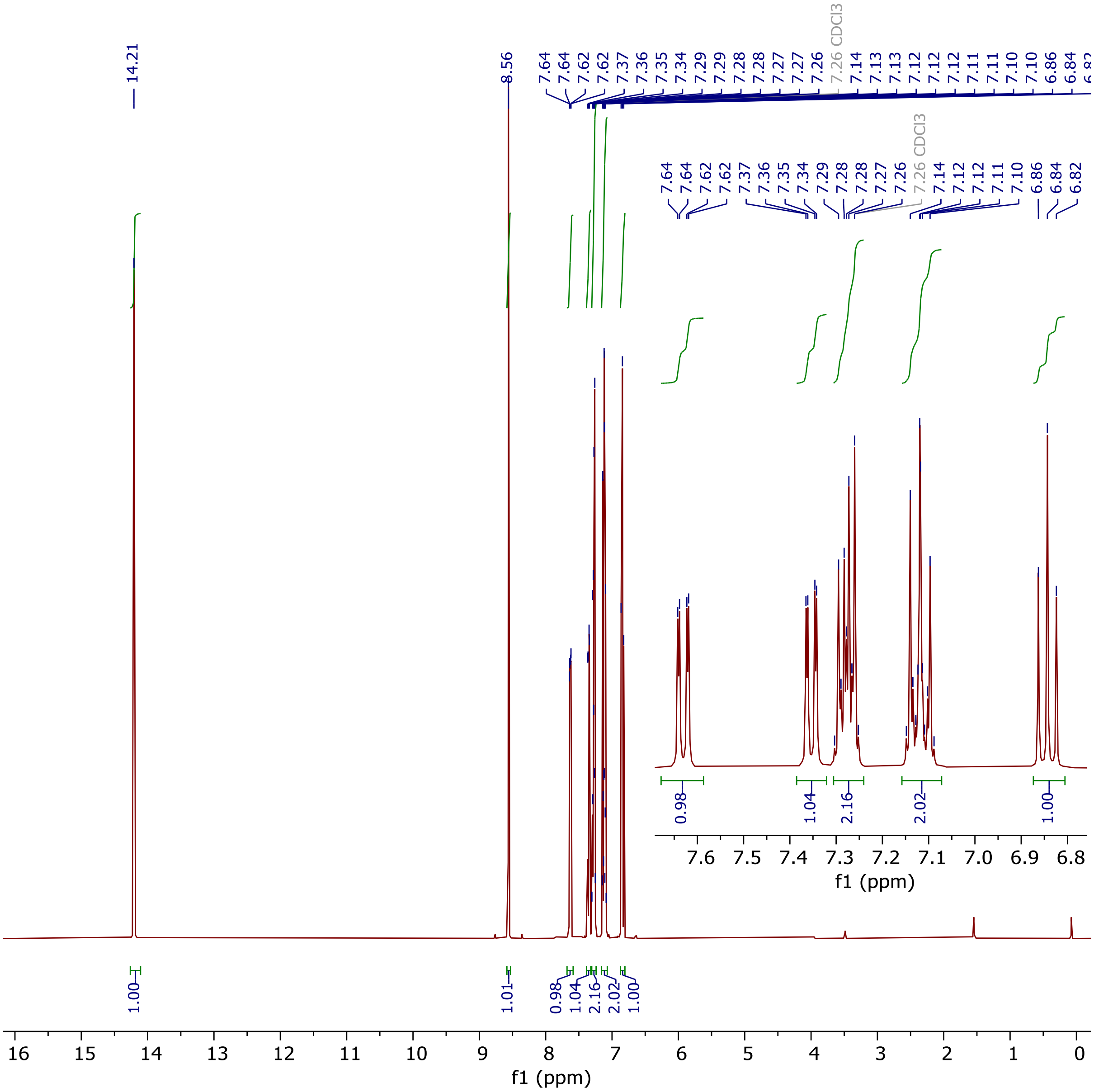

Figure S21: ^{1}H NMR spectrum of compound **2**-Br in $CDCl_3$.

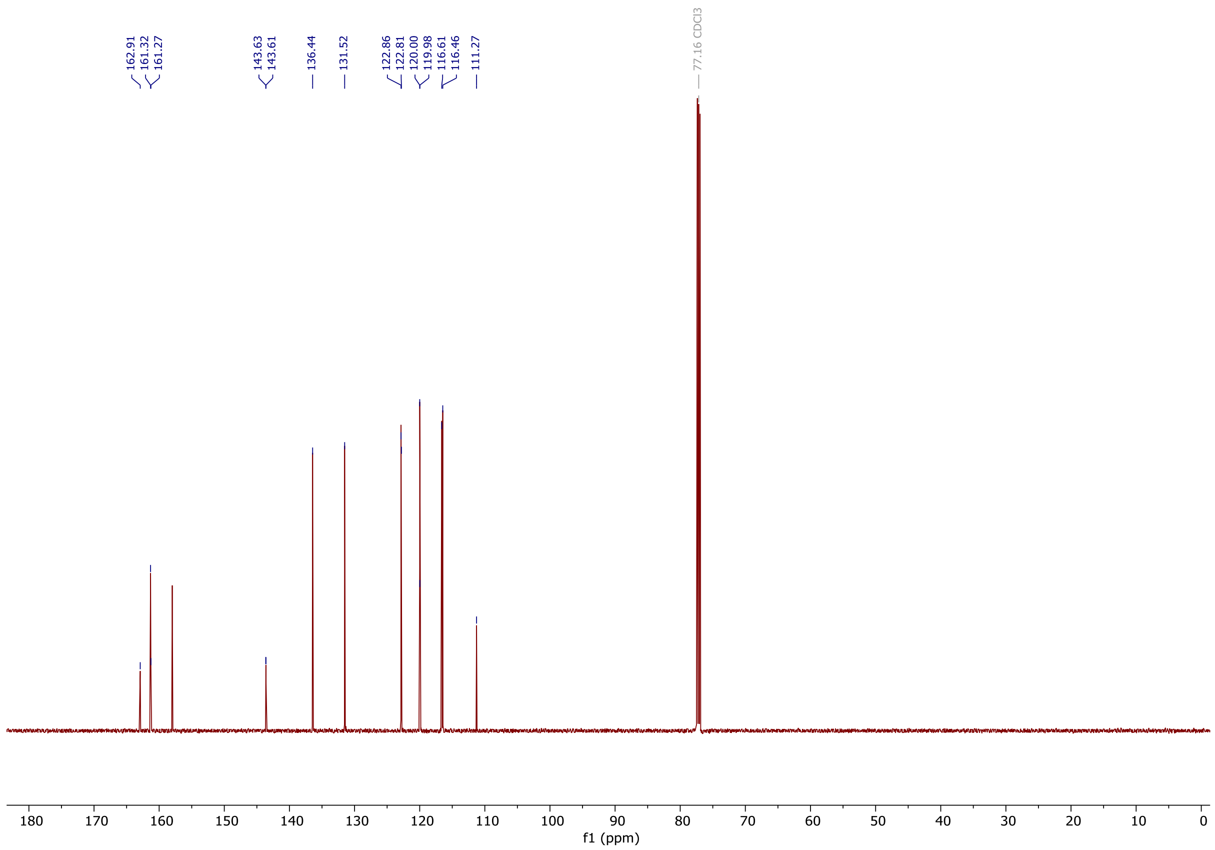

Figure S22: ^{13}C NMR spectrum of compound **2**-Br.

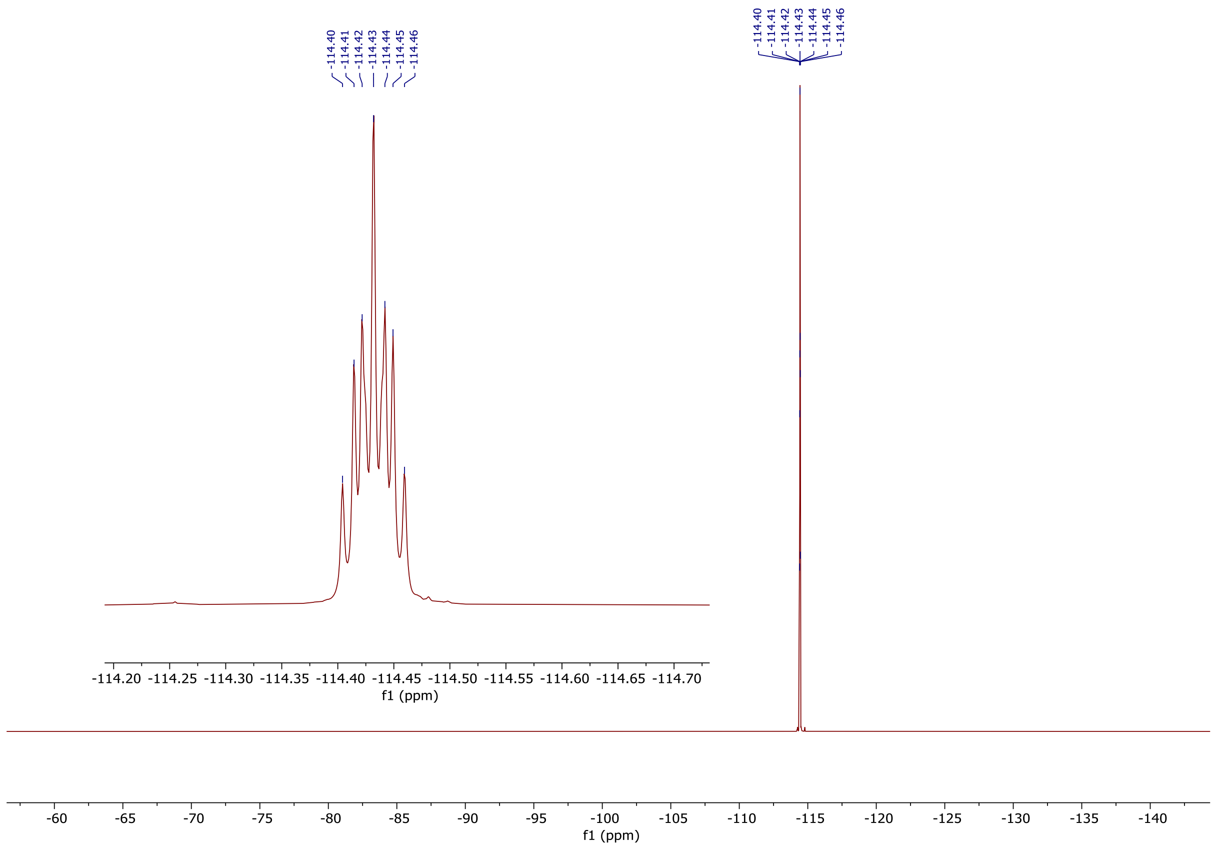

Figure S23: ^{19}F NMR spectrum of compound **2**-Br in $CDCl_3$.

2-[(1*E*)-[(4-fluorophenyl)imino]methyl]-6-nitrophenol (2-NO_2)

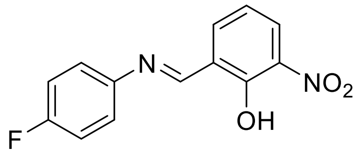

Following general procedure A, 2-hydroxy-3-nitrobenzaldehyde (5 mmol, 835 mg, 1.0 eq), 4-fluoroaniline (6 mmol, 667 mg, 1.2 eq) were dissolved in 10 mL MeOH. Two droplets of acetic acid were added and the reaction mixture was refluxed for 8 h. Upon cooling down, the red precipitate was formed which was collected by filtration and washed with cold methanol to provide the title compound as a red solid (1.00 g, 78%). Mp 132 – 134 °C. ^{1}H NMR (400 MHz, $CDCl_3$) δ 15.05 (br s, 1H), 8.71 (s, 1H), 8.11 (dd, *J* = 8.2, 1.7 Hz, 1H), 7.71 (dd, *J* = 7.6, 1.7 Hz, 1H), 7.38 – 7.28 (m, 2H), 7.20 – 7.10 (m, 2H), 7.02 (dd, *J* = 8.2, 7.6 Hz, 1H). ^{13}C NMR (151 MHz, $CDCl_3$) δ 162.44 (d, *J* = 248.6 Hz), 160.16, 156.68, 142.72 (d, *J* = 3.0 Hz), 138.03, 137.34, 129.50, 122.93 (d, *J* = 8.4 Hz), 121.77, 118.15, 116.77 (d, *J* = 23.1 Hz). ^{19}F NMR (471 MHz, $CDCl_3$) δ -113.28. HRMS (ESI+): m/z calcd. for $C_{13}H_9FN_2O_3Na$ $[M+Na]^+$: 283.0489, found 283.0498.

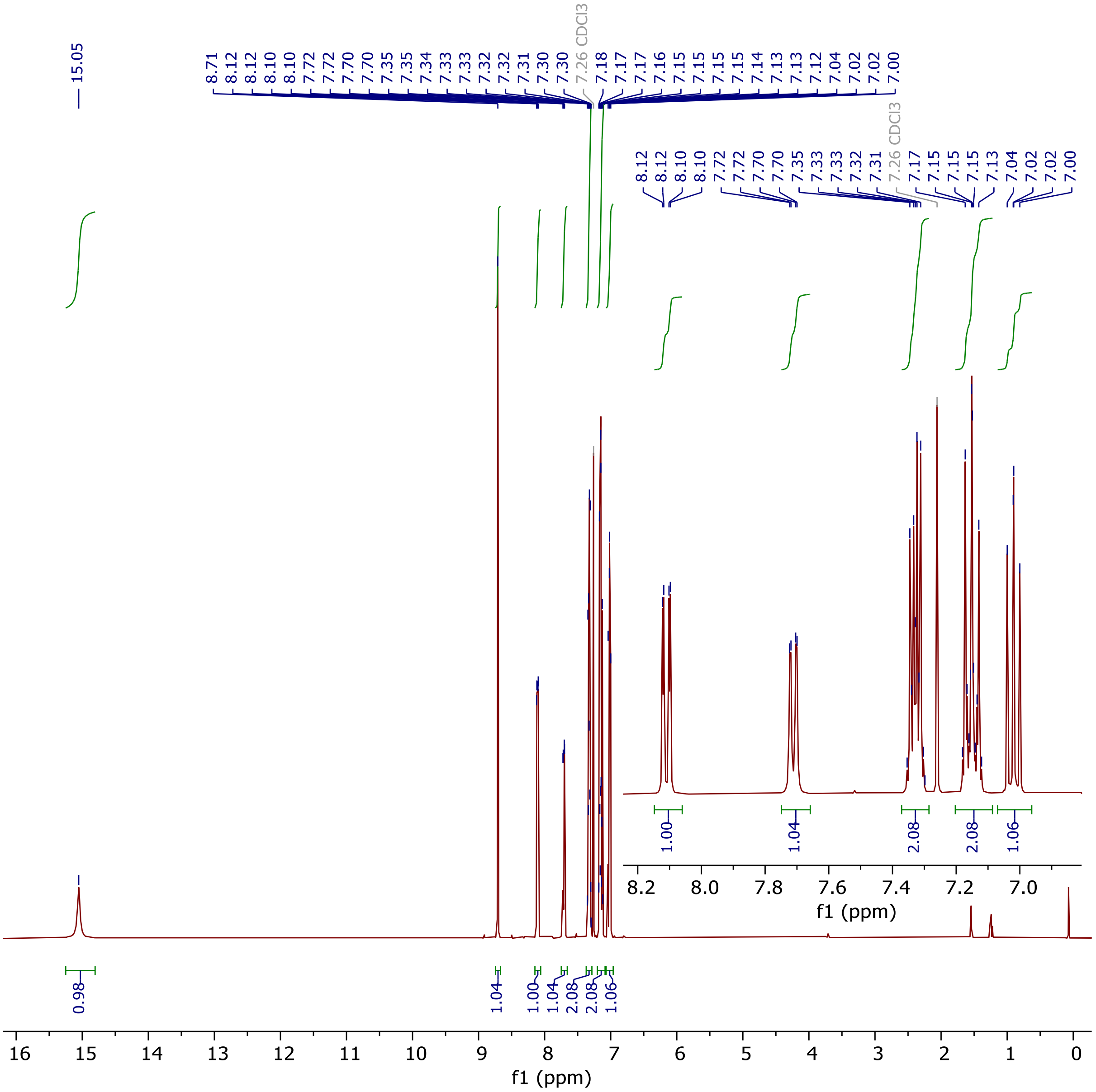

Figure S24: ^{1}H NMR spectrum of compound **2**-NO_2 in $CDCl_3$.

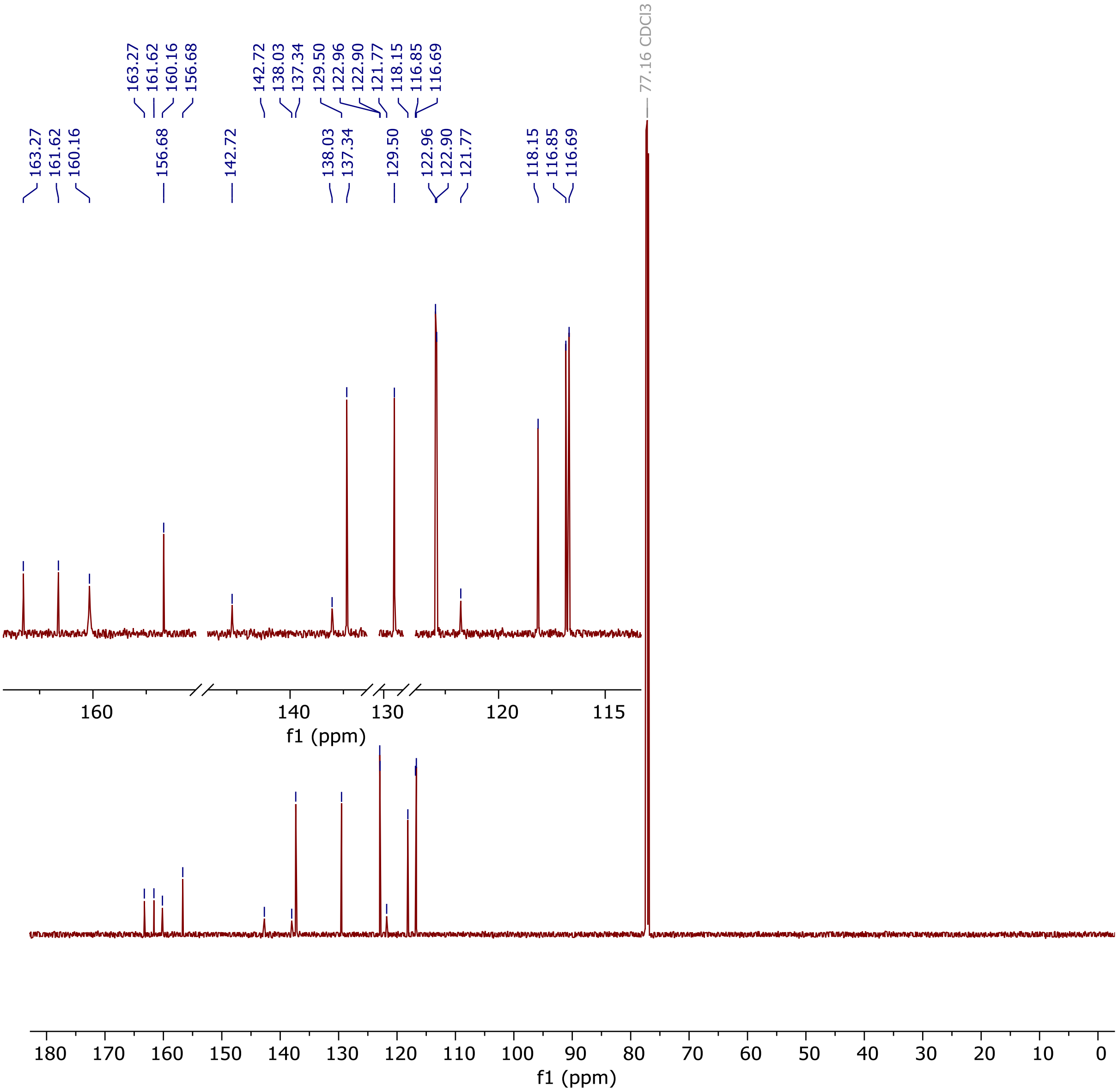

Figure S25: ^{13}C NMR spectrum of compound **2**-NO_2 in $CDCl_3$.

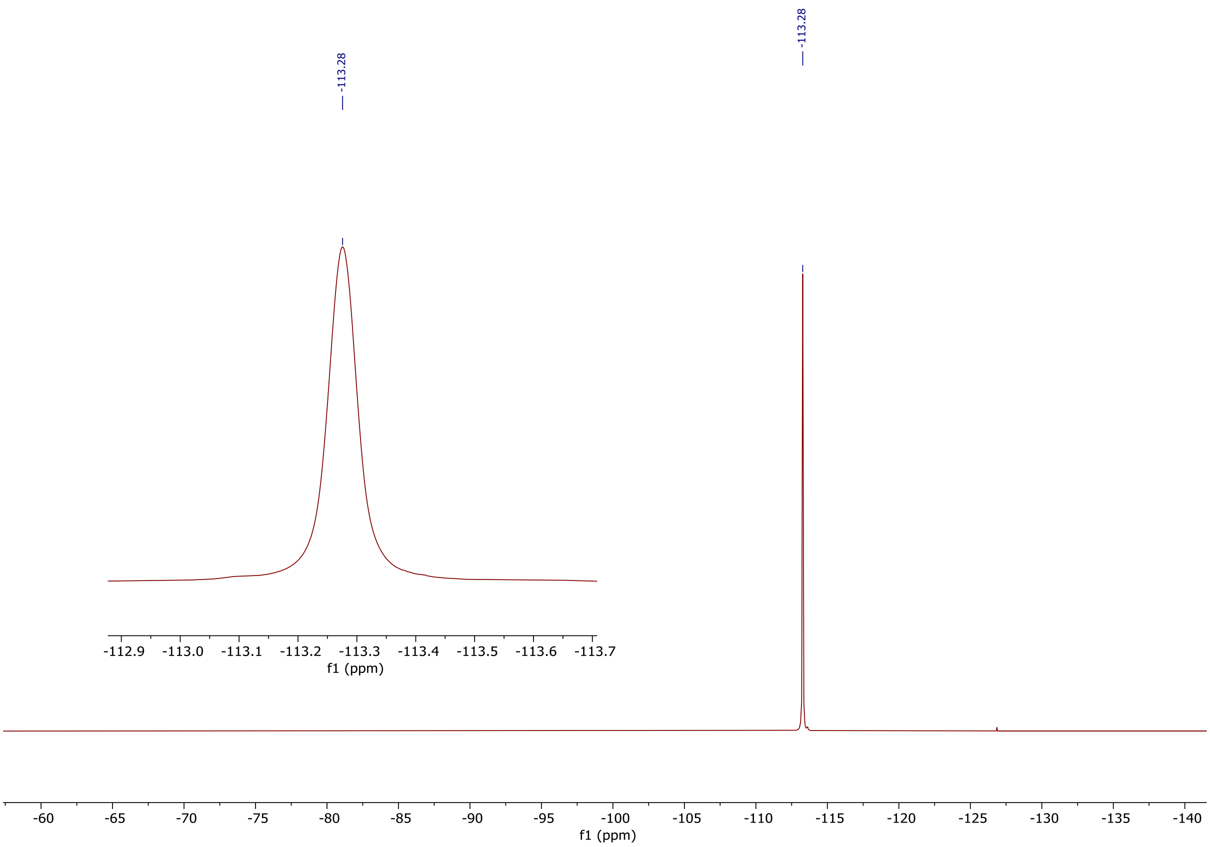

Figure S26: ^{19}F NMR spectrum of compound **2**-NO_2 in $CDCl_3$.

2-chloro-6-[(1*E*)-[(4-fluorophenyl)imino]methyl]phenol (2-Cl)

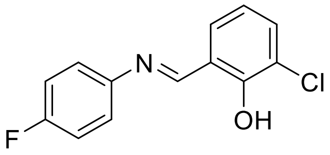

Following general procedure A, 2-hydroxy-3-chlorobenzaldehyde (5 mmol, 783 mg, 1.0 eq), 4-fluoroaniline (6 mmol, 667 mg, 1.2 eq) were dissolved in 10 mL MeOH. Two droplets of acetic acid were added and the reaction mixture was refluxed for 6 h. Upon cooling down, the yellow precipitate was formed which was collected by filtration and washed with cold methanol to provide the title compound as a yellow solid (1.06 g, 85%). Mp 74 – 76 °C. ^{1}H NMR (400 MHz, $CDCl_3$) δ 14.06 (s, 1H), 8.60 (s, 1H), 7.48 (dd, J = 8.0, 1.6 Hz, 1H), 7.32 (dd, J = 7.7, 1.6 Hz, 1H), 7.31 – 7.27 (m, 2H), 7.18 – 7.07 (m, 2H), 6.90 (t, J = 7.8 Hz, 1H). ^{13}C NMR (151 MHz, $CDCl_3$) δ 162.11 (d, J = 247.3 Hz), 161.52, 157.12, 143.79 (d, J = 3.1 Hz), 133.44, 130.74, 122.85 (d, J = 8.4 Hz), 122.05, 120.08, 119.43, 116.56 (d, J = 23.1 Hz). ^{19}F NMR (471 MHz, $CDCl_3$) δ -114.52 (ddd, J = 13.0, 8.3, 4.9 Hz). HRMS (ESI+): m/z calcd. for $C_{13}H_{10}ClFNO$ $[M+H]^+$: 250.0430, found 250.0425.

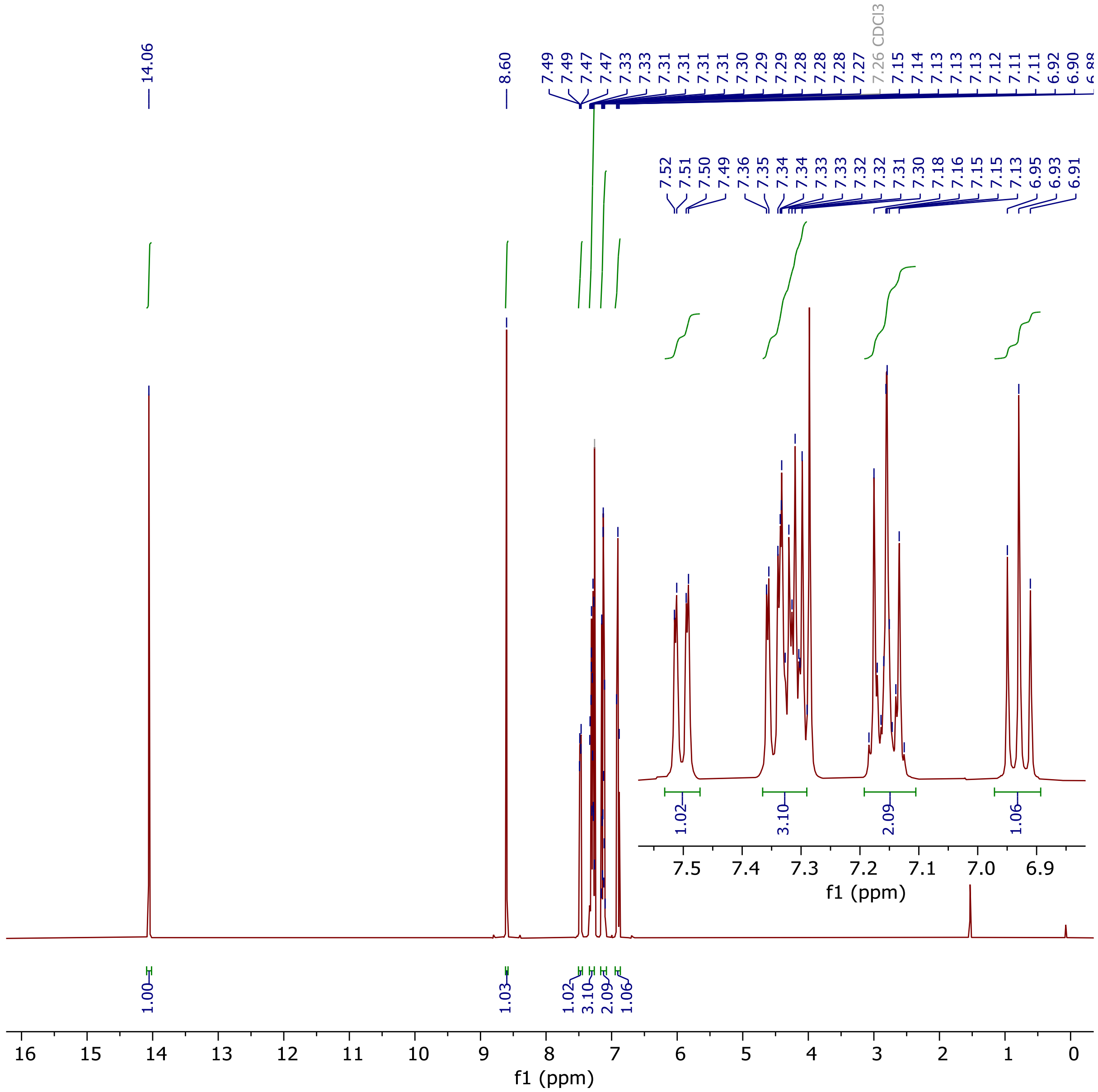

Figure S27: ^{1}H NMR spectrum of compound **2**-Cl in $CDCl_3$.

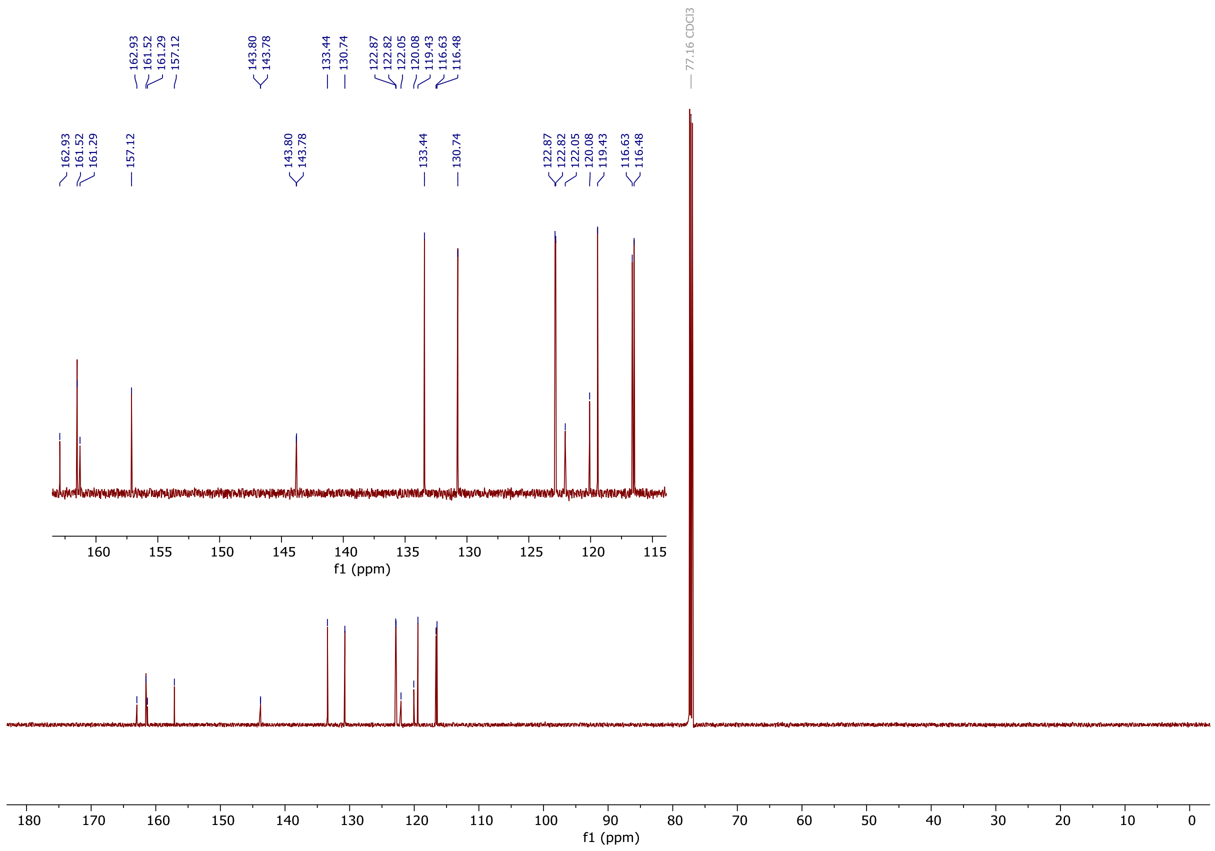

Figure S28: ^{13}C NMR spectrum of compound **2**-Cl in $CDCl_3$.

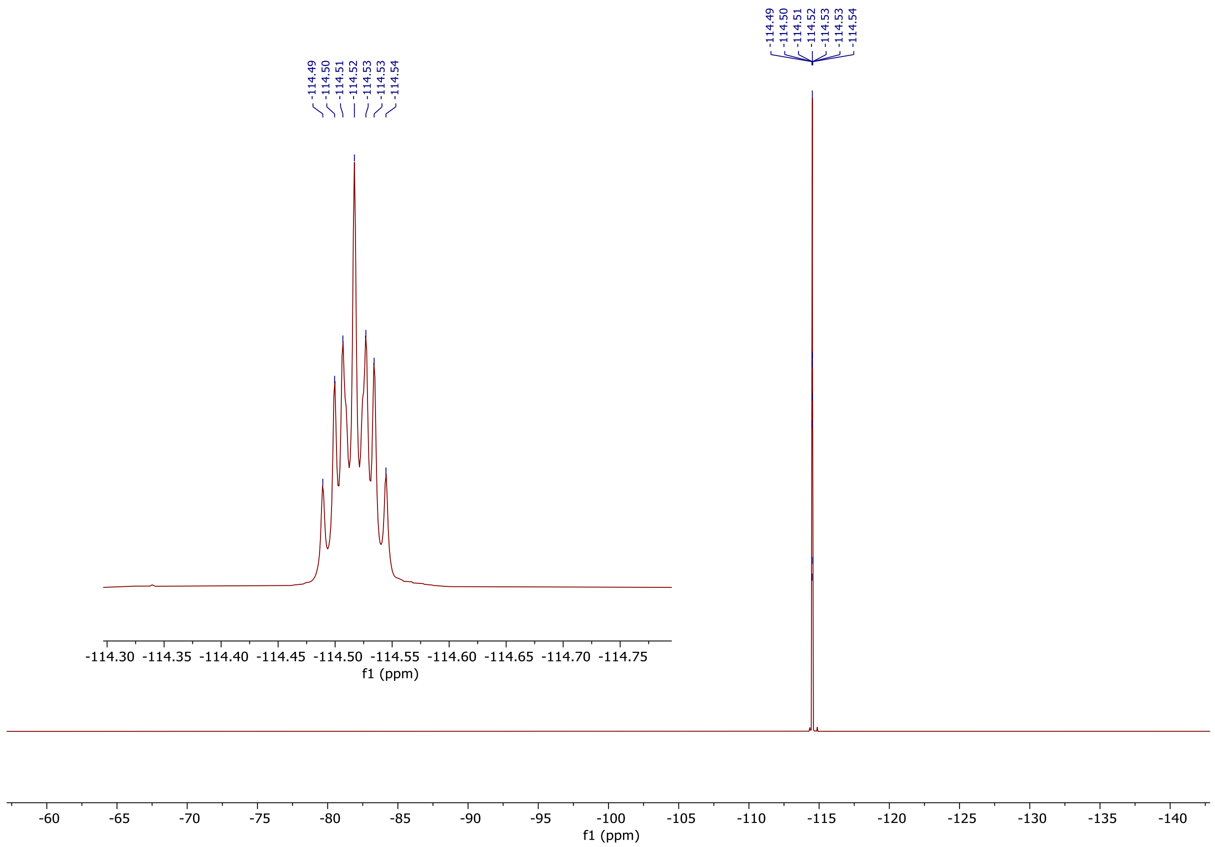

Figure S29: ^{19}F NMR spectrum of compound **2**-Cl in $CDCl_3$.

2-*tert*-butyl-6-[(1*E*)-[4-fluorophenyl)imino]methyl]phenol (2-^{t}Bu)

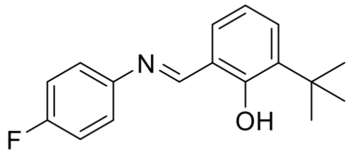

Following general procedure A, 3-*tert*-butyl-2-hydroxybenzaldehyde (5 mmol, 890 mg, 1.0 eq), 4-fluoroaniline (6 mmol, 667 mg, 1.2 eq) were dissolved in 10 mL MeOH. Two droplets of acetic acid were added and the reaction mixture was refluxed for 6 h. Upon cooling down, the volatiles were removed in vacuo. To the residual crude product was added 100 µL acetic acid and the resulting mixture was passed through a 10 cm silica plug (eluting with 100% DCM). After evaporation of the volatiles, the title compound was obtained as an orange oil (1.20 g, 89%). Spectroscopic data matches literature values.[12] ^{1}H NMR (400 MHz, $CDCl_3$) δ 13.75 (s, 1H), 8.60 (s, 1H), 7.40 (dd, *J* = 8.0, 1.5 Hz, 1H), 7.29 – 7.26 (m,2), 7.25 (dd, *J* = 7.4, 1.9 Hz, 1H), 7.16 – 7.06 (m, 2H), 6.89 (t, *J* = 7.7 Hz, 1H), 1.47 (s, 9H). ^{13}C NMR (151 MHz, $CDCl_3$) δ 163.32, 161.70 (d, *J* = 246.1 Hz), 160.58, 144.82 (d, *J* = 3.0 Hz), 137.87, 130.80, 130.59, 122.73 (d, *J* = 8.4 Hz), 118.57, 116.33 (d, *J* = 22.7 Hz), 35.06, 29.48. ^{19}F NMR (471 MHz, $CDCl_3$) δ -115.89 (ddd, *J* = 13.1, 8.3, 4.9 Hz). HRMS (ESI+): m/z calcd. $C_{17}H_{19}FNO$ $[M+H]^+$: 272.1445, found 272.1463.

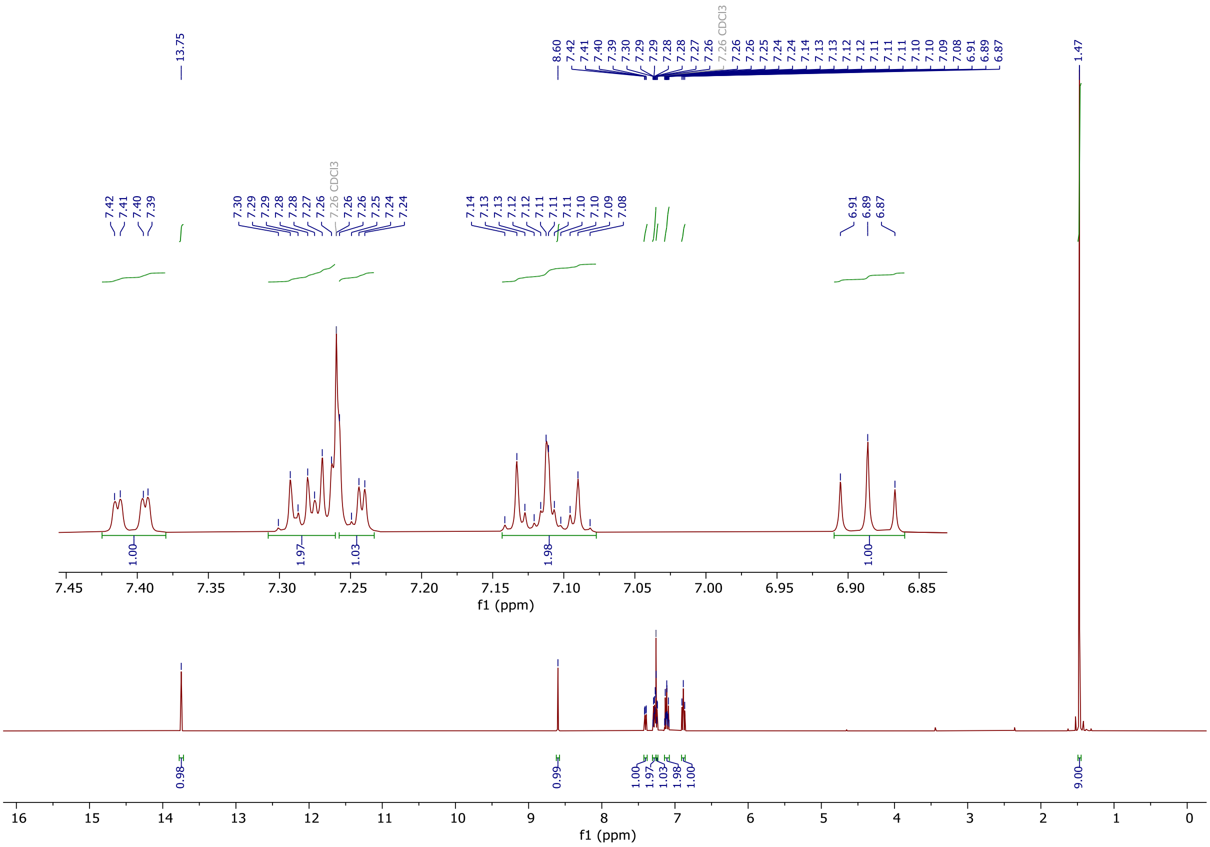

Figure S30: ^{1}H NMR spectrum of compound **2**-^{t}Bu in $CDCl_3$.

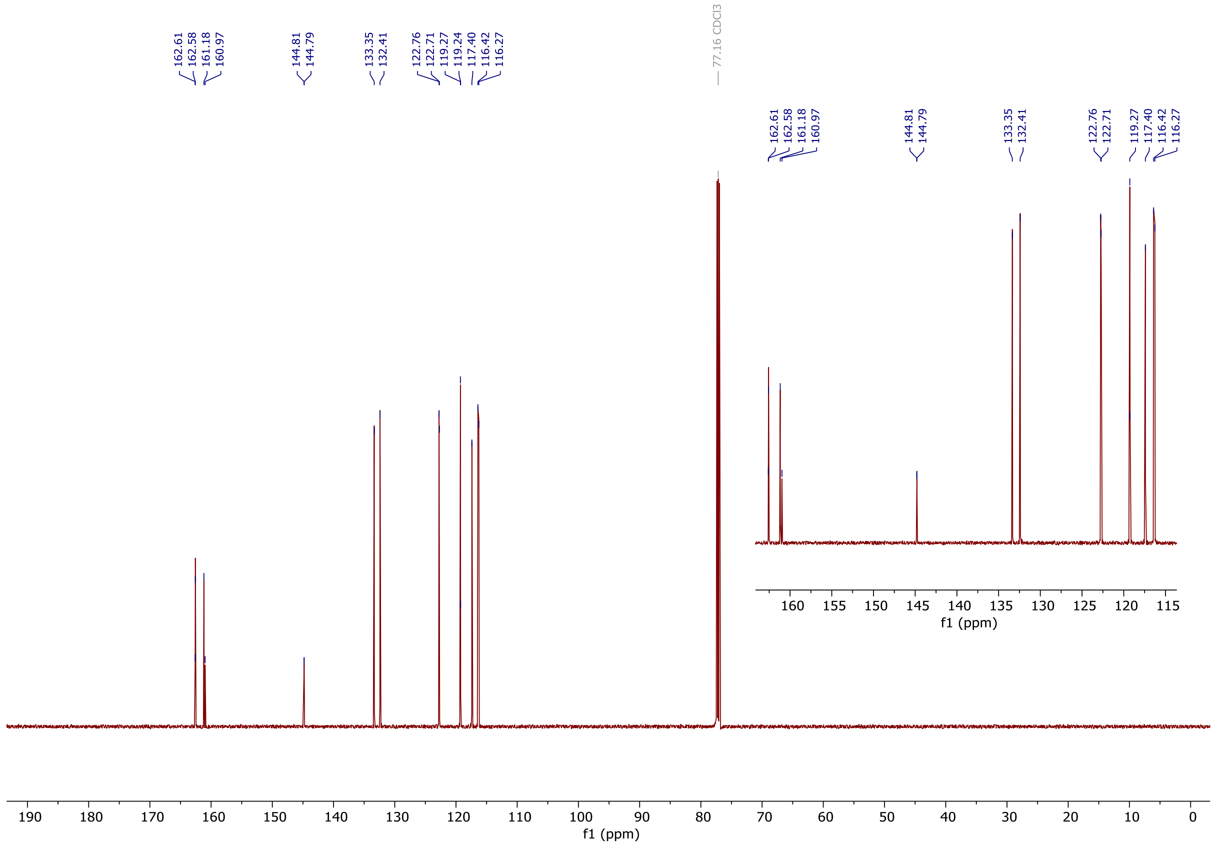

Figure S31: ^{13}C NMR spectrum of compound **2**-^{t}Bu in $CDCl_3$.

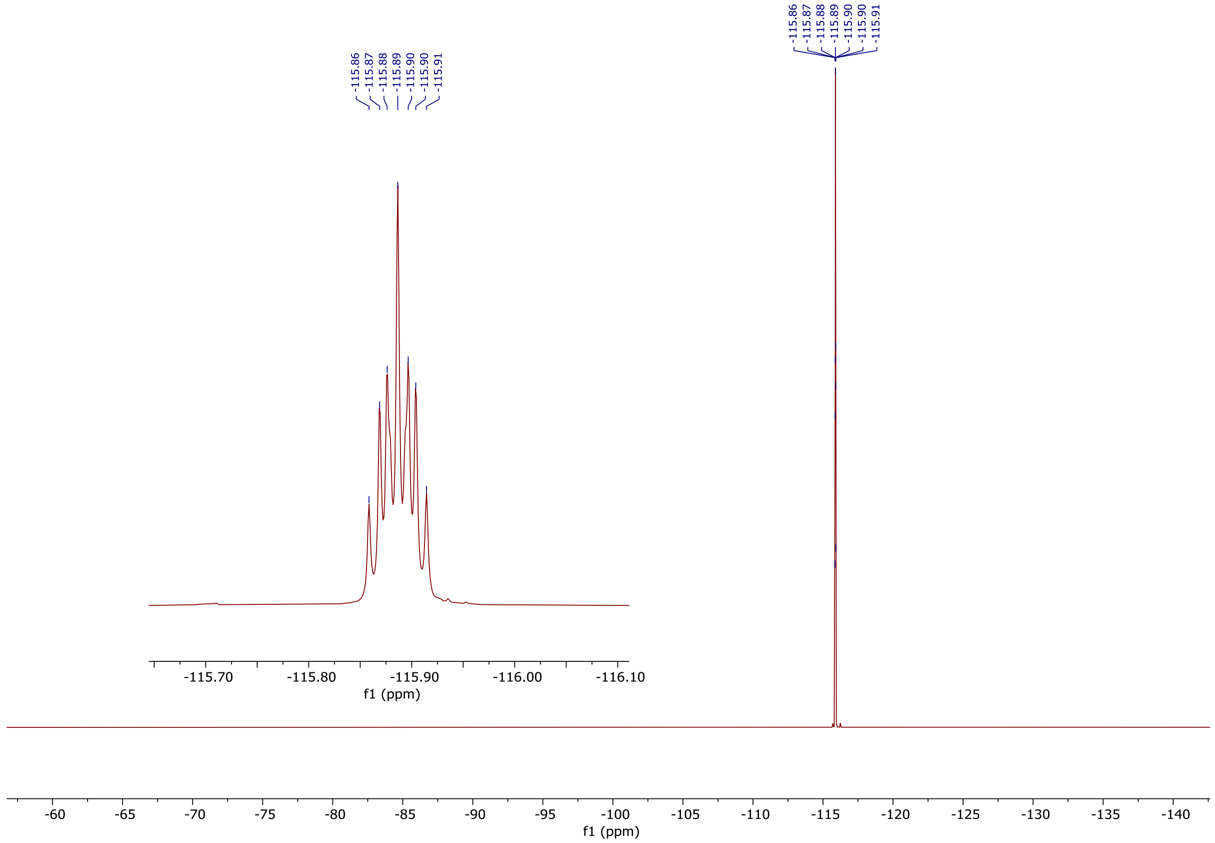

Figure S32: ^{19}F NMR spectrum of compound **2**-^{t}Bu in $CDCl_3$.

2-[(1E)-[(4-fluorophenyl)imino]methyl]phenol (2-H)

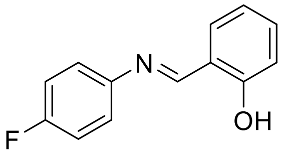

Following general procedure A, salicylaldehyde (5 mmol, 610 mg, 1.0 eq), 4-fluoroaniline (6 mmol, 667 mg, 1.2 eq) were dissolved in 10 mL MeOH. Two droplets of acetic acid were added and the reaction mixture was refluxed for 6 h. Upon cooling down, the yellow precipitate was formed which was collected by filtration and washed with cold methanol to provide the title compound as a yellow solid (967 mg, 90%). Mp 75 – 77 °C. Spectroscopic data matches literature values.[13] ^{1}H NMR (400 MHz, $CDCl_3$) δ 13.10 (s, 1H), 8.59 (s, 1H), 7.43 – 7.34 (m, 2H), 7.30 – 7.22 (m, 2H), 7.17 – 7.07 (m, 2H), 7.03 (dd, J = 8.7, 1.1 Hz, 1H), 6.95 (td, J = 7.4, 1.1 Hz, 1H). ^{13}C NMR (151 MHz, $CDCl_3$) δ 162.58, 161.79 (d, J = 246.8 Hz), 161.18, 144.80 (d, J = 3.0 Hz), 133.35, 132.41, 122.73 (d, J = 8.4 Hz), 119.27, 119.24, 117.40, 116.35 (d, J = 22.7 Hz). ^{19}F NMR (471 MHz, $CDCl_3$) δ -115.54 (ddd, J = 13.1, 8.3, 4.9 Hz). HRMS (ESI+): m/z calcd. for $C_{13}H_{11}FNO$ [M+H]$^+$: 216.0819, found 216.0879.

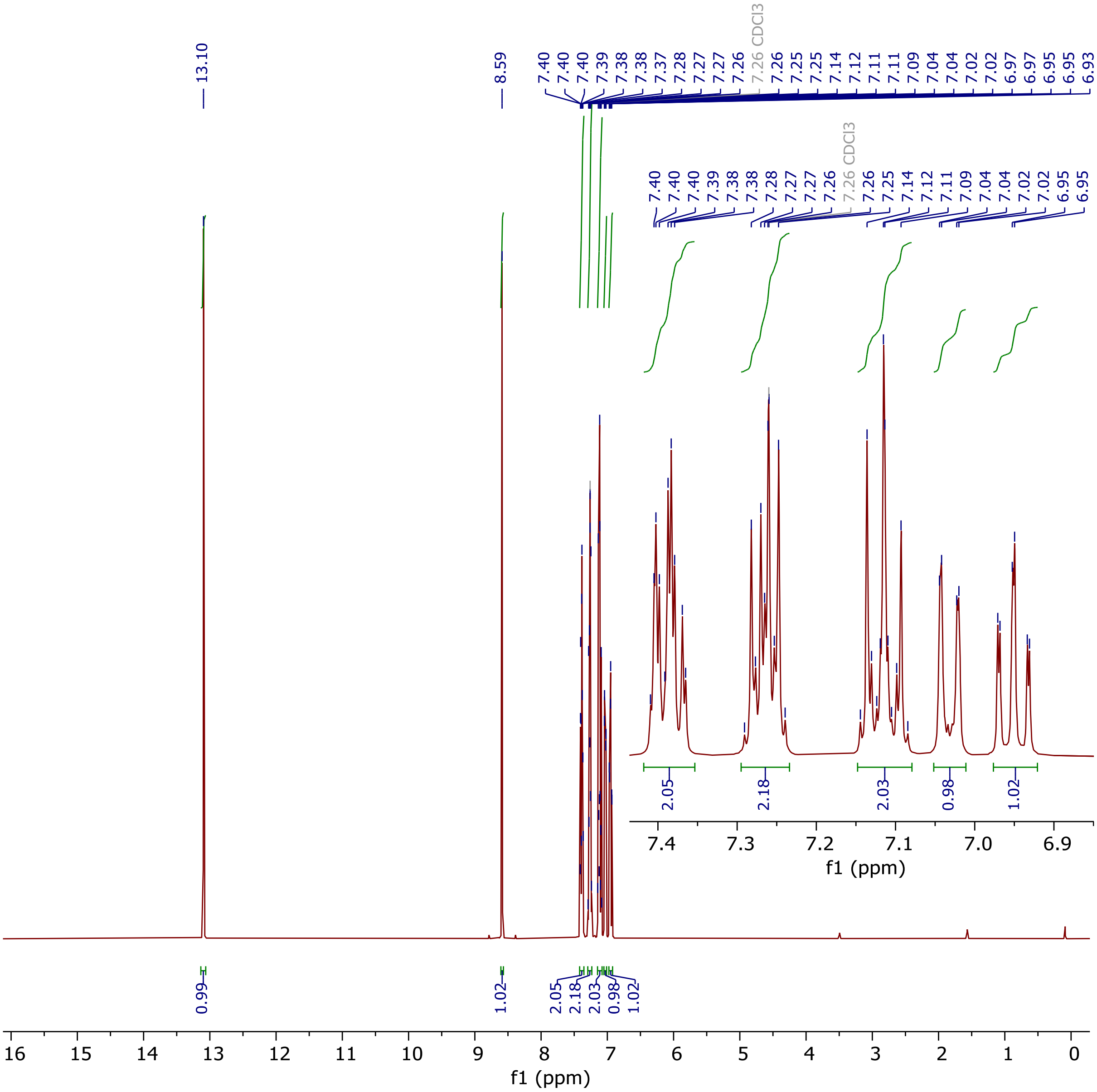

Figure S33: ^{1}H NMR spectrum of compound **2**-H in $CDCl_3$.

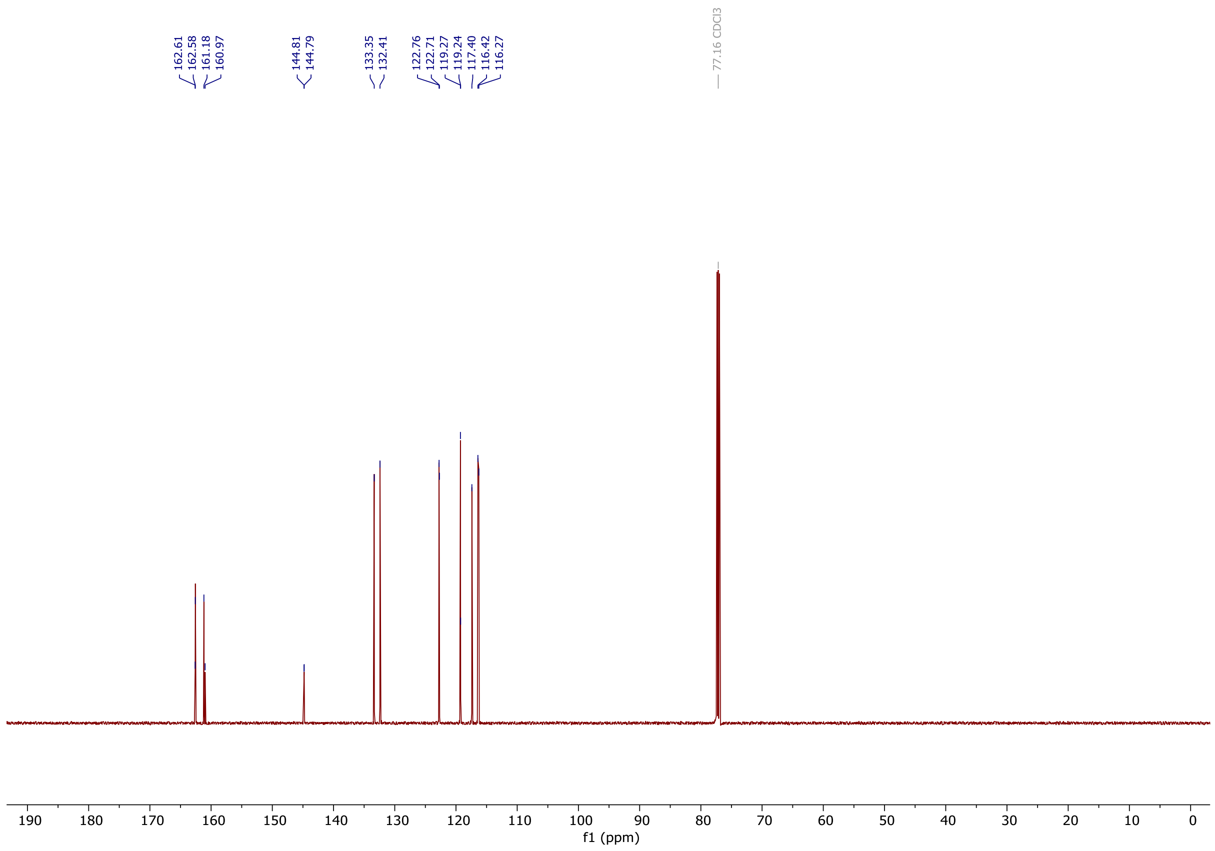

Figure S34: ^{13}C NMR spectrum of compound **2**-H in $CDCl_3$.

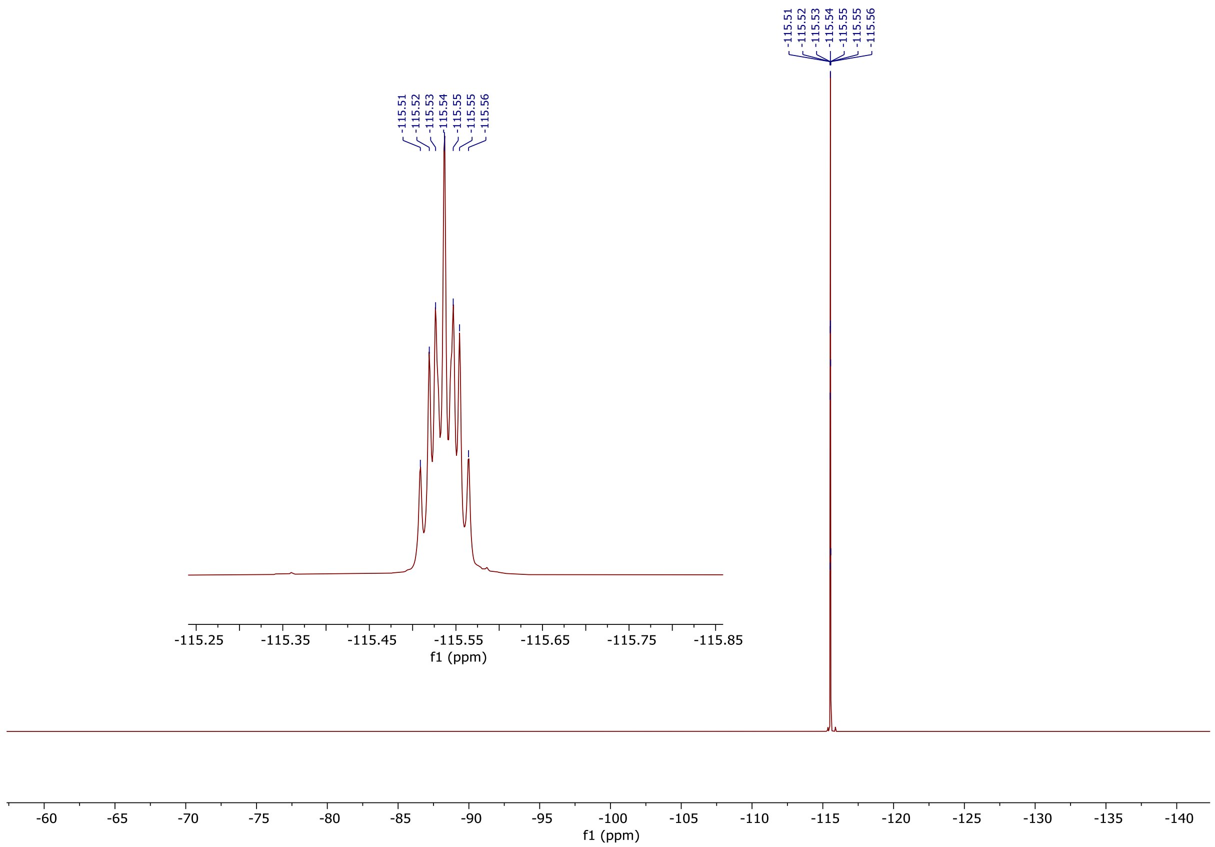

Figure S35: ^{19}F NMR spectrum of compound **2**-H in $CDCl_3$.

2-fluoro-6-[(1*E*)-[(4-fluorophenyl)imino]methyl]phenol (2-F)

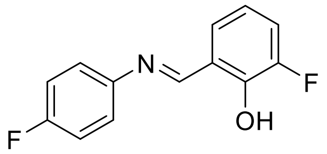

Following general procedure A, 2-hydroxy-3-fluorobenzaldehyde (5 mmol, 700 mg, 1.0 eq), 4-fluoroaniline (6 mmol, 667 mg, 1.2 eq) were dissolved in 10 mL MeOH. Two droplets of acetic acid were added and the reaction mixture was refluxed for 6 h. Upon cooling down, the yellow precipitate was formed which was collected by filtration and washed with cold methanol to provide the title compound as a yellow solid (874 mg, 75%). Mp 125 – 127 °C. ^{1}H NMR (400 MHz, $CDCl_3$) δ 13.40 (s, 1H), 8.61 (d, J = 1.2 Hz, 1H), 7.30 – 7.24 (m, 2H), 7.22 – 7.16 (m, 2H), 7.15 – 7.08 (m, 2H), 6.87 (td, J = 7.9, 4.4 Hz, 1H). ^{13}C NMR (151 MHz, $CDCl_3$) δ 162.05 (d, J = 246.8 Hz), 161.93 (d, J = 5.1 Hz), 151.49 (d, J = 245.6 Hz), 149.67 (d, J = 13.0 Hz), 144.13 (d, J = 3.0 Hz), 127.27 (d, J = 3.3 Hz), 122.82 (d, J = 8.4 Hz), 121.00 (d, J = 4.0 Hz), 119.42 (d, J = 17.8 Hz), 118.61 (d, J = 6.9 Hz), 116.48 (d, J = 22.6 Hz). ^{19}F NMR (471 MHz, $CDCl_3$) δ -114.72 (ddd, J = 13.1, 8.3, 4.9 Hz), -137.47 (dd, J = 10.6, 4.4 Hz). HRMS (ESI+): m/z calcd. for $C_{13}H_9F_2NONa$ $[M+Na]^+$: 256.0544, found 256.0543.

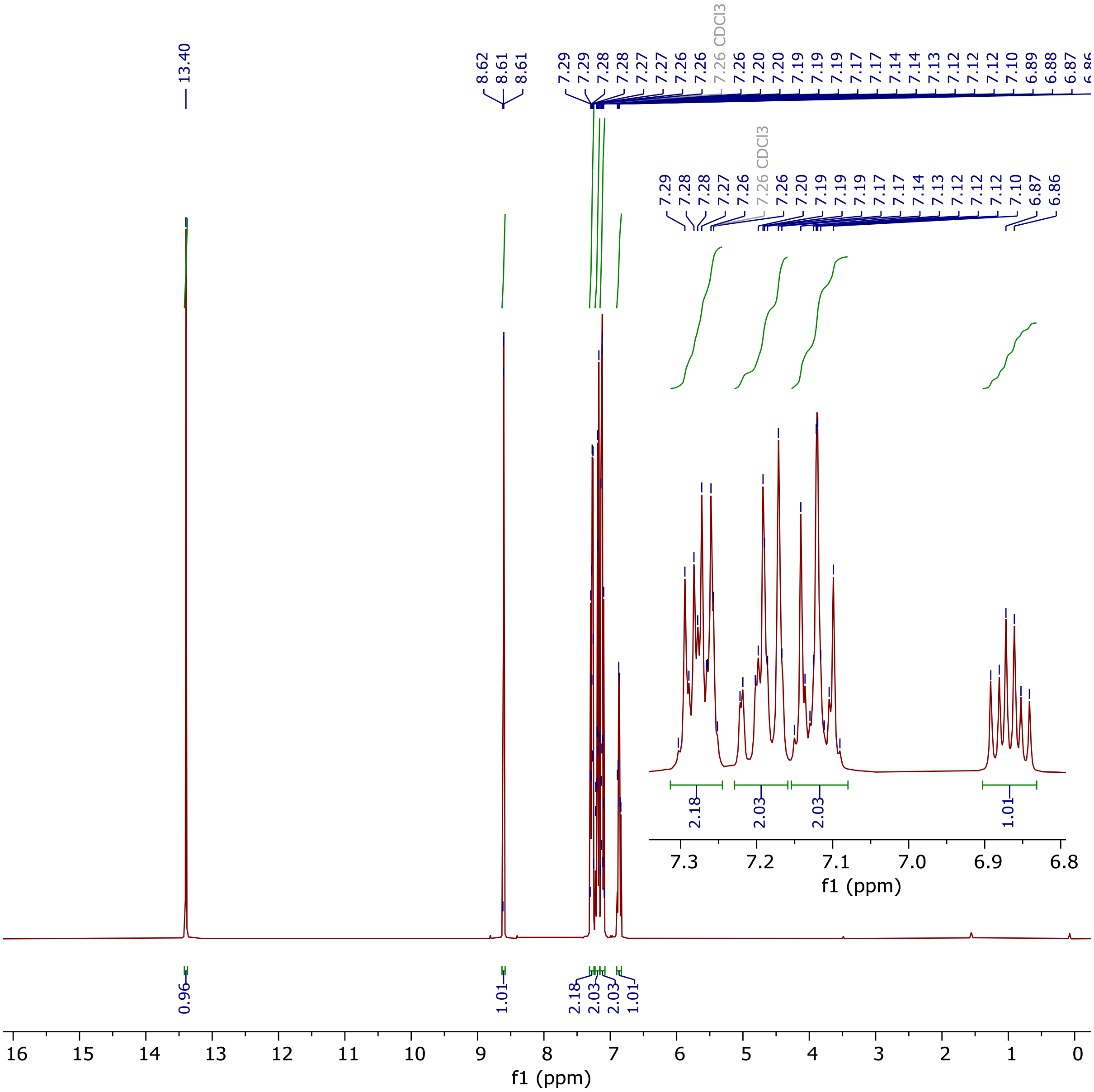

Figure S36: ^{1}H NMR spectrum of compound **2**-F in $CDCl_3$.

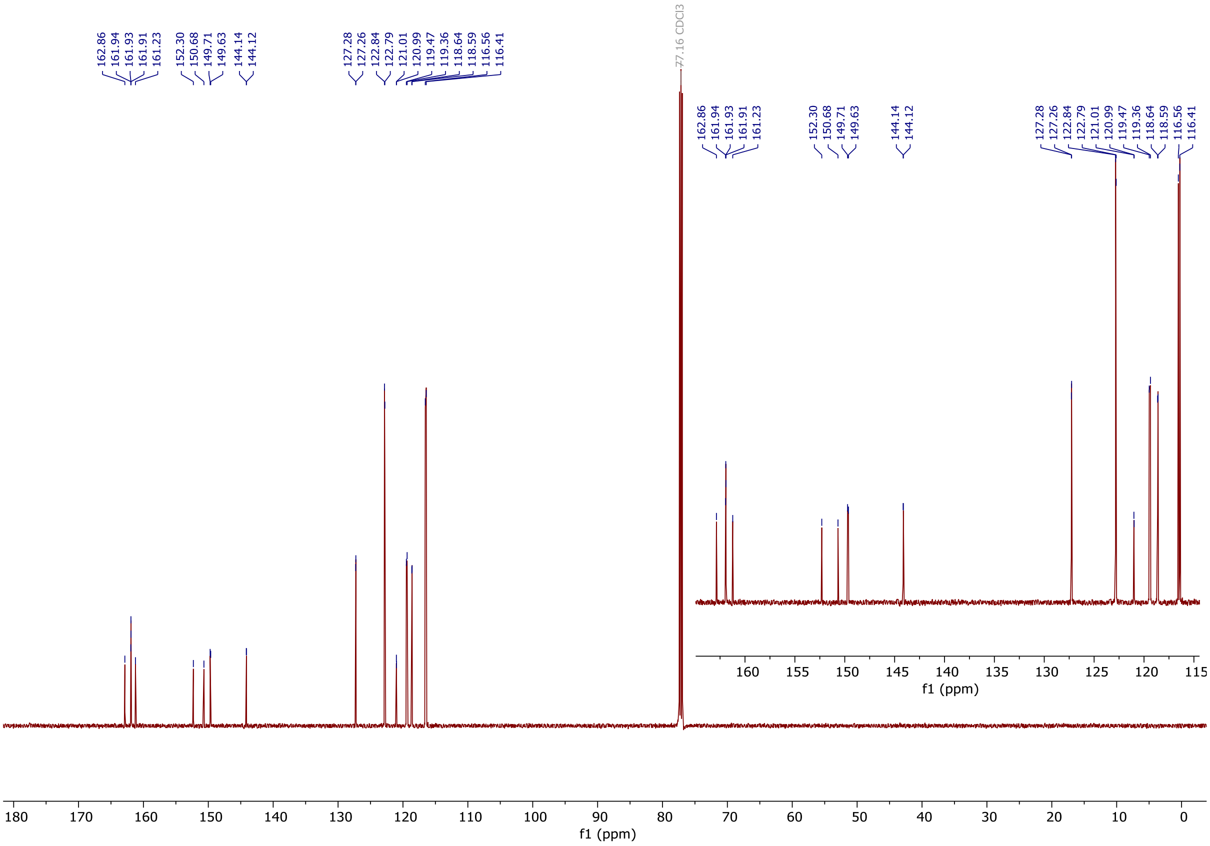

Figure S37: ^{13}C NMR spectrum of compound **2**-F in $CDCl_3$.

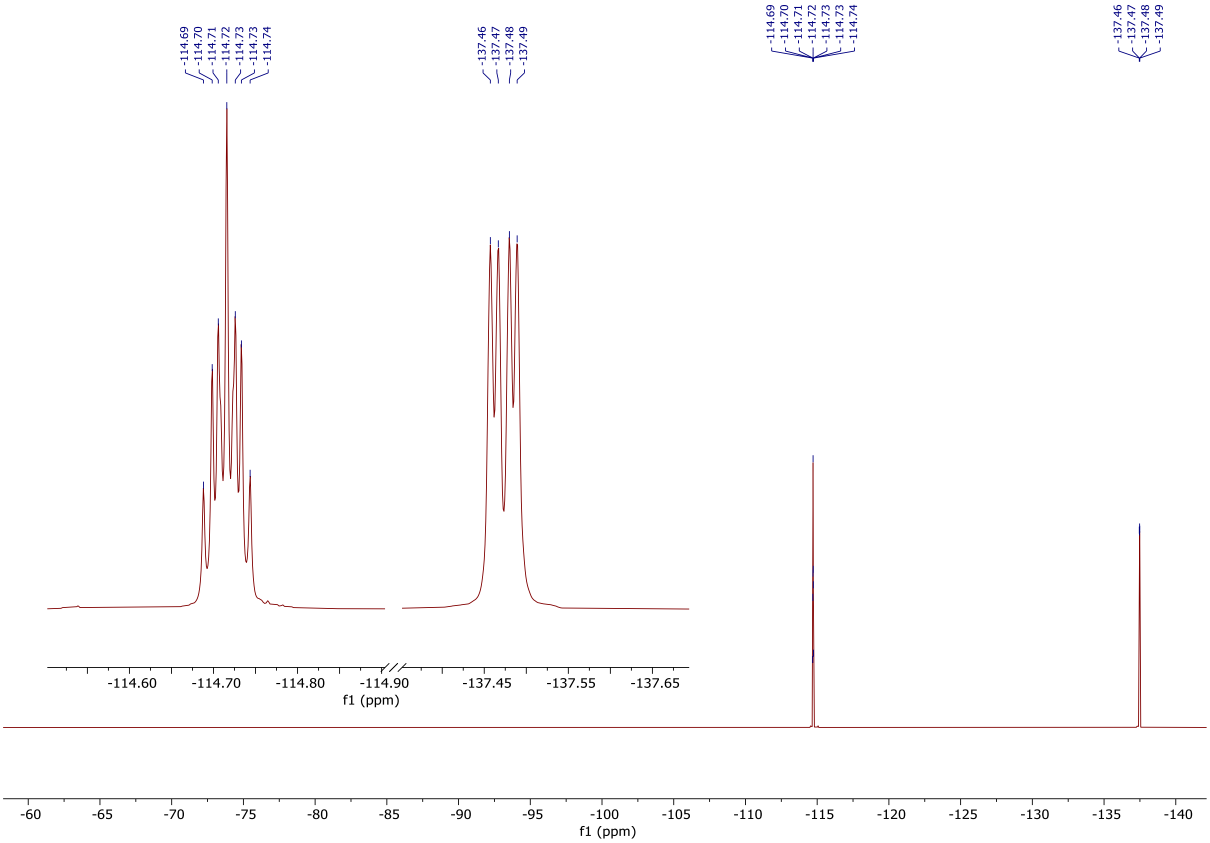

Figure S38: ^{19}F NMR spectrum of compound **2**-F in $CDCl_3$.

2-[(1E)-[(4-fluorophenyl)imino]methyl]-6-methylphenol (2-Me)

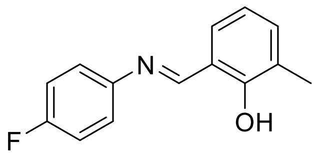

Following general procedure A, 3-*tert*-butyl-2-hydroxybenzaldehyde (5 mmol, 890 mg, 1.0 eq), 4-fluoroaniline (6 mmol, 667 mg, 1.2 eq) were dissolved in 10 mL MeOH. Two droplets of acetic acid were added and the reaction mixture was refluxed for 6 h. Upon cooling down, the volatiles were removed in vacuo. To the residual crude product was added 100 µL acetic acid and the resulting mixture was passed through a 10 cm silica plug (eluting with 100% DCM). After evaporation of the volatiles, the title compound was obtained as an orange oil which slowly crystallised to give a yellow solid (881 mg, 77%). Mp 40 – 42 °C. Spectroscopic data matches the literature values.[14] ^{1}H NMR (601 MHz, $CDCl_3$) δ 13.40 (s, 1H), 8.61 (s, 1H), 7.31 – 7.26 (m, 4H), 7.17 – 7.10 (m, 2H), 6.89 (t, J = 7.5 Hz, 1H), 2.35 (s, 3H). ^{13}C NMR (151 MHz, $CDCl_3$) δ 162.76, 161.75 (d, J = 246.2 Hz), 159.48, 144.84 (d, J = 3.0 Hz), 134.36, 130.11, 126.42, 122.70 (d, J = 8.3 Hz), 118.82, 118.48, 116.35 (d, J = 22.7 Hz), 15.65. ^{19}F NMR (471 MHz, $CDCl_3$) δ -115.74 (ddd, J = 13.1, 8.3, 4.9 Hz). HRMS (ESI+): m/z calcd. for $C_{14}H_{13}FNO$ $[M+H]^+$: 230.0976, found 230.1047.

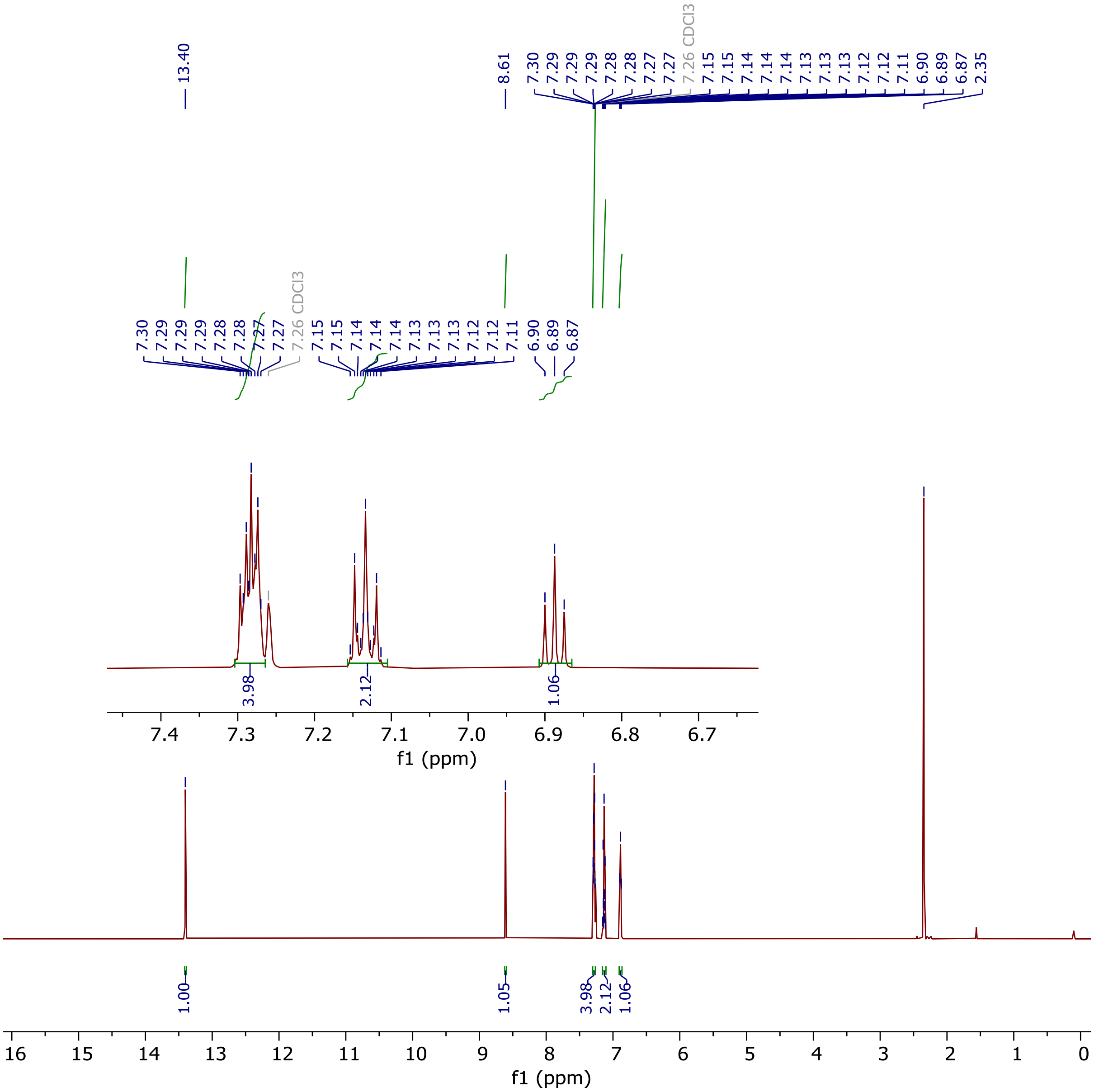

Figure S39: ^{1}H NMR spectrum of compound **2**-Me in $CDCl_3$.

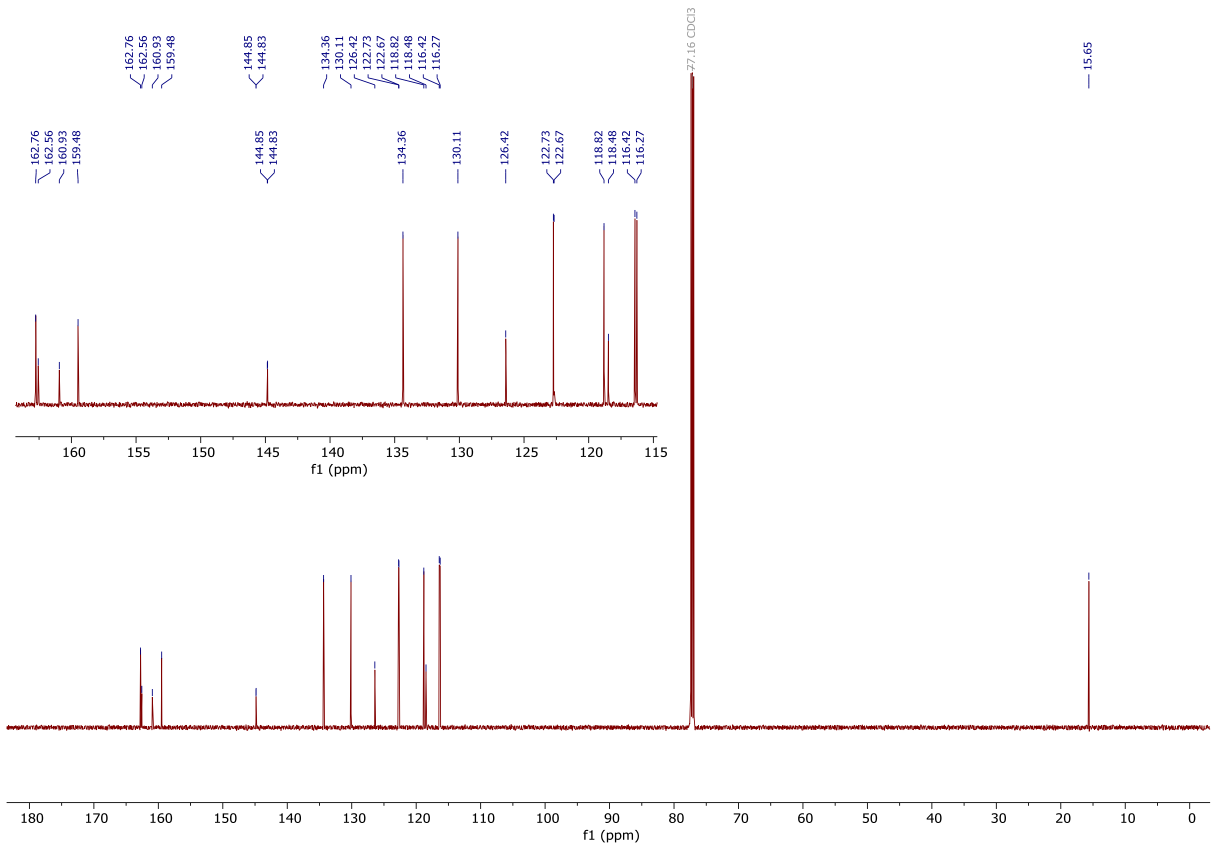

Figure S40: ^{13}C NMR spectrum of compound **2**-Me in $CDCl_3$.

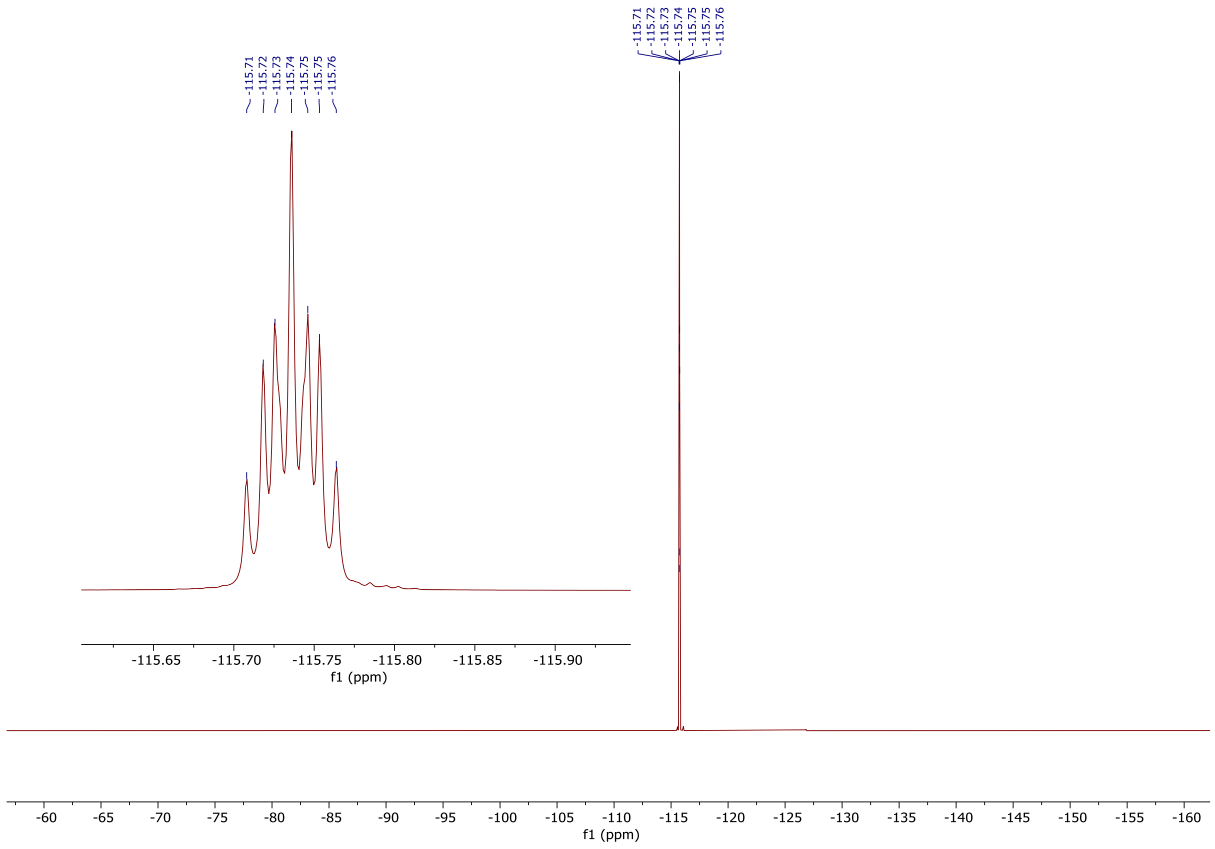

Figure S41: ^{19}F NMR spectrum of compound **2**-Me in $CDCl_3$.

General procedure B: Synthesis of amines **3**-R

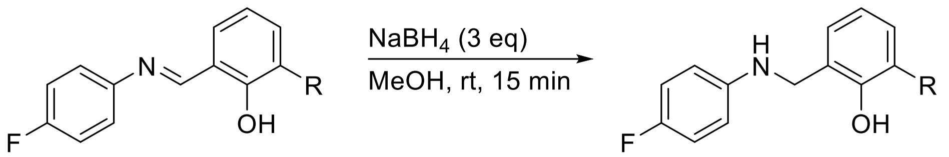

In a 25 mL flask, a suspension of the appropriate imine **2-R** in methanol (5 mL) was heated to 40 °C. Under vigorous stirring, $NaBH_4$ (8 eq) was added in portions over a period of 5 min. In all cases, the full consumption of the starting material was confirmed by TLC (100% DCM) after 5 – 10 minutes of stirring at 40 °C. After the gas evolution was ceased, the reaction mixture was poured into 20 mL water and extracted with DCM (3 x 25 mL). The combined organic phase was washed with brine and dried over $MgSO_4$. After removing of the volatiles *in vacuo*, the desired amines were purified by recrystallization or column chromatography.

2-{[(4-fluorophenyl)amino]methyl}-6-methoxyphenol (3-OMe)

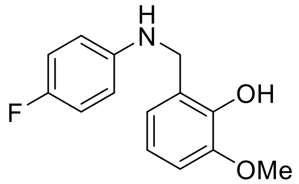

Prepared according to general procedure B using **2-OMe** (3.0 mmol, 736 mg) and $NaBH_4$ (6.0 mmol, 8 eq, 228 mg). After aqueous workup and evaporation of the volatiles, the crude product was passed through a 10 cm silica plug (eluent 50:1 DCM:MeOH). After evaporation of the volatiles, the title compound was obtained as a pale-yellow oil which slowly crystallized to give a colourless solid (608 mg, 82%). Mp 69 – 71 °C. ^{1}H NMR (601 MHz, $CDCl_3$) δ 6.91 – 6.85 (m, 3H), 6.81 (d, J = 4.8 Hz, 2H), 6.67 – 6.61 (m, 2H), 6.38 (br s, 1H), 4.34 (s, 2H), 4.10 (br s, 1H), 3.89 (s, 1H). ^{13}C NMR (151 MHz, $CDCl_3$) δ 156.43 (d, J = 235.9 Hz), 146.93, 144.49, 144.28, 124.58, 121.30, 119.76, 115.76 (d, J = 22.2 Hz), 114.82 (d, J = 7.3 Hz), 110.18, 56.19, 45.09. ^{19}F NMR (471 MHz, $CDCl_3$) δ -127.16 (tt, J = 8.6, 4.4 Hz). HRMS (ESI+): m/z calcd. for $C_{14}H_{15}FNO_2$ $[M+H]^+$: 248.1081, found 248.1083.

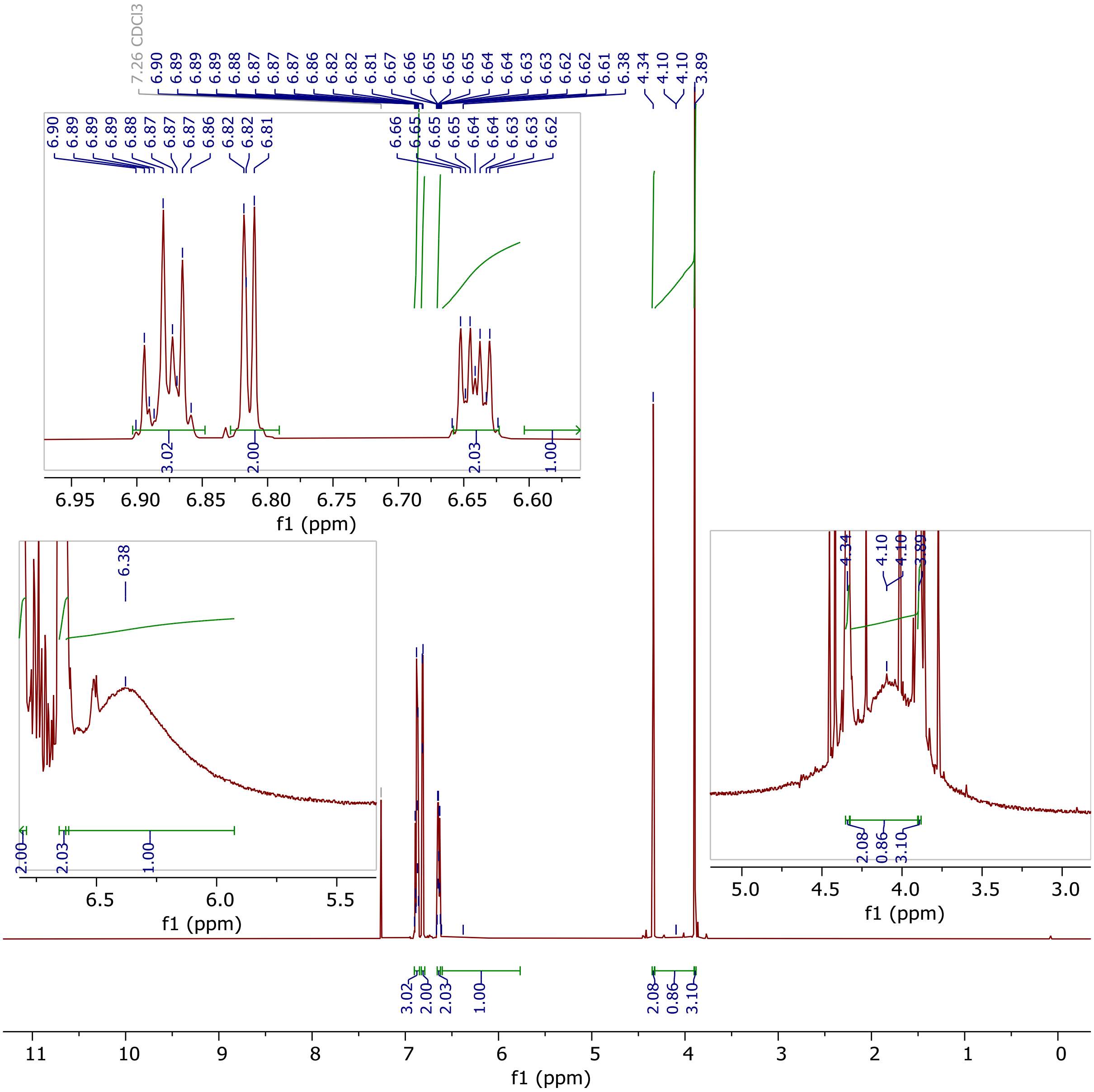

Figure S42: ^{1}H NMR spectrum of compound **3**-OMe in $CDCl_3$.

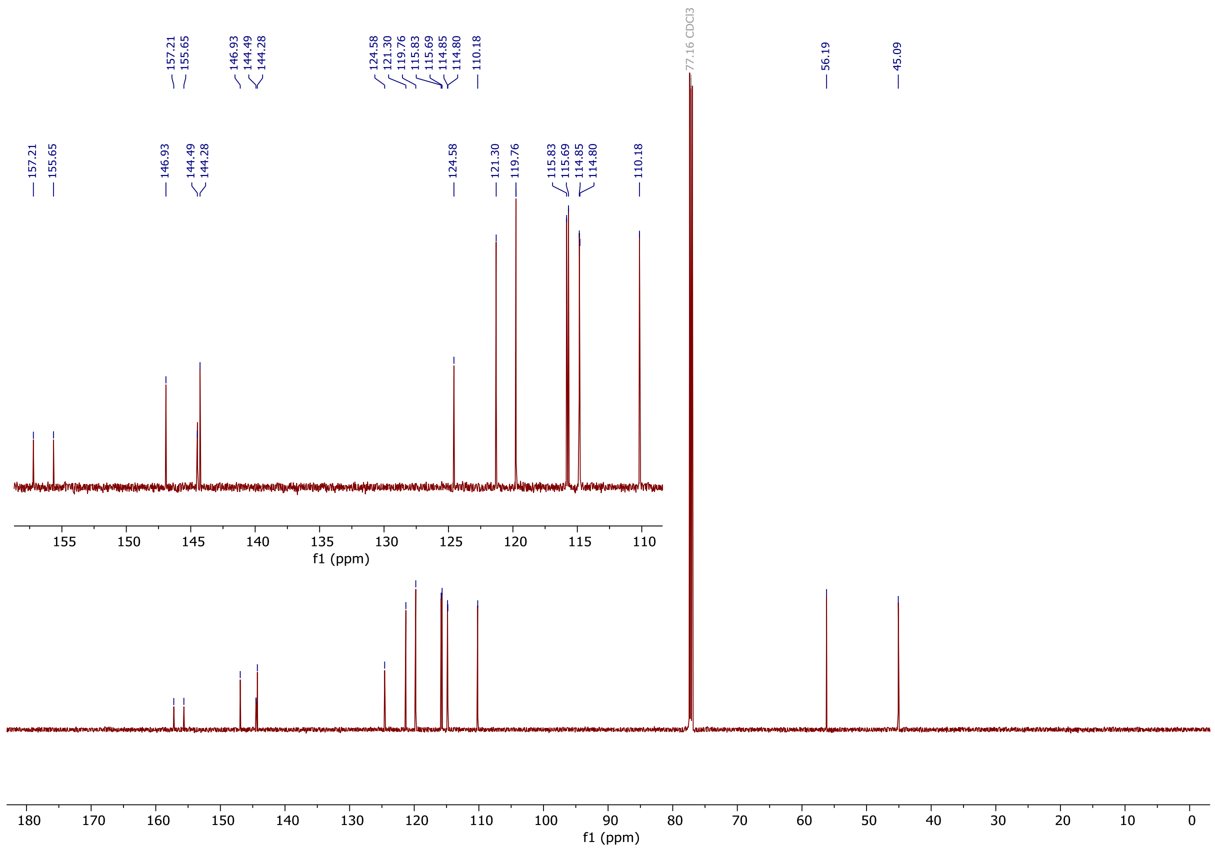

Figure S43: ^{13}C NMR spectrum of compound **3**-OMe in $CDCl_3$.

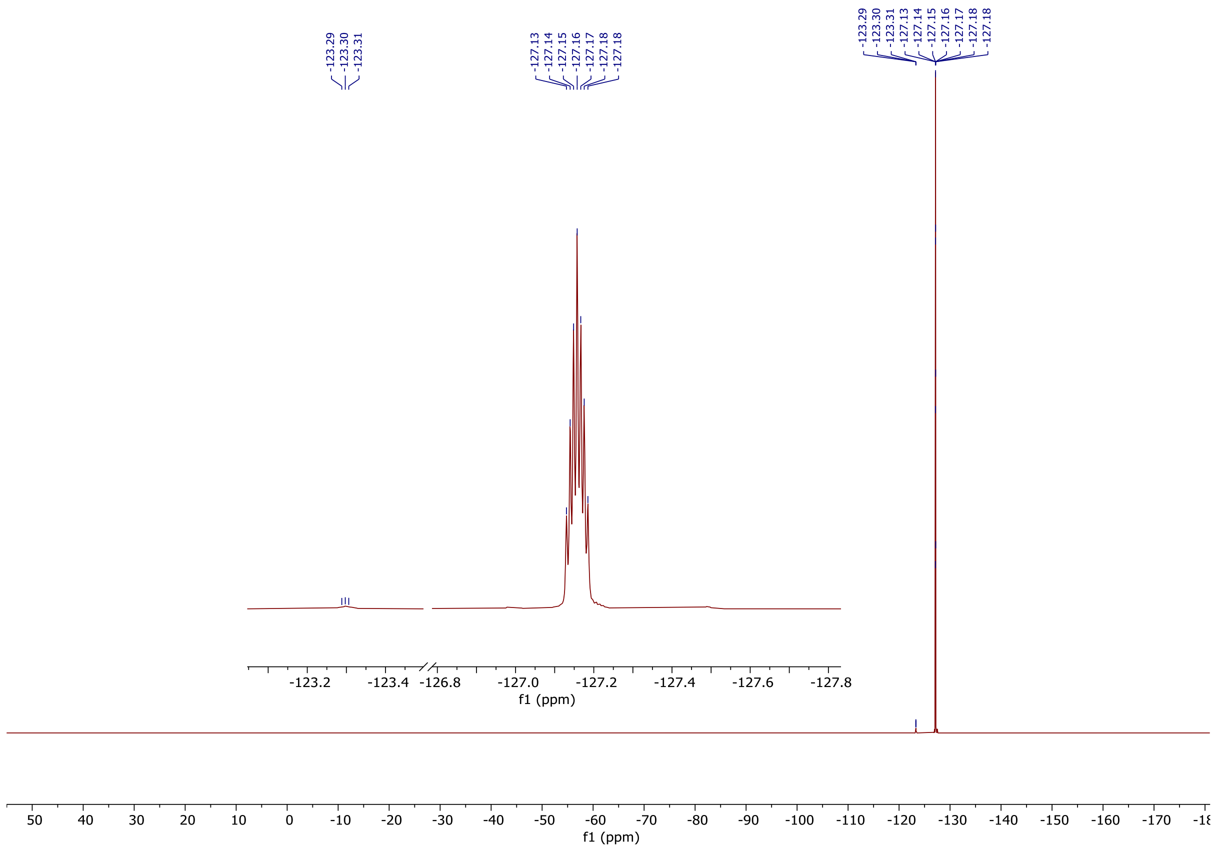

Figure S44: ^{19}F NMR spectrum of compound **3**-OMe in $CDCl_3$.

3-{[(4-fluorophenyl)amino]methyl}-2-hydroxybenzonitrile (3-CN)

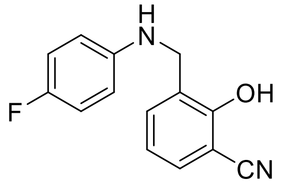

Prepared according to general procedure B using **2-CN** (3.0 mmol, 720 mg) and $NaBH_4$ (6.0 mmol, 8 eq, 228 mg). After aqueous workup and evaporation of the volatiles, the crude product was passed through a 10 cm silica plug (eluent 50:1 DCM:MeOH). After evaporation of the volatiles, the title compound was obtained as a pale-yellow oil which slowly crystallized to give a colourless solid (529 mg, 73% yield). Mp 108 – 110 °C. ^{1}H NMR (601 MHz, $CDCl_3$) δ 10.08 (br s, 1H), 7.46 (d, J = 7.7 Hz, 1H), 7.34 (dd, J = 7.6, 1.6 Hz, 1H), 6.98 – 6.94 (m, 2H), 6.92 (t, J = 7.7 Hz, 1H), 6.84 – 6.78 (m, 2H), 4.46 (s, 2H). ^{13}C NMR (151 MHz, $CDCl_3$) δ 160.00, 158.51 (d, J = 240.7 Hz), 142.26 (d, J = 2.5 Hz), 132.90, 132.69, 123.63, 120.24, 118.21 (d, J = 7.8 Hz), 116.48, 116.28 (d, J = 22.7 Hz), 101.14, 49.96. ^{19}F NMR (471 MHz, $CDCl_3$) δ -121.78 (tt, J = 8.4, 4.5 Hz). HRMS (ESI+): m/z calcd. for $C_{14}H_{12}FN_2O$ [M+H]$^+$: 243.0928, found 243.0934.

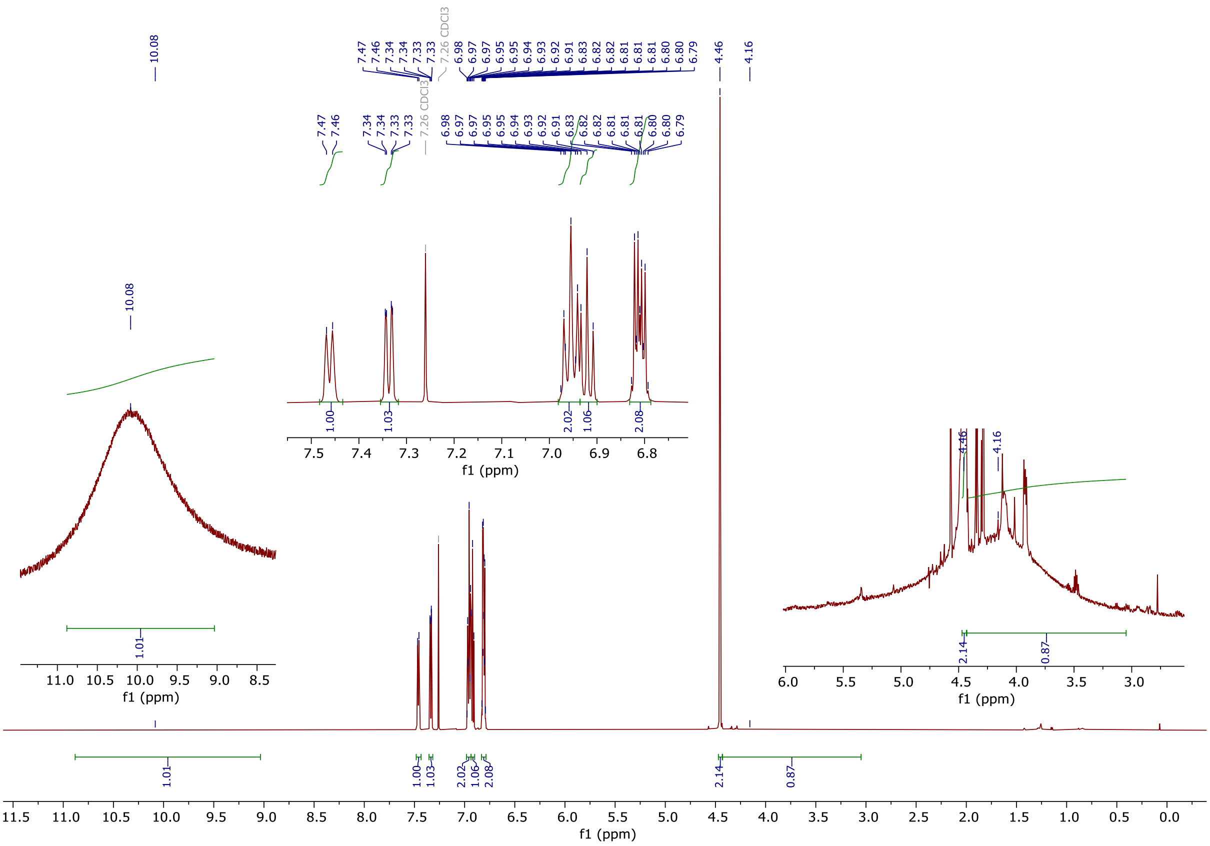

Figure S45: ^{1}H NMR spectrum of compound **3**-CN in $CDCl_3$.

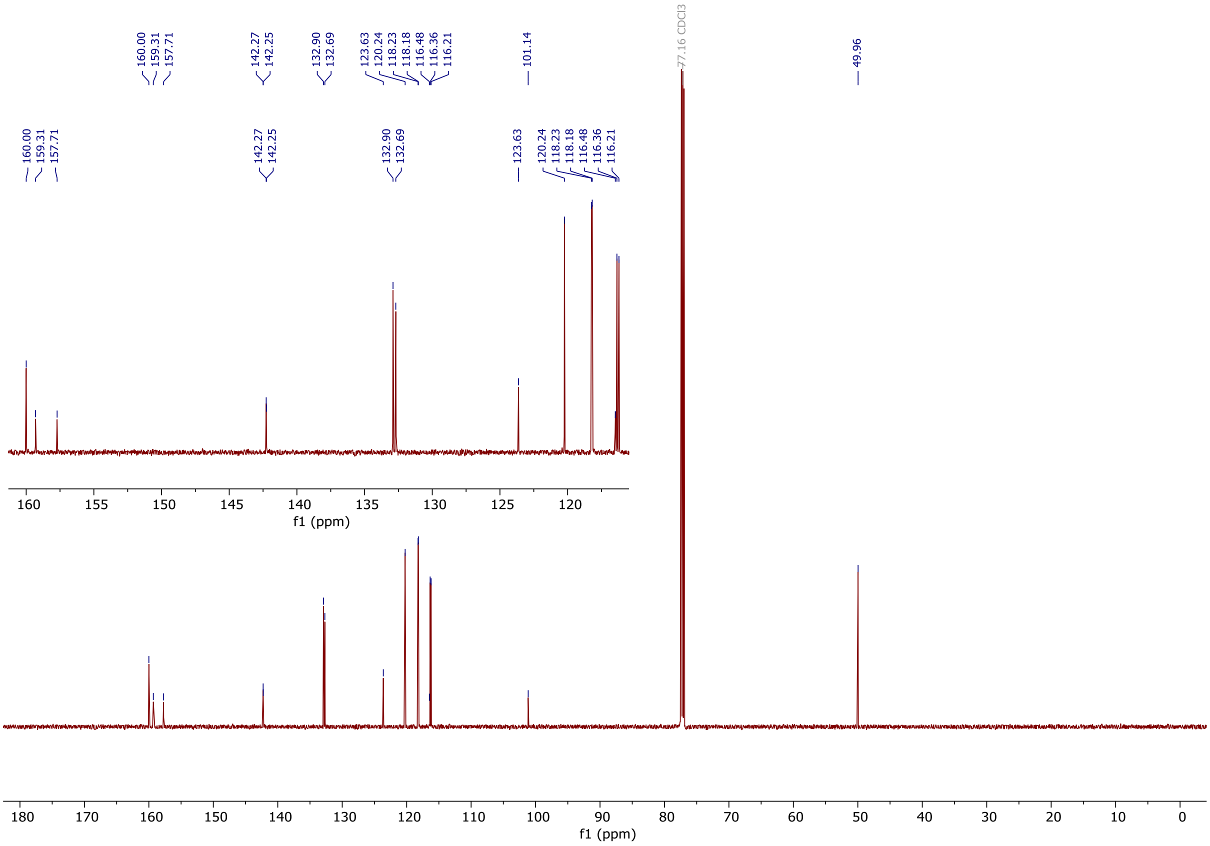

Figure S46: ^{13}C NMR spectrum of compound **3**-CN in $CDCl_3$.

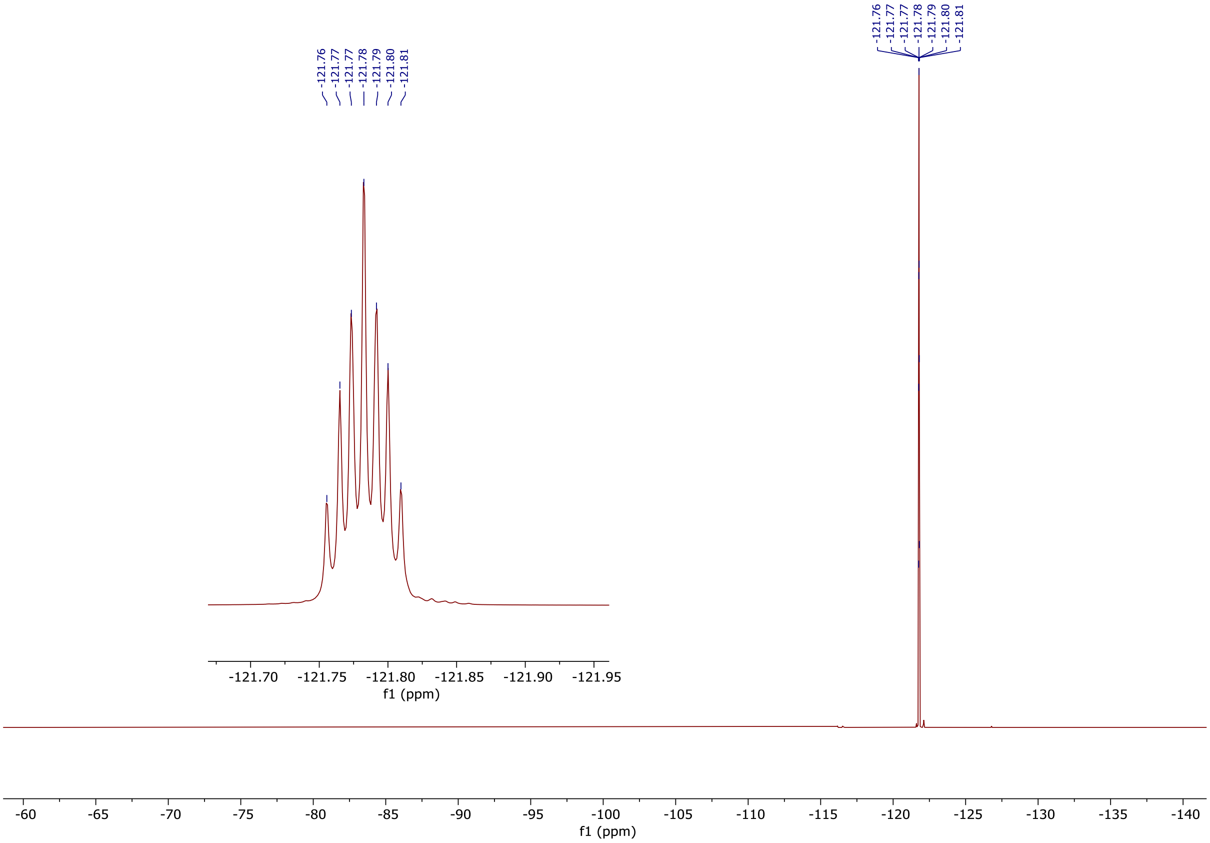

Figure S47: ^{19}F NMR spectrum of compound **3**-CN in $CDCl_3$.

2-{[(4-fluorophenyl)amino]methyl}-6-(trifluoromethyl)phenol (3-CF3)

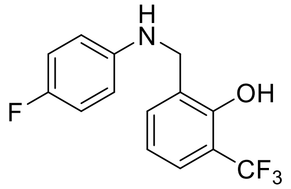

Prepared according to general procedure B using **2-CF3** (3.0 mmol, 849 mg) and $NaBH_4$ (6.0 mmol, 8 eq, 228 mg). After aqueous workup and evaporation of the volatiles, the crude product was passed through a 10 cm silica plug (eluent 50:1 DCM:MeOH). After evaporation of volatiles, the title compound was obtained as a pale-yellow oil, which slowly crystallized to give the title compound as an off-white solid (657 mg, 77% yield). Mp 48 – 50 °C. ^{1}H NMR (601 MHz, $CDCl_3$) δ 9.78 (br s, 1H), 7.51 (dd, J = 7.9, 1.6 Hz, 1H), 7.31 (dd, J = 7.6, 1.6 Hz, 1H), 6.99 – 6.95 (m, 1H), 6.93 (t, J = 7.8 Hz, 1H), 6.87 – 6.80 (m, 2H), 4.45 (s, 2H), 3.96 (br s, 1H). ^{13}C NMR (151 MHz, $CDCl_3$) δ 158.41 (d, J = 240.3 Hz), 155.69, 142.51, 132.22, 126.70 (d, J = 5.0 Hz), 123.96 (d, J = 272.4 Hz), 123.94, 119.42, 118.11 (d, J = 7.8 Hz), 118.02 (q, J = 30.5 Hz), 116.24 (d, J = 22.5 Hz), 50.04. ^{19}F NMR (471 MHz, $CDCl_3$) δ -62.17, -122.15 (tt, J = 8.4, 4.5 Hz). HRMS (ESI+): m/z calcd. for C14H10F4NO $[M+H]^+$: 284.0693, found 284.0708.

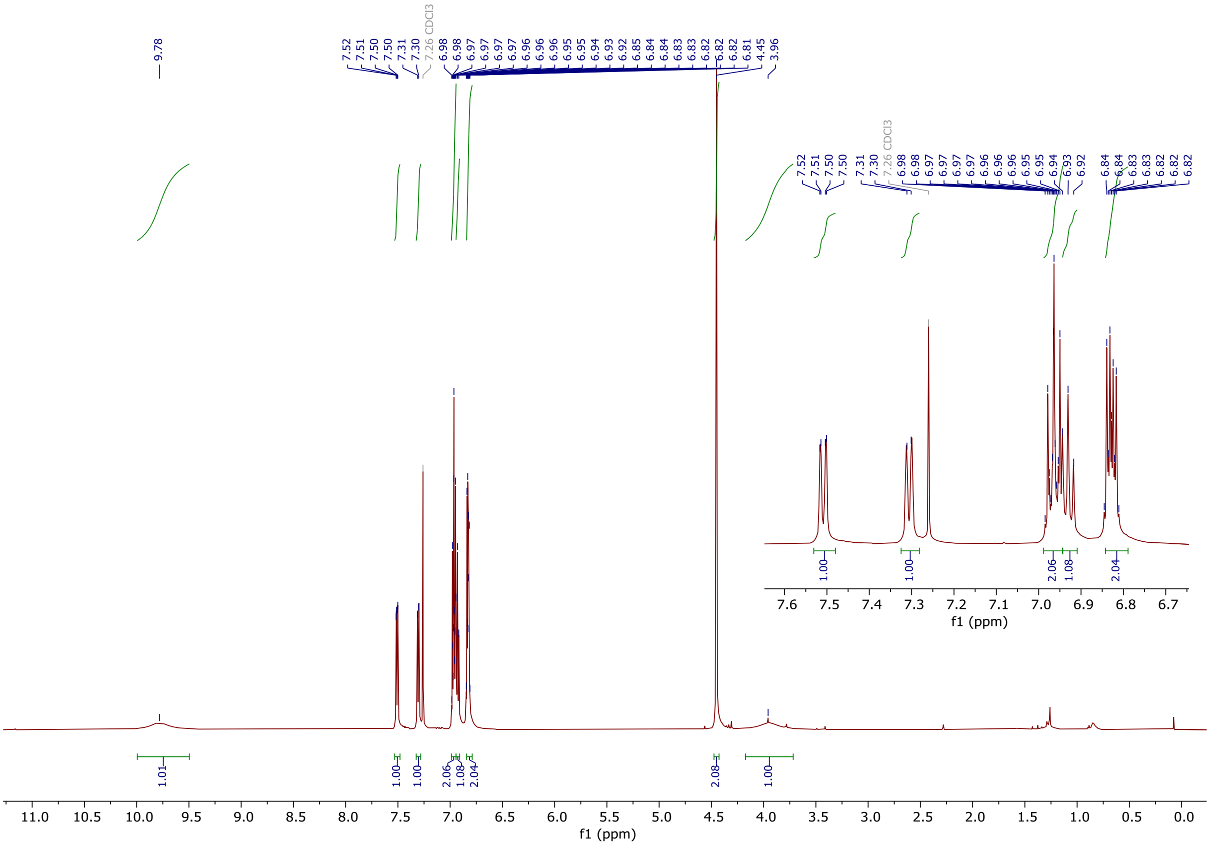

Figure S48: ^{1}H NMR spectrum of compound **3**-CF_3 in $CDCl_3$.

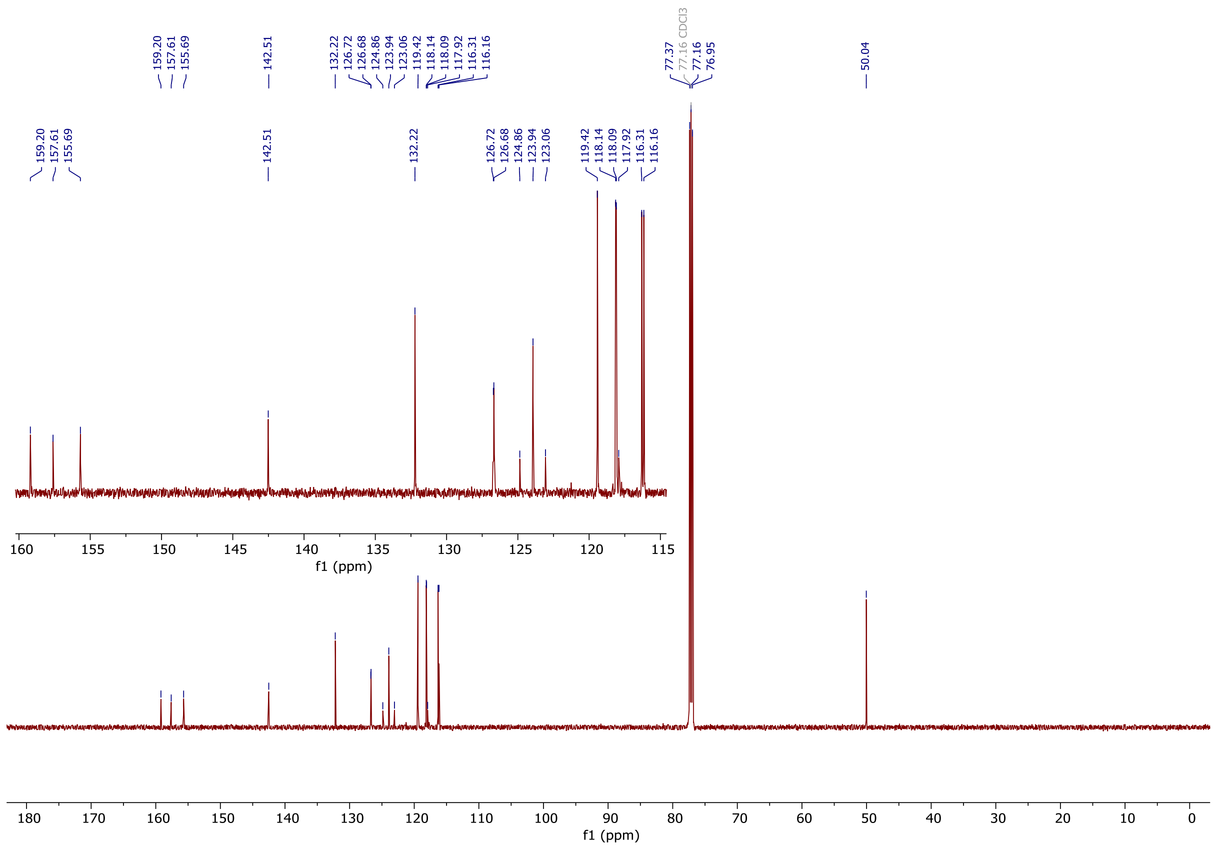

Figure S49: ^{13}C NMR spectrum of compound **3**-CF_3.

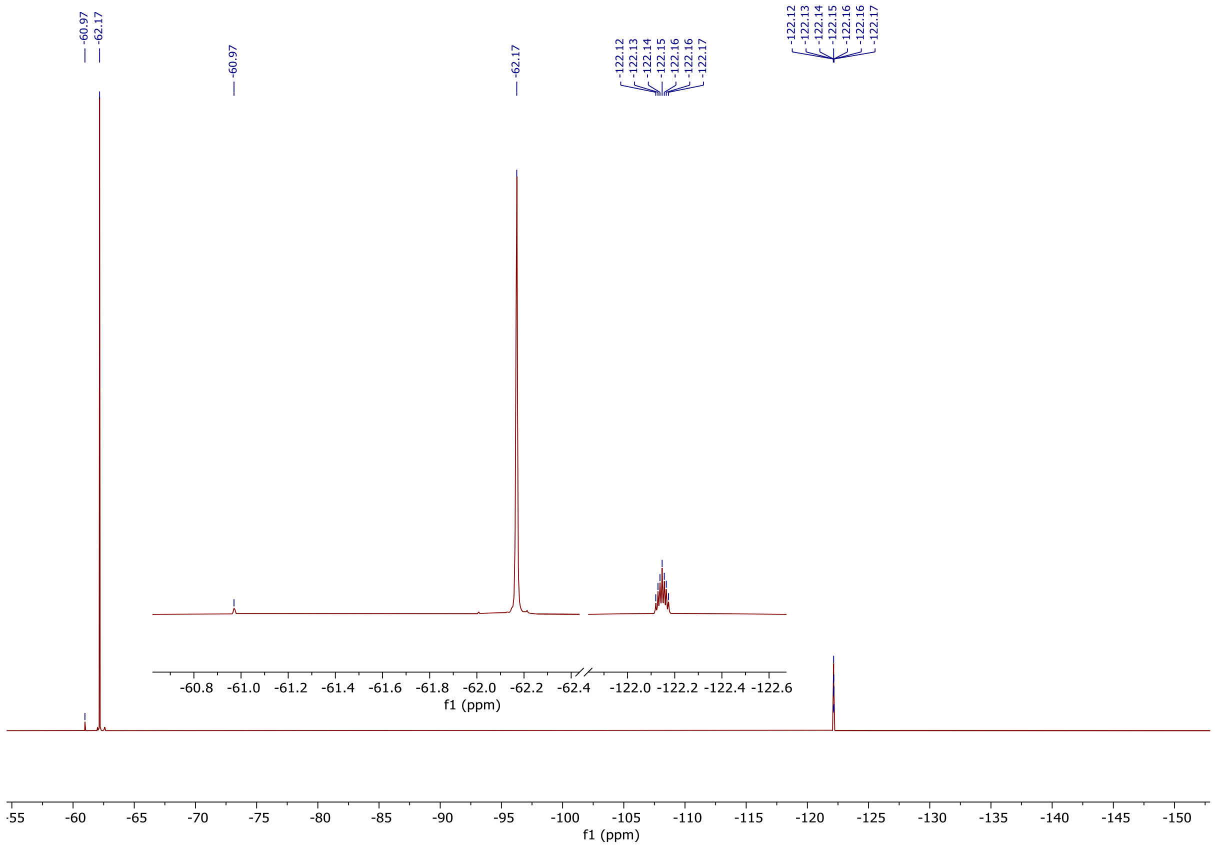

Figure S50: ^{19}F NMR spectrum of compound **3**-CF_3 in $CDCl_3$.

N-benzyl-4-fluoroaniline (3-control)

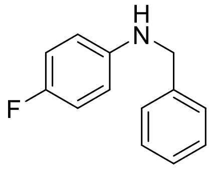

Prepared according to general procedure B using **2-control** (3.0 mmol, 597 mg) and $NaBH_4$ (6.0 mmol, 8 eq, 228 mg). After aqueous workup and evaporation of the volatiles, the crude product was passed through a 10 cm silica plug (eluent 50:1 DCM:MeOH). After evaporation of volatiles, the title compound was obtained as a pale-yellow oil, which slowly crystallised to give the title compound as an off-white solid (404 mg, 67% yield). Mp 34– 36 °C.
^{1}H NMR (601 MHz, $CDCl_3$) δ 7.40 – 7.33 (m, 4H), 7.31 – 7.27 (m, 1H), 6.92 – 6.85 (m, 2H), 6.61 – 6.54 (m, 2H), 4.30 (s, 2H), 3.93 (s, 1H). ^{13}C NMR (151 MHz, $CDCl_3$) δ 156.04 (d, *J* = 234.9 Hz), 144.63 (d, *J* = 1.7 Hz), 139.39, 128.81, 127.63, 127.45, 115.81 (d, *J* = 22.3 Hz), 113.78 (d, *J* = 7.5 Hz), 49.09. ^{19}F NMR (471 MHz, $CDCl_3$) δ -127.92 (tt, *J* = 8.4, 4.4 Hz). HRMS (ESI+): m/z calcd. for $C_{13}H_{13}FN$ $[M+H]^+$: 202.1026, found 202.1022.

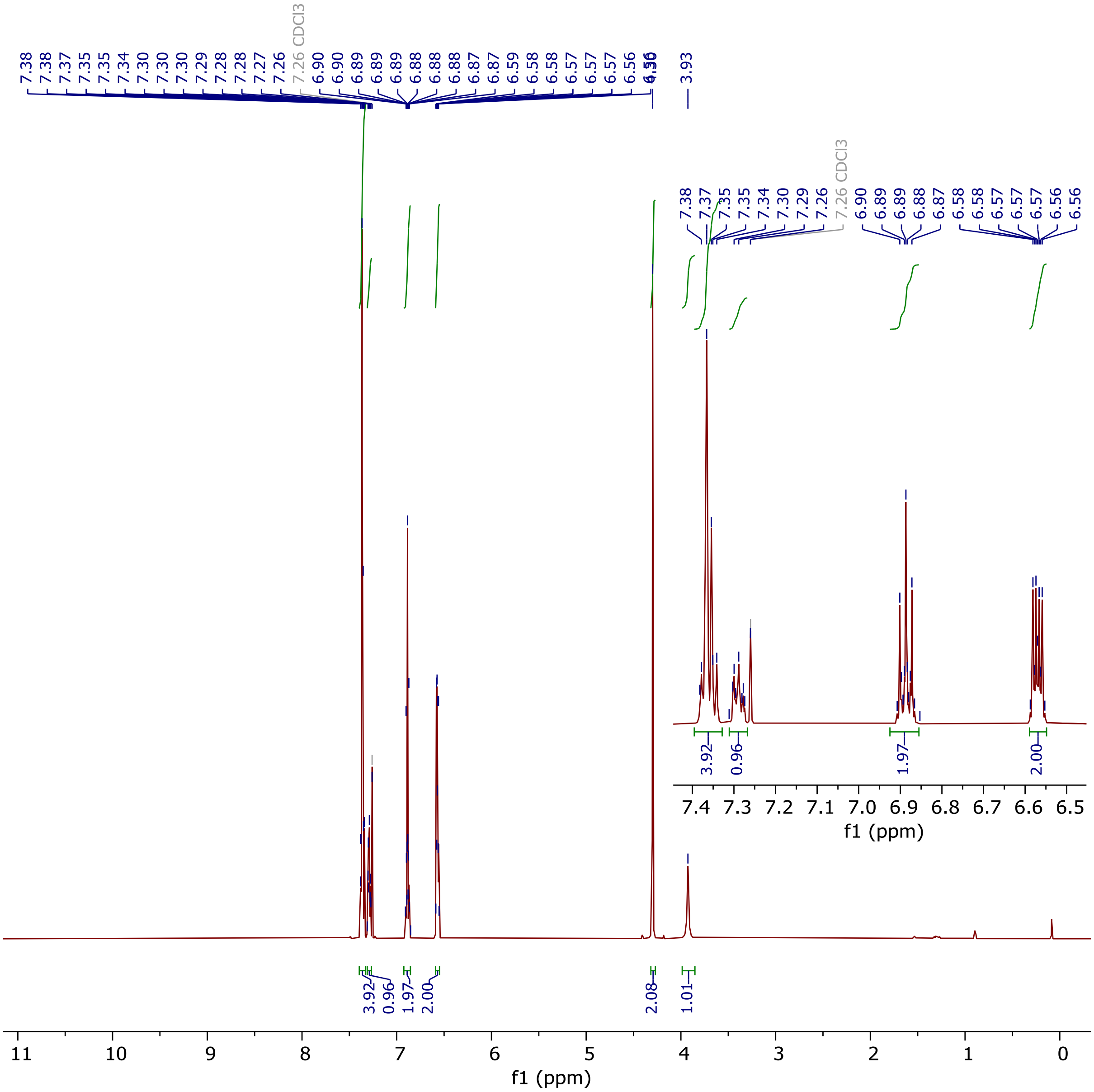

Figure S51: ^{1}H NMR spectrum of compound **3**-control in $CDCl_3$.

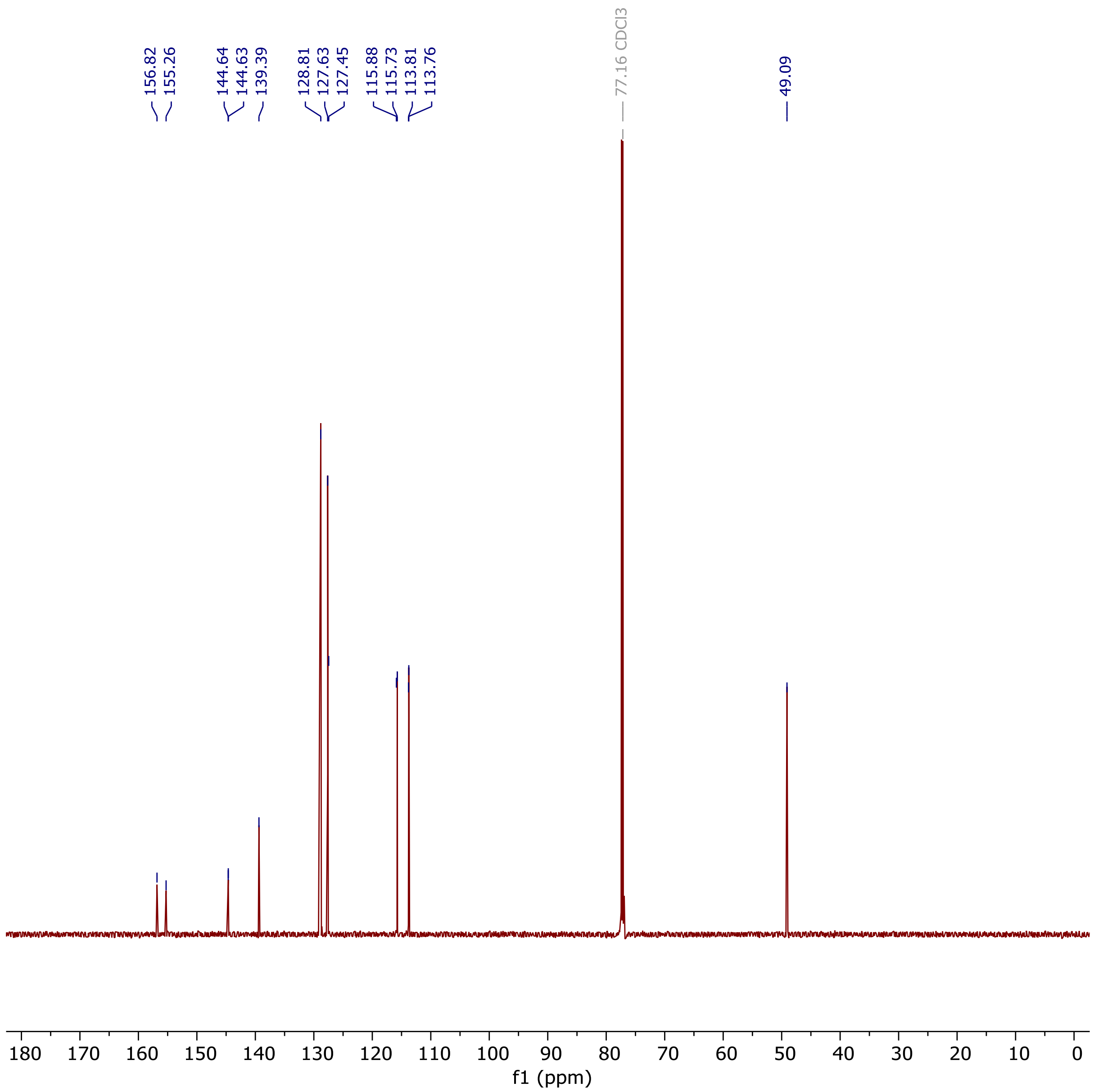

Figure S52: ^{13}C NMR spectrum of compound **3**-control in $CDCl_3$.

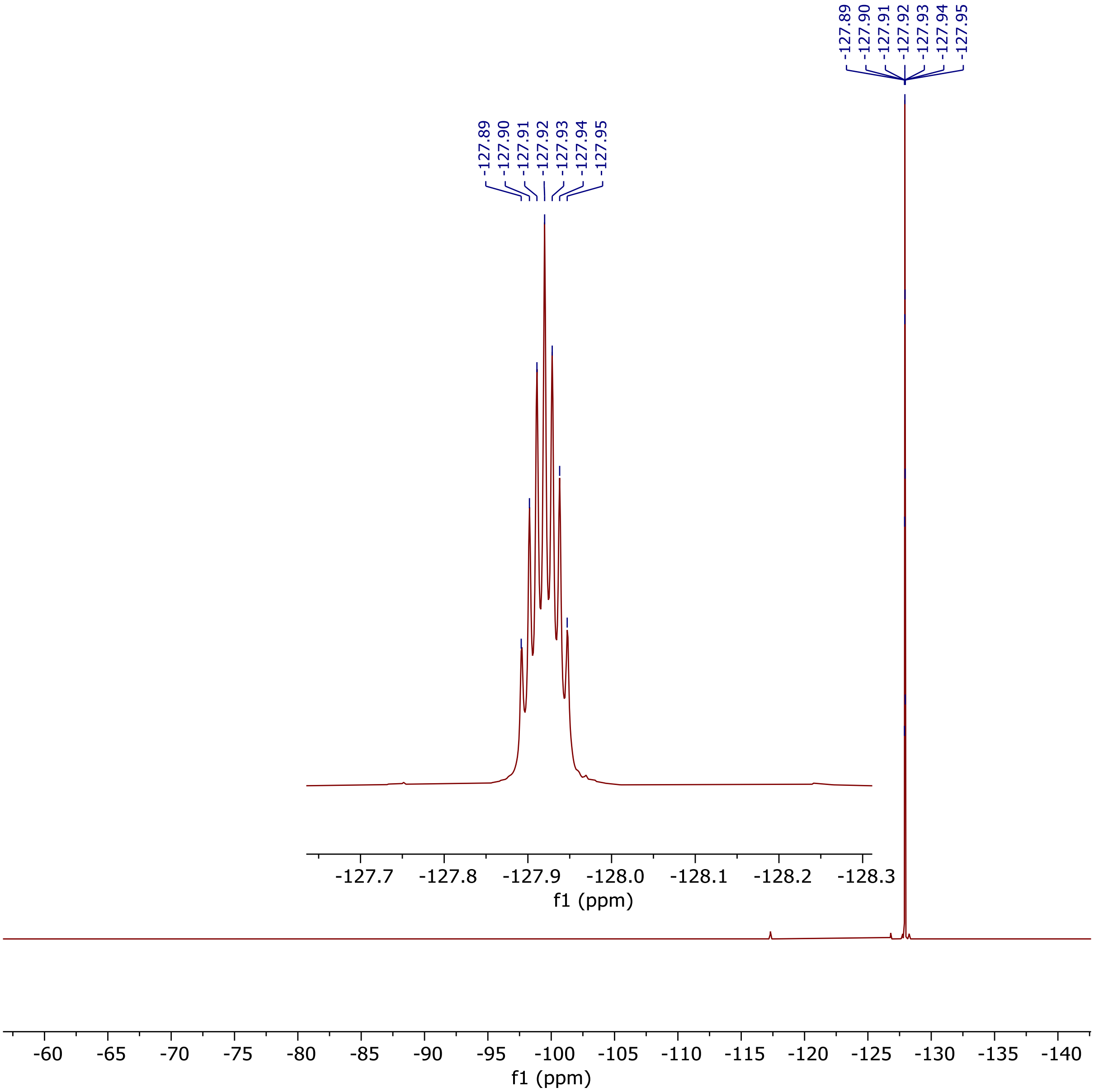

Figure S53: ^{19}F NMR spectrum of compound **3**-control in $CDCl_3$.

2-bromo-6-{[(4-fluorophenyl)amino]methyl}phenol (3-Br)

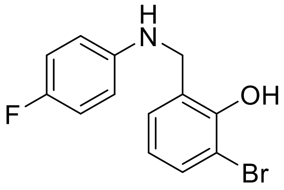

Prepared according to general procedure B using **2-Br** (3.0 mmol, 879 mg) and $NaBH_4$ (6.0 mmol, 8 eq, 228 mg). After the aqueous workup and evaporation of the volatiles, the crude product was passed through a 10 cm silica plug (eluent 50:1 DCM:MeOH). After recrystallization from DCM/Hexane, the title compound was obtained as a colourless solid (787 mg, 89% yield). Mp 84 – 86 °C.

^{1}H NMR (601 MHz, $CDCl_3$) δ 8.18 (s, 1H), 7.44 (dd, J = 8.0, 1.4 Hz, 1H), 7.16 – 7.12 (m, 2H), 6.96 – 6.88 (m, 3H), 6.77 (t, J = 7.8 Hz, 1H), 6.74 – 6.70 (m, 3H), 4.39 (d, J = 4.6 Hz, 3H), 4.01 (s, 1H). ^{13}C NMR (151 MHz, $CDCl_3$) δ 157.46 (d, J = 238.3 Hz), 152.46, 143.31 (d, J = 2.2 Hz), 132.06, 128.08, 125.08, 121.27, 116.48 (d, J = 7.6 Hz), 116.03 (d, J = 22.5 Hz), 110.88, 48.02. ^{19}F NMR (471 MHz, $CDCl_3$) δ -124.43 (tt, J = 8.5, 4.4 Hz). HRMS (ESI+): m/z calcd. for $C_{13}H_{12}BrFNO$ $[M+H]^+$: 296.0081, found 296.0089.

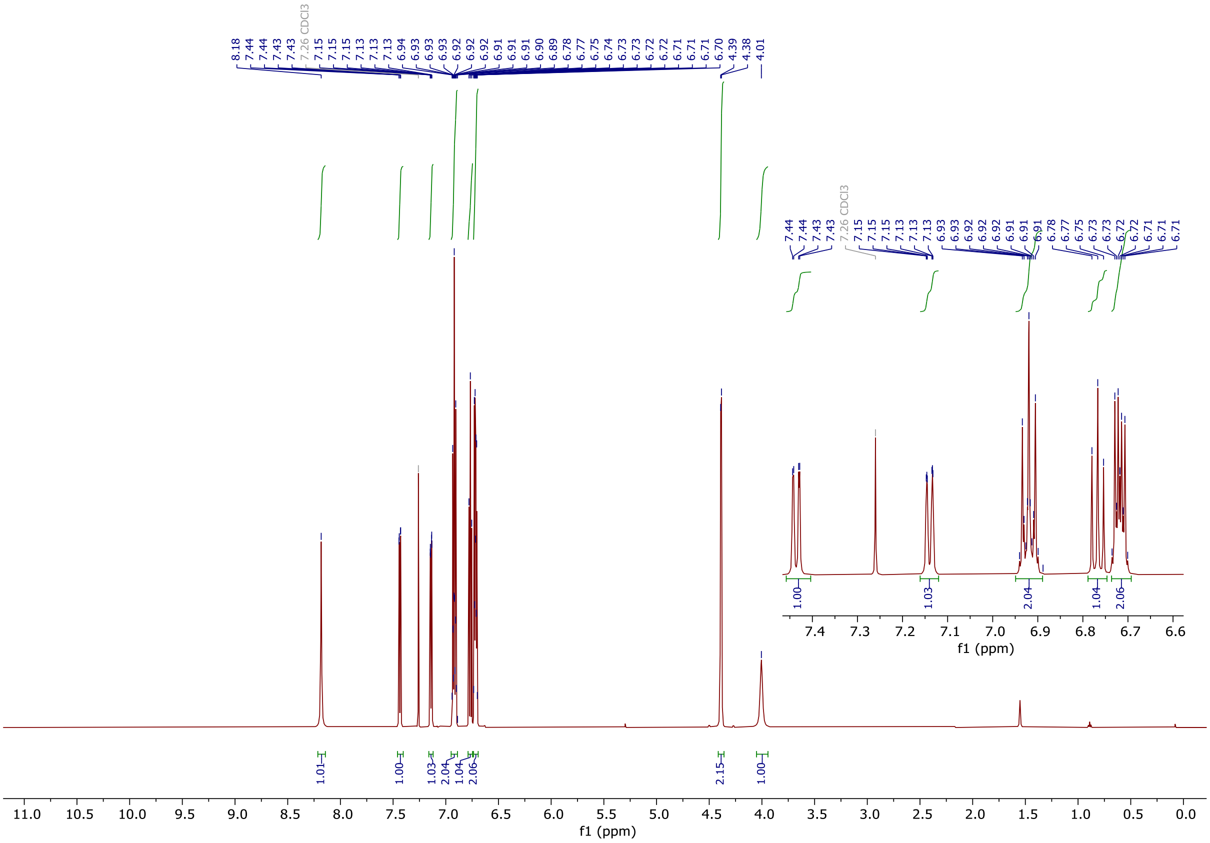

Figure S54: ^{1}H NMR spectrum of compound **3**-Br in $CDCl_3$.

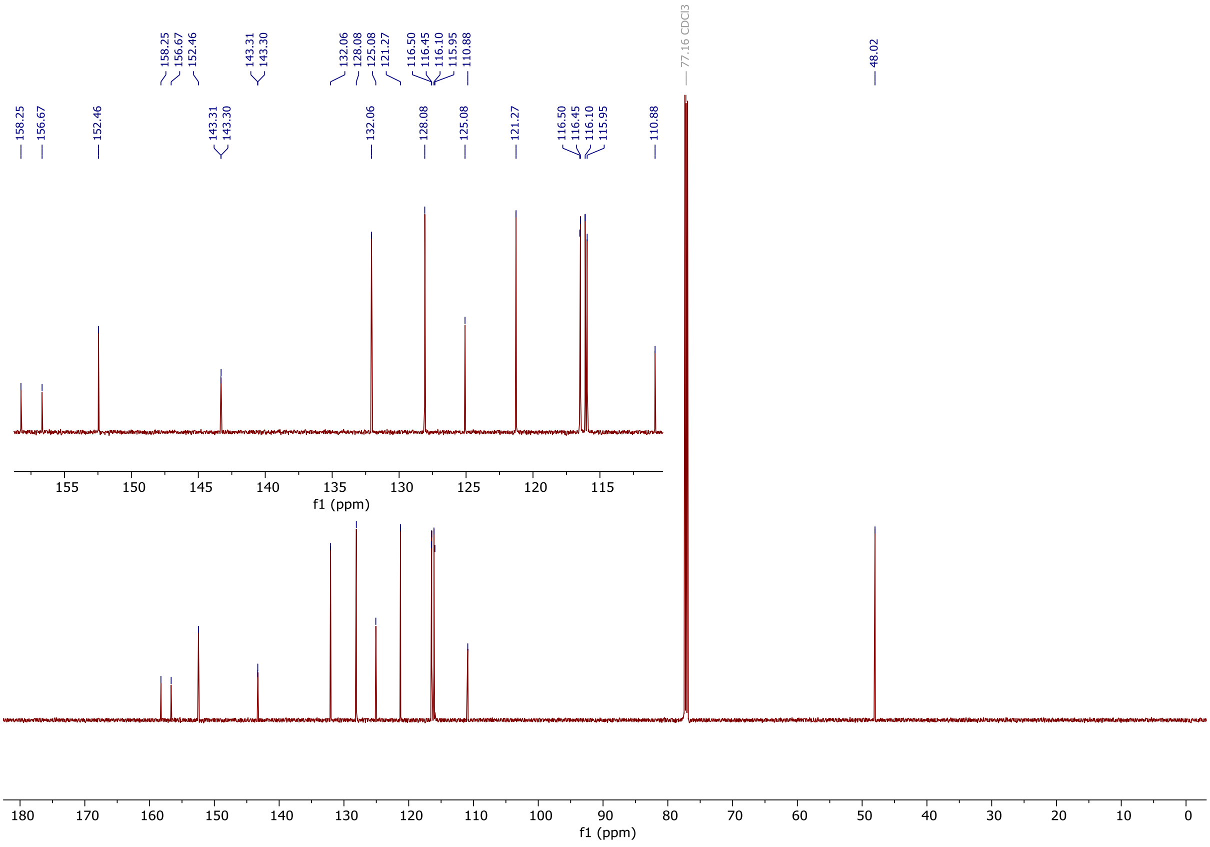

Figure S55: ^{13}C NMR spectrum of compound **3**-Br in $CDCl_3$.

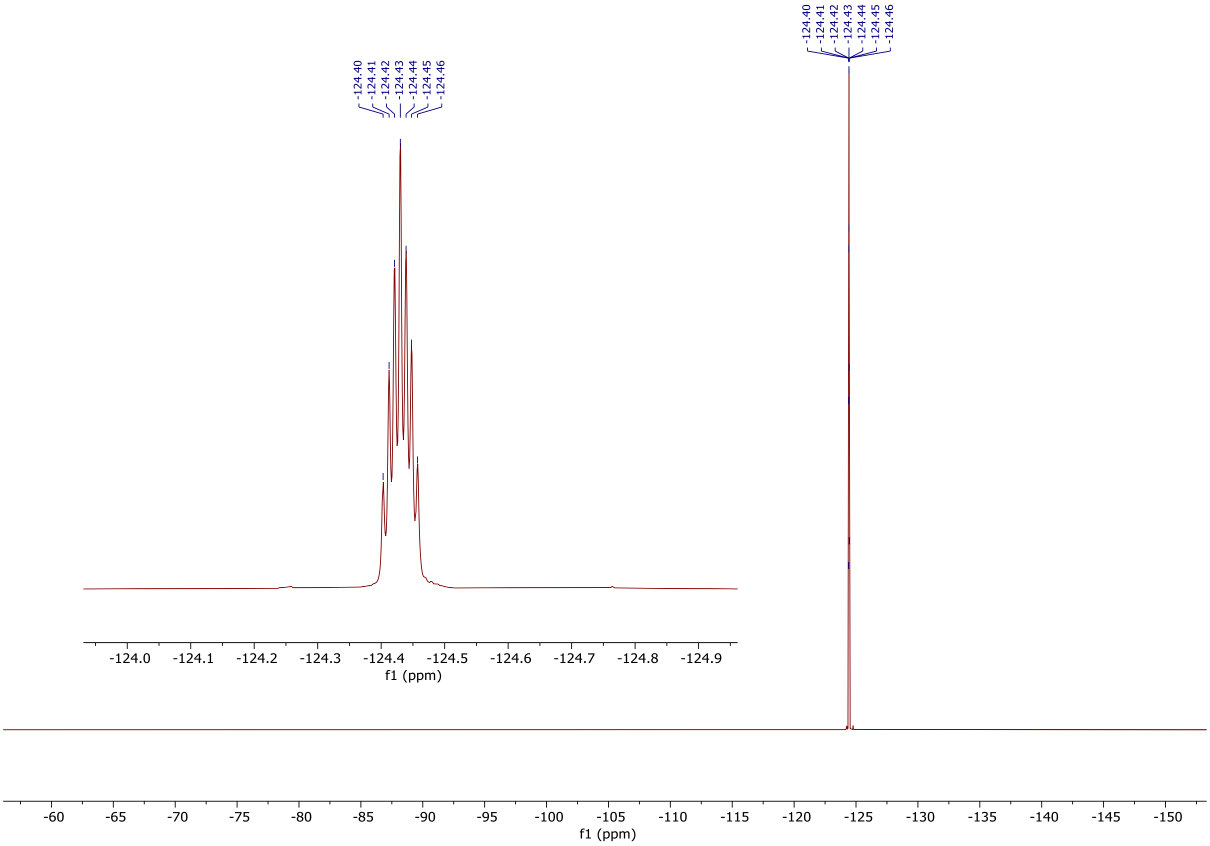

Figure S56: ^{19}F NMR spectrum of compound **3**-Br in $CDCl_3$.

2-{[(4-fluorophenyl)amino]methyl}-6-nitrophenol (3-NO$_2$)

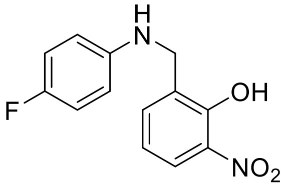

Prepared according to general procedure B using **2-NO$_2$** (3.0 mmol, 780 mg) and $NaBH_4$ (6.0 mmol, 8 eq, 228 mg). After aqueous workup and evaporation of the volatiles, the crude product was passed through a 10 cm silica plug (eluent 50:1 DCM:MeOH). After evaporation of volatiles, the title compound was obtained as a red oil, which slowly crystallised to give title compound as an orange solid (557 mg, 71% yield). Mp 70 – 72 °C. ^{1}H NMR (601 MHz, $CDCl_3$) δ 11.01 (s, 1H), 8.03 (dd, *J* = 8.5, 1.6 Hz, 2H), 7.62 (dt, *J* = 7.4, 0.7 Hz, 3H), 6.94 (dd, *J* = 8.4, 7.5 Hz, 2H), 6.91 – 6.84 (m, 4H), 6.59 – 6.52 (m, 4H), 4.44 (s, 3H), 4.17 (s, 1H). ^{13}C NMR (151 MHz, $CDCl_3$) δ 156.22 (d, *J* = 235.6 Hz), 153.23, 143.93 (d, *J* = 1.7 Hz), 136.27, 133.78, 130.26, 123.97, 119.79, 115.90 (d, *J* = 22.4 Hz), 114.06 (d, *J* = 7.5 Hz), 43.53. ^{19}F NMR (471 MHz, $CDCl_3$) δ -127.31 (tt, *J* = 8.5, 4.4 Hz). HRMS (ESI+): m/z calcd. for $C_{13}H_{12}FN_2O_3$ $[M+H]^+$: 263.0827, found 263.0843.

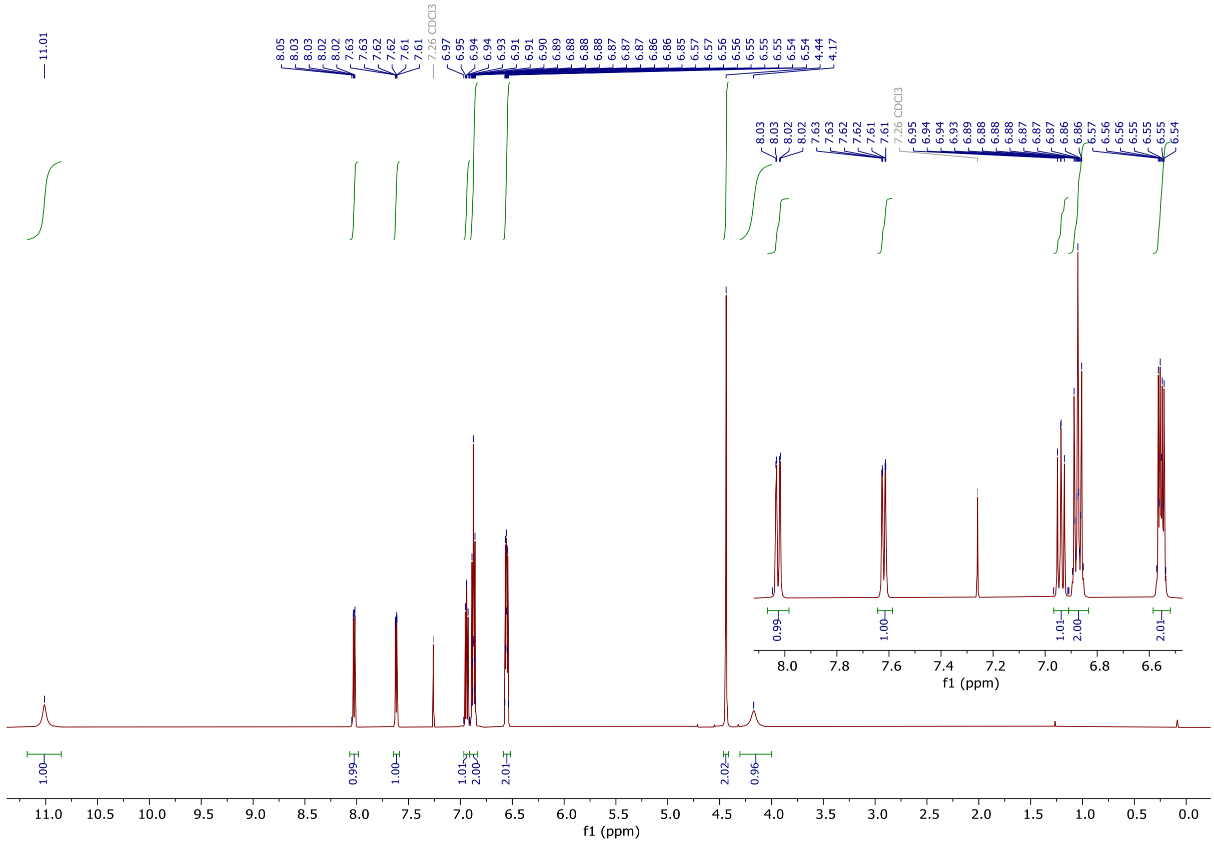

Figure S57: ^{1}H NMR spectrum of compound **3**-NO_2 in $CDCl_3$.

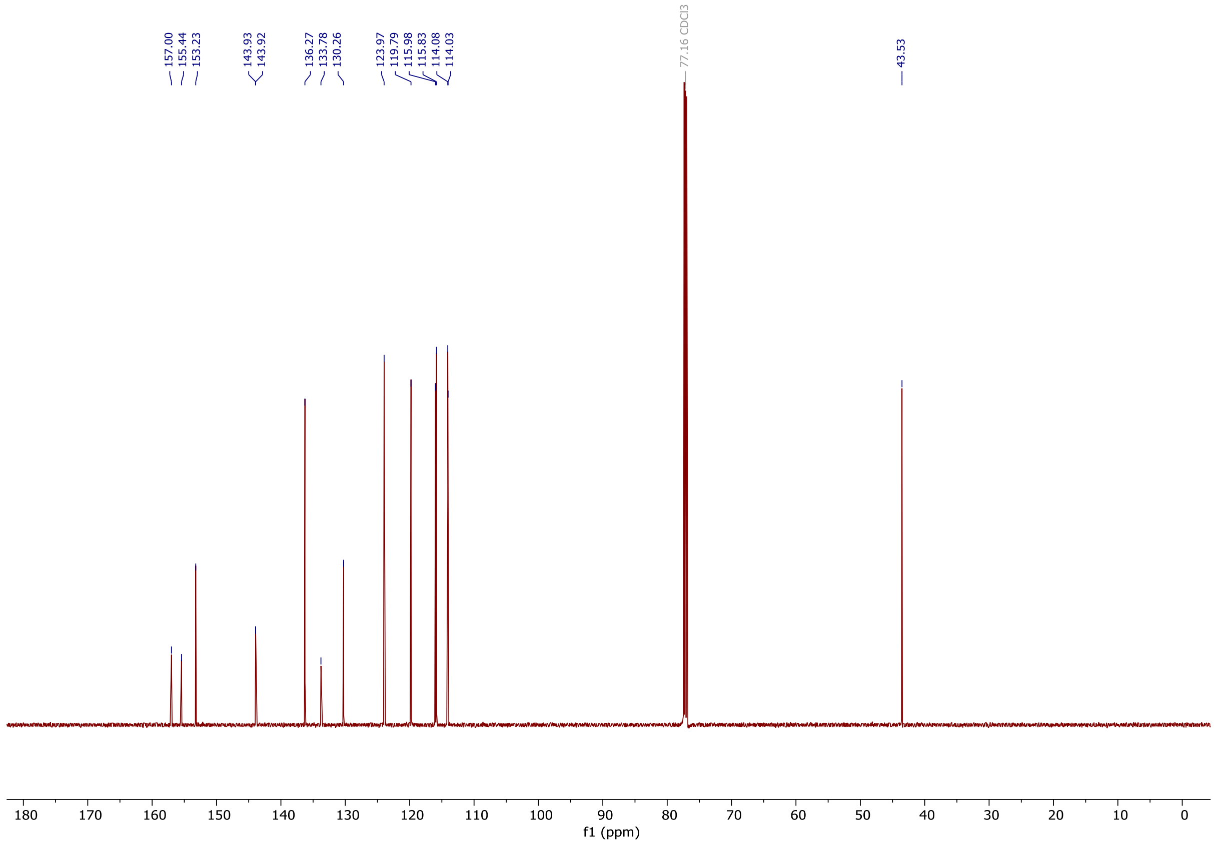

Figure S58: ^{13}C NMR spectrum of compound **3**-NO_2 in $CDCl_3$.

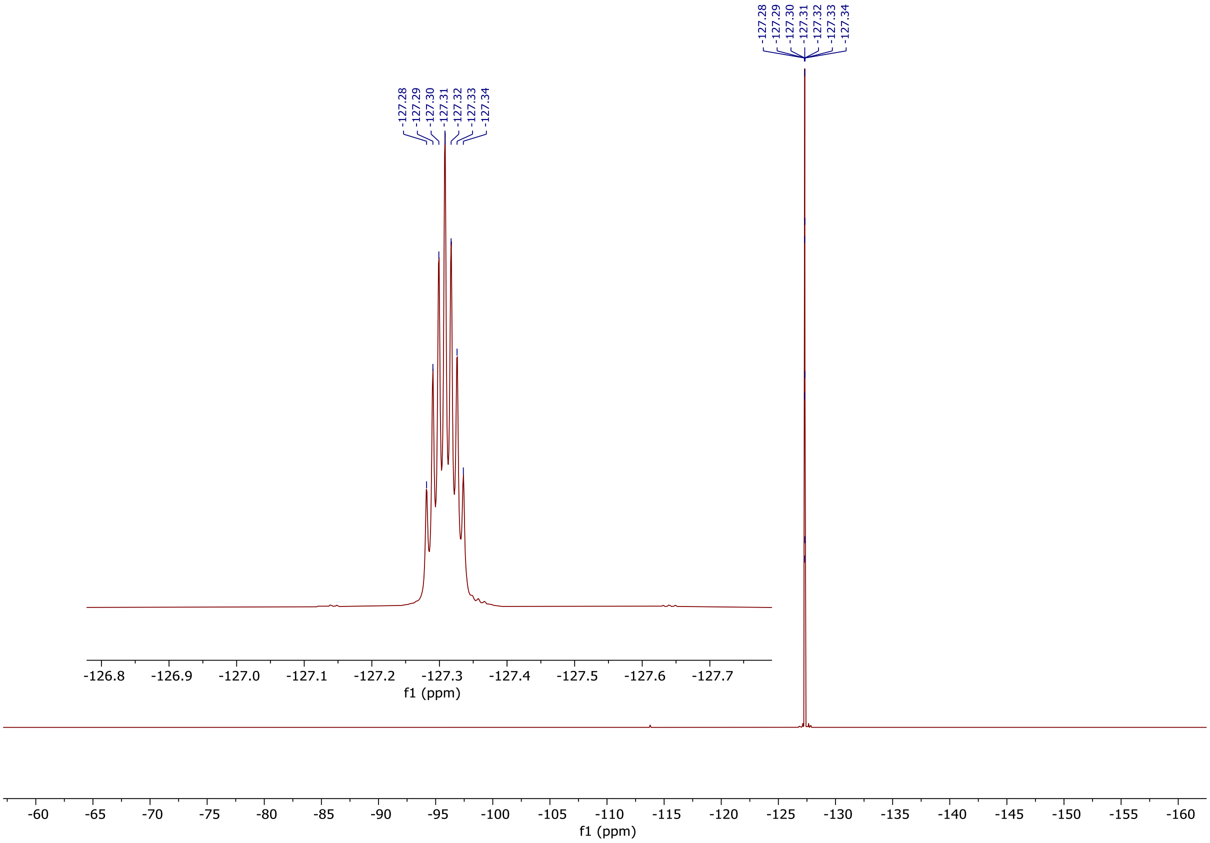

Figure S59: ^{19}F NMR spectrum of compound **3**-NO_2 in $CDCl_3$.

2-chloro-6-{[(4-fluorophenyl)amino]methyl}phenol (3-Cl)

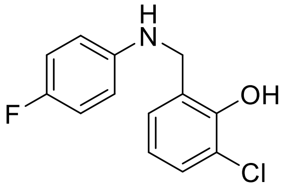

Prepared according to general procedure B using **2-Cl** (3.0 mmol, 747 mg) and $NaBH_4$ (6.0 mmol, 8 eq, 228 mg). After the aqueous workup and evaporation of the volatiles, the crude product was passed through a 10 cm silica plug (eluent 50:1 DCM:MeOH). Recrystallization from DCM/Hexane afforded the title compound as a colourless solid (654 mg, 87% yield). Mp 95 – 97 °C.

^1H NMR (601 MHz, $CDCl_3$) δ 7.94 (br s, 1H), 7.28 (dd, *J* = 8.0, 1.5 Hz, 1H), 7.11 (d, *J* = 7.5 Hz, 1H), 6.95 – 6.88 (m, 2H), 6.82 (t, *J* = 7.8 Hz, 1H), 6.74 – 6.68 (m, 3H), 4.39 (d, *J* = 5.5 Hz, 2H), 4.00 (br s, 1H). ^{13}C NMR (151 MHz, $CDCl_3$) δ 157.38 (d, *J* = 237.9 Hz), 151.43, 143.41 (d, *J* = 2.0 Hz), 128.99, 127.38, 125.23, 121.01, 120.73, 116.31 (d, *J* = 7.6 Hz), 116.02 (d, *J* = 22.5 Hz), 47.62. ^{19}F NMR (471 MHz, $CDCl_3$) δ -124.69 (tt, *J* = 8.5, 4.4 fHz). HRMS (ESI+): m/z calcd. for $C_{13}H_{12}ClFNO$ $[M+H]^+$: 252.0586, found 252.0604.

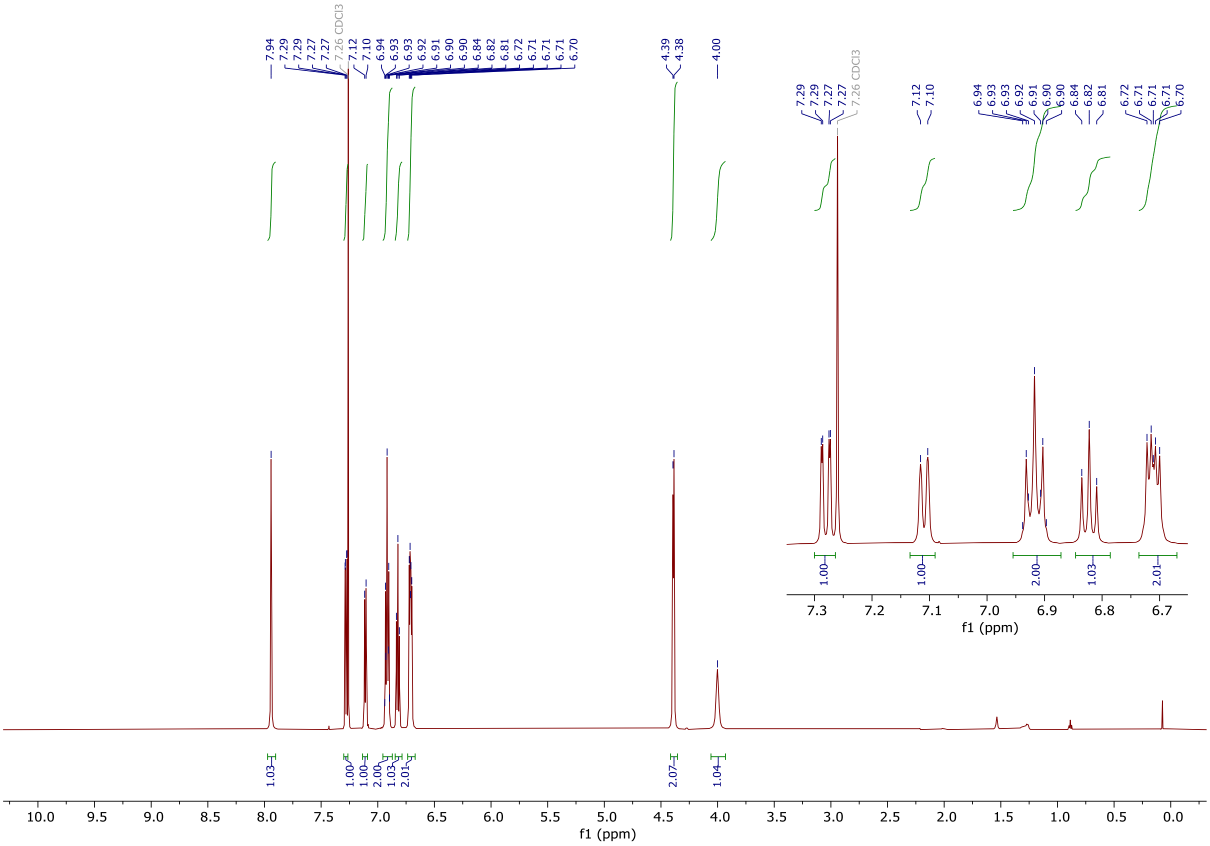

Figure S60: ^{1}H NMR spectrum of compound **3**-Cl in $CDCl_3$.

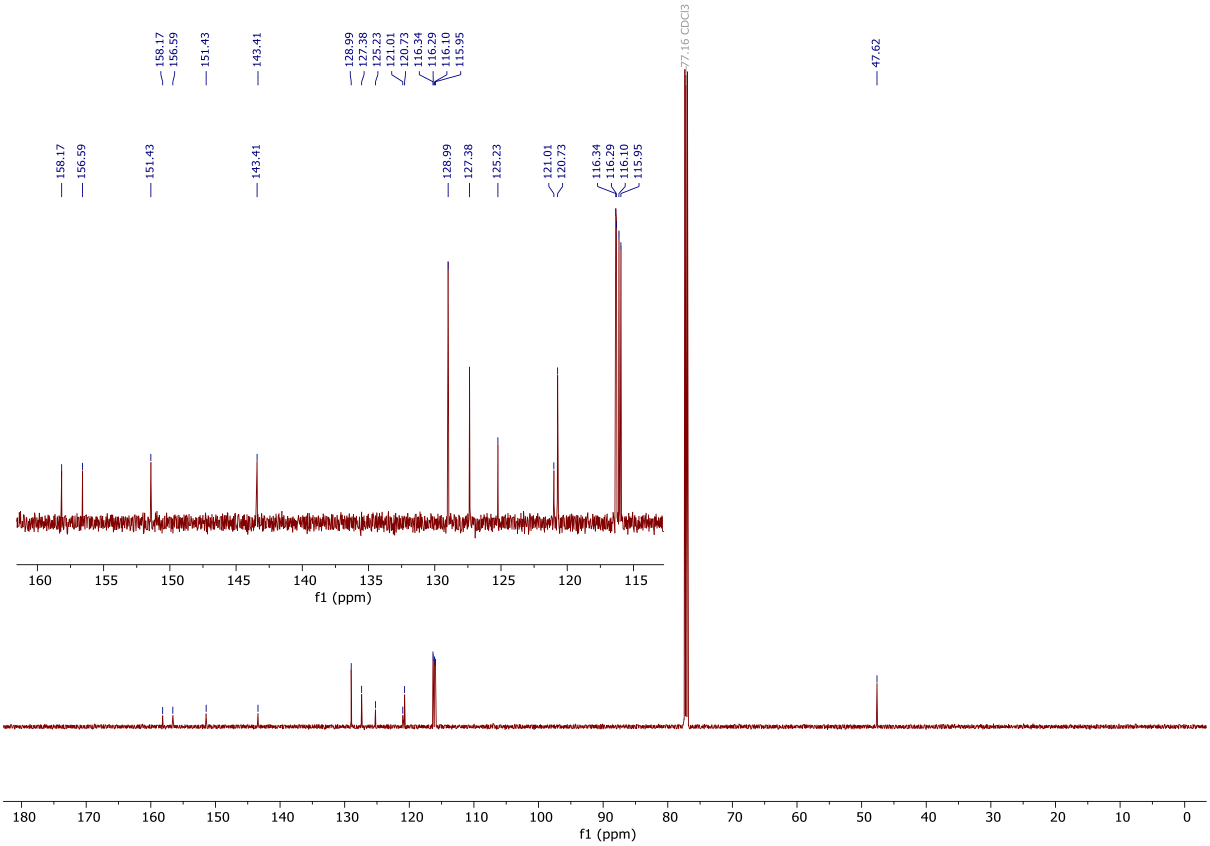

Figure S61: ^{13}C NMR spectrum of compound **3**-Cl in $CDCl_3$.

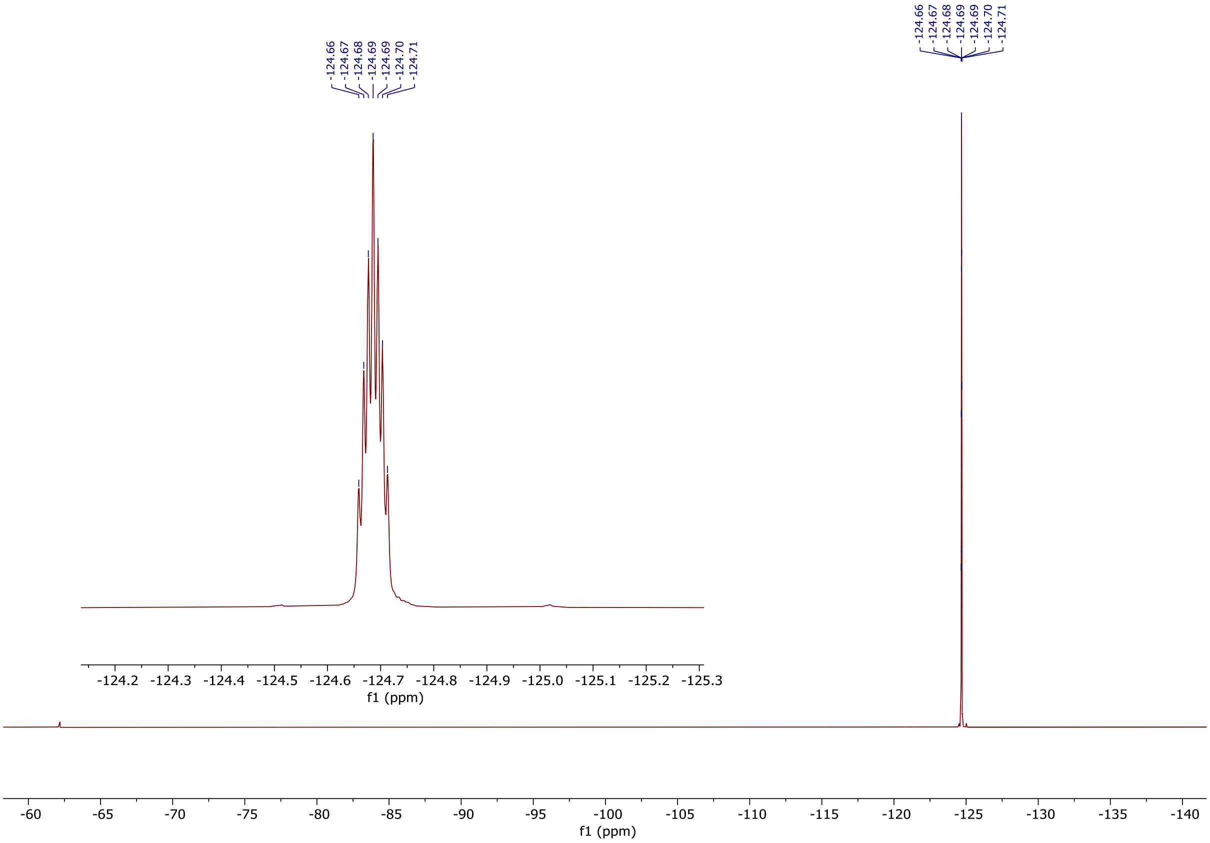

Figure S62: ^{19}F NMR spectrum of compound **3**-Cl in $CDCl_3$.

2-*tert*-butyl-6-{[(4-fluorophenyl)amino]methyl}phenol (3-*t*Bu)

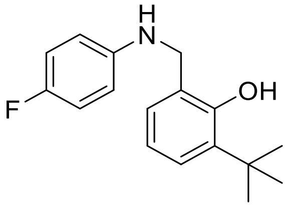

Prepared according to general procedure B using **2-*t*Bu** (3.0 mmol, 813 mg) and $NaBH_4$ (6.0 mmol, 8 eq, 228 mg). After the aqueous workup and evaporation of the volatiles, the crude product was passed through a 10 cm silica plug (eluent 50:1 DCM:MeOH). Recrystallisation from DCM/Hexane afforded the title compound as a colourless solid (744 mg, 91% yield). Mp 108 – 110 °C.

^{1}H NMR (601 MHz, $CDCl_3$) δ 8.90 (br s, 1H), 7.28 (dd, *J* = 7.8, 3.2 Hz, 1H), 7.02 (d, *J* = 7.5 Hz, 1H), 6.99 – 6.95 (m, 2H), 6.86 – 6.80 (m, 3H), 4.36 (s, 2H), 3.82 (br s, 1H), 1.44 (s, 9H). ^{13}C NMR (151 MHz, $CDCl_3$) δ 158.01 (d, *J* = 239.4 Hz), 156.14, 143.28 (d, *J* = 2.2 Hz), 137.46, 126.99, 126.86, 123.00, 119.41, 117.66 (d, *J* = 7.7 Hz), 116.05 (d, *J* = 22.5 Hz), 50.28, 34.92, 29.72. ^{19}F NMR (471 MHz, $CDCl_3$) δ -123.16 (tt, *J* = 12.9, 4.7 Hz). HRMS (ESI+): m/z calcd. for $C_{17}H_{21}FNO$ $[M+H]^+$: 274.1602, found 274.1595.

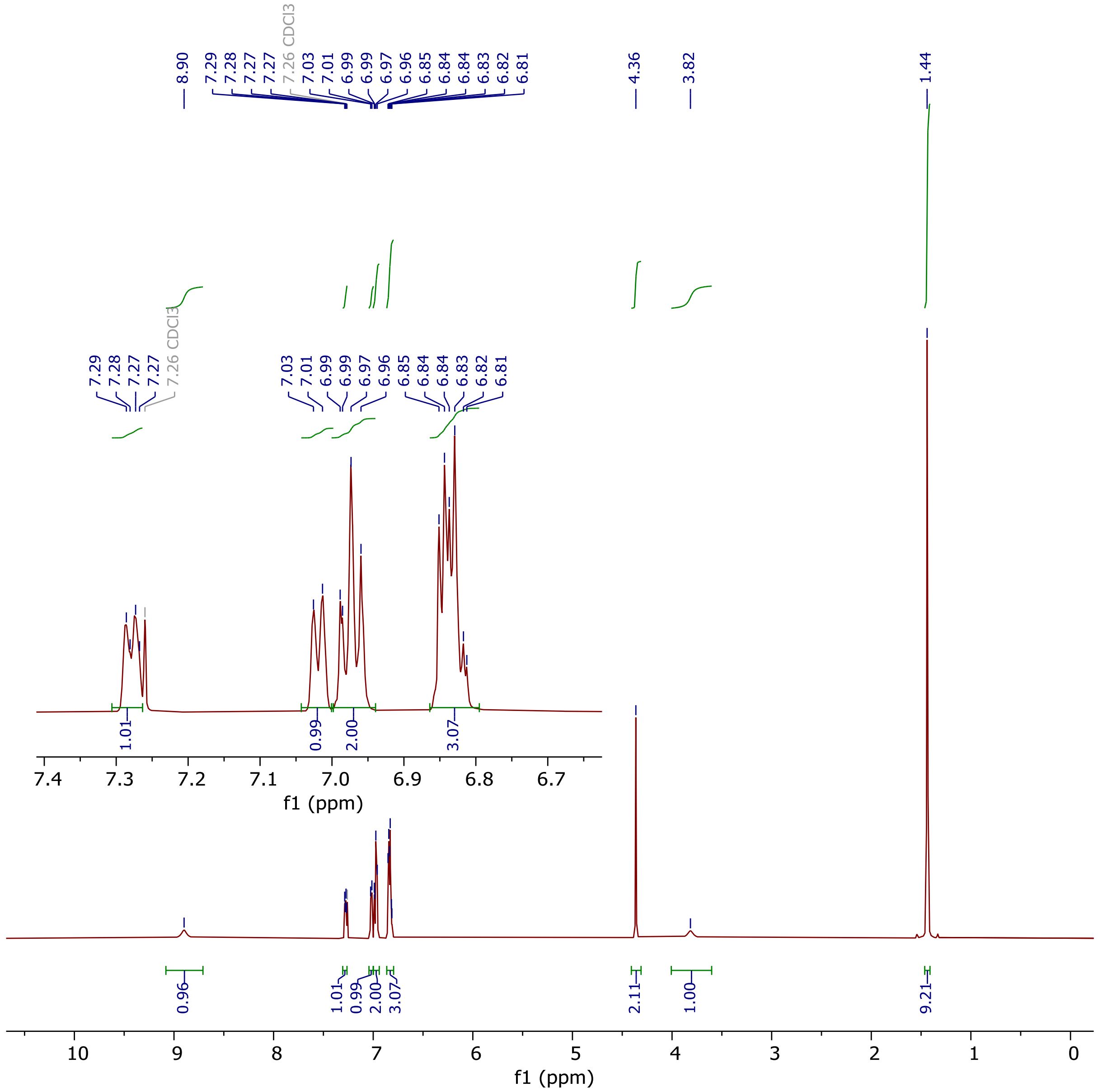

Figure S63: ^{1}H NMR spectrum of compound **3**-^{t}Bu in $CDCl_3$.

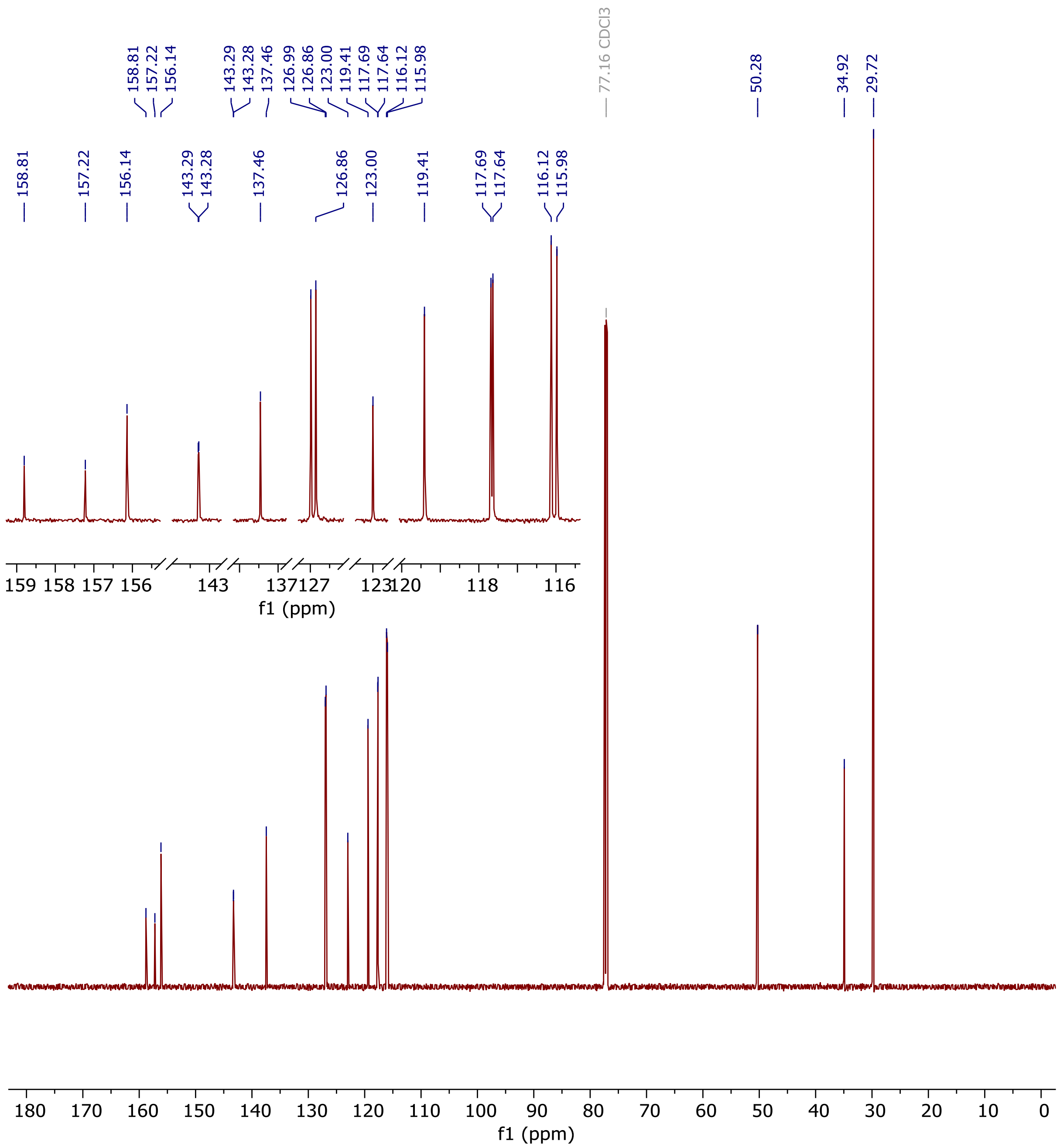

Figure S64: ^{13}C NMR spectrum of compound **3**-^{t}Bu in $CDCl_3$.

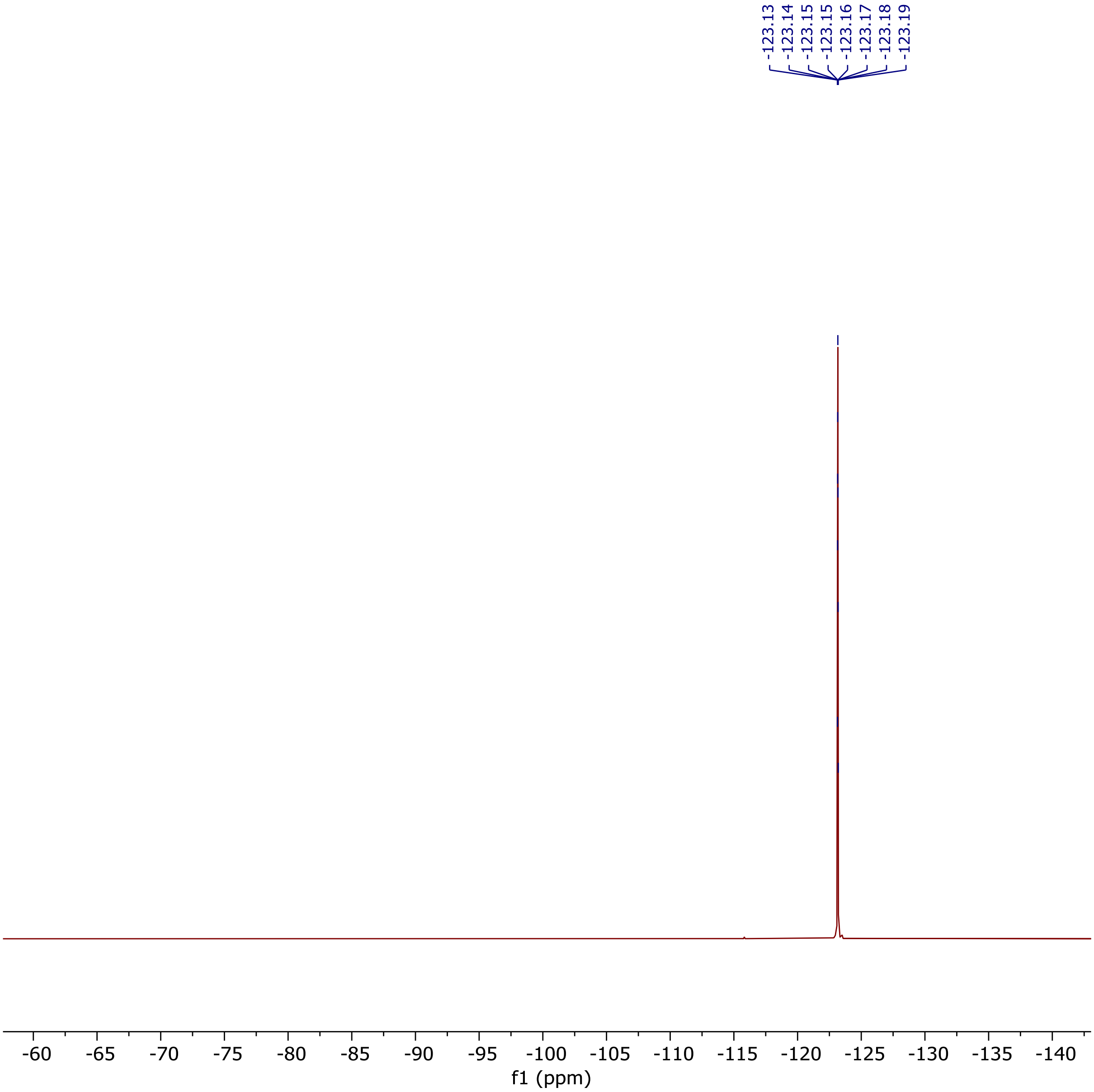

Figure S65: ^{19}F NMR spectrum of compound **3-**^{t}Bu in $CDCl_3$.

2-{[(4-fluorophenyl)amino]methyl}phenol (3-H)

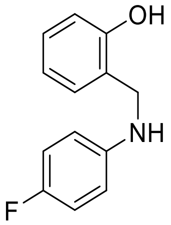

Prepared according to general procedure B using **2-H** (3.0 mmol, 650 mg) and $NaBH_4$ (6.0 mmol, 8 eq, 228 mg). After aqueous workup and evaporation of the volatiles, the crude product was passed through a 10 cm silica plug (eluent 50:1 DCM:MeOH). After evaporation of volatiles, the title compound was obtained as a colourless, which slowly crystallised to give an off-white solid (477 mg, 73% yield). Mp 114 – 116 °C.

^{1}H NMR (601 MHz, $CDCl_3$) δ 7.23 (td, J = 7.8, 1.7 Hz, 1H), 7.15 (dd, J = 7.4, 1.6 Hz, 1H), 6.97 – 6.92 (m, 2H), 6.90 (dd, J = 8.1, 1.3 Hz, 1H), 6.88 (td, J = 7.5, 1.2 Hz, 1H), 6.82 – 6.78 (m, 2H), 4.39 (s, 2H). ^{13}C NMR (151 MHz, $CDCl_3$) δ 157.93 (d, J = 239.2 Hz), 156.98, 143.34 (d, J = 2.3 Hz), 129.45, 128.80, 122.73, 120.22, 117.38 (d, J = 7.7 Hz), 116.84, 116.07 (d, J = 22.4 Hz), 49.67. ^{19}F NMR (471 MHz, $CDCl_3$) δ -123.43 (tt, J = 8.4, 4.5 Hz). HRMS (ESI+): m/z calcd. for $C_{14}H_{13}FNO$ $[M+H]^+$: 230.0976, found 230.0978.

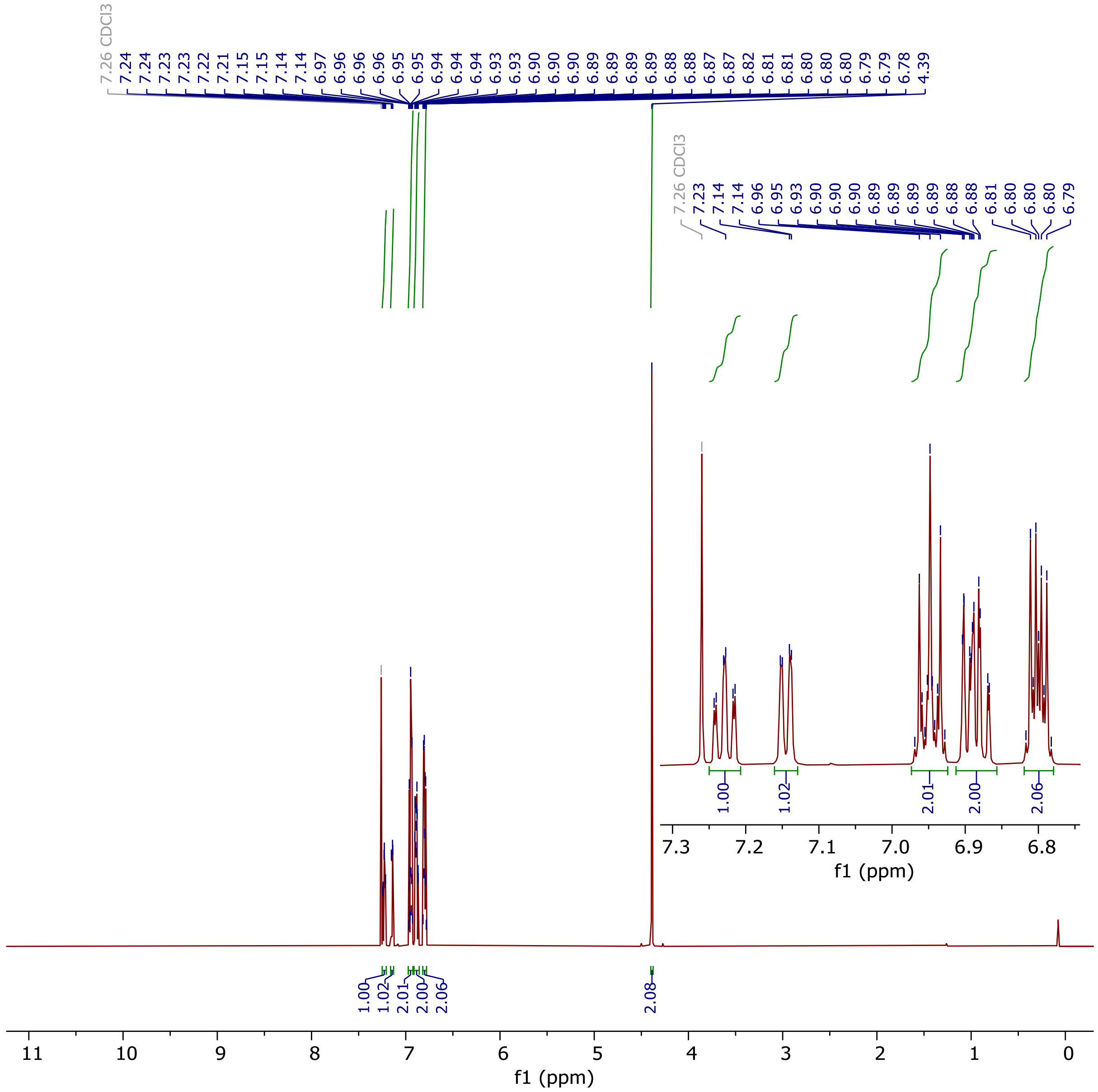

Figure S66: ^{1}H NMR spectrum of compound **3**-H in $CDCl_3$.

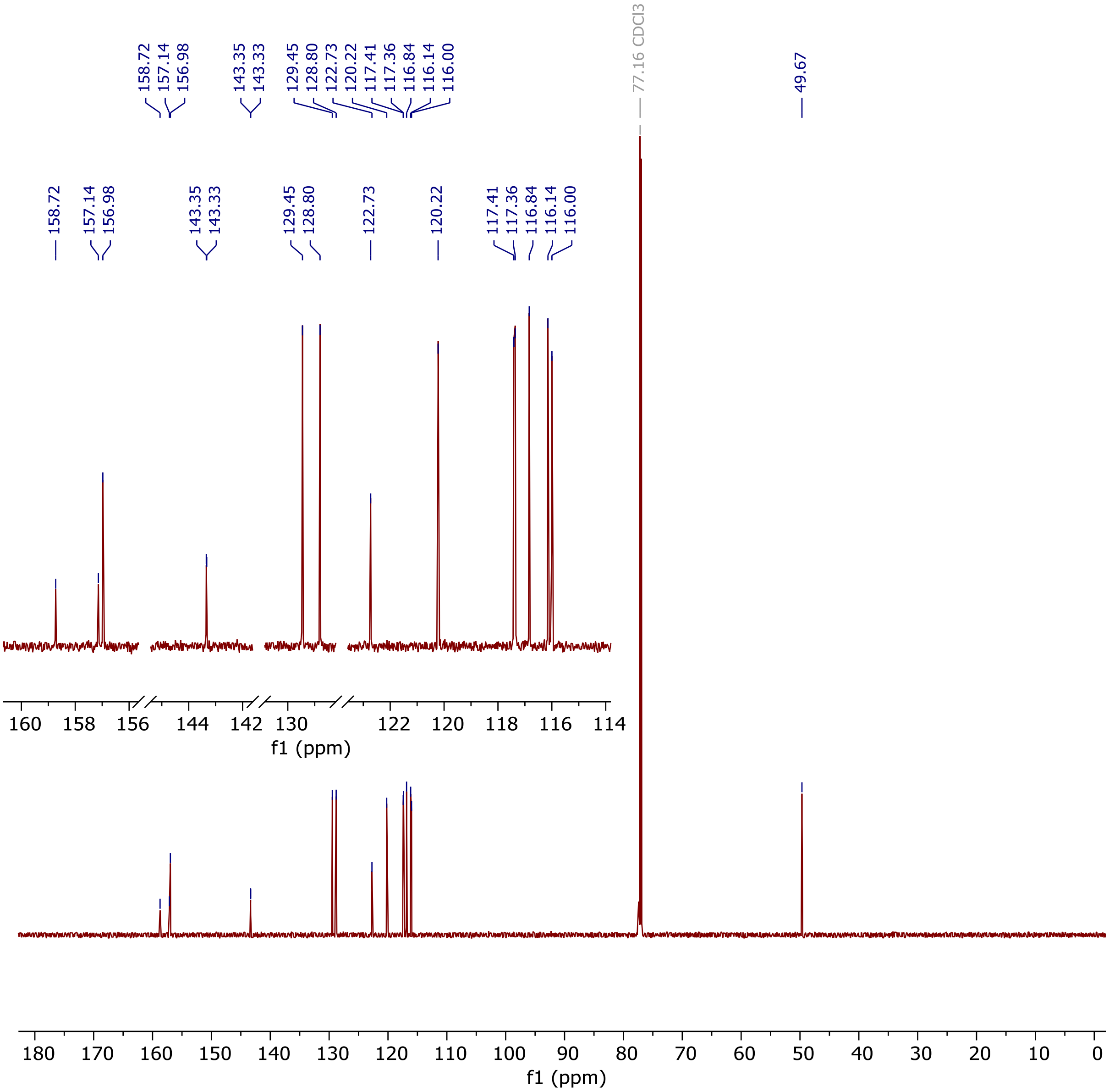

Figure S67: ^{13}C NMR spectrum of compound **3**-H in $CDCl_3$.

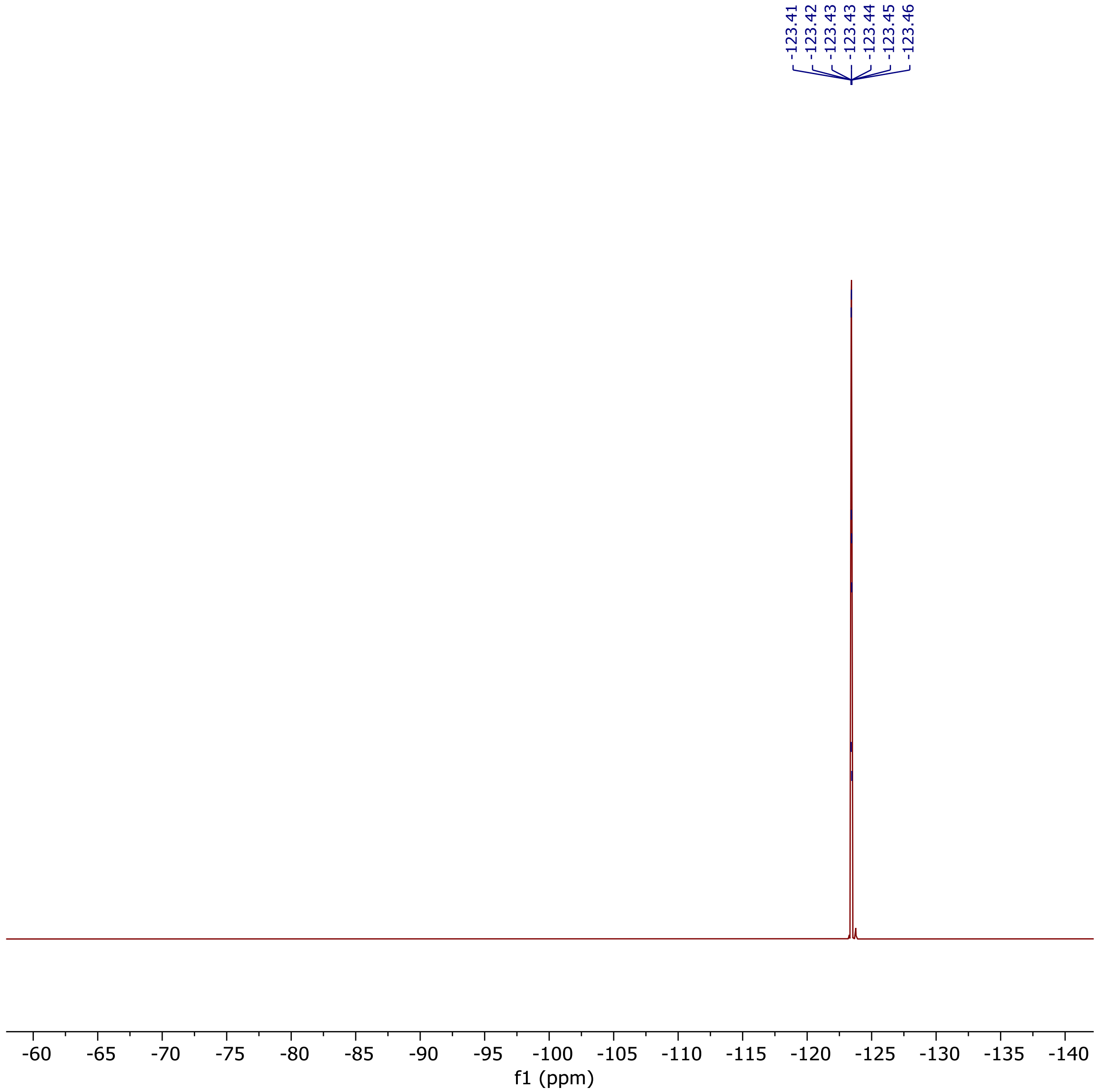

Figure S68: ^{19}F NMR spectrum of compound **3**-H in $CDCl_3$.

2-fluoro-6-{[(4-fluorophenyl)amino]methyl}phenol (3-F)

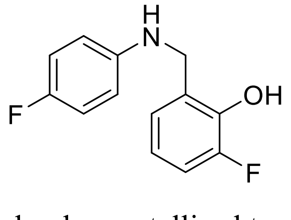

Prepared according to general procedure B using **2-F** (3.0 mmol, 700 mg) and $NaBH_4$ (6.0 mmol, 8 eq, 228 mg). After aqueous workup and evaporation of the volatiles, the crude product was passed through a 10 cm silica plug (eluent 50:1 DCM:MeOH). After evaporation of volatiles, the title compound was obtained as a colourless oil, which slowly crystallised to give a colourless solid (488 mg, 69% yield). Mp 115 – 117 °C.

^{1}H NMR (601 MHz, $CDCl_3$) δ 7.86 (br s, 1H), 7.03 (ddd, *J* = 10.2, 8.2, 1.5 Hz, 1H), 6.96 (d, *J* = 7.7 Hz, 1H), 6.95 – 6.91 (m, 2H), 6.80 (td, *J* = 7.9, 4.9 Hz, 1H), 6.76 – 6.72 (m, 2H), 4.41 (s, 2H), 3.98 (br s, 1H). ^{13}C NMR (151 MHz, $CDCl_3$) δ 157.58 (d, *J* = 238.4 Hz), 151.59 (d, *J* = 241.7 Hz), 144.19 (d, *J* = 13.1 Hz), 143.37 (d, *J* = 2.3 Hz), 125.85, 123.82 (d, *J* = 3.4 Hz), 119.94 (d, *J* = 7.2 Hz), 116.69 (d, *J* = 7.8 Hz), 116.04 (d, *J* = 22.4 Hz), 115.50 (d, *J* = 18.4 Hz), 47.94 (d, *J* = 2.8 Hz). ^{19}F NMR (471 MHz, $CDCl_3$) δ -124.20 (tt, *J* = 8.5, 4.4 Hz), -138.95 (dd,

J = 10.5, 4.9 Hz). HRMS (ESI+): m/z calcd. for $C_{13}H_{12}F_2NO$ [M+H]$^+$: 236.0881, found 236.0872.

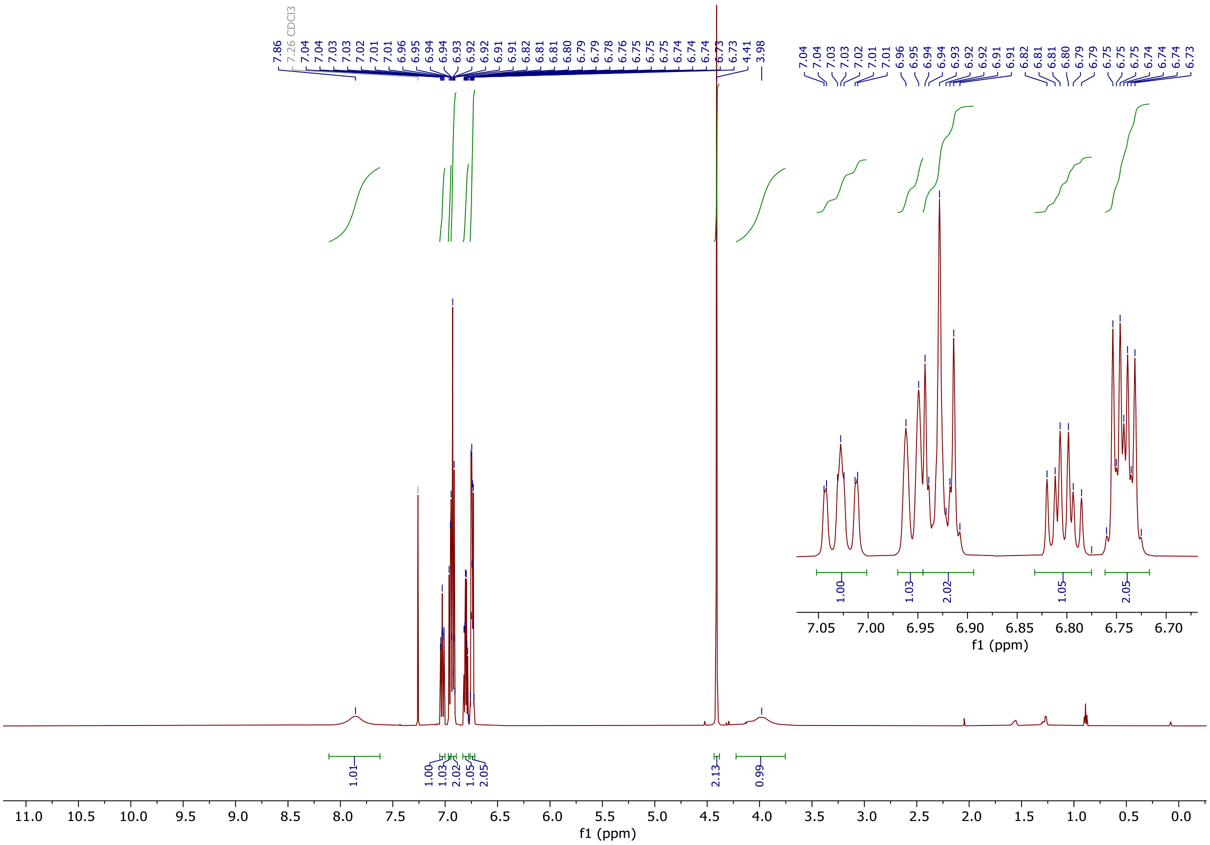

Figure S69: ^{1}H NMR spectrum of compound **3**-F in $CDCl_3$.

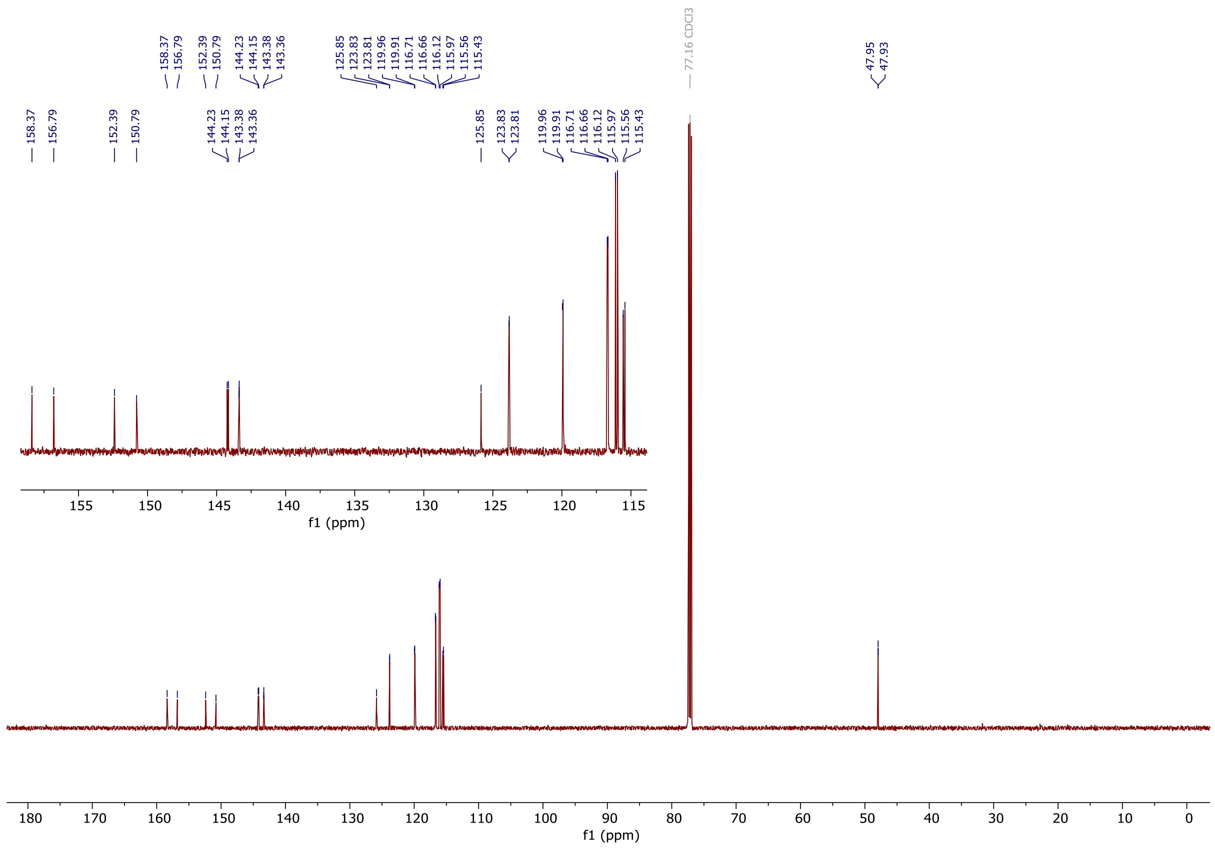

Figure S70: ^{13}C NMR spectrum of compound **3**-F in $CDCl_3$.

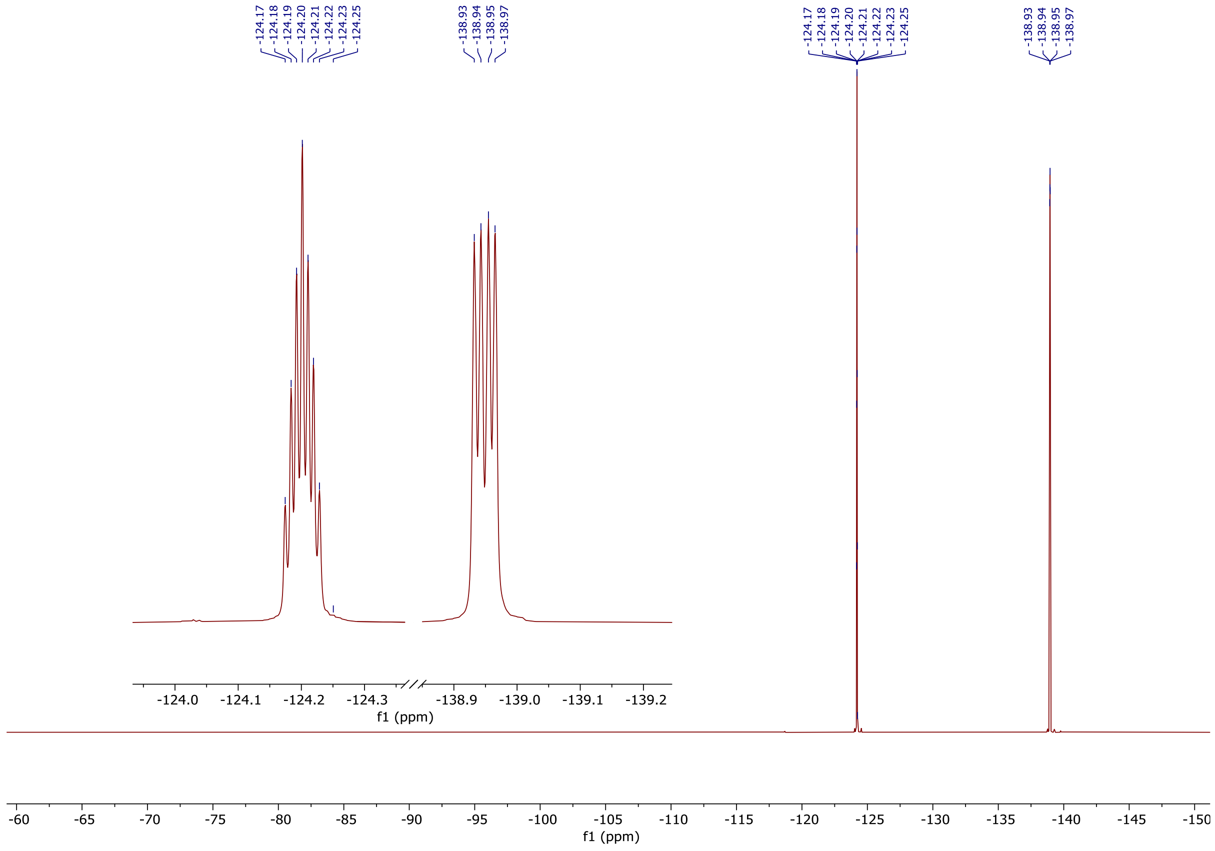

Figure S71: ^{19}F NMR spectrum of compound **3**-F in $CDCl_3$.

2-{[(4-fluorophenyl)amino]methyl}-6-methylphenol (3-Me)

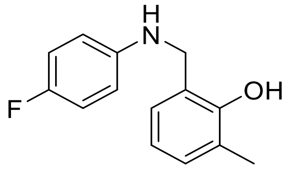

Prepared according to general procedure B using **2**-Me (3.0 mmol, 685 mg) and $NaBH_4$ (6.0 mmol, 8 eq, 228 mg). After aqueous workup and evaporation of the volatiles, the crude product was passed through a 10 cm silica plug (eluent 50:1 DCM:MeOH). Recrystallisation from DCM/Hexane afforded the title compound as a colourless solid (525 mg, 76% yield). Mp 76 – 78 °C.

^{1}H NMR (601 MHz, $CDCl_3$) δ 8.66 (br s, 1H), 7.10 (d, J = 7.5 Hz, 1H), 6.99 (d, J = 6.8 Hz, 1H), 6.97 – 6.92 (m, 2H), 6.83 – 6.79 (m, 2H), 6.79 (t, J = 7.5 Hz, 1H), 4.37 (s, 2H), 3.86 (br s, 1H), 2.25 (s, 3H). ^{13}C NMR (151 MHz, $CDCl_3$) δ 157.94 (d, J = 239.1 Hz), 155.17, 143.38 (d, J = 2.7 Hz), 130.68, 126.39, 125.74, 122.01, 119.68, 117.43 (d, J = 7.6 Hz), 116.06 (d, J = 22.2 Hz), 49.82, 15.83. ^{19}F NMR (471 MHz, $CDCl_3$) δ -123.43 (tt, J = 8.5, 4.5 Hz). HRMS (ESI+): m/z calcd. for $C_{14}H_{15}FNO$ $[M+H]^+$: 232.1132, found 232.1136.

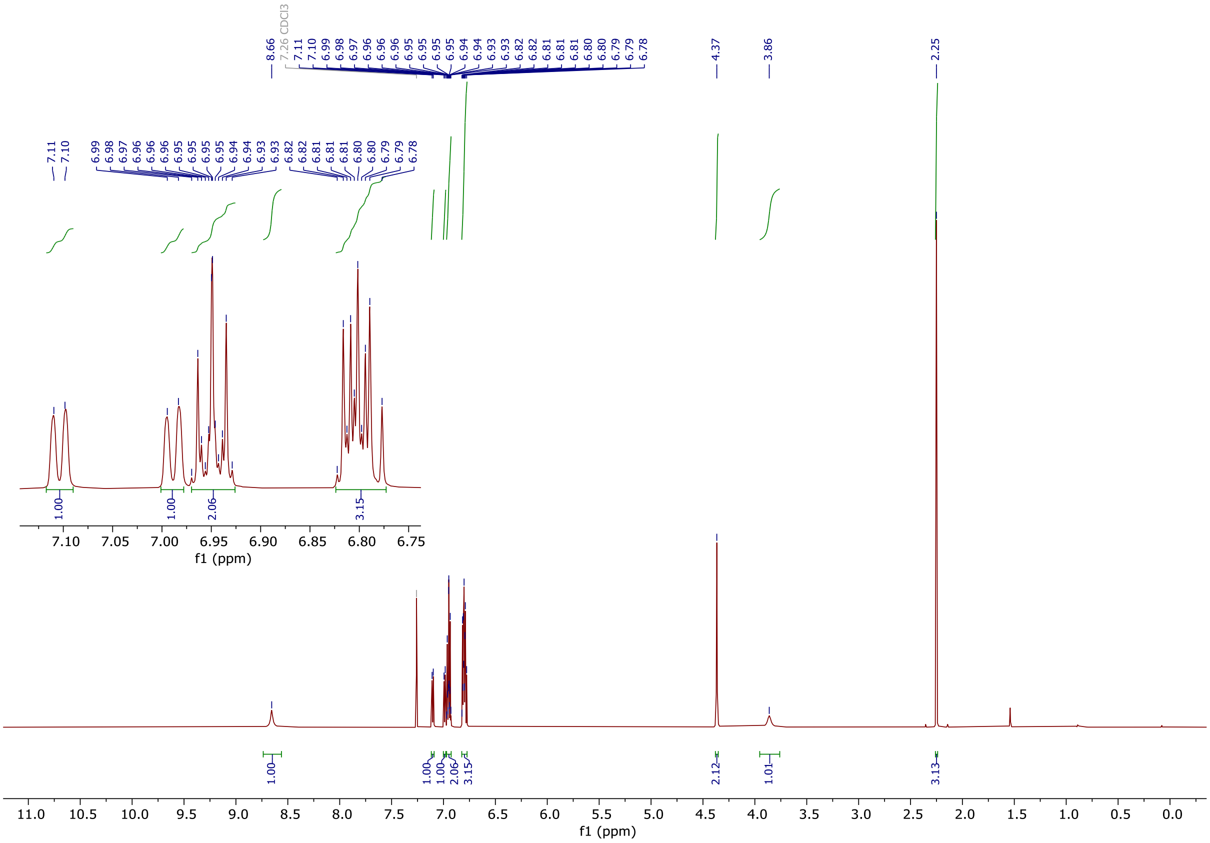

Figure S72: ^{1}H NMR spectrum of compound **3**-Me in $CDCl_3$.

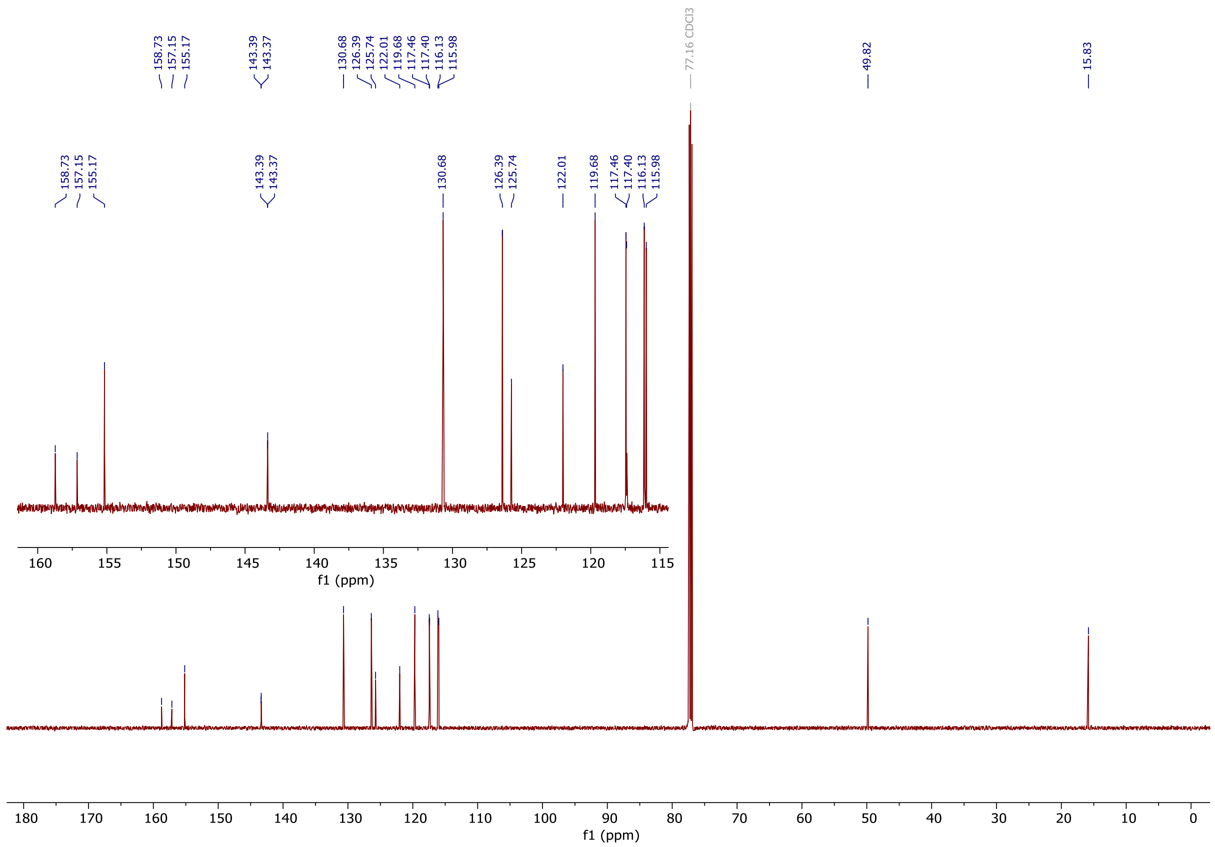

Figure S73: ^{13}C NMR spectrum of compound **3**-Me in $CDCl_3$.

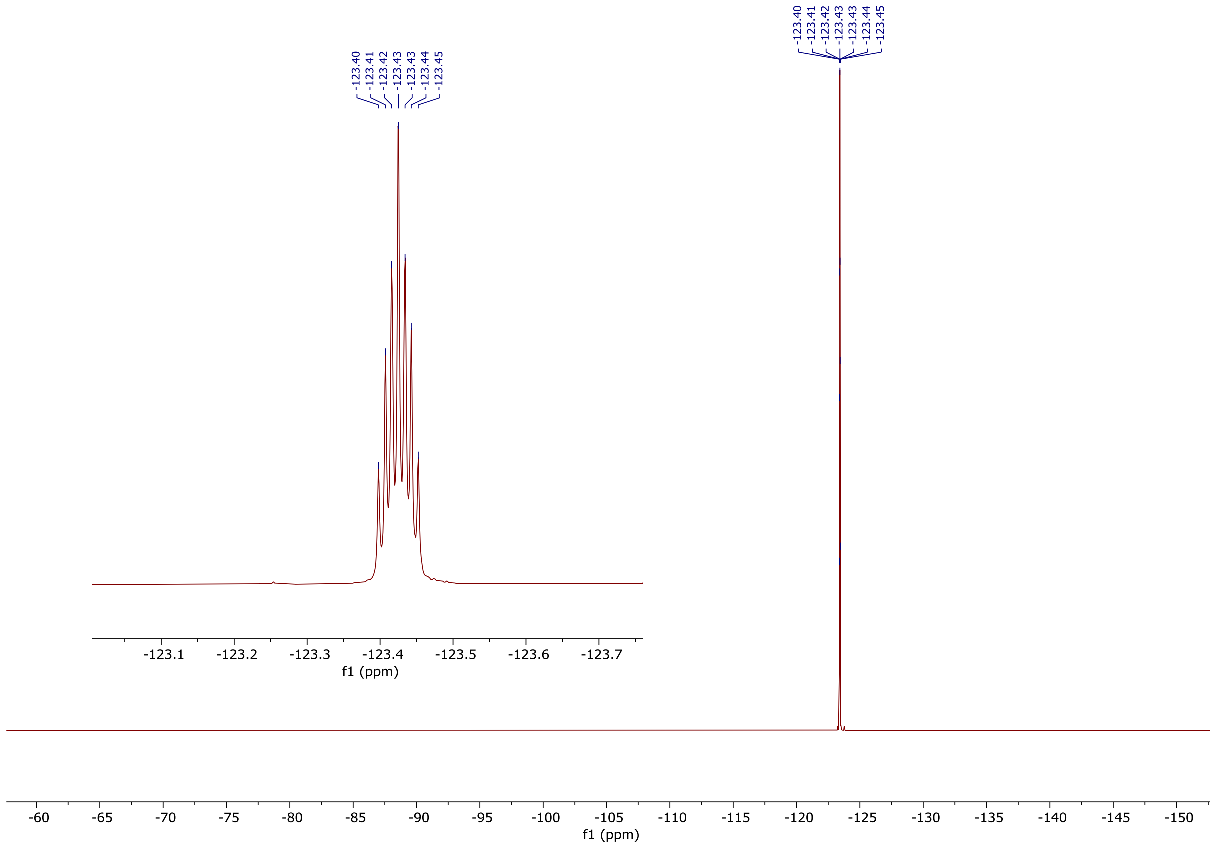

Figure S74: ^{19}F NMR spectrum of compound **3**-Me in $CDCl_3$.

General procedure C: Synthesis of formamide molecular balances **1**-R.

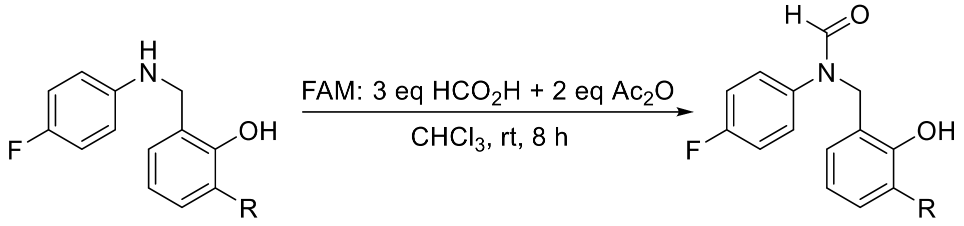

Formic acid-acetic anhydride mixture (FAM) was prepared by mixing neat formic acid and acetic anhydride in 3:2 molar ratio at room temperature. After the initial exothermic reaction was ceased, the FAM mixture was added in one portion to a cold (0 °C, cooling with ice) stirring mixture of the appropriate amine **3**-R in $CHCl_3$. The resulting mixture was left to warm up and stir overnight. Upon consumption of the starting material (as monitored by TLC), the reaction mixture was diluted with 20 mL $CHCl_3$ and washed successively with $NaHCO_3$ (10 mL, sat. aq.), water (2 x 10 mL) and brine (10 mL). After removal of the volatiles, the target formamides were purified *via* recrystallisation or column chromatography.

_N_-(4-fluorophenyl)-_N_-[(2-hydroxy-3methoxyphenyl)methyl]formamide (1-OMe)

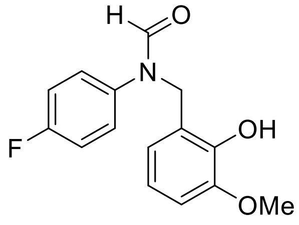

Following general procedure C, **3**-OMe (741 mg, 3 mmol) was dissolved in 2 mL DCM and cooled to 0 °C. FAM (3 eq, made from 5.4 mmol formic acid, 204 µL and 3.6 eq acetic anhydride, 340 µL) was added in one portion and reaction was stirred at RT for 8 h. After removal of volatiles, the residue was recrystallised from DCM/Hex to give the title compound as a colourless solid (741 mg, 90%). Mp 141 – 143 °C. HRMS (ESI+): m/z calcd. for $C_{15}H_{15}FNO_3$ $[M+H]^+$: 276.1031, 276.1034.

closed (major) **open** (minor)

^{1}H assignments

^{13}C assignments

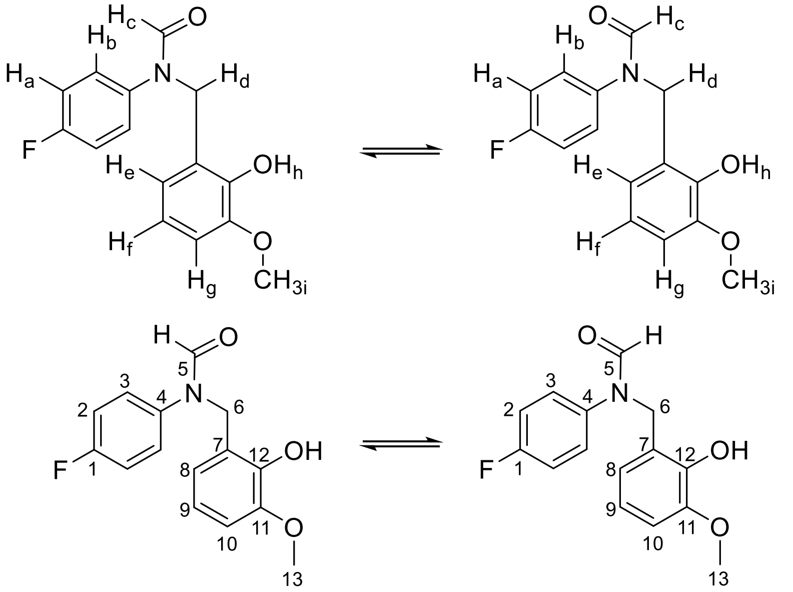

^{1}H NMR (500 MHz, DMSO) δ 8.91 (s, 1H, **H**$_{\mathbf{h}\,\mathrm{open}}$), 8.76 (s, 1H, **H**$_{\mathbf{h}\,\mathrm{closed}}$), 8.57 (s, 1H, **H**$_{\mathbf{c}\,\mathrm{closed}}$), 8.52 (s, 1H, **H**$_{\mathbf{c}\,\mathrm{open}}$), 7.34 (dd, *J* = 8.8, 4.8 Hz, 2H, **H**$_{\mathbf{b}\,\mathrm{closed}}$), 7.28 (t, *J* = 6.9 Hz, 2H, **H**$_{\mathbf{b}\,\mathrm{open}}$), 7.20 (dd, *J* = 8.8, 2.3 Hz, 2H, **H**$_{\mathbf{a}\,\mathrm{closed}}$), 7.13 (t, *J* = 8.6 Hz, 2H, **H**$_{\mathbf{a}\,\mathrm{open}}$), 6.84 (d, *J* = 7.5 Hz, 1H, **H**$_{\mathbf{g}\,\mathrm{open}}$), 6.81 (d, *J* = 8.2 Hz, 1H, **H**$_{\mathbf{g}\,\mathrm{closed}}$), 6.70 – 6.64 (m, 1H, **H**$_{\mathbf{f}\,\mathrm{open}}$, **H**$_{\mathbf{f}\,\mathrm{closed}}$), 6.62 (d, J

= 8.3 Hz, 1H, **H**$_{\mathbf{e}\ \mathrm{closed}}$), 6.57 (d, J = 7.8 Hz, 1H, **H**$_{\mathbf{e}\ \mathrm{open}}$), 4.92 (s, 2H, **H**$_{\mathbf{d}\ \mathrm{closed}}$), 4.84 (s, 2H, **H**$_{\mathbf{d}\ \mathrm{open}}$), 3.75 (s, 3H, **H**$_{\mathbf{i}\ \mathrm{open}}$, **H**$_{\mathbf{i}\ \mathrm{closed}}$).

Closed conformer (major):

^{13}C NMR (126 MHz, DMSO) δ 162.40 (**C**$_{\mathbf{5}}$), 160.05 (d, *J* = 242.8 Hz, **C**$_{\mathbf{1}}$), 147.35, (**C**$_{\mathbf{11}}$), 143.86 (**C**$_{\mathbf{12}}$), 137.38 (d, *J* = 2.7 Hz, **C**$_{\mathbf{4}}$), 124.99 (d, *J* = 8.5 Hz, **C**$_{\mathbf{3}}$), 122.93 (**C**$_{\mathbf{7}}$), 119.83 (**C**$_{\mathbf{8}}$), 118.69 (**C**$_{\mathbf{9}}$), 115.97 (d, *J* = 22.6 Hz, **C**$_{\mathbf{2}}$), 110.60 (**C**$_{\mathbf{10}}$), 55.73 (**C**$_{\mathbf{13}}$), 42.27 (**C**$_{\mathbf{6}}$). ^{19}F NMR (471 MHz, DMSO) δ -116.69 (ddd, *J* = 13.7, 8.8, 5.0 Hz).

Open conformer (minor):

^{13}C NMR (126 MHz, DMSO) δ 163.25 (**C**$_{\mathbf{5}}$), 159.73 (d, *J* = 243.0 Hz, **C**$_{\mathbf{1}}$), 147.37 (**C**$_{\mathbf{11}}$), 144.47 (**C**$_{\mathbf{12}}$), 135.20 (d, *J* = 3.0 Hz, **C**$_{\mathbf{4}}$), 127.36 (d, *J* = 8.5 Hz, **C**$_{\mathbf{3}}$), 123.10 (**C**$_{\mathbf{7}}$), 121.16 (**C**$_{\mathbf{8}}$), 118.58 (**C**$_{\mathbf{9}}$), 115.30 (d, *J* = 22.4 Hz, **C**$_{\mathbf{2}}$), 111.23 (**C**$_{\mathbf{10}}$), 55.73 (**C**$_{\mathbf{13}}$), 48.23 (**C**$_{\mathbf{6}}$). ^{19}F NMR (471 MHz, DMSO) δ -116.17 (ddd, *J* = 13.7, 8.8, 5.0 Hz).

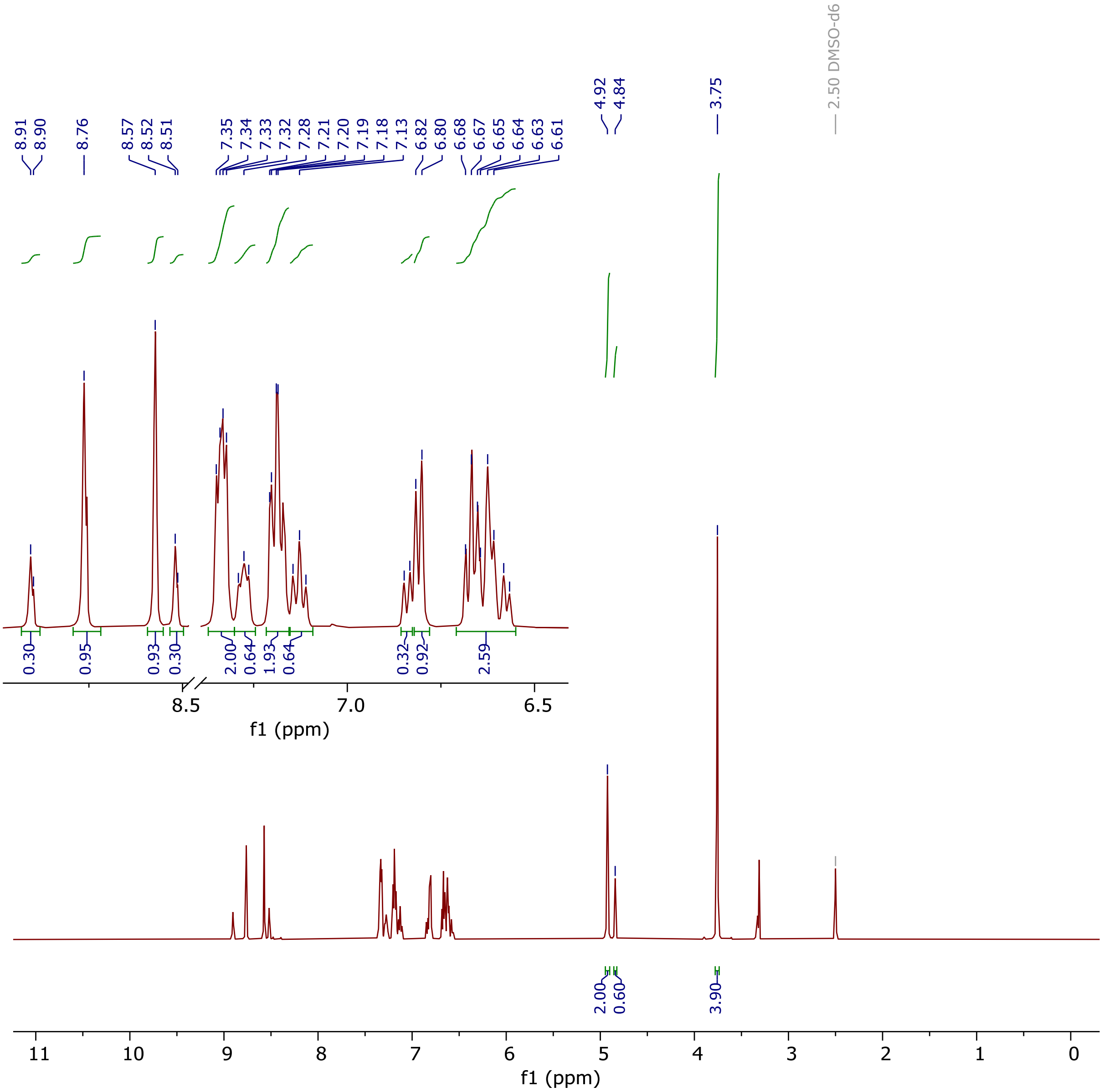

Figure S75: ^{1}H NMR spectrum (DMSO-d_6) of compound **1**-OMe.

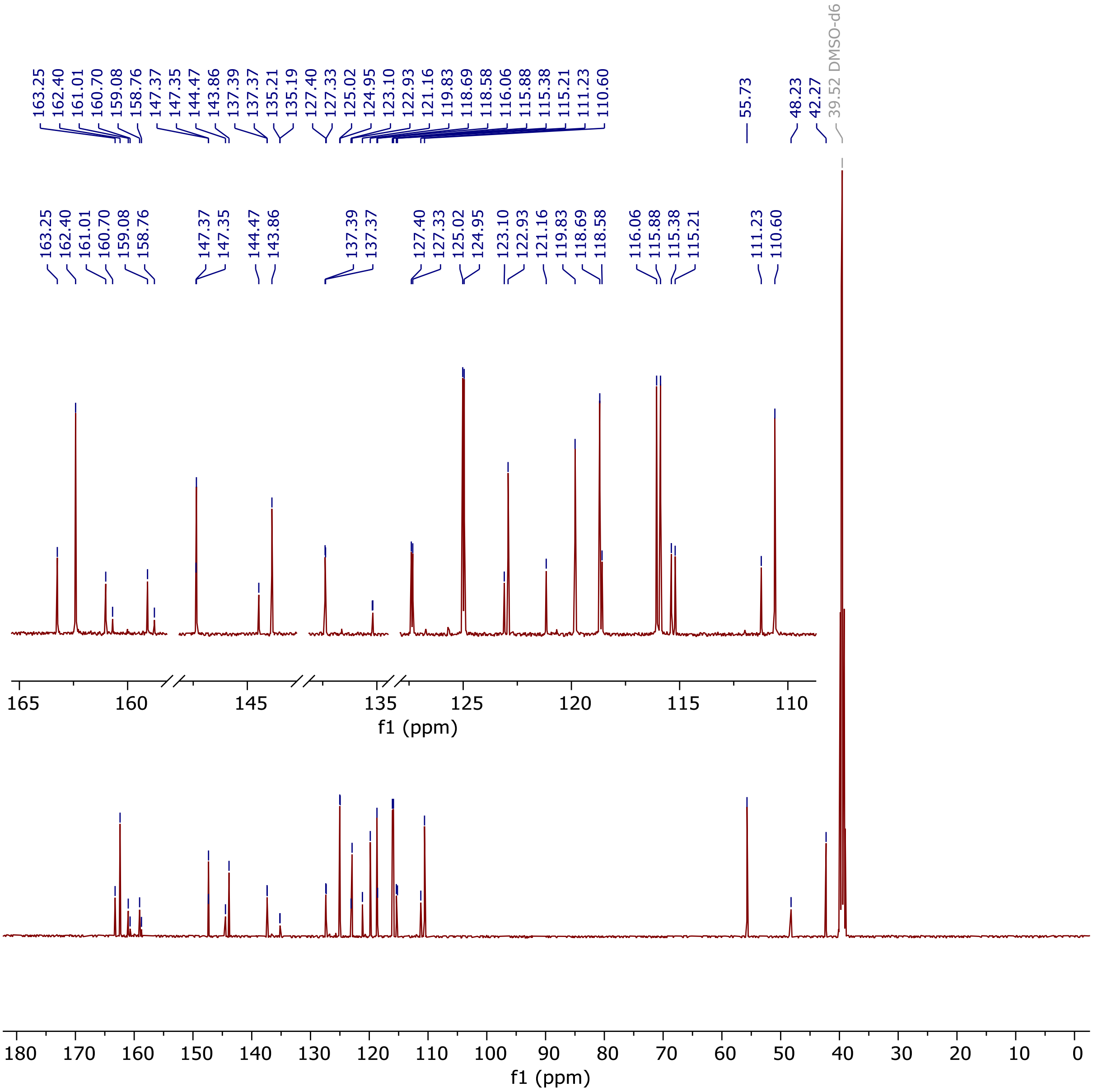

Figure S76: ^{13}C NMR spectrum (DMSO-d_6) of compound **1**-OMe.

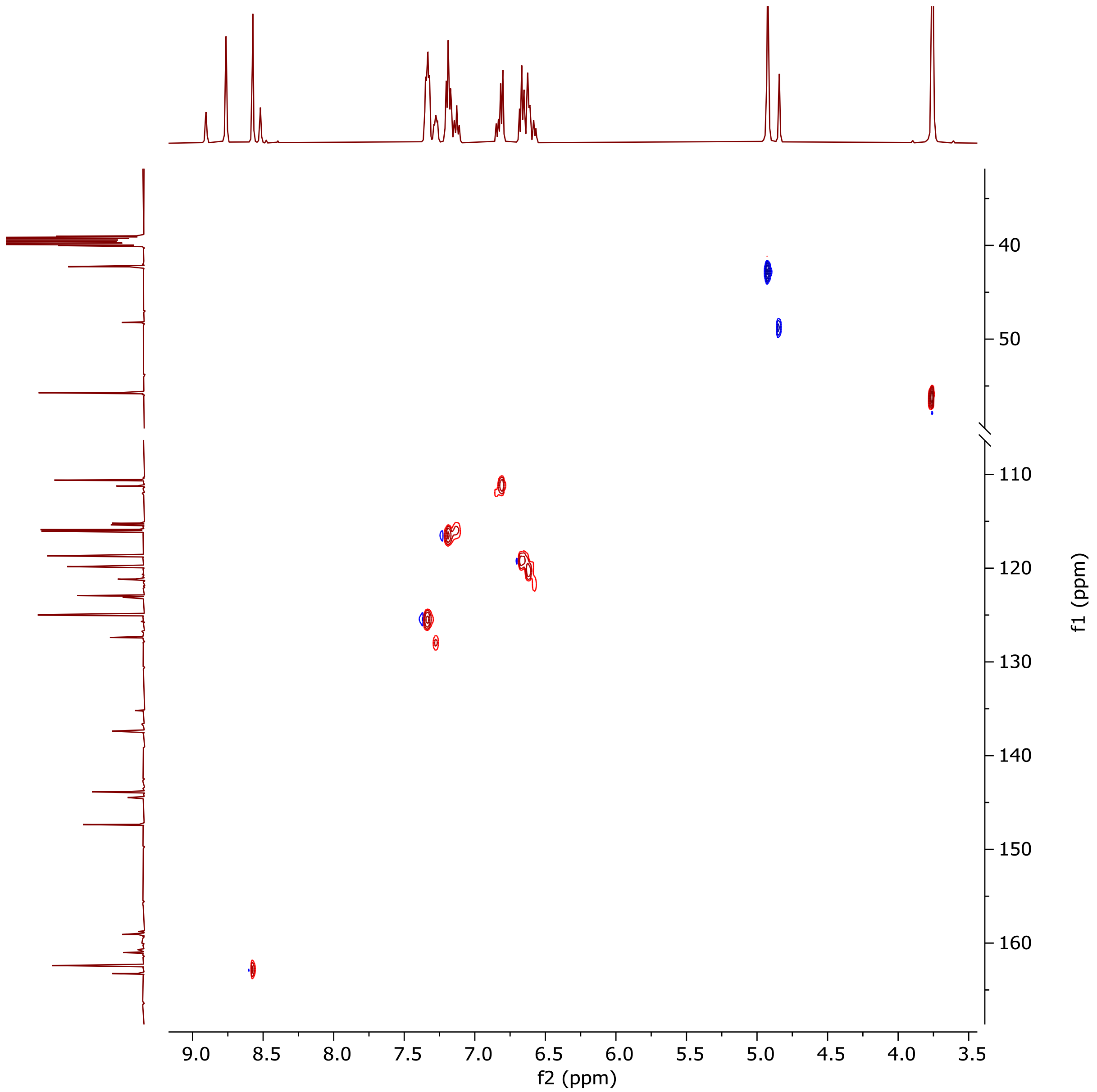

Figure S77: HSQC NMR spectrum (DMSO-d_6) of compound **1**-OMe.

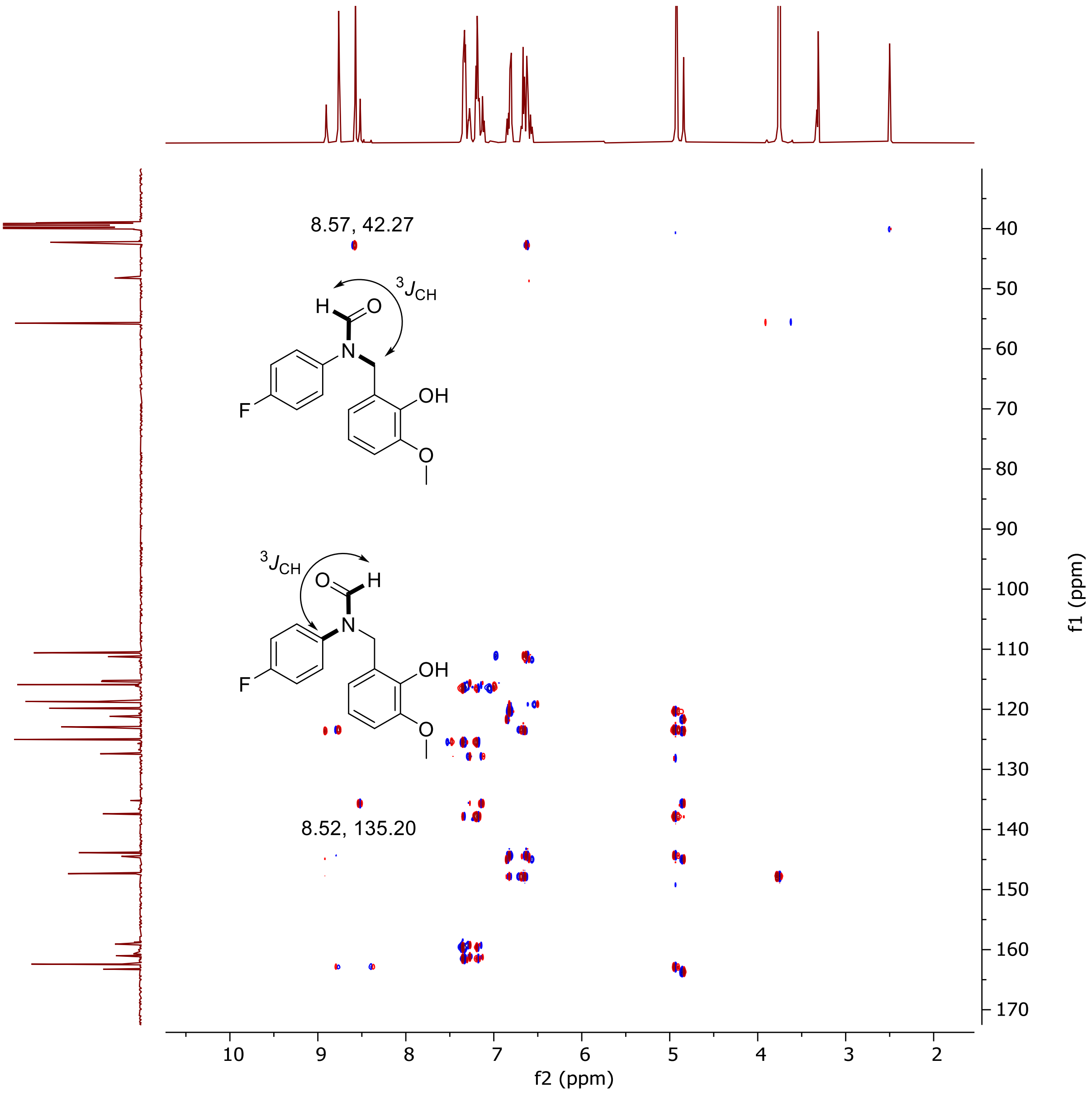

Figure S78: HMBC NMR spectrum (DMSO-d_6) of compound **1**-OMe.

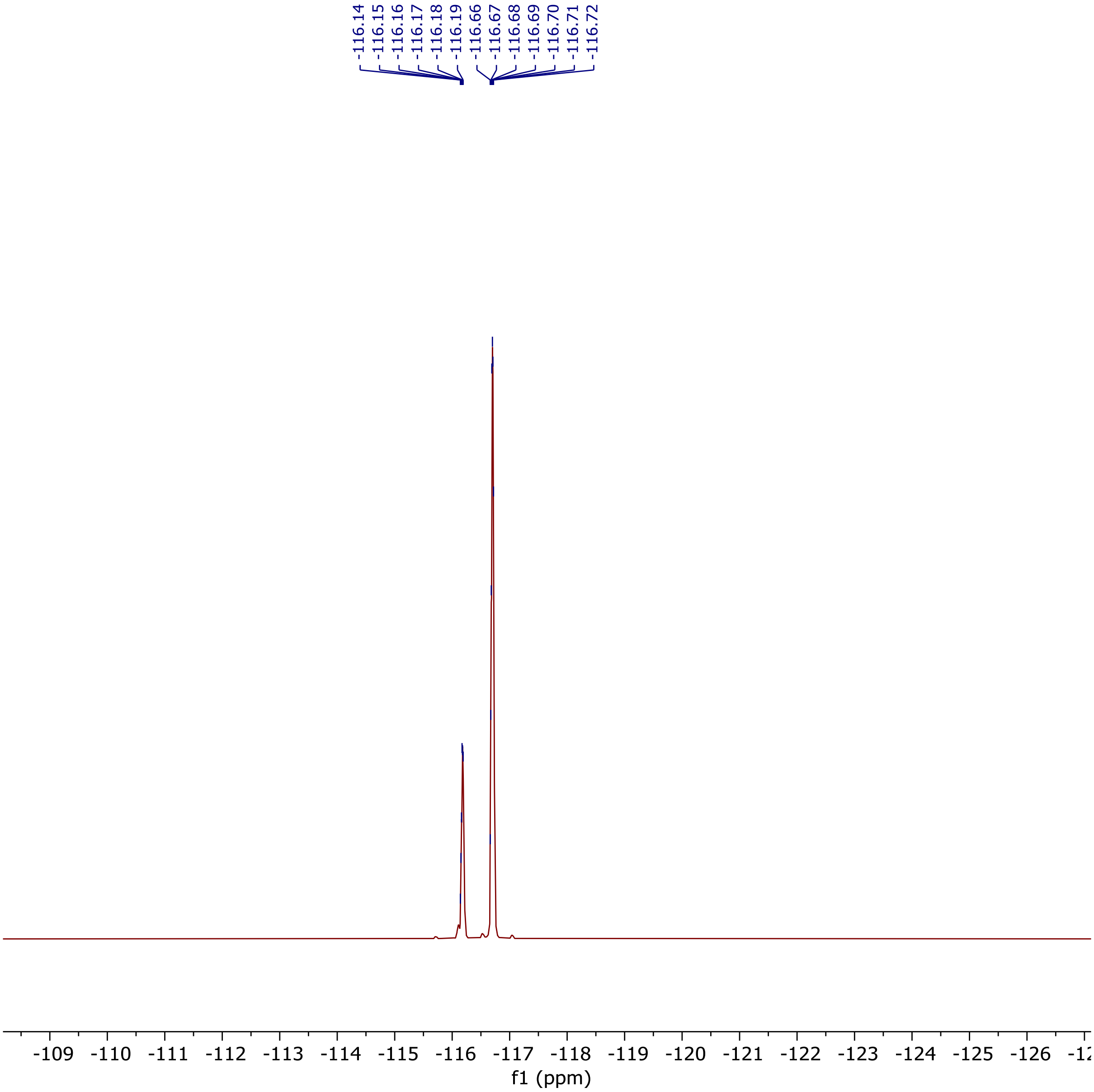

Figure S79: ^{19}F NMR spectrum (DMSO-d_6) of compound **1**-OMe.

***N*-[(3-cyano-2-hydroxyphenyl)methyl]-*N*-(4-fluorophenyl)formamide (1-CN)**

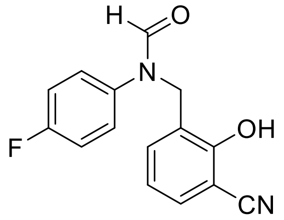

Following general procedure **C**, compound **3**-CN (242 mg, 1.2 mmol) was dissolved in 2 mL DCM and cooled to 0 °C. FAM (3 eq, made from 5.4 mmol formic acid, 204 µL and 3.6 eq acetic anhydride, 340 µL) was added in one portion and reaction was stirred at RT for 8 h. After removal of volatiles, the residue was purified via flash chromatography (eluting with gradient of DCM:MeOH 100:1 to DCM:MeOH 50:1) to afford the title compound as a colourless solid (213 mg, 79%). Mp 109 – 111 °C. HRMS (ESI+): m/z calcd. for $C_{15}H_{11}FN_2O_2Na$ $[M+Na]^+$: 293.0697, found 293.0686.

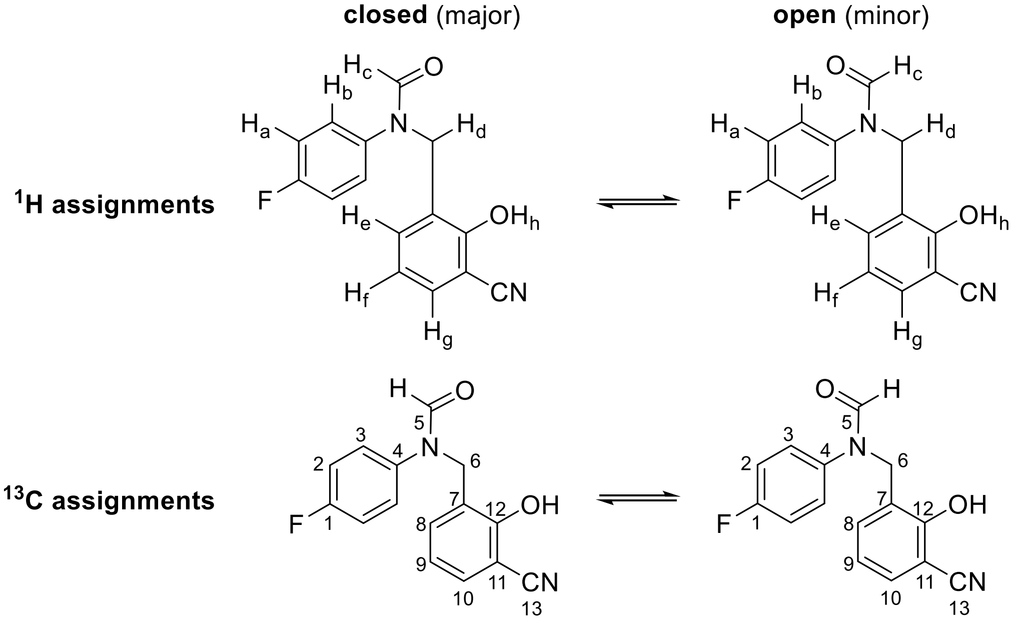

^{1}H NMR (500 MHz, DMSO) δ 10.56 (s, 1H, $\mathbf{H}_{\mathbf{h}\,\mathrm{closed}}$), 8.56 (s, 1H, $\mathbf{H}_{\mathbf{c}\,\mathrm{closed}}$), 8.53 (s, 1H, $\mathbf{H}_{\mathbf{c}\,\mathrm{open}}$), 7.52 (dd, *J* = 7.7, 1.5 Hz, 1H, $\mathbf{H}_{\mathbf{g}\,\mathrm{closed}}$), 7.52 (dd, *J* = 7.7, 1.5 Hz, 1H, $\mathbf{H}_{\mathbf{g}\,\mathrm{open}}$), 7.38 (ddt, *J* = 8.4, 5.9, 2.9 Hz, 2H, $\mathbf{H}_{\mathbf{b}\,\mathrm{closed}}$), 7.30 (dd, *J* = 7.7, 1.5 Hz, 1H, $\mathbf{H}_{\mathbf{e}\,\mathrm{closed}}$), δ 7.27 – 7.21 (m, 2H, overlap, $\mathbf{H}_{\mathbf{a}\,\mathrm{closed}}$), 7.18 – 7.12 (m, 2H, $\mathbf{H}_{\mathbf{a}\,\mathrm{open}}$), 6.92 (t, *J* = 7.7 Hz, 1H, $\mathbf{H}_{\mathbf{f}\,\mathrm{closed}}$), 6.95 – 6.87 (m, 1H, $\mathbf{H}_{\mathbf{f}\,\mathbf{open}}$), 4.96 (s, 2H, $\mathbf{H}_{\mathbf{d}\,\mathrm{closed}}$), 4.94 (s, 2H, $\mathbf{H}_{\mathbf{d}\,\mathrm{open}}$).

Closed conformer (major):

^{13}C NMR (126 MHz, DMSO) δ 163.14 ($\mathbf{C_5}$), 160.33 (d, *J* = 243.2 Hz, $\mathbf{C_1}$), 157.06 ($\mathbf{C_{12}}$), 136.84 (d, *J* = 2.5 Hz, $\mathbf{C_4}$), 133.49 ($\mathbf{C_8}$), 132.38 ($\mathbf{C_{10}}$), 125.50 ($\mathbf{C_7}$), 125.43 (d, *J* = 8.6 Hz, $\mathbf{C_3}$), 120.45 ($\mathbf{C_9}$), 116.88 ($\mathbf{C_{13}}$), 116.21 (d, *J* = 22.7 Hz, $\mathbf{C_2}$), 101.08 ($\mathbf{C_{11}}$), 43.06 ($\mathbf{C_6}$). ^{19}F NMR (471 MHz, DMSO) δ -115.99 (tt, *J* = 8.9, 4.8 Hz).

Open conformer (minor):

^{13}C NMR (126 MHz, DMSO) δ 163.24 ($\mathbf{C_5}$), 159.87 (d, *J* = 243.3 Hz, $\mathbf{C_1}$), 157.33 ($\mathbf{C_{12}}$), 134.71 (d, *J* = 2.4 Hz, $\mathbf{C_4}$), 134.54 ($\mathbf{C_8}$), 132.83 ($\mathbf{C_{10}}$), 127.53 (d, *J* = 8.5 Hz, $\mathbf{C_3}$), 126.31($\mathbf{C_7}$), 116.86 ($\mathbf{C_{13}}$), 115.49 (d, *J* = 22.4 Hz, $\mathbf{C_2}$), 101.25 ($\mathbf{C_{11}}$), 47.77 ($\mathbf{C_6}$). ^{19}F NMR (471 MHz, DMSO) δ -115.73 (tt, *J* = 8.6, 5.1 Hz).

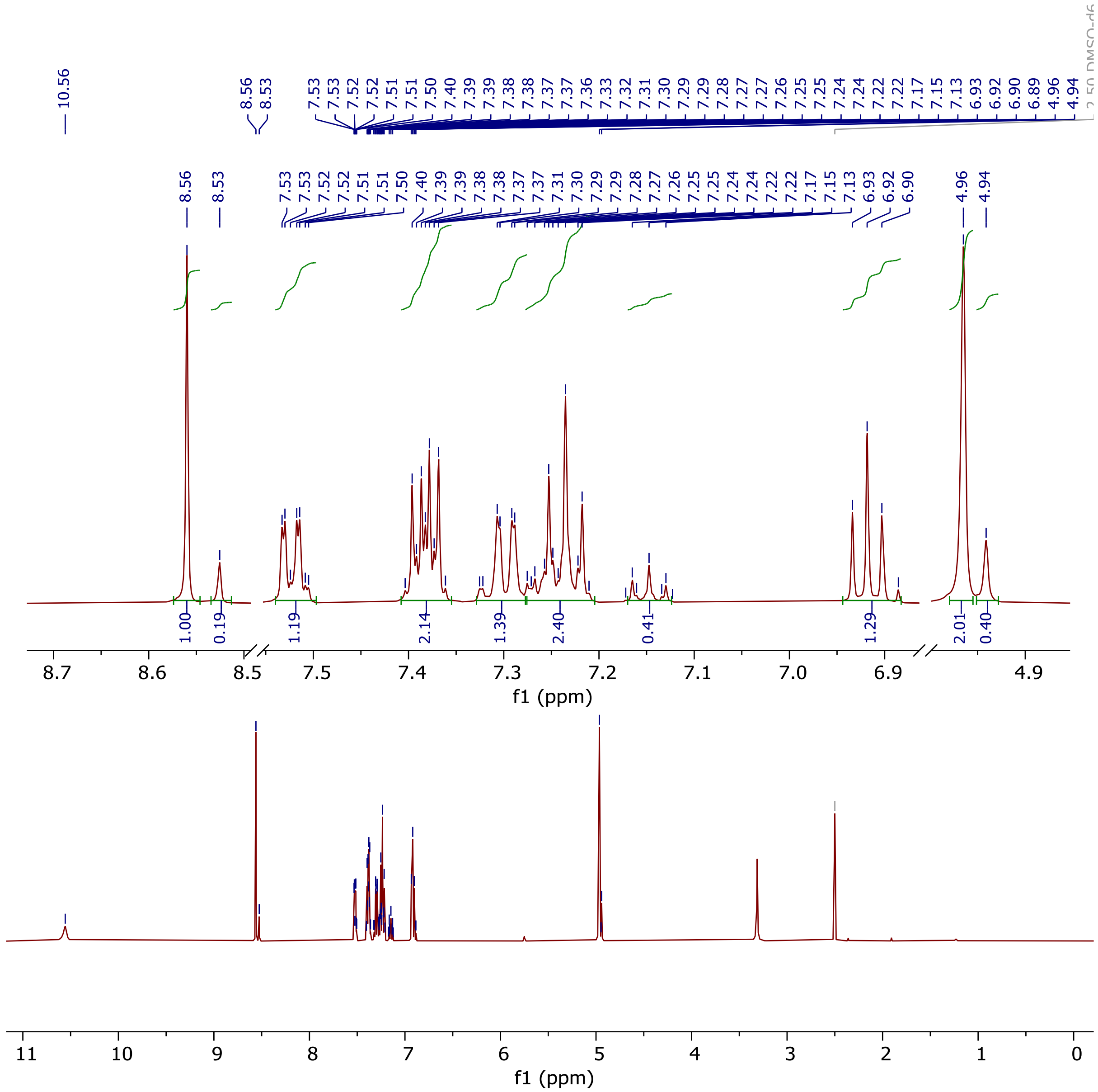

Figure S80: ^{1}H NMR spectrum (DMSO-d_6) of compound **1**-CN.

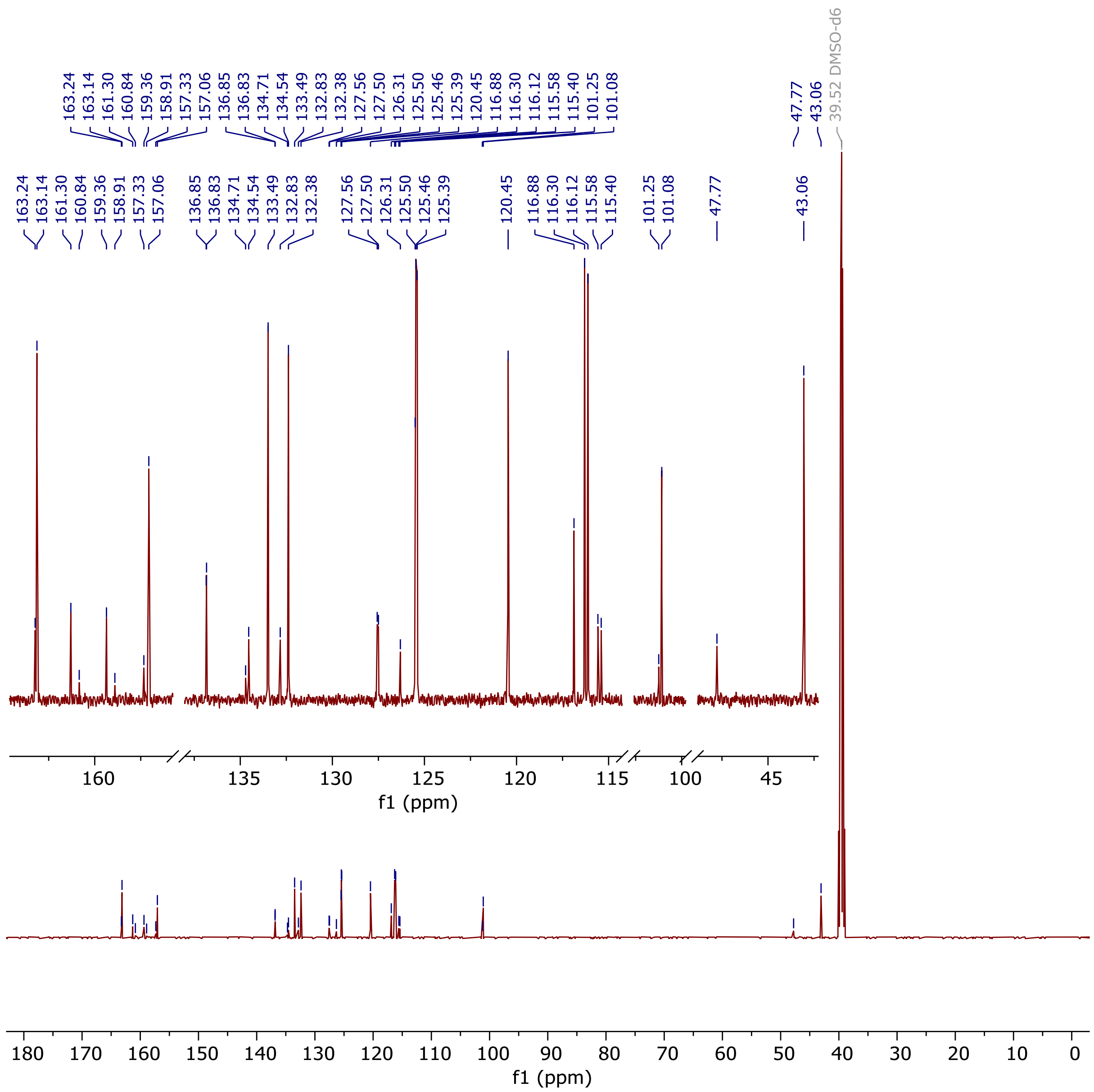

Figure S81: ^{13}C NMR spectrum (DMSO-d_6) of compound **1**-CN.

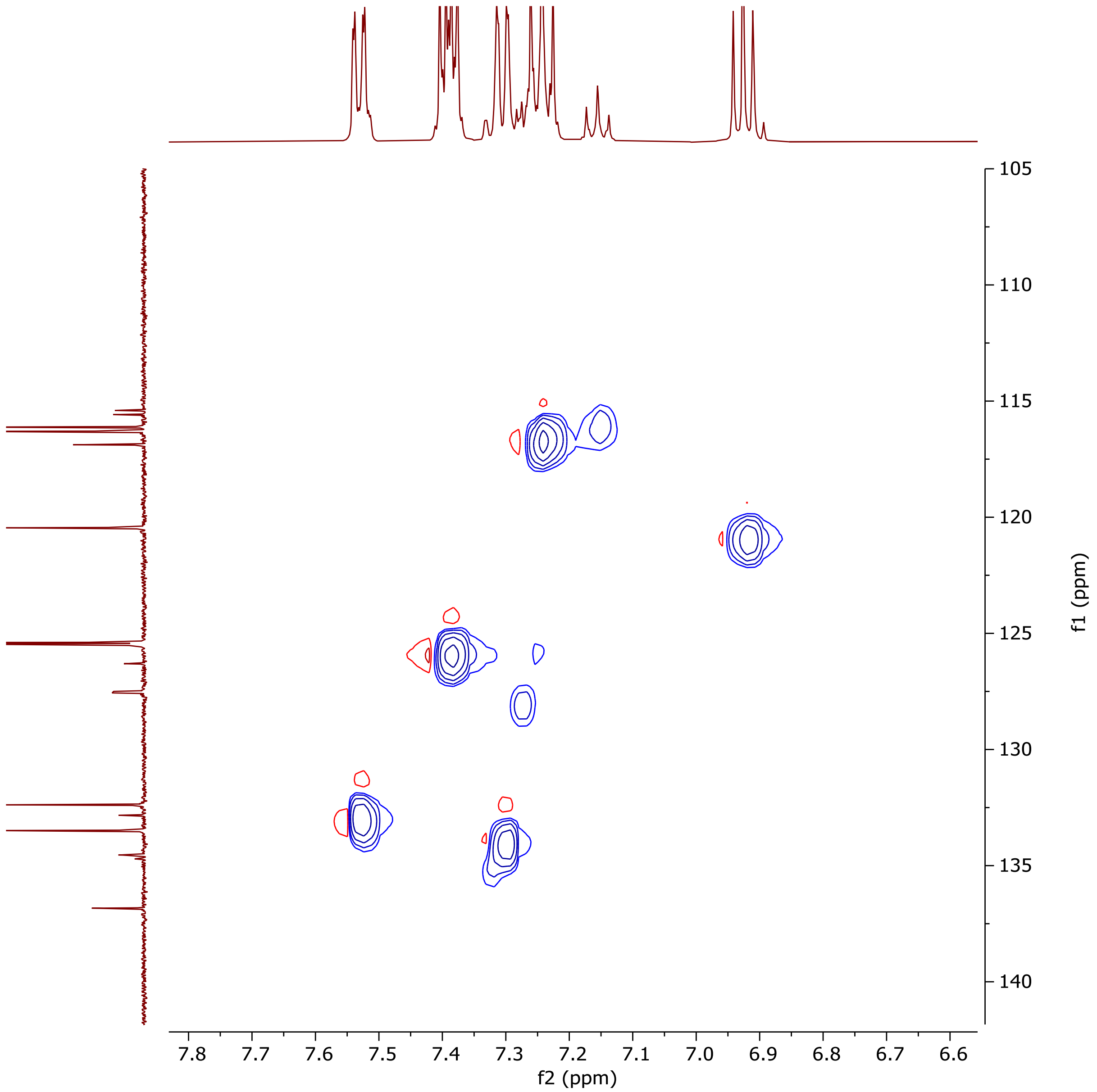

Figure S82: HSQC NMR spectrum (DMSO-d_6) of compound **1**-CN.

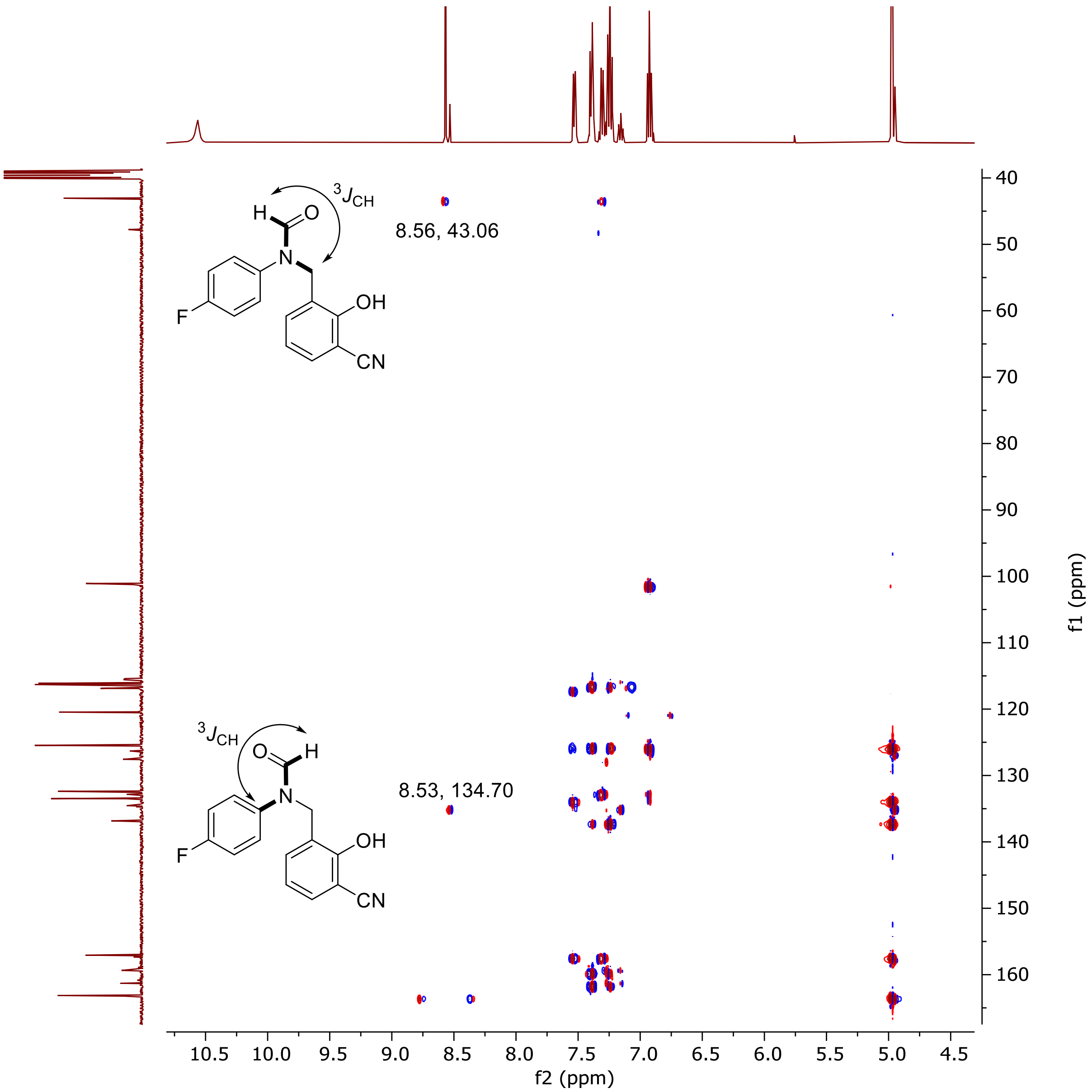

Figure S83: HMBC NMR spectrum (DMSO-d_6) of compound **1**-CN.

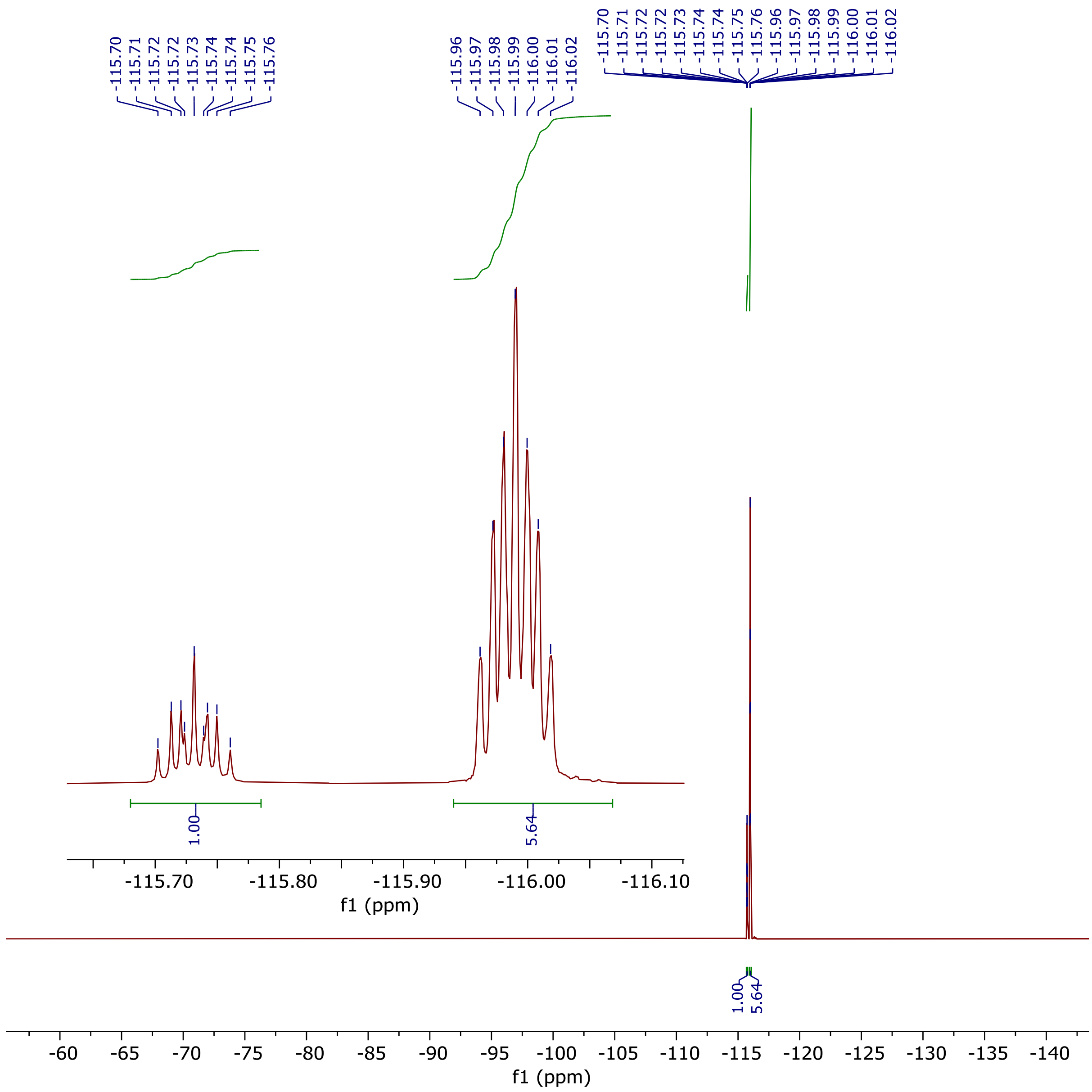

Figure S84: ^{19}F NMR spectrum (DMSO-d_6) of compound **1**-CN.

N-(4-fluorophenyl)-*N*-{[2-hydroxy-3-(trifluoromethyl)phenyl]methyl}formamide (1-CF_3)

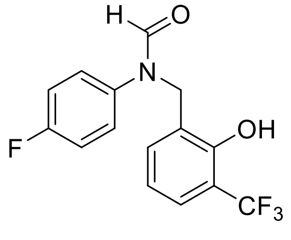

Following general procedure **C**, **3**-CF_3 (2.0 mmol, 626 mg) was dissolved in 2 mL DCM and cooled down to 0 °C. FAM (3 eq, made from 9 mmol formic acid, 340 µL and 6 mmol acetic anhydride, 567 µL) was added in one portion and the reaction was stirred for 8 h. After removal of volatiles, the residue was purified via flash chromatography (eluting with gradient of DCM:MeOH 100:1 to DCM:MeOH 50:1) to afford the title compound as a colourless solid (463 mg, 72%). Mp 91–93 °C. HRMS (ESI+): m/z calcd. for $C_{15}H_{11}F_4NO_2Na$ $[M+Na]^+$: 336.0618, found 336.0620.

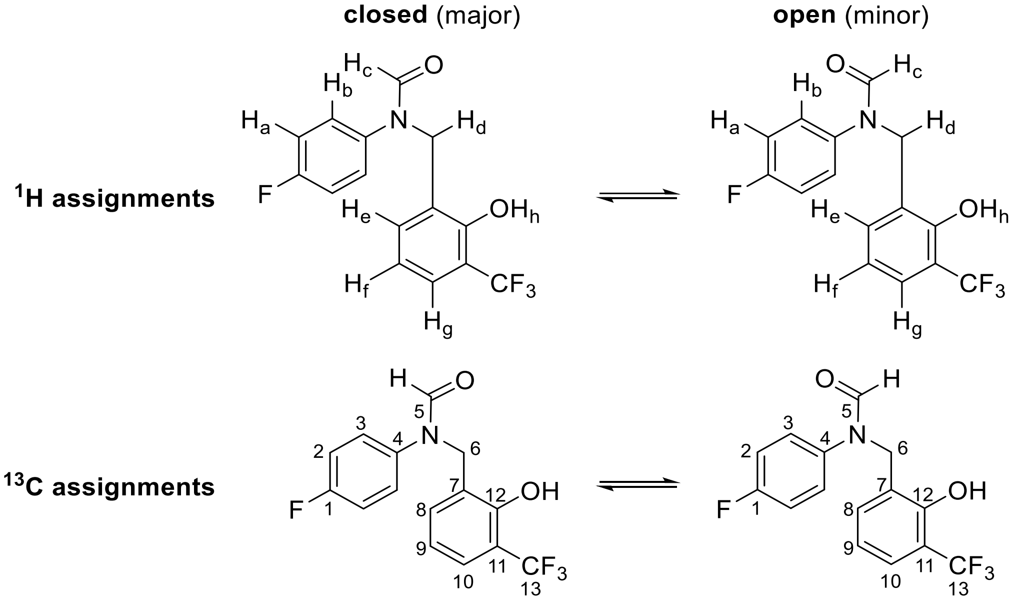

^{1}H NMR (601 MHz, DMSO) δ 9.83 (s, 1H, $\mathbf{H}_{\mathbf{h}\text{ closed}}$), 9.73 (s, 1H, $\mathbf{H}_{\mathbf{h}\text{ open}}$), 8.57 (s, 1H, $\mathbf{H}_{\mathbf{c}\text{ closed}}$), 8.54 (s, 1H, $\mathbf{H}_{\mathbf{c}\text{ open}}$), 7.45 (d, *J* = 7.9 Hz, 1H, $\mathbf{H}_{\mathbf{g}\text{ open}}$), 7.45 (d, *J* = 7.9 Hz, 1H, $\mathbf{H}_{\mathbf{g}\text{ open}}$), 7.42 – 7.38 (m, 2H, $\mathbf{H}_{\mathbf{b}\text{ closed}}$), 7.31 – 7.27 (m, 2H, $\mathbf{H}_{\mathbf{b}\text{ open}}$, $\mathbf{H}_{\mathbf{e}\text{ open}}$), 7.27 – 7.22 (m, 3H, $\mathbf{H}_{\mathbf{a}\text{ closed}}$, $\mathbf{H}_{\mathbf{e}\text{ closed}}$), 7.17 – 7.13 (m, 2H, $\mathbf{H}_{\mathbf{a}\text{ open}}$), 6.93 (t, *J* = 7.7 Hz, 1H, $\mathbf{H}_{\mathbf{f}\text{ closed}}$) 6.93 (t, J = 7.8 Hz, 1H, $\mathbf{H}_{\mathbf{f}\text{ open}}$), 5.01 (s, 2H, $\mathbf{H}_{\mathbf{d}\text{ open}}$), 5.00 (s, 2H, $\mathbf{H}_{\mathbf{d}\text{ closed}}$).

Major conformer

^{13}C NMR (151 MHz, DMSO) δ 163.49 ($\mathbf{C_5}$), 160.43 (d, *J* = 243.3 Hz, $\mathbf{C_1}$), 152.94 (q, *J* = 1.6 Hz, $\mathbf{C_{12}}$), 136.83 (d, *J* = 2.7 Hz, $\mathbf{C_4}$), 132.71 ($\mathbf{C_8}$), 125.81 ($\mathbf{C_7}$), 125.74 (q, *J* = 5.3 Hz, $\mathbf{C_{10}}$), 125.52 (d, *J* = 8.6 Hz, $\mathbf{C_3}$), 123.97 (q, *J* = 272.1 Hz, $\mathbf{C_{13}}$), 119.74 ($\mathbf{C_9}$), 118.03 (q, *J* = 29.3 Hz, $\mathbf{C_{11}}$), 116.28 (d, *J* = 22.7 Hz, $\mathbf{C_2}$), 43.51 ($\mathbf{C_6}$). ^{19}F NMR (471 MHz, DMSO) δ -60.51, -115.85 (td, *J* = 8.7, 4.5 Hz).

Minor conformer

^{13}C NMR (151 MHz, DMSO) δ 163.23 ($\mathbf{C_5}$), 159.86 (d, *J* = 243.3 Hz, $\mathbf{C_1}$), 152.94 (q, *J* = 1.6 Hz, $\mathbf{C_{12}}$), 134.83 (d, *J* = 2.8 Hz, $\mathbf{C_4}$), 133.27 ($\mathbf{C_8}$), 127.37 (d, *J* = 8.5 Hz, $\mathbf{C_3}$), 127.02 ($\mathbf{C_7}$), 125.89 (q, *J* = 5.2 Hz, $\mathbf{C_{10}}$), 123.97 (q, *J* = 272.4 Hz, $\mathbf{C_{13}}$), 119.98 ($\mathbf{C_9}$), 118.41 (q, *J* = 28.8 Hz, $\mathbf{C_{11}}$), 115.49 (d, *J* = 22.5 Hz, $\mathbf{C_2}$), 47.66 ($\mathbf{C_6}$). ^{19}F NMR (471 MHz, DMSO) δ -60.22, -115.80 (td, *J* = 8.7, 4.5 Hz).

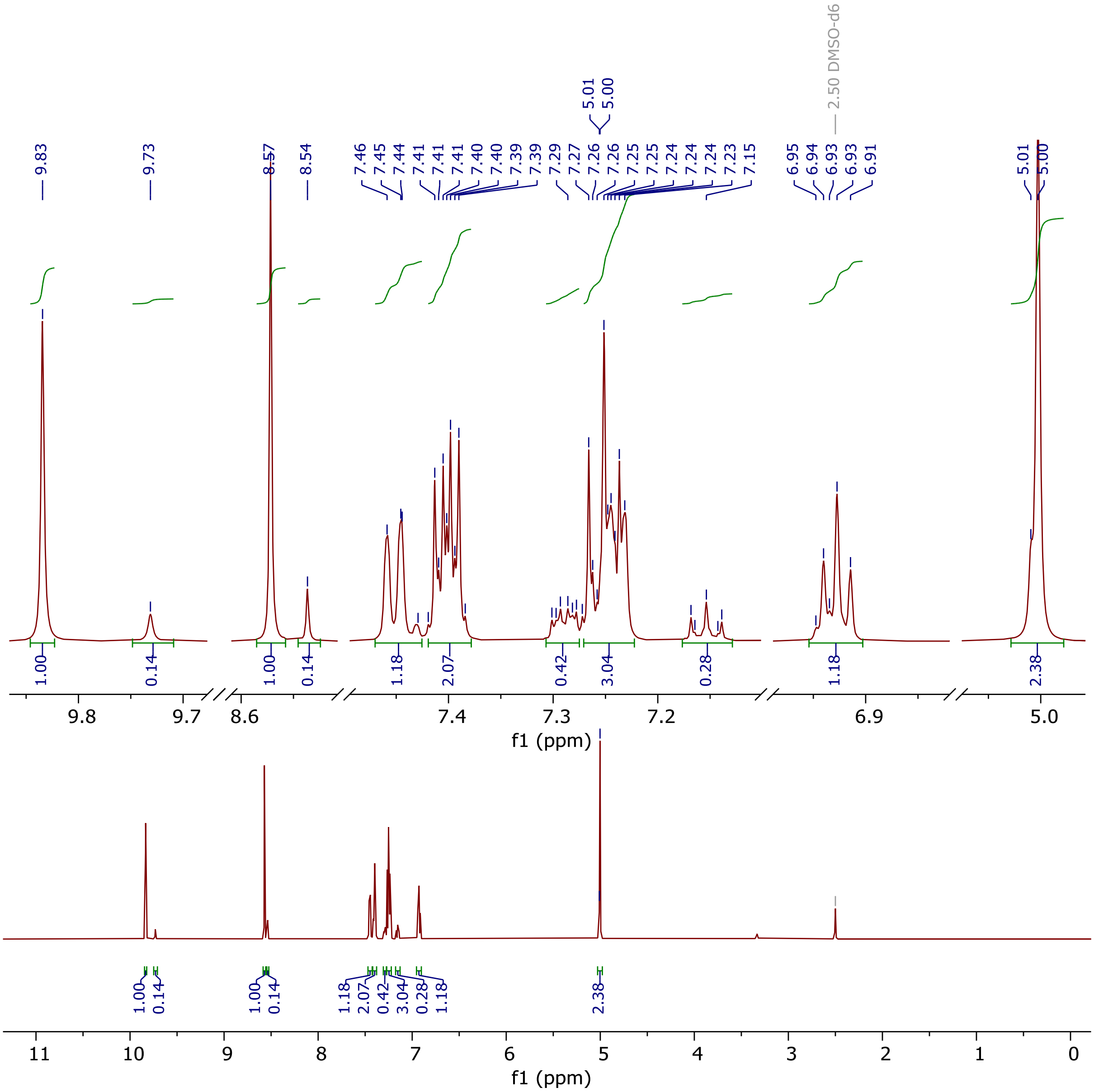

Figure S85: ^{1}H NMR spectrum (DMSO-d_6) of compound **1**-CF_3.

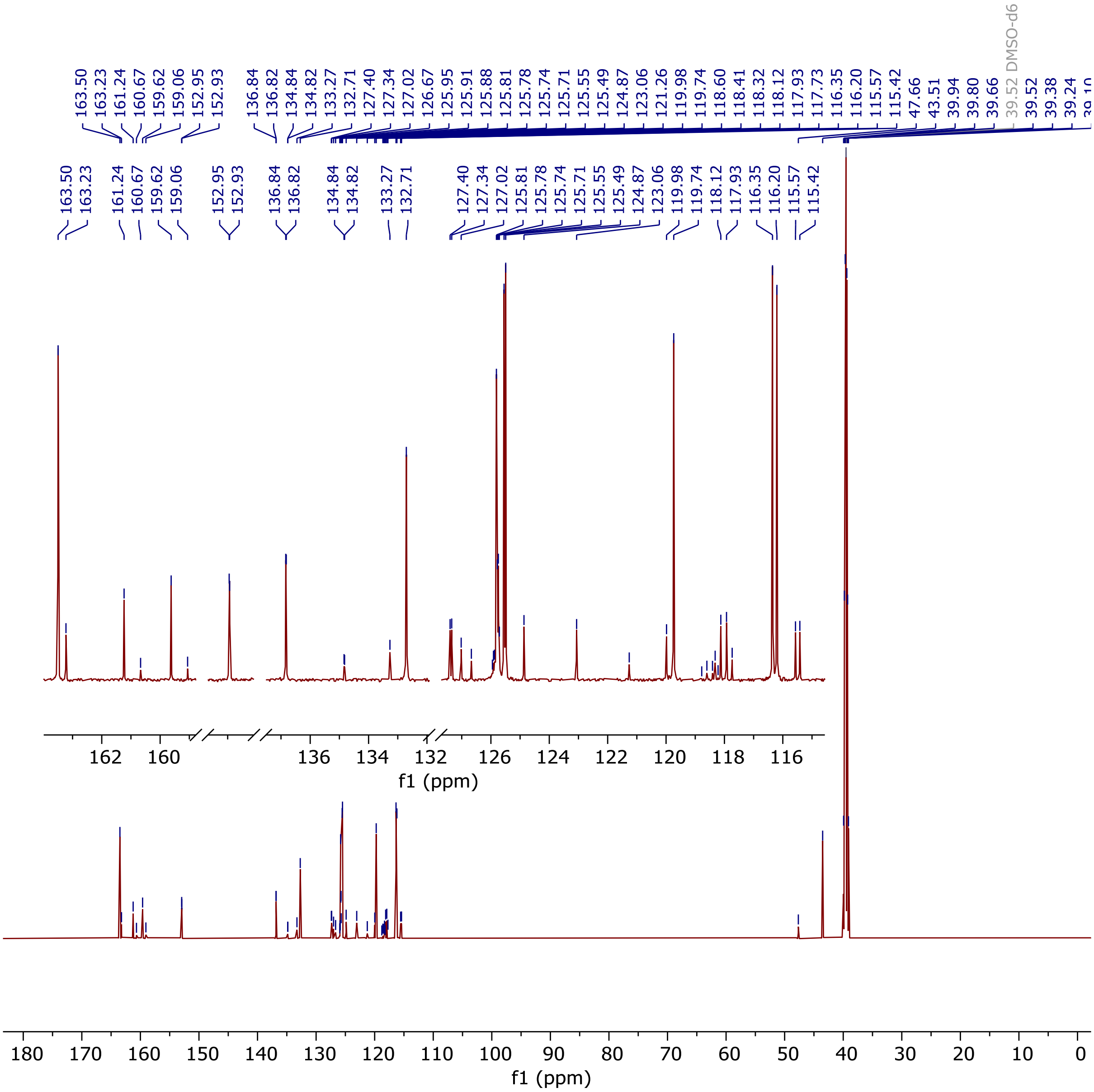

Figure S86: ^{13}C NMR spectrum (DMSO-d_6) of compound **1**-CF_3.

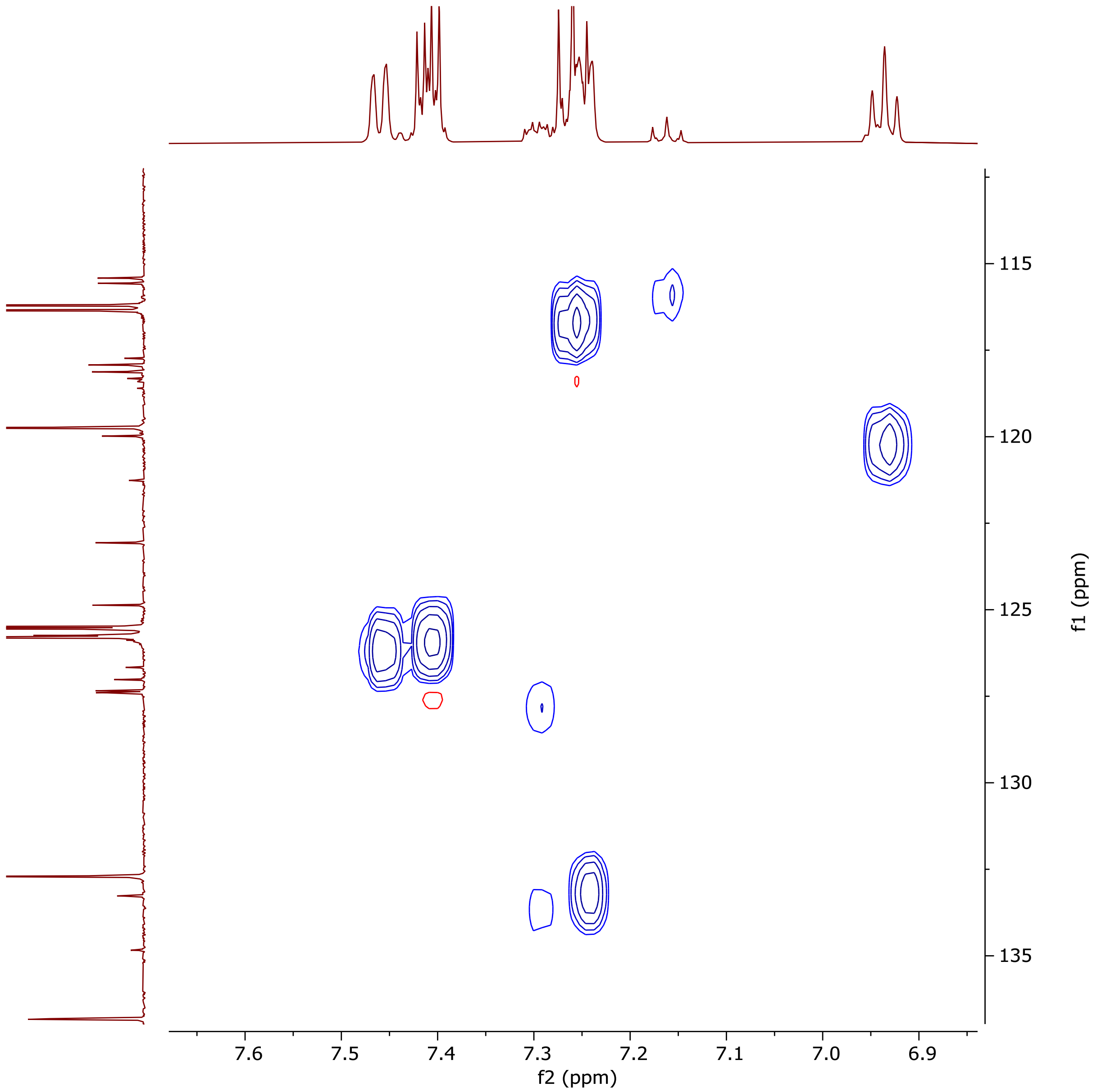

Figure S87: HSQC NMR spectrum (DMSO-d_6) of compound **1**-CF_3.

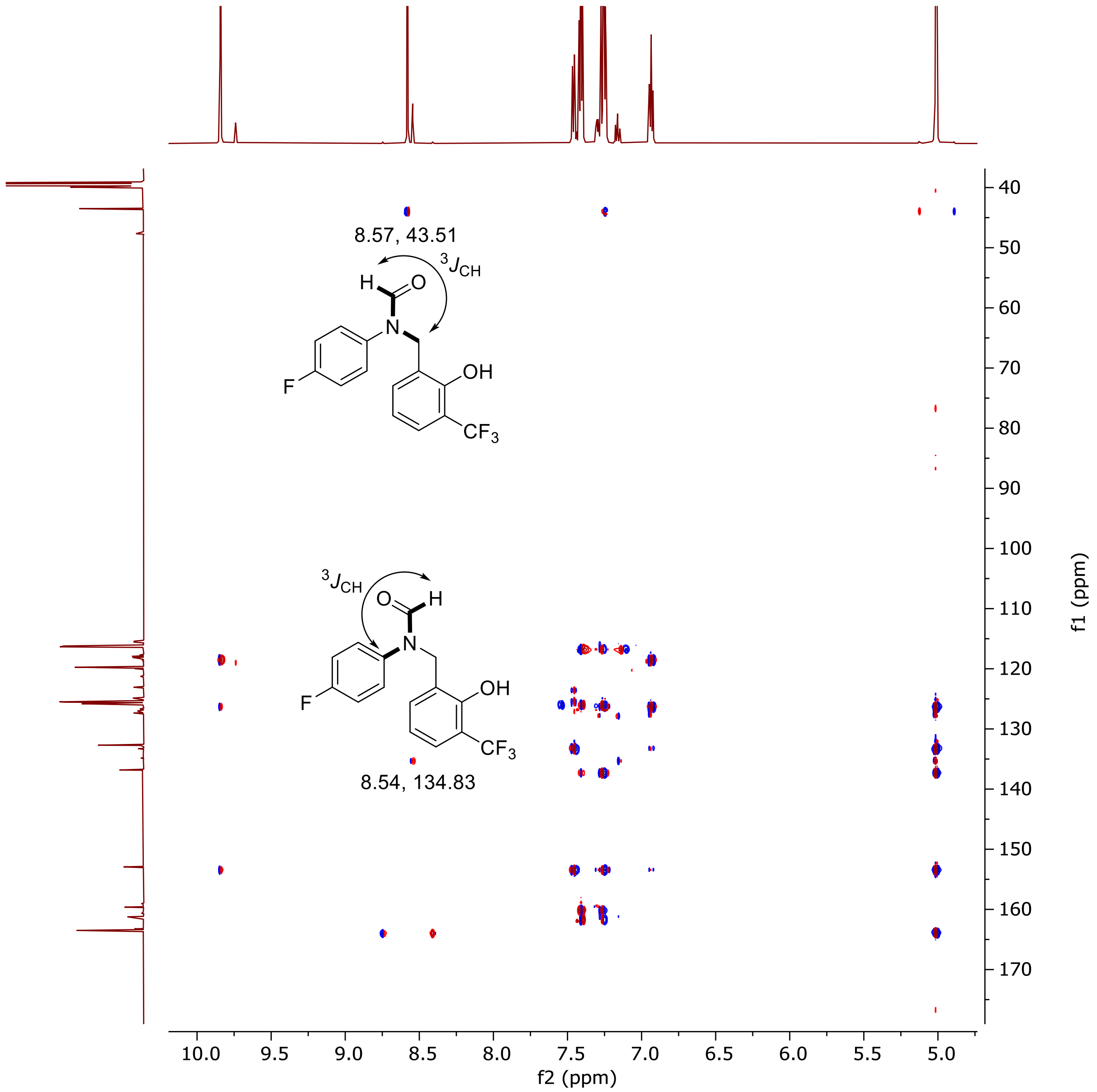

Figure S88: HMBC NMR spectrum (DMSO-d_6) of compound **1**-CF_3.

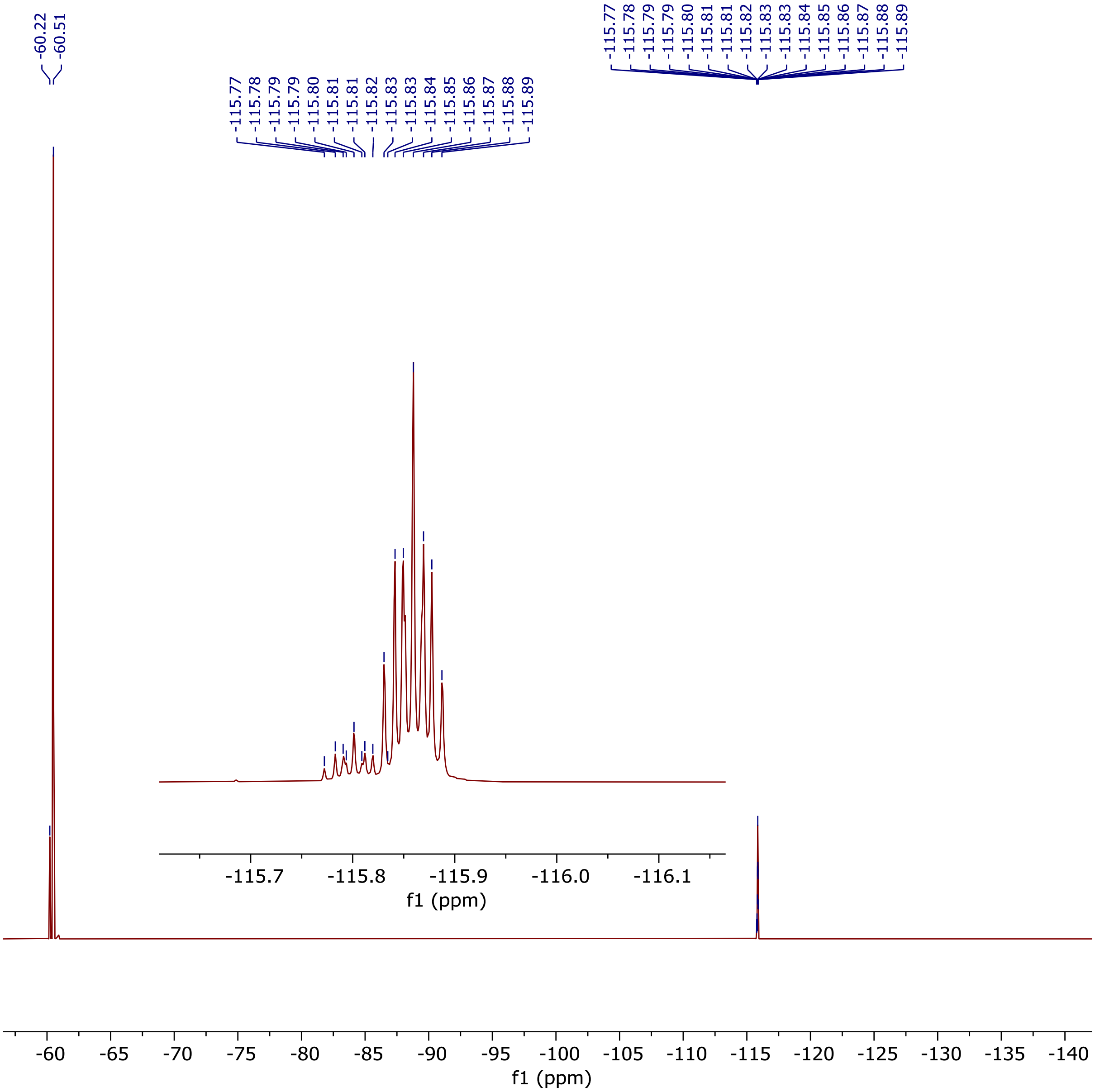

Figure S89: ^{19}F NMR spectrum (DMSO-d_6) of compound **1**-CF_3.

***N*-benzyl-*N*-(4-fluorophenyl)formamide (1-control)**

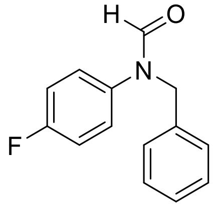

Following general procedure C, compound **3**-control (3.0 mmol, 603 mg) was dissolved in 2 mL DCM and cooled down to 0 °C. FAM (3 eq, made from 9 mmol formic acid, 340 µL and 6 mmol acetic anhydride, 567 µL) was added in one portion and the reaction was stirred for 8 h. After removal of volatiles, the residue was purified via flash chromatography (eluent 100% $CHCl_3$) to afford the title compound as a colourless oil (557 mg, 81%). HRMS (ESI+): m/z calcd. for $C_{14}H_{13}FNO$ $[M+H]^+$: 230.0976, found 230.0985.

closed (major) **open** (minor)

^{1}H assignments

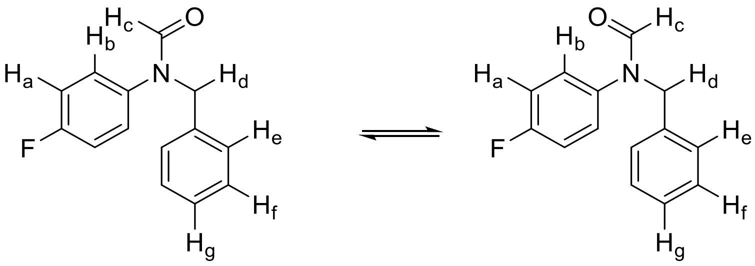

^{13}C assignments

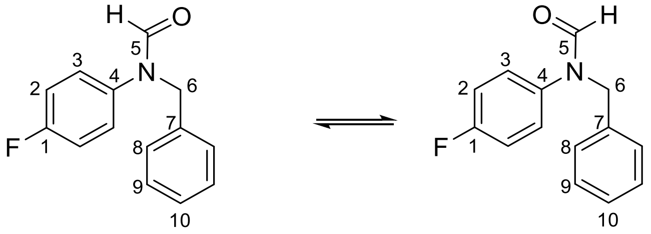

^{1}H NMR (500 MHz, DMSO) δ 8.59 (s, 1H, $\mathbf{H}_{\mathbf{c}\text{ open}}$), 8.57 (s, 1H, $\mathbf{H}_{\mathbf{c}\text{ closed}}$), 7.39 – 7.35 (m, 4H, $\mathbf{H}_{\mathbf{b}\text{ closed}}$, $\mathbf{H}_{\mathbf{b}\text{ open}}$), 7.35 – 7.31 (m, 2H, $\mathbf{H}_{\mathbf{e}\text{ open}}$), 7.31 – 7.26 (m, 4H, $\mathbf{H}_{\mathbf{f}\text{ closed}}$, $\mathbf{H}_{\mathbf{f}\text{ open}}$), 7.25 – 7.18 (m, 4H, $\mathbf{H}_{\mathbf{a}\text{ closed}}$, $\mathbf{H}_{\mathbf{e}\text{ closed}}$, $\mathbf{H}_{\mathbf{g}\text{ open}}$, $\mathbf{H}_{\mathbf{g}\text{ closed,}}$), 7.17 – 7.12 (m, 2H, $\mathbf{H}_{\mathbf{a}\text{ open}}$), 5.01 (s, 2H, $\mathbf{H}_{\mathbf{d}\text{ closed}}$), 4.96 (s, 2H, $\mathbf{H}_{\mathbf{d}\text{ open}}$).

Closed conformer (major):

^{13}C NMR (126 MHz, DMSO) δ 162.40 (**C$_5$**), 160.12 (d, *J* = 243.1 Hz, **C$_1$**), 137.04 (d, *J* = 2.5 Hz, **C$_4$**), 136.74 (**C$_7$**), 128.44 (**C$_9$**), 127.36 (**C$_8$**), 127.11 (**C$_{10}$**), 125.33 (d, *J* = 8.5 Hz, **C$_3$**), 116.09 (d, *J* = 22.6 Hz, **C$_2$**), 46.89 (**C$_6$**). ^{19}F NMR (471 MHz, DMSO) δ -116.45 (tt, *J* = 8.9, 4.8 Hz).

Open conformer (minor):

^{13}C NMR (126 MHz, DMSO) δ 162.98 (**C$_5$**), 159.73 (d, *J* = 243.3 Hz, **C$_1$**), 136.74 (**C$_7$**), 134.96 (d, *J* = 3.0 Hz, **C$_4$**), 128.52 (**C$_9$**), 127.49 (**C$_8$**), 127.29 (**C$_{10}$**), 125.33 (d, *J* = 8.5 Hz, **C$_3$**), 115.41 (d, *J* = 22.4 Hz, **C$_2$**), 52.22 (**C$_6$**). ^{19}F NMR (471 MHz, DMSO) δ -115.99 (ddd, *J* = 13.7, 8.7, 5.0 Hz).

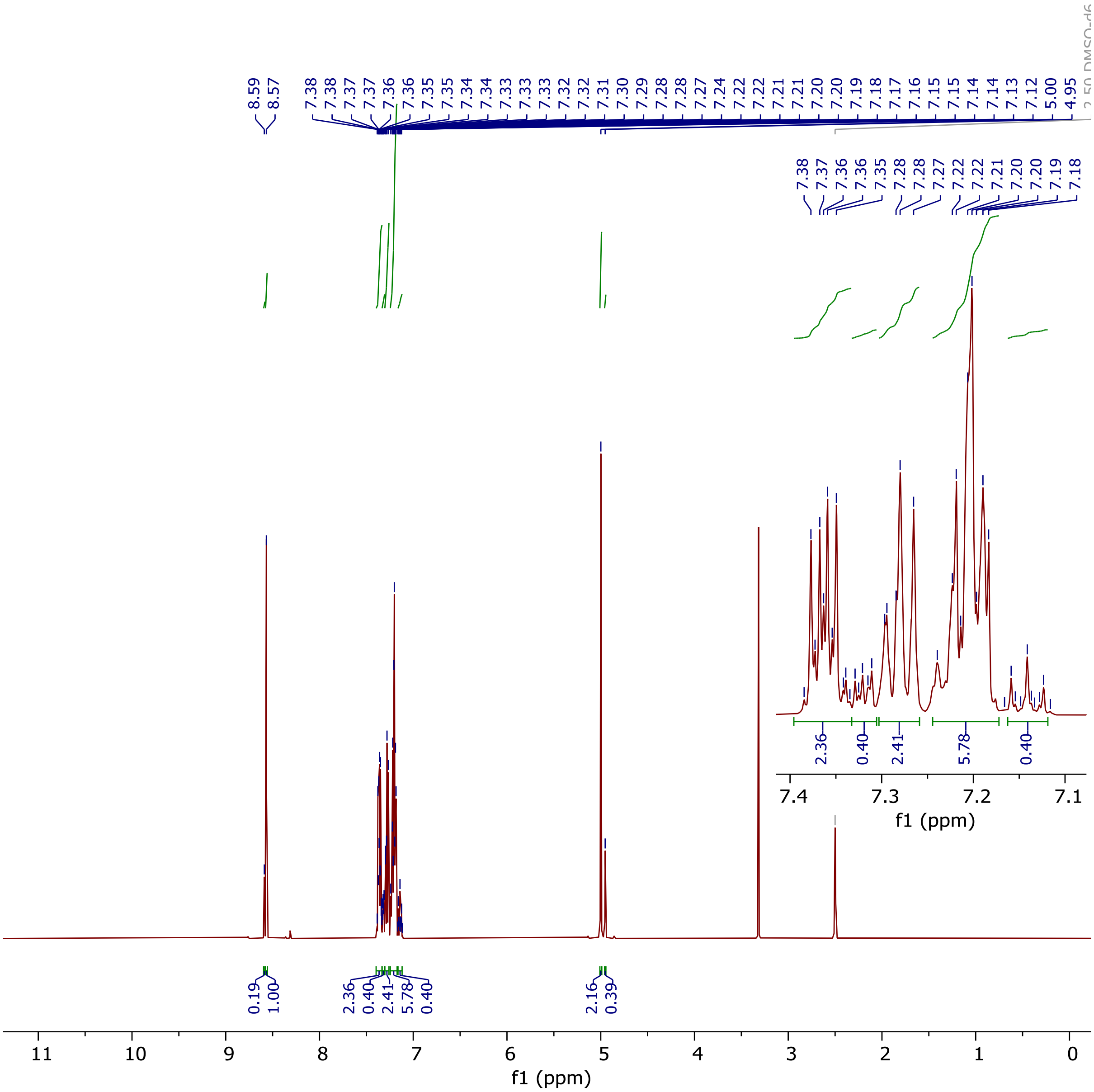

Figure S90: ^{1}H NMR spectrum (DMSO-d_6) of compound **1**-control.

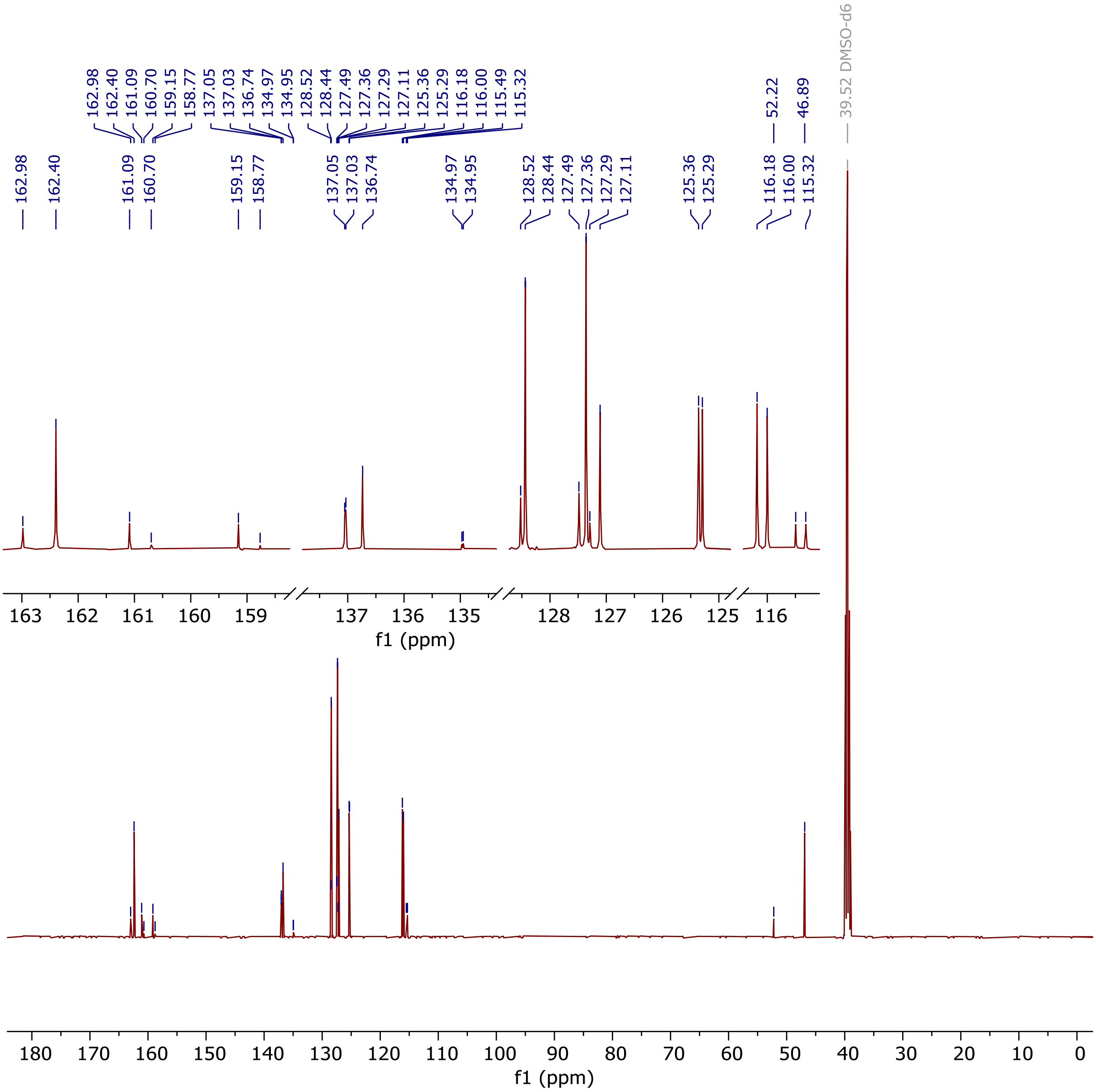

Figure S91: ^{13}C NMR spectrum (DMSO-d_6) of compound **1**-control.

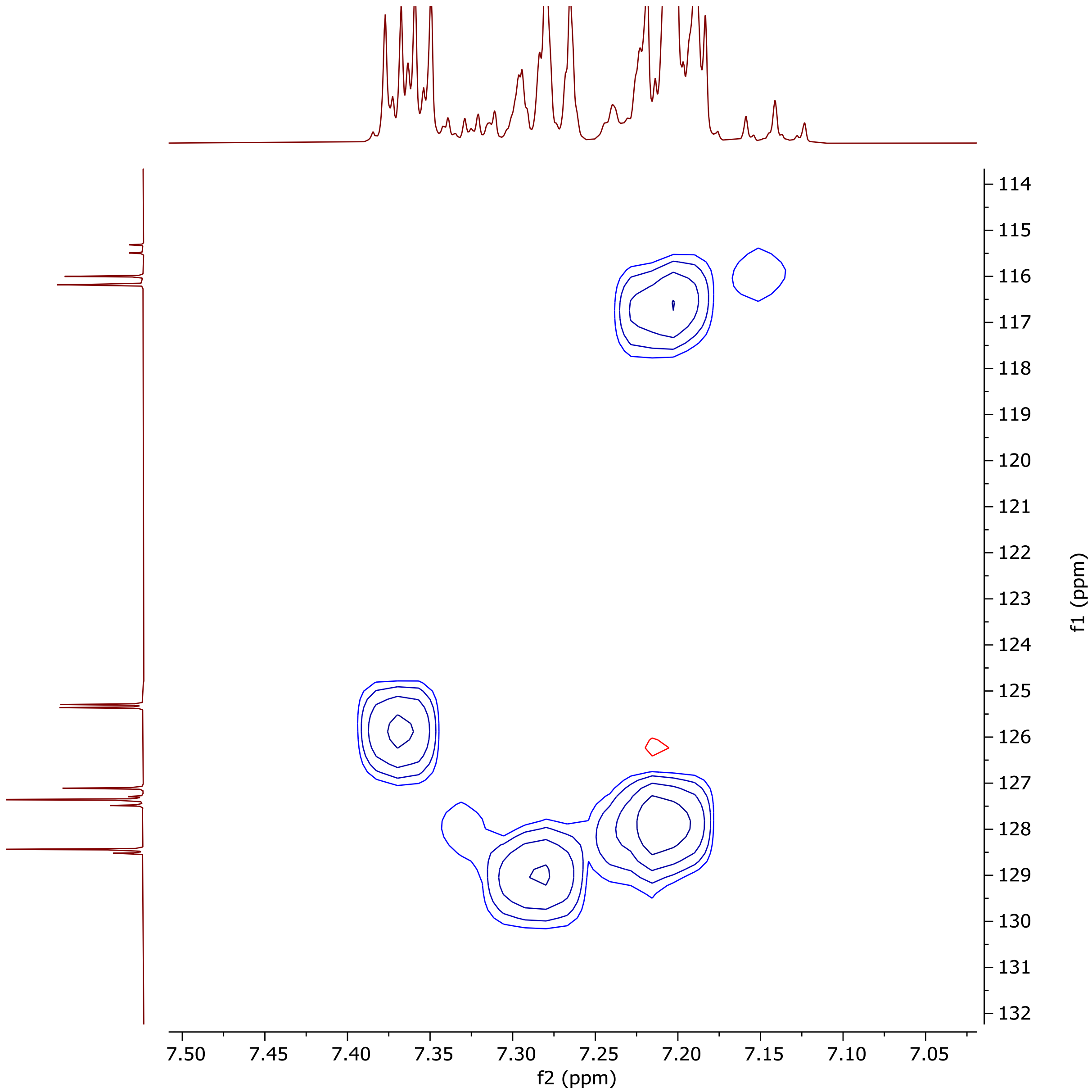

Figure S92: HSQC NMR spectrum (DMSO-d_6) of compound **1**-control.

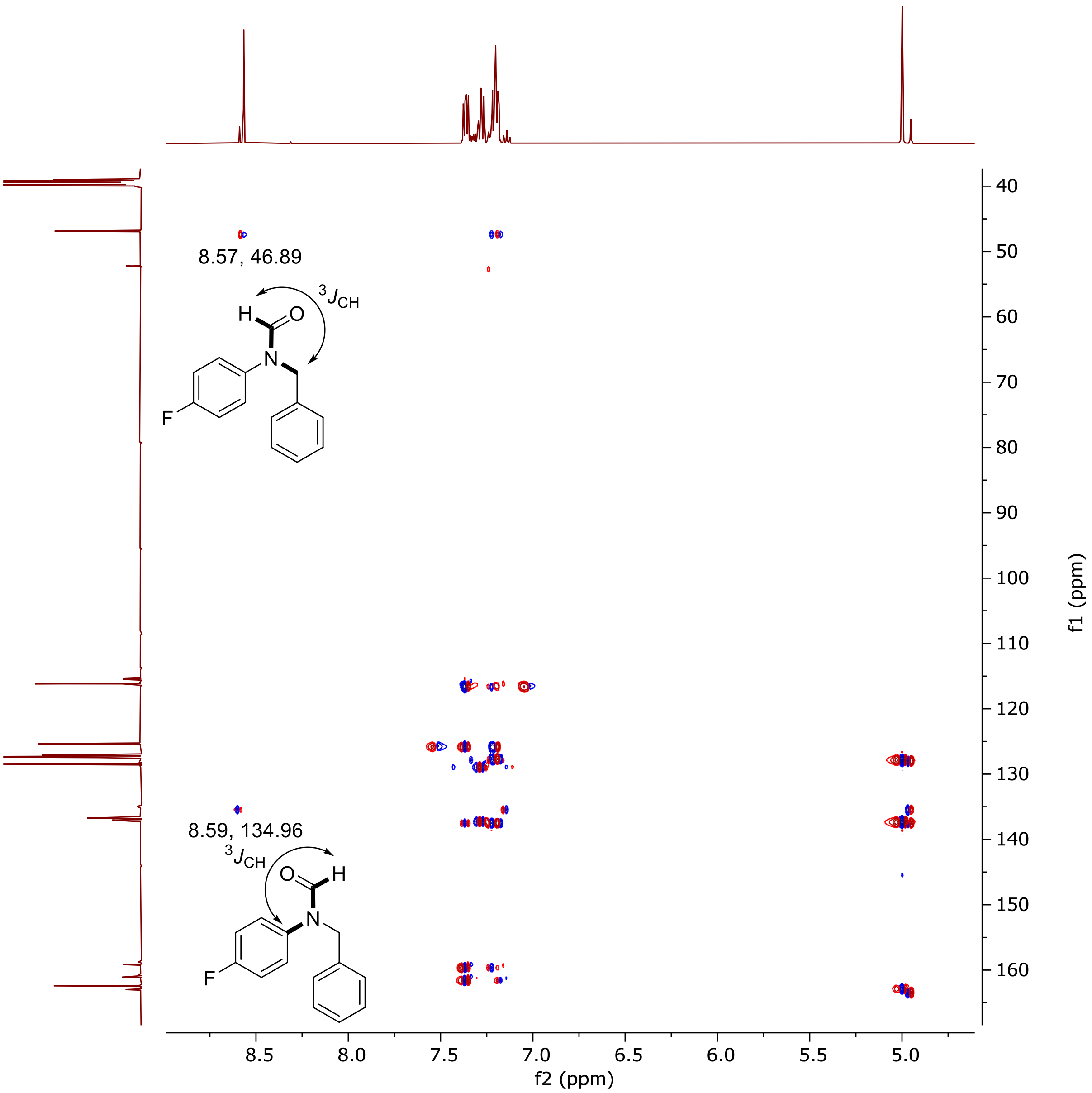

Figure S93: HMBC NMR spectrum (DMSO-d_6) of compound **1**-control.

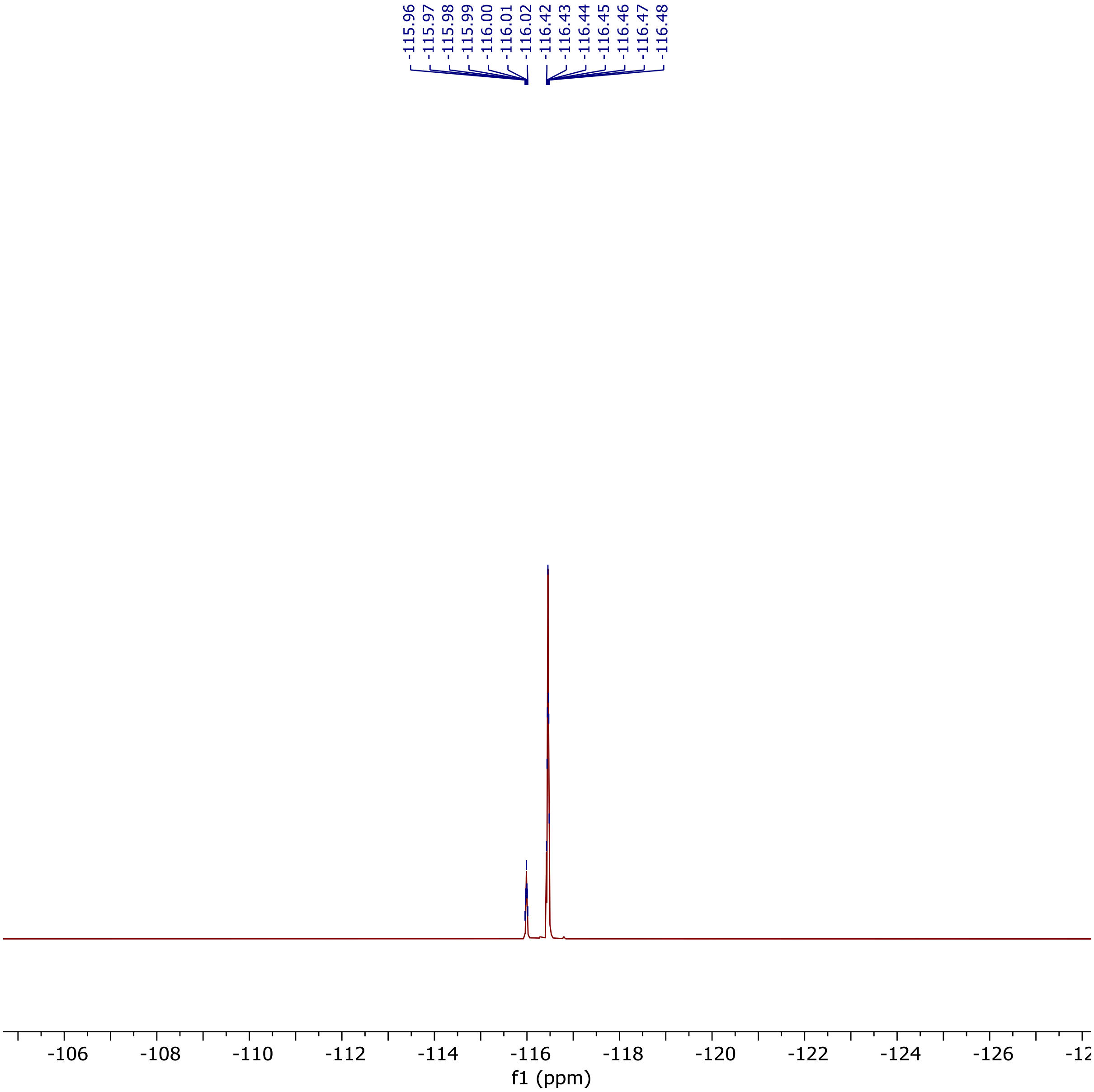

Figure S94: ^{19}F NMR spectrum (DMSO-d_6) of compound **1**-control.

N-[(3-bromo-2-hydroxyphenyl)methyl]-*N*-(4-fluorophenyl)formamide (1-Br)

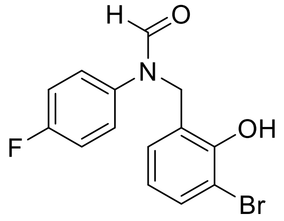

Following general procedure C, **3**-Br (3.0 mmol, 890 mg) was dissolved in 2 mL DCM and cooled down to 0 °C. FAM (3 eq, made from 13.5 mmol formic acid, 510 µL and 9 mmol acetic anhydride, 850 µL) was added in one portion and the reaction was stirred for 8 h. After removal of volatiles, the residue was purified via flash chromatography (eluting with gradient of DCM:MeOH 100:0 to DCM:MeOH 100:1) to afford the title compound as a colourless oil which slowly solidified to give a colourless solid (875 mg, 90%). Mp 75 – 77 °C. HRMS (ESI+): m/z calcd. for $C_{14}H_{12}BrFNO_2$ $[M+H]^+$: 324.0030, found 324.0043.

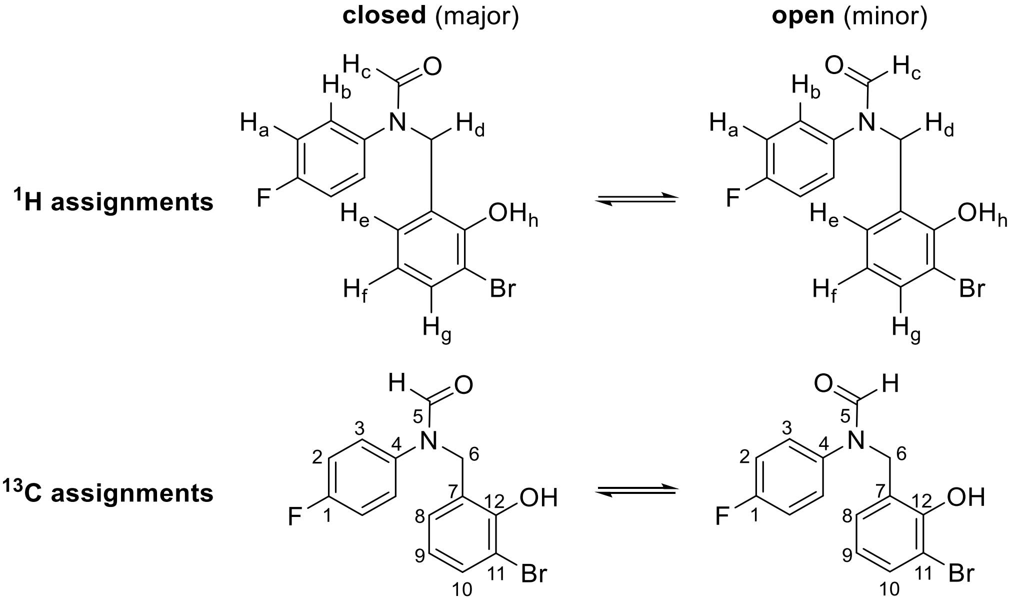

^{1}H NMR (500 MHz, DMSO) δ 9.38 (s, 2H, $\mathbf{H_h}$ closed, $\mathbf{H_h}$ open), 8.57 (s, 1H, $\mathbf{H_c}$ closed), 8.52 (s, 1H, $\mathbf{H_c}$ open), 7.42 – 7.34 (m, 4H, $\mathbf{H_b}$ closed, $\mathbf{H_e}$ closed, $\mathbf{H_e}$ open), 7.32 – 7.24 (m, 2H, $\mathbf{H_b}$ open), 7.25 – 7.20 (m, 2H, $\mathbf{H_a}$ closed), 7.19 – 7.10 (m, 2H, $\mathbf{H_a}$ open), 6.99 (d, *J* = 7.5 Hz, 2H, $\mathbf{H_g}$ closed, $\mathbf{H_g}$ open), 6.72 (t, *J* = 7.8 Hz, 1H, $\mathbf{H_f}$ closed), 6.70 (t, *J* = 7.7 Hz, 1H, $\mathbf{H_f}$ open), 4.97 (s, 2H, $\mathbf{H_d}$ closed), 4.92 (s, 2H, $\mathbf{H_d}$ open).

Closed conformer (major):

^{13}C NMR (126 MHz, DMSO) δ 162.88 ($\mathbf{C_5}$), 160.23 (d, *J* = 243.2 Hz, $\mathbf{C_1}$), 151.25 ($\mathbf{C_{12}}$), 137.06 (d, *J* = 2.8 Hz, $\mathbf{C_4}$), 131.67 ($\mathbf{C_8}$), 127.68 ($\mathbf{C_{10}}$), 125.88 ($\mathbf{C_7}$), 125.27 (d, *J* = 8.5 Hz, $\mathbf{C_3}$), 121.13 ($\mathbf{C_9}$), 116.15 (d, *J* = 22.7 Hz, $\mathbf{C_2}$), 111.41 ($\mathbf{C_{11}}$), 43.64 ($\mathbf{C_6}$). ^{19}F NMR (471 MHz, DMSO) δ -116.23 (td, *J* = 8.9, 4.7 Hz).

Open conformer (minor):

^{13}C NMR (126 MHz, DMSO) δ 163.27 ($\mathbf{C_5}$), 159.81 (d, *J* = 242.9 Hz, $\mathbf{C_1}$), 151.78 ($\mathbf{C_{12}}$), 134.92 (d, *J* = 2.9 Hz, $\mathbf{C_4}$), 132.15 ($\mathbf{C_8}$), 128.87 ($\mathbf{C_{10}}$), 127.45 (d, *J* = 7.9 Hz, $\mathbf{C_3}$), 126.49 ($\mathbf{C_7}$), 121.13 ($\mathbf{C_9}$), 115.42 (d, *J* = 22.5 Hz, $\mathbf{C_2}$), 111.45 ($\mathbf{C_{11}}$), 48.74 ($\mathbf{C_6}$). ^{19}F NMR (471 MHz, DMSO) δ -115.92 (td, *J* = 8.5, 5.1 Hz).

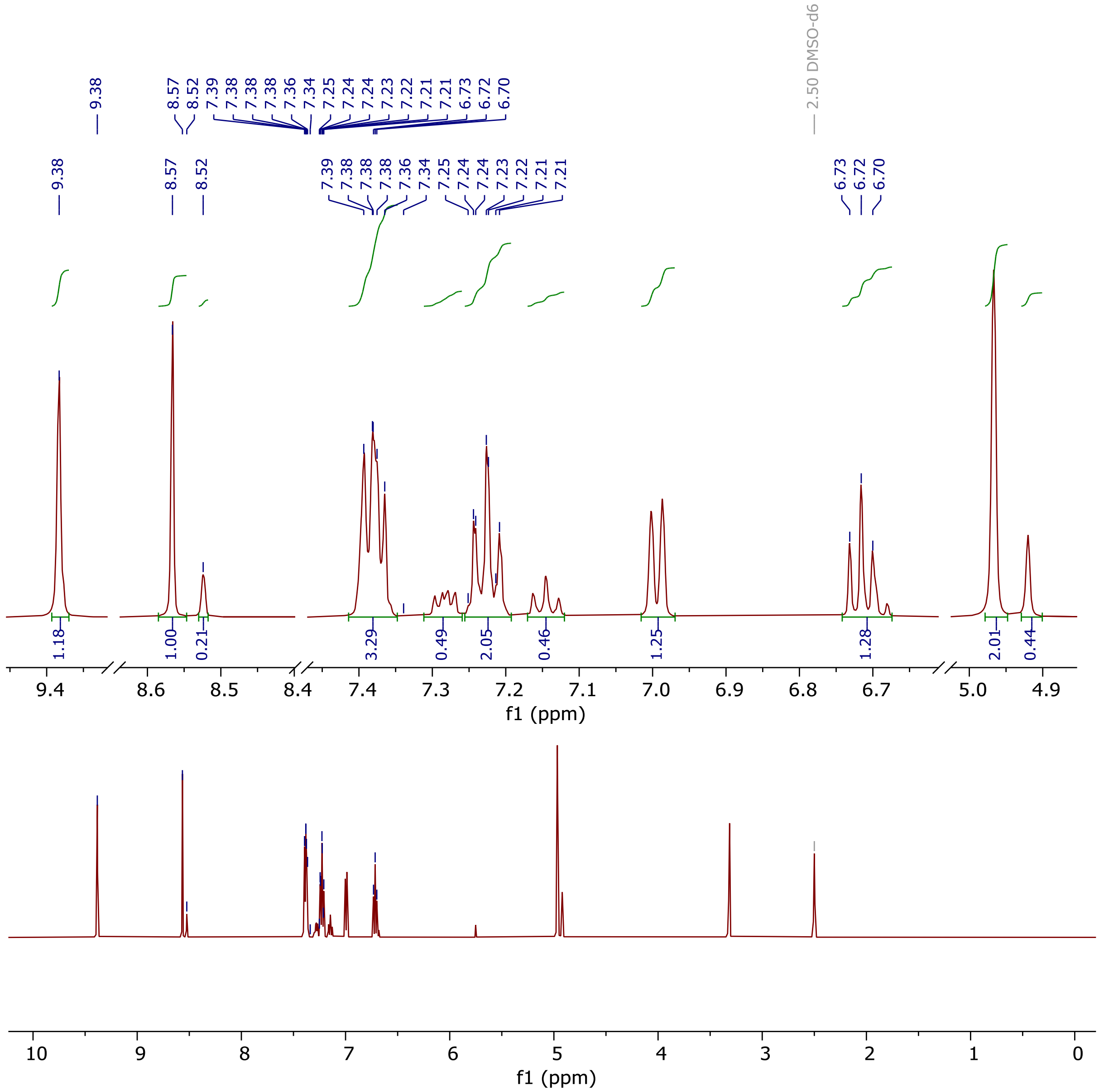

Figure S95: ^{1}H NMR spectrum (*DMSO*-d_6) of compound **1**-Br.

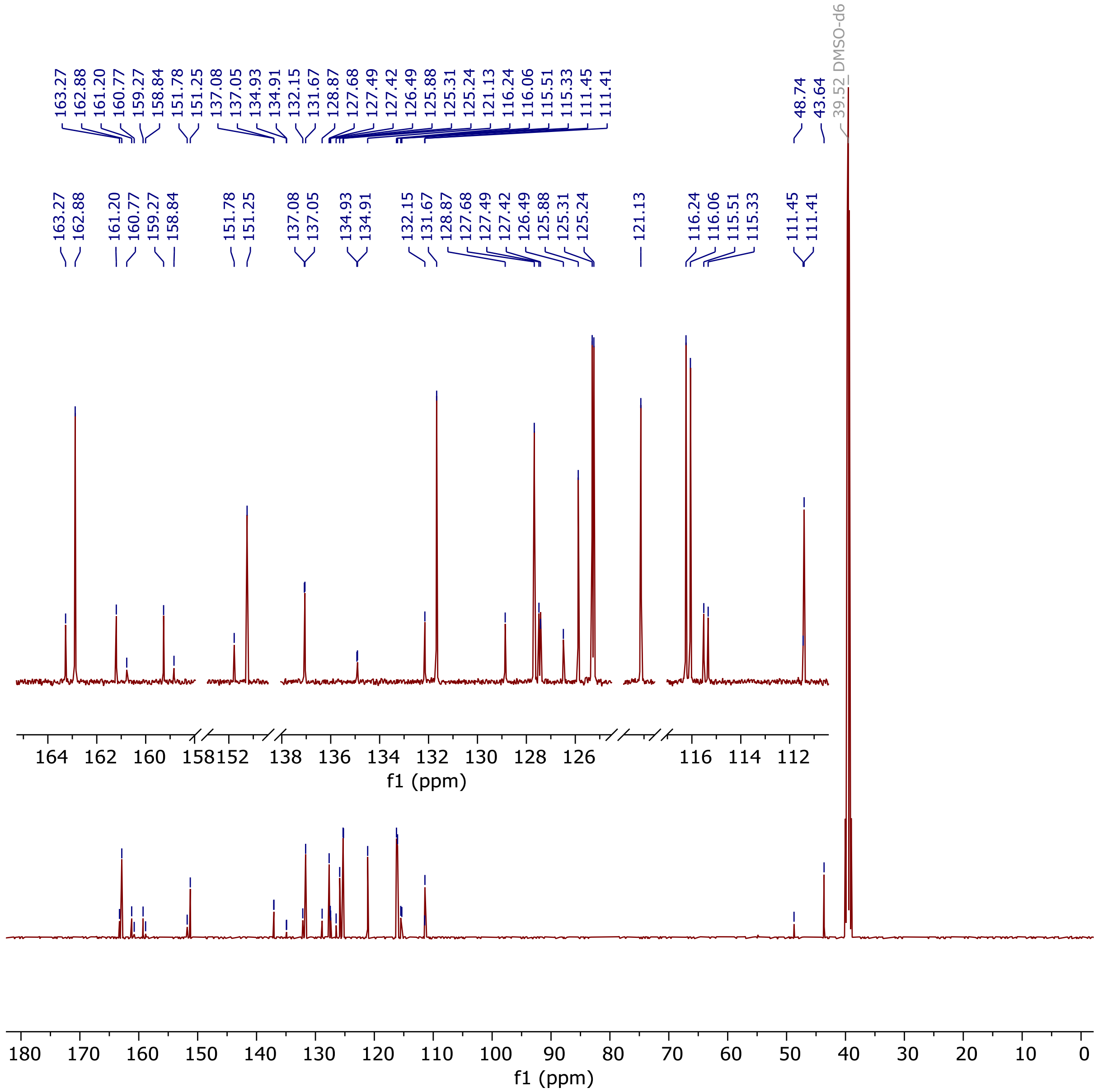

Figure S96: ^{13}C NMR spectrum (DMSO-d_6) of compound **1**-Br.

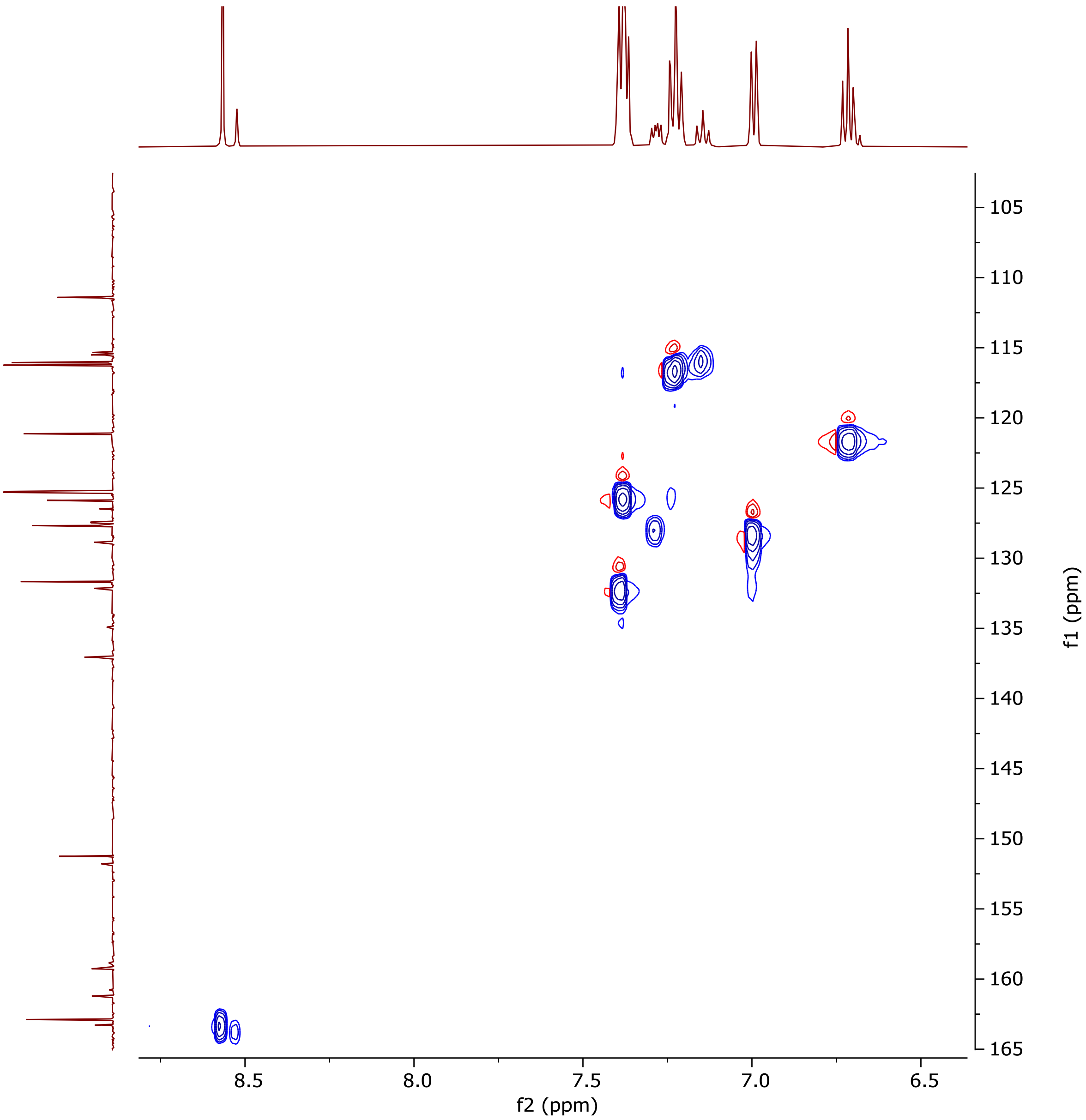

Figure S97: HSQC spectrum (DMSO-d_6) of compound **1**-Br.

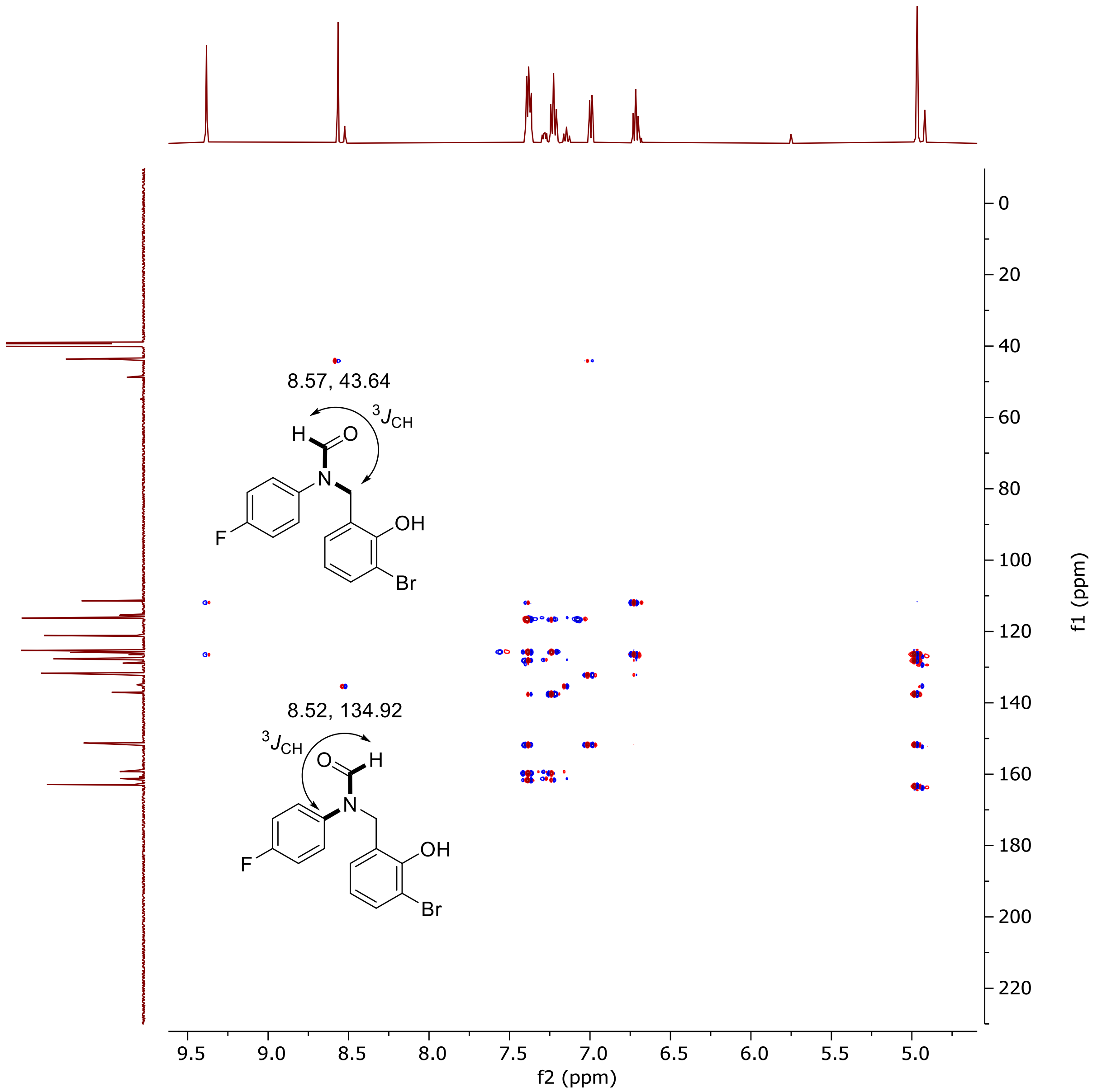

Figure S98: HMBC NMR spectrum (DMSO-d_6) of compound **1**-Br.

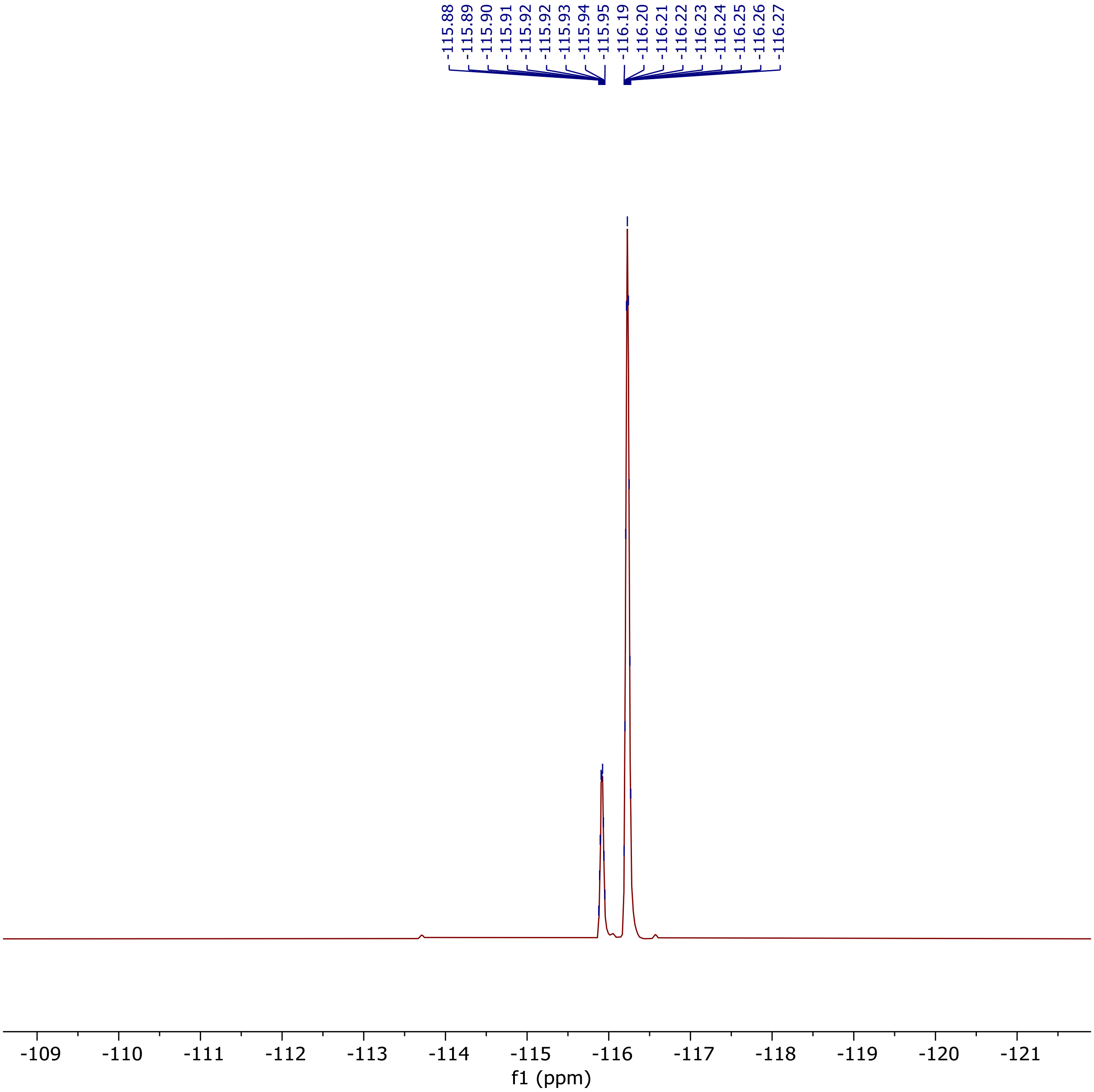

Figure S99: ^{19}F NMR spectrum (DMSO-d_6) of compound **1**-Br.

N-(4-fluorophenyl)-*N*-[(2-hydroxy-3-nitrophenyl)methyl]formamide (1-NO_2)

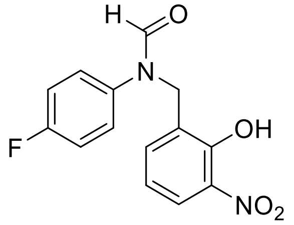

Following general procedure C, **3**-NO_2 (3.0 mmol, 785 mg) was dissolved in 2 mL DCM and cooled down to 0 °C. FAM (3 eq, made from 13.5 mmol formic acid, 510 µL and 9 mmol acetic anhydride, 850 µL) was added in one portion and the reaction was stirred for 8 h. After removal of volatiles, the residue was purified via flash chromatography (eluting with gradient of DCM:MeOH 100:0 to DCM:MeOH 50:1) to afford the title compound as a yellow solid (783 mg, 90%). Mp 91 – 93 °C. HRMS (ESI+): m/z calcd. for $C_{14}H_{12}FN_2O_4$ $[M+H]^+$: 291.0776, found 291.0783.

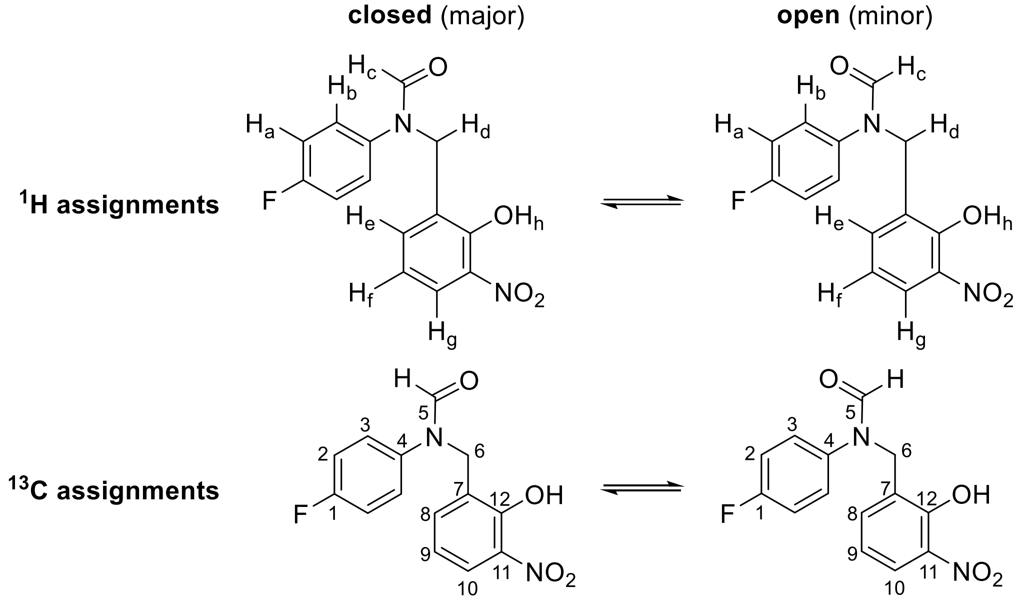

^{1}H NMR (500 MHz, DMSO) δ 10.65 (s, 1H, **H$_h$** closed, **H$_h$** open), 8.59 (s, 1H, **H$_c$** closed), 8.55 (s, 1H, **H$_c$** open), 7.90 (d, *J* = 8.5, 1.7 Hz, 1H, **H$_g$** open), 7.88 (dd, *J* = 8.5, 1.7 Hz, 1H, **H$_g$** closed), 7.46 (dd, *J* = 7.6, 1.6 Hz, 1H, **H$_e$** closed), 7.44 – 7.39 (m, 3H, **H$_b$** closed, **H$_e$** open), 7.33 – 7.28 (m, 2H, **H$_b$** open), 7.25 – 7.19 (m, 2H, **H$_a$** closed), 7.18 – 7.11 (m, 2H, **H$_a$** open), 6.97 (t, *J* = 8.0 Hz, 1H, **H$_f$** closed), 6.93 (t, *J* = 7.9 Hz, 1H, **H$_f$** open), 5.01 (s, 2H, **H$_d$** closed), 4.99 (s, 2H, **H$_d$** open).

Closed conformer (major):

^{13}C NMR (126 MHz, DMSO) δ 162.66 (**C$_5$**), 160.23 (d, *J* = 243.3 Hz, **C$_1$**), 150.41 (**C$_{12}$**), 137.06 (d, *J* = 2.8 Hz, **C$_4$**), 135.65 (**C$_{11}$**), 134.66 (**C$_8$**), 127.54 (**C$_7$**), 125.28 (d, *J* = 8.5 Hz, **C$_3$**), 123.93 (**C$_{10}$**), 119.46 (**C$_9$**), 116.17 (d, *J* = 22.6 Hz, **C$_2$**), 42.76 (**C$_6$**). ^{19}F NMR (471 MHz, DMSO) δ -116.23 (ddd, *J* = 13.3, 8.9, 4.9 Hz).

Open conformer (minor):

^{13}C NMR (126 MHz, DMSO) δ 163.34 (**C$_5$**), 159.95 (d, *J* = 243.3 Hz, **C$_1$**), 151.08 (**C$_{12}$**), 136.38 (**C$_8$**), 135.66 (d, *J* = 2.1 Hz, **C$_4$**), 135.56 (**C$_{11}$**), 127.76 (**C$_7$**), 127.75 (d, *J* = 8.5 Hz, **C$_3$**), 124.64 (**C$_{10}$**), 119.39 (**C$_9$**), 115.53 (d, *J* = 22.5 Hz, **C$_2$**), 47.98, (**C$_6$**). ^{19}F NMR (471 MHz, DMSO) δ -115.65 (tt, *J* = 8.7, 5.1 Hz).

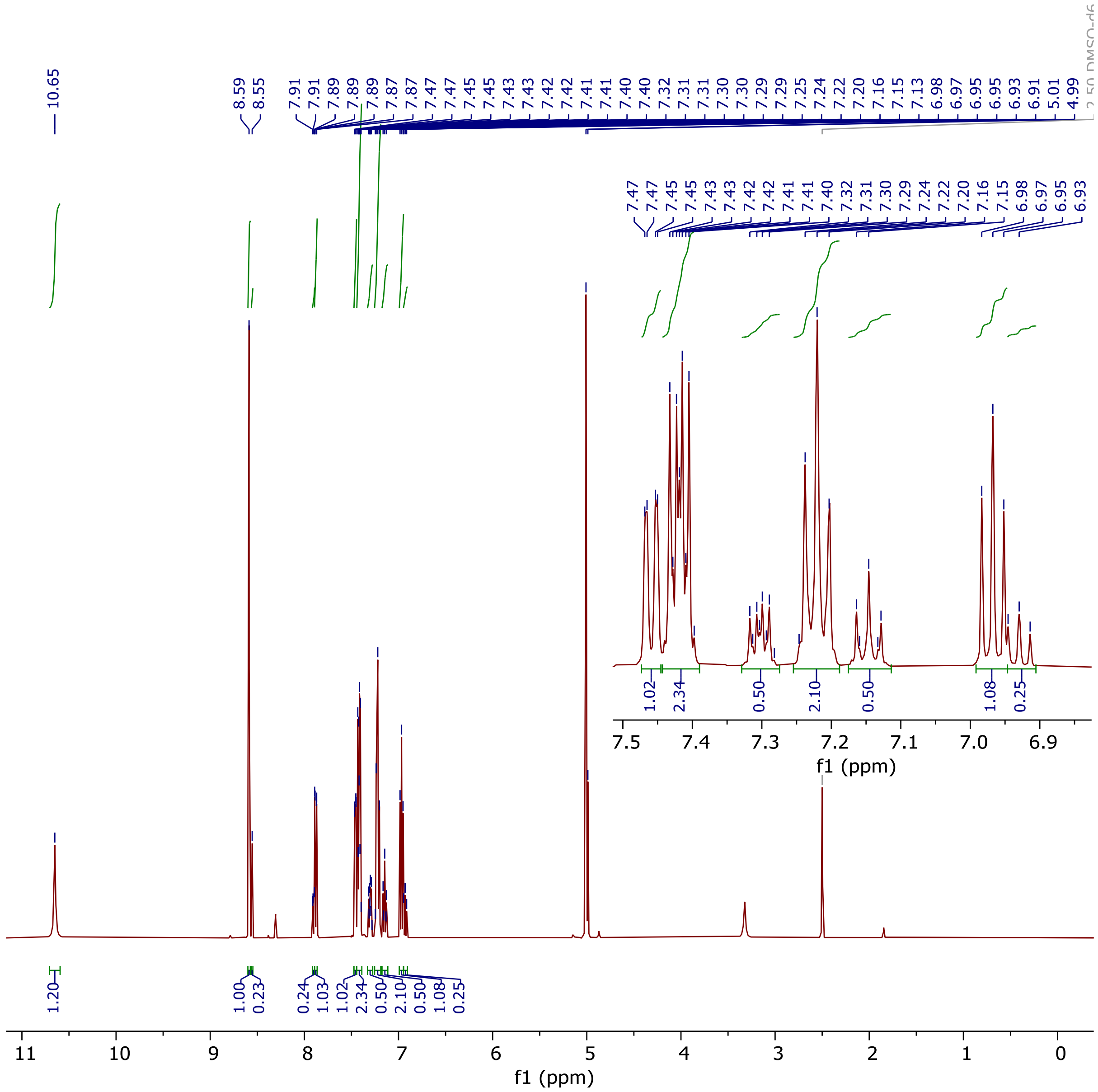

Figure S100: ^{1}H NMR spectrum (DMSO-d_6) of compound **1**-NO_2.

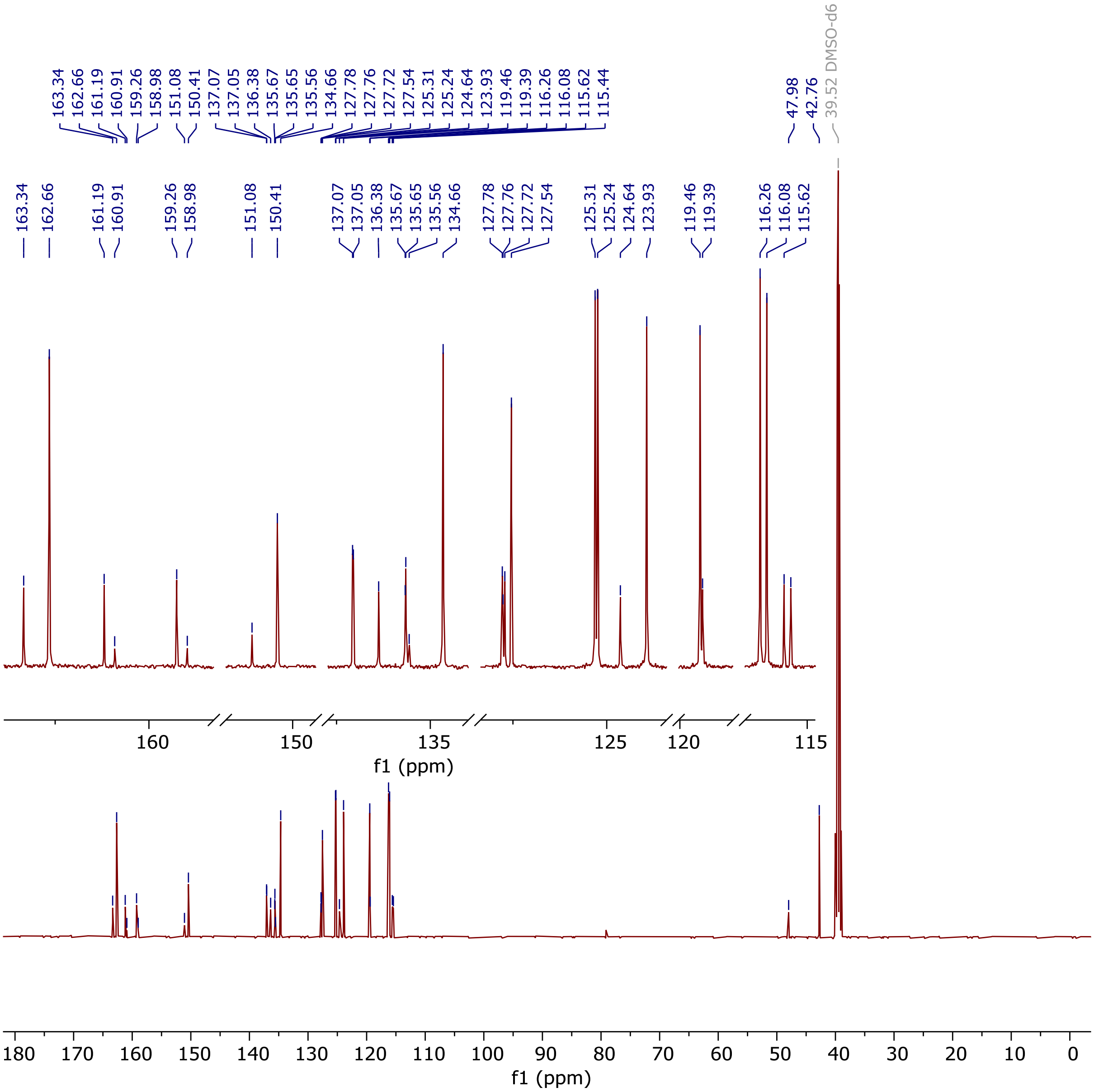

Figure S101: ^{13}C NMR spectrum (DMSO-d_6) of compound **1**-NO_2.

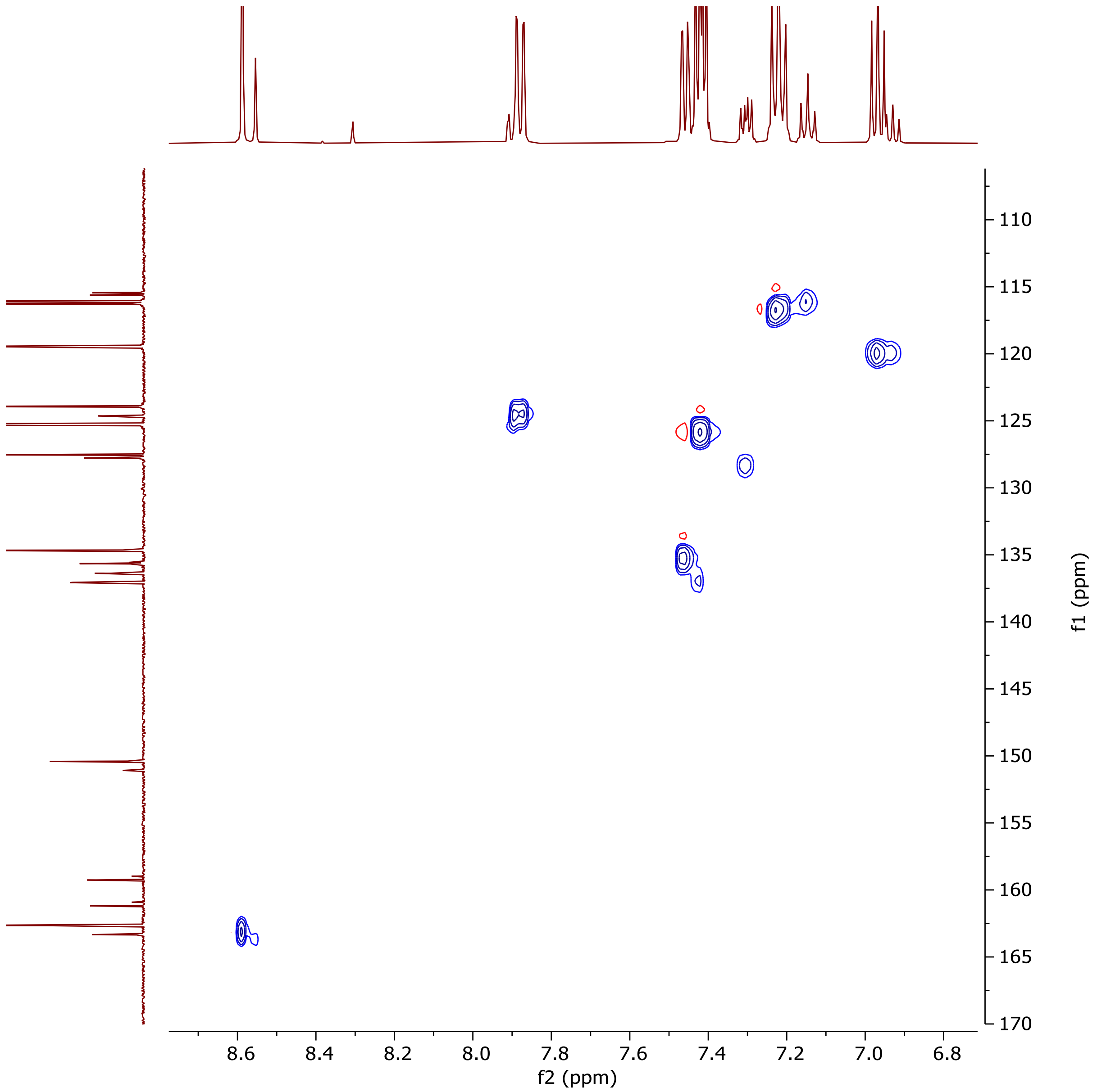

Figure S102: HSQC NMR spectrum (DMSO-d_6) of compound **1**-NO_2.

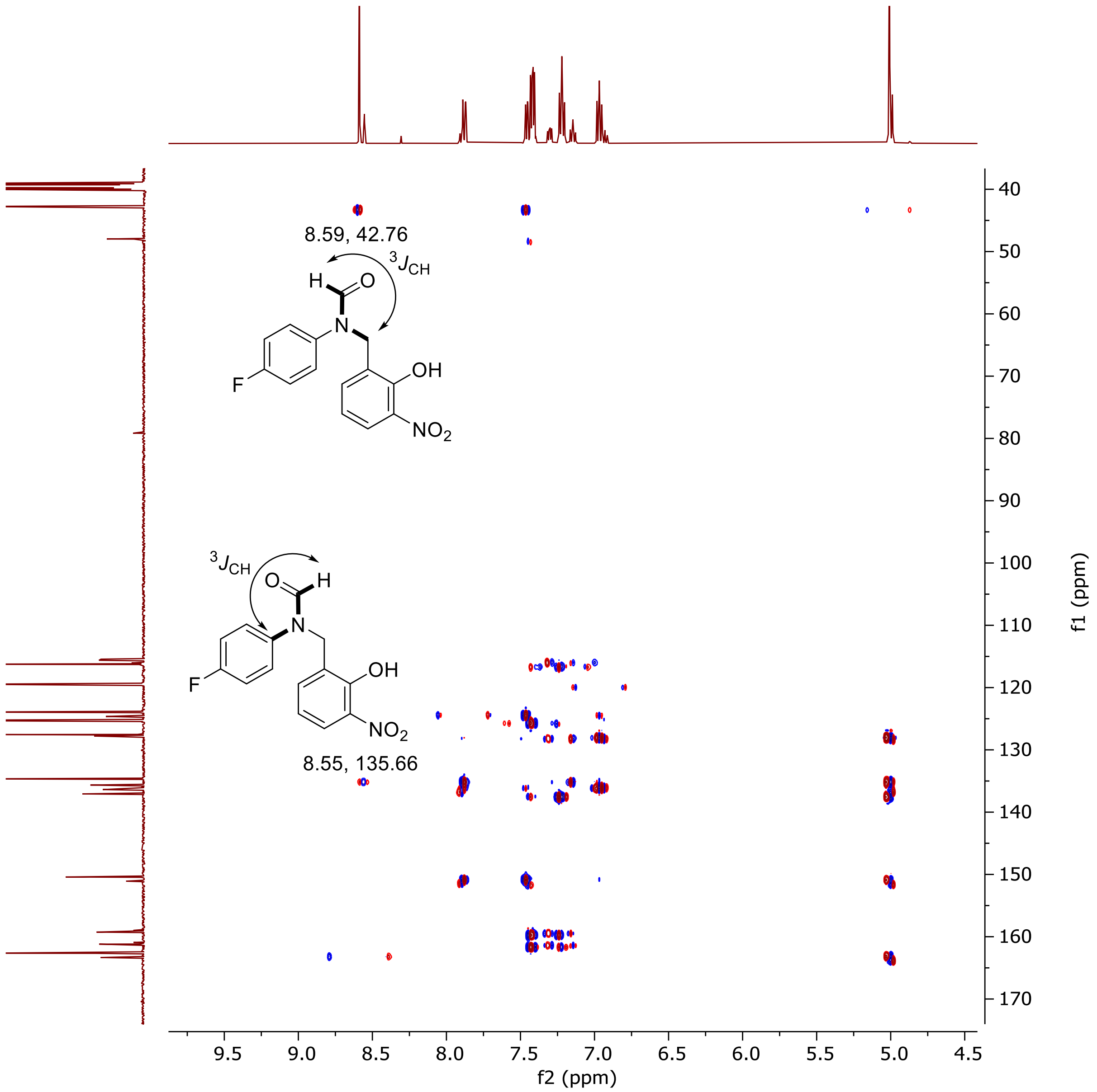

Figure S103: HMBC NMR spectrum (DMSO-d_6) of compound **1**-NO_2.

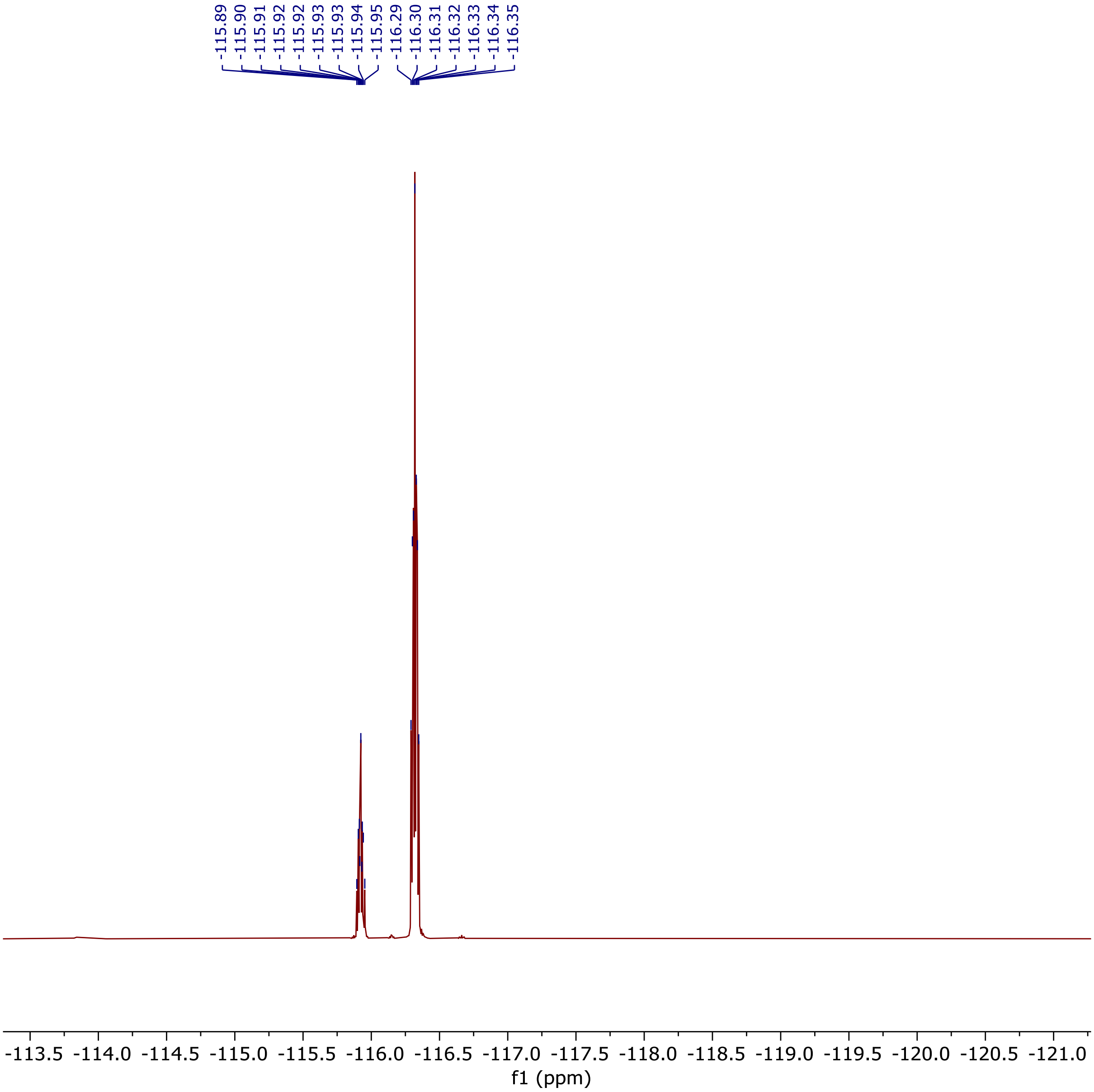

Figure S104: ^{19}F NMR spectrum (DMSO-d_6) of compound **1**-NO_2.

***N*-[(3-chloro-2-hydroxyphenyl)methyl]-*N*-(4-fluorophenyl)formamide (1-Cl)**

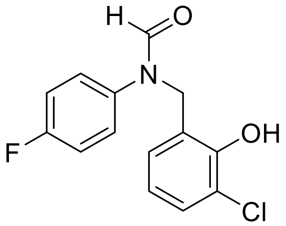

Following general procedure C, compound **3**-Cl (2.0 mmol, 502 mg) was dissolved in 2 mL DCM and cooled down to 0 °C. FAM (3 eq, made from 9 mmol formic acid, 340 µL and 6 mmol acetic anhydride, 560 µL) was added in one portion and the reaction was stirred for 8 h. After removal of volatiles, the residue was purified via flash chromatography (eluting with 100% $CHCl_3$) to give the title compound as a colourless oil, which slowly crystalliZed to give a colourless solid (389 mg, 70%). Mp 83 – 85 °C. HRMS (ESI+): m/z calcd. for $C_{14}H_{12}ClFNO_2$ $[M+H]^+$: 280.0535, found 280.0550.

^{1}H assignments

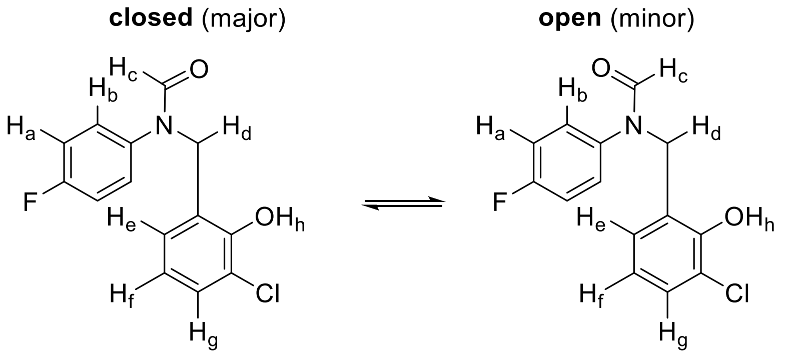

^{13}C assignments

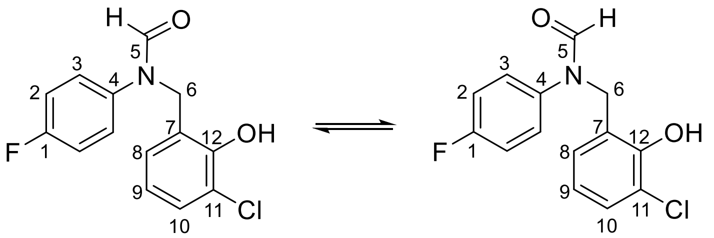

^{1}H NMR (500 MHz, DMSO) δ 9.56 (br s, 2H, $\mathbf{H_h}$ $_{\mathrm{open}}$, $\mathbf{H_h}$ $_{\mathrm{closed}}$), 8.57 (s, 1H, $\mathbf{H_c}$ $_{\mathrm{closed}}$), 8.53 (s, 1H, $\mathbf{H_c}$ $_{\mathrm{open}}$), 7.40 – 7.35 (m, 2H, H_a), 7.30 – 7.26 (m, 2H, $\mathbf{H_b}$ $_{\mathrm{closed}}$), 7.25 – 7.18 (m, 3H, $\mathbf{H_a}$ $_{\mathrm{closed}}$, $\mathbf{H_g}$ $_{\mathrm{closed}}$), 7.17 – 7.11 (m, 2H, $\mathbf{H_a}$ $_{\mathrm{open}}$), 6.97 (dd, J = 7.6, 1.6 Hz, 1H, $\mathbf{H_e}$ $_{\mathrm{closed}}$), 6.95 (d, J = 7.7, 1.5 Hz, 1H, $\mathbf{H_e}$ $_{\mathrm{open}}$), 6.76 (t, J = 7.8 Hz, 1H, $\mathbf{H_f}$ $_{\mathrm{closed}}$), 6.73 (t, J = 7.8 Hz, 1H, $\mathbf{H_f}$ $_{\mathrm{open}}$), 4.96 (s, 2H, $\mathbf{H_d}$ $_{\mathrm{closed}}$), 4.91 (s, 2H, $\mathbf{H_d}$ $_{\mathrm{open}}$).

Major conformer

^{13}C NMR (126 MHz, DMSO) δ 162.73 ($\mathbf{C_5}$), 160.20 (d, J = 243.2 Hz, $\mathbf{C_1}$), 150.38 ($\mathbf{C_{12}}$), 137.13 (d, J = 2.8 Hz, $\mathbf{C_4}$), 128.47 ($\mathbf{C_{10}}$), 126.98 ($\mathbf{C_8}$), 125.80 ($\mathbf{C_7}$), 125.23 (d, J = 8.5 Hz, $\mathbf{C_3}$), 120.90 ($\mathbf{C_{11}}$), 120.29 ($\mathbf{C_9}$), 116.12 (d, J = 22.6 Hz, $\mathbf{C_2}$), 43.27 ($\mathbf{C_6}$). ^{19}F NMR (471 MHz, DMSO) δ -116.32 (ddd, J = 13.4, 8.7, 4.8 Hz).

Minor conformer

^{13}C NMR (126 MHz, DMSO) δ 163.30 ($\mathbf{C_5}$), 159.82 (d, J = 243.3 Hz, $\mathbf{C_1}$), 151.01 ($\mathbf{C_{12}}$), 134.93 (d, J = 2.8 Hz, $\mathbf{C_4}$), 129.02 ($\mathbf{C_{10}}$), 128.31 ($\mathbf{C_{10}}$), 127.49 (d, J = 8.5 Hz, $\mathbf{C_3}$), 126.17 ($\mathbf{C_7}$), 120.85 ($\mathbf{C_{11}}$), 120.17 ($\mathbf{C_9}$), 115.42 (d, J = 22.5 Hz, $\mathbf{C_2}$), 48.63 ($\mathbf{C_6}$). ^{19}F NMR (471 MHz, DMSO) δ -115.92 (tt, J = 8.6, 5.1 Hz).

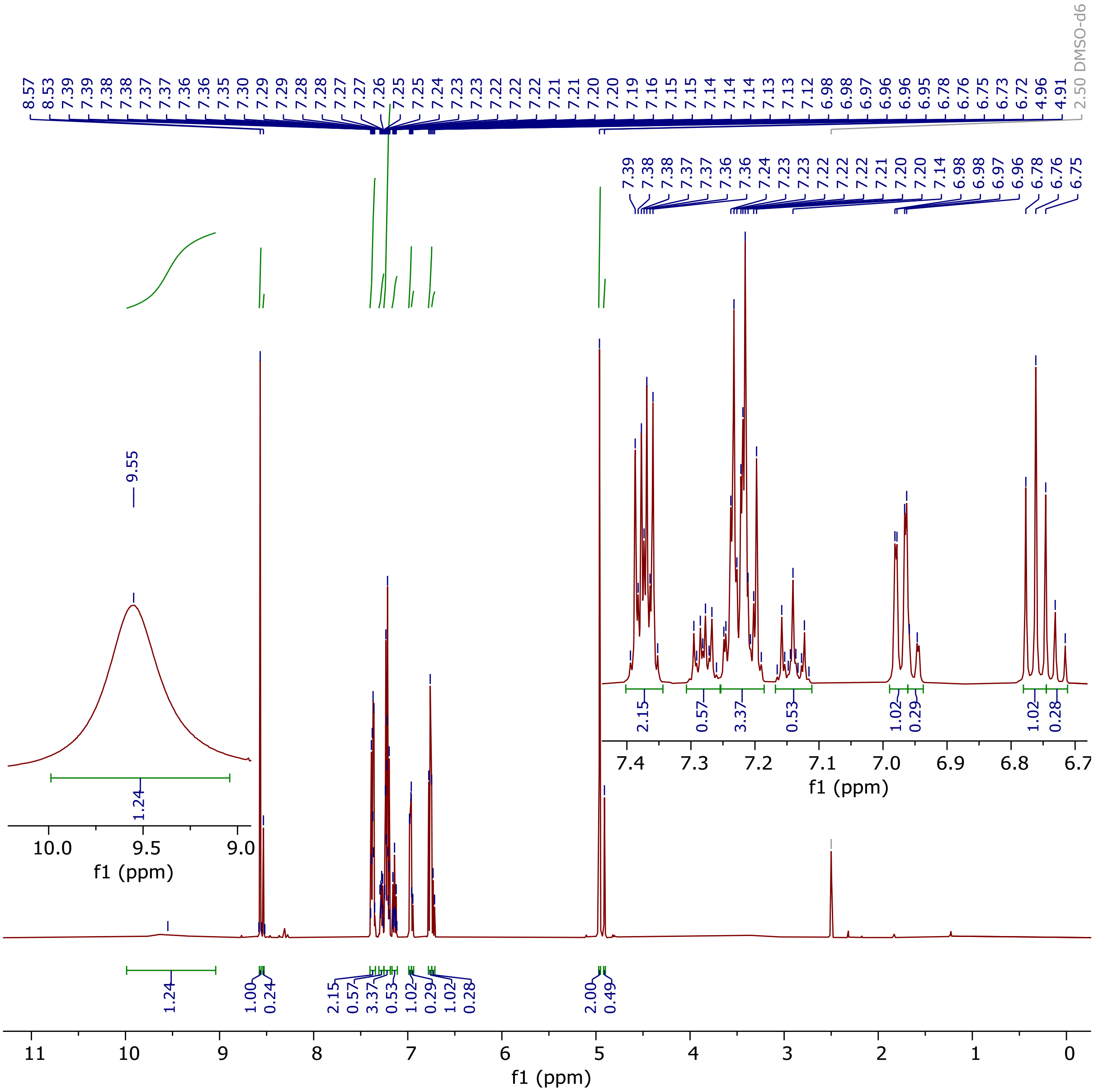

Figure S105: ^1H NMR spectrum (DMSO-d_6) of compound **1**-Cl.

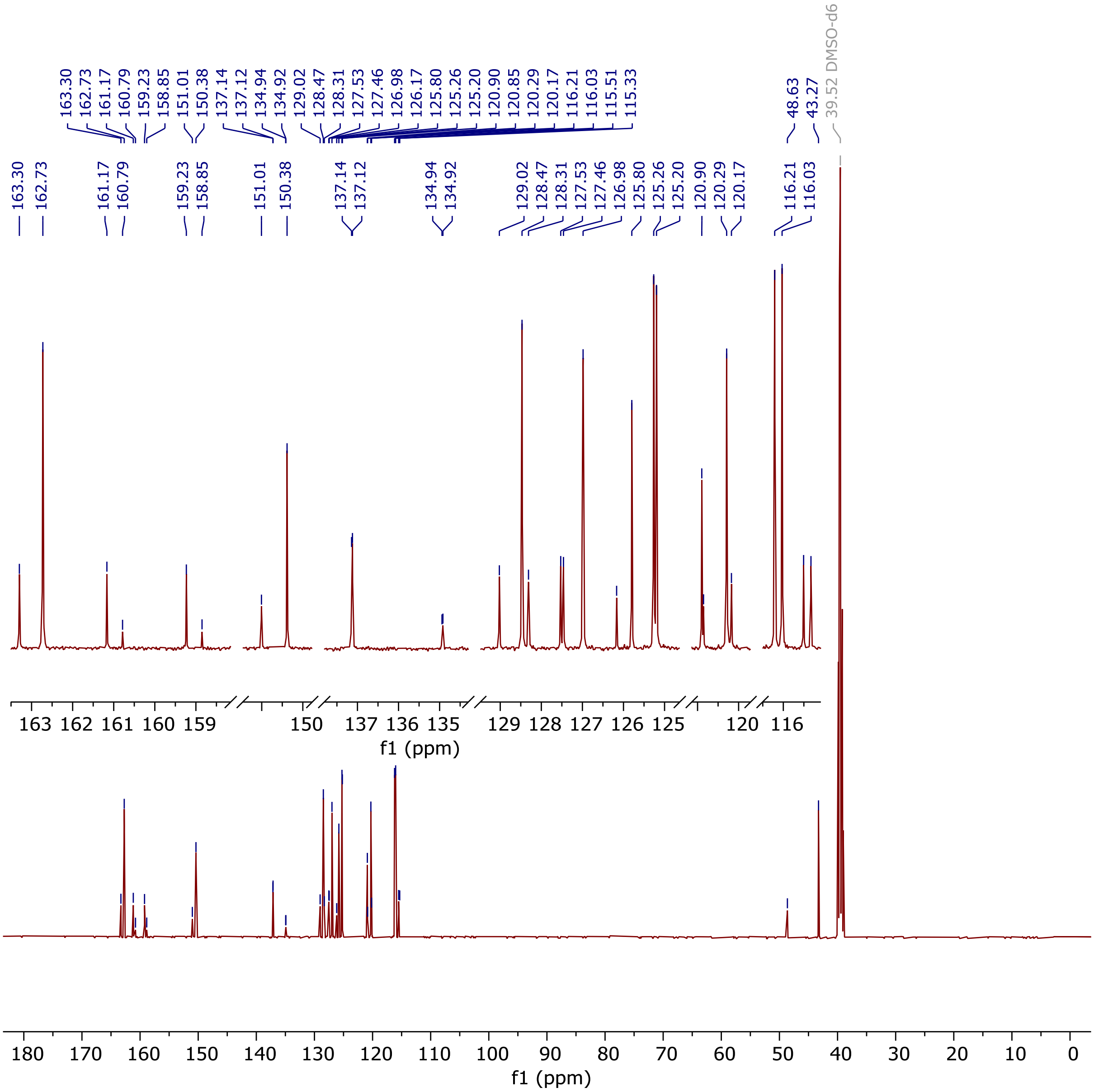

Figure S106: ^{13}C NMR spectrum (DMSO-d_6) of compound **1**-Cl.

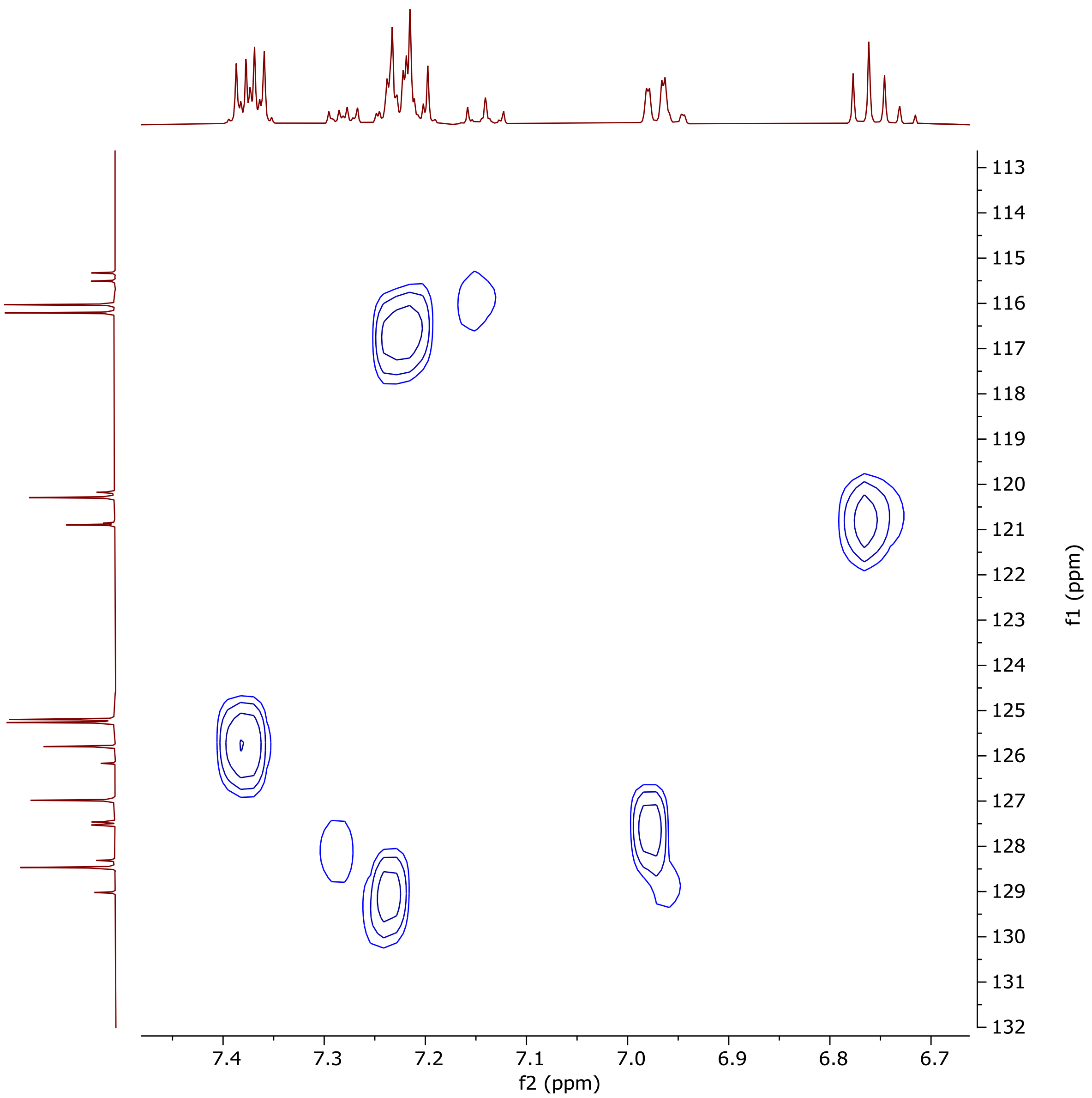

Figure S107: HSQC NMR spectrum (DMSO-d_6) of compound **1**-Cl.

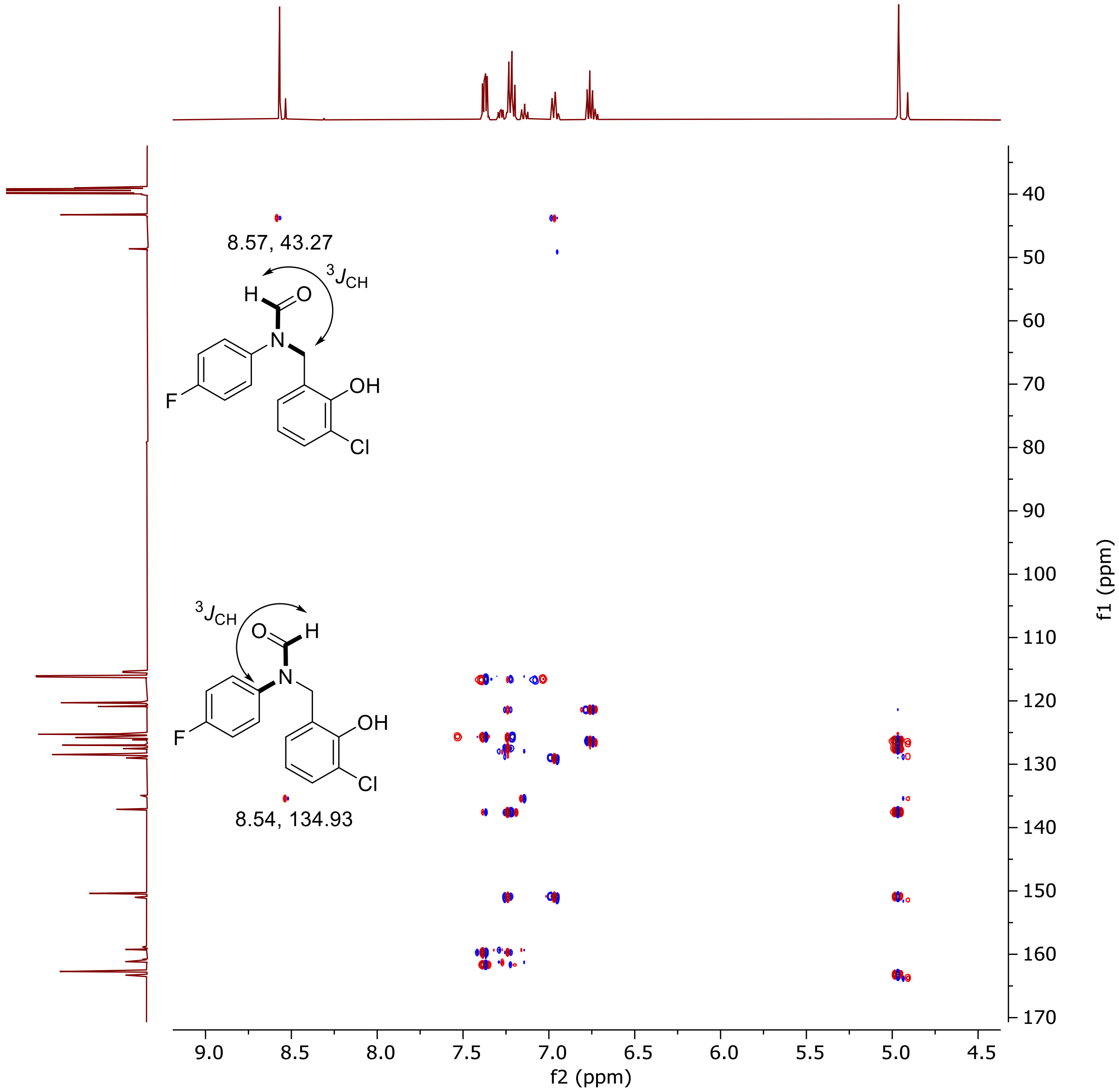

Figure S108: HMBC NMR spectrum (DMSO-d_6) of compound **1**-Cl.

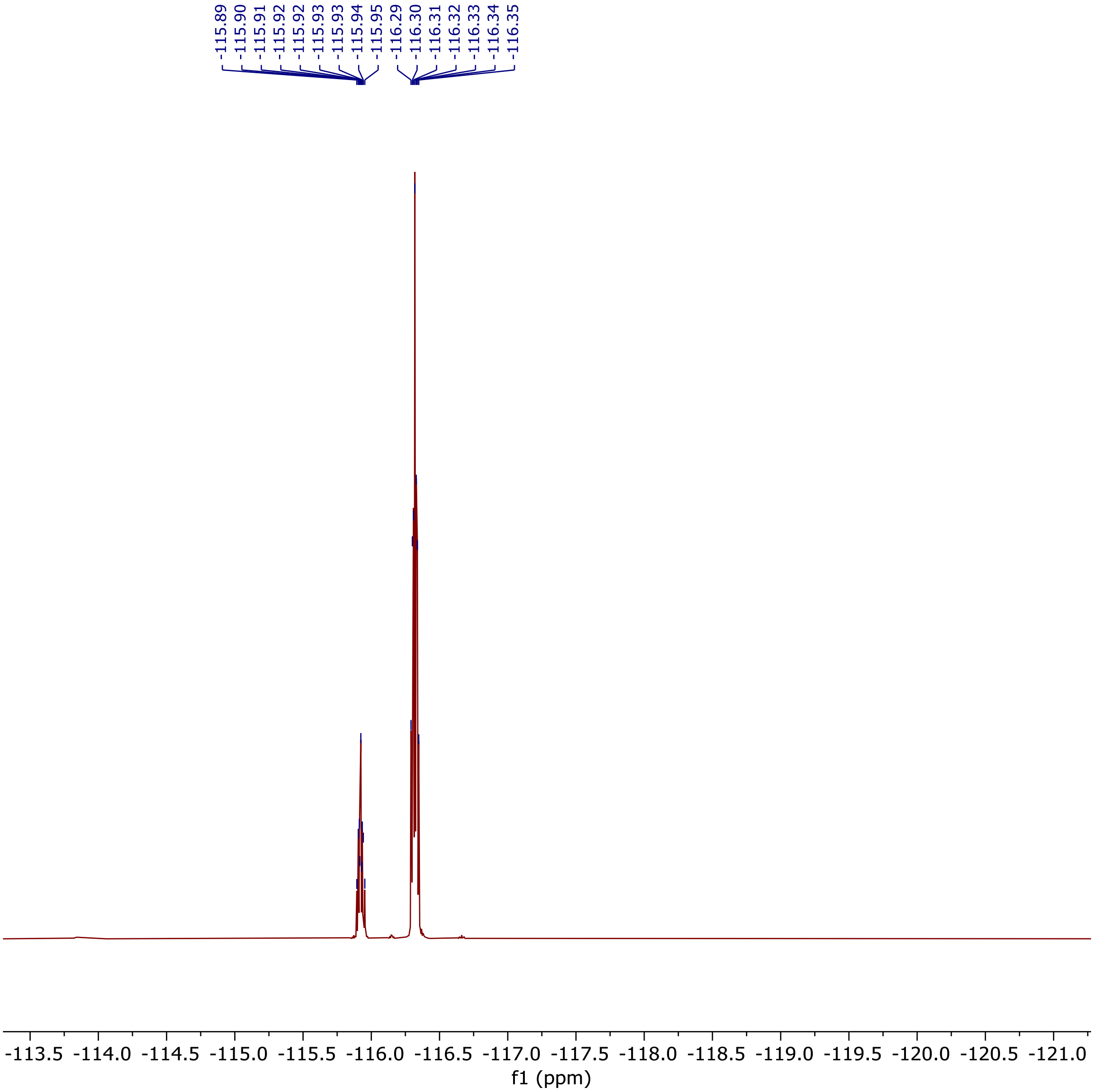

Figure S109: ^{19}F NMR spectrum (DMSO-d_6) of compound **1**-Cl.

***N*-[(3-*tert*-butyl-2-hydroxyphenyl)methyl]-*N*-(4-fluorophenyl)formamide (1-^{t}Bu)**

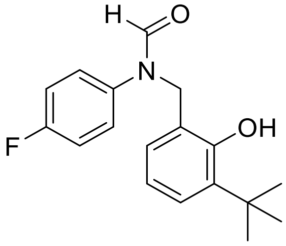

Following general procedure C, **3-^{t}Bu** (819 mg, 3 mmol) was dissolved in 2 mL DCM and cooled to 0 °C. FAM (3 eq, made from 5.4 mmol formic acid, 204 µL and 3.6 eq acetic anhydride, 340 µL) was added in one portion and reaction was stirred at RT for 8 h. After removal of volatiles, the residue was recrystallised from DCM/Hex to give the title compound as a colourless solid (848 mg, 94%). Mp 116 – 118 °C. HRMS (ESI+): m/z calcd. for $C_{18}H_{21}FNO_2$ [M+H]$^+$: 302.1551, 302.1551.

^{1}H assignments

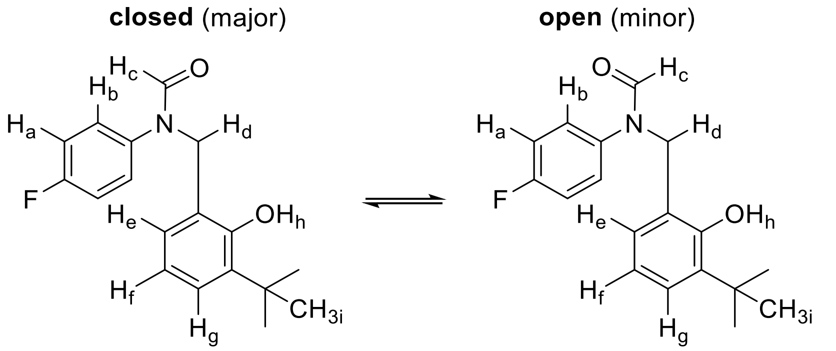

^{13}C assignments

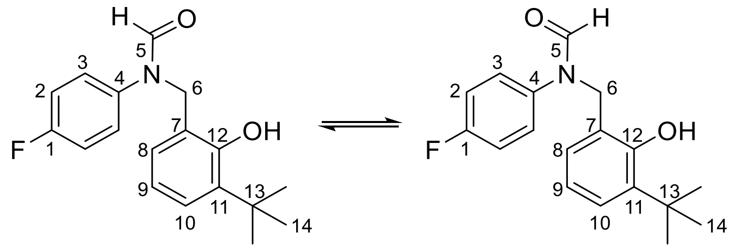

^{1}H NMR (500 MHz, DMSO) δ 8.73 (s, 1H, **H$_h$** $_{closed}$), 8.53 (s, 1H, **H$_c$** $_{closed}$), 8.50 (s, 1H, **H$_c$** $_{open}$), 8.34 (s, 1H, **H$_h$** $_{open}$), 7.41 – 7.33 (m, 2H, **H$_b$** $_{closed}$), 7.30 – 7.20 (m, 4H, **H$_a$** $_{closed,}$ **H$_b$** $_{open}$), 7.16 – 7.12 (m, 2H, **H$_a$** $_{open}$), 7.10 (d, J = 7.6 Hz, 1H, **H$_g$** $_{closed}$), 7.08 (d, J = 7.5 Hz, 1H, **H$_g$** $_{open}$), 6.84 (d, J = 7.5 Hz, 1H, **H$_e$** $_{open}$), 6.77 (dd, J = 8.0, 1.3 Hz, 1H, **H$_e$** $_{closed}$), 6.72 (t, J = 7.7 Hz, 1H, **H$_f$** $_{open}$), 6.69 (t, J = 7.6 Hz, 1H, **H$_f$** $_{closed}$), 4.95 (s, 2H, **H$_d$** $_{open}$), 4.93 (s, 2H, **H$_d$** $_{closed}$), 1.35 (s, 9H, **H$_i$** $_{closed}$), 1.34 (s, 9H, **H$_i$** $_{open}$).

Major conformer

^{13}C NMR (126 MHz, DMSO) δ 163.47 (**C$_5$**), 160.41 (d, J = 243.5 Hz, **C$_1$**), 153.22 (**C$_{12}$**), 137.83 (**C$_{11}$**), 137.03 (d, J = 2.7 Hz, **C$_4$**), 126.52 (**C$_8$**), 125.79 (**C$_{10}$**), 125.70 (d, J = 8.6 Hz, **C$_3$**), 124.15 (**C$_7$**), 119.52 (**C$_9$**), 116.19 (d, J = 22.7 Hz, **C$_2$**), 44.60 (**C$_6$**), 34.46 (**C$_{13}$**), 29.63 (**C$_{14}$**). ^{19}F NMR (471 MHz, DMSO) δ -115.92 (td, J = 8.6, 4.8 Hz).

Minor conformer

^{13}C NMR (126 MHz, DMSO) δ 163.19(**C$_5$**), 159.72 (d, J = 243.2 Hz, **C$_1$**), 153.09 (**C$_{12}$**), 138.59 (**C$_{11}$**), 135.27 (d, J = 2.9 Hz, **C$_4$**), 127.15 (d, J = 8.5 Hz, **C$_3$**), 126.34 (**C$_8$**), 125.85 (**C$_{10}$**), 124.15 (**C$_7$**), 120.00 (**C$_9$**), 115.29 (d, J = 22.5 Hz, **C$_2$**), 48.56 (**C$_6$**), 34.42 (**C$_{13}$**), 29.70 (**C$_{14}$**). ^{19}F NMR (471 MHz, DMSO) δ -116.16 (td, J = 8.7, 5.1 Hz).

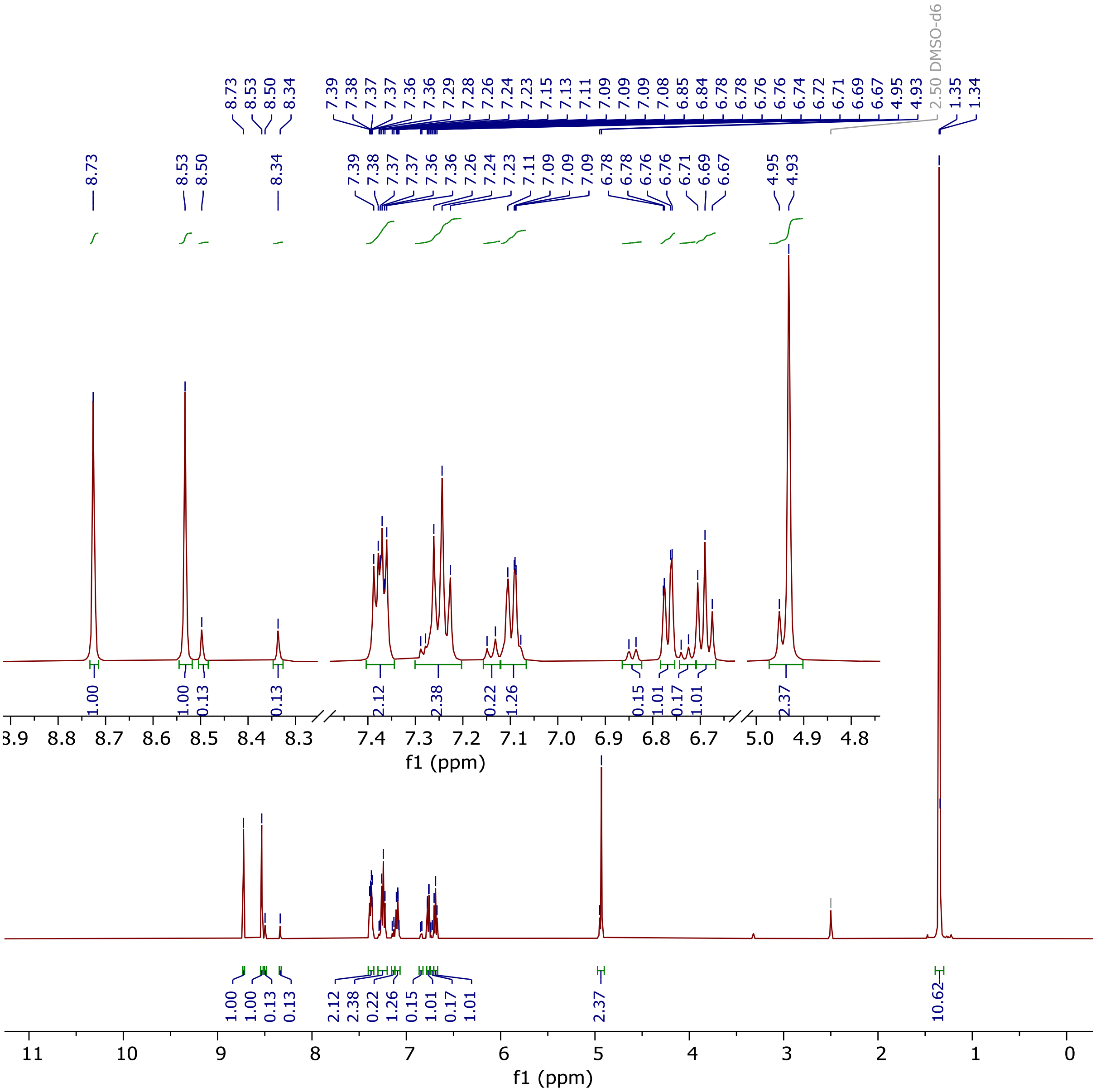

Figure S110: ^{1}H NMR spectrum (DMSO-d_6) of compound **1**-^{t}Bu.

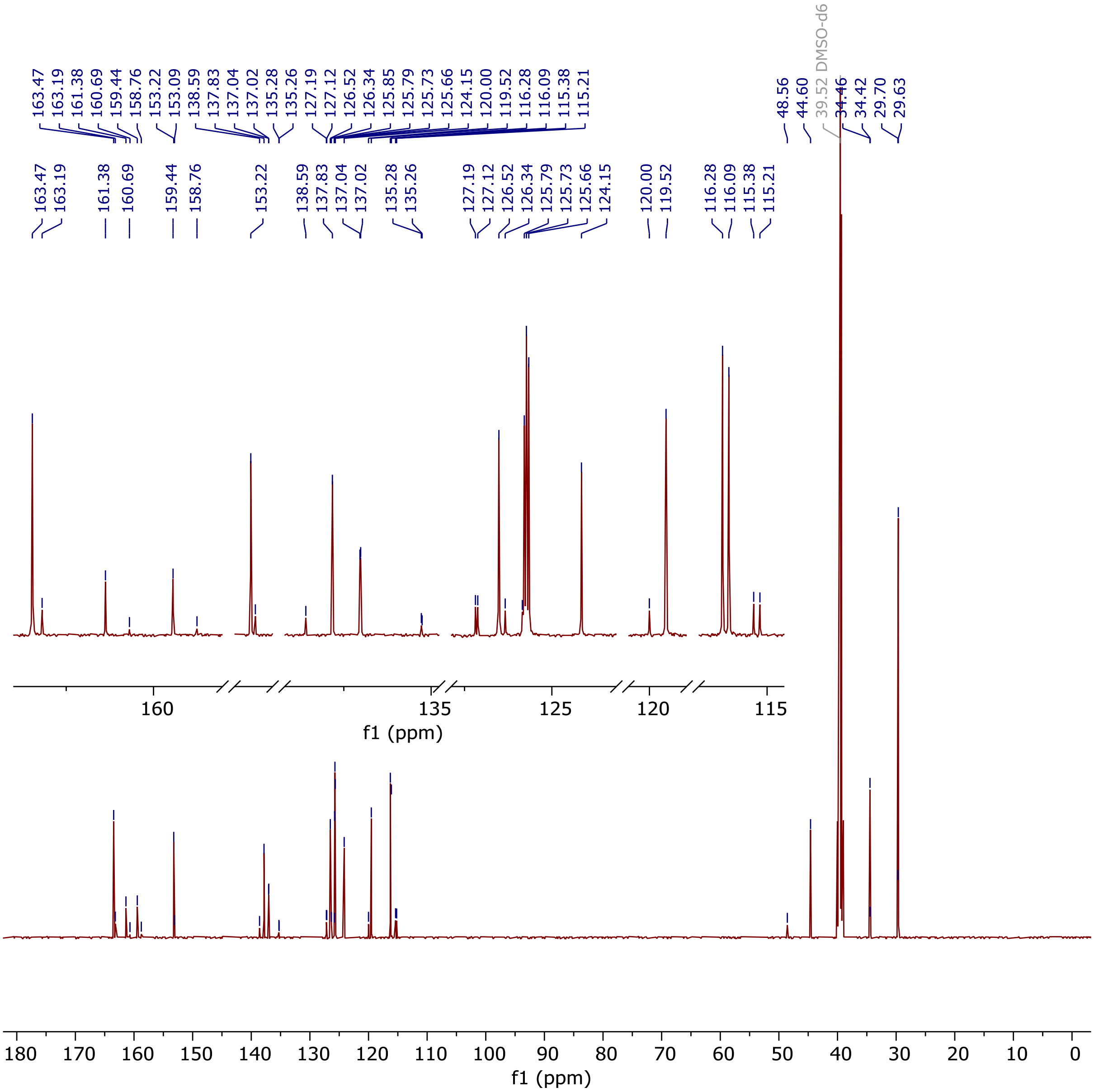

Figure S111: ^{13}C NMR spectrum (DMSO-d_6) of compound **1**-^{t}Bu.

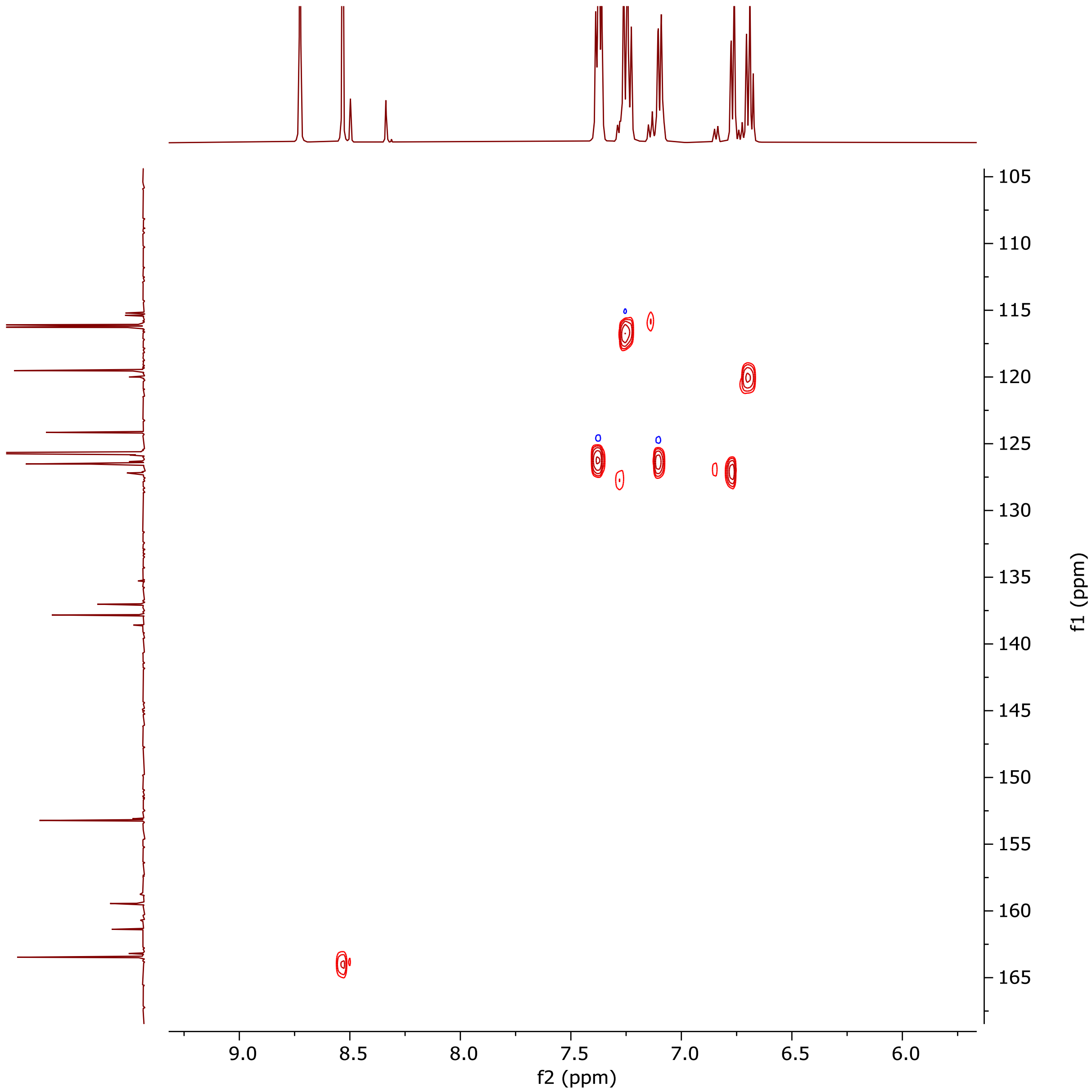

Figure S112: HSQC NMR spectrum (DMSO-d_6) of compound **1**-^{t}Bu.

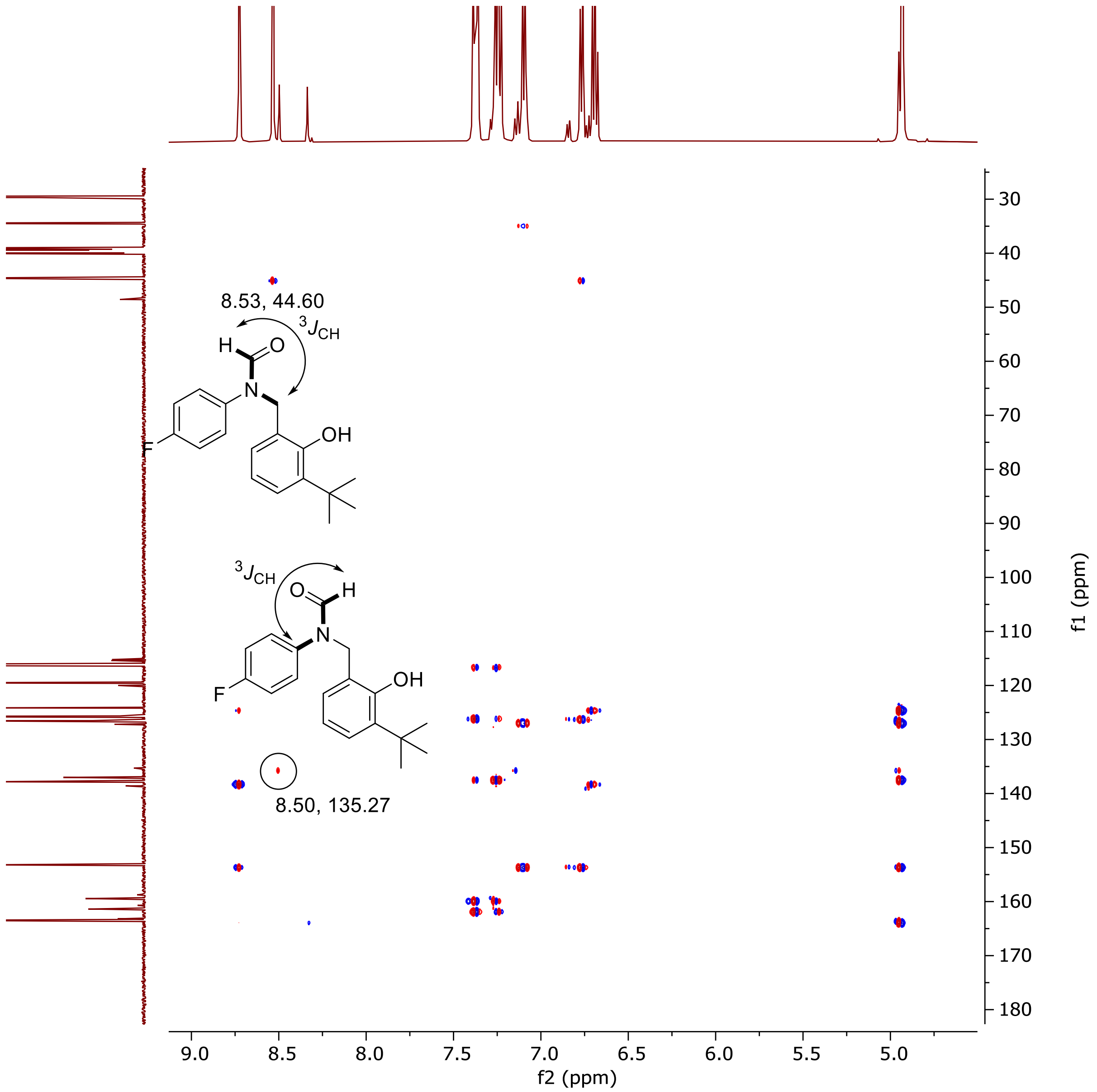

Figure S113: HMBC NMR spectrum (DMSO-d_6) of compound **1**-^{t}Bu.

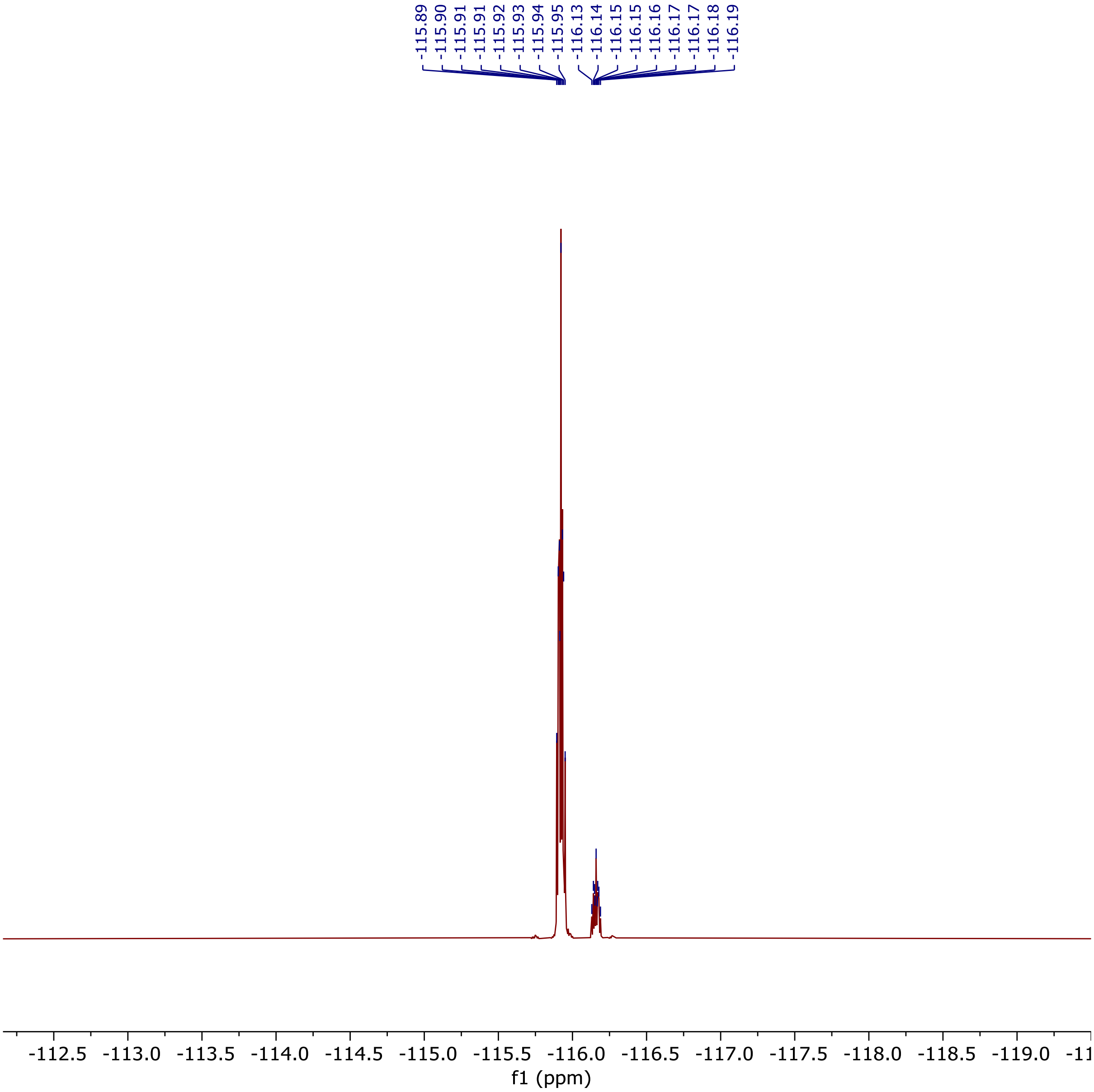

Figure S114: ^{19}F NMR spectrum (DMSO-d_6) of compound **1**-^{t}Bu.

***N*-(4-fluorophenyl)-*N*-[(2-hydroxyphenyl)methyl]formamide 1-H**

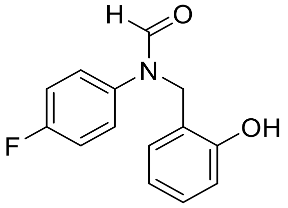

Following general procedure C, compound **3-H** (3.0 mmol, 651 mg) was dissolved in 2 mL DCM and cooled down to 0 °C. FAM (3 eq, made from 13.5 mmol formic acid, 510 µL and 9 mmol acetic anhydride, 850 µL) was added in one portion and the reaction was stirred for 8 h. After removal of volatiles, the residue was purified via flash chromatography (eluting with gradient of DCM:MeOH 100:0 to DCM:MeOH 100:1) to afford the title compound as a colourless oil which slowly crystallised to give a colourless solid (557 mg, 76%). Mp 89 – 91 °C. HRMS (ESI+): m/z calcd. for $C_{14}H_{12}FNO_2Na$ $[M+Na]^+$: 268.0744, found 268.0710.

closed (major) **open** (minor)

^{1}H assignments

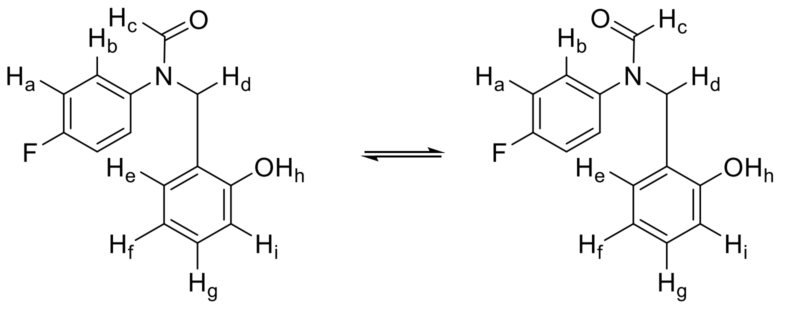

^{13}C assignments

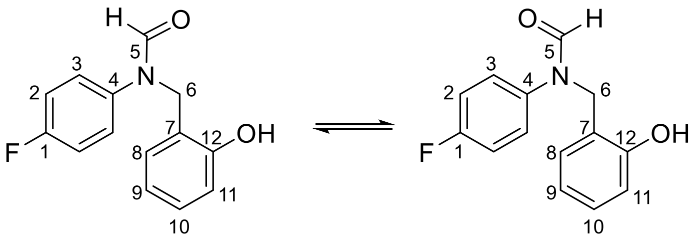

^{1}H NMR (500 MHz, DMSO) δ 9.70 (s, 1H, **H**$_{\mathbf{h}\ \text{open}}$), 9.55 (s, 1H, **H**$_{\mathbf{h}\ \text{closed}}$), 8.57 (s, 1H, **H**$_{\mathbf{c}\ \text{closed}}$), 8.51 (s, 1H, **H**$_{\mathbf{c}\ \text{open}}$), 7.39 – 7.31 (m, 2H, **H**$_{\mathbf{b}\ \text{closed}}$), 7.29 – 7.25 (m, 2H, **H**$_{\mathbf{b}\ \text{open}}$), 7.23 – 7.17 (m, 2H, **H**$_{\mathbf{a}\ \text{closed}}$), 7.16 – 7.10 (m, 2H, **H**$_{\mathbf{a}\ \text{open}}$), 7.05 (dd, J = 7.7, 1.7 Hz, 1H, **H**$_{\mathbf{g}\ \text{open}}$), 7.03 (td, J = 7.9, 1.6 Hz, 1H, **H**$_{\mathbf{g}\ \text{closed}}$), 6.99 (dd, J = 7.6, 1.7 Hz, 1H, **H**$_{\mathbf{e}\ \text{closed}}$), 6.95 (dd, J = 7.6, 1.7 Hz, 1H, **H**$_{\mathbf{e}\ \text{open}}$), 6.78 (dd, J = 8.0, 1.2 Hz, 1H, **H**$_{\mathbf{i}\ \text{open}}$), 6.77 (dd, J = 8.0, 1.2 Hz, 2H, **H**$_{\mathbf{i}\ \text{closed}}$), 6.69 (td, J = 7.5, 1.1 Hz, 1H, **H**$_{\mathbf{f}\ \text{closed}}$), 6.66 (td, J = 7.4, 1.1 Hz, **H**$_{\mathbf{f}\ \text{open}}$), 4.90 (s, 2H, **H**$_{\mathbf{d}\ \text{closed}}$), 4.82 (s, 2H, **H**$_{\mathbf{d}\ \text{open}}$).

Closed conformer (major):

^{13}C NMR (126 MHz, DMSO) δ 162.43 (**C$_5$**), 160.04 (d, J = 242.8 Hz, **C$_1$**), 154.84 (**C$_{12}$**), 137.41 (d, J = 2.7 Hz, **C$_4$**), 128.02 (**C$_8$**), 128.01 (**C$_9$**), 125.05 (d, J = 8.5 Hz, **C$_3$**), 122.35 (**C$_7$**), 118.84 (**C$_9$**), 115.98 (d, J = 22.6 Hz, **C$_2$**), 114.97 (**C$_{11}$**), 42.49 (**C$_6$**). ^{19}F NMR (471 MHz, DMSO) δ -116.71 (tt, J = 9.1, 5.0 Hz).

Open conformer (minor):

^{13}C NMR (126 MHz, DMSO) δ 163.29 (**C$_5$**), 159.73 (d, J = 243.1 Hz, **C$_1$**), 155.53 (**C$_{12}$**), 135.17 (d, J = 3.0 Hz, **C$_4$**), 129.53 (**C$_8$**), 128.79 (**C$_9$**), 127.46 (d, J = 8.5 Hz, **C$_3$**), 122.62 (**C$_7$**), 118.74 (**C$_9$**), 115.30 (d, J = 22.4 Hz, **C$_2$**), 115.13 (**C$_{11}$**), 48.45 (**C$_6$**). ^{19}F NMR (471 MHz, DMSO) δ -116.17 (tt, J = 9.1, 5.2 Hz).

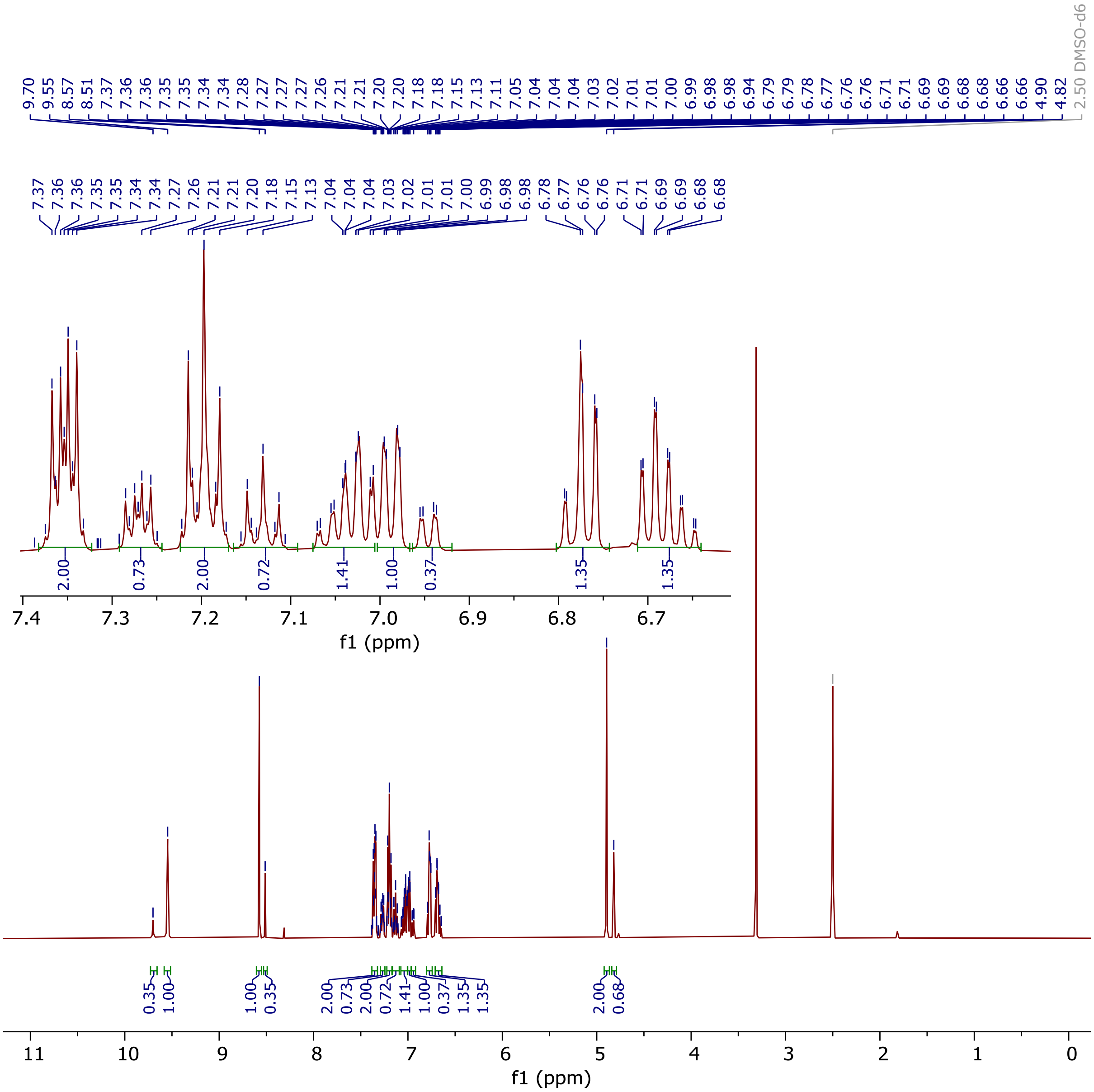

Figure S115: ^{1}H NMR spectrum (DMSO-d_6) of compound **1**-H.

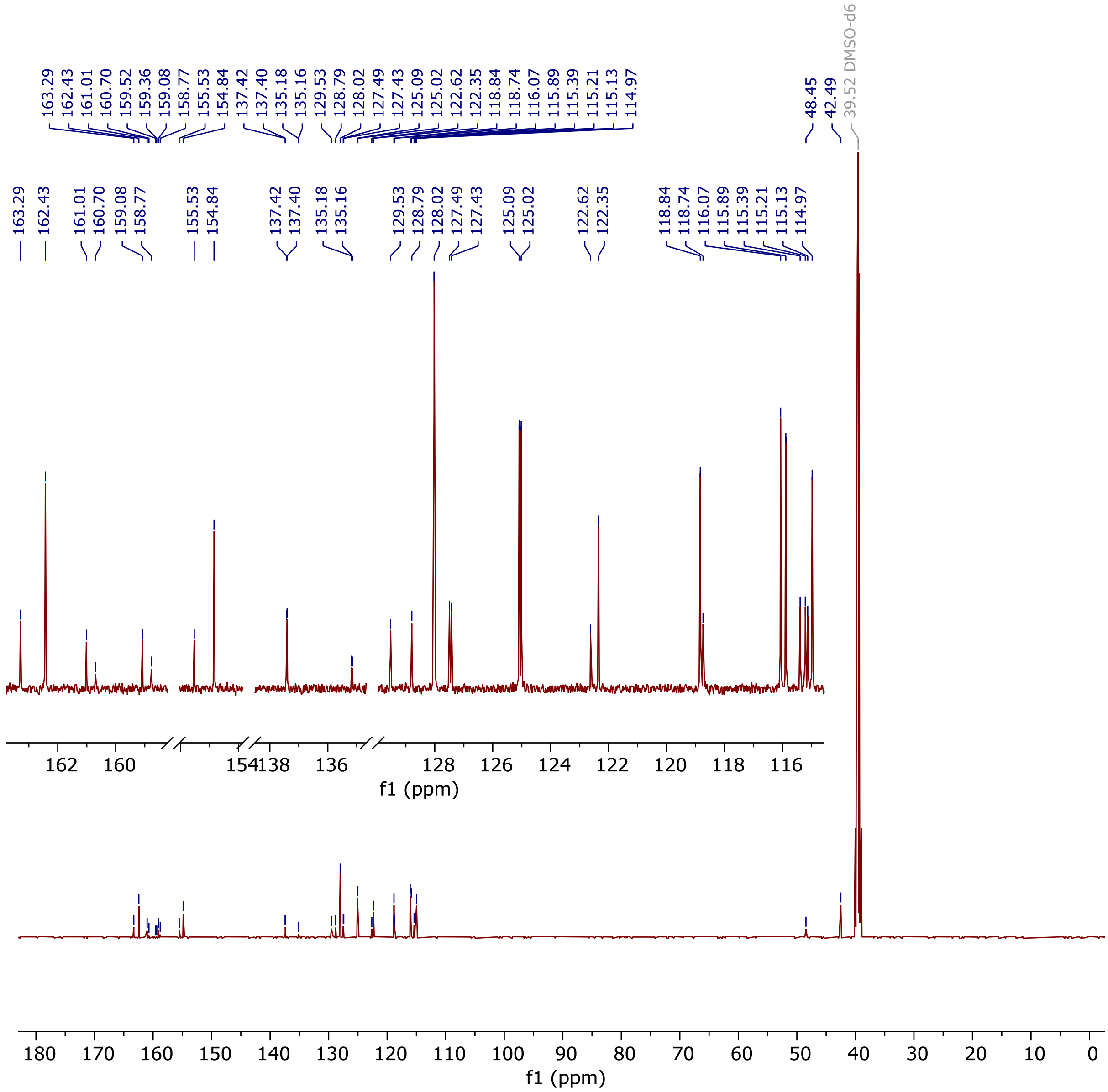

Figure S116: ^{13}C NMR spectrum (DMSO-d_6) of compound **1**-H.

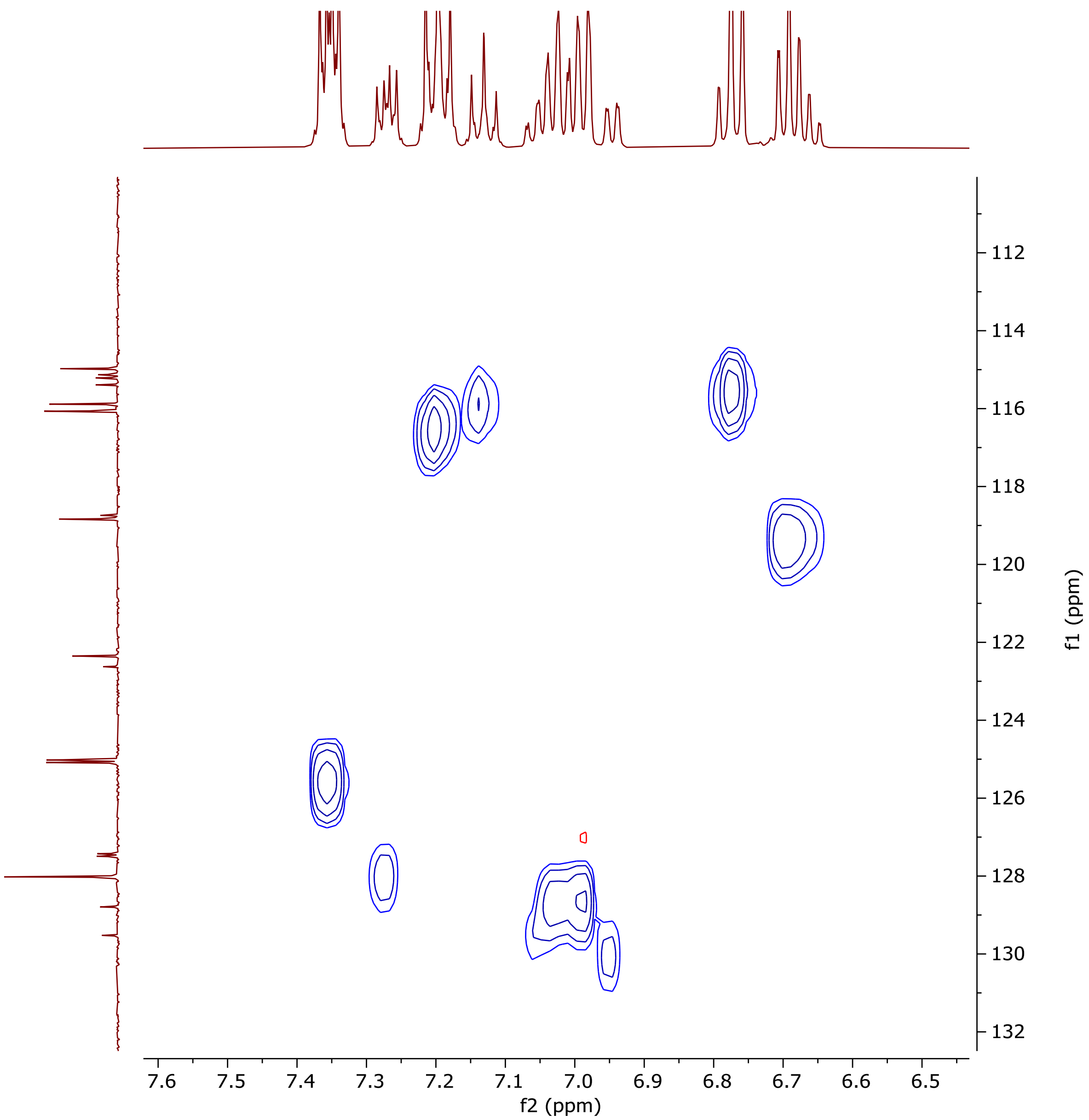

Figure S117: HSQC NMR spectrum (DMSO-d_6) of compound **1**-H.

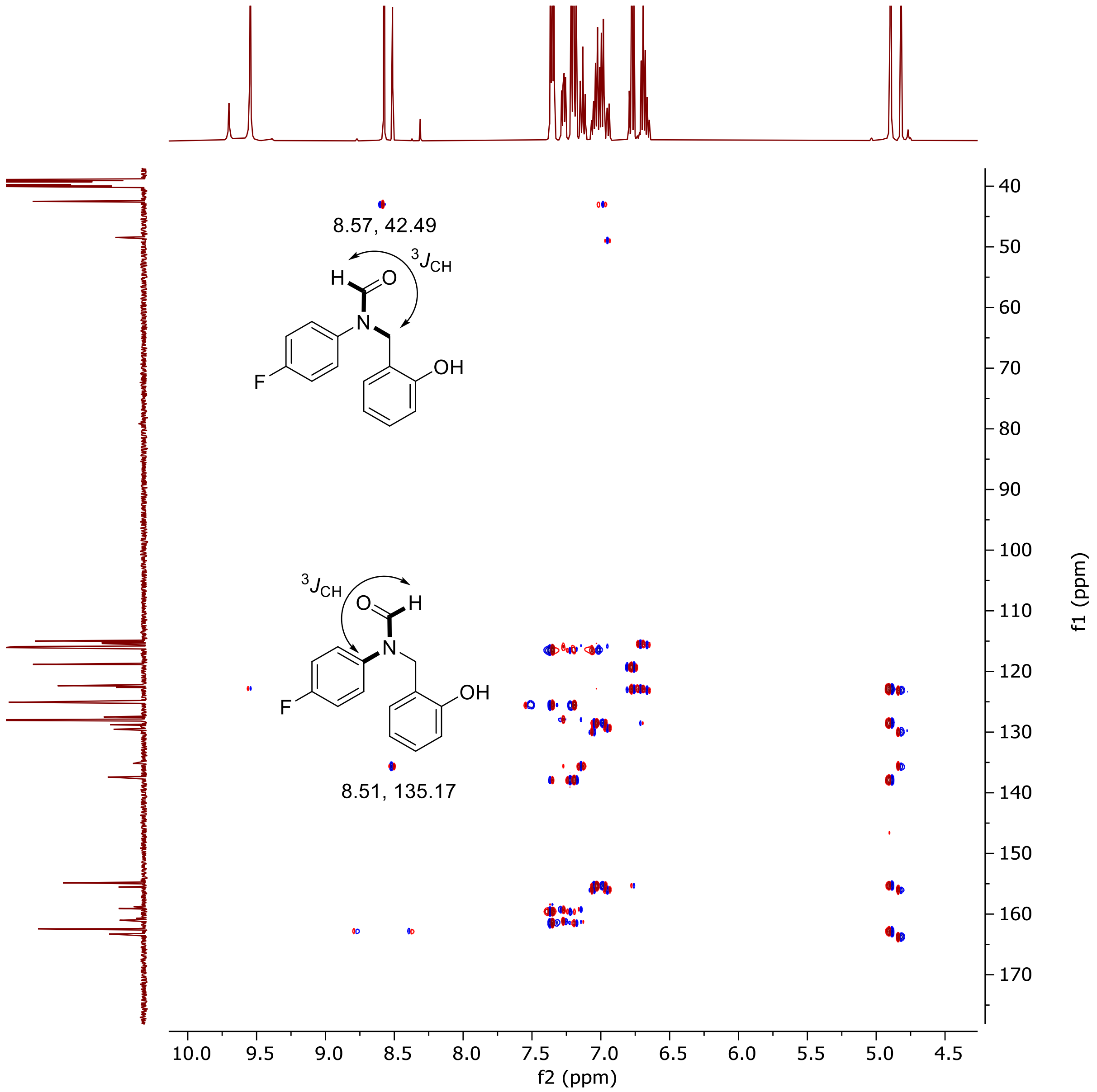

Figure S118: HMBC NMR spectrum (DMSO-d_6) of compound **1**-H.

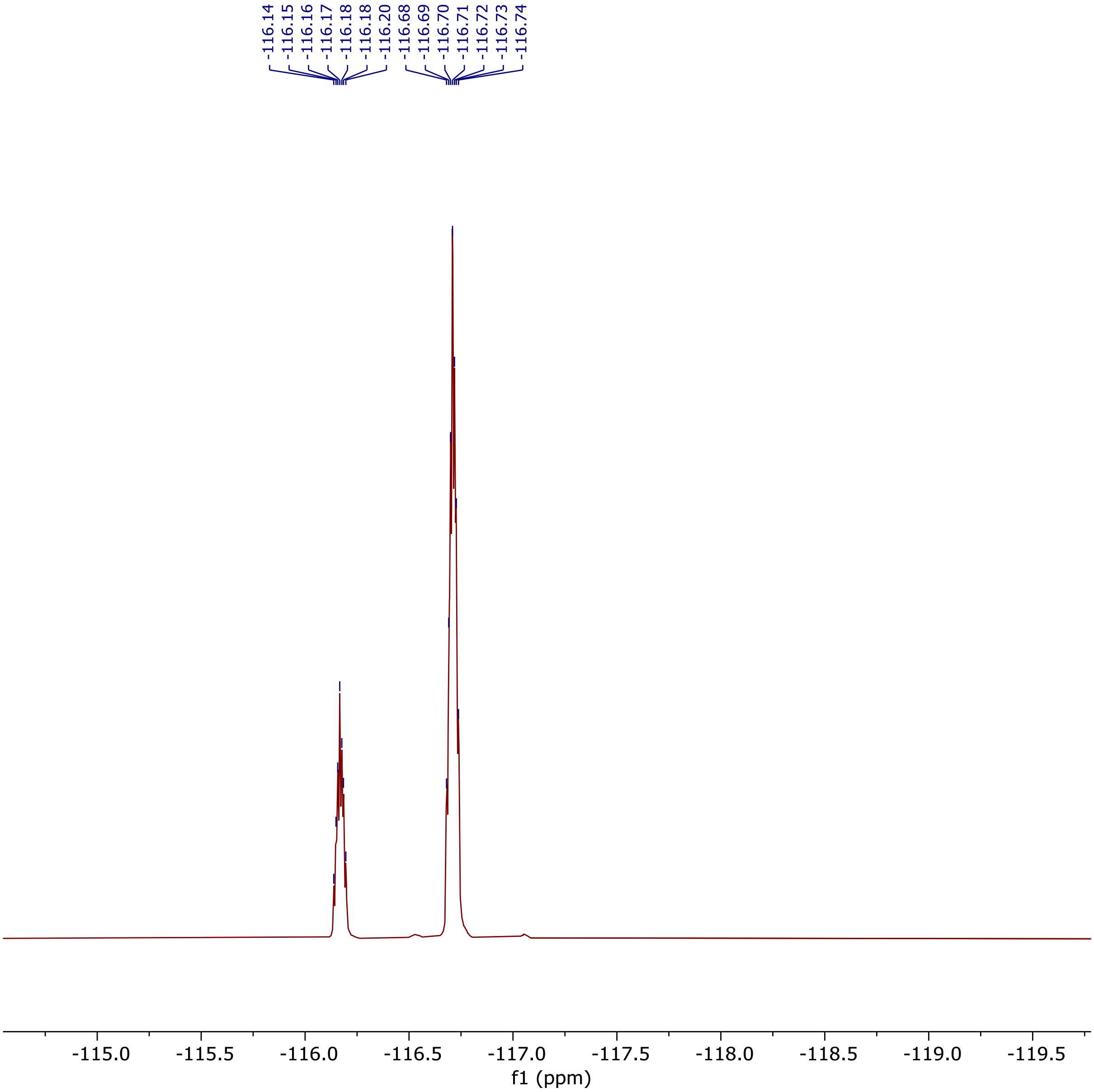

Figure S119: ^{19}F NMR spectrum (DMSO-d_6) of compound **1**-H.

***N*-[(3-fluoro-2-hydroxyphenyl)methyl]-*N*-(4-fluorophenyl)formamide (1-F)**

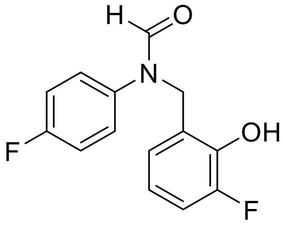

Following general procedure C, compound **3**-F (3.0 mmol, 705 mg) was dissolved in 2 mL DCM and cooled down to 0 °C. FAM (3 eq, made from 13.5 mmol formic acid, 510 µL and 9 mmol acetic anhydride, 850 µL) was added in one portion and the reaction was stirred for 8 h. After removal of volatiles, the residue was purified via flash chromatography (eluting with 100% $CHCl_3$) to give the title compound as a colourless solid (640 mg, 76%). Mp 119 –121 °C. HRMS (ESI+): m/z calcd. for $C_{14}H_{12}F_2NO_2$ $[M+H]^+$: 264.0831, found 264.0826.

closed (major) **open** (minor)

^{1}H assignments

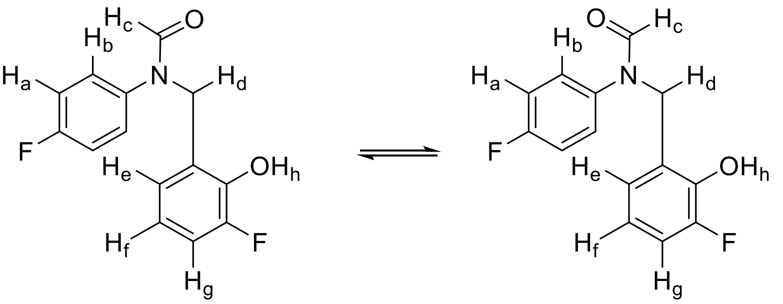

^{13}C assignments

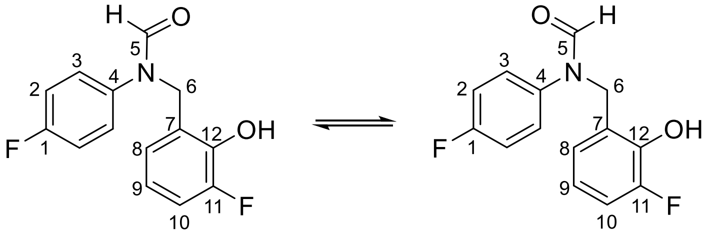

^{1}H NMR (500 MHz, DMSO) δ 9.76 (s, 2H, $\mathbf{H_{h}}$ closed, $\mathbf{H_{h}}$ open), 8.56 (s, 1H, $\mathbf{H_{c}}$ closed), 8.54 (s, 1H, $\mathbf{H_{c}}$ open), 7.39 – 7.31 (m, 2H, $\mathbf{H_{b}}$ closed), 7.32 – 7.24 (m, 2H, $\mathbf{H_{b}}$ open), 7.23 – 7.15 (m, 2H, $\mathbf{H_{a}}$ closed), 7.17 – 7.08 (m, 2H, $\mathbf{H_{a}}$ open), 7.03 (t, J = 8.9 Hz, 1H, $\mathbf{H_{f}}$ open), 7.00 (t, J = 9.2 Hz, 1H, $\mathbf{H_{f}}$ closed), 6.83 (d, J = 7.7 Hz, 1H, $\mathbf{H_{e}}$ closed), 6.79 (d, J = 7.7 Hz, 1H, $\mathbf{H_{e}}$ open), 6.69 (td, J = 7.8, 5.0 Hz, 1H, $\mathbf{H_{g}}$ closed), 6.64 (td, J = 7.8, 5.1 Hz, 1H, $\mathbf{H_{g}}$ open), 4.96 (s, 2H, $\mathbf{H_{d}}$ closed), 4.89 (s, 2H, $\mathbf{H_{d}}$ open).

Closed conformer (major):

^{13}C NMR (126 MHz, DMSO) δ 162.39 ($\mathbf{C_{5}}$), 160.09 (d, J = 243.3 Hz, $\mathbf{C_{1}}$), 151.42 (d, J = 238.5 Hz, $\mathbf{C_{11}}$), 142.64 (d, J = 14.7 Hz, $\mathbf{C_{12}}$), 137.15 (d, J = 2.8 Hz, $\mathbf{C_{4}}$), 126.17 (d, J = 2.8 Hz, $\mathbf{C_{7}}$), 125.15 (d, J = 8.5 Hz, $\mathbf{C_{3}}$), 123.49 (d, J = 2.9 Hz, $\mathbf{C_{8}}$), 118.60 (d, J = 7.0 Hz, $\mathbf{C_{9}}$), 115.95 (d, J = 22.6 Hz, $\mathbf{C_{1}}$), 114.42 (d, J = 18.5 Hz, $\mathbf{C_{10}}$), 42.34 (d, J = 3.4 Hz, $\mathbf{C_{6}}$). ^{19}F NMR (471 MHz, DMSO) δ -116.50 (td, J = 8.0, 3.7 Hz), -135.94 (dd, J = 11.0, 5.2 Hz).

Open conformer (minor):

^{13}C NMR (126 MHz, DMSO) δ 163.18 ($\mathbf{C_{5}}$), 159.75 (d, J = 243.2 Hz, $\mathbf{C_{1}}$), 151.46 (d, J = 238.8 Hz, $\mathbf{C_{11}}$), 143.42 (d, J = 13.6 Hz, $\mathbf{C_{12}}$), 135.01 (d, J = 2.8 Hz, $\mathbf{C_{4}}$), 127.42 (d, J = 8.5 Hz, $\mathbf{C_{3}}$), 126.36 (d, J = 2.6 Hz, $\mathbf{C_{7}}$), 124.76 (d, J = 2.8 Hz, $\mathbf{C_{8}}$), 118.30 (d, J = 7.1 Hz, $\mathbf{C_{9}}$), 115.27 (d, J = 22.5 Hz, $\mathbf{C_{3}}$), 115.05 (d, J = 18.5 Hz, $\mathbf{C_{10}}$), 48.08 (d, J = 3.1 Hz, $\mathbf{C_{6}}$). ^{19}F NMR (471 MHz, DMSO) δ -116.02 (td, J = 9.1, 5.1 Hz), -135.79 (dd, J = 10.9, 5.4 Hz).

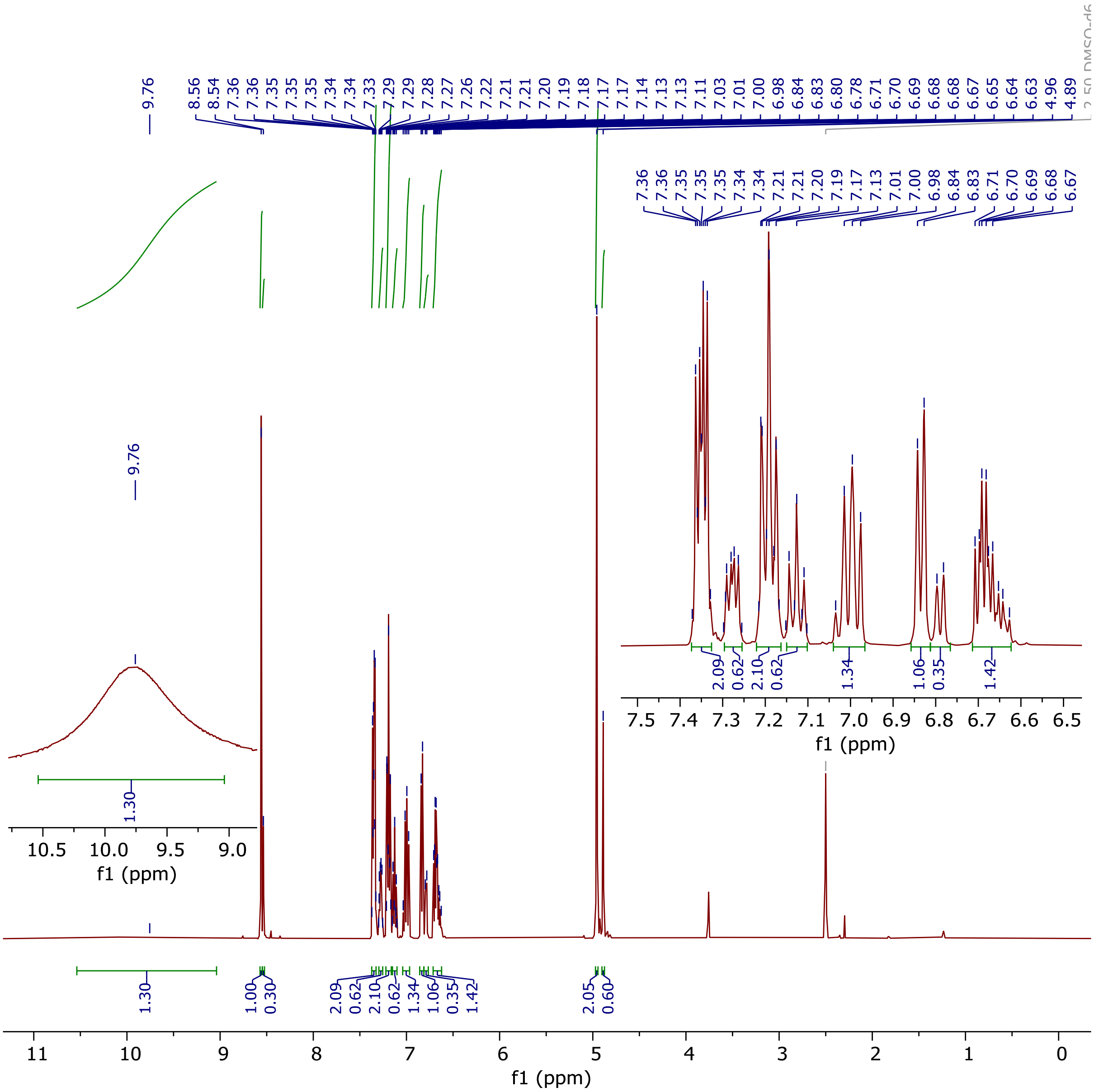

Figure S120: ^{1}H NMR spectrum (DMSO-d_6) of compound **1**-F.

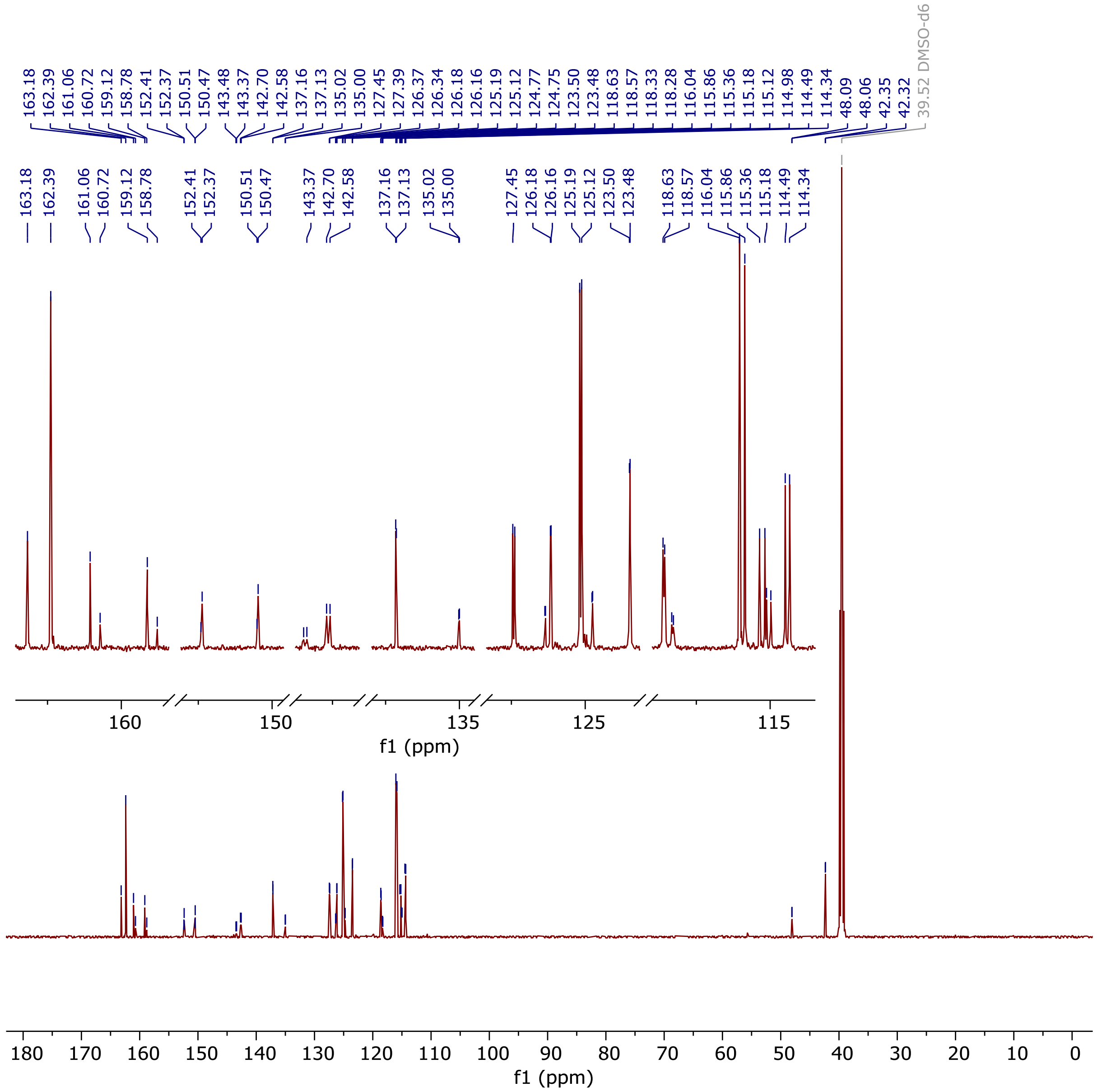

Figure S121: ^{13}C NMR spectrum (DMSO-d_6) of compound **1**-F.

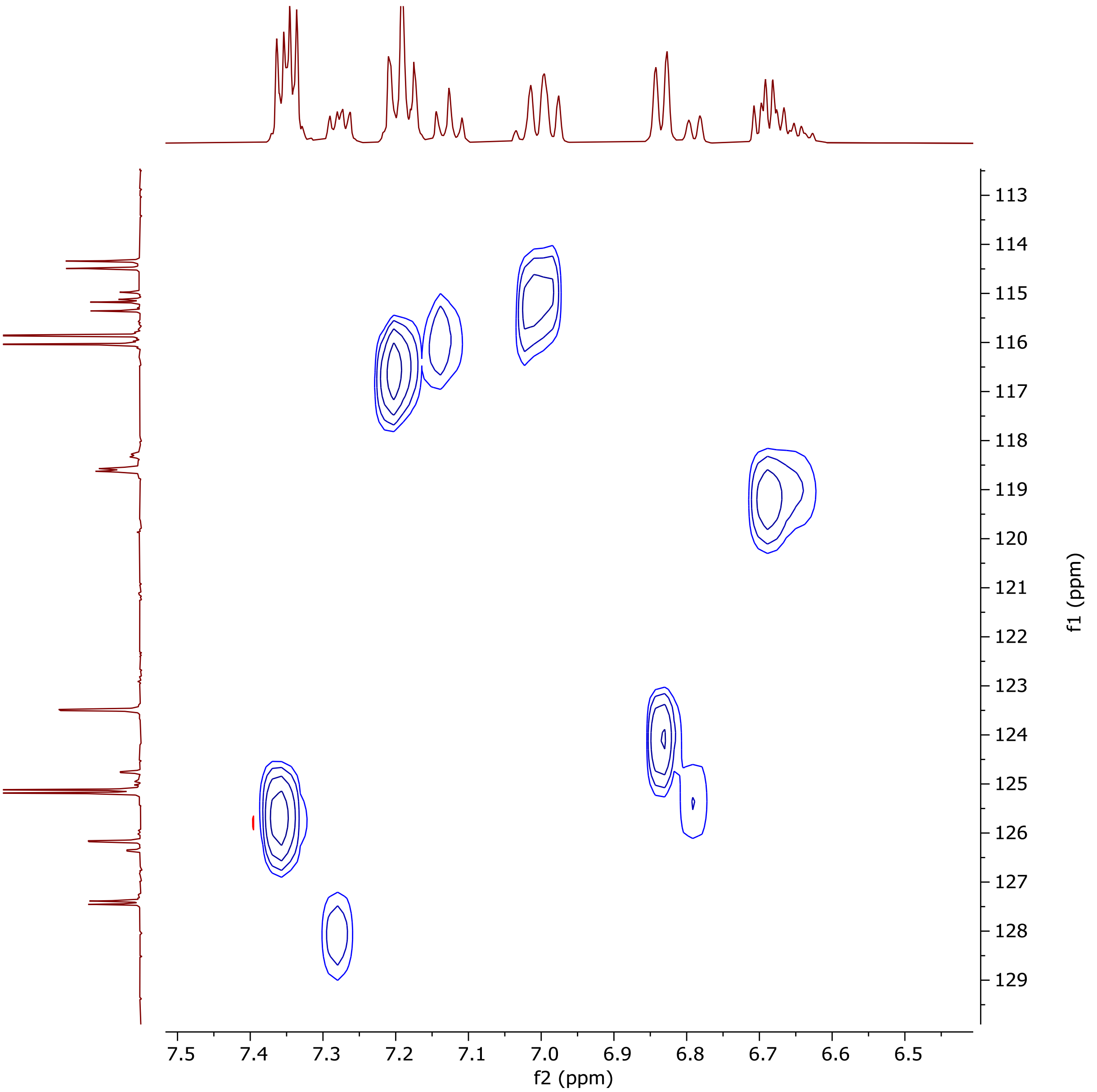

Figure S122: HSQC NMR spectrum (DMSO-d_6) of compound **1**-F.

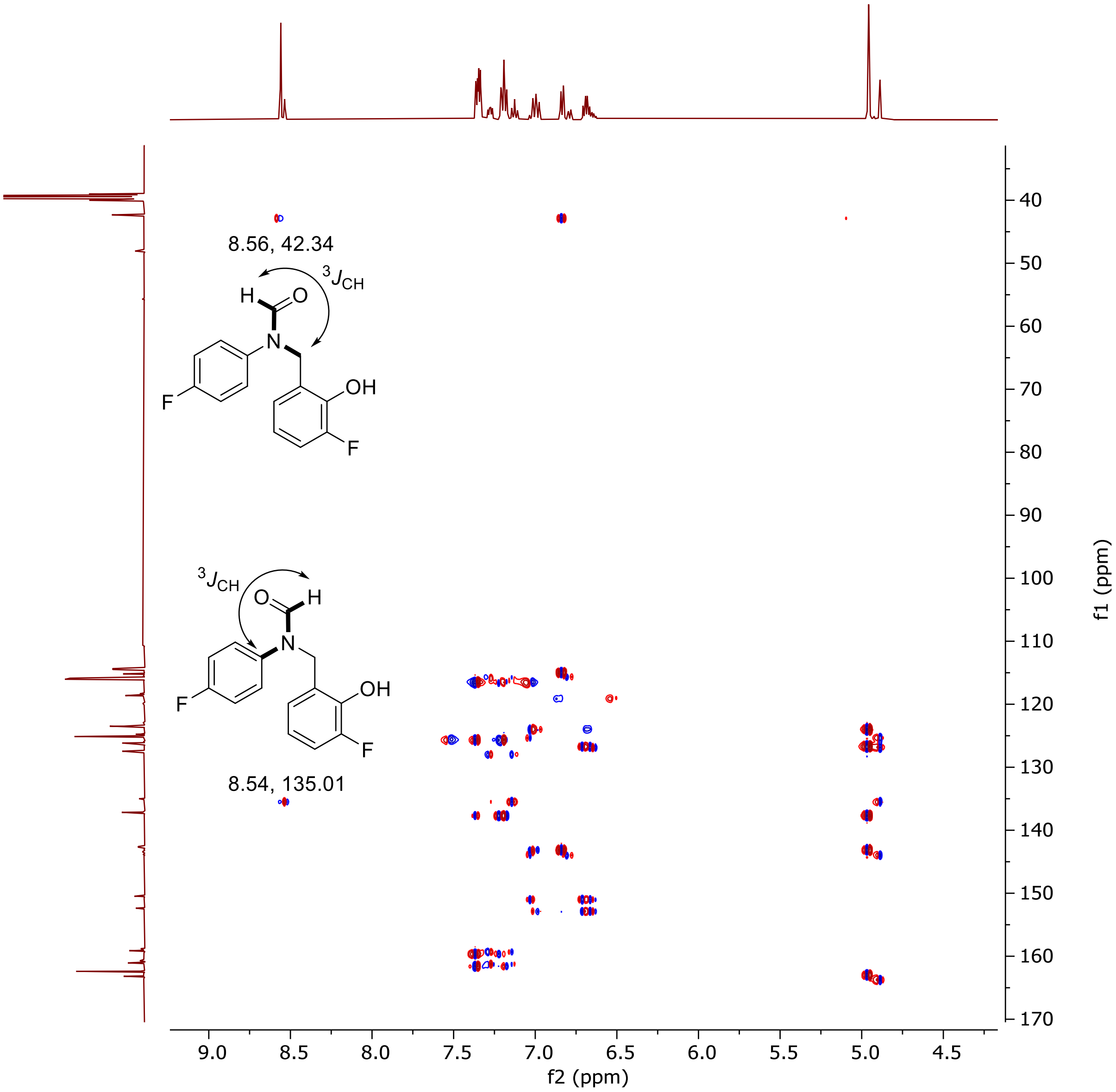

Figure S123: HMBC NMR spectrum (DMSO-d_6) of compound **1**-F.

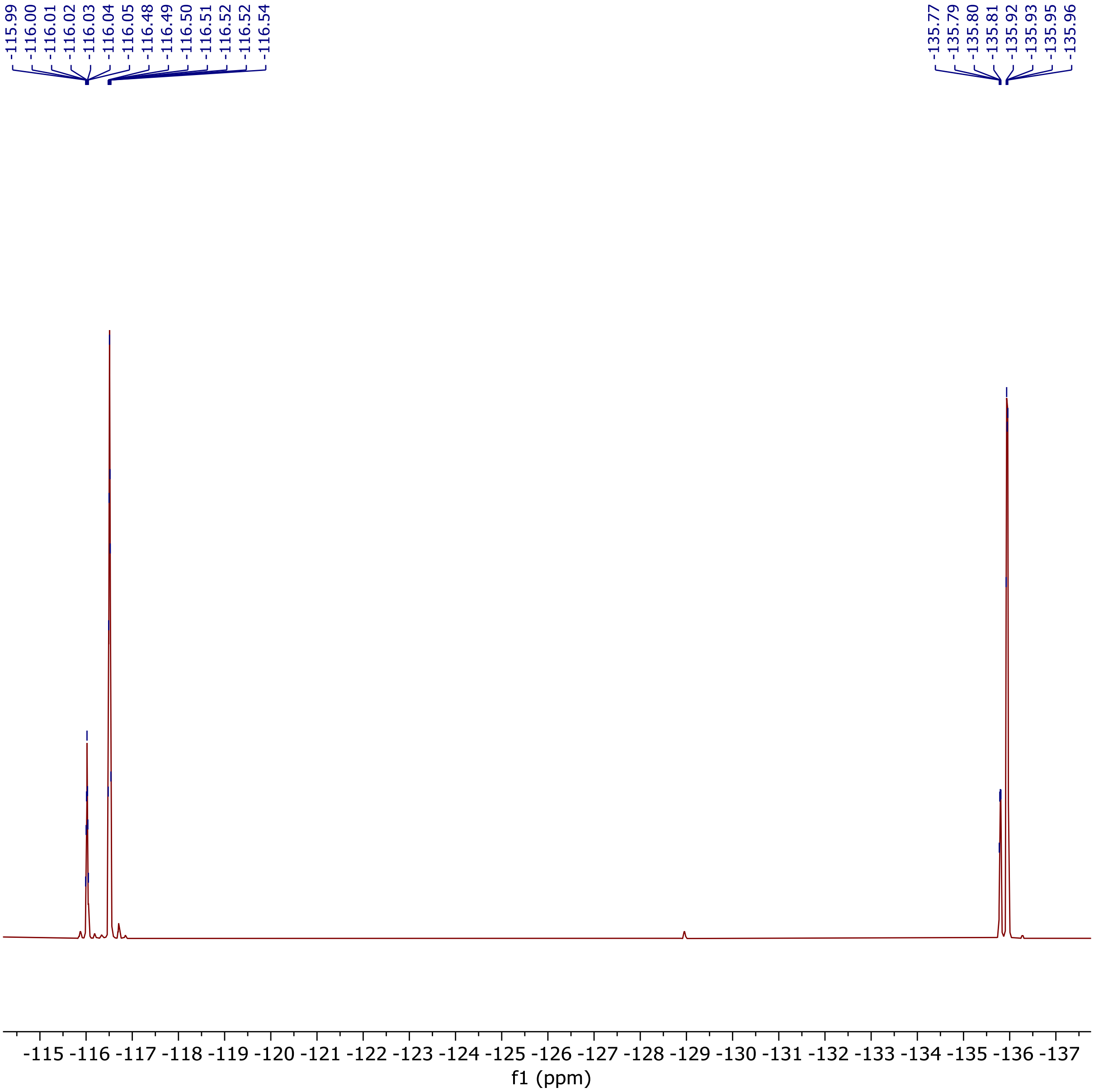

Figure S124: ^{19}F NMR spectrum (DMSO-d_6) of compound **1**-F.

***N*-(4-fluorophenyl)-*N*-[(2-hydroxy-3-methylphenyl)methyl]formamide (1-Me)**

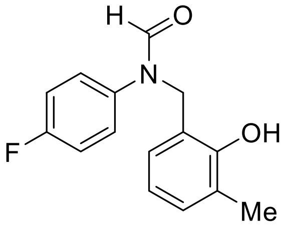

Following general procedure C, compound **3**-Me (3.0 mmol, 693 mg) was dissolved in 2 mL DCM and cooled down to 0 °C. FAM (3 eq, made from 13.5 mmol formic acid, 510 µL and 9 mmol acetic anhydride, 850 µL) was added in one portion and the reaction was stirred for 8 h. After removal of volatiles, the residue was purified via flash chromatography (eluting with 100% DCM) to give the title compound as a colourless oil (590 mg, 76%). HRMS (ESI+): m/z calcd. for $C_{15}H_{15}FNO_2$ $[M+H]^+$: 260.1081, found 260.1089.

^{1}H assignments

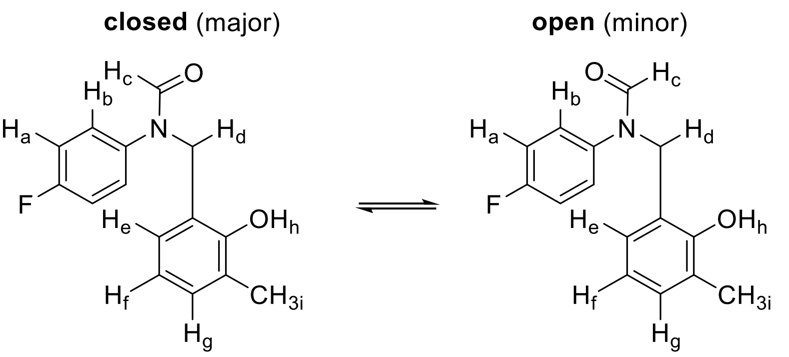

^{13}C assignments

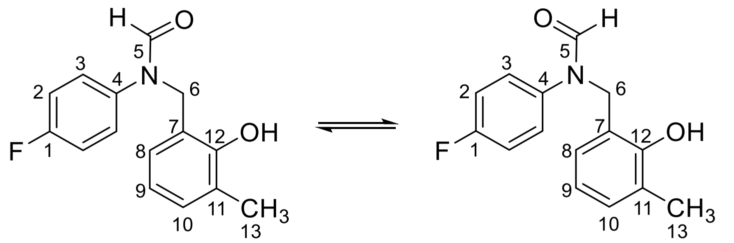

^{1}H NMR (500 MHz, DMSO) δ 8.61 (s, 2H, $\mathbf{H_h}$ $_{closed,}$ $\mathbf{H_h}$ $_{open}$), 8.56 (s, 1H, $\mathbf{H_c}$ $_{closed}$), 8.53 (s, 1H, $\mathbf{H_c}$ $_{open}$), 7.38 – 7.34 (m, 2H, $\mathbf{H_b}$ $_{closed}$), 7.30 – 7.25 (m, 2H, $\mathbf{H_b}$ $_{open}$), 7.24 – 7.19 (m, 2H, $\mathbf{H_a}$ $_{closed}$), 7.16 – 7.10 (m, 2H, $\mathbf{H_a}$ $_{open}$), 6.96 (d, J = 7.4 Hz, 2H, $\mathbf{H_g}$ $_{closed}$, $\mathbf{H_g}$ $_{open}$), 6.80 (d, J = 7.1 Hz, 2H, $\mathbf{H_e}$ $_{closed}$, $\mathbf{H_e}$ $_{open}$), 6.64 (t, J = 7.6 Hz, 1H, $\mathbf{H_f}$ $_{closed}$), 6.63 (t, J = 7.5 Hz, 1H, $\mathbf{H_f}$ $_{open}$), 4.93 (s, 2H, $\mathbf{H_d}$ $_{closed}$), 4.86 (s, 2H, $\mathbf{H_d}$ $_{open}$), 2.16 (s, 3H, $\mathbf{H_i}$ $_{open}$), 2.15 (s, 3H, $\mathbf{H_i}$ $_{closed}$).

Closed conformer (major):

^{13}C NMR (126 MHz, DMSO) δ 162.84 ($\mathbf{C_5}$), 160.18 (d, J = 243.1 Hz, $\mathbf{C_1}$), 152.64 ($\mathbf{C_{12}}$), 137.28 (d, J = 2.8 Hz, $\mathbf{C_4}$), 129.68 ($\mathbf{C_{10}}$), 125.90 ($\mathbf{C_8}$), 125.25 (d, J = 8.5 Hz, $\mathbf{C_3}$), 124.83 ($\mathbf{C_{11}}$), 123.04 ($\mathbf{C_7}$), 119.28 ($\mathbf{C_9}$), 116.08 (d, J = 22.6 Hz, $\mathbf{C_2}$), 43.49 ($\mathbf{C_6}$), 16.40 ($\mathbf{C_{13}}$). ^{19}F NMR (471 MHz, DMSO) δ -116.42 (tt, J = 8.9, 4.8 Hz).

Open conformer (minor):

^{13}C NMR (126 MHz, DMSO) δ 163.32 ($\mathbf{C_5}$), 159.72 (d, J = 242.8 Hz, $\mathbf{C_1}$), 153.21 ($\mathbf{C_{12}}$), 135.24 (d, J = 2.9 Hz, $\mathbf{C_4}$), 130.24 ($\mathbf{C_{10}}$), 127.38 (d, J = 8.4 Hz, $\mathbf{C_3}$), 126.97 ($\mathbf{C_8}$), 124.71 ($\mathbf{C_{11}}$), 123.74 ($\mathbf{C_7}$), 119.25 ($\mathbf{C_9}$), 115.31 (d, J = 22.4 Hz, $\mathbf{C_2}$), 48.79 ($\mathbf{C_6}$), 16.46 ($\mathbf{C_{13}}$). ^{19}F NMR (471 MHz, DMSO) δ -116.19 (ddd, J = 13.7, 8.8, 5.1 Hz)

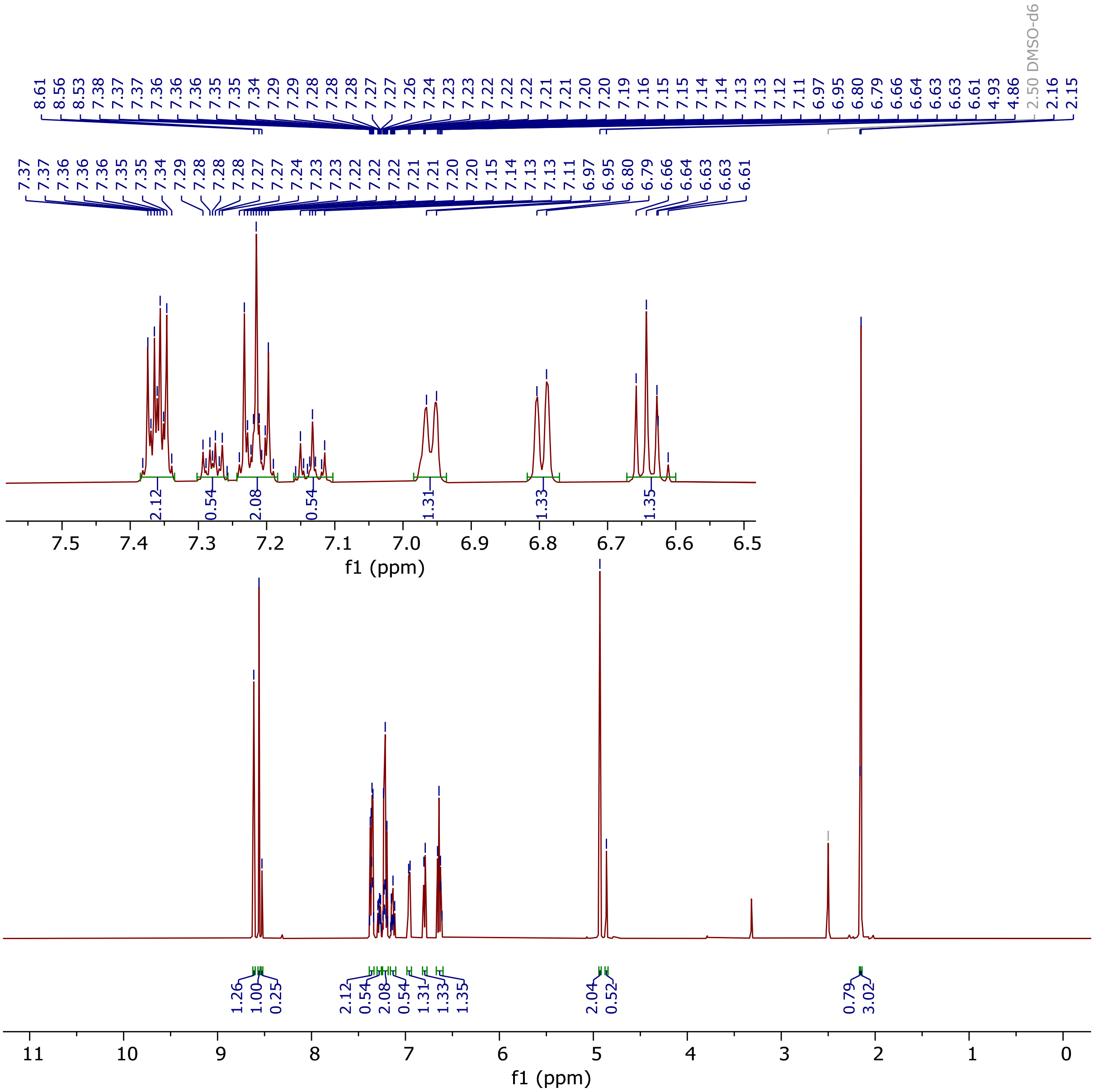

Figure S125: ^{1}H NMR spectrum (DMSO-d_6) of compound **1**-Me.

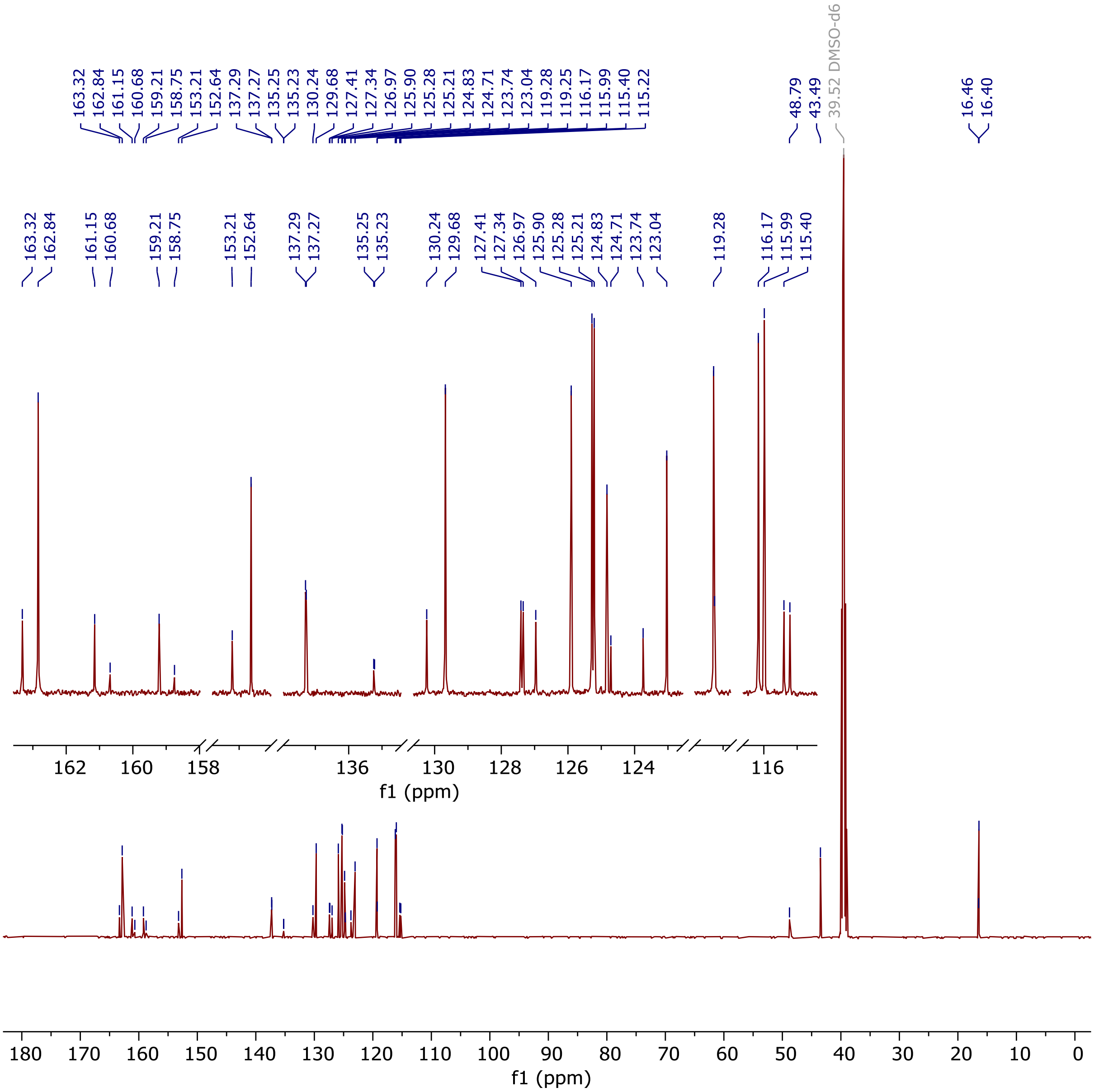

Figure S126: ^{13}C NMR spectrum (DMSO-d_6) of compound **1**-Me.

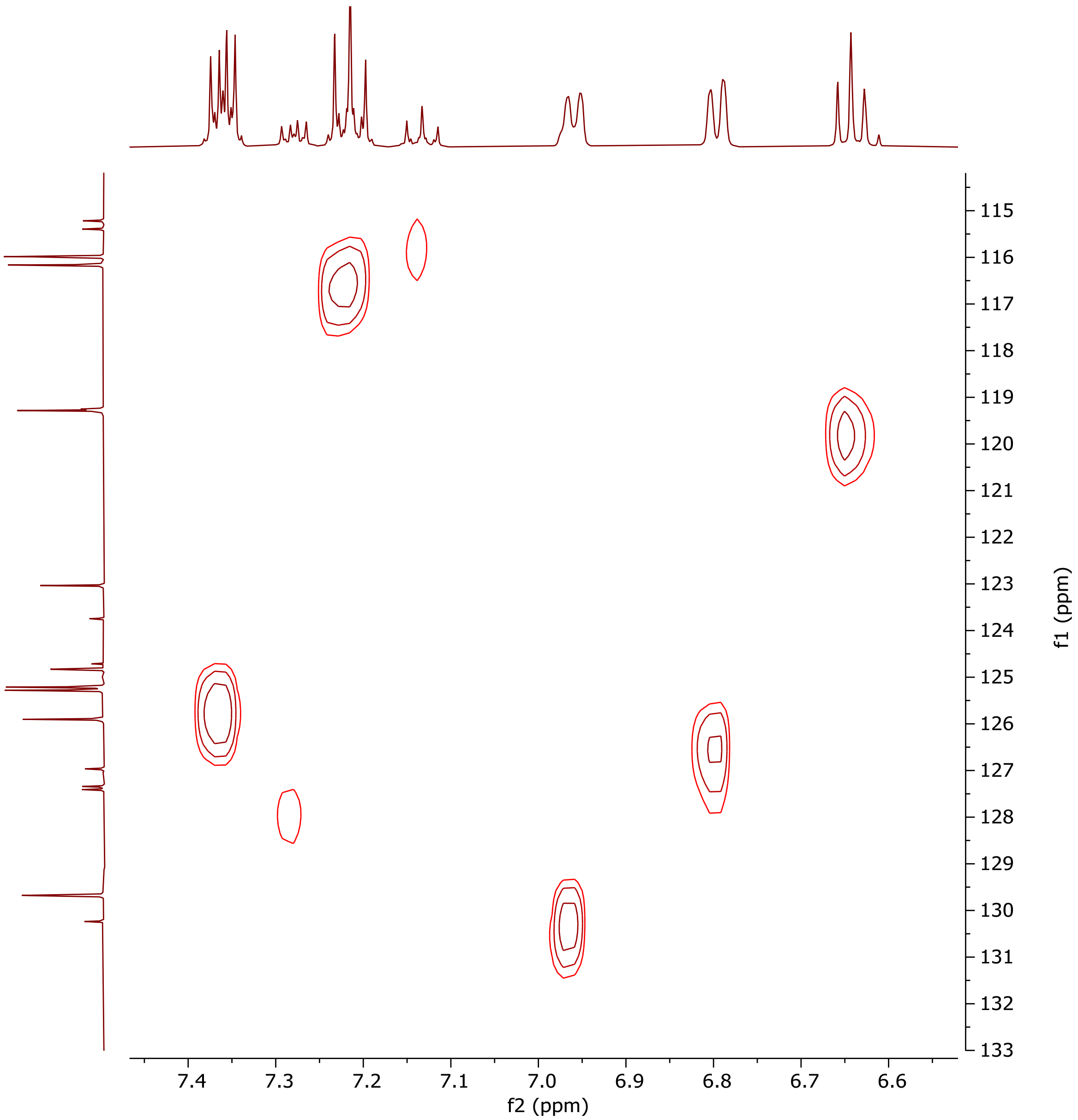

Figure S127: HSQC NMR spectrum (DMSO-d_6) of compound **1**-Me.

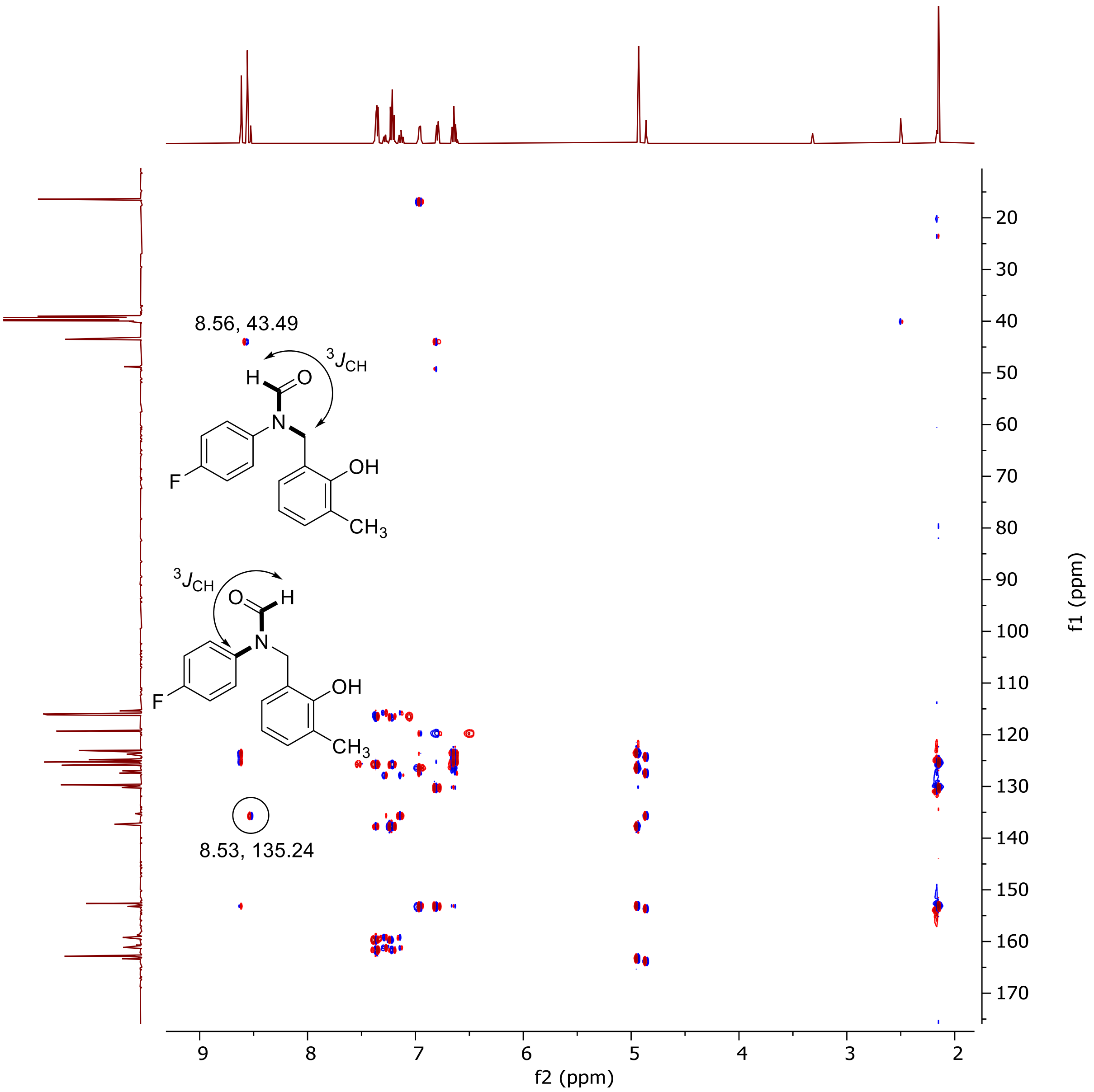

Figure S128: HMBC NMR spectrum (DMSO-d_6) of compound **1**-Me.

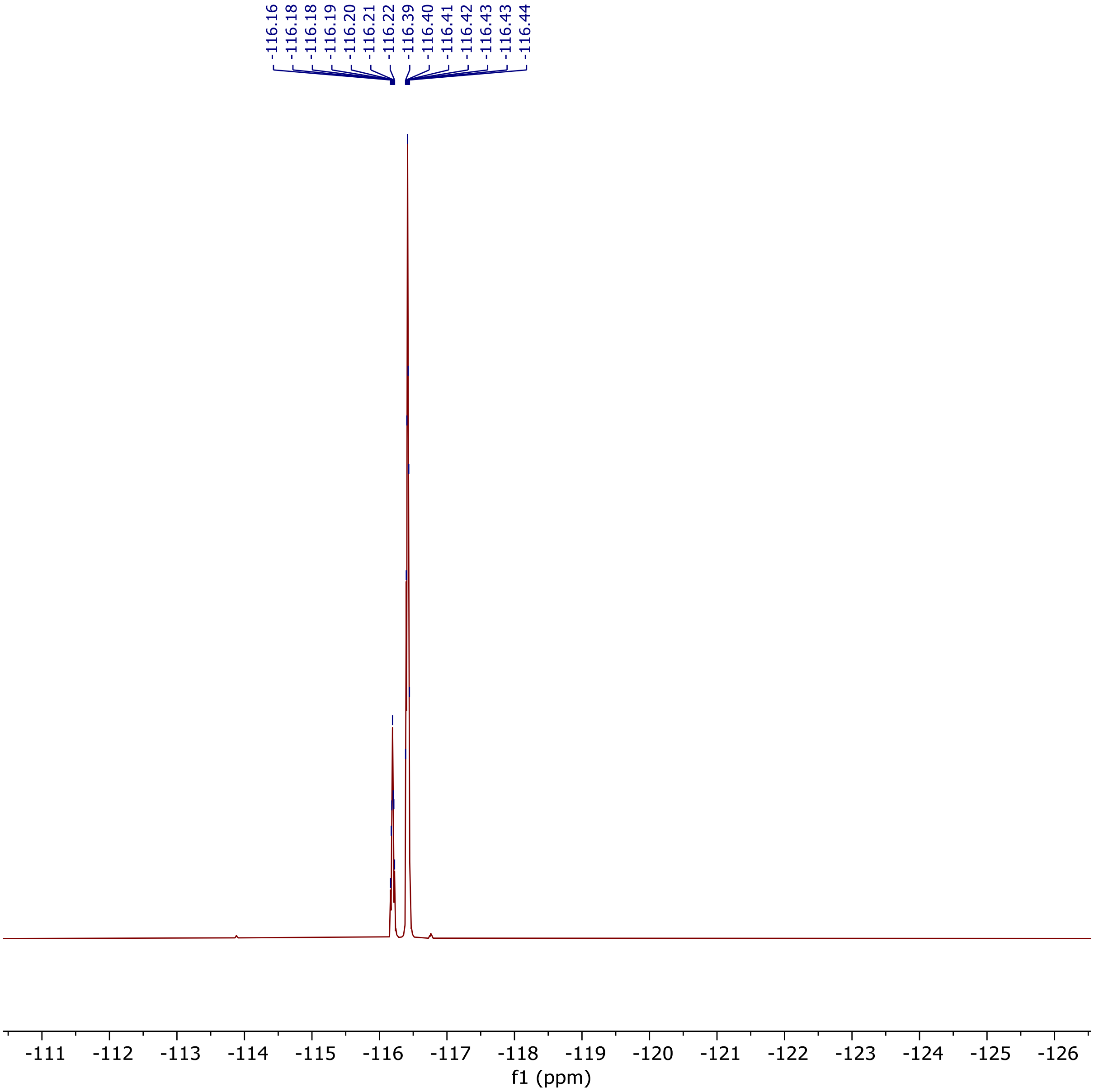

Figure S129: ^{19}F NMR spectrum (DMSO-d_6) of compound **1**-Me.

_N_-(4-fluorophenyl)-_N_-[(2-hydroxy-3-phenylphenyl)methyl]formamide (1-Ph)

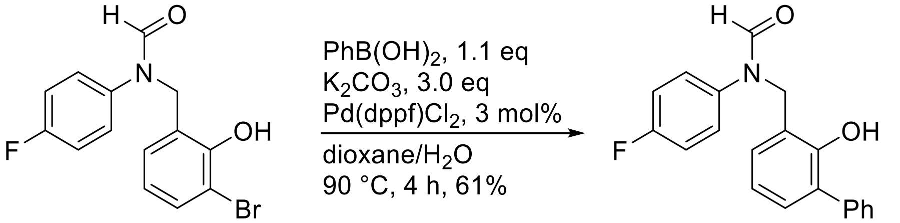

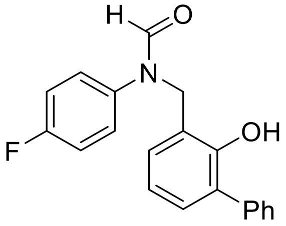

Under the atmosphere of N_2 In a 10 mL flask were combined **1**-Br, (324 mg, 1.0 mmol, 1.0 eq), phenylboronic acid (134 mg, 1.1 mmol, 1.1 eq), 5 mL of dioxane and the mixture was thoroughly degassed. Pd(dppf)Cl_2 (22 mg, 3 mol%) was added to the mixture, followed by a degassed solution of K_2CO_3 (414 mg, 3.0 mmol, 3.0 eq) in 2 mL H_2O. The reaction mixture was stirred at 90 °C for 4 h, after which time the starting material was fully consumed (TLC 100% $CHCl_3$). The reaction mixture was

diluted with 50 mL DCM and washed with 1 M HCl (1 x 10 mL), water (1 x 10 mL), brine and dried over $MgSO_4$. After evaporation of the solvents, the crude material was purified *via* flash chromatography (100% $CHCl_3$) to provide the title compound as a colourless oil which slowly solidified to give a colourless solid (197 mg, 61%). Mp 89 – 91 °C. HRMS (ESI+): m/z calcd. for $C_{20}H_{17}FNO_2$ $[M+H]^+$: 322.1238, found 322.1217

closed (major)

^{1}H assignments

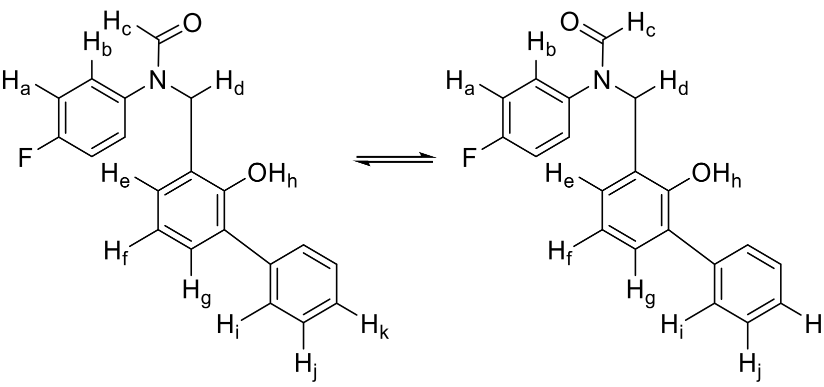

^{13}C assignments

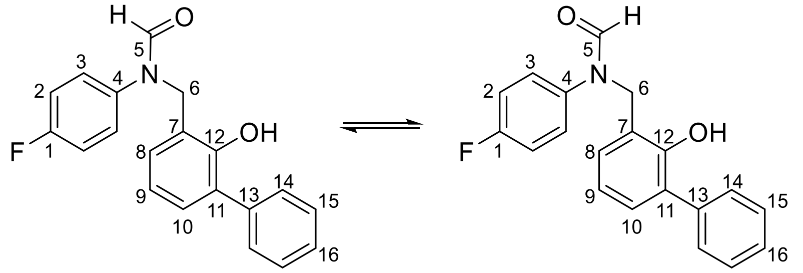

^{1}H NMR (500 MHz, DMSO) δ 8.78 (s, 2H, **H$_h$**$_{closed}$, **H$_h$**$_{open}$), 8.59 (s, 1H, **H$_c$**$_{closed}$), 8.55 (s, 1H, **H$_c$**$_{open}$), 7.50 – 7.46 (m, 4H, **H$_j$**$_{closed}$, **H$_j$**$_{open}$), 7.45 – 7.39 (m, 8H **H$_i$**$_{closed}$, **H$_i$**$_{open}$, **H$_a$**$_{closed}$, **H$_a$**$_{open}$), 7.39 – 7.32 (m, 1H, **H$_k$**$_{open}$), 7.35 – 7.28 (m, 1H, **H$_k$**$_{closed}$), 7.28 – 7.22 (m, 2H, **H$_a$**$_{closed}$), 7.19 – 7.14 (m, 2H, **H$_a$**$_{open}$), 7.13 (dd, *J* = 7.6, 1.7 Hz, 1H, **H$_g$**$_{closed}$), 7.10 (dd, *J* = 7.6, 1.6 Hz, 1H, H$_g$$_{open}$), 7.00 (dd, *J* = 7.7, 1.7 Hz, 1H, **H$_e$**$_{open}$), 6.98 (dd, *J* = 7.6, 1.7 Hz, 1H, **H$_e$**$_{closed}$), 6.86 (t, *J* = 7.6 Hz, 1H, **H$_f$**$_{closed}$), 6.85 (t, *J* = 7.6 Hz, 1H, **H$_f$**$_{open}$), 5.01 (s, 2H, **H$_d$**$_{closed}$), 4.97 (s, 2H, **H$_d$**$_{open}$).

Closed conformer (major):

^{13}C NMR (126 MHz, DMSO) δ 163.13 (**C$_5$**), 160.31 (d, *J* = 243.3 Hz, **C$_1$**), 151.38 (**C$_{12}$**), 138.40 (**C$_{13}$**), 137.19 (d, *J* = 2.7 Hz, **C$_4$**), 130.13 (**C$_{10}$**), 129.70 (**C$_{11}$**), 129.22 (**C$_{15}$**), 128.11 (**C$_{14}$**), 127.64 (**C$_8$**), 126.79 (**C$_{16}$**), 125.43 (d, *J* = 8.5 Hz, **C$_3$**), 124.63 (**C$_7$**), 120.12 (**C$_9$**), 116.17 (d, *J* = 22.7 Hz, **C$_2$**), 44.01 (**C$_6$**). ^{19}F NMR (471 MHz, DMSO) δ -116.14 (tt, *J* = 8.8, 4.8 Hz).

Open conformer (minor):

13C NMR (126 MHz, DMSO) δ 163.32 (**C$_5$**), 159.78 (d, *J* = 242.7 Hz, **C$_1$**), 151.68 (**C$_{12}$**), 138.24 (**C$_{13}$**), 135.26 (d, *J* = 2.9 Hz, **C$_4$**), 130.38 (**C$_{10}$**), 130.05 (**C$_{11}$**), 129.18 (**C$_{15}$**), 128.11 (**C$_{14}$**), 127.36 (d, *J* = 8.5 Hz, **C$_3$**), 126.92 (**C$_8$**), 126.68 (**C$_{16}$**), 125.72 (**C$_7$**), 120.17 (**C$_9$**), 115.39 (d, *J* = 22.4 Hz, **C$_2$**), 48.72 (**C$_6$**). ^{19}F NMR (471 MHz, DMSO) δ -116.07 (td, *J* = 8.8, 4.5 Hz).

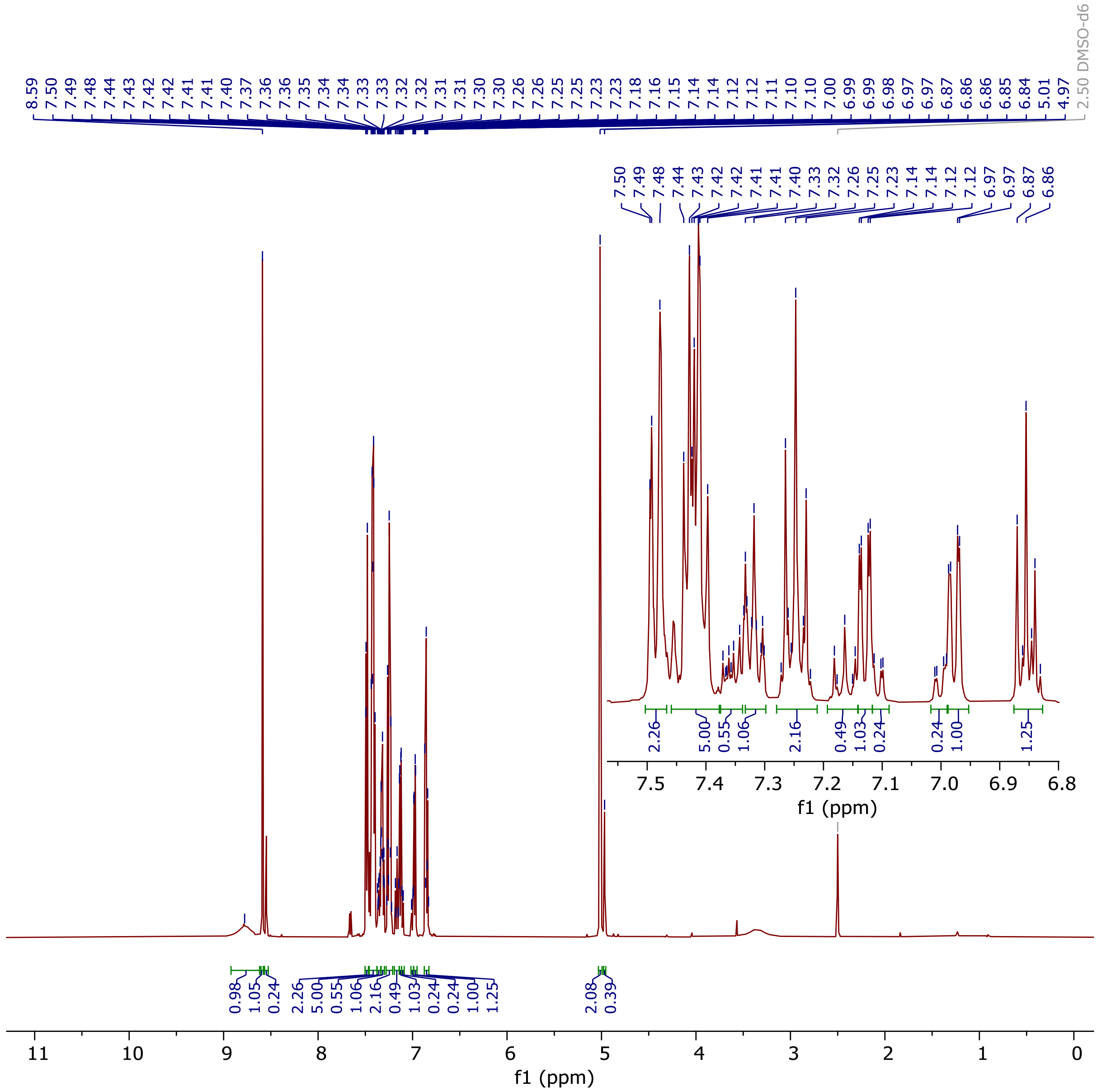

Figure S130: ^{1}H NMR spectrum (DMSO-d_6) of compound **1**-Ph.

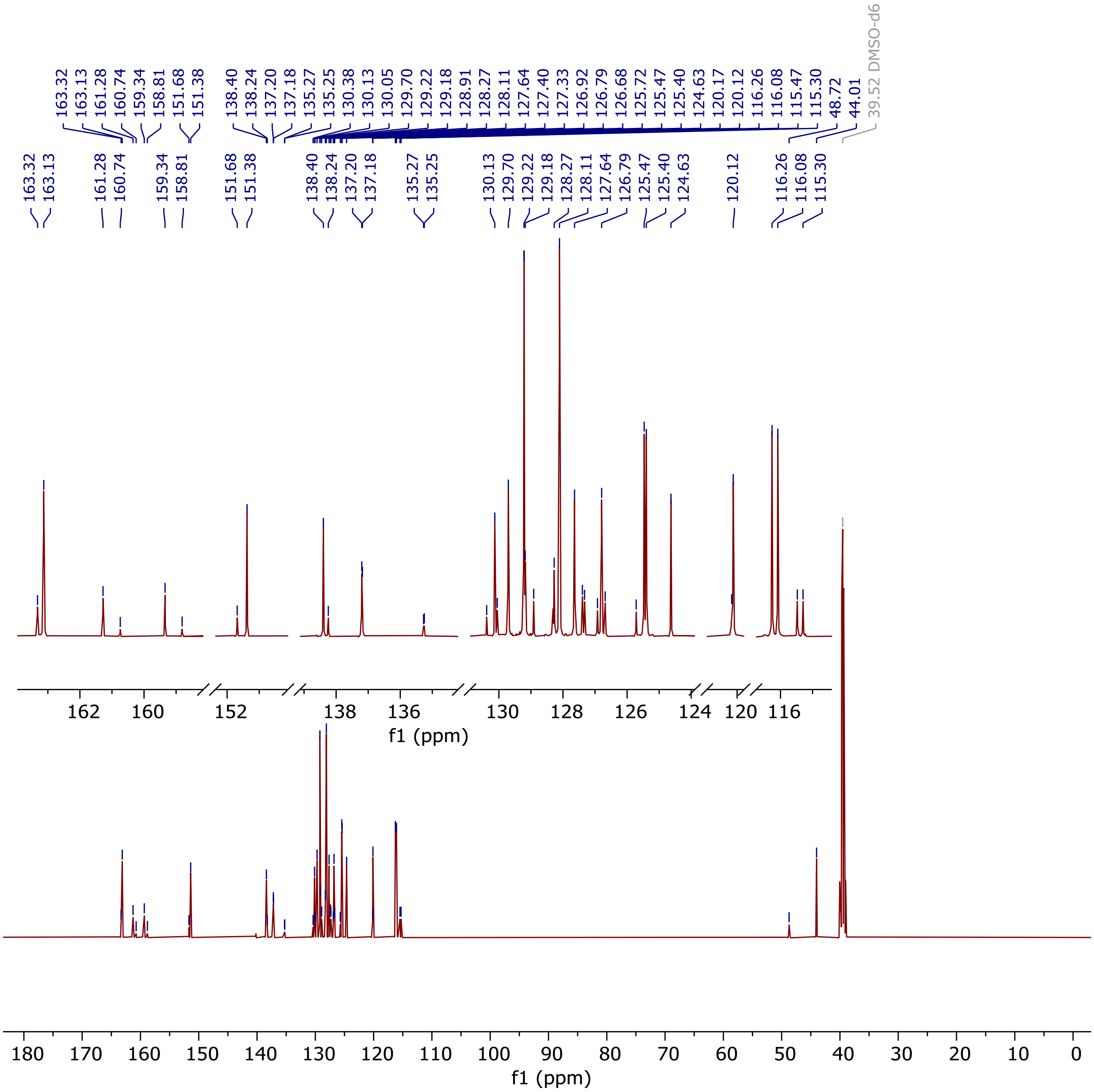

Figure S131: ^{13}C NMR spectrum (DMSO-d_6) of compound **1**-Ph.

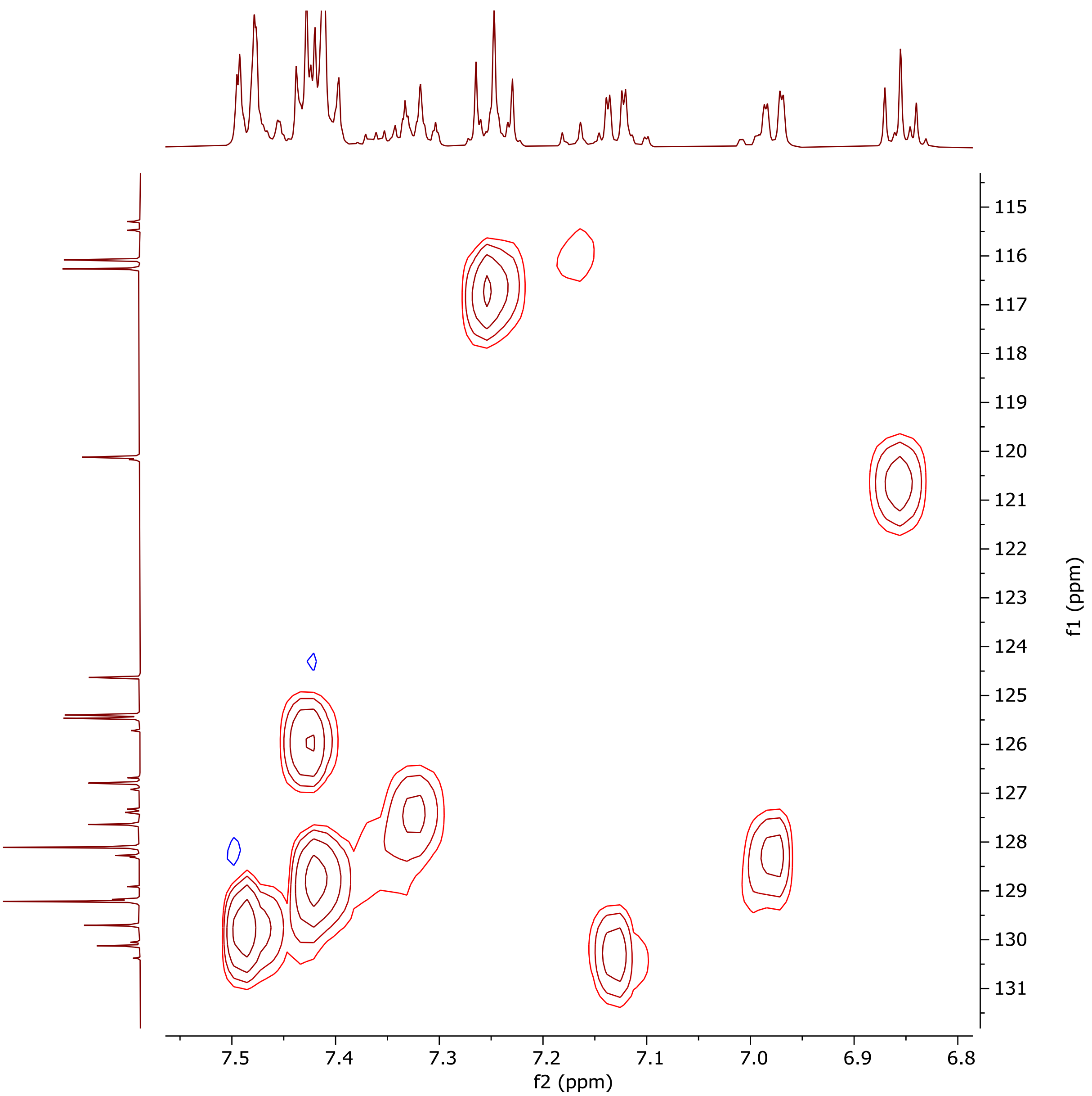

Figure S132: HSQC NMR spectrum (DMSO-d_6) of compound **1**-Ph.

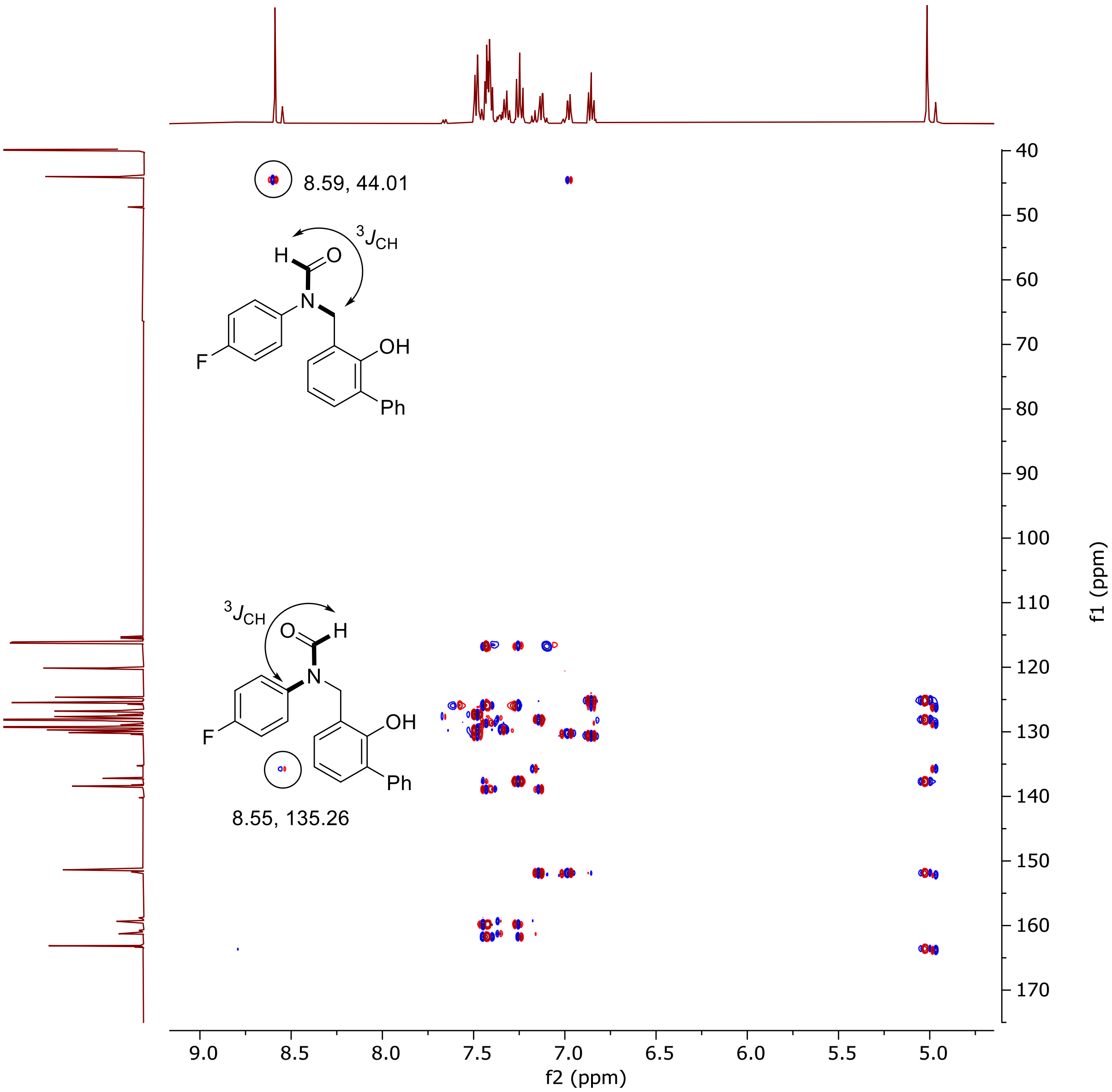

Figure S133: HMBC NMR spectrum (DMSO-d_6) of compound **1**-Ph.

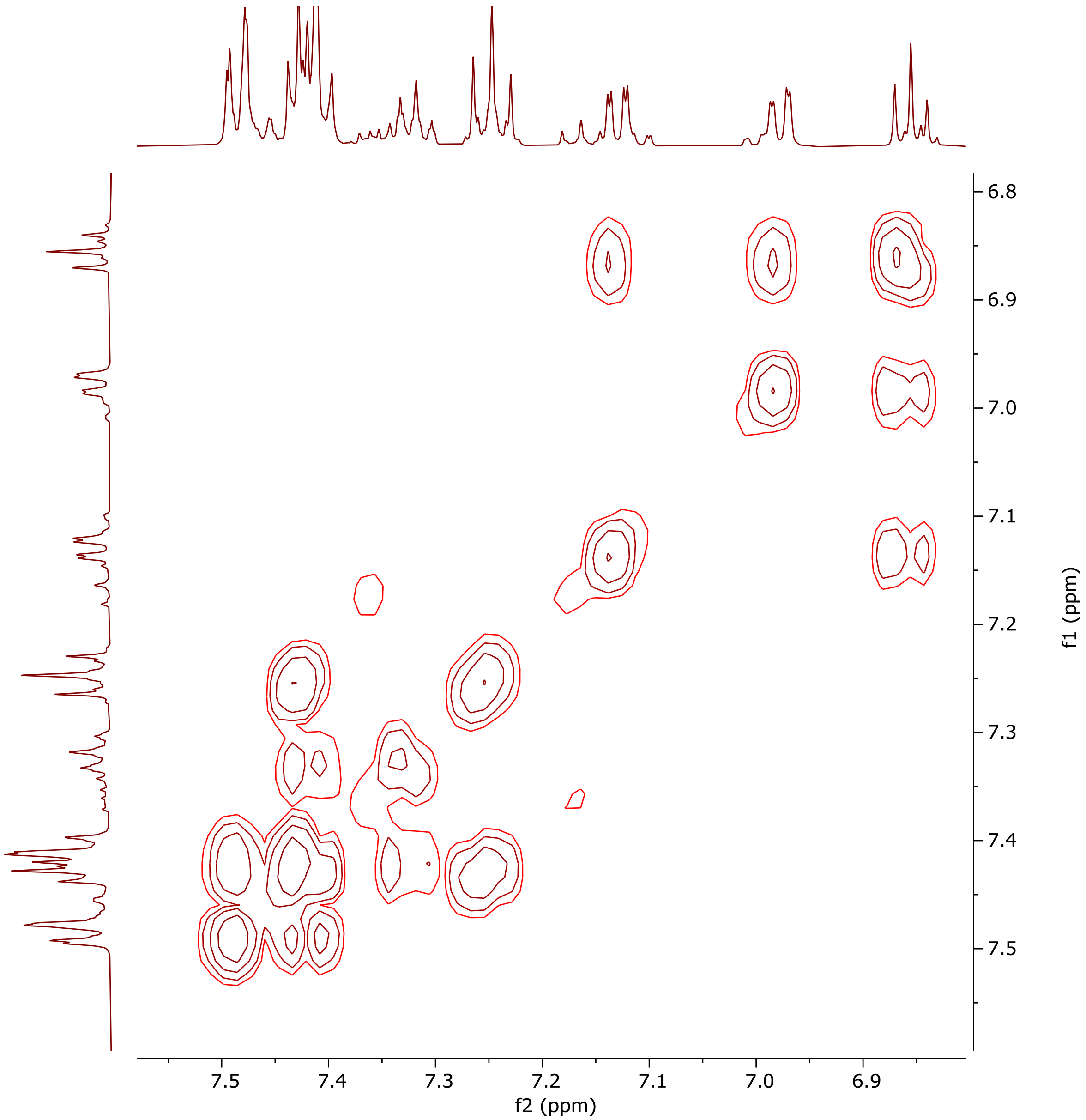

Figure S134: COSY NMR spectrum (DMSO-d_6) of compound **1**-Ph.

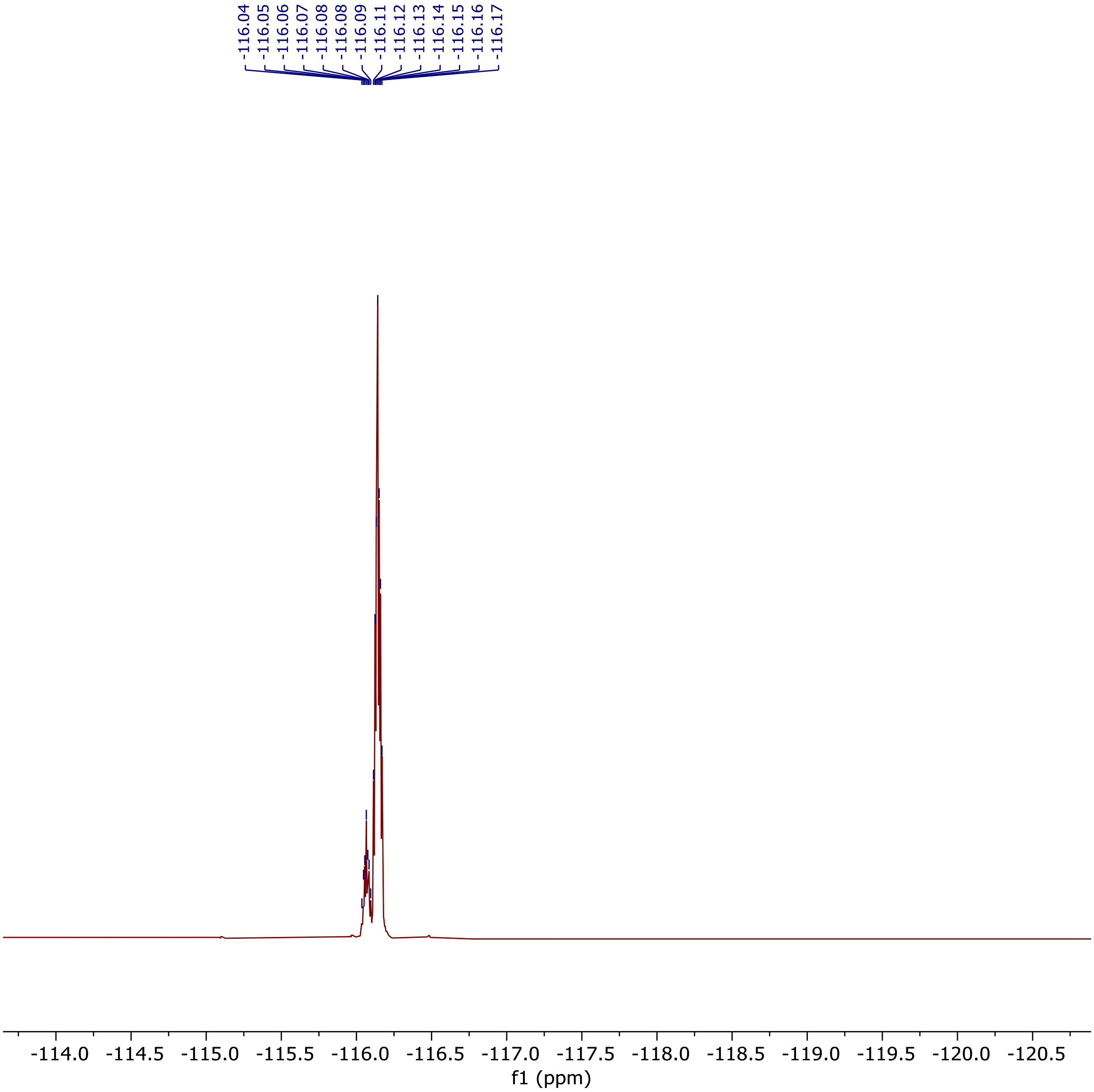

Figure S135: ^{19}F NMR spectrum (DMSO-d_6) of compound **1**-Ph.

***N*-(4-fluorophenyl)-*N*-[(2-hydroxy-3,5-diiodophenyl)methyl]formamide (1-I$_2$)**

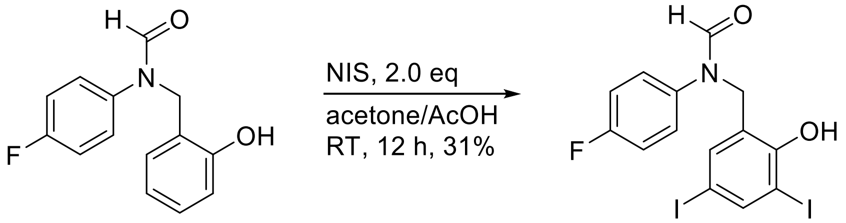

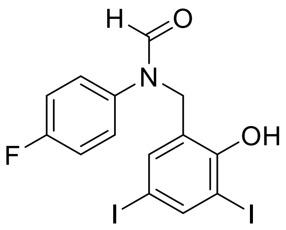

Compound **1**-H (245 mg, 1.0 mmol, 1.0 eq) and *N*-iodosuccinimide (450 mg, 2.0 mmol, 2.0 eq) were dissolved in 3 mL acetone. Acetic acid (1 mL) was added and the resulting mixture was stirred at room temperature overnight. The mixture was diluted with DCM, washed in succession with 5% aqueous $Na_2S_2O_3$, H_2O, brine and dried over Na_2SO_4. The solvent was removed under reduced pressure and the crude mixture was purified *via* flash column chromatography (eluent 100% $CHCl_3$). The title

compound was obtained as a colourless oil which slowly solidified to give a colourless solid (108 mg, 22%). Mp 115 – 117 °C. HRMS (ESI+): m/z calcd. for $C_{14}H_{11}FI_2NO_2$ $[M+H]^+$: 497.8858, found 497.8901.

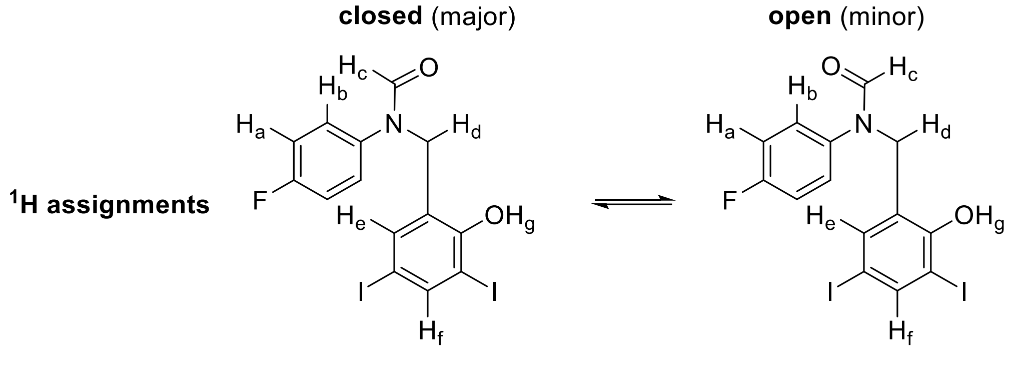

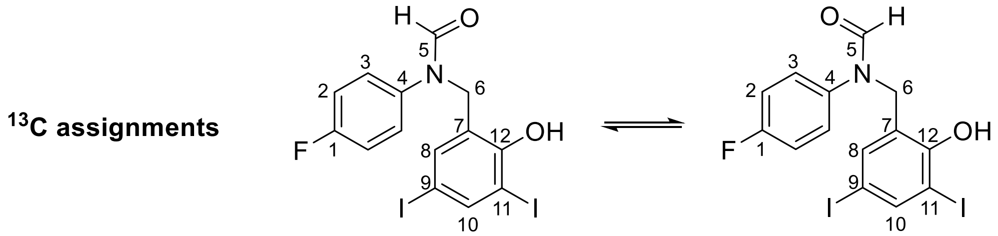

^{1}H NMR (500 MHz, DMSO) δ 9.62 (br s, 2H, **Hg** closed, **Hg** open), 8.56 (s, 1H, **Hc** closed), 8.50 (s, 1H, **Hc** open), 7.88 (d, *J* = 2.2 Hz, 2H, **Hf** closed, **Hf** open), 7.40 – 7.34 (m, 2H, **Hb** closed), 7.29 – 7.26 (m, 2H, **Hb** open), 7.27 – 7.21 (m, 4H, **Ha** closed, **He** closed, **He**open), 7.19 – 7.14 (m, 2H, **Ha** open), 4.92 (s, 2H, **Hd** closed), 4.89 (s, 2H, **Hd** open).

Major conformer

^{13}C NMR (126 MHz, DMSO) δ 163.18 (**C5**), 160.31 (d, *J* = 243.5 Hz, **C1**), 154.10 (**C12**), 144.66 (**C10**), 136.84 (d, *J* = 2.7 Hz, **C4**), 136.57 (**C8**), 127.22 (**C7**), 125.17 (d, *J* = 8.5 Hz, **C3**), 116.25 (d, *J* = 22.7 Hz, **C2**), 90.21 (**C11**), 83.57 (**C9**), 43.60 (**C6**). ^{19}F NMR (471 MHz, DMSO) δ -115.91 (tt, *J* = 8.6, 4.7 Hz).

Minor conformer

^{13}C NMR (126 MHz, DMSO) δ 163.28 (**C5**), 159.87 (d, *J* = 243.5 Hz, **C1**), 154.67 (**C12**), 145.02 (**C10**), 137.79 (**C8**), 134.68 (d, *J* = 2.8 Hz, **C4**), 128.09 (**C7**), 127.38 (d, *J* = 8.5 Hz, **C3**), 115.52 (d, *J* = 22.5 Hz, **C2**), 90.67 (**C11**), 83.62 (**C9**), 48.30 (**C6**). ^{19}F NMR (471 MHz, DMSO) δ -115.61 (tt, *J* = 8.9, 5.1 Hz).

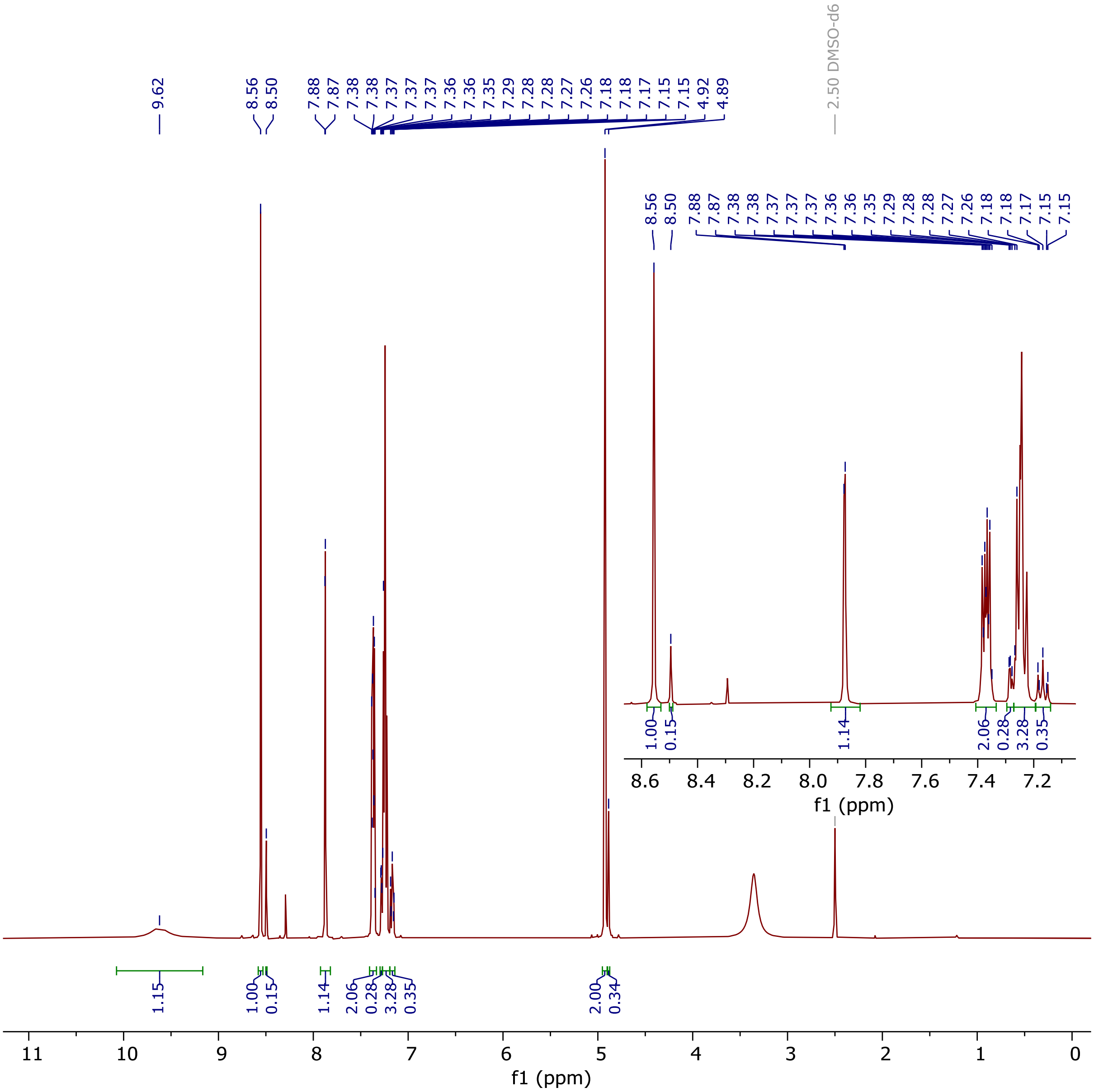

Figure S136: ^{1}H NMR spectrum (DMSO-d_6) of compound **1**-I_2.

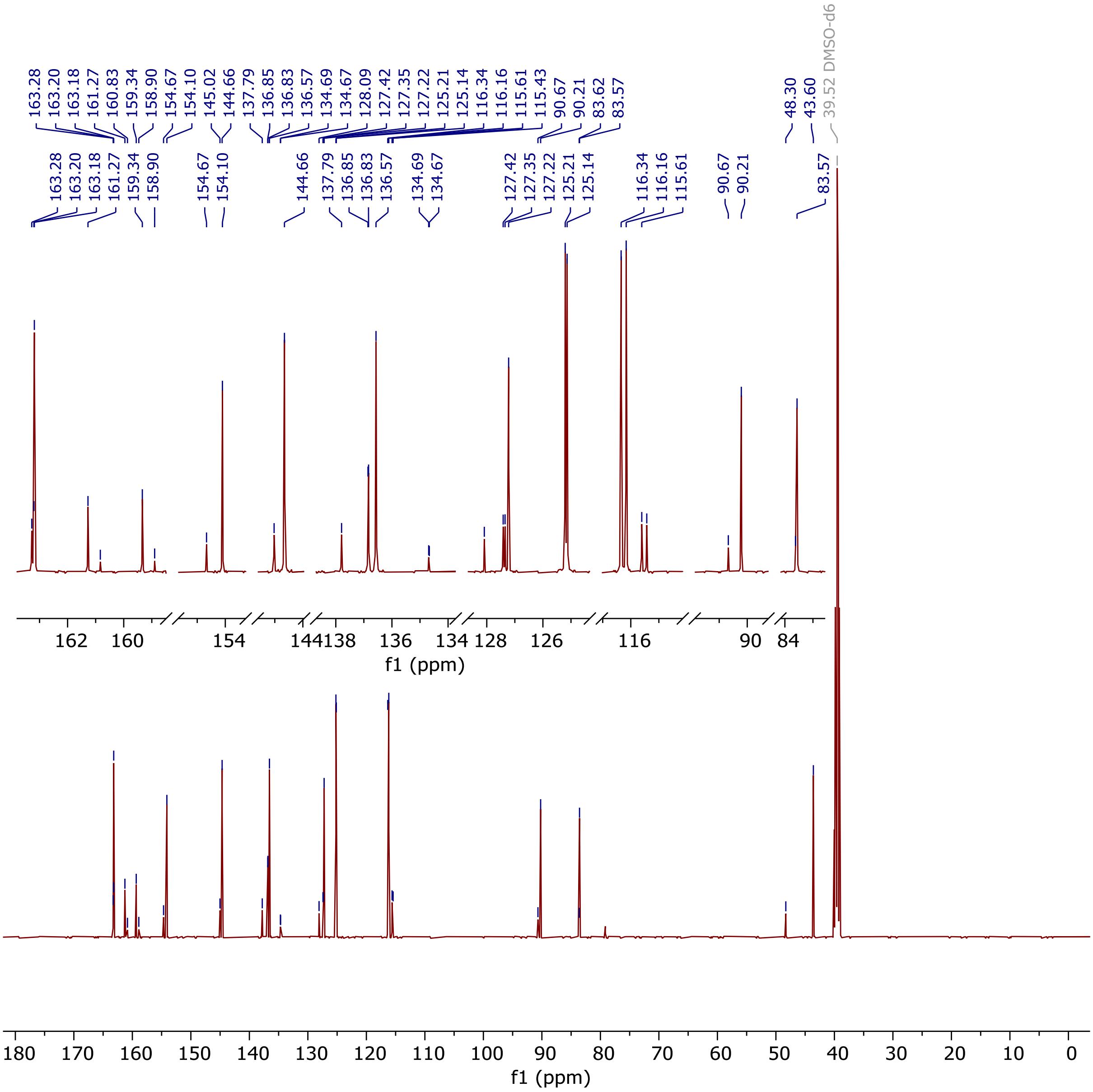

Figure S137: ^{13}C NMR spectrum (DMSO-d_6) of compound **1**-I_2.

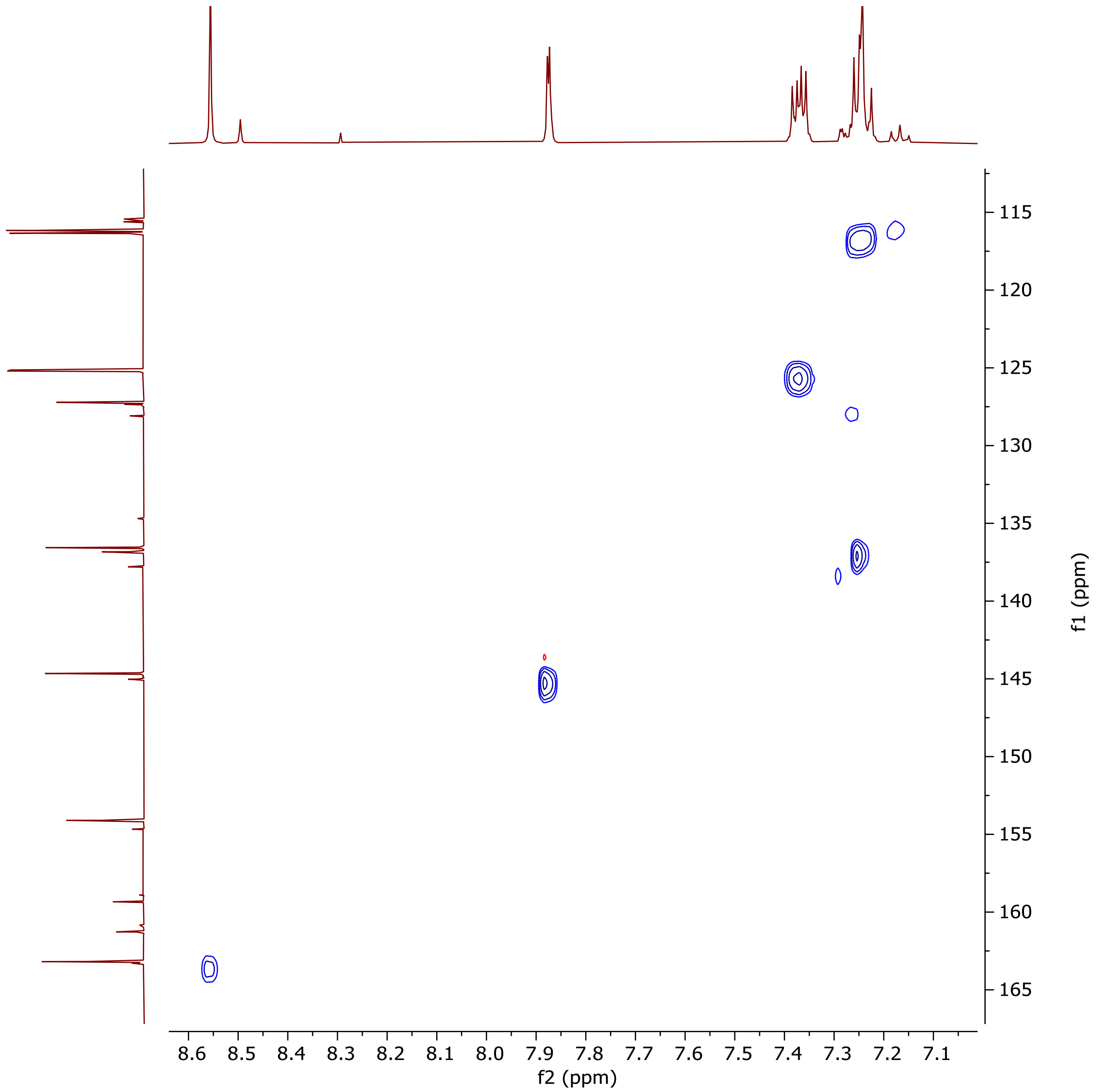

Figure S138: HSQC NMR spectrum (DMSO-d_6) of compound **1**-I_2.

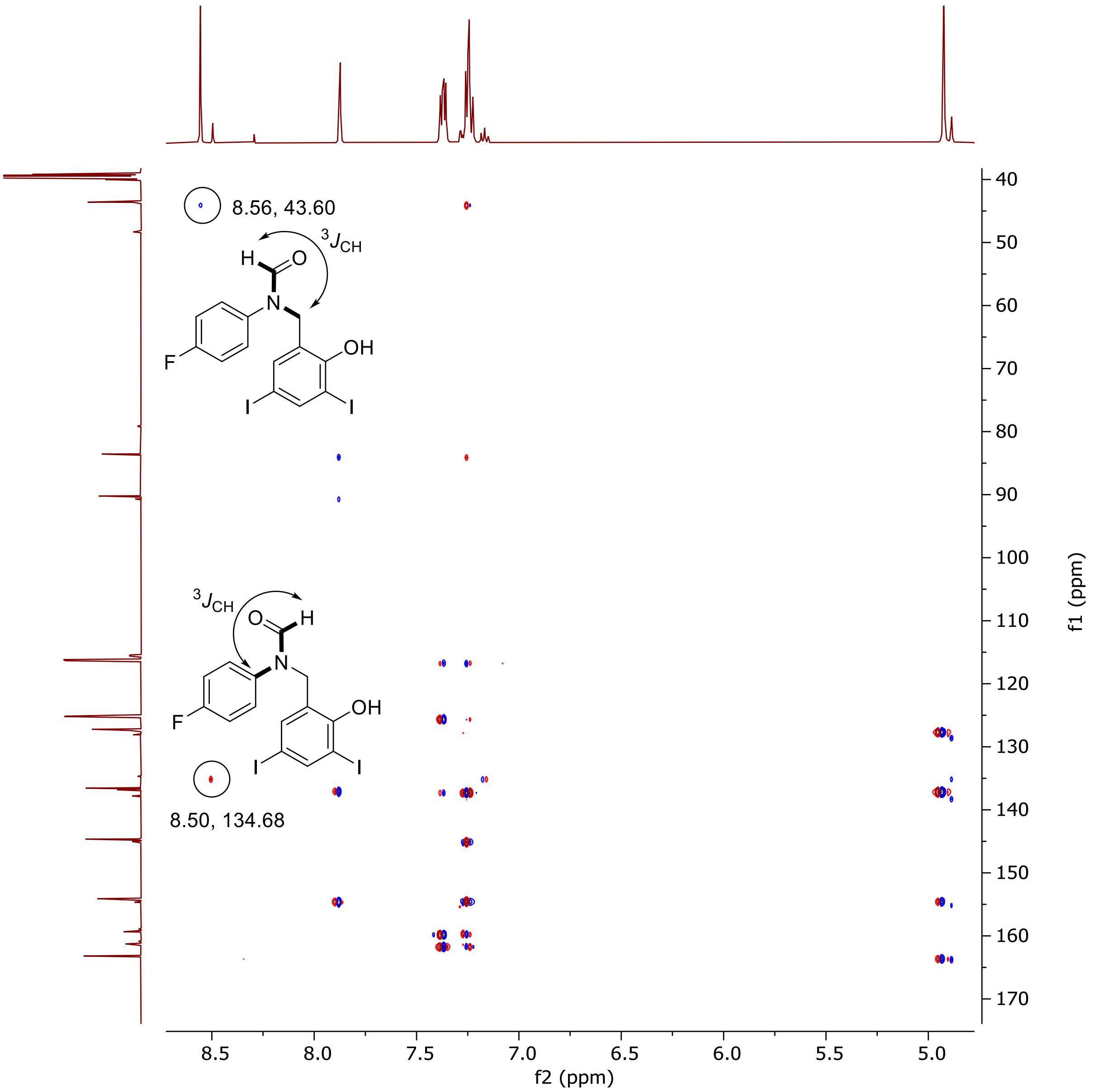

Figure S139: HMBC NMR spectrum (DMSO-d_6) of compound **1**-I_2.

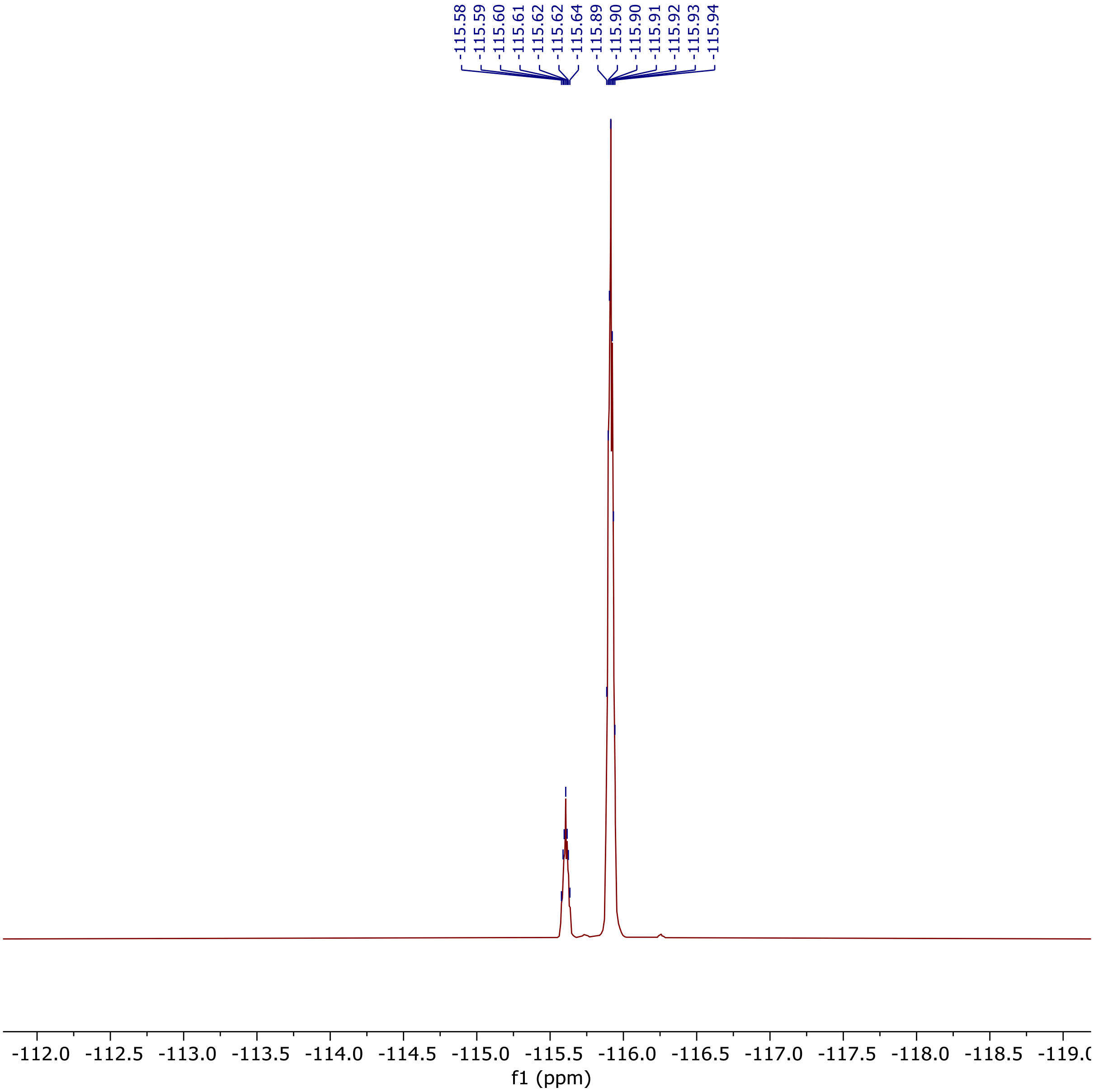

Figure S140: ^{19}F NMR spectrum (DMSO-d_6) of compound **1**-I_2.

References

(1) Charisiadis, P.; Kontogianni, V. G.; Tsiafoulis, C. G.; Tzakos, A. G.; Siskos, M.; Gerothanassis, I. P. 1H-NMR as a Structural and Analytical Tool of Intra- and Intermolecular Hydrogen Bonds of Phenol-Containing Natural Products and Model Compounds. *Molecules* **2014**, *19* (9), 13643–13682.

(2) Jones, R. M.; Van De Water, R. W.; Lindsey, C. C.; Hoarau, C.; Ung, T.; Pettus, T. R. R. A Mild Anionic Method for Generating O-Quinone Methides: Facile Preparations of Ortho-Functionalized Phenols. *J. Org. Chem.* **2001**, *66* (10), 3435–3441.

(3) Xu, J.; Luo, D.-F.; Xiao, B.; Liu, Z.-J.; Gong, T.-J.; Fu, Y.; Liu, L. Copper-Catalyzed Trifluoromethylation of Aryl Boronic Acids Using a CF3+ Reagent. *Chem. Commun.* **2011**, *47* (14), 4300–4302.

(4) *AIST:Spectral Database for Organic Compounds,SDBS.* https://sdbs.db.aist.go.jp/sdbs/cgi-bin/cre_index.cgi (accessed 2024-01-06).

(5) Satkar, Y.; Yera-Ledesma, L. F.; Mali, N.; Patil, D.; Navarro-Santos, P.; Segura-Quezada, L. A.; Ramírez-Morales, P. I.; Solorio-Alvarado, C. R. Iodine(III)-Mediated, Controlled Di- or Monoiodination of Phenols. *J. Org. Chem.* **2019**, *84* (7), 4149–4164.
(6) Abraham, M. H.; Abra, R. J.; Aliev, A. E.; Tormena, C. F. Is There an Intramolecular Hydrogen Bond in 2-Halophenols? A Theoretical and Spectroscopic Investigation. *Phys. Chem. Chem. Phys.* **2015**, *17* (38), 25151–25159.
(7) Li, W.; Gao, G.; Gao, Y.; Yang, C.; Xia, W. Direct Oxidation of the C(Sp2)–C(Sp3) Bond from Benzyltrimethylsilanes to Phenols. *Chem. Commun.* **2017**, *53* (38), 5291–5293.
(8) Smith, D. G. A.; Burns, L. A.; Simmonett, A. C.; Parrish, R. M.; Schieber, M. C.; Galvelis, R.; Kraus, P.; Kruse, H.; Di Remigio, R.; Alenaizan, A.; James, A. M.; Lehtola, S.; Misiewicz, J. P.; Scheurer, M.; Shaw, R. A.; Schriber, J. B.; Xie, Y.; Glick, Z. L.; Sirianni, D. A.; O'Brien, J. S.; Waldrop, J. M.; Kumar, A.; Hohenstein, E. G.; Pritchard, B. P.; Brooks, B. R.; Schaefer, H. F., III; Sokolov, A. Yu.; Patkowski, K.; DePrince, A. E., III; Bozkaya, U.; King, R. A.; Evangelista, F. A.; Turney, J. M.; Crawford, T. D.; Sherrill, C. D. PSI4 1.4: Open-Source Software for High-Throughput Quantum Chemistry. *J. Chem. Phys.* **2020**, *152* (18), 184108..
(9) Parrish, R. M.; Gonthier, J. F.; Corminbœuf, C.; Sherrill, C. D. Communication: Practical Intramolecular Symmetry Adapted Perturbation Theory via Hartree-Fock Embedding. *J. Chem. Phys.* **2015**, *143* (5), 051103.
(10) Yeap, G.-Y.; Ha, S.-T.; Ishizawa, N.; Suda, K.; Boey, P.-L.; Kamil Mahmood, W. A. Synthesis, Crystal Structure and Spectroscopic Study of *Para* Substituted 2-Hydroxy-3-Methoxybenzalideneanilines. *J. Mol. Struct.* **2003**, *658* (1), 87–99.
(11) Khatua, M.; Goswami, B.; Devi, A.; Kamal; Hans, S.; Samanta, S. A Phosphine-Oxide Cobalt(II) Complex and Its Catalytic Activity Studies toward Alcohol Dehydrogenation Triggered Direct Synthesis of Imines and Quinolines. *Inorg. Chem.* **2024**, *63* (21), 9786–9800.
(12) Mitani, M.; Mohri, J.; Yoshida, Y.; Saito, J.; Ishii, S.; Tsuru, K.; Matsui, S.; Furuyama, R.; Nakano, T.; Tanaka, H.; Kojoh, S.; Matsugi, T.; Kashiwa, N.; Fujita, T. Living Polymerization of Ethylene Catalyzed by Titanium Complexes Having Fluorine-Containing Phenoxy−Imine Chelate Ligands. *J. Am. Chem. Soc.* **2002**, *124* (13), 3327–3336.
(13) Cao, C.-T.; Zhou, W.; Cao, C. Abnormal Effect of Hydroxyl on the Longest Wavelength Maximum in Ultraviolet Absorption Spectra for Bis-Aryl Schiff Bases. *J. Phys. Org. Chem.* **2017**, *30* (10), e3672.
(14) Mills, L. R.; Kim, J.; Simmons, E. M.; Wisniewski, S. R.; Chirik, P. J. C(Sp3)–C(Sp3) Reductive Elimination from (Phenoxyimine)Cobalt(III)(CH3)2(PMe3)2 Complexes. *Organometallics* **2024**, *43* (9), 1021–1029.
